# Alternative Splicing and Highly Variable Cadherin Transcripts Associated with Field-Evolved Resistance of Pink Bollworm to Bt Cotton in India

**DOI:** 10.1371/journal.pone.0097900

**Published:** 2014-05-19

**Authors:** Jeffrey A. Fabrick, Jeyakumar Ponnuraj, Amar Singh, Raj K. Tanwar, Gopalan C. Unnithan, Alex J. Yelich, Xianchun Li, Yves Carrière, Bruce E. Tabashnik

**Affiliations:** 1 U.S. Department of Agriculture, Agricultural Research Service, U.S. Arid Land Agricultural Research Center, Maricopa, Arizona, United States of America; 2 National Institute of Plant Health Management, Rajendranagar, Hyderabad, Andhra Pradesh, India; 3 National Centre for Integrated Pest Management, Indian Agricultural Research Institute, New Delhi, Delhi, India; 4 Department of Entomology, University of Arizona, Tucson, Arizona, United States of America; Instituto de Biotecnología, Universidad Nacional Autónoma de México, Mexico

## Abstract

Evolution of resistance by insect pests can reduce the benefits of insecticidal proteins from *Bacillus thuringiensis* (Bt) that are used extensively in sprays and transgenic crops. Despite considerable knowledge of the genes conferring insect resistance to Bt toxins in laboratory-selected strains and in field populations exposed to Bt sprays, understanding of the genetic basis of field-evolved resistance to Bt crops remains limited. In particular, previous work has not identified the genes conferring resistance in any cases where field-evolved resistance has reduced the efficacy of a Bt crop. Here we report that mutations in a gene encoding a cadherin protein that binds Bt toxin Cry1Ac are associated with field-evolved resistance of pink bollworm (*Pectinophora gossypiella*) in India to Cry1Ac produced by transgenic cotton. We conducted laboratory bioassays that confirmed previously reported resistance to Cry1Ac in pink bollworm from the state of Gujarat, where Bt cotton producing Cry1Ac has been grown extensively. Analysis of DNA from 436 pink bollworm from seven populations in India detected none of the four cadherin resistance alleles previously reported to be linked with resistance to Cry1Ac in laboratory-selected strains of pink bollworm from Arizona. However, DNA sequencing of pink bollworm derived from resistant and susceptible field populations in India revealed eight novel, severely disrupted cadherin alleles associated with resistance to Cry1Ac. For these eight alleles, analysis of complementary DNA (cDNA) revealed a total of 19 transcript isoforms, each containing a premature stop codon, a deletion of at least 99 base pairs, or both. Seven of the eight disrupted alleles each produced two or more different transcript isoforms, which implicates alternative splicing of messenger RNA (mRNA). This represents the first example of alternative splicing associated with field-evolved resistance that reduced the efficacy of a Bt crop.

## Introduction

Insecticidal crystalline proteins from the bacterium *Bacillus thuringiensis* (Bt) kill some major insect pests, but are harmless to most non-target organisms including people [1]–[3]. To provide a new tool for pest management, scientists genetically engineered crops to produce Bt proteins for insect control [3]. The area planted to transgenic Bt crops increased from 1 million hectares in 1996 to more than 75 million hectares worldwide in 2013 [4]. These Bt crops can decrease reliance on conventional insecticides, suppress some key pests, and increase yields and farmers' profits [5]–[10]. However, the evolution of resistance to Bt crops by insect pests can diminish such benefits [11]–[13].

Although several mechanisms of resistance to Bt toxins occur, the most common type entails mutations that reduce binding of Bt toxins to larval midgut proteins [2], [14]–[17]. Identification of the genes conferring pest resistance to Bt toxins has been limited to laboratory-selected strains, with three notable exceptions: mutations in an ABCC2 transporter gene are linked with resistance to Cry1Ac in a field-selected strain of *Plutella xylostella* and a greenhouse-selected strain of *Trichoplusia ni* that were derived from populations exposed to sprays containing Cry1Ac [18], and in *Helicoverpa armigera*, mutations in a gene encoding a cadherin protein that binds Cry1Ac are linked with resistance to Cry1Ac in a laboratory-selected strain and in field-selected populations from northern China that were exposed intensively to Bt cotton producing Cry1Ac [19]–[23]. Relative to susceptible populations, the percentage of individuals resistant to Cry1Ac was significantly higher in field populations from northern China, yet it was less than 5% as of 2010 and reduced efficacy of Bt cotton producing Cry1Ac has not been reported there [22], [24].

By contrast with the knowledge of genes responsible for many examples of laboratory-selected resistance and the three examples of field- and greenhouse-selected resistance described above, the genes conferring resistance to Bt toxins have not been identified for any of the first five cases in which reduced efficacy of Bt crops is associated with field-evolved resistance [13], [25]–[29]. Here we examined the genetic basis of resistance for one of these five cases: field-evolved resistance to Bt cotton producing Cry1Ac in India by pink bollworm (*Pectinophora gossypiella*), which is a global pest of cotton [13], [29]–[31].

In India, which grew more hectares of Bt cotton than any other country in the world in 2012 and 2013 [4], [32], Bt cotton hybrids producing Cry1Ac were commercialized in 2002 [33]. However, Bt cotton was planted illegally before 2002 in the state of Gujarat, which leads India in cotton production and typically produces a third of the nation's cotton [33]–[35]. The estimated mean percentage of all cotton hectares planted with Bt cotton from 2003 to 2007 was 75% (range  = 54 to 90%) in Gujarat, compared with 30% (range  = 2 to 73%) in Maharashtra, India's second leading cotton-producing state [33]–[34].

Pink bollworm resistance to Cry1Ac was documented with diet bioassays showing that mean survival at a diagnostic toxin concentration was 72% for a population sampled in 2008 from the district of Amreli in Gujarat, compared with 0 to 4% for populations from four sites outside of Gujarat including Akola in Maharashtra [29]. Monsanto (2010) also reported “unusual survival of pink bollworm” on Bt cotton producing Cry1Ac during 2009 and “confirmed” pink bollworm resistance to Cry1Ac in four districts of Gujarat: Amreli, Bhavnagar, Junagarh and Rajkot [30]. Farmers in India have switched to cotton hybrids producing two Bt toxins (Cry1Ac and Cry2Ab), which are effective against pink bollworm larvae resistant to Cry1Ac [29]–[30], [36]–[37]. These two-toxin hybrids were planted on 10.4 million hectares in 2013, representing 94% of India's cotton [4].

We hypothesized that field-evolved resistance to Cry1Ac of pink bollworm in India is associated with mutations in a cadherin gene called *PgCad1*, because resistance to Cry1Ac is linked with mutations in this gene for five laboratory-selected strains of pink bollworm from Arizona in the southwestern United States [38]–[42]. Unlike the situation in India, pink bollworm field populations in Arizona have remained susceptible to Cry1Ac despite more than 16 years of extensive exposure to Bt cotton producing this toxin [9], [43]–[44]. From 1996–2005, the main factors that delayed pink bollworm resistance in Arizona appear to be abundant refuges of non-Bt cotton, recessive inheritance of resistance, fitness costs associated with resistance and incomplete resistance [43], [44]. Since 2006, an eradication program using mass releases of sterile pink bollworm moths and other tactics in combination with up to 98% adoption of Bt cotton statewide has dramatically suppressed this pest in Arizona [9], [44]. In contrast, lack of compliance with the refuge strategy apparently promoted rapid evolution of pink bollworm resistance to Cry1Ac in India [44]–[46]. Despite the absence of field-evolved resistance of pink bollworm to Bt cotton in the United States, our previous work identified four recessive cadherin alleles (*r1*, *r2*, *r3*, and *r4*) of *PgCad1* linked with resistance to Cry1Ac in laboratory-selected strains from Arizona [38]–[42].

In this study of pink bollworm from India, we detected none of the four cadherin resistance alleles from Arizona, but we discovered eight novel, severely disrupted cadherin alleles associated with resistance to Cry1Ac. Analysis of messenger RNA (mRNA) from these eight alleles revealed 19 transcript isoforms. Each of these 19 transcript isoforms has a premature stop codon, a deletion of at least 99 base pairs (bp), or both. For seven of the eight disrupted cadherin alleles, we detected two or more mRNA transcripts produced by a single allele, which indicates alternative splicing of pre-cursor mRNA (pre-mRNA) [47]–[48].

## Results

### 2.1 Larval Survival in Diet Bioassays with Cry1Ac

**Figure 1:**
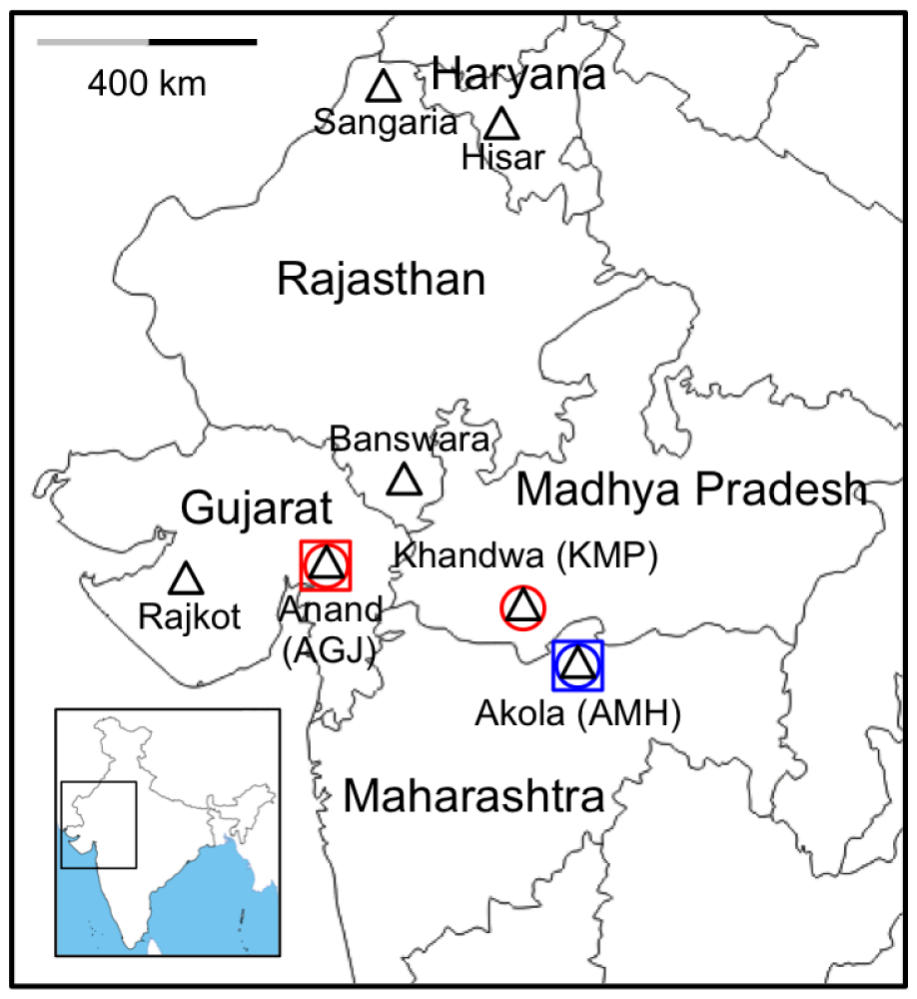
Sampling locations for pink bollworm field populations in India. We screened DNA of 425 pink bollworm collected from all seven sites for cadherin resistance alleles *r1*, *r2*, and *r3* (triangles). We sequenced cadherin cDNA and gDNA of 11 larvae from three sites: Akola (AMH), Anand (AGJ), and Khandwa (KMP) (circles) and conducted bioassays with 130 larvae from two sites: AMH and AGJ (squares). Based on cadherin DNA sequences (circles) and bioassay data (squares) from this study, red indicates evidence of resistance for AGJ and KMP; blue indicates evidence of susceptibility for AMH. Resistance was reported previously from four districts of Gujarat including Rajkot [29]–[30].

We used diet incorporation bioassays with a diagnostic concentration (10 micrograms Cry1Ac per ml diet) [49] to evaluate resistance to Cry1Ac of the first-generation progeny of pink bollworm collected from the field during the 2010–2011 growing season from Anand in Gujarat (AGJ) and from Akola in Maharashtra (AMH) (Fig. 1). We obtained F1 larvae from AGJ parents collected from Bt Cry1Ac cotton whereas the AMH parents were from non-Bt cotton. Larval survival adjusted for control mortality was 65% for AGJ (n = 17 treated and 10 control larvae) and 0% for AMH (n = 43 treated and 60 control larvae) (Fisher's exact test, P<0.0001). These results indicate that a substantial proportion of the AGJ population was resistant to Cry1Ac, whereas the AMH population was predominantly susceptible.

### 2.2 DNA Screening of Populations from India for Cadherin Resistance Alleles from Arizona

We used established PCR methods to screen the genomic DNA (gDNA) of pink bollworm from India for three cadherin alleles that are linked with laboratory-selected resistance to Cry1Ac in pink bollworm from Arizona (*r1*, *r2* and *r3*) [38], [50]–[51]. We found none of these three cadherin alleles in 425 pink bollworm collected during 2010 and 2011 from seven sites in India (Fig. 1, Table S1). The sample from India screened for *r1*, *r2* and *r3* included 46 individuals from two resistant populations in Gujarat: 19 from AGJ, where resistance was detected in our bioassay (described above); and 27 from Rajkot, where resistance was reported previously [30]. In addition, the screened samples included 38 individuals from Khandwa in the state of Madhya Pradesh (KMP) that were collected as fourth instars on Bt cotton and were expected to be predominantly resistant. These results indicate that cadherin resistance alleles *r1*, *r2* and *r3* from Arizona were not common in India, even in samples expected to have a high proportion of individuals resistant to Cry1Ac.

### 2.3 Cadherin DNA and Transcripts from Resistant and Susceptible Larvae

To determine if resistance to Cry1Ac in pink bollworm from India was associated with cadherin mutations different from those identified in Arizona, we sequenced cadherin gDNA and cDNA of larvae preserved in RNAlater from three sources: AMH, AGJ, and KMP. Based on 0% survival of AMH larvae at a diagnostic concentration of Cry1Ac, we inferred that the AMH larvae were susceptible (as described above). We analyzed DNA from three AGJ larvae that we identified as resistant because they became fourth instars while feeding on diet containing a diagnostic concentration of Cry1Ac. We also analyzed DNA from five individuals from KMP that we expected to be predominantly resistant because they were collected as second and third instars from bolls in Bt cotton fields.

**Figure 2:**
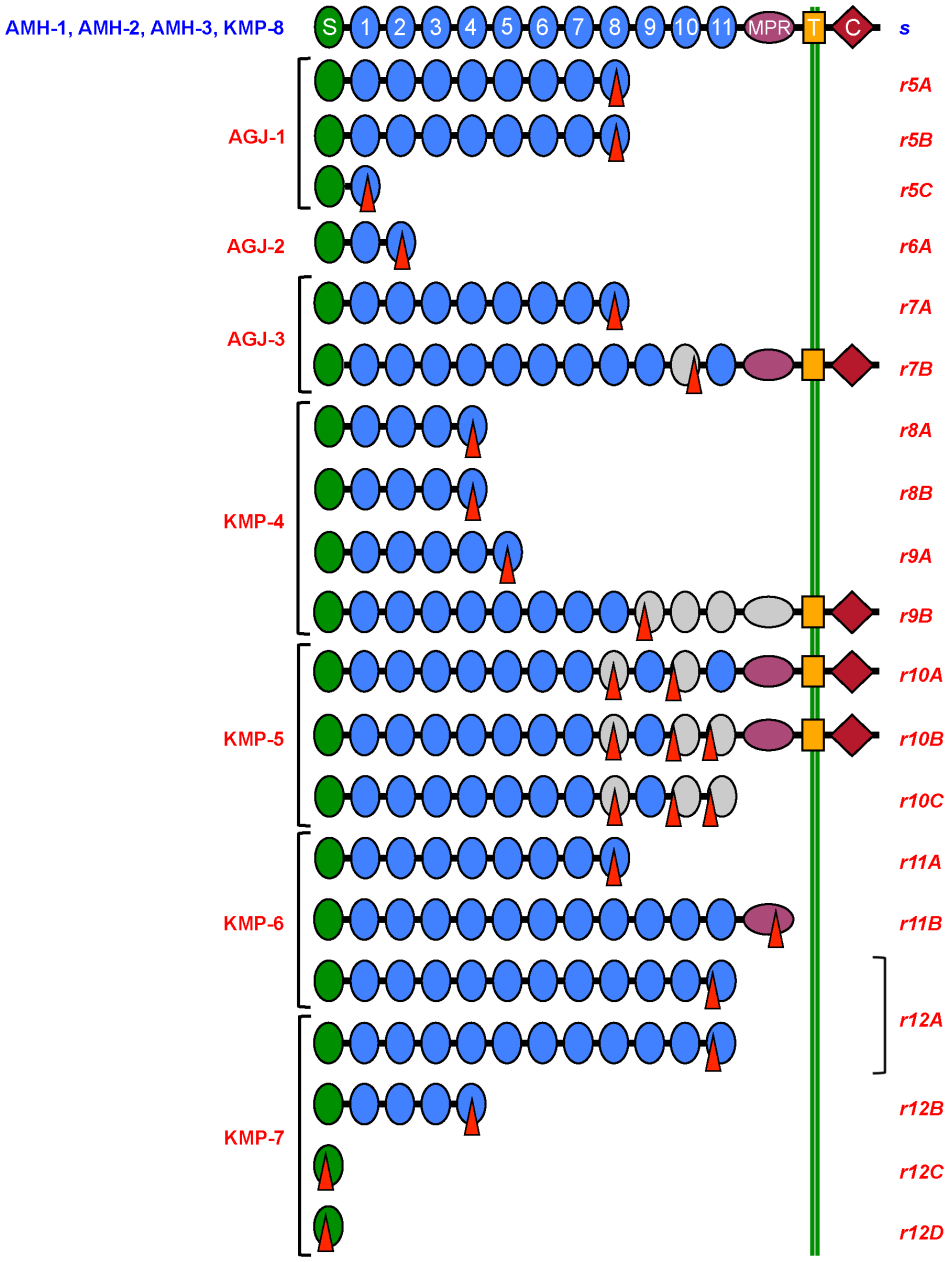
Predicted cadherin proteins in pink bollworm from three populations in India. We isolated and sequenced full-length *PgCad1* cDNA clones from 11 individuals: three from Akola, Maharashtra (AMH-1 to AMH-3), three from Anand, Gujarat (AGJ-1 to AGJ-3), and five from Khandwa, Madhya Pradesh (KMP-4 to KMP-8). Predicted proteins are shown for cDNA of the *PgCad1* susceptible (*s*) allele and 19 isoforms (*r5A, r5B,* etc.) of mutant alleles *r5*–*r12*. The amino-terminal membrane signal sequence (S), cadherin repeats (1–11), membrane-proximal region (MPR), transmembrane region (T), and cytoplasmic domain (C) are shown for the *s* allele. Red triangles indicate mutations predicted to cause loss of at least 33 amino acids (see Table 1). Truncated structures indicate proteins predicted from cDNA with premature stop codons. Gray indicates missing regions of proteins caused by deletions. The 3-bp deletion (corresponding to bp 72–74 in the *s* allele) that occurred in one sequence from AMH-3 and four sequences from KMP-8 as well as in two sequences from AGJ-1 and one sequence from KMP-7 is not shown.

Sequencing revealed no severe disruptions in the cDNA of cadherin from the three susceptible larvae from AMH (Fig. 2, Fig. S1), whereas severe disruptions occur in all three of the cadherin alleles from the resistant AGJ larvae, and in 5 of the 6 alleles from the KMP larvae that were collected from Bt cotton (Table 1, Fig. 2). In the eight larvae analyzed from AGJ and KMP, we found eight novel, severely disrupted cadherin alleles (*r5*–*r12*) with a total of 19 different cDNA sequences (Table 1, Fig. 2). Seven of these eight alleles have at least two transcript isoforms, which implicates alternative splicing of these alleles (Table 1, Fig. 2).

**Table 1:** Nineteen transcript isoforms of eight disrupted cadherin alleles in seven pink bollworm larvae from two populations in India: Anand, Gujarat (AGJ) and Khandwa, Madhya Pradesh (KMP).

Indivi-dual(s)	Allele	Iso-form	Deletion size(s) (bp)^a^	Inser-tion size (bp)^a^	Cadherin region^b^	Pre-mature stop codon(s)	Complete exon(s) missing
AGJ-1	*r5*	*r5A*	**478** c	-	CR8-9	Yes	21–24
AGJ-1	*r5*	*r5B*	3^d^, **478** c	-	CR8-9	Yes	21–24
AGJ-1	*r5*	*r5C*	478^c^	**20**	Signal-CR1	Yes	21–24
AGJ-2	*r6*	*r6A*	**1051** e	-	CR2-5	Yes	8–13
AGJ-3	*r7*	*r7A*	**247**	-	CR8-9	Yes	21–22
AGJ-3	*r7*	*r7B*	99	-	CR10	No	27
KMP-4	*r8*	*r8A*	**170**	4	CR4-5	Yes	13
KMP-4	*r8*	*r8B*	-	**4**	CR4	Yes	No
KMP-4	*r9*	*r9A*	165^f^	-	CR5, CD	Yes	32
KMP-4	*r9*	*r9B*	1157	-	CR9-MPR	No	23–31
KMP-5	*r10*	*r10A*	126, 105	-	CR8, 10	No	21, 25
KMP-5	*r10*	*r10B*	126, 105, 303	-	CR8, 10, 11	No	21, 25, 28–29
KMP-5	*r10*	*r10C*	126, 105, **193**	-	CR8, 10, 11	Yes	21, 25, 28
KMP-6	*r11*	*r11A*	**23** g	127	CR8, MPR	Yes	No
KMP-6	*r11*	*r11B*	-	**125**	MPR	Yes	No
KMP-6 KMP-7	*r12*	*r12A*	-	**1** h	CR11	Yes	No
KMP-7	*r12*	*r12B*	3^d^, **118** i	1^h^	CR4, 11	Yes	11
KMP-7	*r12*	*r12C*	**11** j, 148^k^	1^h^	Signal, CR11	Yes	5
KMP-7	*r12*	*r12D*	**11** j, 230^l^	1^h^	Signal, CR4, 11	Yes	11–12

a Mutations shown in bold cause premature stop codons.

b Region of cadherin protein where major mutations occur (see Figure 2).

c The 478-bp deletion found in *r5A*, *r5B* and *r5C* is caused by insertion of 3,120 bp similar to transposons (Table 2), causing the loss of exons 21–24 from gDNA and cDNA.

d The 3-bp deletion in *r5B* and *r12B* is caused by mis-splicing, occurs at exon-intron splice junction 1, and is found in both *r* and *s PgCad1* alleles.

e gDNA from AGJ-2 was not available to compare with cDNA, but the absence of exons 8–13 occurs exactly at the exon-intron junctions, suggesting that mis-splicing occurred.

f *r9A* includes A-to-G (I) RNA editing at base position 2,289 and results in the introduction of a premature stop codon (see Fig. 4). The 165-bp deletion causes the loss of exon 32.

g The 23-bp deletion corresponds to the final 23 nucleotides of exon 20 in cDNA clone KMP-6_3.

h The single base insertion introduces a premature stop codon and truncates the mRNA transcript in CR11.

i The 118-bp deletion causes the loss of exon 11 resulting in the introduction of a premature stop codon and truncates the mRNA transcript in CR4.

j The 11-bp deletion occurs in the membrane signal sequence of *r12C* and *r12D* transcripts resulting in the introduction of a premature stop codon.

k The 148-bp deletion causes the loss of exon 5 in mRNA transcript between the membrane signal sequence and CR1.

l The 230-bp deletion causes the loss of exons 11–12 in mRNA transcript found in CR4.

As expected for susceptible pink bollworm [38], cadherin cDNA isolated from three susceptible AMH larvae had 5,208 bp encoding a predicted protein of 1,735 amino acids (Fig. S1, Fig. 2). The predicted open reading frame (ORF) for the consensus AMH cDNA has 99% homology with the translated sequence from the *PgCad1 s* allele (AY198374.1) from the susceptible APHIS-S strain of pink bollworm from Arizona [38]. As with the *s* allele from Arizona, the translated protein encoded by cDNA from AMH includes a putative membrane signal sequence, 11 extracellular cadherin repeats (CR1-CR11), a membrane-proximal region, a transmembrane domain, and a cytoplasmic domain (Fig. 2).

Eight of the nine complete cDNA sequences we obtained from three susceptible AMH larvae have no insertions or deletions (indels) (Fig. S1). In the exceptional sequence from one AMH individual, we found a single deletion of 3 bp corresponding to nucleotides 72–74 of the *s* allele from Arizona encoding alanine in the membrane signal sequence (sequence AMH-3_16, Figs. S1 and S2). This deletion was also detected in one larva from AGJ (AGJ-1, Table 1) and two larvae from KMP (KMP-7 and KMP-8, see details below). We also identified 195 putative single nucleotide polymorphisms (SNPs) in the full-length cDNA sequences from AMH (Fig. S1). Of the 96 putative SNPs encoding amino acid changes, 52 are conservative substitutions (Fig. S2). Several missense mutations (e.g., Leu/His1274, Asp/Gly1371, Glu/Gly1381and Arg/Gly1469) occur in CR10-CR11, the region involved in binding Cry1Ac in pink bollworm [52]. However, we found no insertions, deletions, or missense mutations in the specific portions of these domains that bind Cry1Ac in pink bollworm [52].

**Figure 3:**
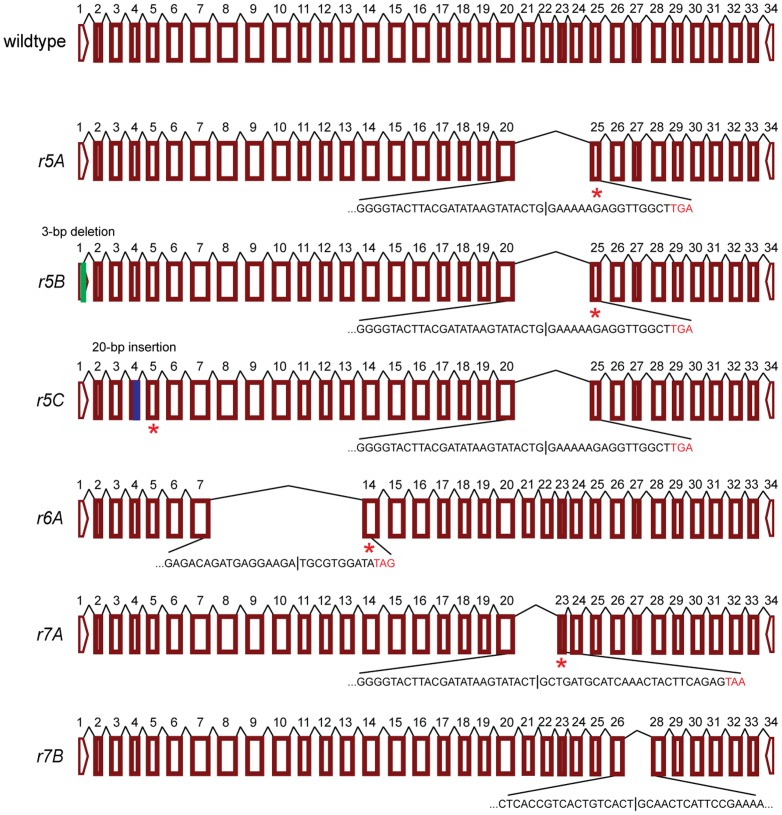
Cadherin mRNA transcripts of a susceptible allele and three severely disrupted alleles found in three resistant pink bollworm larvae from Anand, Gujarat (AGJ). Exons are numbered (1–34). Sequences are shown for exons missing from transcripts. Blue boxes show insertions, green boxes show deletions, and stars show premature stop codons. The six transcript isoforms shown are *r5A*-*r7B* (GenBank accession KJ480757-KJ480762).

In contrast with the conserved cadherin cDNA sequences from susceptible AMH larvae, the cadherin cDNA sequences from three resistant AGJ larvae are highly variable and severely disrupted (Table 1, Figs. 2, 3, and S3). In three AGJ larvae, we found three novel cadherin alleles (*r5, r6,* and *r7*; Table 1 and Fig. 2). Two of these three alleles have multiple isoforms (e.g., *r5A, r5B,* and *r5C* of allele *r5*) yielding a total of six isoforms (Table 1, Figs. 2 and 3). Five of these six isoforms have premature stop codons; the sixth isoform (*r7B*) has a 99-bp deletion encoding a cadherin protein that lacks the entire CR10 (Table 1, Fig. 2).

**Figure 4:**
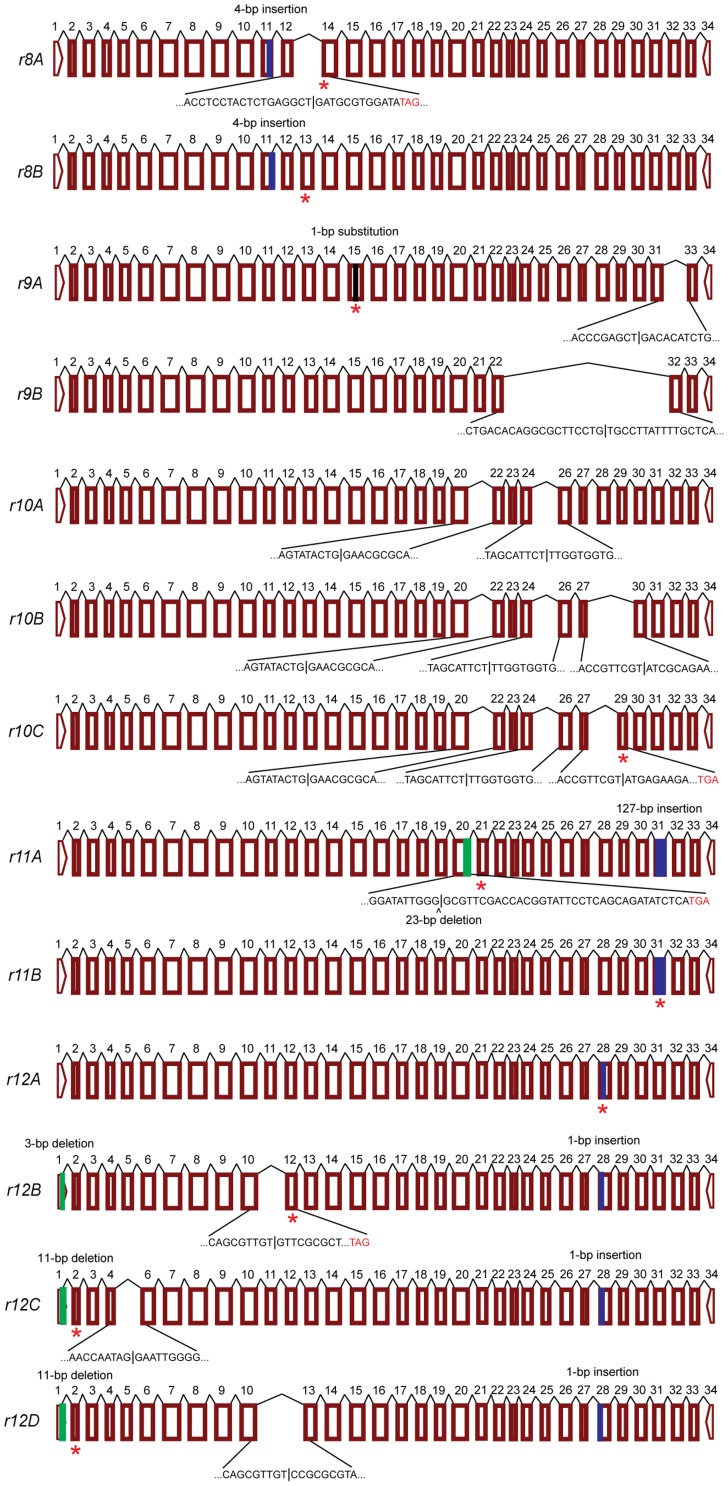
Cadherin mRNA transcripts from five severely disrupted alleles found in four pink bollworm larvae collected on Bt cotton in Khandwa, Madhya Pradesh (KMP). Transcript isoforms of alleles *r8*–*r12* from individuals KMP-4, KMP-5, KMP-6, and KMP-7. Exons are numbered. Sequences are shown for exons missing from transcripts. Blue boxes show insertions, green boxes show deletions, black boxes show substitutions, and stars show premature stop codons. The 13 transcript isoforms shown are *r8A*-*r12D* (GenBank accession KJ480763-KJ480775).

The cadherin cDNA sequences are also highly variable and severely disrupted in four of the five larvae from KMP (Table 1, Figs. 2, 4 and S5), which were collected from Bt cotton and expected to be resistant (the fifth larva is described below). These four KMP larvae carried a total of five different disrupted cadherin alleles (*r8*–*r12*). Two of these four larvae each had two different disrupted alleles (alleles *r8* and *r9* in individual KMP-4 and alleles *r11* and *r12* in individual KMP-6, Table 1). Each of the five mutant cadherin alleles in KMP has two to four isoforms, yielding 13 isoforms in four larvae (Table 1 and Figs. 2 and 4). In each of the five mutant KMP alleles, we identified one or more indels of 1 to 1,157 bp, with 10 of the 13 isoforms bearing indels that introduce premature stop codons (Table 1, Fig. 2). In addition, cDNA from isoform *r9A* has a single base substitution (guanine 2,289 to adenine) that introduces a premature stop codon. Of the three disrupted KMP isoforms lacking a premature stop codon (*r9B*, *r10A*, and *r10B*), *r10A* and *r10B* shared deletions of 126 and 105 bp; *r10B* also had a third deletion of 303 bp (Table 1). The *r9B* isoform has the largest deletion identified: 1,157 bp corresponding to the portion of the cadherin protein from CR9 to the membrane-proximal region (Table 1, Fig. 2).

Unlike the cDNA sequences from the four KMP larvae described above, none of the five cDNA sequences obtained from five different clones isolated from one KMP larva (KMP-8) are severely disrupted by indels or substitutions (Figs. 2, S5, and S6). Of the two deletions in KMP-8 (Fig. S5), one is the same 3-bp deletion found in one sequence from the susceptible larva AMH-3 (Fig. S1, Fig. S2). The second is the 3-bp deletion corresponding to bases 1,008–1,010 in the *s* allele from Arizona encoding glutamate in CR2 (sequence KMP-8_35; Figs. S5 and S6). Both of these deletions result from alternative mRNA splicing, as they both occur at exon-intron splice junctions and are not present in gDNA. The consensus ORF from KMP-8 has 5,205 bp encoding 1,734 amino acids and shares 99% identity with the *PgCad1 s* allele (AY198374.1) (Fig. S5, Fig. S6). Although the cDNAs from AMH-1, AMH-2, AMH-3, and KMP-8 are not severely disrupted, the 14 cDNA sequences from these four individuals have 27 informative SNPs corresponding to seven unique *s* alleles (Fig. S7). Even with these 27 informative SNPs, no more than two alleles are evident from each single diploid individual. Each of these seven *s* alleles from India shares >99% identity with the PgCad1 *s* allele from Arizona (AY198374.1).

In total, fifteen of the 19 transcript isoforms of the eight severely disrupted alleles have deletions corresponding to the complete loss of one or more exons (Table 1). This includes *r6A*, the only transcript we detected for allele *r6*, which contains a premature stop codon in its cDNA and lacks exons 8–13 (Table 1, Fig. 3, Fig. S3). Although we were not able to obtain gDNA for allele *r6*, the deletion of exons 8–13 in the *r6A* transcript occurs exactly at the exon-intron junctions (Fig. S3 and Fig. S4). Thus, we suspect that mis-splicing, which entails a mistake in splicing [47], causes the disruption in transcript *r6A* in this allele. Mis-splicing is also implicated in the 3-bp deletion found in cDNA but not gDNA from larvae in each of the three populations studied (AMH-3, AGJ-1, KMP-7, and KMP-8) (Table 1 and Figs. S1, S3, and S5).

In addition to a 20-bp insertion that occurs only in the *r5C* isoform and reflects alternative splicing, the gDNA of *r5* and all three isoforms of *r5* have an insertion of 3,120 bp that causes the loss of exons 21–24 (Table 1, Fig. 3, Fig. S8). Thus, this 3,120-bp insertion reflects mis-splicing rather than alternative splicing. A CENSOR search of Repbase [53] reveals that this insert is similar to several transposable elements (Table 2). Several smaller insertions that introduce premature stop codons also occur in both cDNA and gDNA and do not reflect altered splicing (four bp in *r8*, 125 to 127 bp in *r11*, and one bp in *r12*; Table 1, Table S2, Fig. 4, Fig. S8).

**Table 2:** Similarity between transposons and the insertion in intron 20 of the *r5 PgCad1* allele.

Position in insertion (bp)^a^	Repbase transposon name^b^	Position in transposon (bp)^c^	Transposon class	Orientation^d^	Sim^e^	BLAST score^f^
524–619	LYDIA_LTR	205–300	LTR/Gypsy	comp.	0.71	229
1,580–1,737	TED	1–162	LTR/Gypsy	comp.	0.75	609
2,332–2,432	CoeSINE4	81–178	NonLTR/SINE/SINE2	comp.	0.78	306
2,449–2,489	HaSE3	112–152	NonLTR/SINE/SINE3	comp.	0.83	237
2,587–2,651	HATN3_DR	274–338	DNA/hAT	comp.	0.73	280
3,146–3,197	Transib–4_DBp	2,848–2,899	DNA/Transib	direct	0.83	213
3,568–3,660	ISL2EU–3_HM	1,655–1,746	DNA/ISL2EU	direct	0.74	207

a Nucleotide position in the 3,827-bp fragment from pink bollworm cadherin (which includes the 3,120-bp insertion in the *r5* allele) cloned from AGJ-1 gDNA using primers 20PgCad5 + 81PgCad3 (See Figure S8).

b LYDIA_LTR, long terminal repeat retrotransposon from LYDIA, a gypsy-like endogenous retrovirus from *Lymantria dispar*; TED, internal part of retrotransposon TED inserted in *Autographa californica* nuclear polyhedrosis virus; CoeSINE4, coelacanth SINE non-long terminal repeat retrotransposon from *Latimeria chalumnae*; HaSE3, SINE non-long terminal repeat retrotransposon from *Helicoverpa armigera*; HATN3_DR, nonautonomous DNA transposon from *Danio rerio*; Transib-4_DBp, Transib-type DNA transposon from the *Drosophila bipectinata* genome; ISL2EU-3_HM, autonomous ISL2EU DNA transposon from *Hydra magnipapillata*.

c Nucleotide position in the transposon sequence.

d Orientation of the insertion sequence relative to the corresponding sequence in the transposon; comp. indicates complementary.

e Similarity between the fragment sequence and the corresponding sequence in the transposon; calculated as the number of exact matches/(alignment length - total length gaps in the fragment sequence - total length of gaps in the transposon sequence + total number of gaps).

f Alignment score from BLAST.

## Discussion

The bioassay results here with pink bollworm derived from the field in India during 2010 and 2011 show 65% of individuals resistant to Cry1Ac in the Anand population from Gujarat (AGJ) compared with 0% in the Akola population from Maharashtra (AMH). These results confirm previous reports from 2008 and 2009 indicating pink bollworm resistance to Cry1Ac in Gujarat, where Bt cotton was adopted rapidly, but not in Akola, where adoption was much slower [29]–[30], [33].

Whereas previous results show that resistance to Cry1Ac in laboratory-selected strains of pink bollworm from Arizona is linked with mutations in a gene encoding a cadherin protein that binds Cry1Ac in the larval midgut [38]–[42], the data here show an association between field-evolved resistance to Cry1Ac in India and different mutations in the same gene. In the susceptible AMH population, none of the cadherin DNA sequences from three larvae were severely disrupted. By contrast, all of the cadherin DNA sequences were severely disrupted in the three resistant larvae from AGJ that survived exposure to a diagnostic concentration of Cry1Ac.

Among five individuals from Khandwa in Madhya Pradesh (KMP) collected as second or third instars from Bt cotton and expected to be predominantly resistant, four had only severely disrupted cadherin alleles and the fifth had no disrupted cadherin alleles. We cannot exclude the hypothesis that the fifth larva from KMP was susceptible, because we did not determine the concentration of Cry1Ac in the bolls on which the field-collected larvae fed and cannot rule out the possibility that the fifth larva fed on plant tissues with a reduced concentration of Cry1Ac. We also cannot exclude an alternative hypothesis that the fifth KMP larva was resistant, with the resistance conferred by a gene other than cadherin. Although cadherin mutations are sufficient to cause resistance to Cry1Ac in pink bollworm, mutations at other loci also can confer resistance to this toxin in pink bollworm and other Lepidoptera [18], [54]–[60].

In eight larvae from the field-selected populations AGJ and KMP, we discovered eight novel, severely disrupted cadherin alleles (*r5*–*r12*) with a total of 19 novel cDNA isoforms (Table 1 and Fig. 2). Among the 19 isoforms, 15 have premature stop codons and the other four have one or more deletions of at least 99 bp in the sequence encoding the Cry1Ac-binding region (Table 1, Fig. 2). The premature stop codons are expected to yield truncated cadherin proteins that are not anchored in the midgut membrane and cannot mediate toxicity of Cry1Ac. The predicted omission of at least 33 amino acids from the Cry1Ac-binding region of cadherin protein could also reduce binding of Cry1Ac and thus confer resistance to this toxin. In contrast with these severely disrupted alleles from India, among the four pink bollworm cadherin resistance alleles from Arizona, only *r2* has a deletion (202 bp) that introduces a premature stop codon [38], [42] and each of the other three (*r1, r3,* and *r4*) has only a single deletion (24, 126 and 15 bp, respectively) that does not occur in the sequence encoding the Cry1Ac-binding region [38], [42]. Given that the relatively minor disruptions in three of four cadherin alleles of pink bollworm from Arizona are genetically linked with resistance to Cry1Ac, we conclude that the severe disruptions in the eight cadherin alleles in pink bollworm from India probably confer resistance to Cry1Ac.

Although mutations in the same cadherin gene are associated with pink bollworm resistance to Cry1Ac in laboratory-selected strains from Arizona and field-selected populations from India, we did not find any of the four cadherin resistances alleles from Arizona in the 436 pink bollworm from India that we analyzed. These include 425 individuals from seven populations screened for alleles *r1, r2* and *r3* and 11 individuals from AMH, AGJ and KMP from which we sequenced cadherin cDNA. The difference in cadherin resistance alleles between Arizona and India could reflect the respective geographic origins from which the pink bollworm were derived, as well as laboratory versus field selection. With highly variable cadherin in the AGJ and KMP populations from India, we also found no resistance alleles in common between these two field-selected populations separated by ca. 400 km, and only one resistance allele that occurred in two individuals within a population from India (*r12* in KMP-6 and KMP-7, Table 1). Given the high diversity of cadherin resistance alleles within each population, it is surprising that all three AGJ individuals and three of the five KMP individuals were homozygous for disrupted alleles at the cadherin locus (Table 1). This pattern may reflect assortative mating, because random mating would generate a higher frequency of individuals carrying two different resistance alleles.

To our knowledge, the two or more transcript isoforms associated with seven of the eight severely disrupted cadherin alleles from India (Table 1) represent the first examples of alternative splicing associated with resistance to a Bt toxin. Although alternative splicing generated five cadherin isoforms in a Cry1Ac-resistant strain of *T. ni* [61], resistance in this strain is genetically linked with the ABCC2 gene, and is not associated with variation in either the transcripts or gDNA for cadherin [18], [55]. However, mutations in cadherin gDNA of pink bollworm and *H. armigera* that cause mis-splicing and produce a single altered transcript linked with resistance to Cry1Ac have been reported [62]–[63]. In *H. armigera*, four different indels in gDNA yield the same altered cDNA transcript that lacks exon 32 [63]. For the previously characterized pink bollworm cadherin resistance allele *r3*, insertion of a non-LTR chicken-repeat retrotransposon (*CR1-1_Pg*) causes splicing out of exon 21 from mRNA [62]. Here we found that loss of exons 21-24 in all three isoforms of the pink bollworm *r5* allele is caused by a 3,120-bp insertion that has sequences similar to several transposons (Table 2).

Because we found eight different cadherin resistance alleles and 19 variant isoforms in only eight pink bollworm larvae from two field-selected populations in India, we expect that larger sample sizes from these and other field-selected populations in India would reveal even more genetic variation at the pink bollworm cadherin locus. To put the diversity of pink bollworm cadherin from India in perspective, we note that only 22 cadherin resistance alleles have been reported previously based on more than a decade of work by several research teams analyzing thousands of individuals representing three major cotton pests. These previously reported cadherin resistance alleles consist of the four in pink bollworm from Arizona [38], [42], one in *H. virescens* from the southeastern United States [64], and 17 in *H. armigera* from northern China and western India [19]–[23], [63], [65]–[66]. Mis-splicing was reported for one cadherin resistance allele from pink bollworm [62] and another from *H. armigera* [63], as noted above, but not for the other previously reported cadherin alleles. Genetic variation in cadherin that is not associated with resistance has also been reported in other pests [59], [67]–[69].

Whereas severe disruptions occurred in all three of the cadherin alleles from the resistant AGJ larvae and in 5 of the 6 alleles from the KMP larvae collected from Bt cotton, we found no severe disruptions in the cDNA of cadherin from the three susceptible larvae from AMH. Likewise, our previous work with pink bollworm from Arizona revealed four disrupted cadherin alleles linked with resistance to Cry1Ac in laboratory-selected strains and no such disruptions in susceptible insects [38]–[42]. These results suggest that in the AGJ and KMP populations, the high genetic variation in cadherin and the high frequency of disrupted cadherin alleles reflect selection of these populations in the field for resistance to Bt cotton producing Cry1Ac. We hypothesize that fitness costs, which have been identified for cadherin resistance alleles of pink bollworm from Arizona [40]–[41], [70]–[74], keep the frequency of such alleles low in the absence of selection for resistance.

Similar to the results with pink bollworm, the only other comparison reported between the molecular genetic basis of laboratory- and field-selected resistance to a Bt toxin in a transgenic crop shows cadherin resistance alleles linked with resistance to Cry1Ac selected in both environments for *H. armigera* from northern China [22]. In northern China, the *r1* cadherin resistance allele of *H. armigera*, which includes a premature stop codon and was first detected in a laboratory-selected strain derived in 2001 [19], was also found in three independently isolated resistant strains initiated in 2009 from the field-selected Anyang population in Henan province [22]. In that case, the collection sites for the laboratory- and field-selected populations are separated by only 300 km.

Also similar to the results with pink bollworm in India, previous studies identified 15 cadherin resistance alleles from four populations of *H. armigera* in China [20]–[23]. Only two of these 15 alleles were found in more than one individual within a population (*r1* from Anyang and *r8* from Jiangpu) [20] and only one allele (*r15*) was detected in more than one population [23], [63].

The diversity of cadherin mutations associated with resistance to Cry1Ac in field-selected populations of pink bollworm in India and *H. armigera* in China implies that it would not be efficient to monitor resistance in these populations by screening cadherin DNA for specific resistance alleles, as was done previously in the United States for pink bollworm and *H. virescens* [9], [51], [75]. An alternative approach that would detect any resistance alleles at the cadherin locus, as well as non-recessive resistance alleles at any locus, is the F1 screen in which field-collected adults are allowed to mate in single pairs with adults from a strain that is homozygous for a recessive cadherin mutation [20]–[22], [76]–[77]. In general, laboratory-selected strains that are homozygous for recessive resistance alleles at any locus can be used in this way to screen field populations for recessive resistance alleles at the same locus, even if the gene is not identified and the alleles differ between the lab- and field-selected populations [76]–[77].

Generation of resistance alleles by alternative splicing, as seen in seven of eight cadherin resistance alleles from India (Table 1), can reduce the feasibility of resistance monitoring with DNA screening not only by increasing the diversity of transcripts, but also by making it necessary to analyze mRNA, which requires better sample preservation and more steps than screening gDNA. Alternative splicing may also accelerate evolution of resistance by generating a greater diversity of mutations that include altered proteins conferring higher levels of resistance, lower fitness costs associated with resistance, or both.

Mutations affecting splicing of mRNA are pervasive in eukaryotes [48] and are associated with some cases of resistance to neurotoxic insecticides [78]–[82]. Whereas previous work identified mis-splicing of cadherin mRNA linked with resistance to Cry1Ac in pink bollworm [38], [62] and *H. armigera* [63], our results suggest that alternative splicing at this genetic locus is important in field-evolved resistance of pink bollworm to Cry1Ac produced by Bt cotton in India. The general significance of this genetic mechanism in pest resistance to Bt crops remains to be determined.

## Materials and Methods

### 4.1 Pink Bollworm Field Collections

We studied pink bollworm collected at seven sites from five states in India (Fig. 1 and Table S1). No permission or permit was required for these collections. Pink bollworm is a crop pest that is not an endangered or protected species.

### 4.2 Diet Bioassays

We conducted diet bioassays at the National Centre for Integrated Pest Management laboratory in New Delhi to determine susceptibility to Cry1Ac of first-generation (F_1_) progeny of field-collected pink bollworm from Anand in Gujarat (AGJ) and Akola in Maharashtra (AMH). We obtained 37 live AGJ larvae from 650 bolls of Bt cotton that produces Cry1Ac (Bollgard) collected on 17 January 2011. We obtained ca. 100 live AMH larvae from ca. 1000 non-Bt cotton bolls collected at the Panjabrao Deshmukh Agricultural University Cotton Research Station in Akola on 30 November 2010. Field-collected larvae from each site were reared to pupation on untreated wheat germ diet [49] and allowed to emerge as adults and mate. We obtained eggs and tested the resulting F_1_ neonates individually in 30-mL plastic cups with ca. 5 g diet containing either 0 (control) or 10 micrograms Cry1Ac per mL diet [49], [83]. The source of Cry1Ac was MVPII (Dow Agrosciences, San Diego, CA); a liquid formulation containing protoxin encapsulated in *Pseudomonas fluorescens* [84]. After 21 d at 25°C and a photoperiod of 12 light:12 dark, we scored live third instars, fourth instars, pupae, and adults as survivors. We used Fisher's exact test (http://graphpad.com/quickcalcs/contingency1/) to determine if survival differed significantly between the AGJ and AMH.

### 4.3 DNA Screening of Populations from India for Cadherin Resistance Alleles from Arizona

For DNA-based detection of three cadherin resistance alleles (*r1, r2,* and *r3*) previously identified from laboratory-selected strains of pink bollworm from Arizona [38], [40], [50]–[51], we collected larvae from cotton bolls and adults from pheromone traps at seven sites in five states of India (Table S1 and Fig. 1). As detailed in Table S1, some of the field-collected larvae were reared to the pupal or adult stage on diet in the laboratory. Larvae, pupae, and adults were frozen in ethanol (>95%) for subsequent analysis.

We extracted gDNA from each individual using the PUREGENE DNA Isolation Kit (Qiagen, Valencia, CA). We screened the gDNA of 425 field-collected insects from India for *r1*, *r2*, and *r3* using the protocol and PCR primers described by Morin et al. (2003, 2004) [38], [50] and Tabashnik et al. (2005) [40]. PCR products were separated on 1% agarose gels and visually inspected for the presence of DNA bands of appropriate size. Individuals were counted as screened only if their cadherin gDNA was of good quality, as indicated by successful amplification of one or both bands from conserved portions of the pink bollworm cadherin gene: the ∼700 bp “intron control” band and the ∼1,600 bp “X” band from the *r3x* reaction [42], [50]. Furthermore, we used gDNA previously extracted from laboratory-selected resistant strains containing known *r* alleles (*r1* from AZP-R and BX-H [38], [40]; *r2* from AZP-R [38], [40]; and *r3* from BX-R [37], [40]) as positive controls for genotyping [50]. For 58 insects, we screened the gDNA separately for each individual. For 367 insects, we tested gDNA in 39 pools with 3 to 10 insects per pool (mean  = 9.4 per pool). The tests for each of the three known cadherin *r* alleles included a positive control for each individual or pool, as well as additional positive and negative controls.

### 4.4 Cloning and Sequencing of Pink Bollworm Cadherin cDNA and Gene

We cloned and sequenced cadherin DNA of 11 fourth instar larvae of pink bollworm that were preserved in RNAlater (Ambion-Life Technologies, Carlsbad, CA) from three sites in India: three from AGJ, three from AMH, and five from Khandwa in Madhya Pradesh (KMP) (Fig. 1). The three AGJ larvae used for cloning and sequencing were a subset of the resistant F_1_ larvae that survived exposure to a diagnostic toxin concentration (10 micrograms Cry1Ac per mL diet) in the bioassay described above. The AMH larvae used for cloning and sequencing were collected on 30-November-2010 from non-Bt cotton bolls at the Panjabrao Deshmukh Agricultural University Cotton Research Station in Akola and immediately preserved in RNAlater. KMP larvae were collected during the first week of December 2010 from bolls of Bollgard cotton (Rasi variety) grown by farmers.

#### 4.4.1 cDNA Cloning

Each of the 11 larvae (three from AGJ, three from AMH, and five from KMP) were removed from RNAlater and cut in half. The posterior halves were used to extract RNA, while the anterior halves were returned to RNAlater for later genomic DNA extractions. Total RNA was extracted using TRIzol (Invitrogen-Life Technologies, Carlsbad, CA) according to the manufacturer's instructions. RNA concentration was determined using a NanoDrop ND1000 spectrophotometer (Thermo Scientific, Wilmington, DE) and total RNA quality was assessed with an Agilent BioAnalyzer 2100 with RNA Nano 6000 LabChip Kit (Agilent Technologies, Santa Clara, CA). cDNA was produced using random hexamer primers and ThermoScript RT-PCR System (Invitrogen-Life Technologies) according to the manufacturer's recommendations. From each individual, we used primers 52PgCad5 and 25PgCad3 with high-fidelity SuperTaq Plus DNA Polymerase (Ambion-Life Technologies) to amplify full-length *PgCad1* cDNA (Table S3). PCR products were A-tailed with 1 unit of Takara ExTaq (Takara Bio USA, Madison, WI) and precipitated in ammonium acetate and ethanol. PCR products were resuspended and separated on 0.8% agarose gels stained with Crystal Violet (Invitrogen-Life Technologies). DNA bands were gel-purified and ligated into pCR-XL-TOPO using TOPO XL Gel Purification and PCR cloning kits (Invitrogen-Life Technologies). Plasmids were propagated in OneShot TOP10 electrocompetent *Escherichia coli* (Invitrogen-Life Technologies) and purified using QIAprep Spin MiniPrep kit in QIAcube robot (Qiagen, Valencia, CA). Inserts were sequenced using M13 reverse vector primer, 52PgCad5, 89PgCad5, 57PgCad3, 70PgCad5, 72PgCad5, 73PgCad3, 75PgCad3, 76PgCad5, 77PgCad3, 78PgCad5, 79PgCad3, 20PgCad5, 81PgCad3, 85PgCad3, 87PgCad3, 25PgCad3, and T7 vector primer as appropriate. The nucleotide sequences reported in this paper are deposited in the GenBank public database.

#### 4.4.2 gDNA Cloning

We used a PUREGENE DNA Isolation Kit (Qiagen, Valencia, CA) to extract gDNA from the anterior half of 9 of the 11 larvae described above: AMH-1, AMH-3, AGJ-1, AGJ-3, KMP-4, KMP-5, KMP-6, KMP-7, and KMP-8. gDNA was not extracted from individuals AMH-2 and AGJ-2 because we used all of their tissue for cDNA preparation. *PgCad1*-specific primers (Table S3), designed using Primer3Plus [85], were used with SuperTaq Plus DNA Polymerase to PCR-amplify partial genomic fragments corresponding to mutations found in cDNA from each of the eight individuals. PCR products were gel-purified, ligated into pCR-XL-TOPO or pCR2.1-TOPO (Invitrogen-Life Technologies), and plasmids were propagated in *E. coli* as indicated above. Additional gene-specific primers were used to completely sequence genomic clones (Table S3). The Arizona State University DNA Core Lab (Tempe, AZ) performed the DNA sequencing.

### 4.5 DNA Sequence Analysis

DNA sequences were trimmed, edited, and assembled in Vector NTI (LifeTechnologies). Multiple sequence alignments for DNA and predicted translated proteins were performed using CLUSTAL Omega (1.2.0) [86]. Repbase (http://www.girinst.org/) was searched using CENSOR [53]. Protein translations were obtained using ExPASy Translate tool (http://web.expasy.org/translate/).

## Supporting Information

Figure S1 **Alignment of cadherin cDNA sequences of pink bollworm from Akola, Maharashtra (AMH) with the susceptible allele** ***PgCad1 s*** **(AY198374.1).** Eight of the nine cDNA clones from three individuals (AMH-1, AMH-2, AMH-3) have no insertions or deletions. One cDNA clone (AMH-3_16) has a single 3-bp deletion at base positions 72–74. Stars show nucleotides conserved in all of the sequences. The deletion is highlighted in gray.
(DOCX)

Figure S2 **Alignment of predicted amino acid sequences of pink bollworm cadherin from Akola, Maharashtra (AMH) with** ***PgCad1 s*** **(AY198374.1).** Stars show amino acids conserved in all of the sequences. The symbols “:” and “.” indicate conservative amino acid substitutions scoring >0.5 and ≤0.5 in the Gonnet PAM 250 matrix, respectively. Red boxes show amino acids corresponding to lepidopteran cadherin Cry1Ac toxin binding regions.
(DOCX)

Figure S3 **Alignment of cadherin cDNA sequences of pink bollworm from Anand, Gujarat (AGJ) with the susceptible allele** ***PgCad1 s*** **(AY198374.1).** Thirteen clones from three individuals (AGJ-1, AGJ-2, AGJ-3) had six isoforms of three alleles [*r5A* (KJ480757), *r5B* (KJ480758), *r5C* (KJ480759), *r6A* (KJ480760), *r7A* (KJ480761), and *r7B* (KJ480762)]. Stars show nucleotides conserved in all of the sequences. Deletions are highlighted in gray and the insertion is highlighted in yellow. Codons highlighted in red indicate the positions of premature stop codons.
(DOCX)

Figure S4 **Alignment of predicted amino acid sequences of pink bollworm cadherin from Anand, Gujarat (AGJ) with** ***PgCad1 s*** **(AY198374.1.1).** Stars show amino acids conserved in all of the sequences. The symbols “:” and “.” indicate conservative amino acid substitutions scoring >0.5 and ≤0.5 in the Gonnet PAM 250 matrix, respectively. Red boxes show amino acids corresponding to lepidopteran cadherin Cry1Ac toxin binding regions.
(DOCX)

Figure S5 **Alignment of cadherin cDNA sequences of pink bollworm from Khandwa, Madhya Pradesh (KMP) with the susceptible allele** ***PgCad1 s*** **(AY198374.1).** Twenty-three clones from five individuals (KMP-4, KMP-5, KMP-6, KMP-7, KMP-8) had thirteen isoforms of five *r* alleles [*r8A* (KJ480763), *r8B* (KJ480764), *r9A* (KJ480765), *r9B* (KJ480766), *r10A* (KJ480767), *r10B* (KJ480768), *r10C* (KJ480769), *r11A* (KJ480770), *r11B* (KJ480771), *r12A* (KJ480772), *r12B* (KJ480773), *r12C* (KJ480774), and *r12D* (KJ480775)] and two *s* alleles [clones KMP-8_5, KMP-8_24, and KMP-8_46 for *s6A* (KJ480754), clone KMP-8_35 for *s6B* (KJ480755), and clone KMP-8_3 for *s7* (KJ480756)]. Stars show nucleotides conserved in all of the sequences. Deletions are highlighted in gray and insertions are highlighted in yellow. Codons highlighted in red indicate the positions of premature stop codons.
(DOCX)

Figure S6 **Alignment of predicted amino acid sequences of pink bollworm cadherin from Khandwa, Madhya Pradesh (KMP) with** ***PgCad1 s*** **(AY198374.1).** Stars show amino acids conserved in all of the sequences. The symbols “:” and “.” indicate conservative amino acid substitutions scoring >0.5 and ≤0.5 in the Gonnet PAM 250 matrix, respectively. Red boxes show amino acids corresponding to lepidopteran cadherin Cry1Ac toxin binding regions.
(DOCX)

Figure S7 **Alignment of cadherin cDNA sequences corresponding to susceptible alleles from Akola, Maharashtra (AMH) and Khandwa, Madhya Pradesh (KMP) with the susceptible allele** ***PgCad1 s*** **(AY198374.1).** Fourteen clones from four individuals (AMH-1, AMH-2, AMH-3, KMP-8) have 27 allelic sites [single nucleotide polymorphisms that occur more than once and are not from C-to-U or A-to-I (G) RNA editing]. A total of seven *s* alleles are present from four individuals, including AMH-1 with two alleles, *s1* (clone AMH-1_2, KJ480749) and *s2* (clones AMH-1_7 and 11, KJ480750), AMH-2 with *s3* (clones AMH-2_1, 4, and 5, KJ480751), AMH-3 with *s4* (clones AMH-3_1 and 13, KJ480752) and *s5* (clone AMH-3_16, KJ480753), and two alleles from KMP-8 (*s6A* from clones KMP-8_5, 24, and 46, KJ480754; *s6B* from clone KMP-8_35, KJ480755; and *s7* from KMP-8_3, KJ480756). Allelic bases are shown in red boxes. Stars show nucleotides conserved in all of the sequences. Deletions from mis-spliced mRNA are highlighted in gray.
(DOCX)

Figure S8 **Partial genomic DNA sequencing of seven novel disrupted cadherin alleles in pink bollworm larvae from Anand (AGJ) in Gujarat and Khandwa (KMP) in Madhya Pradesh.** Four mutations (found in isoforms *r5A*, *r8B*, *r11B*, and *r12A*) have altered gDNA, whereas 16 mutations are due to post-transcription modifications. Green-highlighted sequences show location of sense and antisense primers (from Table S3). Exon coding regions are shown as normal text and introns are highlighted in gray. Exon/intron splice junction nucleotides are highlighted in light blue. Yellow-highlighted sequence indicates insertions. Pink-highlighted sequence indicates gaps in sequencing. The 20 gDNA fragments shown are r5A_20-81, r5B_227-228, r5C_89-10, r7A_20-165, r7B_164-163, r8A_186-166, r8B_219-220, r9A_171-25, r9B_58-87, r10A-r10C_20-21, r10A-r10C_169-170, r10B_86-167, r10C_24-85, r11A_20-49, r11B_171-172, r12A_221-222, r12B_168-187, r12C-r12D_227-228, r12C_89-10, and r12D_186-73 (GenBank accession KJ724990-KJ725008). Note that r12A_221-222 does not have an accession number because it does not meet the minimum number of bases required by GenBank.
(DOCX)

Table S1 **Pink bollworm from India screened for cadherin alleles** ***r1-r3*** **from Arizona.**
(DOCX)

Table S2 **gDNA sequencing of eight novel disrupted cadherin alleles in pink bollworm larvae from Anand (AGJ) in Gujarat and Khandwa (KMP) in Madhya Pradesh.**
(DOCX)

Table S3 **Nucleotide primers used to amplify and sequence** ***PgCad1*** **from India pink bollworm.**
(DOCX)
