# Supplementary material for: Alternative Splicing and Highly Variable Cadherin Transcripts Associated with Field-Evolved Resistance of Pink Bollworm to Bt Cotton in India
Source: PLoS One. 2014 May 19;9(5):e97900. doi: 10.1371/journal.pone.0097900 (PMC4026531; doi:10.1371/journal.pone.0097900)
Supplement: Figure S2 — Alignment of predicted amino acid sequences of pink bollworm cadherin from Akola, Maharashtra (AMH) with PgCad1 s (AY198374.1). Stars show amino acids conserved in all of the sequences. The symbols “:” and “.” indicate conservative amino acid substitutions scoring >0.5 and ≤0.5 in the Gonnet PAM 250 matrix, respectively. Red boxes show amino acids corresponding to lepidopteran cadherin Cry1Ac toxin binding regions. (DOCX) [file pone.0097900.s002.docx]

Figure S2. Alignment of predicted amino acid sequences of pink bollworm cadherin from Akola, Maharashtra (AMH) with *PgCad1* *s* (AY198374.1). Stars show amino acids conserved in all of the sequences. The symbols “:” and “.” indicate conservative amino acid substitutions scoring >0.5 and ≤0.5 in the Gonnet PAM 250 matrix, respectively. Red boxes show amino acids corresponding to lepidopteran cadherin Cry1Ac toxin binding regions.

AMH-3_16 MAGDACILVTVLLTFATSVFGQET-SSRCYYMTDAIPREPKPDDLPDLEWTGGWTNWPLI 59

AMH-2_5 MAGDACILVTVLLIFAISVFGQETASSRCYYMTDAIPREPKPDDLPDLEWTGGWTDWPLI 60

AMH-3_13 MAGDACILVTVLLTFATSVFGQETASSRCYYMTDAIPREPKPDDLPDLEWTGGWTDWPLI 60

AMH-2_1 MAGDACILVTVLLIFAISVFGQETASSRCYYMTDAIPREPKPDDLPDLEWTGGWTDWPLI 60

AMH-1_7 MAGDACILVTVLLTFATSVFGQETASSRCYYMTDAIPREPKPDDLPDLEWTGGWTDWPLI 60

AMH-3_1 MAGDACILVTVLLAFATSVFGQETASSRCYYMTDAIPREPKPDDLPDLEWTGGWTDWPLI 60

AMH-2_4 MAGDACILVTVLLIFAISVFGQETASSRCYYMTDAIPREPKPDDLPDLEWTGGWTDWPLI 60

AMH-1_2 MAGDACILVTVLLTFATSVFGQETTSSRCYYMTDAIPREPKPDDLPDLEWTGGWTDWPLI 60

AMH-1_11 MAGDACILVTVLLTFATSVFGQETASSRCYYMTDAIPREPKPDDLPDLEWTGGWTDWPLI 60

AY198374 MAGDACILVTVLLTFATSVFGQETTSSRCYYMTDAIPREPKPDDLPDLEWTGGWTDWPLI 60

************* ** ******* ******************************:****

AMH-3_16 PAEPRDDVCINGWYPQLTSTSLGTIIIHMEEEIEGDVAIAKLNYDGSGTPEIVQPMVIGS 119

AMH-2_5 PAEPRDDVCINDWYPQLTSTSLGTIIIHMEEEIEGDVAIAKLNYDGSGTPEIVQPMVIGS 120

AMH-3_13 PAEPRDDVCINGWYPQLTSTSLGTIIIHMEEEIEGDVAIAKLNYDGSGTPEIVQPMVIGS 120

AMH-2_1 PAEPRDDVCINGWYPQLTSTSLGTIIIHMEEEIEGDVAIAKLYYDGSGTPEIVQPMVIGS 120

AMH-1_7 PAEPRDDVCINGWYPQLTSTSLGTIIIHMEEEIEGDVAIAKLNYDGSGTPEIVQPMVIGS 120

AMH-3_1 PAEPRDDVCINGWYPQLTSTSLGTIIIHMEEEIEGDVAIAKLNYDGSGTPEIVQPMVIGS 120

AMH-2_4 PAEPRDDVCINGWYPQLTSTSLGTIIIHMEEEIEGDVAIAKLNYDGSGTPEIVQPMVIGS 120

AMH-1_2 PAEPRDDVCINGWYPQLTSTSLGTIIIHMEEEIEGDVAIAKLNYDGSGTPEIVQPMVIGS 120

AMH-1_11 PAEPRDDVCINGWYPQLTSTSLGTIIIHMEEEIEGDVAIAKLNYDGSGTPEIVQPMVIGS 120

AY198374 PAEPRDDVCINGWYPQLTSTSLGTIIIHMEEEIEGDVAIAKLNYDGSGTPEIVQPMVIGS 120

*********** ****************************** *****************

AMH-3_16 FNLLSPEIRNENGAWYLYITNRQDYETPTMRRYTFDVRVPDETRAARVSLSIENIDDNDP 179

AMH-2_5 FNLLSPGIRNENGAWYLYITNRQDYETPTMRRYTFDVRVPDETRAARVSLSIENIDDNDP 180

AMH-3_13 FNLLSPEIRNENGAWYLYITNRQDYETPTMRRYTFDVRVPDETRAARVSLSIENIDDNDP 180

AMH-2_1 FNLLSPEIRNENGAWYLYITNRQDYETPTMRRYTFDVRVPDETRAARVSLSIENIDDNDP 180

AMH-1_7 FNLLSPEIRNENGAWYLYITNRQDYETPTMRRYTFDVRVPDETRAARVSLSIENIDDNDP 180

AMH-3_1 FNLLSPEIRNENGAWYLYITNRQDYETPTMRRYTFDVRVPDETRAARVSLSIENIDDNDP 180

AMH-2_4 FNLLSPEILNENGAWYLYITNRQDYETPTMRRYTFDVRVPDETRAARVSLSIENIDDNDP 180

AMH-1_2 FNLLSPGIRNENGAWYLYITNRQDYETPTMRRYTFDVRVPDETRAARVSPSIENIDDNDP 180

AMH-1_11 FNLLSPEIRNENGAWYLYITNRQDYETPTMRRYTFDVRVPDETRAARVSLSIENIDDNDP 180

AY198374 FNLLSPEIRNENGAWYLYITNRQDYETPTMRRYTFDVRVPDETRAARVSLSIENIDDNDP 180

****** * **************************************** **********

AMH-3_16 IVRVLDACQVPELGEPRLTDCVYRVSDEDGRLSIEPMTFRLTSDREDVQIFYVEPAHITG 239

AMH-2_5 IVRVLDACQVPELGEPRLTDCVYQVSDEDGRLSIEPMTFRLTSDREDVQIFYVEPAHITG 240

AMH-3_13 IVRVLDACQVPELGEPRLTDCVYQVSDEDGRLSIEPMTFRLTSDREDVQIFYVEPAHITG 240

AMH-2_1 IVRVLDACQVPELGEPRLTDCVYQVSDEDGRLSIEPMTFRLTSDREDVQIFYVEPAHITG 240

AMH-1_7 IVRVLDACQVPELGEPRLTDCVYQVSDEDGRLSIEPMTFRLTSDREDVQIFYVEPAHITG 240

AMH-3_1 IVRVLDACQVPELGEPRLTDCAYQVSDEDGRLSIEPMTFRLTSDREDVQIFYVEPAHITG 240

AMH-2_4 IVRVLDACQVPELGEPRLTDCVYQVSDEDGRLSIEPMTFRLTSDREDVQIFYVEPAHITG 240

AMH-1_2 IVRVLDACQVPELGEPRLTDCVYQVSDEDGRLSIEPMTFRLTSDREDVQIFYVEPAHITG 240

AMH-1_11 IVRVLDACQVPELGEPRLTDCVYQVSDEDGRLSIEPMTFRLTSDREDVQIFYVEPAHITG 240

AY198374 IVRVLDACQVPELGEPRLTDCVYQVSDEDGRLSIEPMTFRLTSDREDVQIFYVEPAHITG 240

*********************.*:************************************

AMH-3_16 DWFNMQITIGILSALNFESNPLHIFQITALDSWPNNHTVTVMVQVQNVEHRPPRWMEIFA 299

AMH-2_5 DWFNMQITIGILSALNFESNPLHIFQITALDSWPNNHTVTVMVRVQNVEYRPPRWMEIFA 300

AMH-3_13 DWFNMQITIGILSALNFESNPLHIFQITALDSWPNNHTVTVMVQVQNVEHRPPRWMEIFA 300

AMH-2_1 DWFNMQITIGILSALNFESNPPHIFQITALDSWPNYHTVTVMVQVQNVEHRPPRWMEIFA 300

AMH-1_7 DWFNMQITIGILSALNFESNPLHIFQITALDSWPNNHTVTVMVQVQNVEHRPPRWMEIFA 300

AMH-3_1 DWFNMQITIGILSALNFESNPLHIFQITALDSWPNNHTVTVMVQVQNVEHRPPRWMEIFA 300

AMH-2_4 DWFNMQITIGILSALNFESNPLHIFQITALDSWPNNHTVTVMVQVQNVEHRPPRWMEIFA 300

AMH-1_2 DWFNMQITIGILSALNFESNPLHIFQITALDSWPNNHTVTVMVQVQNVEHRPPRWMEIFA 300

AMH-1_11 DWFNMQITIGILSALNFESNPLHIFQITALDSWPNNHTVTVMVQVQNVEHRPPRWMEISA 300

AY198374 DWFNMQITIGILSALNFESNPLHIFQITALDSWPNNHTVTVMVQVQNVEHRPPRWMEIFA 300

********************* ************* *******:*****:******** *

AMH-3_16 VQQFDEMTEQQFQVRAIDGDTGIGKAIHYTLETDEEEDLFFIETLPGGHDGAIFSTAMID 359

AMH-2_5 VQQFDEMTEQQFQVRAIDGDTGIGKAIHYTLETDEEEDLFFIETLPGGHDGAIFGTAMID 360

AMH-3_13 VQQFDEMTEQQFQVRAIDGDTGIGKAIHYTLETDEEEDLFFIETLPGGHDGAIFSTAMID 360

AMH-2_1 VQQFDEMTEQQFQVRAIDGDTGIGKAIHYTLETDEEEDLFFIETLPGGHDGAIFSTAMID 360

AMH-1_7 VQQFDEMTEQQFQVRAIDGDTGIGKAIHYTLETDEEEDLFFIETLPGGHDGAIFSTAMID 360

AMH-3_1 VQQFDEMTEQQFQVRAIDGDTGIGKAIHYTLETDEEEDLFFIETLPGGHDGAIFSTAMID 360

AMH-2_4 AQQFDEMTEQQFQVRAIDGDTGIGKAIHYTLETDEEEDLFFIETLPGGHDGAIFSTAMID 360

AMH-1_2 VQQFDEMTEQQFQVRAIDGDTGIGKAIHYTLETDEEEDLFFIKTLPGGHDGAIFSTAMID 360

AMH-1_11 VQQFDEMTEQQFQVRAIDGDTGIGKAIHYTLETDEEEDLFFIETLPGGHDGAIFSTAMID 360

AY198374 VQQFDEMTEQQFQVRAIDGDTGIGKAIHYTLETDEEEDLFFIETLPGGHDGAIFSTAMID 360

.*****************************************:***********.*****

AMH-3_16 VDRLRRDVFRLSLVAYKYDNVSFATPTPVVIIVNDINNKQPQPLQDEYTISIMEETPLSL 419

AMH-2_5 VDRFRRDVFRLSLVAYKYDNVSFATPTPVVIIVNDINNKQPQPLQDEYTISIMEETPLSL 420

AMH-3_13 VDRLRRDVFRLSLVAYKYDNVSFATPTPVVIIVNDINNKQPQPPQDEYTISIMEETPLSL 420

AMH-2_1 VDRLRRDVFRLSLVAYKYDNVSFATPTPVVIIVNDINNKQPQPLQDEYTISIMEETPLSL 420

AMH-1_7 VDRLRRDVFRLSLVAYKYDNVSFATPTPVVIIVNDINNKQPQPLQDEYTISIMEETPLSL 420

AMH-3_1 VDRLRRDVFRLSLVAYKYDNVSFATPTPVVIIVNDINNKQPQPLQDEYTISIMEETPLSL 420

AMH-2_4 VDRLRRDVFRLSLVAYKYDNVSFATPTPVVIIVNDINNKQPQPLQDEYTISIMEETPLSL 420

AMH-1_2 VDRLRRDVFRLSLVAYKYDNVSFATPTPVVIIVNDINNKKPQPLQDEYTISIMEETPLSL 420

AMH-1_11 VDRLRRDVFRLSLVAYKYDNVSFATPTPVVIIVNDINNKQPQPLQDEYTISIMEETPLSL 420

AY198374 VDRLRRDVFRLSLVAYKYDNVSFATPTPVVIIVNDINNKKPQPLQDEYTISIMEETPLSL 420

***:***********************************:*** ****************

AMH-3_16 NFAELFGFYDEDLIYAQFLVEIQGENPPGVEQAFYIAPTAGFQNQTFAIGTQDHRMLDYE 479

AMH-2_5 NFAELFGFYVEDLIYAQFLVEIQGENPPGVEQAFYIAPTAGFQNQTFAIGTQDHRMLDYE 480

AMH-3_13 NFAELFGFYDEDLIYAQFLVGIQGENPPGVEQAFYIAPTAGFQNQTFAIGTQDHRMLDYE 480

AMH-2_1 NFAELFGFYDEDLIYAQFLVEIQGENPPGVEQAFYIAPTAGFQNQTFAIGTQDHRMLDYE 480

AMH-1_7 NFAELFGFYDEDLIYAQFLVEIQGENPPGVEQAFYIAPTAGFQNQTFAIGTQDHRMLDYE 480

AMH-3_1 NFAELFGFYDEDLIYAQFLVEIQGENPPGVEQAFYIAPTAGFQNQTFAIGTQDHRMLDYE 480

AMH-2_4 NFAELFGFYDEDLIYAQFLVEIQGENPPGVEQAFYIAPTAGFQNQTFAIGTQDHRMLDYE 480

AMH-1_2 NFAELFGFYDEDLIYAQFLVEIQGENPPGVEQAFYIAPTAGFQNQTFAIGTQDHRMLDYE 480

AMH-1_11 NFAELFGFYDEDLIYAQFLVEIQGENPPGVEQAFYIAPTAGFQNQTFAIGTQDHRMLDYE 480

AY198374 NFAELFGFYDEDLIYAQFLVEIQGENPPGVEQAFYIAPTAGFQNQTFAIGTQDHRMLDYE 480

********* ********** ***************************************

AMH-3_16 DVPFQNIKLKVIATDRDNTNFTGVAEVNVNLINWNDEEPIFEEDQLVVKFKETVPKDYHV 539

AMH-2_5 DVPFQNIKLKVIATDRDNTNFTGVAEVNVNLINWNDEEPIFEEDQLVVKLKETVPKDYHV 540

AMH-3_13 DVPFQNIKLKVIATDRDNTNFTGVAEVNVNLINWNDEEPIFEEDQLVVKFKETVPKDYHV 540

AMH-2_1 DVPFQNIKLKVIATDRDNTNFTGVAEVNVNLINWNDEEPIFEEDQLVVKFKETVPKDYHV 540

AMH-1_7 DVPFQNIKLKVIATDRDNTNLTGVAEVNVNLINWDDEEPIFEEDQLVVKFKETVPKDYHV 540

AMH-3_1 DVPFQNIKLKVIATDRDNTNFTGVAEVNVNLINWNDEEPIFEEDQLVVKFKETVPKDYHV 540

AMH-2_4 DVPFQNIKLKVIATDRDNTNFTGVAEVNVNLINWNDEEPIFEEDQLVVKFKETVPKDYHV 540

AMH-1_2 DVPFQNIKLKVIATDRDNTNFTGVAEVNVNLINWNDEEPIFEEDQLVVKFKETVPKDYHV 540

AMH-1_11 DVPFQNIKLKVIATDRDNTNFTGVAEVNVNLINWNDEEPIFEEDQLVVKFKETVPKDYHV 540

AY198374 DVPFQNIKLKVIATDRDNTNFTGVAEVNVNLINWNDEEPIFEEDQLVVKFKETVPKDYHV 540

********************:*************:**************:**********

AMH-3_16 GRLRAHDRDIGDSVVHSILGNANTFLRIDEETGDIYVTIDDAFDYHRQNEFNIQVRAQDT 599

AMH-2_5 GRLRAHDRDIGDSVVHSILGNANTFLRIDEETGDIYVAIDDAFDYHRQNEFNIQVRAQDT 600

AMH-3_13 GRLRAHDRDIGDSVVHSILGNANTFLRIDEETGDIYVTIDDAFDYHRQNEFNIQVRAQDT 600

AMH-2_1 GRLRAHDRDIGDSVVHSILGNANTFLRIDEETGDIYVAIDDAFDYHRQNEFNIQVRAQDT 600

AMH-1_7 GRLRAHDRDIGDSVVHSILGNANTFLRIDEETGDIYVTIDDAFDYHRQNEFNIQVRAQDT 600

AMH-3_1 GRLRAHDRDIGDSVVHSILGNANTFLRIDEETGDIYVTIDDAFDYHRQNEFNIQVRAQDT 600

AMH-2_4 GRLRAHDRDIGDSVVHSILGNANTFLRIDEETGDIYVAIDDAFDYHRQNEFNIQVRAQDT 600

AMH-1_2 GRLRAHDRDIGDSVVHSILGNANTFLRIDEETGDIYVAIDDAFDYHRQNEFNIQVRAQDT 600

AMH-1_11 GRLRAHDRDIGDSVVHSILGNANTFLRIDEETGDIYVTIDDAFDYHRQNEFNIQVRAQDT 600

AY198374 GRLRAHDRDIGDSVVHSILGNANTFLRIDEETGDIYVAIDDAFDYHRQNEFNIQVRAQDT 600

*************************************:**********************

AMH-3_16 MSEPESRHTATAQLVIELEDVNNTPPTLRLPRVSPSVEENVPEGFEINREITATDPDTTA 659

AMH-2_5 MSEPESRHTATAQLVIELEDVNNTPPTLRLPRVSPSVEENVPEGFEINREITATDPDTTA 660

AMH-3_13 MSEPESRHTATAQLVIELEDVNNTPPTLRLPRVSPSVEENVPEGFEINREITATDPDTAA 660

AMH-2_1 MSEPESRHTATAQLVIELEDVNNTPPTLRLPRVSPSVEENVPEGFEINREITATDPDTTA 660

AMH-1_7 MSEPESRHTATAQLVIELEDVNNTPPTLRLPRVSPSVEENVPEGFEINREITATDPDTTA 660

AMH-3_1 MSEPESRHTATAQLVIELEDVNNTPPTLRLPRVSPSVEENVPEGFEINREITATDPDTTA 660

AMH-2_4 MSEPESRHTATAQLVIELEDVNNTPPTLRPPRVSPSVEENVPEGFEINREITATDPDTTA 660

AMH-1_2 MSEPESRHTATAQLVIELEDVNNTPPTLRLPRVSPSVEENVPEGFEINREITATDPDTTA 660

AMH-1_11 MSEPESRHTATAQLVIELEDVNNTPPTLRLPRVSPFVEENVPEGFEINREITATDPDTTA 660

AY198374 MSEPESRHTATAQLVIELEDVNNTPPTLRLPRVSPSVEENVPEGFEINREITATDPDTTA 660

***************************** ***** **********************:*

AMH-3_16 YLQFEIDWDTSFATKQGRDTNPIEFHGCVDIETIFPNPADTREAVGRVVAKEIRHNVTID 719

AMH-2_5 YLQFEIDWDTSFATKQGRDTNPIEFHGCVDIETIFPNPADTGEAVGRVVAKEIRHNVTID 720

AMH-3_13 YLQFEIDWDTSFATKQGRDTNPIEFHGCVDIETIFPNPADTGEAVGRVVAKEIRHNVTID 720

AMH-2_1 YLQFEIDWDTSFATKQGRDTNPIEFHECVDIETIFPNPADTREAVGRVVAKEIRHNVTID 720

AMH-1_7 YLQFEIDWDTSFATKQGRDTNPVEFHGCVDIETIFPNPADTREAVGRVVAKEIRHNVTID 720

AMH-3_1 YLQFEIDWDTSFATKQGRDTNPIEFHGCVDIETIFPNPADTREAVGRVVAKEIRHNVTID 720

AMH-2_4 YLQFEIDWDTSFATKQGRDTNPIEFHGCVDIETIFPNPADTREAVGRVVAKEIRHNVTID 720

AMH-1_2 YLQFEIDWDTSFATKQGRDTNPIEFHGCVDIETIFPNPADTREAVGRVVAKEIRHNVTID 720

AMH-1_11 YLQFEIDWDTSFATKQGRDTNPVEFHGCVDIETIFPNPADTREAVGRVVAKEIRHNVTID 720

AY198374 YLQFEIDWDTSFATKQGRDTNPIEFHGCVDIETIFPNPADTREAVGRVVAKEIRHNVTID 720

**********************:*** ************** ******************

AMH-3_16 FEEFEFLYLTVRVRDLHTEDGRDYDESTFTIIIIDMNDNWPIWASGFLNQTFSIRERSST 779

AMH-2_5 FEEFEFRYLTVRVRDLHTDDGRDYDESTFTIIIIDMNDNWPIWASGFLNQTFSIRERSST 780

AMH-3_13 FEEFEFLYLTVRVRDLHTEDGRDYDESTFTIIIIDANDNWPIWASGFLNQTFSIRERSST 780

AMH-2_1 FEEFEFLYLTVRVRDLHTDDGRDYDESTFTIIIIDMNDNWPIWASGFLNQTFSIRERSST 780

AMH-1_7 FEEFEFLYLTVRVRDLHTEDGRDYDESTFTIIIIDVNDNWPIWASGFLNQTFSIRERSST 780

AMH-3_1 FEEFELLYLTVRVRDLHTEDGRDYDESTFTIIIIDMNDNWPIWASGFLNQTFSIRERSST 780

AMH-2_4 FEEFESLYLTVRVRDLHTDDGRDYDESTFTIIIIDMNDNWPIWASGFLNQTFSIRERSST 780

AMH-1_2 FEEFEFLYLTVRVRDLHTDDGRDYDESTFTIIIIDMNDNWPIWASGFLNQTFSIRERSST 780

AMH-1_11 FEEFEFLYLTVRVRDLHTEDGRDYDESTFTIIIIDMNDNWPIWASGFLNQTFSIRERSST 780

AY198374 FEEFEFLYLTVRVRDLHTDDGRDYDESTFTIIIIDMNDNWPIWASGFLNQTFSIRERSST 780

***** ***********:**************** ************************

AMH-3_16 GVVIGSVLATDIDGPLYNQVRYTIIPQEDTPEGLVQIHFDTGQITVDENGAIDADIPPRW 839

AMH-2_5 GVVIGSVLATDIDGPLYNQVRYTIIPQEDTPEGLVQIHFVTGQITVDENGAIDADIPPRW 840

AMH-3_13 GVVIGSVLATDIDGPLYNQVRYTIIPQEDTPEGLVQIHFVTGQITVDENGAIDADIPPRW 840

AMH-2_1 GVVIGSVLATDIDGPLYNQVRYTIIPQEDTPEGLVQIHFVTGQITVDENGAIDADIPPRW 840

AMH-1_7 GVVIGSVLATDIDGPLYNQVRYTIIPQEDTPEGLVQIHFVTGQITVDENGAIDADIPPRW 840

AMH-3_1 GVVIGSVLATDIDGPLYNQVRYTIIPQEDTPEGLVQIHFVTGQITVDENGAIDADIPPRW 840

AMH-2_4 GVVIGSVLATDIDGPLYNQVRYTIIPQEDTPEGLVQIHFVTGQITVDENGAIDADIPPRW 840

AMH-1_2 GVVIGSVLATDIDGPLYNQVRYTIIPQEDTPEGPVQIHFVTGQITVDENGAIDADIPPRW 840

AMH-1_11 GVVIGSVLATDIDGPLYNQVRYTIIPQEDTPEGLVQIHFVTGQITVDENGAIDADIPPRW 840

AY198374 GVVIGSVLATDIDGPLYNQVRYTIIPQEDTPEGLVQIHFVTGQITVDENGAIDADIPPRW 840

********************************* ***** ********************

AMH-3_16 HLNYAVIASDKCSEENEENCPPDPVFWDTLRDNVINIVDINNKVPAADLSRLNETVYIHE 899

AMH-2_5 HLNYTVIASDKCSEENEENCPPDPVFWDTLRDNVINIVDINNKVPAADLSRFNETVYIYE 900

AMH-3_13 HLNYTVIASDKCSEENEENRPPDPVSWDTLGDNVINIVDINNKVPAADLSRFNETVYIYE 900

AMH-2_1 HLNYTVIASDKCSEENEENCPPDPVFWDTLRDNVINIVDINNKVPAADLSRFNETVYIYE 900

AMH-1_7 HLNYTVIASDKCSEENEENCPPDPVFWDTLGDNVINIVDINNKVPAADLSRFNETVYIYE 900

AMH-3_1 HLNYTVIASDKCSEENEENCPPDPVFWDTLGDNVINIVDINNKVPAADLSRFNETVYIYE 900

AMH-2_4 HLNYTVIASDKCSEENEENCPPDPVFWDTLRDNVINIVDINNKVPAADLSRFNETVYIYE 900

AMH-1_2 HLNYTVIASDKCSEENEENCPPDPVFWDTLGDNVINIVDINNKVPAADLSRFNETVYIYE 900

AMH-1_11 HLNYTVIASDKCSEENEENCPPDPVFWDTLGDNVINIVDINNKVPAADLSRFNETVYIYE 900

AY198374 HLNYTVIASDKCSEENEENCPPDPVFWDTLGDNVINIVDINNKVPAADLSRFNETVYIYE 900

****:************** ***** **** ********************:******:*

AMH-3_16 NAPDFTNVVKIYSIDEDRDEIYHTVRYQINYAVNQRLRGFFAIDLDSGQVYVENTNNELL 959

AMH-2_5 NAPDFTNVVKIYSIDEDRDEIYHTVRYQINYAVNQRLRDFFAIDLDSGQVYVENTNNELL 960

AMH-3_13 NAPDFTNVVKIYSIDEDRDEIYHTVRYQINYAVNQRLRDFFAIDLDSGQVYVENANNELL 960

AMH-2_1 NAPDFTNVVKIYSIDEDRDEIYHTVRYQINYAVNQRLRDFFAIDLDSGQVYVENTNNELL 960

AMH-1_7 NAPDFTNVVKIYSIDEGRDEIYHTVRYQINYAVNQRLRDFFAIDLDSGQVYVENTNNELL 960

AMH-3_1 NAPDFTNVVKIYSIDEDRDEIYHTVRYQINCAVNQRLRDFFAIDLDSGQVYVENTNNELL 960

AMH-2_4 NAPDFTNVVKIYSIDEDRDEIYHTVRYQINYAVNQRLRDFFAIDLDSGQVYVENTNNELL 960

AMH-1_2 NAPDFTNVVKIYSIDEDRDEIYHTVRYQINYAVNQRLRDFFAIDLDSGQVYVENTNNELL 960

AMH-1_11 NAPDFTNVVKIYSIDEDRDEIYHTVRYQINYAVNQRLRDFFAIDLDSGQVYVENTNNELL 960

AY198374 NAPDFTNVVKIYSIDEDRDEIYHTVRYQINYAVNQRLRDFFAIDLDSGQVYVENTNNELL 960

**************** ************* ******* ***************:*****

AMH-3_16 DRDRGEDQHRIFINLIDNFYSEGDGNRNVNTTEVLVILLDENDNAPELPTPEELSWSISE 1019

AMH-2_5 DRDRGEDQHRIFINLIDNFYSEGDGNRNVNTTEVLVILLDENDNAPELPTPEELSWSISE 1020

AMH-3_13 DRDRGEDQHRIFINLIDNFYSEGDGNRNVNTTEVLVILLDENDNAPELPTPEELSWSISE 1020

AMH-2_1 DRGRGEDQHRIFINFIDNFYSEGDGNRNVNTTEVLVILLDENDNAPELPTPEELSWSISE 1020

AMH-1_7 DRDGGEDQHRIFINLIDNFYSEGDGNRNVNTTEVLVILLDENDNAPELSTPEELSWSISE 1020

AMH-3_1 DRGRGEDQHRIFINHIDNFYSEGDGNRNVNTTEVLVILLDENDNAPELPTPEELSWSISE 1020

AMH-2_4 DRDRGEDQHRIFINLIDNFYSEGDGNRNVNTTEVLVILLDENDNAPELPTPEELSWSISE 1020

AMH-1_2 DRDRGEDQHRIFINLIDNFYSEGDGNRNVNTTEVLVILLDENDNAPELPTPEELSWSISE 1020

AMH-1_11 DRDRGEDQHRIFINLIDNFYSEGDGNRNVNTTEVLVILLDENDNAPELPTPEELSWSISE 1020

AY198374 DRDRGEDQHRIFINLIDNFYSEGDGNRNVNTTEVLVILLDENDNAPELPTPEELSWSISE 1020

** ********** ********************************* ***********

AMH-3_16 NLQEGITLDGESDVIYAPDIDEEDTPNSHVGYAILAMTVTNRDLDTVPRLLNMLSPNNVT 1079

AMH-2_5 NLQEGITLDGESDVIYAPDIDEEDTPNSHVGYAILAMTVTNRDLDTVPRLLNMLSPNNVT 1080

AMH-3_13 NLQEGITLDGERDVIYAPDIDEEDTPNSHVGYAILAMTVTNRDLDTVPRLLNMLSPNNVT 1080

AMH-2_1 NLQEGITLDGESDVVYAPDIDEEDTPNSHVGYAILAMTVTNRDLDTVPRLLNMLSPNNVT 1080

AMH-1_7 NLQEGITLDGESDVIYAPDIDEEDTPNSHVGYAILAMTVTNRDLDTVPRLLNMLSPNNVT 1080

AMH-3_1 NLQEGITLDGERDVIYAPDIDEEDTPNSHVGYAILAMTVTNRDLDTVPRLFNMLSPNNVT 1080

AMH-2_4 NLQEGITLDGESDVIYAPDIDEEDTPNSHVGYAILAMTVTNRDLDTVPRLLNMLSPNNVT 1080

AMH-1_2 NLQEGITLDGERDVIYAPDIDEEDTPNSHVGYAILAMTVTNRDLDTVPRLLNMLSPNNVT 1080

AMH-1_11 NLQEGITLDGESDVIYAPDIDEEDTPNSHVGYAILAMTVTNRDLDTVPRLLNMLSPNNVT 1080

AY198374 NLQEGITLDGESDVIYAPDIDEEDTPNSHVGYAILAMTVTNRDLDTVPRLLNMLSPNNVT 1080

*********** **:***********************************:*********

AMH-3_16 GFLQTAMPLRGYWGTYDISVLAFDHGIPQQISHEVYELEIRPYNYNPPQFVFPESGTILR 1139

AMH-2_5 GFLQTAMPLRGYWGTYDISVLAFDHGIPQQISHEVYELEIRPYNYNPPQFVFPESGTILR 1140

AMH-3_13 GFLQTAMPLRGYWGTYDISILAFDHGIPQQISHEVYELEIRPYNYNPPQFVFPESGTILR 1140

AMH-2_1 GFLQTAMPLRGYWGTYDISVLAFDHGIPQQISHEVYELEIRPYNYNPPQFVFPESGTILR 1140

AMH-1_7 GFLQTAMPLRGYWGTYDISILAFDHGIPQQISHEVYELEIRPYNYNPPQFVFPESGTILR 1140

AMH-3_1 GFLQTAMPLRGYWGTYDISILAFDHGIPQQISHEVYELEIRPYNYNPPQFVFPESGTILR 1140

AMH-2_4 GFLQTAMPLRGYWGTYDISVLAFDHGIPQQISHEVYELEIRPFNYNPPQFVFPESGTILR 1140

AMH-1_2 GFLQTAMPLRGYCGTYDISILAFDHGIPQQISHEVYELEIRPYNYNPPQFVFPESGTILR 1140

AMH-1_11 GFLQTAMPLRGYWGTYDISILAFDHGIPQQISHEVYELEIRPYNYNPPQFVFPESGTILR 1140

AY198374 GFLQTAMPLRGYWGTYDISILAFDHGIPQQISHEVYELEIRPYNYNPPQFVFPESGTILR 1140

************ ******:**********************:*****************

AMH-3_16 LALERAVVNNVLSLVNGDPLDRIQAVDDDGLDAGVVTFDIVGDADASNYFRVNNDGDNFG 1199

AMH-2_5 LALERAVVNNVLSLVNGDLLDRIQAIDDDGLDAGVVTFDIVGDADASNYFRVNNDGDNFG 1200

AMH-3_13 LALERAVVNNVLSLVNGDPLDRIQAIDDDGLDAGVVTFDIVGDADASNYFRVNNDGDNFG 1200

AMH-2_1 LALERAVVNNVLSLVNGDLLDRIQAIDDDGLDAGVVTFDIVGDADASNYFRVNNDGDNFG 1200

AMH-1_7 LALERAVVNNVLSLVNGDPLDRIQAIDDDGLDAGVVTFDIVGDADASNYFRVNNDGDNFG 1200

AMH-3_1 LALERAVVNNVLSLVNGDPLDRIQAIDDDGLDAGVVTFDIVGDADASNYFRVNNDGDNFG 1200

AMH-2_4 LALERAVVNNVLSLVNGDLLDRIQAIDDDGLDAGVVTFDIVGDADASNYFRVNNDGDNFG 1200

AMH-1_2 LALERAVVNNVLSLVNGDPLDRIQAIDDDGLDAGVATFDIVGDADASNYFRVNNDGDNFG 1200

AMH-1_11 LALERAVVNNVLSLVNGDPLDRMQAIDDDGLDAGVVTFDIVGDADASNYFRVNNDGDNFG 1200

AY198374 LALERAVVNNVLSLVNGDPLDRIQAIDDDGLDAGVVTFDIVGDADASNYFRVNNDGDNFG 1200

****************** ***:**:*********.************************

AMH-3_16 TLLLTQALPEEGKEFEVTIRATDGGTEPRSYSTDSTITVLFVPTLGDPIFQDNTYSVAFF 1259

AMH-2_5 TLLLTQALPEEGKEFEVTIRATDGGTEPRSYSTDSTITVLFVPTLGDPIFQDNTYSVAFF 1260

AMH-3_13 TLLLTQALPEEGKEFEVTIRATGGGTEPRSYSTDSTITVLFVPTLGDPIFQDNTYSVAFF 1260

AMH-2_1 TLLLTQALPEEGKEFEVTIRATDGGTEPRSYSTDSTITVLFVPTLGDPIFQDNTYSVAFF 1260

AMH-1_7 TLLLTQALPEEGKEFEVTIRATDGGTEPRSYSTDSTITVLFVPTLGDPIFQDNTYSVAFF 1260

AMH-3_1 TLLLTQALPEEGKEFEVTIRATGGGTEPRSYSTDSTITVLFVPTLGDPIFQDNTYSVAFF 1260

AMH-2_4 TLLLTQALPEEGKEFEVTIRATDGGTEPRSYSTDSTITVLFVPTLGDPIFQDNTYSVAFF 1260

AMH-1_2 TLLLTQALPEEGKEFEVTIRATDGGTEPRSYSTDSTITVLFVPTLGDPIFQDNTYSVAFF 1260

AMH-1_11 TLLLTQALPEEGKEFEVTIRATDGGTEPRSYSTDSTITVLFVPTLGDPIFQDNTYSVAFF 1260

AY198374 TLLLTQALPEEGKEFEVTIRATDGGTEPRSYSTDSTITVLFVPTLGDPIFQDNTYSVAFF 1260

********************** *************************************

AMH-3_16 EKEVGLTERFTLPLAEDPKNKLCTDDCHDIYYRIFGGVDYEPFDLDPVTNVIFLKSELDR 1319

AMH-2_5 EKEVGLTERFSLPHAEDPKNKLCTDDCHDIYYRIFGGVDYEPFDLDPVTNVIFLKSELDR 1320

AMH-3_13 EKEVGLTERFSLPLAEDPKNKLCTDDCHDIYYRIFGGADYEPFDLDPVTNVIFLKSELDR 1320

AMH-2_1 EKEVGLTERFSLPHAEDPKNKLCTDDCHDIYYRIFGGVDYEPFDLDPVTNVIFLKSELDR 1320

AMH-1_7 EKEVGLTERFSLPHAEDPKNKLCTDDCHDIYYRIFGSVDYEPFDLDPVTNVIFLKSELDR 1320

AMH-3_1 EKEVGLTERFSLPLAEDPKNKLCTDDCHDIYYRIFGGVDYEPFDLDPVTNVIFLKSELDR 1320

AMH-2_4 EKEVGLTERFSLPHAEDPKNKLCTDDCHDIYYRIFGGVDYEPFDLDPVTNVIFLKSELDR 1320

AMH-1_2 EKEVGLTERFSLPHAEDPKNKLCTDDCHDIYYRIFGGVDYEPFDLDPVTNVIFLKSELDR 1320

AMH-1_11 EKEVGLTERFSLPHAEDPKNKLCTDDCHDIYYRIFGGVDYEPFDLDPVTNVIFLKSELDR 1320

AY198374 EKEVGLTERFSLPHAEDPKNKLCTDDCHDIYYRIFGGVDYEPFDLDPVTNVIFLKSELDR 1320

**********:** **********************..**********************

AMH-3_16 DTTATHVVQVAASNSPTGGGIPLPGSLLTVTVTVREADPRPVFEQRLYTAGISTSDNINR 1379

AMH-2_5 ETTATHVVQVAASNSPTGGGIPLPGSLLTVTVTVREADPRPVFEQRLYTAGISTSDNINR 1380

AMH-3_13 ETTATHVVQVAASNSPTGGGIPLPGSLLTVTVTVREADPRPVFEQRLYTAGISTSDNINR 1380

AMH-2_1 ETTATHVVQVAASNSPTGGGIPLPGSLLTVTVTVREADPRPVFEQRLYTAGISTSDNINR 1380

AMH-1_7 ETTATHVVQVAASNSPTGGGIPLPGSLLTVTVTVREADPRPVFEQRLYTADISTSDNINR 1380

AMH-3_1 ETTATHVVQVAASNSPTGGGIPLPGSLLTVTVTVREADPRPVFEQRLYTAGISTSDNINR 1380

AMH-2_4 ETTATQVVQVAASNSPTGGGIPLPGSLLTVTVTVREADPRPVFEQRLYTAGISTSDNINR 1380

AMH-1_2 ETTATHVVQVAASNSPTGGGIPLPGSLLTVTVTVREADPRPVFEQRLYTAGISTSDNINR 1380

AMH-1_11 ETTATHVVQVAASNSPTGGGIPLPGSLLTVTVTVREADPRPVFEQRLYTAGISTSDNINR 1380

AY198374 ETTATHVVQVAASNSPTGGGIPLPGSLLTVTVTVREADPRPVFEQRLYTAGISTSDNINR 1380

:****:******************************************** *********

AMH-3_16 ELLTVRATHSENAQLTYTIEDGSMVVDSTLEAVKDSAFHLNAQTGVLILRIQPTASMQGM 1439

AMH-2_5 ELLTVRATHSENAQLTYTIEDGSMVVDSTLEAVKDSAFHLNAQTGVLILRIQPTASMQGM 1440

AMH-3_13 ELLTVRATHSENAQLTYTIEDGSMVVDSTLEAVKDSAFHLNAQTGVLILRIQPTASMQGM 1440

AMH-2_1 ELLTVRATHSENAQLTYTIENGSMVVDSTLEAVKDSAFHLNAQTGVLILRIQPTASMQGM 1440

AMH-1_7 GLLTVRATHSENAQLTYTIEDGSMAVDSTLEAVKDSAFHLNAQTGVLILRIQPTASMQGM 1440

AMH-3_1 ELLTVRATHSENAQLTYTIEDGSMVVDSTLEAVKDSAFHLNAQTGVLILRIQPTASMQGM 1440

AMH-2_4 ELLTVRATHSENAQLTYTIEDGSMVVDSTLEAVKDSAFHLNAQTGVLILRIQPTASMQGM 1440

AMH-1_2 ELLTVRATHSENAQLTYTIEDGSMAVDSTLEAVKDSAFHLNAQTGVLILRIQPTASMQGM 1440

AMH-1_11 ELLTVRATHSENAQLTYTIEDGSMAVDSTLEAVKDSALHLNAQTGVLILRIQPTASMQGM 1440

AY198374 ELLTVRATHSENAQLTYTIEDGSMAVDSTLEAVKDSAFHLNAQTGVLILRIQPTASMQGM 1440

*******************:***.************:**********************

AMH-3_16 FEFNVIATDPDEKTDTAEVKVYLISSQNRVSFIFLNDVETVESNRDFIAETFSVGFNMTC 1499

AMH-2_5 FEFNVIATDPDEKTDTAEVKVYLISSQNRVSFIFLNDVETVESNRDFIAETFSVGFNMTC 1500

AMH-3_13 FEFNVIATDPDEKTDTAEVKVYLISSQNRVSFIFLNDVETVESNRDFIAETFSVGFNMTC 1500

AMH-2_1 FEFNVIATDPDEKTDTAEVKVYLISSQNRVSFIFLNDVETVESNGDFIAEAFSVGFNMTC 1500

AMH-1_7 FEFNVIATDPDEKTDAAEVKVYLISSQNRVSFIFLNDVETVESNRDFIAETFSVGFNMTC 1500

AMH-3_1 FEFNVIATDPDEKTDTAEVKVYLISSQNRVSFIFLNDVETVESNRDFIAETFSVGFNMTC 1500

AMH-2_4 FEFNVIATDPDERTDTAEVKVYLISSQNRVSFIFLNDVETVESNRDFIAETISVGFNMTC 1500

AMH-1_2 FEFNVIATDPDEKTDTAEVKVYLISSQNGVSFIFLNDVETVESNRDFIAETFSVGFNMTC 1500

AMH-1_11 FEFNVVATDPDEKTDTAEVKVYLISSQNRVSFIFLNDVETVESNRDFIAETFSVGFNMTC 1500

AY198374 FEFNVIATDPDEKTDTAEVKVYLISSQNRVSFIFLNDVETVESNRDFIAETFSVGFNMTC 1500

*****:******:**:************ *************** *****::********

AMH-3_16 NIDQVLPGTNDAGVIQEAMAEVHAHFIQDNIPVSADSIEELRSDTQLLRSVQGVLNQRLL 1559

AMH-2_5 NIDQVLPGTNDAGVIQEAMAEVHAHFIQDNIPVSADGIEELRSDTQLLRSVQGALNQRLL 1560

AMH-3_13 NIDQVLPGTNDAGVIQEAMAEVHAHFIQDNIPVSADSTEELRSDTQLLRSVQGVLNQRLL 1560

AMH-2_1 NIDQVLPGTNDAGVIQEAMAEVHAHFIQDNIPVSADSIEELRSDTQLLRSVQGVLNQRLL 1560

AMH-1_7 NIDQVLPGTNDAGVIQEAMAEVHAHFMQDNIPVSADSIEELRSDTQLLRSVQGVLNQRLL 1560

AMH-3_1 NIDQVLPGTNDAGVIQEAMAEVHAHFIQDNIPVSADSIEELRGDTQLLRSVQGVLNQRLL 1560

AMH-2_4 NIDQVLPGTNDAGVIQEAMAEVHAHFIQDNIPVSADSIEELRSDTQLLRSVQGVLNQRLL 1560

AMH-1_2 NIDQVLPGTNDAGVIQEAMAEVHAHFIQDNIPVSADSIEELRSDTQLLRSVQGVLNRRLL 1560

AMH-1_11 NIDQVLPGTNDAGVIQEAMAEVHAHFIQDNIPVSADSIEELRSDTQLLRSVQGVLNQRLL 1560

AY198374 NIDQVLPGTNDAGVIQEAMAEVHAHFIQDNIPVSADSIEELRSDTQLLRSVQGVLNQRLL 1560

**************************:*********. ****.**********.**:***

AMH-3_16 VLNDLVTGVSPDLGTAGVQITIYVLAGLSAILAFLCLILLITFIVRTRALNRRLEALSMT 1619

AMH-2_5 VLNDLVTGVSPDLGTAGVQITIYVLAGLSAILAFLCLILLITFIVRTRALNRRLEALSMT 1620

AMH-3_13 VLNDLVTGVSPDLGTAGVQITIYVLAGLSAILAFLCLILLITFIVRTRALNRRLEALSMT 1620

AMH-2_1 VLNDLVTGVSPDLGTAGVQITIYVLAGLSAILAFLCLILLITFIVRTRALNRRLEALSMT 1620

AMH-1_7 VLDDLVTGVSPDLGTAGVQITIYVLAGLSAILAFLCLILLITFIVRTRALNRRLEALSMT 1620

AMH-3_1 VLNDLVTGVSPDLGTAGVQITIYVLAGLSAILAFLCLILLITFIVRTRALNRRLEALSMT 1620

AMH-2_4 VLNDLVTGVSPDLGTAGVQITIYVLAGLSAILAFLCLILLITFIVRTRALNRRLEALSMT 1620

AMH-1_2 VLNDLVTGVSPDLGTAGVQITIYVLAGLSAILAFLCLILLITFIVRTRALNRRLEALSVT 1620

AMH-1_11 VLNDLVTGVSPDLGTAGVQITIYVLAGLSAILAFLCLILLITFIVRTRALNRRLEALSMT 1620

AY198374 VLNDLVTGVSPDLGTAGVQITIYVLAGLSAILAFLCLILLITFIVRTRALNRRLEALSMT 1620

**:*******************************************************:*

AMH-3_16 KYGSVDSGLNRVGIAAPGTNKHAIEGSNPIWNEQIKAPDFDAISDTSDESDLIGIEDLPQ 1679

AMH-2_5 KYGSVDSGLNRVGIAAPGTNKHAIEGSNPIWNEQIKAPDFDAISDTSDESDLIGIEDLPQ 1680

AMH-3_13 KYGSVDSGLSRVGIAAPGTNKHAIEGSNPIWNEQIKAPDFDAISDTSDESDLIGIEDLPQ 1680

AMH-2_1 KYGSVDSGLNRVGIAAPGTNKHAIEGSNPIWNEQIKAPDFDAISDTSDESDLIGIEDLPQ 1680

AMH-1_7 KYGSVDSGLNRVGIAAPGTNKHAIEGSHPIWNEQIKAPDFDAISDTSDESDLIGIEDLPQ 1680

AMH-3_1 KYGSVDSGLNRVGIAAPGTNKHAIEGSNPIWNEQIKAPDFDAISDTSDESDLIGIEDLPQ 1680

AMH-2_4 KYGSVDSGLNRVGIAAPGTNKHAIEGSNPIWNEQIKAPDFDAISDTSDESDLIGIEDLPQ 1680

AMH-1_2 KYGSVDSGLNRVGIAAPGTNKHAIEGSNPIWNEQIKAPDFDAISDTSDESDLIGIEDLPQ 1680

AMH-1_11 KYGSVDSGLNRVGIAAPGTNKHAIEGSNPIWNEQIKAPDFDAISDTSDESDLIGIEDLPQ 1680

AY198374 KYGSVDSGLNRVGIAAPGTNKHAIEGSNPIWNEQIKAPDFDAISDTSDESDLIGIEDLPQ 1680

*********.*****************.********************************

AMH-3_16 FRSDYFPPEDSESAHASFSDRTPRGNDAPIAHSSNNFGFNTSPFSAEFTNRRMRP 1734

AMH-2_5 FKSDYFPPEDSESAHAAFSDRTPRGNDAPIAHSSNNFGFNTSPFSAGFTNRRMRP 1735

AMH-3_13 FKSDYFPPEDSESAHAAFSDRTPRGNDAPIAHSSNNFGFNTSPFSAEFTNRRMRP 1735

AMH-2_1 FKSDYFPPEDSESAHAAFSDRTPRGNDAPIAHSSNNFGFNTSPFSAEFTNRRMRP 1735

AMH-1_7 FKSDYFPPEDSESAHAAFSDRTPRGNDAPIAHSSNNFGFNTSPFSAEFTNRRMRP 1735

AMH-3_1 FKSDYFPPEDSESAHAAFSDRTPRGNDAPIAHSSNNFGFNTSPFSAEFTNRRMRP 1735

AMH-2_4 FKSDYFPPEDSESAHAAFSDRTPRGNDAPIAHSSNNFGFNTSPFSAEFTNRRMRP 1735

AMH-1_2 FKSGYFPPEDSESAHAAFSDRTPRGNDAPIAHSSNNFGFNTSPFSAEFTNRRMRP 1735

AMH-1_11 FKSDYFPPEDSESAHAAFSDRTPRGNDAPIAHSSNNFGFNTSPFSAEFTNRRMRP 1735

AY198374 FKSDYFPPEDSESAHAAFSDRTPRGNDAPIAHSSNNFGFNTSPFSAEFTNRRMRP 1735

*:* ************:***************************** ********
