# Supplementary material for: Alternative Splicing and Highly Variable Cadherin Transcripts Associated with Field-Evolved Resistance of Pink Bollworm to Bt Cotton in India
Source: PLoS One. 2014 May 19;9(5):e97900. doi: 10.1371/journal.pone.0097900 (PMC4026531; doi:10.1371/journal.pone.0097900)
Supplement: Figure S3 — Alignment of cadherin cDNA sequences of pink bollworm from Anand, Gujarat (AGJ) with the susceptible allele PgCad1 s (AY198374.1). Thirteen clones from three individuals (AGJ-1, AGJ-2, AGJ-3) had six isoforms of three alleles [r5A (KJ480757), r5B (KJ480758), r5C (KJ480759), r6A (KJ480760), r7A (KJ480761), and r7B (KJ480762)]. Stars show nucleotides conserved in all of the sequences. Deletions are highlighted in gray and the insertion is highlighted in yellow. Codons highlighted in red indicate the positions of premature stop codons. (DOCX) [file pone.0097900.s003.docx]

Figure S3. Alignment of cadherin cDNA sequences of pink bollworm from Anand, Gujarat (AGJ) with the susceptible allele *PgCad1* *s* (AY198374.1). Thirteen clones from three individuals (AGJ-1, AGJ-2, AGJ-3) had six isoforms of three alleles [*r5A* (KJ480757), *r5B* (KJ480758), *r5C* (KJ480759), *r6A* (KJ480760), *r7A* (KJ480761), and *r7B* (KJ480762)]. Stars show nucleotides conserved in all of the sequences. Deletions are highlighted in gray and the insertion is highlighted in yellow. Codons highlighted in red indicate the positions of premature stop codons.

AGJ-1_5 (*r5B*) ATGGCGGGTGACGCCTGCATACTGGTGACGGTGCTTCTGACCTTCGCAACATCAGTTTTC 60

AGJ-1_6 (*r5B*) ATGGCGGGTGACGCCTGCATACTGGTGACGGTGCTTCTGACCTTCGCAACATCAGTTTTC 60

AGJ-1_1 (*r5A*) ATGGCGGGTGACGCCTGCATACTGGTGACGGTGCTTCTGACCTTCGCAACAGCAGTTTTC 60

AGJ-1_3 (*r5C*) ATGGCGGGTGACGCCTGCATACTGGTGACGGTGCTTCTGACCTTCGCAACATCAGTTTTC 60

AGJ-1_2 (*r5A*) ATGGCGGGTGACGCCTGCATACTGGTGACGGTGCTTCTGACCTTCGCAACATCAGTTTTC 60

AGJ-1_4 (*r5A*) ATGGCGGGTGACGCCTGCATACTGGTGACGGTGCTTCTGACCTTCGCAACATCAGTTTTC 60

AGJ-3_15 (*r7A*) ATGGCGGGTGACGCCTGCATACTGGTGACGGTGCTTCTCACCTTCGCAACATCAGTTTTC 60

AGJ-3_3 (*r7B*) ATGGCGGGTGACGCCTGCATACTGGTGACGGTGCTTCTCACCTTCGCAACATCAGTTTTC 60

AGJ-3_1 (*r7B*) ATGGCGGGTGACGCCTGCATACTGGTGACGGTGCTTCTCACCTTCGCAACATCAGTTTTC 60

AGJ-3_20 (*r7B*) ATGGCGGGTGACGCCTGCATACTGGTGACGGTGCTTCTCACCTTCGCAACATCAGTTTTC 60

AY198374.1 ATGGCGGGTGACGCCTGCATACTGGTGACGGTGCTTCTGACCTTCGCAACATCAGTTTTC 60

AGJ-2_20 (*r6A*) ATGGCGGGTGACGCCTGCATACTGGTGACGGTGCTTCTCACCTTCGCAACATCAGTTTTC 60

AGJ-2_10 (*r6A*) ATGGCGGGTGACGCCTGCATACTGGTGACGGTGCTTCTCACCTTCGCAACATCAGTTTTC 60

AGJ-2_15 (*r6A*) ATGGCGGGTGACGCCTGCATACTGGTGACGGTGCTTCTCACCTTCGCAACATCAGTTTTC 60

************************************** ************ ********

AGJ-1_5 (*r5B*) GGGCAAGAAAC---ATCGTCGAGATGTTACTACATGACTGACGCTATTCCGAGAGAACCG 117

AGJ-1_6 (*r5B*) GGGCAAGAAAC---ATCGTCGAGATGTTACTACATGACTGACGCTATTCCGAGAGAACCG 117

AGJ-1_1 (*r5A*) GGGCAAGAAACAGCATCGTCGAGATGTTACTACATGACTGACGCTATTCCGAGAGAACCG 120

AGJ-1_3 (*r5C*) GGGCAAGAAACAGCATCGTCGAGATGTTACTACATGACTGACGCTATTCCGAGAGAACCG 120

AGJ-1_2 (*r5A*) GGGCAAGAAACAGCATCGTCGAGATGTTACTACATGACTGACGCTATTCCGAGAGAACCG 120

AGJ-1_4 (*r5A*) GGGCAAGAAACAGCATCGTCGAGATGTTACTACATGACTGACGCTATTCCGAGAGAACCG 120

AGJ-3_15 (*r7A*) GGGCAAGAAACAGCATCGTCGAGATGTTACTACATGACTGACGCTATTCCGAGAGAACCG 120

AGJ-3_3 (*r7B*) GGGCAAGAAACAGCATCGTCGAGATGTTACTACATGACTGACGCTATTCCGAGAGAACCG 120

AGJ-3_1 (*r7B*) GGGCAAGAAACAGCATCGTCGAGATGTTACTACATGACTGACGCTATTCCGAGAGAACCG 120

AGJ-3_20 (*r7B*) GGGCAAGAAACAGCATCGTCGAGATGTTACTACATGACTGACGCTATTCCGAGAGAACCG 120

AY198374.1 GGGCAAGAAACAACATCGTCGAGATGTTACTACATGACTGACGCTATTCCGAGAGAACCG 120

AGJ-2_20 (*r6A*) GGGCAAGAAACAACATCGTCGAGATGTTACTACATGACTGACGCTATTCCGAGGGAACCG 120

AGJ-2_10 (*r6A*) GGGCAAGAAACAACATCGTCGAGATGTTACTACATGACTGACGCTATTCCGAGGGAACCG 120

AGJ-2_15 (*r6A*) GGGCAAGAAACAACATCGTCGAGATGTTACTACATGACTGACGCTATTCCGAGGGAACCG 120

*********** ***************************************.******

AGJ-1_5 (*r5B*) AAACCGGATGATTTGCCTGATTTAGAATGGACTGGTGGATGGACCGACTGGCCTTTGATC 177

AGJ-1_6 (*r5B*) AAACCGGATGATTTGCCTGATTTAGAATGGACTGGTGGATGGACCGACTGGCCTTTGATC 177

AGJ-1_1 (*r5A*) AAACCGGATGATTTGCCTGATTTAGAATGGACTGGTGGATGGACCGACTGGCCTTTGATC 180

AGJ-1_3 (*r5C*) AAACCGGATGATTTGCCTGATTTAGAATGGACTGGTGGATGGACCGACTGGCCTTTGATC 180

AGJ-1_2 (*r5A*) AAACCGGATGGTTTGCCTGATTTAGAATGGACTGGTGGATGGACCGACTGGCCTTTGATC 180

AGJ-1_4 (*r5A*) AAACCGCATGATTTGCCTGATTTAGAATGGACTGGTGGATGGACCGACTGGCCTTTGATC 180

AGJ-3_15 (*r7A*) AAACCGGATGATTTGCCTGATTTAGAATGGACTGGTGGATGGACCGACTGGCCTTTGATC 180

AGJ-3_3 (*r7B*) AAACCGGATGATTTGCCTGATTTAGAATGGGCTGGTGGATGGACCGACTGGCCTTTGATC 180

AGJ-3_1 (*r7B*) AAACCGGATGATTTGCCTGATTTAGAATGGACTGGTGGATGGACCGACTGGCCTTTGATC 180

AGJ-3_20 (*r7B*) AAACCGGATGATTTGCCTGATTTAGAATGGACTGGTGGATGGACCGACTGGCCTTTGATC 180

AY198374.1 AAACCGGATGATTTGCCTGATTTAGAATGGACTGGTGGATGGACCGACTGGCCTTTGATC 180

AGJ-2_20 (*r6A*) AAACCGGATGATTTGCCTGACTTAGAATGGACTGGTGGATGGACCGACTGGCCTTTGATC 180

AGJ-2_10 (*r6A*) AAACCGGATGATTTGCCTGACTTAGAATGGACTGGTGGATGGACCGACTGGCCTTTGATC 180

AGJ-2_15 (*r6A*) AAACCGGATGATTTGCCTGACTTAGAATGGACTGGTGGATGGACCGACTGGCCTTTGATC 180

****** ***.********* *********.*****************************

AGJ-1_5 (*r5B*) CCGGCTGAGCCAAGAGACGACGTGTGCATAAACGGCTGGTACCCGCAACTCACCAGCACT 237

AGJ-1_6 (*r5B*) CCGGCTGAGCCAAGAGACGACGTGTGCATAAACGGCTGGTACCCACAACTCACCAGCACT 237

AGJ-1_1 (*r5A*) CCGGCTGAGCCAAGAGACGACGTGTGCATAAACGGCTGGTACCCACAACTCACCAGCACC 240

AGJ-1_3 (*r5C*) CCGGCTGAGCCAAGAGACGACGTGTGCATAAACGGCTGGTACCCACAACTCACCAGCACT 240

AGJ-1_2 (*r5A*) CCGGCTGAGCCAAGAGACAACGTGTGCATAAACGGCTGGTACCCACAACTCACCAGCACT 240

AGJ-1_4 (*r5A*) CCGGCTGAGCCAAGAGACGACGTGTGCATAAACGGCTGGTACCCACAACTCACCAGCACT 240

AGJ-3_15 (*r7A*) CCGGCTGAGCCAAGAGACGACGTGTGCATAAACGGCTGGTACCCACAACTCACCAGCACT 240

AGJ-3_3 (*r7B*) CCGGCTGAGCCAAGAGACGACGTGTGCATAAACGGCTGGTACCCACAACTCACCAGCACT 240

AGJ-3_1 (*r7B*) CCGGCTGAGCCAAGAGACGACGTGTGCATAAACGGCTGGTACCCACAACTCACCAGCACT 240

AGJ-3_20 (*r7B*) CCGGCTGAGCCAAGAGACGACGTGTGCATAAACGGCTGGTACCCACAACTCACCAGCACT 240

AY198374.1 CCGGCTGAGCCAAGAGACGACGTGTGCATAAACGGCTGGTACCCACAACTCACCAGCACT 240

AGJ-2_20 (*r6A*) CCGGCTGAGCCAAGAGACGACGTGTGCATAAACGGCTGGTACCCACAACTCACCAGCACT 240

AGJ-2_10 (*r6A*) CCGGCTGAGCCAAGAGACGACGTGTGCATAAACGGCTGGTACCCACAACTCACCAGCACT 240

AGJ-2_15 (*r6A*) CCGGCTGAGCCAAGAGACGACGTGTGCATAAACGGCTGGTACCCACAACTCACCAGCACT 240

******************.*************************.**************

AGJ-1_5 (*r5B*) TCTCTCGGCACCATCATCATCCACATGGAAGAGGAGATCGAGGGAGATGTTGCTATCGCT 297

AGJ-1_6 (*r5B*) TCTCTCGGCACCATCATCATCCACATGGAAGAGGAGATCGAGGGAGATGTTGCTATCGCT 297

AGJ-1_1 (*r5A*) TCTCTCGGCACCATCATCATCCACATGGAAGAGGAGATCGAGGGAGATGTTGCTATCGCT 300

AGJ-1_3 (*r5C*) TCTCTCGGCACCATCATCATCCACATGGAAGAGGAGATCGAGGGAGATGTTGCTATCGCT 300

AGJ-1_2 (*r5A*) TCTCCCGGCACCATCATCATCCACATGGAAGAGGAGATCGAGGGAGATGTTGCTATCGCT 300

AGJ-1_4 (*r5A*) TCTCTCGGCACCATCATCATCCACATGGAAGAGGAGATCGAGGGAGATGTTGCTATCGCT 300

AGJ-3_15 (*r7A*) TCTCTCGGCACCATCATCATCCACATGGAAGAGGAGATCGAGGGAGATGTCGCTATCGCT 300

AGJ-3_3 (*r7B*) TCTCTCGGCACCATCATCATCCACATGGAAGAGGAGATCGAGGGAGATGTCGCTATCGCT 300

AGJ-3_1 (*r7B*) TCTCTCGGCACCATCATCATCCACATGGAAGAGGAGATCGAGGGAGATGTCGCTATCGCT 300

AGJ-3_20 (*r7B*) TCTCTCGGCACCATCATCATCCACATGGAAGAGGAGATCGAGGGAGATGTCGCTATCGCT 300

AY198374.1 TCTCTCGGCACCATCATCATCCACATGGAAGAGGAGATCGAGGGAGATGTTGCTATCGCT 300

AGJ-2_20 (*r6A*) TCTCTCGGCACCATCATCATCCACATGGAAGAGGAGATCGAGGGAGATGTTGCTATCGCT 300

AGJ-2_10 (*r6A*) TCTCTCGGCACCATCATCATCCACATGGAAGAGGAGATCGAGGGAGATGTTGCTATCGCT 300

AGJ-2_15 (*r6A*) TCTCTCGGCACCATCATCATCCACATGGAAGAGGAGATCGAGGGAGATGTTGCTATCGCT 300

**** ********************************************* *********

AGJ-1_5 (*r5B*) AAACTTAACTATGATGGTTCTGGAACCCCAGAAATTGTCCAGCCGATGGTTATAGGATCT 357

AGJ-1_6 (*r5B*) AAACTTAACTATGATGGTTCTGGAACCCCAGAAATTGTCCAGCCGATGGTTATAGGATCT 357

AGJ-1_1 (*r5A*) AAACTTAACTATGATGGTTCTGGAACCCCAGAAATTGTCCAGCCGATGGTTATAGGATCT 360

AGJ-1_3 (*r5C*) AAACTTAACTATGATGGTTCTGGAACCCCAGAAATTGTCCAGCCGATGGTTATAGGATCT 360

AGJ-1_2 (*r5A*) AAACTTAACTATGATGGTTCTGGAACCCCAGAAATTGTCCAGCCGATGGTTATAGGATCT 360

AGJ-1_4 (*r5A*) AAACTTAACTATGATGGTTCTGGAACCCCAGAAATTGTCCAGCCGATGGTTATAGGATCT 360

AGJ-3_15 (*r7A*) AAACTTAACTATGATGGTTCTGGAACCCCAGAAATTGTCCAGCCGATGGTTATAGGATCT 360

AGJ-3_3 (*r7B*) AAACTTAACTATGATGGTTCTGGAACCCCAGAAATTGTCCAGCCGATGGTTATAGGATCT 360

AGJ-3_1 (*r7B*) AAACTTAACTATGATGGTTCTGGAACCCCAGAAATTGTCCAGCCGATGGTTATAGGATCT 360

AGJ-3_20 (*r7B*) AAACTTAACTATGATGGTTCTGGAACCCCAGAAATTGTCCAGCCGATGGTTATAGGATCT 360

AY198374.1 AAACTTAACTATGATGGTTCTGGAACCCCAGAAATTGTCCAGCCGATGGTTATAGGATCT 360

AGJ-2_20 (*r6A*) AAACTTAACTATGATGGTTCTGGAACCCCAGAAATTGTCCAGCCGATGGTTATAGGATCT 360

AGJ-2_10 (*r6A*) AAACTTAACTATGATGGTTCTGGAACCCCAGAAATTGTCCAGCCGATGGTTATAGGATCT 360

AGJ-2_15 (*r6A*) AAACTTAACTATGATGGTTCTGGAACCCCAGAAATTGTCCAGCCGATGGTTATAGGATCT 360

************************************************************

AGJ-1_5 (*r5B*) TTTAACCTGCTAAGTCCAGAGATCCGGAATGAAAACGGGGCGTGGTACCTTTATATAACC 417

AGJ-1_6 (*r5B*) TTTAACCTGCTAAGTCCAGAGATCCGGAATGAAAACGGGGCGTGGTACCTTTATATAACC 417

AGJ-1_1 (*r5A*) TTTAACCTGCTAAGTCCAGAGATCCGGAATGAAAACGGGGCGTGGTACCTTTATATAACC 420

AGJ-1_3 (*r5C*) TTTAACCTGCTAAGTCCAGAGATCCGGAATGAAAACGGGGCGTGGTACCTTTATATAACC 420

AGJ-1_2 (*r5A*) TTTAACCTGCTAAGTCCAGAGATCCGGAATGAAAACGGGGCGTGGTACCTTTATATAACC 420

AGJ-1_4 (*r5A*) TTTAACCTGCTAAGTCCAGAGATCCGGAATGAAAACGGGGCGTGGTACCTTTATATAACC 420

AGJ-3_15 (*r7A*) TTTAACCTGCTAAGTCCAGAGATCCGGAATGAAAACGGGGCGTGGTACCTTTATATAACC 420

AGJ-3_3 (*r7B*) TTTAACCTGCTAAGTCCAGAGATCCGGAATGAAAACGGGGCGTGGTACCTTTATATAACC 420

AGJ-3_1 (*r7B*) TTTAACCTGCTAAGTCCAGAGATCCGGAATGAAAACGGGGCGTGGTACCTTTATATAACC 420

AGJ-3_20 (*r7B*) TTTAACCTGCTAAGTCCAGAGATCCGGAATGAAAACGGGGCGTGGTACCTTTATATAACC 420

AY198374.1 TTTAACCTGCTAAGTCCAGAGATCCGGAATGAAAACGGGGCGTGGTACCTTTATATAACC 420

AGJ-2_20 (*r6A*) TTTAACCTGCTAAGTCCAGAGATCCGGAATGAAAACGGGGCGTGGTACCTTTATATAACC 420

AGJ-2_10 (*r6A*) TTTAACCTGCTAAGTCCAGAGATCCGAAATGAAAACGGGGCGTGGTACCTTTATATAACC 420

AGJ-2_15 (*r6A*) TTTAACCTGCTAAGTCCAGAGATCCGGAATGAAAACGGGGCGTGGTACCTTTATATAACC 420

**************************.*********************************

AGJ-1_5 (*r5B*) AATAG--------------------GCAAGATTATGAAACACCAACAACGCGTCGGTATA 457

AGJ-1_6 (*r5B*) AATAG--------------------GCAAGATTATGAAACACCAACAATGCGTCGGTATA 457

AGJ-1_1 (*r5A*) AATAG--------------------GCAAGATTATGAAACACCAACAATGCGTCGGTATA 460

AGJ-1_3 (*r5C*) AATAGTTCTGAACTTGAAATCGCAGGCAAGATTATGAAACACCAACAATGCGTCGGTATA 480

AGJ-1_2 (*r5A*) AATAG--------------------GCAAGATTATGAAACACCAACAATGCGTCGGTATA 460

AGJ-1_4 (*r5A*) AATAG--------------------GCAAGATTATGAAACACCAACAATGCGTCGGTATA 460

AGJ-3_15 (*r7A*) AATAG--------------------GCAAGATTATGAAACACCAACAATGCGTCGGTATA 460

AGJ-3_3 (*r7B*) AATAG--------------------GCAAGATTATGAAACACCAACAATGCGTCGGTATA 460

AGJ-3_1 (*r7B*) AATAG--------------------GCAAGATTATGAAGCACCAACAATGCGTCGGTATA 460

AGJ-3_20 (*r7B*) AATAG--------------------GCAAGATTATGAAACACCAACAATGCGTCGGTATA 460

AY198374.1 AATAG--------------------GCAAGATTATGAAACACCAACAATGCGTCGGTATA 460

AGJ-2_20 (*r6A*) AATAG--------------------GCAAGATTATGAGACACCAACAATGCGTCGGTATA 460

AGJ-2_10 (*r6A*) AATAG--------------------GCAAGATTATGAGACACCAACAATGCGTCGGTATA 460

AGJ-2_15 (*r6A*) AATAG--------------------GCAAGATTATGAAACACCAACAATGCGTCGGTATA 460

***** ************..********* ***********

AGJ-1_5 (*r5B*) CATTCGACGTCCGAGTGCCAGACGAGACCCGTGCCGCACGAGTGAGTCTCTCCATCGAAA 517

AGJ-1_6 (*r5B*) CATTCGACGTCCGAGTGCCAGACGAGACTCGTGCCGCACGAGTGAGTCTCTCCATCGAAA 517

AGJ-1_1 (*r5A*) CATTCGACGTCCGAGTGCCAGACGAGACTCGTGCCGCACGAGTGAGTCTCTCCATCGAAA 520

AGJ-1_3 (*r5C*) CATTCGACGTCCGAGTGCCAGACGAGACTCGTGCCGCACGTGTGAGTCTCTCCATCGAAA 540

AGJ-1_2 (*r5A*) CATTCGACGTCCGAGTGCCAGACGAGACTCGTGCCGCACGAGTGAGTCTCTCCATCGAAA 520

AGJ-1_4 (*r5A*) CATTCGACGTCCGAGTGCCAGACGAGACTCGTGCCGCACGAGTGAGTCTCTCCATCGAAA 520

AGJ-3_15 (*r7A*) CATTCGACGTCCGAGTGCCAGACGAGACTCGTGCGGCACGAGTGAGTCTCTCCATCGAAA 520

AGJ-3_3 (*r7B*) CATTCGACGTCCGAGTGCCAGACGAGACTCGTGCGGCACGAGTGAGTCTCTCCATCGAAA 520

AGJ-3_1 (*r7B*) CATTCGACGTCCGAGTGCCAGACGAGACTCGTGCGGCACGAGTGAGTCTCTCCATCGAAA 520

AGJ-3_20 (*r7B*) CATTCGACGTCCGAGTGCCAGACGAGACTCGTGCGGCACGAGTGAGTCTCTCCATCGAAA 520

AY198374.1 CATTCGACGTCCGAGTGCCAGACGAGACTCGTGCGGCACGAGTGAGTCTGTCCATCGAAA 520

AGJ-2_20 (*r6A*) CATTCGACGTCCGAGTGCCAGACGAGACTCGTGCGGCACGAGTGAGTCTGTCCATCGAGA 520

AGJ-2_10 (*r6A*) CATTCGACGTCCGAGTGCCAGACGAGACTCGTGCGGCACGAGTGAGTCTGTCCATCGAAA 520

AGJ-2_15 (*r6A*) CATTCGACGTCCGAGTGCCAGACGAGACTCGTGCGGCACGAGTGAGTCTGTCCATCGAAA 520

**************************** ***** *****:******** ********.*

AGJ-1_5 (*r5B*) ACATTGACGATAACGACCCTATCGTCAGGGTGCTAGACGCTTGCCAAGTGCCGGAATTGG 577

AGJ-1_6 (*r5B*) ACATTGACGATAACGACCCTATCGTCAGGGTGCTAAACGCTTGCCAAGTGCCGGAATTGG 577

AGJ-1_1 (*r5A*) ACATTGACGATAACGACCCTATCGTCAGGGTGCTAGACGCTTGCCAAGTGCCGGAATTGG 580

AGJ-1_3 (*r5C*) ACATTGACGATAACGACCCTATCGTCAGGGTGCTAGACGCTTGCCAAGTGCCGGAATTGG 600

AGJ-1_2 (*r5A*) ACATTGACGATAACGACCCTATCGTCAGGGTGCTAGACGCTTGCCAAGTGCCGGAATTGG 580

AGJ-1_4 (*r5A*) ACATTGACGATAACGACCCTATCGTCGGGGTGCTAGACGCTTGCCAAGTGCCGGAATTGG 580

AGJ-3_15 (*r7A*) ACATTGACGATAACGACCCTATCGTCAGGGTGCTAGTCGCTTGCCAAGTGCCGGAATTGG 580

AGJ-3_3 (*r7B*) ACATTGACGATAACGACCCTATCGTCAGGGTGCTAGACGCTTGCCAAGTGCCGGAATTGG 580

AGJ-3_1 (*r7B*) ACATTGACGATAACGACCCTATCGTCAGGGTGCTAGACGCTTGCCAAGTGCCGGAATTGG 580

AGJ-3_20 (*r7B*) ACATTGACGATAACGACCCTATCGTCAGGGTGCTAGACGCTTGCCAAGTGCCGGAATTGG 580

AY198374.1 ACATTGACGATAACGACCCTATCGTCAGGGTGCTAGACGCTTGCCAAGTGCCGGAATTGG 580

AGJ-2_20 (*r6A*) ACATTGACGATAACGACCCTATCGTCAGGGTGCTAGACGCTTGCCAAGTGCCGGAATTGG 580

AGJ-2_10 (*r6A*) ACATTGACGATAACGACCCTATCGTCAGGGTGCTAGACGCTTGCCAAGTGCCGGAATTGG 580

AGJ-2_15 (*r6A*) ACATTGACGATAACGACCCTATCGTCAGGGTGCTAGACGCTTGCCAAGTGCCGGAATTGG 580

**************************.********.:***********************

AGJ-1_5 (*r5B*) GGGAGCCTCGACTAACTGACTGCGTTTACCAAGTGTCAGACGAAGATGGGAGGCTTAGTA 637

AGJ-1_6 (*r5B*) GGGAGCCTCGACTAACTGACTGCGTTTACCAAGTGTCAGACGAAGATGGGAGGCTTAGTA 637

AGJ-1_1 (*r5A*) GGGAGCCTCGACTAACTGACTGTGTTTACCAAGTGTCAGACGAAGATGGGAGGCTTAGTA 640

AGJ-1_3 (*r5C*) GGGAGCCTCGACTAACTGACTGCGTTTACCAAGTGTCAGACGAAGATGGGAGGCTTAGTA 660

AGJ-1_2 (*r5A*) GGGAGCCTCGACTAACTGACTGCGTTTACCAAGTGTCAGACGAAGATGGGAGGCTTGGTA 640

AGJ-1_4 (*r5A*) GGGAGCCTCGACTAACTGACTGCGTTTACCAAGTGTCAGACGAAGATGGGAGGCTTAGTA 640

AGJ-3_15 (*r7A*) GGGAGCCTCGACTAACTGACTGCGTTTACCAAGTGTCAGACGAAGATGGGAGGCTTAGTA 640

AGJ-3_3 (*r7B*) GGGAGCCTCGACTAACTGACTGCGGTTACCAAGTGTCAGACGAAGATGGGAGGCTTAGTA 640

AGJ-3_1 (*r7B*) GGGAGCCTCGACTAACTGACTGCGTTTACCAAGTGTCAGACGAAGATGGGAGGCTTAGTA 640

AGJ-3_20 (*r7B*) GGGAGCCTCGACTAACTGACTGCGTTTACCAAGTGTCAGACGAAGATGGGAGGCTTAGTA 640

AY198374.1 GGGAGCCTCGACTAACAGACTGCGTTTACCAAGTGTCAGACGAAGATGGGAGGCTTAGTA 640

AGJ-2_20 (*r6A*) GGGAGCCTCGACTAACAGACTGCGTTTACCAAGTGTCAGACGAAGATGGGAGGCTTAGTA 640

AGJ-2_10 (*r6A*) GGGAGCCTCGACTAACAGACTGCGTTTACCAAGTGTCAGACGAAGATGGGAGGCTTAGTA 640

AGJ-2_15 (*r6A*) GGGAGCCTCGACTAACAGACTGCGTTTACCAAGTGTCAGACGAAGATGGGAGGCTTAGTA 640

****************:***** * *******************************.***

AGJ-1_5 (*r5B*) TCGAGCCCATGACATTCCGCCTCACATCAGACCGTGAAGACGTACAGATATTCTATGTGG 697

AGJ-1_6 (*r5B*) TCGAGCCCATGACATTCCGCCTCACATCAGACCGTGAAGACGTACAGATATTCTATGTGG 697

AGJ-1_1 (*r5A*) TCGAGCCCATGACATTCCGCCTCACATCAGACCGTGAAGACGTACAGATATTCTATGTGG 700

AGJ-1_3 (*r5C*) TCGAGCCCATGACATTCCGCCTCACATCAGACCGTGAAGACGTACAGATATTCTATGTGG 720

AGJ-1_2 (*r5A*) TCGAGCCCATGACATTCCGCCTCACATCAGACCGTGAAGACGTACAGATATTCTATGTGG 700

AGJ-1_4 (*r5A*) TCGAGCCCATGACATTCCGCCTCACATCAGACCGTGAAGACGTACAGATATTCTATGTGG 700

AGJ-3_15 (*r7A*) TCGAGCCCATGACATTCCGCCTCACATCAGACCGTGAAGACGTACTGATATTCTACGTGG 700

AGJ-3_3 (*r7B*) TCGAGCCCATGACATTCCGCCTCACATCAGACCGTGAAGACGTACAGATATTCTACGTGG 700

AGJ-3_1 (*r7B*) TCGAGCCCATGACATTCCGCCTCACATCAGACCGTGAAGACGTACAGATATTCTACGTGG 700

AGJ-3_20 (*r7B*) TCGAGCCCATGACATTCCGCCTCACATCAGACCGTGAAGACGTACAGATATTCTACGTGG 700

AY198374.1 TCGAGCCCATGACATTCCGCCTCACATCAGACCGTGAAGACGTACAGATATTCTATGTGG 700

AGJ-2_20 (*r6A*) TCGAGCCCATGACATTCCGCCTCACATCAGACCGTGAAGACGTACAGATATTCTATGTGG 700

AGJ-2_10 (*r6A*) TCGAGCCCATGACATTCCGCCTCACATCAGACCGTGAAGACGTACAGATATTCTATGTGG 700

AGJ-2_15 (*r6A*) TCGAGCCCATGACATTCCGCCTCACATCAGACCGTGAAGACGTACAGATATTCTATGTGG 700

*********************************************:********* ****

AGJ-1_5 (*r5B*) AGCCAGCTCACATTACTGGTGATTGGTTCAACATGCAAATTACTATCGGTATCCTATCAG 757

AGJ-1_6 (*r5B*) AGCCAGCTCACATTACTGGTGATTGGTTCAACATGCAAATTACTATCGGTATCCTATCAG 757

AGJ-1_1 (*r5A*) AGCCAGCTCACATTACTGGTGATTGGTTCAACATGCAAATTACTATCGGTATCCTATCAG 760

AGJ-1_3 (*r5C*) AGCCAGCTCACATTACTGGTGATTGGTTCAACATGCAAATTACTATCGGTATCCTATCAG 780

AGJ-1_2 (*r5A*) AGCCAGCTCACATTACTGGTGATTGGTTCAACATGCAAATTACTATCGGTATCCTATCAG 760

AGJ-1_4 (*r5A*) AGCCAGCTCACATTACTGGTGATTGGTTCAACATGCAAATTACTATCGGTATCCTATCAG 760

AGJ-3_15 (*r7A*) AGCCAGCTCACATTACTGGAGATTGGTTCAACATGCAAATTACTATCGGTATCCTATCAG 760

AGJ-3_3 (*r7B*) AGCCAGCTCACATCACTGGAGATTGGTTCAACATGCAAATTACTATCGGTATCCTATCAG 760

AGJ-3_1 (*r7B*) AGCCAGCTCACATTACTGGAGATTGGTTCAACATGCAAATTACTATCGGTATCCTATCAG 760

AGJ-3_20 (*r7B*) AGCCAGCTCACATTACTGGAGATTGGTTCAACATGCAAATTACTATCGGTATCCTATCAG 760

AY198374.1 AGCCAGCTCACATTACTGGTGATTGGTTCAACATGCAAATTACTATCGGTATCCTATCAG 760

AGJ-2_20 (*r6A*) AGCCAGCTCACATTACTGGTGATTGGTTCAACATGCAAATTACTATCGGTATCCTATCAG 760

AGJ-2_10 (*r6A*) AGCCAGCTCACATTACTGGTGATTGGTTCAACATGCAAATTACTATCGGTATCCTATCAG 760

AGJ-2_15 (*r6A*) AGCCAGCTCACATTACTGGTGATTGGTTCAACATGCAAATTACTATCGGTATCCTATCAG 760

************* *****:****************************************

AGJ-1_5 (*r5B*) CGCTTAACTTCGAAAGCAACCCGCTGCACATCTTTCAAATCACTGCTTTGGACTCCTGGC 817

AGJ-1_6 (*r5B*) CGCTTAACTTCGAAAGCAACCCGCTGCACATCTTTCAAATCACTGCTTTGGACTCCTGGC 817

AGJ-1_1 (*r5A*) CGCTTAACTTCGAAAGCAACCCGCTGCACATCTTTCGAATCACTGCTTTGGACTCCTGGC 820

AGJ-1_3 (*r5C*) CGCTTAACTTCGAAAGCAACCCGCTGCACATCTTTCAAATCACTGCTTTGGACTCCTGGC 840

AGJ-1_2 (*r5A*) CGCTTAACTTCGAAAGCAACCCGCTGCACATCTTTCAAATCACTGCTTTGGACTCCTGGC 820

AGJ-1_4 (*r5A*) CGCTTAACTTCGAAAGCAACCCGCTGCACATCTTTCAAATCACTGCTTTGGACTCCTGGC 820

AGJ-3_15 (*r7A*) CGCTTAACTTCGAAAGCAACCCGCTTCACATCTTTCAAATCACTGCTTTGGACTCCTGGC 820

AGJ-3_3 (*r7B*) CGCTTAACTTCGAAAGCAACCCGCTTCACATCTTTCAAATCACTGCTTTGGACTCCTGGC 820

AGJ-3_1 (*r7B*) CGCTTAACTTCGAAAGCAACCCGCTTCACATCTTTCAAATCACTGCTTTGGACTCCTGGC 820

AGJ-3_20 (*r7B*) CGCTTAACTTCGAAAGCAACCCGCTTCACATCTTTCAAATCACTGCTTTGGACTCCTGGC 820

AY198374.1 CGCTTAACTTCGAAAGCAACCCGCTGCACATCTTTCAAATCACTGCTTTGGACTCCTGGC 820

AGJ-2_20 (*r6A*) CGCTTAACTTCGAAAGCAACCCGCTGCACATCTTTCAAATCACTGCTTTGGACTCCTGGC 820

AGJ-2_10 (*r6A*) CGCTTAACTTCGAAAGCAACCCGCTGCACATCTTTCAAATCACTGCTTTGGACTCCTGGC 820

AGJ-2_15 (*r6A*) CGCTTAACTTCGAAAGCAACCCGCTGCACATCTTTCAAATCACTGCTTTGGACTCCTGGC 820

************************* **********.***********************

AGJ-1_5 (*r5B*) CCAACAACCATACGGTGACGGTGATGGTGCAAGTCCAGAATGTGGAACACCGACCGCCGC 877

AGJ-1_6 (*r5B*) CCAACAACCATACGGTGACGGTGATGGTGCAAGTCCAGAATGTGGAACACCGACCGCCGC 877

AGJ-1_1 (*r5A*) CCAACAACCATACGGTGACGGTGATGGTGCAAGTCCAGAATGTGGAACACCGACCGCCGC 880

AGJ-1_3 (*r5C*) CCAACAACCATACGGTGACGGTGATGGTGCAAGTCCAGAATGTGGAACACCGACCGCCGC 900

AGJ-1_2 (*r5A*) CCAACAACCATACGGTGACGGTGATGGTGCAAGTCCAGAATGTGGAACACCGACCGCCGC 880

AGJ-1_4 (*r5A*) CCAACAACCATACGGTGACGGTGATGGTGCAAGTCCAGAATGTGGAACACCGACCGCCGC 880

AGJ-3_15 (*r7A*) CCAACAACCATACGGTGACGGTGATGGTGCAAGTCCAGAATGTGGAGCGCCGACCGCCGC 880

AGJ-3_3 (*r7B*) CCAACAACCATACGGTGACGGTGATGGTGCAAGTCCAGAATGTGGAGCACCGACCGCCGC 880

AGJ-3_1 (*r7B*) CCAACAACCATACGGTGACGGTGATGGTGCAAGTCCAGAATGTGGAGCACCGACCGCCGC 880

AGJ-3_20 (*r7B*) CCAACAACCATACGGTGACGGTGATGGTGCAAGTCCAGAATGTGGAGCACCGACCGCCGC 880

AY198374.1 CCAACAACCATACGGTGACGGTGATGGTGCAAGTTCAGAATGTGGAGCACCGACCGCCGC 880

AGJ-2_20 (*r6A*) CCAACAACCATGCGGTGACGGTGATGGTGCAAGTCCAGAATGTGGAACACCGACCGCCGC 880

AGJ-2_10 (*r6A*) CCAACAACCATACGGTGACGGTGATGGTGCAAGTCCAGAATGTGGAACACCGACCGCCGC 880

AGJ-2_15 (*r6A*) CCAACAACCATACGGTGACGGTGATGGTGCAAGTCCAGAATGTGGAACACCGACCGCCGC 880

***********.********************** ***********.*************

AGJ-1_5 (*r5B*) GATGGATGGAAATCTTCGCAGTCCAGCAGTTTGACGAGATGACGGAGCAGCAATTCCAGG 937

AGJ-1_6 (*r5B*) GATGGATGGAAATCTTCGCAGTCCAGCAGTTTGACGAGATGACGGAGCAGCAATTCCAGG 937

AGJ-1_1 (*r5A*) GATGGATGGAAATCTTCGCAGTCCAGCAGTTTGACGAGATGACGGAGCAGCAATTCCAGG 940

AGJ-1_3 (*r5C*) GATGGATGGAAATCTTCGCAGTCCAGCAGTTTGACGAGATGACGGAGCAGCAATTCCAGG 960

AGJ-1_2 (*r5A*) GATGGATGGAAATCTTCGCAGTCCAGCAGTTTGACGAGATGACGGAGCAGCAATTCCAGG 940

AGJ-1_4 (*r5A*) GATGGATGGAAATCTTCGCAGTCCAGCAGTTTGACGAGATGACGGAGCAGCAATTCCAGG 940

AGJ-3_15 (*r7A*) GATGGATGGAAATCTTCGCAGTCCAGCAGTTTGACGAGATGACGGAGCAGCAATTCCAGG 940

AGJ-3_3 (*r7B*) GATGGATGGAAATCTTCGCAGTCCAGCAGTTTGACGAGATGACGGAGCAGCAATTCCAGG 940

AGJ-3_1 (*r7B*) GATGGATGGAAATCTTCGCAGTCCAGCAGTTTGACGAGATGACGGAGCAGCAACTCCAGG 940

AGJ-3_20 (*r7B*) GATGGATGGAAATCTTCGCAGTCCAGCAGTTTGACGAGATGACGGAGCAGCAATTCCAGG 940

AY198374.1 GATGGATGGAAATCTTCGCAGTCCAGCAGTTTGACGAGATGACGGAGCAGCAATTCCAGG 940

AGJ-2_20 (*r6A*) GATGGATGGAAATCTTCGCAGTCCAGCAGTTTGACGAGATGACGGAGCAGCAATTCCAGG 940

AGJ-2_10 (*r6A*) GATGGATGGAAATCTTCGCAGTCCAGCAGTTTGACGAGATGACGGAGCAGCAATTCCAGG 940

AGJ-2_15 (*r6A*) GATGGATGGAAATCTTCGCAGTCCAGCAGTTTGACGAGATGACGGAGCAGCAATCCCAGG 940

***************************************************** *****

AGJ-1_5 (*r5B*) TGCGCGCCATCGACGGAGACACTGGCATCGGGAAAGCTATACACTATACCCTCGAGACAG 997

AGJ-1_6 (*r5B*) TGCGCGCCATCGACGGAGACACTGGCATCGGGAAAGCTATACACTATACCCTCGAGACAG 997

AGJ-1_1 (*r5A*) TGCGCGCCATCGACGGAGACACTGGCATCGGGAAAGCTATACACTATACCCTCGAGACAG 1000

AGJ-1_3 (*r5C*) TGCGCGCCATCGACGGAGACACTGGCATCGGGAAAGCTATACACTATACCCTCGAGACAG 1020

AGJ-1_2 (*r5A*) TGCGCGCCATCGACGGAGACACTGGCATCGGGAAAGCTATACACTATACCCTCGAGACAG 1000

AGJ-1_4 (*r5A*) TGCGCGCCATCGACGGAGACACTGGCATCGGGAAAGCTATACACTATACCCTCGAGACAG 1000

AGJ-3_15 (*r7A*) TGCGCGCCATCGACGGAGACACTGGCATCGGGAAAGCTATACACTATACCCTCGAGACAG 1000

AGJ-3_3 (*r7B*) TGCGCGCCATCGACGGAGACACTGGCATCGGGAAAGCTATACACTATACCCTCGAGACAG 1000

AGJ-3_1 (*r7B*) TGCGCGCCATCGACGGAGACACTGGCATCGGGAAAGCTATACACTATACCCTCGAGACAG 1000

AGJ-3_20 (*r7B*) TGCGCGCCATCGACGGAGACACTGGCATCGGGAAAGCTATACACTATACCCTCGAGACAG 1000

AY198374.1 TGCGCGCCATCGACGGAGACACTGGCATCGGGAAAGCTATACACTATACCCTCGAGACAG 1000

AGJ-2_20 (*r6A*) TGCGCGCCATCGACGGAGACACTGGCATCGGGAAAGCTATACACTATACCCTCGAGACAG 1000

AGJ-2_10 (*r6A*) TGCGCGCCATCGACGGAGACACTGGCATCGGGAAAGCTATACACTATACCCTCGAGACAG 1000

AGJ-2_15 (*r6A*) TGCGCGCCATCGACGGAGACACTGGCATCGGGAAAGCTATACACTATACCCTCGAGACAG 1000

************************************************************

AGJ-1_5 (*r5B*) ATGAGGAAGAAGATTTGTTCTTCATCGAAACACTTCCGGGCGGCCATGACGGAGCCATCT 1057

AGJ-1_6 (*r5B*) ATGAGGAAGAAGATTTGTTCTTCATCGAAACACTTCCGGGCGGCCATGACGGAGCCATCT 1057

AGJ-1_1 (*r5A*) ATGAGGAAGAAGATTTGTTCTTCATCGAAACACTTCCGGGCGGCCATGACGGAGCCATCT 1060

AGJ-1_3 (*r5C*) ATGAGGAAGAAGATTTGTTCTTCATCGAAACACTTCCGGGCGGCCATGACGGAGCCATCT 1080

AGJ-1_2 (*r5A*) ATGAGGAAGAAGATTTGTTCTTCATCGAAACACTTCCGGGCGGCCATGACGGAGCCATCT 1060

AGJ-1_4 (*r5A*) ATGAGGAAGAAGATTTGTTCTTCATCGAAACACTTCCGGGCGGCCATGACGGAGCCATCT 1060

AGJ-3_15 (*r7A*) ATGAGGAAGAAGATTTGTTCTTCATCGAAACACTTCCGGGCGGCCATGACGGAGCCATCT 1060

AGJ-3_3 (*r7B*) ATGAGGAAGAAGATTTGTTCTTCATCGGAACACTTCCGGGCGGCCATGACGGAGCCATCT 1060

AGJ-3_1 (*r7B*) ATGAGGAAGAAGATTTGTTCTTCATCGAAACACTTCCGGGCGGCCATGACGGAGCCATCT 1060

AGJ-3_20 (*r7B*) ATGAGGAAGAAGATTTGTTCTTCATCGAAACACTTCCGGGCGGCCATGACGGAGCCATCT 1060

AY198374.1 ATGAGGAAGAAGATTTGTTCTTCATCGAAACACTTCCGGGCGGCCATGACGGAGCCATCT 1060

AGJ-2_20 (*r6A*) ATGAGGAAGA-------------------------------------------------- 1010

AGJ-2_10 (*r6A*) ATGAGGAAGA-------------------------------------------------- 1010

AGJ-2_15 (*r6A*) ATGAGGAAGA-------------------------------------------------- 1010

**********

AGJ-1_5 (*r5B*) TCAGCACTGCCATGATTGATGTGGATAGGCTCCGGCGAGATGTCTTCAGACTGTCCCTGG 1117

AGJ-1_6 (*r5B*) TCAGCACTGCCATGATTGATGTGGATAGGCTCCGGCGAGATGTCTTCAGACTGTCCCTGG 1117

AGJ-1_1 (*r5A*) TCAGCACTGCCATGATTGATGTGGATAGGCTCCGGCGAGATGTCTTCAGACTGTCCCTGG 1120

AGJ-1_3 (*r5C*) TCAGCACTGCCATGATTGATGTGGATAGGCTCCGGCGAGATGTCTTCAGACTGTCCCTGG 1140

AGJ-1_2 (*r5A*) TCAGCACTGCCATGATTGATGTGGATAGGCTCCGGCGAGATGTCTTCAGACTGTCCCTGG 1120

AGJ-1_4 (*r5A*) TCAGCACTGCCATGATTGATGTGGATAGGCTCCGGCGAGATGTCTTCAGACTGTCCCTGG 1120

AGJ-3_15 (*r7A*) TCAGCACTGCCATGATTGATGTGGATAGGCTCCGGCGAGATGTCTTCAGACTGTCCCTGG 1120

AGJ-3_3 (*r7B*) TCAGCACTGCCATGATTGATGTGGATAGGCTCCGGCGAGATGTCTTCAGACTGTCCCTGG 1120

AGJ-3_1 (*r7B*) TCAGCACTGCCATGATTGATGTGGATAGGCTCCGGCGAGATGTCTTCAGACTGTCCCTGG 1120

AGJ-3_20 (*r7B*) TCAGCACTGCCATGATTGATGTGGATAGGCTCCGGCGAGATGTCTTCAGACTGTCCCTGG 1120

AY198374.1 TCAGCACTGCCATGATTGATGTGGATAGGCTCCGGCGAGATGTCTTCAGACTGTCCCTGG 1120

AGJ-2_20 (*r6A*) ------------------------------------------------------------ 1010

AGJ-2_10 (*r6A*) ------------------------------------------------------------ 1010

AGJ-2_15 (*r6A*) ------------------------------------------------------------ 1010

AGJ-1_5 (*r5B*) TGGTATACAAGTACGACAATGTGTCCTTCGCCACCCCGACACCCGTCGTGATCATAGTCA 1177

AGJ-1_6 (*r5B*) TGGCATACAAGTACGACAATGTGTCCTTCGCCACCCCGACACCCGTCGTGATCATAGTCA 1177

AGJ-1_1 (*r5A*) TGGCATACAAGTACGACAATGTGTCCTTCGCCACCCCGACACCCGTCGTGATCATAGTCA 1180

AGJ-1_3 (*r5C*) TGGCATACAAGTACGACAATGTGTCCTTCGCCACCCCGACACCCGTCGTGATCATAGTCA 1200

AGJ-1_2 (*r5A*) TGGCATACAAGTACGACAATGTGTCCTTCGCCACCCCGACACCCGTCGTGATCATAGTCA 1180

AGJ-1_4 (*r5A*) TGGCATACAAGTACGACAATGTGTCCTTCGCCACCCCGACACCCGTCGTGATCATAGTCA 1180

AGJ-3_15 (*r7A*) TGGCATACAAGTACGACAATGTGTCCTTCGCCACCCCGACACCCGTCGTGATCATAGTTA 1180

AGJ-3_3 (*r7B*) TGGCATACAAGTACGACAATGTGTCCTTCGCCACCCCGACACCCGTCGTGATCATAGTTA 1180

AGJ-3_1 (*r7B*) TGGCATACAAGTACGACAATGTGTCCTTCGCCACCCCGACACCCGTCGTGATCATAGTTA 1180

AGJ-3_20 (*r7B*) TGGCATACAAGTACGACAATGTGTCCTTCGCCACCCCGACACCCGTCGTGATCATAGTTA 1180

AY198374.1 TGGCATACAAGTACGACAATGTGTCCTTCGCCACCCCGACACCCGTCGTGATCATAGTCA 1180

AGJ-2_20 (*r6A*) ------------------------------------------------------------ 1010

AGJ-2_10 (*r6A*) ------------------------------------------------------------ 1010

AGJ-2_15 (*r6A*) ------------------------------------------------------------ 1010

AGJ-1_5 (*r5B*) ACGACATCAACAACAAGCAACCCCAACCGCTGCAAGATGAGTACACAATCTCCATAATGG 1237

AGJ-1_6 (*r5B*) ACGACATCAACAACAAGCAACCCCAACCGCTGCAAGATGAGTACACAATCTCCATAATGG 1237

AGJ-1_1 (*r5A*) ACGACATCAACAACAAGCAACCCCAACCGCTGCAAGATGAGTACACAATCTCCATAATGG 1240

AGJ-1_3 (*r5C*) ACGACATCAACAACAAGCAACCCCAACCGCTGCAAGATGAGTACACAATCTCCATAATGG 1260

AGJ-1_2 (*r5A*) ACGACATCAACAACAAGCAACCCCAACCGCTGCAAGATGAGTACACAATCTCCATAATGG 1240

AGJ-1_4 (*r5A*) ACGACATCAACAACAAGCAACCCCAACCGCTGCAAGATGAGTACACAATCTCCATAATGG 1240

AGJ-3_15 (*r7A*) ACGACATCAACAACAAGAAACCCCAACCGCTGCAAGATGAGTACACAATCTCCATAATGG 1240

AGJ-3_3 (*r7B*) ACGACATCAACAACAAGAAACCCCAACCGCTGCAAGATGAGTACACAATCTCCATAATGG 1240

AGJ-3_1 (*r7B*) ACGACATCAACAACAAGAAACCCCAACCGCTGCAAGATGAGTACACAATCTCCATAATGG 1240

AGJ-3_20 (*r7B*) ACGACATCAACAACAAGAAACCCCAACCGCTGCAAGATGAGTACACAATCTCCATAATGG 1240

AY198374.1 ATGACATCAACAACAAGAAACCCCAACCGCTGCAAGATGAGTACACAATCTCCATAATGG 1240

AGJ-2_20 (*r6A*) ------------------------------------------------------------ 1010

AGJ-2_10 (*r6A*) ------------------------------------------------------------ 1010

AGJ-2_15 (*r6A*) ------------------------------------------------------------ 1010

AGJ-1_5 (*r5B*) AAGAAACTCCACTGTCGCTGAATTTTGCTGAACCTTTTGGTTTCTATGATGAAGATTTGA 1297

AGJ-1_6 (*r5B*) AAGAAACTCCACTGTCGCTGAATTTTGCTGAACCTTTTGGTTTCTATGATGAAGATTTGA 1297

AGJ-1_1 (*r5A*) AAGAAACTCCACTGTCGCTGAATTTTGCTGAACTTTTTGGTTTCTATGATGAAGATTTGA 1300

AGJ-1_3 (*r5C*) AAGAAACTCCACTGTCGCTGAATTTTGCTGAACTTTTTGGTTTCTATGATGAAGATTTGA 1320

AGJ-1_2 (*r5A*) AAGAAACTCCACTGTCGCTGAATTTTGCTGAACTTTTTGGTTTCTATGATGAAGATTTGA 1300

AGJ-1_4 (*r5A*) AAGAAACTCCACTGTCGCTGAATTTTGCTGAACTTTTTGGTTTCTATGATGAAGATTTGA 1300

AGJ-3_15 (*r7A*) AAGAAACTCCACTGTCGCTGAATTTTGCTGAACTTTTTGGTTTCTATGATGAAGATTTGA 1300

AGJ-3_3 (*r7B*) AAGAAACTCCACTGTCGCTGAATTTTGCTGAACTTTTTGGTTTCTATGATGAAGATTTGA 1300

AGJ-3_1 (*r7B*) AAGAAACTCCACTGTCGCTGAATTTTGCTGAACTTTTTGGTTTCTATGATGAAGATTTGA 1300

AGJ-3_20 (*r7B*) AAGAAACTCCACTGTCGCTGAATTTTGCTGAACTTTTTGGTTTCTATGATGAAGATTTGA 1300

AY198374.1 AAGAAACTCCACTGTCGCTGAATTTTGCTGAACTTTTTGGTTTCTATGATGAAGATTTGA 1300

AGJ-2_20 (*r6A*) ------------------------------------------------------------ 1010

AGJ-2_10 (*r6A*) ------------------------------------------------------------ 1010

AGJ-2_15 (*r6A*) ------------------------------------------------------------ 1010

AGJ-1_5 (*r5B*) TCTACGCACAATTCTTGGTGGAAATACAAGGCGAGAACCCTCCAGGCGTAGAGCAAGCGT 1357

AGJ-1_6 (*r5B*) TCTACGCACAATTCTTGGTGGAAATACAAGGCGAGAACCCTCCAGGCGTAGAGCAAGCGT 1357

AGJ-1_1 (*r5A*) TCTACGCACAATTCTTGGTGGAAATACAAGGCGAGAACCCTCCAGGCGTAGAGCAAGCGT 1360

AGJ-1_3 (*r5C*) TCCACGCACAATTCTTGGTGGAAATACAAGGCGAGAACCCTCCAGGCGTAGAGCAAGCGT 1380

AGJ-1_2 (*r5A*) TCTACGCACAATTCTTGGTGGAAATACAAGGCGAGAACCCTCCAGGCGTAGAGCAAGCGT 1360

AGJ-1_4 (*r5A*) TCTACGCACAATTCTTGGTGGAAATACAAGGCGAGAACCCTCCAGGCGTAGAGCAAGCGT 1360

AGJ-3_15 (*r7A*) TCTACGCACAATTCTTGGTGGAGATACAAGGCGAGAACCCTCCAGGCGTAGAGCAAGCGT 1360

AGJ-3_3 (*r7B*) TCTACGCACAATTCTTGGTGGAAATACAAGGCGAGAACCCTCCAGGCGTAGAGCAAGCGT 1360

AGJ-3_1 (*r7B*) TCTACGCACAATTCTTGGTGGAAATACAAGGCGAGAACCCTCCAGGCGTAGAGCAAGCGT 1360

AGJ-3_20 (*r7B*) TCTACGCACAATTCTTGGTGGAAATACAAGGCGAGAACCCTCCAGGCGTAGAGCAAGCGT 1360

AY198374.1 TCTACGCACAATTCTTGGTGGAAATACAAGGCGAGAACCCTCCAGGCGTAGAGCAAGCGT 1360

AGJ-2_20 (*r6A*) ------------------------------------------------------------ 1010

AGJ-2_10 (*r6A*) ------------------------------------------------------------ 1010

AGJ-2_15 (*r6A*) ------------------------------------------------------------ 1010

AGJ-1_5 (*r5B*) TTTATATTGCGCCCACCGCAGGCTTCCAGAACCAGACATTCGCCATAGGGACTCAAGATC 1417

AGJ-1_6 (*r5B*) TTTATATTGCGCCCACCGCAGGCTTCCAGAACCAGACATTCGCCATAGGGACTCAAGATC 1417

AGJ-1_1 (*r5A*) TTTATATTGCGCCCACCGCAGGCTTCCAGAACCAGACATTCGCCATAGGGACTCAAGATC 1420

AGJ-1_3 (*r5C*) TTTATATTGCGCCCACCGCAGGCTTCCAGAACCAGACATTCGCCATAGGGACTCAAGATC 1440

AGJ-1_2 (*r5A*) TTTATATTGCGCCCGCCGCAGGCTTCCAGAACCAGACATTCGCCATAGGGACTCAAGATC 1420

AGJ-1_4 (*r5A*) TTTATATTGCGCCCACCGCAGGCTTCCAGAACCAGACATTCGCCATAGGGACTCAAGATC 1420

AGJ-3_15 (*r7A*) TTTATATTGCGCCCACCGCAGGCTTCCAGAACCAGACATTCGCCATAGGGACTCAAGATC 1420

AGJ-3_3 (*r7B*) TTTATATTGCGCCCACCGCAGGCTTCCAGAACCAGACATTCGCCATAGGGACTCAAGATC 1420

AGJ-3_1 (*r7B*) TTTATATTGCGCCCACCGCAGGCTTCCAGAACCAGACATTCGCCATAGGGACTCAAGATC 1420

AGJ-3_20 (*r7B*) TTTATATTGCGCCCACCGCAGGCTTCCAGAACCAGACATTCGCCATAGGGACTCAAGATC 1420

AY198374.1 TTTATATTGCGCCCACCGCAGGCTTCCAGAACCAGACATTCGCCATAGGGACTCAAGATC 1420

AGJ-2_20 (*r6A*) ------------------------------------------------------------ 1010

AGJ-2_10 (*r6A*) ------------------------------------------------------------ 1010

AGJ-2_15 (*r6A*) ------------------------------------------------------------ 1010

AGJ-1_5 (*r5B*) ACCGAATGCTGGATTATGAGGATGTTCCTTTCCAAAACATCAAGCTCAAGGTAATAGCAA 1477

AGJ-1_6 (*r5B*) ACCGAATGCTGGATTATGAGGATGTTCCTTTCCAAAACATCAAGCTCAAGGTAATAGCAA 1477

AGJ-1_1 (*r5A*) ACCGAATGCTGGATTATGAGGATGTTCCTTTCCAAAACATCAAGCTCAAGGTAATAGCAA 1480

AGJ-1_3 (*r5C*) ACCGAATGCTGGATTATGAGGATGTTCCTTTCCAAAACATCAAGCTCAAGGTAATAGCAA 1500

AGJ-1_2 (*r5A*) ACCGAATGCTGGATTATGAGGATGTTCCTTTCCAAAACATCAAGCTCAAGGTAATAGCAA 1480

AGJ-1_4 (*r5A*) ACCGAATGCTGGATTATGAGGATGTTCCTTTCCAAAACATCAAGCTCAAGGTAATAGCAA 1480

AGJ-3_15 (*r7A*) ACCGAATGCTGGATTATGAGGATGTTCCTTTCCAAAACATCGAGCTCAAGGTAATAGCAA 1480

AGJ-3_3 (*r7B*) ACCGAATGCTGGATTATGAGGATGTTCCTTTCCAAAACATCAAGCTCAAGGTAATAGCAA 1480

AGJ-3_1 (*r7B*) ACCGAATGCTGGATTATGAGGATGTTCCTTTCCAAAACATCAAGCTCAAGGTAATAGCAA 1480

AGJ-3_20 (*r7B*) ACCGAATGCTGGATTATGAGGATGTTCCTTTCCAAAACATCAAGCTTAAGGTAATAGCAA 1480

AY198374.1 ACCGAATGCTGGATTATGAGGATGTTCCTTTCCAAAACATCAAGCTCAAGGTAATAGCAA 1480

AGJ-2_20 (*r6A*) ------------------------------------------------------------ 1010

AGJ-2_10 (*r6A*) ------------------------------------------------------------ 1010

AGJ-2_15 (*r6A*) ------------------------------------------------------------ 1010

AGJ-1_5 (*r5B*) CGGACCGTGACAATACCAATTTTACTGGAGTCGCGGAAGTCAACGCGAACCTGATTAATT 1537

AGJ-1_6 (*r5B*) CGGACCGTGACAATACCAATTTTACTGGAGTCGCGGAAGTCAACGTGAGCCTGATTAATT 1537

AGJ-1_1 (*r5A*) CGGACCGCGACAATACCAATTTTACTGGAGTCGCGGAAGTCAACGTGAACCTGATTAATT 1540

AGJ-1_3 (*r5C*) CGGACCGTGACAATACCAATTTTACTGGAGTCGCGGAAGTCAACGTGAACCTGATTAATT 1560

AGJ-1_2 (*r5A*) CGGACCGTGACAATACCAATTTTACTGGAGTCGCGGAAGTCAACGTGAACCTGATTAATT 1540

AGJ-1_4 (*r5A*) CGGACCGTGACAATACCAATTTTACTGGAGTCGCGGAAGCCAACGTGAACCTGATTAATT 1540

AGJ-3_15 (*r7A*) CGGACCGTGACAATACCAATTTTACTGGAGTCGCGGAAGTCAACGTGAATCTGATTAATT 1540

AGJ-3_3 (*r7B*) CGGACCGTGACAATACCAATTTTACTGGAGTCGCGGAAGTCAACGTGAATCTGATTAATT 1540

AGJ-3_1 (*r7B*) CGGACCATGACAATACCAATTTTACTGGAGTCGCGGAAGTCAACGTGAATCTGATTAATT 1540

AGJ-3_20 (*r7B*) CGGACCGTGACAATACCAATTTTACTGGAGTCGCGGAAGTCAACGTGAATCTGATTAATT 1540

AY198374.1 CGGACCGTGACAATACCAATTTTACTGGAGTCGCGGAAGTCAACGTGAACCTGATTAATT 1540

AGJ-2_20 (*r6A*) ------------------------------------------------------------ 1010

AGJ-2_10 (*r6A*) ------------------------------------------------------------ 1010

AGJ-2_15 (*r6A*) ------------------------------------------------------------ 1010

AGJ-1_5 (*r5B*) GGAACGACGAGGAGCCGATCATTGAGGAAGACCAGCTCGTTGTCAAGTTCAAGGAGACTG 1597

AGJ-1_6 (*r5B*) GGAACGACGAGGAGCCGATCTTTGAGGAAGACCAGCTCGTTGTCAAGTTCAAGGAGACTG 1597

AGJ-1_1 (*r5A*) GGAACGACGAGGAGCCGATCTTTGAGGAAGACCAGCTCGTTGTCAAGTTCAAGGAGACTG 1600

AGJ-1_3 (*r5C*) GGAACGACGAGGAGCCGATCTTTGAGGAAGACCAGCTCGTTGTCAAGTTCAAGGAGACTG 1620

AGJ-1_2 (*r5A*) GGAACGACGAGGAGCCGATCTTTGAGGAAGACCAGCTCGTTGTCAAGTTCAAGGAGACTG 1600

AGJ-1_4 (*r5A*) GGAACGACGAGGAGCCGATCTTTGAGGAAGACCAGCTCATTGTCAAGTTCAAGGAGACTG 1600

AGJ-3_15 (*r7A*) GGAACGACGAGGAGCCGATCTTTGAGGAGGACCAGCTGGTTGTCAAGTTCAAGGAGACTG 1600

AGJ-3_3 (*r7B*) GGAACGACGAGGAGCCGATCTTTGAGGAGGACCAGCTGGTCGTCAAGTTCAAGGAGACTG 1600

AGJ-3_1 (*r7B*) GGAACGACGAGGAGCCGATCTTTGAGGAGGACCAGCTGGTTGTCAAGTTCAAGGAGACTG 1600

AGJ-3_20 (*r7B*) GGAACGACGAGGAGCCGATCTTTGAGGAGGACCAGCTGGTTGTCAAGTTCAAGGAGACTG 1600

AY198374.1 GGAACGACGAGGAGCCGATCTTTGAGGAAGACCAGCTCGTTGTCAAGTTCAAGGAGACTG 1600

AGJ-2_20 (*r6A*) ------------------------------------------------------------ 1010

AGJ-2_10 (*r6A*) ------------------------------------------------------------ 1010

AGJ-2_15 (*r6A*) ------------------------------------------------------------ 1010

AGJ-1_5 (*r5B*) TACCCAAGGACTATCACGTCGGCAGACTGAGGGCTCACGACCGGGACATAGGAGACAGCG 1657

AGJ-1_6 (*r5B*) TACCCAAGGACTATCACGTCGGCAGACTGAGGGCTCACGACCGGGACATAGGAGACAGCG 1657

AGJ-1_1 (*r5A*) TACCCAAGGACTATCACGTCGGCAGACTGAGGGCTCACGACCGGGACATAGGAGACAGCG 1660

AGJ-1_3 (*r5C*) TACCCAAGGACTATCACGTCGGCAGACTGAGGGCTCACGACCGGGACATAGGAGACAGCG 1680

AGJ-1_2 (*r5A*) TACCCAAGGACTATCACGTCGGCAGACTGAGGGCTCACGACCGGGACATAGGAGACAGCG 1660

AGJ-1_4 (*r5A*) TACCCAAGGACTATCACGTCGGCAGACTGAGGGCTCACGACCGGGACATAGGAGACAGCG 1660

AGJ-3_15 (*r7A*) TACCCAAGGACTATCACGTCGGCAGACTGAGGGCTCACGACCGGGACATAGGAGACAGCG 1660

AGJ-3_3 (*r7B*) TACCCAAGGACTATCACGTCGGCAGACTGAGGGCTCACGACCGGGACATAGGAGACAGCG 1660

AGJ-3_1 (*r7B*) TACCCAAGGACTATCACGTCGGCAGACTGAGGGCTCACGACCGGGACATAGGAGACAGCG 1660

AGJ-3_20 (*r7B*) TACCCAAGGACTATCACGTCGGCAGACTGAGGGCTCACGACCGGGACATAGGAGACAGCG 1660

AY198374.1 TACCCAAGGACTATCACGTCGGCAGACTGAGGGCTCACGACCGGGACATAGGAGACAGCG 1660

AGJ-2_20 (*r6A*) ------------------------------------------------------------ 1010

AGJ-2_10 (*r6A*) ------------------------------------------------------------ 1010

AGJ-2_15 (*r6A*) ------------------------------------------------------------ 1010

AGJ-1_5 (*r5B*) TTGTGCATTCCATCTTGGGAAATGCGAATACATTTTTAAGAATCGACGAAGAAACTGGCG 1717

AGJ-1_6 (*r5B*) TTGTGCATTCCATCTTGGGAAATGCGAATACATTTTTAAGAATCGACGAAGAAACTGGCG 1717

AGJ-1_1 (*r5A*) TTGTGCATTCCATCTTGGGAAATGCGAATACATTTTTAAGAATCGACGAAGAAACTGGCG 1720

AGJ-1_3 (*r5C*) TTGTGCATTCCATCTTGGGAAATGCGAATACATTTTTAAGAATCGACGAAGAAACTGGCG 1740

AGJ-1_2 (*r5A*) TTGTGCATTCCATCTTGGGAAATGCGAATACATTTTTAAGAATCGACGAAGAAACTGGCG 1720

AGJ-1_4 (*r5A*) TTGTGCATTCCATCTTGGGAAATGCGAATACATTTTTAAGAATCGACGAAGAAACTGGCG 1720

AGJ-3_15 (*r7A*) TTGTGCATTCCATCTTGGGAAATGCGAATACATTTTTGAGAATCGACGAAGAAACTGGCG 1720

AGJ-3_3 (*r7B*) TTGTGCATTCCATCTTGGGAAATGCGAATACATTTTTGAGAATCGACGAAGAAACTGGCG 1720

AGJ-3_1 (*r7B*) TTGTGCATTCCATCTTGGGAAATGCGAATACATTTTTGAGAATCGACGAAGAAACTGGCG 1720

AGJ-3_20 (*r7B*) TTGTGCATTCCATCTTGGGAAATGCGAATACATTTTTGAGAATCGACGAGGAAACTGGCG 1720

AY198374.1 TTGTGCATTCCATCTTGGGAAATGCGAATACATTTTTGAGAATCGACGAAGAAACTGGCG 1720

AGJ-2_20 (*r6A*) ------------------------------------------------------------ 1010

AGJ-2_10 (*r6A*) ------------------------------------------------------------ 1010

AGJ-2_15 (*r6A*) ------------------------------------------------------------ 1010

AGJ-1_5 (*r5B*) ACATCTACGTAACTATTGATGACGCGTTCGATTATCACAGACAGAATGAATTCAACATAC 1777

AGJ-1_6 (*r5B*) ACATCTACGTAACTATTGATGACGCGTTCGATTATCACAGACAGAATGAATTTAACATAC 1777

AGJ-1_1 (*r5A*) ACATCTACGTAACTATTGATGACGCGTTCGATTATCACAGACAGAATGAATTTAACATAC 1780

AGJ-1_3 (*r5C*) ACATCTACGTAACTATTGATGACGCGTTCGATTATCACAGACAGAATGAATTTAACATAC 1800

AGJ-1_2 (*r5A*) ACATCTACGTAACTATTGATGACGCGTTCGATTATCACAGACAGAATGAATTTAACATAC 1780

AGJ-1_4 (*r5A*) ACATCTACGTAACTATTGATGACGCGTTCGATTATCACAGACAGAATGAATTTAACATAC 1780

AGJ-3_15 (*r7A*) ACATCTACGTAGCTATTGATGACGCGTCCGATTATCACAGACAGAATGAATTTAACATAC 1780

AGJ-3_3 (*r7B*) ACATCTACGTAGCTATTGATGACGCGTTCGATTATCACAGACAGAATGAATTTAACATAC 1780

AGJ-3_1 (*r7B*) ACATCTACGTAGCTATTGATGACGCGTTCGATTATCACAGACAGAATGAATTTAATATAC 1780

AGJ-3_20 (*r7B*) ACATCTACGTAGCTATTGATGACGCGTTCGATTATCACAGACAGAATGAATTTAACATAC 1780

AY198374.1 ACATATACGTAGCTATTGATGACGCGTTCGATTATCACAGACAGAATGAATTTAACATAC 1780

AGJ-2_20 (*r6A*) ------------------------------------------------------------ 1010

AGJ-2_10 (*r6A*) ------------------------------------------------------------ 1010

AGJ-2_15 (*r6A*) ------------------------------------------------------------ 1010

AGJ-1_5 (*r5B*) AAGTTCGCGCTCAGGACACCATGTCGGAGCCAGAGTCCAGGCATACAGCGACTGCTCAGC 1837

AGJ-1_6 (*r5B*) AAGTTCGCGCTCAGGACACCATGTCGGAGCCAGAGTCCAGGCATACAGCGACTGCTCAGC 1837

AGJ-1_1 (*r5A*) AAGTTCGCGCTCAGGACACCATGTCGGAGCCAGAGTCCAGGCATACAGCGACTGCTCAGC 1840

AGJ-1_3 (*r5C*) AAGTTCGCGCTCAGGACACCATGTCGGAGCCAGAGTCCAGGCATACAGCGACTGCTCAGC 1860

AGJ-1_2 (*r5A*) AAGTTCGCGCTCGGGACACCATGTCGGAGCCAGAGTCCAGGCATACAGCGACTGCTCAGC 1840

AGJ-1_4 (*r5A*) AAGTTCGCGCTCAGGACACCATGTCGGAGCCAGAGTCCAGGCATACAGCGACTGCTCAGC 1840

AGJ-3_15 (*r7A*) AAGTTCGCGCTCAGGACACCATGTCGGAGCCAGAGTCCAGGCATACAGCGACTGCTCAGC 1840

AGJ-3_3 (*r7B*) AAGTTCGCGCTCAGGACACCATGTCGGAGCCAGAGTCCAGGCATACAGCGACTGCTCAGC 1840

AGJ-3_1 (*r7B*) AAGTTCGCGCTCAGGACACCATGTCGGAGCCAGAGTCCAGGCATACAGCGACTGCTCAGC 1840

AGJ-3_20 (*r7B*) AAGTTCGCGCTCAGGACACCATGTCGGAGCCAGAGTCCAGGCATACAGCGACTGCTCAGC 1840

AY198374.1 AAGTTCGCGCTCAGGACACCATGTCGGAGCCAGAGTCCAGGCATACAGCGACTGCTCAGC 1840

AGJ-2_20 (*r6A*) ------------------------------------------------------------ 1010

AGJ-2_10 (*r6A*) ------------------------------------------------------------ 1010

AGJ-2_15 (*r6A*) ------------------------------------------------------------ 1010

AGJ-1_5 (*r5B*) TGGTCATAGAACTCGAGGACGTCAACAACACACCTCCTACTCTGAGGCTGCCGCGCGTAA 1897

AGJ-1_6 (*r5B*) CGGTCATAGAACTCGAGGACGTCAACAACACACCTCCTACTCTGAGGCTGCCGCGCGTAA 1897

AGJ-1_1 (*r5A*) TGGTCATAGAACTCGAGGACGTCAACAACACACCTCCTACTCTGAGGCTGCCGCGCGTAA 1900

AGJ-1_3 (*r5C*) TGGTCATAGAACTCGAGGACGTCAACAACACACCTCCTACTCTGAGGCTGCCGCGCGTAA 1920

AGJ-1_2 (*r5A*) TGGTCATAGAACTCGAGGACGTCAACAACACACCTCCTACTCTGAGGCTGCCGCGCGTAA 1900

AGJ-1_4 (*r5A*) TGGTCATAGAACTCGAGGACGTCAACAACACACCTCCTACTCTGAGGCTGCCGCGCGTAA 1900

AGJ-3_15 (*r7A*) TGGTCATAGAACTCGAGGACGTCAACAACACACCTCCTACTCTGAGGCTGCCTCGCGTAA 1900

AGJ-3_3 (*r7B*) TGGTCATAGAACTCGAGGACGTCAACAACACACCTCCTACTCTGAGGCTGCCTCGCGTAA 1900

AGJ-3_1 (*r7B*) TGGTCATAGAACTCGAGGACGTCAACAACACACCTCCTACTCTGAGGCTGCCTCGCGTAA 1900

AGJ-3_20 (*r7B*) TGGTCATAGAACTCGAGGACGTCAACAACACACCTCCTACTCTGAGGCTGCCTCGCGTAA 1900

AY198374.1 TGGTCATAGAACTCGAGGACGTCAACAACACACCTCCTACTCTGAGGCTGCCTCGCGTAA 1900

AGJ-2_20 (*r6A*) ------------------------------------------------------------ 1010

AGJ-2_10 (*r6A*) ------------------------------------------------------------ 1010

AGJ-2_15 (*r6A*) ------------------------------------------------------------ 1010

AGJ-1_5 (*r5B*) GTCCGTCTGTAGAAGAGAATGTGCCAGAGGGCTTTGAAATCAACCGGGAGATAACCGCCA 1957

AGJ-1_6 (*r5B*) GTCCGTCTGTAGAAGAGAATGTGCCAGAGGGCTTTGAAATCAACCGGGAGATAACCGCCA 1957

AGJ-1_1 (*r5A*) GTCCGTCTGTAGAAGAGAATGTGCCAGAGGGCTTTGAAATCAACCGGGAGATAACCGCCA 1960

AGJ-1_3 (*r5C*) GTCCGTCTGTAGAAGAGAATGTGCCAGAGGGCTTTGAAATCAACCGGGAGATAACCGCCA 1980

AGJ-1_2 (*r5A*) GTCCGTCTGTAGAAGAGAATGTGCCAGAGGGCTTTGAAATCAACCGGGAGATAACCGCCA 1960

AGJ-1_4 (*r5A*) GTCCGTCTGTAGAAGAGAATGTGCCAGAGGGCTTTGAAATCAACCGGGAGATAACCGCCA 1960

AGJ-3_15 (*r7A*) GTCCGTCTGTAGAAGAGAATGTGCCAGAGGGCTTTGAAGTCAACCGGGAGATAACCGCCA 1960

AGJ-3_3 (*r7B*) GTCCGTCTGTAGAAGAGAATGTGCCAGAGGGCTTTGAAGTCAACCGGGAGATAACCGCCA 1960

AGJ-3_1 (*r7B*) GTCCGTCTGTAGAAGAGAATGTGCCAGAGGGCTTTGAAGTCAACCGGGAGATAACCGCCA 1960

AGJ-3_20 (*r7B*) GTCCGTCTGTAGAAGAGAATGTGCCAGAGGGCTTTGAAGTCAACCGGGAGATAACCGCCA 1960

AY198374.1 GTCCGTCTGTAGAAGAGAATGTGCCAGAGGGCTTTGAAATCAACCGGGAGATAACCGCCA 1960

AGJ-2_20 (*r6A*) ------------------------------------------------------------ 1010

AGJ-2_10 (*r6A*) ------------------------------------------------------------ 1010

AGJ-2_15 (*r6A*) ------------------------------------------------------------ 1010

AGJ-1_5 (*r5B*) CGGACCCCGACACCACAGCATACCTGCAGTTTGAAATAGATTGGGACACATCCTTTGCCA 2017

AGJ-1_6 (*r5B*) CGGACCCTGACACCACAGCATACCTGCAGTTTGAAATAGATTGGGACACATCCTTTGCCA 2017

AGJ-1_1 (*r5A*) CGGACCCTGACACCACAGCATACCTGCAGTTTGAAATAGATTGGGACACATCCTTTGCCA 2020

AGJ-1_3 (*r5C*) CGGACCCTGACACCACAGCATACCTGCAGTTTGAAATAGATTGGGACACATCCTTTGCCA 2040

AGJ-1_2 (*r5A*) CGGACCCTGACACCACAGCATACCTGCAGTTTGAAATAGATTGGGACACATCCTTTGCCA 2020

AGJ-1_4 (*r5A*) CGGACCCTGACACCACAGCATACCTGCAGTTTGAAATAGATTGGGACACATCCTTTGCCA 2020

AGJ-3_15 (*r7A*) CGGACCCTGACACCACAGCATACCTGCAGTTTGAAATAGATTGGGACACATCCTTTGCCA 2020

AGJ-3_3 (*r7B*) CGGACCCTGACACCACAGCATACCTGCAGTTTGAAATAGATTGGGACACATCCTTTGCCA 2020

AGJ-3_1 (*r7B*) CGGACCCTGACACCACAGCATACCTGCAGTTTGAAATAGATTGGGACACATCCTTTGCCA 2020

AGJ-3_20 (*r7B*) CGGACCCTGACACCACAGCATACCTGCAGTTTGAAATAGATTGGGACACATCCTTTGCCA 2020

AY198374.1 CGGACCCTGACACCACAGCATACCTGCAGTTTGAAATAGATTGGGACACATCCTTTGCCA 2020

AGJ-2_20 (*r6A*) ------------------------------------------------------------ 1010

AGJ-2_10 (*r6A*) ------------------------------------------------------------ 1010

AGJ-2_15 (*r6A*) ------------------------------------------------------------ 1010

AGJ-1_5 (*r5B*) CTAAACAGGGGCGTGATACCAATCCAGTAGAGTTCCACGGATGCGTGGATATAGAAACCA 2077

AGJ-1_6 (*r5B*) CTAAACAGGGGCGTGATACCAATCCAGTAGAGTTCCACGGATGCGTGGATATAGAAACCA 2077

AGJ-1_1 (*r5A*) CTAAACAGGGGCGTGATACCAATCCAGTAGAGTTCCACGGATGCGTGGATATAGAAACCA 2080

AGJ-1_3 (*r5C*) CTAAACAGGGGCGTGATACCAATCCAGTAGAGTTCCACGGATGCGTGGATATAGAAACCA 2100

AGJ-1_2 (*r5A*) CTAAACAGGGGCGTGATACCAATCCAGTAGAGTTCCACGGATGCGTGGATATAGAAACCA 2080

AGJ-1_4 (*r5A*) CTAAACAGGGGCGTGATACCAATCCAGTAGAGTTCCACGGATGCGTGGATATAGAAACCA 2080

AGJ-3_15 (*r7A*) CTAAACAGGGGCGTGATACCAATCCAGTAGAGTTCCACGGATGCGTGGATATAGAAACCA 2080

AGJ-3_3 (*r7B*) CTAAACAGGGGCGTGATACCAATCCAATAGAGTTCCACGGATGCGTGGATATAGAAACCA 2080

AGJ-3_1 (*r7B*) CTAAACAGGGGCGTGATACCAATCCAATAGAGTTCCACGGATGCGTGGATATAGAAACCA 2080

AGJ-3_20 (*r7B*) CTAAACAGGGGCGTGATACCAATCCAATAGAGTTCCACGGATGCGTGGATATAGAAACCA 2080

AY198374.1 CTAAACAGGGGCGTGATACCAATCCAATAGAGTTCCACGGATGCGTGGATATAGAAACCA 2080

AGJ-2_20 (*r6A*) -----------------------------------------TGCGTGGATATAGAAACCA 1029

AGJ-2_10 (*r6A*) -----------------------------------------TGCGTGGATATAGAAACCA 1029

AGJ-2_15 (*r6A*) -----------------------------------------TGCGTGGATATAGAAACCA 1029

*******************

AGJ-1_5 (*r5B*) TCTTCCCAAACCCAGCCGACACCAGAGAAGCTGTGGGGCGAGTGGTAGCGAAGGAGATCC 2137

AGJ-1_6 (*r5B*) TCTTCCCAAACCCAGCCGACACCAGAGAAGCTGTGGGGCGAGTGGTAGCGAAGGAGATCC 2137

AGJ-1_1 (*r5A*) TCTTCCCAAACCCAGCCGACACCAGAGAAGCTGTGGGGCGAGTGGTAGCGAAGGAGATCC 2140

AGJ-1_3 (*r5C*) TCTTCCCAAACCCAGCCGACACCAGAGAAGCTGTGGGGCGAGTGGTAGCGAAGGAGATCC 2160

AGJ-1_2 (*r5A*) TCATCCCAAACCCAGCCGACACTAGAGAAGCTGTGGGGCGAGTGGTAGCGAAGGAGATCC 2140

AGJ-1_4 (*r5A*) TCTTCCCAAACCCAGCCGACACCAGAGAAGCTGTGGGGCGAGTGGTAGCGAAGGAGATCC 2140

AGJ-3_15 (*r7A*) TCTTCCCAAACCCAGCCGACACCAGAGAAGCTGTGGGGCGAGTGGTAGCGAAGGAGATCC 2140

AGJ-3_3 (*r7B*) TCTTCCCAAACCCAGCCGACACCAGAGAGGCTGTGGGGCGAGTGGTAGCGAAGGAGATCC 2140

AGJ-3_1 (*r7B*) TCTTCCCAAACCCAGCCGACACCAGAGAGGCTGTGGGGCGAGTGGTAGTGAAGGAGATCC 2140

AGJ-3_20 (*r7B*) TCTTCCCAAACCCAGCCGACACCAGAGAGGCTGTGGGGCGAGTGGTAGCGAAGGAGATCC 2140

AY198374.1 TCTTCCCAAACCCAGCCGACACCAGAGAGGCTGTGGGGCGAGTGGTAGCGAAGGAGATCC 2140

AGJ-2_20 (*r6A*) TCTTCCCAAACCCAGCCGACACCAGAGAGGCTGTGGGGCGAGTGGTAGCGAAGGAGATCC 1089

AGJ-2_10 (*r6A*) TCTTCCCAAACCCAGCCGACACCAGAGAGGCTGTGGGGCGAGTGGTAGCGAAGGAGATCC 1089

AGJ-2_15 (*r6A*) TCTTCCCAAACCCAGCCGACACCAGAGAGGCTGTGGGGCGAGTGGTAGCGAAGGAGATCC 1089

**:******************* *****.******************* ***********

AGJ-1_5 (*r5B*) GCCATAACGTGACCATCGATTTTGAAGAGTTTGAATTTCTGTACCTCACAGTGAGAGTTC 2197

AGJ-1_6 (*r5B*) GCCATAACGTGACCATCGATTTTGAAGAGTTTGAATTTCTGTACCTCACAGTGAGAGTTC 2197

AGJ-1_1 (*r5A*) GCCATAACGTGACCATCGATTTTGGAGAGTTTGAATTTCTGTACCTCACAGTGAGAGTTC 2200

AGJ-1_3 (*r5C*) GCCATAACGTGACCATCGATTTTGAAGAGTTTGAATTTCTGTACCTCACAGTGAGAGTTC 2220

AGJ-1_2 (*r5A*) GCCATAACGTGACCATCGATTTTGAAGAGTTTGAATTTCTGTACCTCACAGTGAGAGTTC 2200

AGJ-1_4 (*r5A*) GCCATAACGTGACCATCGATTTTGAAGAGTTTGAATTTCTGTACCTCACAGTGAGAGTTC 2200

AGJ-3_15 (*r7A*) GCCATAACGTGACCATCGATTTTGAGGAGTTTGAATTTCTGTACCTCACAGTGAGAGTTC 2200

AGJ-3_3 (*r7B*) GCCATAACGTGACCATCGATTTTGAAGAGTTTGAATTTCTCTACCTCACAGTGAGAGTTC 2200

AGJ-3_1 (*r7B*) GCCATAACGTGACCATCGATTTTGAAGAGTTTGAATTTCTCTACCTCACAGTGAGAGTTC 2200

AGJ-3_20 (*r7B*) GCCATAACGTGACCATCGATTTTGAAGAGTTTGAATTTCTCTACCTCACAGTGAGAGTTC 2200

AY198374.1 GCCATAACGTGACCATCGATTTTGAAGAGTTTGAATTTCTCTACCTCACAGTGAGAGTTC 2200

AGJ-2_20 (*r6A*) GCCATAACGTGACCATCGATTTTGAAGAGTTTGAATTTCTCTACCTCACAGTGAGAGTTC 1149

AGJ-2_10 (*r6A*) GCCATAACGTGACCATCGATTTTGAAGAGTTTGAATTTCTCTACCTCACAGTGAGAGTTC 1149

AGJ-2_15 (*r6A*) GCCATAACGTGACCATCGATTTTGAAGAGTTTGAATTTCTCTACCTCACAGTGAGAGTTC 1149

************************..************** *******************

AGJ-1_5 (*r5B*) GAGACTTGCACACAGAAGATGGACGAGATTACGATGAATCTACCTTCACGATAATAATAA 2257

AGJ-1_6 (*r5B*) GAGACTTGCACACAGAAGATGGACGAGATTATGATGAATCTACCTTCACGATAATAATAA 2257

AGJ-1_1 (*r5A*) GAGACTTGCACACAGAAGATGGACGAGATTATGATGAATCTACCTTCACGATAATAATAA 2260

AGJ-1_3 (*r5C*) GAGACTTGCACACAGAAGATGGACGAGATTATGATGAATCTACCTTCACGATAATAATAA 2280

AGJ-1_2 (*r5A*) GAGACTTGCACACAGAAGATGGACGAGATTATGATGGATCTACCTTCACGATAATAATAA 2260

AGJ-1_4 (*r5A*) GAGACTTGCACACAGAAGATGGACGAGATTATGATGAATCTACCTTCACGATAATAATAA 2260

AGJ-3_15 (*r7A*) GAGACTTGCACACAGAAGATGGACGAGATTATGATGTGTCTACCTTCACGATAATAATAA 2260

AGJ-3_3 (*r7B*) GGGACTTGCACACAGATGACGGACGAGATTATGATGAATCTACCTTCACGATAATAATAA 2260

AGJ-3_1 (*r7B*) GGGACTTGCACACAGGTGACGGACGAGATTATGATGAATCTACCTTCACGATAATAATAA 2260

AGJ-3_20 (*r7B*) GGGACTTGCACACAGATGACGGACGAGATTATGATGAATCTACCTTCACGATAATAATAA 2260

AY198374.1 GGGACTTGCACACAGATGACGGACGAGATTATGATGAATCTACCTTCACGATAATAATAA 2260

AGJ-2_20 (*r6A*) GGGACTTGCACACAGATGACGGACGAGATTATGATGAATCTACCTTCACGATAATAATAA 1209

AGJ-2_10 (*r6A*) GGGACTTGCACACAGATGACGGACGAGATTATGATGAATCTACCTTCACGATAATAATAA 1209

AGJ-2_15 (*r6A*) GGGACTTGCACACAGATGACGGACGAGATTATGATGAATCTACCTTCACGATAATAATAA 1209

*.*************.:** *********** **** .**********************

AGJ-1_5 (*r5B*) TAGATATGAACGACAACTGGCCTATCTGGGCGTCTGGTTTCCTGAACCAGACCTTCAGTA 2317

AGJ-1_6 (*r5B*) TAGATATGAACGACAACTGGCCTATCTGGGCGTCTGGTTTCCTGAACCAGACCTTCAGTA 2317

AGJ-1_1 (*r5A*) TAGATATGAACGACAACTGGCCTACCTGGGCGTCTGGTTTCCTGAACCAGACCTTCAGTA 2320

AGJ-1_3 (*r5C*) TAGATATGAACGACAACTGGCCTATCTGGGCGTCTGGTTTCCTGAACCAGACCTTCAGTA 2340

AGJ-1_2 (*r5A*) TAGATATGAACGACAACTGGCCTATCTGGGCGTCTGGTTTCCTGAACCAGACCTTCAGTA 2320

AGJ-1_4 (*r5A*) TAGATATGAACGACAACTGGCCTATCTGGGCGTCTGGTTTCCTGAACCAGACCTTCAGTA 2320

AGJ-3_15 (*r7A*) TAGATATGAACGACAACTGGCCTATCTGGGCGTCTGGTTTCCTGAACCAGACCTTCAGTA 2320

AGJ-3_3 (*r7B*) TAGATATGAACGACAACCGGCCTATCTGGGCGTCTGGTTTCCTGAACCAGACCTTCAGTA 2320

AGJ-3_1 (*r7B*) TAGATATGAACGACAACTGGCCTATCTGGGCGTCTGGTTTCCTGAACCAGACCTTCAGTA 2320

AGJ-3_20 (*r7B*) TAGATATGAACGACAACTGGCCTATCTGGGCGTCTGGTTTCCTGAACCAGACCTTCAGTA 2320

AY198374.1 TAGATATGAACGACAACTGGCCTATCTGGGCGTCTGGTTTCCTGAACCAGACCTTCAGTA 2320

AGJ-2_20 (*r6A*) TAGATATGAACGACAACTGGCCTATCTGGGCGTCTGGTTTCCTGAACCAGACCTTCAGTA 1269

AGJ-2_10 (*r6A*) TAGATATGAACGACAACTGGCCTATCTGGGCGTCTGGTTTCCTGAACCAGACCTTCAGTA 1269

AGJ-2_15 (*r6A*) TAGATATGAACGACAACTGGCCTATCTGGGCGTCTGGTTTCCTGAACCAGACCTTCAGTA 1269

***************** ****** ***********************************

AGJ-1_5 (*r5B*) TTCGGGAGCGATCATCTACCGGCGTCGTCATCGGGTCCGTACTCGCTACAGACATTGATG 2377

AGJ-1_6 (*r5B*) TTCGGGAGCGATCATCTACCGGCGTCGTCATCGGGTCCGTACTCGCTACAGACATTGATG 2377

AGJ-1_1 (*r5A*) TTCGGGAGCGATCATCTACCGGCGTCGTCATCGGGTCCGTACTCGCTACAGACATTGATG 2380

AGJ-1_3 (*r5C*) TTCGGGAGCGATCATCTACCGGCGTCGTCATCGGGTCCGTACTCGCTACAGACATTGATG 2400

AGJ-1_2 (*r5A*) TTCGGGAGCGATCATCTACCGGCGTCGTCATCGGGTCCGTACTCGCTACAGACATTGATG 2380

AGJ-1_4 (*r5A*) TTCGGGAGCGATCATCTACCGGCGTCGTCATCGGGTCCGTACTCGCTACAGACATTGATG 2380

AGJ-3_15 (*r7A*) TTCGGGAGCGATCATCTACCGGCGTCGTCATCGGGTCCGTACTCGCTACAGACATTGATG 2380

AGJ-3_3 (*r7B*) TTCGGGAGCGATCATCTACCGGCGTCATCATCGGGTCCGTACTCGCTACAGACATTGATG 2380

AGJ-3_1 (*r7B*) TTCGGGAGCGATCATCTACCGGCGTCGTCATCGGGTCCGTACTCGCTACAGACATTGATG 2380

AGJ-3_20 (*r7B*) TTCGGGAGCGATCATCTACCGGCGTCGTCATCGGGTCCGTACTCGCTACAGACATTGATG 2380

AY198374.1 TTCGGGAGCGATCATCTACCGGCGTCGTCATCGGGTCCGTACTCGCTACAGACATTGATG 2380

AGJ-2_20 (*r6A*) TCCGGGAGCGATCATCTACCGGCGTCGTCATCGGGTCCGTACTCGCTACAGACATTGATG 1329

AGJ-2_10 (*r6A*) TCCGGGAGCGATCATCTACCGGCGTCGTCATCGGGTCCGTACTCGCTACAGACATTGATG 1329

AGJ-2_15 (*r6A*) TCCGGGAGCGATCATCTACCGGCGTCGTCATCGGGTCCGTACTCGCTACAGACATTGATG 1329

* ************************.*********************************

AGJ-1_5 (*r5B*) GCCCACTTTACAACCAAGTCCGGTACACCATTATCCCCCAGGAAGATACTCCTGAAGGTC 2437

AGJ-1_6 (*r5B*) GCCCACTTTACAACCAAGTCCGGTACACCATTATCCCCCAGGAAGATACTCCTGAAGGTC 2437

AGJ-1_1 (*r5A*) GCCCACTTTACAACCAAGTCCGGTACACCATTATCCCCCAGGAAGATACTCCTGAAGGTC 2440

AGJ-1_3 (*r5C*) GCCCACTTTACAACCAAGTCCGGTACACCATTATCCCCCAGGAAGATACTCCTGAAGGTC 2460

AGJ-1_2 (*r5A*) GCCCACTTTACAACCAAGTCCGGTACACCATTATCCCCCAGGAAGATACTCCTGAAGGTC 2440

AGJ-1_4 (*r5A*) GCCCACTTTACAACCAAGTCCGGTACACCATTATCCCCCAGGAAGATACTCCTGAAGGTC 2440

AGJ-3_15 (*r7A*) GCCCACTTTACAACCAAGTCCGGTACACCATTATCCCCCAGGAAGATACTCCTGAAGGTC 2440

AGJ-3_3 (*r7B*) GCCCACTTTACAACCAAGTCCGGTACACCATTATCCCCCAGGAAGATACTCCTGAAGGTC 2440

AGJ-3_1 (*r7B*) GCCCACTTTACAACCAAGTCCGGTACACCATTATCCCCCAGGAAGATACTCCTGAAGGTC 2440

AGJ-3_20 (*r7B*) GCCCACTTTACAACCAAGTCCGGTACACCATTATCCCCCAGGAAGATACTCCTGAAGGTC 2440

AY198374.1 GCCCACTTTACAACCAAGTCCGGTACACCATTATCCCCCAGGAAGATACTCCTGAAGGTC 2440

AGJ-2_20 (*r6A*) GCCCACTTTACAACCAAGTCCGGTACACCATTATCCCCCAGGAAGATACTCCTGAAGGTC 1389

AGJ-2_10 (*r6A*) GCCCACTTTACAACCAAGTCCGGTACACCATTATCCCCCAGGAAGATACTCCTGAAGGTC 1389

AGJ-2_15 (*r6A*) GCCCACTTTACAACCAAGTCCGGTACACCATTATCCCCCAGGAAGATACTCCTGAAGGTC 1389

************************************************************

AGJ-1_5 (*r5B*) TAGTCCAGATACACTTCGTTACGGGTCAGATTACAGTTGATGAGAATGGTGCAATCGACG 2497

AGJ-1_6 (*r5B*) TAGTCCAGATACACTTCGTTACGGGTCAGATTACAGTTGATGAGAATGGTGCAATCGACG 2497

AGJ-1_1 (*r5A*) TAGTCCAGATACACTTCGTTACGGGTCAGATTACAGTTGATGAGAGTGGTGCAATCGACG 2500

AGJ-1_3 (*r5C*) TAGTCCAGATACACTTCGTTACGGGTCAGATTACAGTTGATGAGAATGGTGCAATCGACG 2520

AGJ-1_2 (*r5A*) TAGTCCAGATACACTTCGTTACGGGTCAGATTACAGTTGATGAGAATGGTGCAATCGACG 2500

AGJ-1_4 (*r5A*) TAGTCCAGATACACTTCGTTACGGGTCAGATTACAGTTGATGAGAATGGTGCAATCGACG 2500

AGJ-3_15 (*r7A*) TAGTCCAGATACACTTCGTTACGGGTCAGATTACAGTTGATGAGAATGGTGCAATCGACG 2500

AGJ-3_3 (*r7B*) TAGTCCAGATACACTTCGTTACGGGTCAGATTACAGTTGATGAGAATGGTGCAATCGACG 2500

AGJ-3_1 (*r7B*) TAGTCCAGATACACTTCGTTACGGGTCAGATTACAGTTGATGAGAATGGTGCAATCGACG 2500

AGJ-3_20 (*r7B*) TAGTCCAGATACACTTCGTTACGGGTCAGATTACAGTTGATGAGAATGGTGCAATCGACG 2500

AY198374.1 TAGTCCAGATACACTTCGTTACGGGTCAGATTACAGTTGATGAGAATGGTGCAATCGACG 2500

AGJ-2_20 (*r6A*) TAGTCCAGATACACTTCGTTACGGGTCAGATTACAGTTGATGGGAATGGTGCAATCGACG 1449

AGJ-2_10 (*r6A*) TAGTCCAGATACACTTCGTTACGGGTCAGATTACAGTTGATGAGAATGGTGCAATCGACG 1449

AGJ-2_15 (*r6A*) TAGTCCAGATACACTTCGTTACGGGTCAGATTACAGTTGATGAGAATGGTGCAATCGACG 1449

******************************************.**.**************

AGJ-1_5 (*r5B*) CTGATATTCCACCTCGTTGGCACCTCAACTACACGGTTATAGCCAGCGACAAATGTTCTG 2557

AGJ-1_6 (*r5B*) CTGATATTCCACCTCGTTGGCACCTCAACTACACGGTTATAGCCAGCGACAAATGTTCTG 2557

AGJ-1_1 (*r5A*) CTGATATTCCACCTCGTTGGCACCTCAACTACACGGTTATAGCCAGCGACAAATGCTCTG 2560

AGJ-1_3 (*r5C*) CTGATATTCCACCTCGTTGGCACCTCAACTACACGGTTATAGCCAGCGACAAATGTTCTG 2580

AGJ-1_2 (*r5A*) CTGATATTCCACCTCGTTGGCACCTCAACTACACGGTTATAGCCAGCGACAAATGTTCTG 2560

AGJ-1_4 (*r5A*) CTGATATTCCACCTCGTTGGCACCTCAACTACACGGTTATAGCCAGCGACAAATGTTCTG 2560

AGJ-3_15 (*r7A*) CTGATATTCCACCTCGTTGGCACCTCAACTACACAGTTATAGCCAGCGACAAATGTTTCG 2560

AGJ-3_3 (*r7B*) CTGATATTCCGCCTCGTTGGCACCTCAACTACACAGTTATAGCCAGCGACAAATGTTCCG 2560

AGJ-3_1 (*r7B*) CTGATATTCCACCTCGTTGGCACCTCAACTACACAGTTATAGCCAGCGACATATGTTCCG 2560

AGJ-3_20 (*r7B*) CTGATATTCCACCTCGTTGGCACCTCAACTACACAGTTATAGCCAGCGACAAATGTTCCG 2560

AY198374.1 CTGATATTCCACCTCGTTGGCACCTCAACTACACGGTTATAGCCAGCGACAAATGTTCCG 2560

AGJ-2_20 (*r6A*) CTGATATTCCACCTCGTTGGCACCTCAACTACACGGTTATAGCCAGCGACAAATGTTCCG 1509

AGJ-2_10 (*r6A*) CTGATATTCCACCTCGTTGGCACCTCAACTACACGGTTATAGCCAGCGACAAATGTTCCG 1509

AGJ-2_15 (*r6A*) CTGATATTCCACCTCGTTGGCACCTCAACTACACGGTTATAGCCAGCGACAAATGTTCCG 1509

**********.***********************.****************:*** * *

AGJ-1_5 (*r5B*) AAGAAAATGAAGAGAACTGTCCCCCGGATCCAGTATTCTGGGATACTCTGCGCGACAACG 2617

AGJ-1_6 (*r5B*) AAGAAAATGAGGAGAACTGTCCCCCGGATCCAGTATTCTGGGATACTCTGCGCGACAACG 2617

AGJ-1_1 (*r5A*) AAGAAAATGAAGAGAACTGTCCCCCGGATCCAGTATTCTGGGATACTCTGCGCGACAACG 2620

AGJ-1_3 (*r5C*) AAGAAAATGAAGAGAACTGTCCCCCGGATCCAGTATTCTGGGATACTCTGCGCGACAACG 2640

AGJ-1_2 (*r5A*) AAGAAAATGAAGAGAACTGTCCCCCGGATCCAGTATTCTGGGATACTCTGCGCGACAACG 2620

AGJ-1_4 (*r5A*) AAGAAAATGAAGAGAACTGTCCCCCGGATCCAGTATTCTGGGATACTCTGCGCGACAACG 2620

AGJ-3_15 (*r7A*) AAGAAAATGAAGAGAACTGTCCCCCGGATCCAGTGTTCTGGGATACTCTGGGCGACAATG 2620

AGJ-3_3 (*r7B*) AAGAAAATGAAGAGAACTGTCCCCCGGATCCAGTGTTCTGGGATACTCTGGGCGACAATG 2620

AGJ-3_1 (*r7B*) AAGAAAATGAAGAGAACTGTCCCCCGGATCCAGTGTTCTGGGATACTCTGGGCGACAATG 2620

AGJ-3_20 (*r7B*) AAGAAAATGAAGAGAACTGTCCCCCGGATCCAGTGTTCTGGGATACTCTGGGCGACAATG 2620

AY198374.1 AAGAAAATGAAGAGAACTGTCCCCCGGATCCAGTGTTCTGGGATACTCTGGGCGACAATG 2620

AGJ-2_20 (*r6A*) AAGAAAATGAAGAGAACTGTCCCCCGGATCCAGTGTTCTGGAATACTCTGGGCGACAATG 1569

AGJ-2_10 (*r6A*) AAGAAAATGAAGAGAACTGTCCCCCGGATCCAGTGTTCTGGGATACTCTGGGCGACAATG 1569

AGJ-2_15 (*r6A*) AAGAAAATGAAGAGAACTGTCCCCCGGATCCAGTGTTCTGGGATACTCTGGGCGACAATG 1569

**********.***********************.******.******** ******* *

AGJ-1_5 (*r5B*) TAATTAACATCGTGGACATAAACAACAAGGTCCCGGCAGCAGACCTCAGTAGATTCAACG 2677

AGJ-1_6 (*r5B*) TAATTAACATCGTGGACATAGACAACAAGGTCCCGGCAGCAGACCTCAGTAGATTCAACG 2677

AGJ-1_1 (*r5A*) TAATTAACATCGTGGACATAAACAACAAGGTCCCGGCAGCAGACCTCAGTAGATTCAACG 2680

AGJ-1_3 (*r5C*) TAATTAACATCGTGGACATAAACAACAAGGTCCCGGCAGCAGACCTCAGTAGATTCAACG 2700

AGJ-1_2 (*r5A*) TAATTAACATCGTGGACATAAACAACAAGGTCCCGGCAGCAGACCTCAGTAGATTCAACG 2680

AGJ-1_4 (*r5A*) TAATTAACATCGTGGACATAAACAACAAGGTCCCGGCAGCAGACCTCAGTAGATTCAACG 2680

AGJ-3_15 (*r7A*) TAATTAACATCGTGGACATAAACAACAAGGTCCCGGCAGCAGACCTCAGTAGATTCAACG 2680

AGJ-3_3 (*r7B*) TAATTAACATCGTGGACATAAACAACAAGGTCCCGGCAGCAGACCTCAGTAGATTCAACG 2680

AGJ-3_1 (*r7B*) TAATTAACATCGTGGACATAAACAACAAGGTCCCGGCAGCAGACCTCAGTAGATTCAACG 2680

AGJ-3_20 (*r7B*) TAATTAACATCGTGGACATAAACAACAAGGTCCCGGCAGCAGACCTCAGTAGATTCAACG 2680

AY198374.1 TAATTAACATCGTGGACATAAACAACAAGGTCCCGGCAGCAGACCTCAGTCGATTCAACG 2680

AGJ-2_20 (*r6A*) TAATTAACATCGTGGACATAAACAACAAGGTCCCGGCAGCAGACCTCAGTCGATTCAACG 1629

AGJ-2_10 (*r6A*) TAATTAACATCGTGGACATAAACAACAAGGTCCCGGCAGCAGACCTCAGTCGATTCAACG 1629

AGJ-2_15 (*r6A*) TAATTAACATCGTGGACATAAACAACAAGGTCCCGGCAGCGGACCTCAGTCGATTCAACG 1629

********************.*******************.*********.*********

AGJ-1_5 (*r5B*) AAACGGTGTACATTTATGAAAATGCACCCGATTTCACAAACGTGGTCAAGATATACTCCA 2737

AGJ-1_6 (*r5B*) AAACGGTGTACATTTATGAAAATGCACCCGATTTCACAAACGTAGTCAAGATATACTCCA 2737

AGJ-1_1 (*r5A*) AAACGGTGTACATTTATGAAAATGCACCCGATTTCACAAACGTGGTCAAGATATACTCCA 2740

AGJ-1_3 (*r5C*) AAACGGTGTACATTTATGAA-ATGCACCCGATTTCACAAACGTGGTCAAGATATACTCCA 2759

AGJ-1_2 (*r5A*) AAACGGTGTACATTTATGAAAATGCACCCGATTTCACAAACGTGGTCAAGATATACTCCA 2740

AGJ-1_4 (*r5A*) AAACGGTGTACATTTATGAAAATGCACCCGATTTCACAAACGTGGTCAAGATATACTCCA 2740

AGJ-3_15 (*r7A*) AAACGGTGTACATTTATGAAAATGCACCCGATTTCACAAACGTGGTCAAGATATACTCCA 2740

AGJ-3_3 (*r7B*) AAACGGTGTACATTTATGAAAATGCACCCGATTTCACAAACGTGGTCAAGATATACTCCA 2740

AGJ-3_1 (*r7B*) AAACGGTGTACATTTATGAAAATGCACCCGATTTCACAAACGTGGTCAAGATACACTCCA 2740

AGJ-3_20 (*r7B*) AAACGGTGTACATTTATGAAAATGCACCCGATTTCACAAACGTGGTCAAGATATACTCCA 2740

AY198374.1 AAACGGTGTACATTTATGAAAATGCACCCGATTTCACAAACGTGGTCAAGATATACTCCA 2740

AGJ-2_20 (*r6A*) AAACGGTGTACATTTATGAAAATGCACCCGATTTCACAAACGTGGTCAAGATATACTCCA 1689

AGJ-2_10 (*r6A*) AAACGGTGTACATTTATGAAAATGCACCCGATTTCACAAACGTGGTCAAGATATACTCCA 1689

AGJ-2_15 (*r6A*) AAACGGTGTACATTTATGAAAATGCACCCGATTTCACAAACGTGGTCAAGATATACTCCA 1689

******************** **********************.********* ******

AGJ-1_5 (*r5B*) TCGACGAAGACAGAGACGAAATATATCACACGGTGCGGTACCAGATCAATTATGCTGTGA 2797

AGJ-1_6 (*r5B*) TCGACGAAGACAGAGACGAAATATATCACACGGTGCGGTACCAGATCAATTATGCTGTGA 2797

AGJ-1_1 (*r5A*) TCGACGAAGACAGAGACGAAGTATATCACACGGTGCGGTACCAGATCAATTATGCTGTGA 2800

AGJ-1_3 (*r5C*) TCGACGAAGACAGAGACGAAATATATCACACGGTGCGGTACCAGATCAATTATGCTGTGA 2819

AGJ-1_2 (*r5A*) TCGACGAAGACAGAGACGAAATATATCACACGGTGCGGTACCAGATCAATTATGCTGTGA 2800

AGJ-1_4 (*r5A*) TCGACGAAGACAGAGACGAAATATATCACACGGTGCGGTACCAGATCAACTATGCTGTGA 2800

AGJ-3_15 (*r7A*) TCAACGAAGACAGAGACGAAATATATCACACGGTGCGGTACCAGATCAATTATGCTGTGA 2800

AGJ-3_3 (*r7B*) TCGACGAAGACAGAGACGAAATATATCACACGGTGCGGTACCAGATCAATTATGCTGTGA 2800

AGJ-3_1 (*r7B*) TCGACGAAGACAGAGACGAAATATATCACACGGTGCGGTACCAGATCAATTATGCTGTGA 2800

AGJ-3_20 (*r7B*) TCGACGAAGACAGAGACGAAATATATCACACGGTGCGGTACCAGATCAATTATGCTGTGA 2800

AY198374.1 TCGACGAAGACAGAGACGAAATATATCACACGGTGCGGTACCAGATCAATTATGCTGTGA 2800

AGJ-2_20 (*r6A*) TCGACGAAGACAGAGACGAAATATATCGCACGGTGCGGTACCAGATCAATTATGCTGTGA 1749

AGJ-2_10 (*r6A*) TCGACGAAGACAGAGACGAAATATATCACACGGTGCGGTACCAGATCAATTATGCTGTGA 1749

AGJ-2_15 (*r6A*) TCGACGAAGACAGAGACGAAATATATCACACGGTGCGGTACCAGATCAATTATGCTGTGA 1749

**.*****************.******.********************* **********

AGJ-1_5 (*r5B*) ACCAGCGGCTGCGAGATTTCTTCGCCATAGACCTGGATTCAGGCCAGGTGTACGTGGAGA 2857

AGJ-1_6 (*r5B*) ACCAGCGGCTGCGAGATTTCTTCGCCATAGACCTGGATTCAGGCCAGGTGTACGTGGAGA 2857

AGJ-1_1 (*r5A*) ACCAGCGGCTGCGAGATTTCTTCGCCATAGACCTGGATTCAGGCCAGGTGTACGTGGAGA 2860

AGJ-1_3 (*r5C*) ACCAGCGGCTGCGAGATTTCTTCGCCATAGACCTGGATTCAGGCCAGGTGTACGTGGAGA 2879

AGJ-1_2 (*r5A*) ACCAGCGGCTGCGAGATTTCTTCGCCATAGACCTGGATTCAGGCCAGGTGTACGTGGAGA 2860

AGJ-1_4 (*r5A*) ACCAGCGGCTGCGAGATTTCTTCGCCATAGACCTGGATTCAGGCCAGGTGTACGTGGAGA 2860

AGJ-3_15 (*r7A*) ACCAGCGGCTGCGAGACTTCTTCGCCATAGACCTGGATTCAGGCCAGGTGTACGTGGAGA 2860

AGJ-3_3 (*r7B*) ACCAGCGGCTGCGAGACTTCTTCGCCATAGACCTGGATTCAGGCCAGGTGTACGTAGAGA 2860

AGJ-3_1 (*r7B*) ACCAGCGGCTGCGAGACTTCTTCGCCATAGACCTGGATTCAGGCCAGGTGTACGTGGAGA 2860

AGJ-3_20 (*r7B*) ACCAGCGGCTGCGAGACTTCTTCGCCATAGACCTGGATTCAGGCCAGGTGTACGTGGAGA 2860

AY198374.1 ACCAGCGGCTGCGAGACTTCTTCGCCATAGACCTGGATTCAGGCCAGGTGTACGTGGAGA 2860

AGJ-2_20 (*r6A*) ACCAGCGGCTGCGAGACTTCTTCGCCATAGACCTGGATTCAGGCCAGGTGTACGTGGAGA 1809

AGJ-2_10 (*r6A*) ACCAGCGGCTGCGAGACTTCTTCGCCATAGACCTGGATTCAGGCCAGGTGTACGTGGAGA 1809

AGJ-2_15 (*r6A*) ACCAGCGGCTGCGAGACTTCTTCGCCATAGACCTGGATTCAGGCCAGGTGTACGTGGAGA 1809

**************** **************************************.****

AGJ-1_5 (*r5B*) ACACCAACAATGAGCTCCTGGATCGGGACAGAGGCGAAGACCAACACAGGATATTCATTA 2917

AGJ-1_6 (*r5B*) ACACCAACAATGAGCTCCTGGATCGGGACAGAGGCGAAGACCAACACAGGATATTCATTA 2917

AGJ-1_1 (*r5A*) ACACCAACAATGAGCTCCTGGATCGGGACAGAGGCGAAGACCAACACAGGATATTCATTA 2920

AGJ-1_3 (*r5C*) ACACCAACAATGAGCTCCTGGATCGGGACAGAGGCGAAGACCAACACAGGATATTCATTA 2939

AGJ-1_2 (*r5A*) ACACCAACAATGAGCTCCTGGATCGGGACAGGGGCGAAGACCAACACAGGATATTCATTA 2920

AGJ-1_4 (*r5A*) ACACCAACAATGAGCTCCTGGATCGGGACAGAGGCGAAGACCAACACAGGATATTCATTA 2920

AGJ-3_15 (*r7A*) ACACCAACAATGAGCTCCTGGATCGGGACAGAGGCGAAGACCAACACAGGATATTCATTA 2920

AGJ-3_3 (*r7B*) ACACCAACAATGAGCTCCTGGATCGGGACAGAGGCGAAGACCAACACAGGATATTCATTA 2920

AGJ-3_1 (*r7B*) ACACCAACAATGAGCTCCTGGATCGGGACAGAGGCGAAGACCAACACAGGATATTCATTA 2920

AGJ-3_20 (*r7B*) ACACCAACAATGAGCTCCTGGATCGGGACAGAGGCGAAGACCAACACAGGATATTCATTA 2920

AY198374.1 ACACCAACAATGAGCTCCTGGATCGGGACAGAGGCGAAGACCAACACAGGATATTCATTA 2920

AGJ-2_20 (*r6A*) ACACCAACAATGAGCTCCTGGATCGGGACAGAGGCGAGGACCAACACAGGATATTCATTA 1869

AGJ-2_10 (*r6A*) ACACCAACAATGAGCTCCTGGATCGGGACAGAGGCGAGGACCAACACAGGATATTCATTA 1869

AGJ-2_15 (*r6A*) ACACCAACAATGAGCTCCTGGATCGGGACAGAGGCGAGGACCAACACAGGATATTCATTA 1869

*******************************.*****.**********************

AGJ-1_5 (*r5B*) ACCTCATTGACAACTTTTATAGCGAAGGAGATGGAAATAGAAATGTAAACACTACAGAGG 2977

AGJ-1_6 (*r5B*) ACCTCATTGACAACTTTTATAGCGAAGGAGATGGAAATAGAAATGTAAACACTACAGAGG 2977

AGJ-1_1 (*r5A*) ACCTCATTGACAACTTTTATAGCGAAGGAGATGGAAATAGAAATGTAAACACTACAGAGG 2980

AGJ-1_3 (*r5C*) ACCTCATTGACAACTTTTATAGCGAAGGAGATGGAAATAGAAATGTAAACACTACAGAGG 2999

AGJ-1_2 (*r5A*) ACCTCATTGACAACTTTTATAGCGAAGGAGATGGAAATAGAAATGTAAACACTACAGAGG 2980

AGJ-1_4 (*r5A*) ACCTCATTGACAACTTCTATAGCGAAGGAGATGGAAATAGAAATGTAAACACTACAGAGG 2980

AGJ-3_15 (*r7A*) ACCTCATCGACAACTTTTATAGCGAAGGAGATGGAAATAGAAATGTAAACACTACAGAGG 2980

AGJ-3_3 (*r7B*) ACCTCATTGACAACTTTTATAGCGAAGGAGATGGAAATAGAAATGTAAACACTACAGAGG 2980

AGJ-3_1 (*r7B*) ACCTCATTGACAACTTTTATAGCGAAGGAGATGGAAATAGAAATGTAAACACTACAGAGG 2980

AGJ-3_20 (*r7B*) ACCTCATTGACAACTTTTATAGCGAAGGAGATGGAAATAGAAATGTAAACACTACAGAGG 2980

AY198374.1 ACCTCATTGACAACTTTTATAGCGAAGGAGATGGAAATAGAAATGTAAACACTACAGAGG 2980

AGJ-2_20 (*r6A*) ACCTCATTGACAACTTTTATAGCGAAGGAGATGGAAATAGAAATGTAAACACTACAGAGG 1929

AGJ-2_10 (*r6A*) ACCTCATTGACAACTTTTATAGCGAAGGAGATGGAAATAGAAATGTAAACACTACAGAGG 1929

AGJ-2_15 (*r6A*) ACCTCATTGACAACTTTTATAGCGAAGGAGATGGAAATAGAAATGTAAACACTACAGAGG 1929

******* ******** *******************************************

AGJ-1_5 (*r5B*) TGCTGGTGATACTATTAGATGAGAATGACAACGCTCCTGAATTGCCGACTCCAGAAGAGC 3037

AGJ-1_6 (*r5B*) TGCTGGTGATACTATTAGATGAGAATGACAACGCTCCTGAATTGCCGACTCCAGAAGAGC 3037

AGJ-1_1 (*r5A*) TGCTGGTGATACTATTAGATGAGAATGACAACGCTCCTGAATTGCCGACTCCAGAAGAGC 3040

AGJ-1_3 (*r5C*) TGCTGGTGATACTATTAGATGAGAATGACAACGCTCCTGAATTGCCGACTCCAGAAGAGC 3059

AGJ-1_2 (*r5A*) TGCTGGTGATACTATTAGATGAGAATGACAACGCTCCTGAATTGCCGACTCCAGAAGAGC 3040

AGJ-1_4 (*r5A*) TGCTGGTGATACTATTAGATGAGAATGACAACGCTCCTGAATTGCCGACTCCAGAAGAGC 3040

AGJ-3_15 (*r7A*) TGCTGGTGATACTATTAGATGAGAATGACAACGCTCCTGAATTGCCGACTCCAGAAGAGC 3040

AGJ-3_3 (*r7B*) TGCTGGTGATACTATTAGATGAGAATGACAACGCTCCTGAATTGCCGACTCCAGAAGAGC 3040

AGJ-3_1 (*r7B*) TGCTGGTGATACTATTAGATGAGAATGACAACGCTCCTGAATTGCCGACTCCAGAAGAGC 3040

AGJ-3_20 (*r7B*) TGCTGGTGATACTATTAGATGAGAATGACAACGCTCCTGAATTGCCGACTCCAGAAGAGC 3040

AY198374.1 TGCTGGTGATACTATTAGATGAGAATGACAACGCTCCTGAATTGCCGACTCCAGAAGAGC 3040

AGJ-2_20 (*r6A*) TGCTGGTGATACTATTAGATGAAAATGACAACGCTCCTGAATTGCCGACTCCAGAAGAGC 1989

AGJ-2_10 (*r6A*) TGCTGGTGATACTATTAGATGAAAATGACAACGCTCCTGAATTGCCGACCCCAGAAGAGC 1989

AGJ-2_15 (*r6A*) TGCTGGTGATACTATTAGATGAAAATGACAACGCTCCTGAATTGCCGACTCCAGAAGAGC 1989

**********************.************************** **********

AGJ-1_5 (*r5B*) TGAGTTGGAGCATTTCCGAGAATTTACAAGAGGGTATAACACTCGATGGCGAAAGCGATG 3097

AGJ-1_6 (*r5B*) TGAGTTGGAGCATTTCCGAGAATTTACAAGAGGGTATAACACTCGATGGCGAAAGCGATG 3097

AGJ-1_1 (*r5A*) TGAGTTGGAGCATTTCCGAGAATCTACAAGAGGGTATAACACTCGATGGCGAAAGCGATG 3100

AGJ-1_3 (*r5C*) TGAGTTGGAGCATTTCCGAGAATTTACAAGAGGGTATAACACTCGATGGCGAAAGCGATG 3119

AGJ-1_2 (*r5A*) TGAGTTGGAGCATTTCCGAGAATTTACAAGAGGGTATAACACTCGATGGCGAAAGCGATG 3100

AGJ-1_4 (*r5A*) TGAGTTGGAGCATTTCCGAGAATTTACAAGAGGGTATAACACTCGATGGCGAAAGCGATG 3100

AGJ-3_15 (*r7A*) TGAGTTGGAGCATTTCCGAGAATTTACAAGAGGGTATAACACTCGATGGCGAAAGCGATG 3100

AGJ-3_3 (*r7B*) TGAGTTGGAGCATTTCCGAGAATTTACAAGAGGGTATAACACTCGATGGCGAAAGCGATG 3100

AGJ-3_1 (*r7B*) TGAGTTGGAGCATTTCCGAGAATTTACAAGAGGGTATAACACTCGATGGCGAAAGCGATG 3100

AGJ-3_20 (*r7B*) TGAGTTGGAGCATTTCCGAGAATTTACAAGAGGGTATAACACTCGATGGCGAAAGCGATG 3100

AY198374.1 TGAGTTGGAGCATTTCCGAGAATTTACAAGAGGGTATAACACTCGATGGCGAAAGCGATG 3100

AGJ-2_20 (*r6A*) TGAGTTGGAGCATTTCCGAGAATTTACAAGAGGGTATAACACTCGATGGCGAACGCGATG 2049

AGJ-2_10 (*r6A*) TGAGTTGGAGCATTTCCGAGAATTTACAAGAGGGTATAACACTCGATGGCGAACGCGATG 2049

AGJ-2_15 (*r6A*) TGAGTTGGAGCATTTCCGAGAATTTACAAGAGGGTATAACACTCGATGGCGAACGCGATG 2049

*********************** *****************************.******

AGJ-1_5 (*r5B*) TGATATACGCACCGGATATAGCCGAAGAGGACACGCCAAACTCTCACGTTGGCTACGCAA 3157

AGJ-1_6 (*r5B*) TGATATACGCACCGGATATAGACGAAGAGGACACGCCAAACTCTCACGTTGGCTACGCAA 3157

AGJ-1_1 (*r5A*) TGATATACGCACCGGATATAGACGAAGAGGACACGCCAAACTCTCACGTTGGCTACGCAA 3160

AGJ-1_3 (*r5C*) TGATATACGCACCGGATATAGACGAAGAGGACACGCCAAACTCTCACGTTGGCTACGCAA 3179

AGJ-1_2 (*r5A*) TGATATACGCACCGGATATAGACGAAGAGGACACGCCAAACTCTCACGTTGGCTACGCAA 3160

AGJ-1_4 (*r5A*) TGATATACGCACCGGATATAGACGAAGAGGACACGCCAAACTCTCACGTTGGCTACGCAA 3160

AGJ-3_15 (*r7A*) TGATATACGCACCGGATATAGACGAAGAGGACACGCCAAACTCTCACGTTGGCTACGCAA 3160

AGJ-3_3 (*r7B*) TGATATACGCACCGGATATAGACGAAGAGGACACGCCAAACTCTCACGTTGGCTACGCAA 3160

AGJ-3_1 (*r7B*) TGATATACGCACCGGATATAGACGAAGAGGACACGCCAAACTCTCACGTTGGCTACGCAA 3160

AGJ-3_20 (*r7B*) TGATATACGCACCGGATATAGACGAAGAGGACACGCCAAACTCTCACGTTGGCTACGCAA 3160

AY198374.1 TGATATACGCACCGGATATAGACGAAGAGGACACGCCAAACTCTCACGTTGGCTACGCAA 3160

AGJ-2_20 (*r6A*) TAATATACGCACCGGATATAGACGAAGAGGACACGCCAAACTCTCACGTTGGCTACGCAA 2109

AGJ-2_10 (*r6A*) TAATATACGCACCGGATATAGACGAAGAGGACACGCCAAACTCTCACGTTGGCTACGCAA 2109

AGJ-2_15 (*r6A*) TAATATACGCACCGGATATAGACGAAGAGGACACGCCAAACTCTCACGTTGGCTACGCAA 2109

*.*******************.**************************************

AGJ-1_5 (*r5B*) TCCTGGCCATGACAGTCACCAATAGAGACCTGGACACTGTTCCGAGTCTTCTCAACATGC 3217

AGJ-1_6 (*r5B*) TCCTGGCCATGACAGTCACCAATAGAGACCTGGACACTGTTCCGAGACTTCTCAACATGC 3217

AGJ-1_1 (*r5A*) TCCTGGCCATGACAGTCACCAATAGAGACCTGGACACTGTTCCGAGACTTCTCAACATGC 3220

AGJ-1_3 (*r5C*) TCCTGGCCATGACAGTCACCAATAGAGACCTGGACACTGTTCCGAGACTTCTCAACATGC 3239

AGJ-1_2 (*r5A*) TCCTGGCCATGACAGTCACCAATAGAGACCTGGACACTGTTCCGAGACTTCTCAACATGC 3220

AGJ-1_4 (*r5A*) TCCTGGCCATGACAGTCACCAATAGAGACCTGGACACTGTTCCGAGACTTCTCAACATGC 3220

AGJ-3_15 (*r7A*) TCCTGGCCATGACAGTCACCAATAGAGACCTGGACACTGTTCCGAGACTTCTCAACATGC 3220

AGJ-3_3 (*r7B*) TCCTGGCCATGACAGTCACCAATAGAGACCTGGACACTGTTCCGAGACTTCTCAACATGC 3220

AGJ-3_1 (*r7B*) TCCTGGCCATGACAGTCACCAATAGAGACCTGGACACTGTTCCGAGACTTCTCAACATGC 3220

AGJ-3_20 (*r7B*) TCCTGGCCATGACAGTCACCAATAGAGACCTGGACACTGTTCCGAGACTTCTCAACATGC 3220

AY198374.1 TCCTGGCCATGACAGTCACCAATAGAGACCTGGACACTGTTCCGAGACTTCTCAACATGC 3220

AGJ-2_20 (*r6A*) TCCTGGCCATGACAGTCACCAATAGAGACCTGGACACTGTTCCGAGACTTCTCAACATGC 2169

AGJ-2_10 (*r6A*) TCCTGGCCATGACAGTCACCAATAGAGACCTGGACACTGTTCCGAGACTTCTCAACATGC 2169

AGJ-2_15 (*r6A*) TCCTGGCCATGACAGTCACCAATAGAGACCTGGACACTGTTCCGAGACTTCTCAACATGC 2169

**********************************************:*************

AGJ-1_5 (*r5B*) TGTCGCCTAACAACGTAACCGGATTCCTCCAGACAGCAATGCCTTTGAGAGGATATTGGG 3277

AGJ-1_6 (*r5B*) TGTCGCCTAACAACGTAACCGGATTCCTCCAGACAGCAATGCCTTTGAGAGGATATTGGG 3277

AGJ-1_1 (*r5A*) TGTCGCCTAACAACGTAACCGGATTCCTCCAGACAGCAATGCCTTTGAGAGGATATTGGG 3280

AGJ-1_3 (*r5C*) TGTCGCCTAACAACGTAACCGGATTCCTCCAGACAGCAATGCCTTTGAGAGGATATTGGG 3299

AGJ-1_2 (*r5A*) TGTCGCCTAACAACGTTACCGGATTCCTCCAGACAGCAATGCCTTTGAGAGGATATTGGG 3280

AGJ-1_4 (*r5A*) TGTCGCCTAACAACGTAACCGAATTCCTCCAGACAGCAATGCCTTTGAGAGGATATTGGG 3280

AGJ-3_15 (*r7A*) TGTCGCCTAACAACGTAACCGGATTCCTTCAGACAGCAATGCCTTTGAGAGGATATTGGG 3280

AGJ-3_3 (*r7B*) TGTCGCCTAACAACGTAACCGGATTCCTTCAGACAGCAATGCCTTTGAGAGGATATTGGG 3280

AGJ-3_1 (*r7B*) TGTCGCCTAACAACGTAACCGGATTCCTTCAGACAGCAATGCCTTTGAGAGGATATTGGG 3280

AGJ-3_20 (*r7B*) TGTCGCCTAACAACGTAACCGGATTCCTTCAGACAGCAATGCCTTTGAGAGGATATTGGG 3280

AY198374.1 TGTCGCCTAACAACGTAACCGGATTCCTCCAGACAGCAATGCCTTTGAGAGGATATTGGG 3280

AGJ-2_20 (*r6A*) TGTCGCCTAACAACGTAACCGGATTCCTCCAGACAGCAATGCCTTTAAGAGGATATTGGG 2229

AGJ-2_10 (*r6A*) TGTCGCCTAACAACGTAACCGGATTCCTCCAGACAGCAATGCCTTTAAGAGGATATTGGG 2229

AGJ-2_15 (*r6A*) TGTCGCCTAACAACGTAACCGGATTCCTCCAGACAGCAATGCCTTTAAGAGGATATTGGG 2229

****************:****.****** *****************.*************

AGJ-1_5 (*r5B*) GGACTTACGATATAAGTGTACT-------------------------------------- 3299

AGJ-1_6 (*r5B*) GGACTTACGATATAAGTGTACT-------------------------------------- 3299

AGJ-1_1 (*r5A*) GGACTTACGATATAAGTGTACT-------------------------------------- 3302

AGJ-1_3 (*r5C*) GGACTTACGATATAAGTGTACT-------------------------------------- 3321

AGJ-1_2 (*r5A*) GGACTTACGATATAAGTGTACT-------------------------------------- 3302

AGJ-1_4 (*r5A*) GGACTTACGATATAAGTGTACT-------------------------------------- 3302

AGJ-3_15 (*r7A*) GTACTTACGATATAAGTATACT-------------------------------------- 3302

AGJ-3_3 (*r7B*) GTACTTACGATATAAGTATACTGGCGTTCGACCACGGTATTCCTCAGCAGATATCTCATG 3340

AGJ-3_1 (*r7B*) GTACTTACGATATAAGTATACTGGCGTTCGACCACGGTATTCCTCAGCAGATATCTCATG 3340

AGJ-3_20 (*r7B*) GTACTTACGATATAAGTATACTGGCGTTCGACCACGGTATTCCTCAGCAGATATCTCATG 3340

AY198374.1 GGACTTACGATATAAGTATACTGGCGTTCGACCACGGTATTCCTCAGCAGATATCTCATG 3340

AGJ-2_20 (*r6A*) GGACTTACGATATAAGTATACTGGCGTTCGACCACGGTATTCCTCAGCAGATATCTCATG 2289

AGJ-2_10 (*r6A*) GGACTTACGATATAAGTATACTGGCGTTCGACCACGGTATTCCTCAGCAGATATCTCATG 2289

AGJ-2_15 (*r6A*) GGACTTACGATATAAGTATACTGGCGTTCGACCACGGTATTCCTCAGCAGATATCTCATG 2289

* ***************.****

AGJ-1_5 (*r5B*) ------------------------------------------------------------ 3299

AGJ-1_6 (*r5B*) ------------------------------------------------------------ 3299

AGJ-1_1 (*r5A*) ------------------------------------------------------------ 3302

AGJ-1_3 (*r5C*) ------------------------------------------------------------ 3321

AGJ-1_2 (*r5A*) ------------------------------------------------------------ 3302

AGJ-1_4 (*r5A*) ------------------------------------------------------------ 3302

AGJ-3_15 (*r7A*) ------------------------------------------------------------ 3302

AGJ-3_3 (*r7B*) AGGTGTATGAATCGGAAATTCGACCTTACAATTACAATCCTCCCCAGTTCGTTTTTCCTG 3400

AGJ-3_1 (*r7B*) AGGTGTATGAATTGGAAATTCGACCTTACAATTACAATCCTCCCCAGTTCGTTTTTCCTG 3400

AGJ-3_20 (*r7B*) AGGTGTATGAATTGGAAATTCGACCTTACAATTACAATCCTCCCCAGTTCGTTTTTCCTG 3400

AY198374.1 AGGTGTATGAATTGGAAATTCGACCTTACAATTACAATCCTCCCCAGTTCGTTTTTCCTG 3400

AGJ-2_20 (*r6A*) AGGTGTATGAATTGGAAATTCGACCTTACAATTACAATCCTCCCCAGTTCGTTTTTCCTG 2349

AGJ-2_10 (*r6A*) AGGTGTATGAATTGGAAATTCGACCTTACAATTACAATCCTCCCCAGTTCGTTTTTCCTG 2349

AGJ-2_15 (*r6A*) AGGTGTATGAATTGGAAATTCGACCTTACAATTACAATCCTCCCCAGTTCGTTTTTCCTG 2349

AGJ-1_5 (*r5B*) ------------------------------------------------------------ 3299

AGJ-1_6 (*r5B*) ------------------------------------------------------------ 3299

AGJ-1_1 (*r5A*) ------------------------------------------------------------ 3302

AGJ-1_3 (*r5C*) ------------------------------------------------------------ 3321

AGJ-1_2 (*r5A*) ------------------------------------------------------------ 3302

AGJ-1_4 (*r5A*) ------------------------------------------------------------ 3302

AGJ-3_15 (*r7A*) ------------------------------------------------------------ 3302

AGJ-3_3 (*r7B*) AATCCGGGACGATTCTACGACTGGCTTTGGAACGCGCAGTGGTAAATAATGTTTTGTCAC 3460

AGJ-3_1 (*r7B*) AATCCGGGACGATTCTACGACTGGCTTTGGAACGCGCAGTGGTAAATAATGTTTTGTCAC 3460

AGJ-3_20 (*r7B*) AATCCGGGACGATTCTACGACTGGCTTTGGAACGCGCAGTGGTAAATAATGTTTTGTCAC 3460

AY198374.1 AATCCGGGACGATTCTACGACTGGCTTTGGAACGCGCAGTGGTAAATAATGTTTTGTCAC 3460

AGJ-2_20 (*r6A*) AATCCGGGACGATTCTACGACTGGCTTTGGAACGCGCAGTGGTAAATAATGTTTTGTCAC 2409

AGJ-2_10 (*r6A*) AATCCGGGACGATTCTACGACTGGCTTTGGAACGCGCAGTGGTAAATAATGTTTTGTCAC 2409

AGJ-2_15 (*r6A*) AATCCGGGACGATTCTACGACTGGCTTTGGAACGCGCAGTGGTAAATAATGTTTTGTCAC 2409

AGJ-1_5 (*r5B*) ------------------------------------------------------------ 3299

AGJ-1_6 (*r5B*) ------------------------------------------------------------ 3299

AGJ-1_1 (*r5A*) ------------------------------------------------------------ 3302

AGJ-1_3 (*r5C*) ------------------------------------------------------------ 3321

AGJ-1_2 (*r5A*) ------------------------------------------------------------ 3302

AGJ-1_4 (*r5A*) ------------------------------------------------------------ 3302

AGJ-3_15 (*r7A*) ------------------------------------------------------------ 3302

AGJ-3_3 (*r7B*) TTGTAAACGGTGACCCGTTAGACAGGATACAAGCAATTGACGACGATGGTCTTGATGCTG 3520

AGJ-3_1 (*r7B*) TTGTAAACGGTGACCCGTTAGACAGGATACAAGCAATTGACGACGATGGTCTTGATGCTG 3520

AGJ-3_20 (*r7B*) TTGTAAATGGTGACCCGTTAGACAGGATACAAGCAATTGACGACGATGGTCTTGATGCTG 3520

AY198374.1 TTGTAAACGGTGACCCGTTAGACAGGATACAAGCAATTGACGACGATGGTCTTGATGCTG 3520

AGJ-2_20 (*r6A*) TTGTACACGGTGACCCGTTAGACAGGATACAAGCAATTGACGACGATGGTCTTGATGCTG 2469

AGJ-2_10 (*r6A*) TTGTAAACGGTGACCCGTTAGACAGGATACAAGCAATTGACGACGATGGTCTTGATGCTG 2469

AGJ-2_15 (*r6A*) TTGTAAACGGTGACCCGTTAGACAGGATACAAGCAATTGACGACGATGGTCTTGATGCTG 2469

AGJ-1_5 (*r5B*) ------------------------------------------------------------ 3299

AGJ-1_6 (*r5B*) ------------------------------------------------------------ 3299

AGJ-1_1 (*r5A*) ------------------------------------------------------------ 3302

AGJ-1_3 (*r5C*) ------------------------------------------------------------ 3321

AGJ-1_2 (*r5A*) ------------------------------------------------------------ 3302

AGJ-1_4 (*r5A*) ------------------------------------------------------------ 3302

AGJ-3_15 (*r7A*) -----------------------------GCTGATGCATCAAACTACTTCAGAGTAAATA 3333

AGJ-3_3 (*r7B*) GCGTGGTGACTTTCGATATTGTTGGAGATGCTGATGCATCAAACTACTTCAGAGTAAATA 3580

AGJ-3_1 (*r7B*) GCGTGGTGACTTTCGATATTGTTGGAGATGCTGATGCATCAAACTACTTCAGAGTAAATA 3580

AGJ-3_20 (*r7B*) GCGTGGTGACCTTCGATATTGTTGGAGATGCTGATGCATCAAACTACTTCAGAGTAAATA 3580

AY198374.1 GCGTGGTGACTTTCGATATTGTTGGAGATGCTGATGCATCAAACTACTTCAGAGTAAATA 3580

AGJ-2_20 (*r6A*) GCGTGGTGACTTTCGATATTGTTGGAGATGCTGATGCATCAAACTACTTCAGAGTAAATA 2529

AGJ-2_10 (*r6A*) GCGTGGTGACTTTCGATATTGTTGGAGATGCTGATGCATCAAACTACTTCAGAGTAAATA 2529

AGJ-2_15 (*r6A*) GCGTGGTGACTTTCGATATTGTTGGAGATGCTGATGCATCAAACTACTTCAGAGTAAATA 2529

AGJ-1_5 (*r5B*) ------------------------------------------------------------ 3299

AGJ-1_6 (*r5B*) ------------------------------------------------------------ 3299

AGJ-1_1 (*r5A*) ------------------------------------------------------------ 3302

AGJ-1_3 (*r5C*) ------------------------------------------------------------ 3321

AGJ-1_2 (*r5A*) ------------------------------------------------------------ 3302

AGJ-1_4 (*r5A*) ------------------------------------------------------------ 3302

AGJ-3_15 (*r7A*) ATGATGGCGACAACTTTGGAACCTTGTTGCTGACACAGGCGCTTCCTGAGGAAGGCAAGG 3393

AGJ-3_3 (*r7B*) ATGATGGCGACAACTTTGGAACCTTGTTGCTGACACAGGCGCTTCCTGAGGAAGGCAAGG 3640

AGJ-3_1 (*r7B*) ATGATGGCGACAACTTTGGAACCTTGTTGCTGACACAGGCGCTTCCTGAGGAAGGCAAGG 3640

AGJ-3_20 (*r7B*) ATGATGGCGACAACTTTGGAACCTTGTTGCTGACACAGGCGCTTCCTGAGGAAGGCAAGG 3640

AY198374.1 ATGATGGCGACAACTTTGGAACCTTGTTGCTGACACAGGCGCTTCCTGAGGAAGGCAAGG 3640

AGJ-2_20 (*r6A*) ATGATGGCGACAACTTTGGAACCTTGTTGCTGACACAGGCGCTTCCTGAGGAAGGCAAGG 2589

AGJ-2_10 (*r6A*) ATGATGGCGACAACTTTGGAACCTTGTTGCTGACGCAGGCGCTTCCTGAGGAAGGCAAGG 2589

AGJ-2_15 (*r6A*) ATGATGGCGACAACTTTGGAACCTTGTTGCTGACACAGGCGCTTCCTGAGGAAGGCAAGG 2589

AGJ-1_5 (*r5B*) ------------------------------------------------------------ 3299

AGJ-1_6 (*r5B*) ------------------------------------------------------------ 3299

AGJ-1_1 (*r5A*) ------------------------------------------------------------ 3302

AGJ-1_3 (*r5C*) ------------------------------------------------------------ 3321

AGJ-1_2 (*r5A*) ------------------------------------------------------------ 3302

AGJ-1_4 (*r5A*) ------------------------------------------------------------ 3302

AGJ-3_15 (*r7A*) AATTTGAGGTTACCATCCGGGCTACAGACGGCGGAACAGAACCTCGATCATATTCAACAG 3453

AGJ-3_3 (*r7B*) AATTTGAGGTTAGCATCCGGGCTACAGACGGCGGAACAGAACCTCGATCATATTCAACAG 3700

AGJ-3_1 (*r7B*) AATTTGAGGTTACCATCCGGGCTACAGACGGCGGAACAGAACCTCGATCATATTCAACAG 3700

AGJ-3_20 (*r7B*) AATTTGAGGTTACCATCCGGGCTACAGACGGCGGAACAGAACCTCGATCATATTCAACAG 3700

AY198374.1 AATTTGAGGTTACCATCCGGGCTACAGACGGCGGAACAGAACCTCGATCATATTCAACAG 3700

AGJ-2_20 (*r6A*) AATTTGAGGTTACCATCCGGGCTACAGACGGCGGAACGGAACCTCGATCATATTCAACAG 2649

AGJ-2_10 (*r6A*) AATTTGAGGTTACCATCCGGGCTACAGACGGCGGAACGGAACCTCGATCATATTCAACAG 2649

AGJ-2_15 (*r6A*) AATTTGAGGTTACCATCCGGGCTACAGACGGCGGAACGGAACCTCGATCATATTCAACAG 2649

AGJ-1_5 (*r5B*) ------------------------------------------------------------ 3299

AGJ-1_6 (*r5B*) ------------------------------------------------------------ 3299

AGJ-1_1 (*r5A*) ------------------------------------------------------------ 3302

AGJ-1_3 (*r5C*) ------------------------------------------------------------ 3321

AGJ-1_2 (*r5A*) ------------------------------------------------------------ 3302

AGJ-1_4 (*r5A*) ------------------------------------------------------------ 3302

AGJ-3_15 (*r7A*) ACTCCACTATAACAGTGCTCTTCGTTCCGACTCTGGGTGATCCGATCTTTCAAGATAACA 3513

AGJ-3_3 (*r7B*) ACTCCACTATAACAGTGCTCTTCGTTCCGACTTTGGGTGATCCGATCTTTCAAGATAACA 3760

AGJ-3_1 (*r7B*) ACTCCACTATAACAGTGCTCTTCGTTCCGACTTTGGGTGATCCGATCTTTCAAGATAACA 3760

AGJ-3_20 (*r7B*) ACTCCACTATAACAGTGCTCTTCGTTCCGACTTTGGGTGATCCGATCTTTCAAGATAACA 3760

AY198374.1 ACTCCACTATAACAGTGCTCTTCGTTCCGACTTTGGGTGATCCGATCTTTCAAGATAACA 3760

AGJ-2_20 (*r6A*) ACTCCACTATAACAGTGCTCTTCGTTCCGACTTTGGGTGATCCGATCTTTCAAGATAACA 2709

AGJ-2_10 (*r6A*) ACTCCACTATAACAGTGCTCTTCGTTCCGACTTTGGGTGATCCGATCTTTCAAGATAACA 2709

AGJ-2_15 (*r6A*) ACTCCACTATAACAGTGCTCTTCGTTCCGACTTTGGGTGATCCGATCTTTCAAGATAACA 2709

AGJ-1_5 (*r5B*) --------------------GAAAAAGAGGTTGGCTTGACTGAGAGGTTCACGCTCCCAC 3339

AGJ-1_6 (*r5B*) --------------------GAAAAAGAGGTTGGCTTGACTGAGAGGTTCACGCTCCCAC 3339

AGJ-1_1 (*r5A*) --------------------GAAAAAGAGGTTGGCTTGACTGAGAGGTTCACGCTCCCAC 3342

AGJ-1_3 (*r5C*) --------------------GAAAAAGAGGTTGGCTTGACTGAGAGGTTCACGCTCCCAC 3361

AGJ-1_2 (*r5A*) --------------------GAAAAAGAGGTTGGCTTGACTGAGAGGTTCACGCTCCCAC 3342

AGJ-1_4 (*r5A*) --------------------GAAAAAGAGGTTGGCTTGACTGAGAGGTTCACGCTCCCAC 3342

AGJ-3_15 (*r7A*) CTTACTCAGTAGCATTCTTTGAAAAAGAGGTTGGCTTGACTGAGAGGTTCTCGCTCCCAC 3573

AGJ-3_3 (*r7B*) CTTACTCAGTAGCATTCTTTGAAAAAGAGGTTGGCTTGACTGAGAGGTTCTCGCTCCCAC 3820

AGJ-3_1 (*r7B*) CTTACTCAGTAGCATTCTTTGAAAAAGAGGTTGGCTTGACTGAGAGGTTCTCGCTCCCAC 3820

AGJ-3_20 (*r7B*) CTTACTCAGTAGCATTCTTTGAAAAAGAGGTTGGCTTGACTGAGAGGTTCTCGCTCCCAC 3820

AY198374.1 CTTACTCAGTAGCATTCTTTGAAAAAGAGGTTGGCTTGACTGAGAGGTTCTCGCTCCCAC 3820

AGJ-2_20 (*r6A*) CTTACTCAGTAGCATTCTTTGAAAAAGAGGTTGGCTTGACTGAGAGGTTCTCGCTCCCAC 2769

AGJ-2_10 (*r6A*) CTTACTCAGTAGCATTCTTTGAAAAAGAGGTTGGCTTGACTGAGAGGTTCTCGCTCCCAC 2769

AGJ-2_15 (*r6A*) CTTACTCAGTAGCATTCTTTGAAAAAGAGGTTGGCTTGACTGAGAGGTTCTCGCTCCCAC 2769

******************************:*********

AGJ-1_5 (*r5B*) TAGCAGAGGACCCTAAGAACAAACTTTGCACTGACGACTGTCACGATATTTACTACAGGA 3399

AGJ-1_6 (*r5B*) TAGCAGAGGACCCTAAGAACAAACTTTGCACTGACGACTGTCACGATATTTACTACAGGA 3399

AGJ-1_1 (*r5A*) TAGCAGAGGACCCTAAGAACAAACTTTGCACTGACGACTGTCACGATATTTACTACAGGA 3402

AGJ-1_3 (*r5C*) TAGCAGAGGACCCTAAGGACAAACTTTGCACTGACGACTGTCACGATATTTACTACAGGA 3421

AGJ-1_2 (*r5A*) TAGCAGAGGACTCTAAGAACAAACTTTGCACCGACGACTGTCACGATATTTACTACAGGA 3402

AGJ-1_4 (*r5A*) TAGCAGAGGACCCTAAGAACAAACTTTGCACTGACGACTGTCACGATATTTACTACAGGA 3402

AGJ-3_15 (*r7A*) ATGCAGAGGACCCTAAGAACAAACTCTGCACTGACGACTGTCACGATATTTACTACAGGA 3633

AGJ-3_3 (*r7B*) ATGCAGAGGACCCTAAGAACAAACTCTGCACTGACGACTGTCACGATATTTACTACAGGA 3880

AGJ-3_1 (*r7B*) ATGCAGAGGACCCTAAGAACAAACTCTGCACTGACGACTGTCACGATATTTACTACAGGA 3880

AGJ-3_20 (*r7B*) ATGCAGAGGACCCTAAGAACAAACTCTGCACTGACGACTGTCACGATATTTACTACAGGA 3880

AY198374.1 ATGCAGAGGACCCTAAGAACAAACTCTGCACTGACGACTGTCACGATATTTACTACAGGA 3880

AGJ-2_20 (*r6A*) ATGCAGAGGACCCTAAGAACAAACTCTGCACTGACGACTGTCACGATATTTACTACAGGA 2829

AGJ-2_10 (*r6A*) ATGCAGAGGACCCTAAGAACAAACTCTGCACTGACGACTGTCACGATATTTACTACAGGA 2829

AGJ-2_15 (*r6A*) ATGCAGAGGACCCTAAGAACAAACTCTGCACTGACGACTGTCACGATATTTACTACAGGA 2829

::********* *****.******* ***** ****************************

AGJ-1_5 (*r5B*) TCTTTGGTGGTGTGGATTACGAGCCATTTGACCTGGACCCGGTGACGAACGTGATCTTCC 3459

AGJ-1_6 (*r5B*) TCTTTGGTGGTGTGGATTACGAGCCATTTGACCTGGACCCGGTGACGAACGTGATCTTCC 3459

AGJ-1_1 (*r5A*) TCTTTGGTGGTGTGGATTACGAGCCATTTGACCTGGACCCGGTGACGAACGTGATCTTCC 3462

AGJ-1_3 (*r5C*) TCTTTGGTGGTGTGGATTACGAGCCATTTGACCTGGACCCGGTGACGAACGTGATCTTCC 3481

AGJ-1_2 (*r5A*) TCTTTGGTGGTGTGGATTACGAGCCATTTGACCTGGACCCGGTGACGAACGTGATCTTCC 3462

AGJ-1_4 (*r5A*) TCTTTGGTGGTGTGGATTACGAGCCATTTGACCTGGACCCGGTGACGAACGTGATCTTCC 3462

AGJ-3_15 (*r7A*) TCTTTGGTGGTGTGGATTACGAGCCATTTGACCTGGACCCGGTGACGAACGTGATCTTCC 3693

AGJ-3_3 (*r7B*) TCTTTGGTGGTGTGGATTACGAGCCATTTGACCTGGACCCGGCGACGAACGTGATCTTCC 3940

AGJ-3_1 (*r7B*) TCTTTGGTGGTGTGGATTACGAGCCATTTGACCTGGGCCCGGTGACGAACGTGATCTTCC 3940

AGJ-3_20 (*r7B*) TCTTTGGTGGTGTGGATTACGAGCCATTTGACCTGGACCCGGTGACGAACGTGATCTTCC 3940

AY198374.1 TCTTTGGTGGTGTGGATTACGAGCCATTTGACCTGGACCCGGTGACGAACGTGATCTTCC 3940

AGJ-2_20 (*r6A*) TCTTTGGTGGTGTGGATTACGAGCCATTTGACCTGGACCCGGTGACGAACGTGATCTTCC 2889

AGJ-2_10 (*r6A*) TCTTTGGTGGTGTGGATTACGAGCCATTTGACCTGGACCCGGTGACGAACGTGATCTTCC 2889

AGJ-2_15 (*r6A*) TCTTTGGTGGTGTGGATTACGAGCCATTTGACCTGGACCCGGTGACGAACGTGATCTTCC 2889

************************************.***** *****************

AGJ-1_5 (*r5B*) TGAAATCAGAACTAGACCGGGATACCACTGCCACGCATGTGGTGCAAGTGGCAGCCAGTA 3519

AGJ-1_6 (*r5B*) TGAAATCAGAACTAGACCGGGATACCACTGCCACGCATGTGGTGCAAGTGGCAGCCAGTA 3519

AGJ-1_1 (*r5A*) TGAAATCAGAACTAGACCGGGATACCACTGCCACGCATGTGGTGCAAGTGGCAGCCAGTA 3522

AGJ-1_3 (*r5C*) TGAAATCAGAACTAGACCGGGATACCACTGCCACGCATGTGGTGCAAGTGGCAGCCAGTA 3541

AGJ-1_2 (*r5A*) TGAAATCAGAACTAGACCGGGATACCACTGCCACGCATGTGGTGCAAGTGGCAGCCAGTA 3522

AGJ-1_4 (*r5A*) TGAAATCAGAACTAGACCGGGATACCACTGCCACGCATGTGGTGCAAGTGGCAGCCAGTA 3522

AGJ-3_15 (*r7A*) TGAAATCAGAACTAGACCGGGAGACCACTGCTACGCATGTGGTGCAAGTGGCAGCCAGTA 3753

AGJ-3_3 (*r7B*) TGAAATCAGAACTAGACCGGGAGACCACTGCTACGCATGTGGTGCAGGTGGCAGCCAGTA 4000

AGJ-3_1 (*r7B*) TGAAATCAGAACTAGACCGGGAGACCACTGCTACGCATGTGGTGCAAGTGGCAGCCAGTA 4000

AGJ-3_20 (*r7B*) TGAAATCAGAACTAGACCGGGAGACCACTGCTACGCATGTGGTGCAAGTGGCAGCCAGTA 4000

AY198374.1 TGAAATCAGAACTAGACCGGGAGACCACTGCTACGCATGTGGTGCAAGTGGCAGCCAGTA 4000

AGJ-2_20 (*r6A*) TGAAATCAGAACTAGACCGGGAGACCACTGCCACGCATGTGGTGCAAGTGGCAGCCAGTA 2949

AGJ-2_10 (*r6A*) TGAAATCAGAACTAGACCGGGAGACCACTGCCACGCATGTGGTGCAAGTGGCAGCCAGTG 2949

AGJ-2_15 (*r6A*) TGAAATCAGAACTTGACCGGGAGACCACTGCCACGCATGTGGTGCAAGTGGCAGCCAGTA 2949

*************:******** ******** **************.************.

AGJ-1_5 (*r5B*) ATTCGCCCACAGGAGGCGGAATACCACTCCCTGGGTCTCTTCTCACCGTCACTGTCACTG 3579

AGJ-1_6 (*r5B*) ATTCGCCCACAGTAGGCGGAATACCACTCCCTGGGTCTCTTCTCACCGTCACTGTCACTG 3579

AGJ-1_1 (*r5A*) ATTCGCCCACAGGAGGCGGAATACCACTCCCTGGGTCTCTTCTCACCGTCACTGTCACTG 3582

AGJ-1_3 (*r5C*) ATTCGCCCACAGGAGGCGGAATACCACTCCCTGGGTCTCTTCTCACCGTCACTGTCACTG 3601

AGJ-1_2 (*r5A*) ATTCGCCCACAGGAGGCGGAATACCACTCCCTGGGTCTCTTCTCACCGTCACTGTCACTG 3582

AGJ-1_4 (*r5A*) ATTCGCCCACAGGAGGCGGAATACCACTCCCTGGGTCTCTTCTCACCGTCACTGTCACTG 3582

AGJ-3_15 (*r7A*) ATTCGCCCACAGGAGGCGGAATACCACTCCCTGGGTCTCTTCTCACCGTCACTGTCACTG 3813

AGJ-3_3 (*r7B*) ATTCGCCCACAGGAGGCGGAATACCACTCCCTGGGTCTCTTCTCACCGTCACTGTCACT- 4059

AGJ-3_1 (*r7B*) ATTCGCCCACAGGAGGCGGAATACCACTCCCTGGGTCTCTTCTCACCGTCACTGACACT- 4059

AGJ-3_20 (*r7B*) ATTCGCCCACAGGAGGCGGAATACCACTCCCTGGGTCTCTTCTCACCGTCACTGTCACT- 4059

AY198374.1 ATTCGCCCACAGGAGGCGGAATACCACTCCCTGGGTCTCTTCTCACCGTCACTGTCACTG 4060

AGJ-2_20 (*r6A*) ATTCGCCCACAGGAGGCGGAATACCACTCCCTGGGTCTCTTCTCACCGTCACTGTCACTG 3009

AGJ-2_10 (*r6A*) ATTCGCCCACAGGAGGCGGAATACCACTCCCTGGGTCTCTTCTCACCGCCACTGTCACTG 3009

AGJ-2_15 (*r6A*) ATTCGCCCACAGGAGGCGGAATACCACTCCCTGGGTCTCTTCTCACCGTCACTGTCACTG 3009

************ *********************************** *****:****

AGJ-1_5 (*r5B*) TACGAGAAGCGGATCCACGGCCTGTGTTCGAGCAGCGTCTGTACACGGCTGGCATTTCCA 3639

AGJ-1_6 (*r5B*) TACGAGAAGCGGATCCACGGCCTGTGTTCGAGCAGCGTCTGTACACGGCTGGCATTTCCA 3639

AGJ-1_1 (*r5A*) TACGAGAAGCGGATCCACGGCCTGTGTTCGAGCAGCGTCTGTACACGGCTGGCATTTCCA 3642

AGJ-1_3 (*r5C*) TACGAGAAGCGGATCCACGGCCTGTGTTCGAGCAGCGTCTGTACACGGCTGGCATTTCCA 3661

AGJ-1_2 (*r5A*) TACGAGAAGCGGATCCACAGCCTGTGTTCGAGCAGCGTCTGTACACGGCTGGCATTTCCA 3642

AGJ-1_4 (*r5A*) TACGAGAAGCGGATCCACGGCCTGTGTTCGAGCAGCGTCTGTACACGGCTGGCATTTCCA 3642

AGJ-3_15 (*r7A*) TACGAGAAGCGGATCCACGGCCTGTGTTCGAGCAGCGTCTGTACACGGCTGGCATTTCCA 3873

AGJ-3_3 (*r7B*) ------------------------------------------------------------ 4059

AGJ-3_1 (*r7B*) ------------------------------------------------------------ 4059

AGJ-3_20 (*r7B*) ------------------------------------------------------------ 4059

AY198374.1 TACGAGAAGCGGATCCACGGCCTGTGTTCGAGCAGCGTCTGTACACGGCTGGCATTTCCA 4120

AGJ-2_20 (*r6A*) TACGAGAAGCGGATCCACGGCCTGTGTTCGAGCAGCGTCTGTACACGGCTGGCATTTCCA 3069

AGJ-2_10 (*r6A*) TACGAGAAGCGGATCCACGGCCTGTGTTCGAGCAGCGTCTGTACACGGCTGGCATTTCCA 3069

AGJ-2_15 (*r6A*) TACGAGAAGCGGATCCACGGCCTGTGTTCGAGCAGCGTCTGTACACGGCTGGCATTTCCA 3069

AGJ-1_5 (*r5B*) CTTCCGATAACATCAACAGGGAACTACCCACCGTTCGTGCAACTCATTCCGAAAACGCAC 3699

AGJ-1_6 (*r5B*) CTTCCGATAACATCAACAGGGAACTACTCACCGTTCGTGCAACTCATTCCGAAAACGCAC 3699

AGJ-1_1 (*r5A*) CTTCCGATAACATCAACAGGGAACTACTCACCGTTCGTGCAACTCATTCCGAAAACGCAC 3702

AGJ-1_3 (*r5C*) CTTCCGATAACATCAACAGGGAACTACTCACCGTTCGTGCAACTCATTCCGAAAACGCAC 3721

AGJ-1_2 (*r5A*) CTTCCGATAACATCAACAGGGAACTACTCACCGTTCGTGCAACTCATTCCGAAAACGCAC 3702

AGJ-1_4 (*r5A*) CTTCCGATAACATCAACAGGGAACTACTCACCGTTCGTGCAACTCATTCCGAAAACGCAC 3702

AGJ-3_15 (*r7A*) CTTCCGATAACATCAACAGGGAACTACTCACCGTTCGTGCAACTCACTCCGAAAACGCAC 3933

AGJ-3_3 (*r7B*) --------------------------------------GCAACTCATTCCGAAAACGCAC 4081

AGJ-3_1 (*r7B*) --------------------------------------GCAACTCATTCCGAAAACGCAC 4081

AGJ-3_20 (*r7B*) --------------------------------------GCAACTCATTCCGAAAACGCAC 4081

AY198374.1 CTTCCGATAACATCAACAGGGAACTACTCACCGTTCGTGCAACTCATTCCGAAAACGCAC 4180

AGJ-2_20 (*r6A*) CTTCCGATAACATCAACAGGGAACTACTCACCGTTCGTGCAACTCATTCCGAAAACGCAC 3129

AGJ-2_10 (*r6A*) CTTCCGATAACATCAACAGGGAACTACTCACCGTTCGTGCAACTCATTCCGAAAACGCAC 3129

AGJ-2_15 (*r6A*) CTTCCGATAACATCAACAGGGAACTACTCACCGTTCGTGCAACTCGTTCCGAAAACGCAC 3129

*******. *************

AGJ-1_5 (*r5B*) AATTGACATATACCATCGAAGACGGTTCTATGGTGGTGGACTCCGCTCTGGAAGCCGTCA 3759

AGJ-1_6 (*r5B*) AATTGACATATACCATCGAAGACGGTTCTATGGTGGTGGACTCCACTCTGGAAGCCGTCA 3759

AGJ-1_1 (*r5A*) AATTGACATATACCATCGAAGACGGTTCTATGGTGGTGGACTCCACTCTGGAAGCCGTCA 3762

AGJ-1_3 (*r5C*) AATTGACATATACCATCGAAGACGGTTCTATGGTGGTGGACTCCACTCTGGAAGCCGTCA 3781

AGJ-1_2 (*r5A*) AATTGACATATACCATCGAAGACGGTTCTATGGTGGTGGACTCCACTCTGGAAGCCGTCA 3762

AGJ-1_4 (*r5A*) AATTGACATATACCATCGAAGACGGTTCTATGGTGGTGGACTCCACTCTGGAAGCCGTCA 3762

AGJ-3_15 (*r7A*) AATTGACATATACCATCGAAGACGGTTCTATGGCGGTGGTCTCCACTCTGGAAGCCGTCA 3993

AGJ-3_3 (*r7B*) AATTGACATATACCATCGAAGACGGTTCTATGGCGGTGGACTCCACTCTGGAAGCCGTCA 4141

AGJ-3_1 (*r7B*) AATTGACATATACCATCGAAGACGGTTCTATGGCGGTGGACTCCACTCTGGAAGCCGTCA 4141

AGJ-3_20 (*r7B*) AATTGACATATACCATCGAAGACGGTTCTATGGCGGTGGACTCCACTCTGGAAGCCGTCA 4141

AY198374.1 AATTGACATATACCATCGAAGACGGTTCTATGGCGGTGGACTCCACTCTGGAAGCCGTCA 4240

AGJ-2_20 (*r6A*) AATTGACGTATACCATCGAAGACGGTTCTATGGCGGTGGACTCCACTCTGGAAGCCGTCA 3189

AGJ-2_10 (*r6A*) AATTGACGTATACCATCGAAGACGGTTCTATGGCGGTGGACTCCACTCTAGAAGCCGTCA 3189

AGJ-2_15 (*r6A*) AATTGACATATACCATCGAAGACGGTTCTATGGCGGTGGACTCCACTCTGGAAGCCGTCA 3189

*******.************************* *****:****.****.**********

AGJ-1_5 (*r5B*) AGGACACGGCGTTCCATCTGAACGCGCAGACCGGCGTCCTCATACTGAGGATACAACCTA 3819

AGJ-1_6 (*r5B*) AGGACTCGGCGTTCCATCTGAACGCGCAGACCGGCGTCCTCATACTGAGGATACAACCTA 3819

AGJ-1_1 (*r5A*) AGGACTCGGCGTTCCATCTGAACGCGCAGACCGGCGTCCTCATACTGAGGATACAACCTA 3822

AGJ-1_3 (*r5C*) AGGACTCGGCGTTCCATCTGAGCGCGCAGACCGGCGTCCTCATACTGAGGATACAACCTA 3841

AGJ-1_2 (*r5A*) AGGACTCGGCGTTCCATCTGAACGCGCAGACCGGCGTCCTCATACTGAGGATACAACCTA 3822

AGJ-1_4 (*r5A*) AGGACTCGGCGTTCCATCTGAACGCGCAGACCGGCGTCCTCATACTGAGGATACAACCTA 3822

AGJ-3_15 (*r7A*) AGGACTCGGCGTTCCATCTGAACGCGCAGACCGGCGTCCTCATACTGAGGATACAACCTA 4053

AGJ-3_3 (*r7B*) AGGACTCGGCGTTCCATCTGAACGCGCAGACCGGCGTCCTCATACTGAGGATACAACCTA 4201

AGJ-3_1 (*r7B*) AGGACTCGGCGTTCCATCTGAACGCGCAGACCGGCGTCCTCATACTGAGGATACAACCTA 4201

AGJ-3_20 (*r7B*) AGGACTCGGCGTTCCATCTGAACGCGCAGACCGGCGTCCTCATACTGAGGATACAACCTA 4201

AY198374.1 AGGACTCGGCGTTCCATCTGAACGCGCAGACCGGCGTCCTCATACTGAGGATACAACCTA 4300

AGJ-2_20 (*r6A*) AGGACTCGGCGTTCCATCTGAACGCGCAGACCGGCGTCCTCATACTGAGGATACAACCTA 3249

AGJ-2_10 (*r6A*) AGGACTCGGCGCTCCATCTGAACGCGCAGACCGGCGTCCTCATACTGAGGATACAACCTA 3249

AGJ-2_15 (*r6A*) AGGACTCGGCGTTCCATCTGAACGCGCAGACCGGCGTCCTCATACTGAGGATACAACCTA 3249

*****:***** *********.**************************************

AGJ-1_5 (*r5B*) CTGCCAGCATGCAGGGCATGTTCGAGTTCAACGTCATCGCTACTGACCCAGATGAGAAGA 3879

AGJ-1_6 (*r5B*) CTGCCAGCATGCAGGGCATGTTCGAGTTCAACGTCATCGCTACTGACCCAGATGAGAAGA 3879

AGJ-1_1 (*r5A*) CTGCCAGCATGCAGGGCATGTTCGAGTTCAACGTCATCGCTACTGACCCAGATGAGAAGA 3882

AGJ-1_3 (*r5C*) CTGCCAGCATGCAGGGCATGTTCGAGTTCAACGTCATCGCTACTGACCCAGATGAGAAGA 3901

AGJ-1_2 (*r5A*) CTGCCAGCATGCAGGGCATGTTCGAGTTCAACGTCATCGCTACTGACCCAGATGAGAAGA 3882

AGJ-1_4 (*r5A*) CTGCCAGCATGCAGGGCATGTTCGAGTTCAACGTCATCGCTACTGACCCAGATGAGAAGA 3882

AGJ-3_15 (*r7A*) CTGCCAGCATGCAGGGCATGTTCGAGTTCAACGTCATCGCTACTGATCCAGATGAGAAGA 4113

AGJ-3_3 (*r7B*) CTGCCAGCATGCAGGGCATGTTCGAGTTCAACGTCATCGCTACTGATCCAGATGAGAAGA 4261

AGJ-3_1 (*r7B*) CTGCCAGCATGCAGGGCATGTTCGAGTTCAGCGTCATCGCTACTGATCCAGATGAGAAGA 4261

AGJ-3_20 (*r7B*) CTGCCAGCATGCAGGGCATGTTCGAGTTCAACGTCATCGCTACTGATCCAGATGAGAAGA 4261

AY198374.1 CTGCCAGCATGCAGGGCATGTTCGAGTTCAACGTCATCGCTACTGATCCAGATGAGAAGA 4360

AGJ-2_20 (*r6A*) CTGCCAGCATGCAGGGCATGTTCGAGTTCAACGTCATCGCTACTGATCCAGATGAGAAGA 3309

AGJ-2_10 (*r6A*) CTGCCAGCATGCAGGGCATGTTCGAGTTCAACGTCATCGCTACTGATCCAGATGAGAAGA 3309

AGJ-2_15 (*r6A*) CTGCCAGCATGCAGGGCATGCTCGAGTTCAACGTCATCGCTACTGATCCAGATGAGAAGA 3309

******************** *********.*************** *************

AGJ-1_5 (*r5B*) CAGATACGGCAGAGGTGAAAGTCTACCTCATTTCATCCCAAAATAGGGTGTCCTTCATAT 3939

AGJ-1_6 (*r5B*) CAGATACGGCAGAGGTGAAAGTCTACCTCATTTCATCCCAAAATAGGGTGTCCTTCATAT 3939

AGJ-1_1 (*r5A*) CAGATACGGCAGAGGTGAAAGTCTACCTCATTTCATCCCAAAATAGGGTGTCCTTCATAT 3942

AGJ-1_3 (*r5C*) CAGATACGGCAGAGGTGAAAGTCTACCTCATTTCATCCCAAAATAGGGTGTCCTTCATAT 3961

AGJ-1_2 (*r5A*) CAGATACGGCAGAGGTGAAAGTCTACCTCATTTCATCCCAAAATAGGGTGTCCGTCATAT 3942

AGJ-1_4 (*r5A*) CAGATACGGCAGAGGTGAAAGTCTACCTCATTTCATCCCAAAATAGGGTGTCCTTCATAT 3942

AGJ-3_15 (*r7A*) CAGATACGGCAGAGGTGAAAGTCTACCTCATTTCATCCCAAAATAGGGTGTCCTTCATAT 4173

AGJ-3_3 (*r7B*) CAGATACGGCAGAGGTGAAAGTCTACCTCATTTCATCCCAAAATAGGGTGTCCTTCATAT 4321

AGJ-3_1 (*r7B*) CAGATACGGCAGAGGTGAAAGTCTACCTCATCTCATCCCAAAATAGGGTGTCCTTCATAT 4321

AGJ-3_20 (*r7B*) CAGATACGGCAGAGGTGAAAGTCTACCTCATTTCATCCCAAAATAGGGTGTCCTTCATAT 4321

AY198374.1 CAGATACGGCAGAGGTGAAAGTCTACCTCATTTCATCCCAAAATAGGGTGTCCTTCATAT 4420

AGJ-2_20 (*r6A*) CAGATACGGCAGAGGTGAAAGTCTACCCCATTTCATCCCAAAATAGGGTGTCCTTCATAT 3369

AGJ-2_10 (*r6A*) CAGATACGGCAGAGGTGAAAGTCTACCTCATTTCATCCCAAAATAGGGTGTCCTTCATAT 3369

AGJ-2_15 (*r6A*) CAGATACGGCAGAGGTGAAAGTCTACCTCATTTCATCCCAAAATAGGGTGTCCTTCATAT 3369

*************************** *** ********************* ******

AGJ-1_5 (*r5B*) TCCTGAACGATGTGGAGACGGTTGAGAGTAACAGAGACTTTATCGCAGAAACGTTCAGCG 3999

AGJ-1_6 (*r5B*) TCCTGAACGATGTGGAGACGGTTGAGAGTAACAGAGACTTTATCGCAGAAACGTACAGCG 3999

AGJ-1_1 (*r5A*) TCCTGAACGATGTGGGGACGGTTGAGAGTAACAGAGACTTTATCGCAGAAACGTTCAGCG 4002

AGJ-1_3 (*r5C*) TCCTGAACGATGTGGAGACGGTTGAGAGTAACAGAGACTTTATCGCAGAAACGTTCAGCG 4021

AGJ-1_2 (*r5A*) TCCTGAACGATGTGGAGACGGTTGAGAGTAACAGAGACTTTATCGCAGAAACGTTCAGCG 4002

AGJ-1_4 (*r5A*) TCCTGAACGATGTGGAGACGGTTGAGAGTAACAGAGACTTTATCGCAGAAACGTACAGCG 4002

AGJ-3_15 (*r7A*) TCCTGAACGATGTGGAGACGGTTGAGAGTAACAGAGACTTTATCGCAGAAACGTTCAGCG 4233

AGJ-3_3 (*r7B*) TCCTGAACGATGTGGAGACGGTTGAGAGTAACAGAGACTTTATCGCAGAAACGTTCAGCG 4381

AGJ-3_1 (*r7B*) TCCTGAACGATGTGGAGACGGTTGAGAGTAACAGAGACTTTATCGCAGAAACGTTCAGCG 4381

AGJ-3_20 (*r7B*) TCCTGAACGATGTGGAGACGGTTGAGAGTAACAGAGACTTTATCGCAGAAACGTTCAGCG 4381

AY198374.1 TCCTGAACGATGTGGAGACGGTTGAGAGTAACAGAGACTTTATCGCAGAAACGTTCAGCG 4480

AGJ-2_20 (*r6A*) TCCTGAACGATGTGGAGACGGTTGAGAGTAACAGAGACTTTATCGCAGAAACGTTCAGCG 3429

AGJ-2_10 (*r6A*) TCCTGAACGATGTGGAGACGGTTGAGAGTAACAGAGACTTTATCGCAGAAACGTTCAGCG 3429

AGJ-2_15 (*r6A*) TCCTGAACGATGTGGAGACGGTTGAGAGTAACAGAGACTTTATCGCAGAAACGTTCAGCG 3429

***************.**************************************:*****

AGJ-1_5 (*r5B*) TTGGCTTCAACATGACCTGCAATATAGATCAGGTGCTGCCGGGCACCAACGACGCCGGGG 4059

AGJ-1_6 (*r5B*) TTGGCTTCAACATGACCTGCGATATAGATCAGGTGCTGCCGGGCACCAACGACGCCGGGG 4059

AGJ-1_1 (*r5A*) TTGGCTTCAACATGACCTGCAATATAGATCAGGTGCTGCCGGGCACCGACGACGCCGGGG 4062

AGJ-1_3 (*r5C*) TTGGCTTCAACATGACCTGCAATATAGATCAGGTGCTGCCGGGCACCAACGACGCCGGGG 4081

AGJ-1_2 (*r5A*) TTGGCTTCAACATGACCTGCAATATAGATCAGGTGCTGCCGGGCACCAACGACGCCGGGG 4062

AGJ-1_4 (*r5A*) TTGGCTTCAACATGACCTGCAATATAGATCAGGTGCTGCCGGGCACCAACGACGCCGGGG 4062

AGJ-3_15 (*r7A*) TTGGCTTCAACATGACCTGCAATATAGATCAGGTGCTGCCGGGCACCAACGACGCCGGGG 4293

AGJ-3_3 (*r7B*) TTGGCTTCAACATGACCTGCAATATAGATCAGGTGCTGCCGGGCACCAACGACGCCGGGG 4441

AGJ-3_1 (*r7B*) TTGGCTTCAACATGACCTGCAATATAGATCAGGTGCTGCCGGGCACCAACGACGCCGGGG 4441

AGJ-3_20 (*r7B*) TTGGCTTCAACATGACCTGCAATATAGATCAGGTGCTGCCGGGCACCAACGACGCCGGGG 4441

AY198374.1 TTGGCTTCAACATGACCTGCAATATAGATCAGGTGCTGCCGGGCACCAACGACGCCGGGG 4540

AGJ-2_20 (*r6A*) TTGGCTTCAACATGACCTGCAATATAGATCAGGTGCTGCCGGGCACCAACGACGCCGGGG 3489

AGJ-2_10 (*r6A*) TTGGCTTCAACATGACCTGCAATATAGATCAGGTGCTGCCGGGCACCAACGACGCCGGGG 3489

AGJ-2_15 (*r6A*) TTGGCTTCAACATGACCTGCAATATAGATCAGGTGCTGCCGGGCACCAACGACGCCGGGG 3489

********************.**************************.************

AGJ-1_5 (*r5B*) TGATTCAGGAGGCCATGGCAGAAGTCCATGCTCACTTCATACAGGATAACATCCCTGTGA 4119

AGJ-1_6 (*r5B*) TGATTCAGGAGGCCATGGCAGAAGTCCATGCTCACTTCATACAGGATAACATCCCTGTGA 4119

AGJ-1_1 (*r5A*) TGATTCAGGAGGCCATGGCAGAAGTCCATGCTCACTTCATACAGGATAACATCCCTGTGA 4122

AGJ-1_3 (*r5C*) TGATTCAGGAGGCCATGGCAGAAGTCCATGCTCACTTCATACAGGATAACATCCCTGTGA 4141

AGJ-1_2 (*r5A*) TGATTCAGGAGGCCATGGCAGAAGTCCATGCTCACTTCATACAGGATAACATCCCTGTGA 4122

AGJ-1_4 (*r5A*) TGATTCAGGAGGCCATGGCAGAAGTCCATGCTCACTTCATACAGGATAACATCCCTGTGA 4122

AGJ-3_15 (*r7A*) TGATTCAGGAGGCCATGGCGGAAGTCCATGCTCACTTCATACAGGATAACATCCCTGTGA 4353

AGJ-3_3 (*r7B*) TGATTCAGGAGGCCATGGCGGAAGTCCATGCTCACTTCATACAGGATAACATCCCTGTGA 4501

AGJ-3_1 (*r7B*) TGATTCAGGAGGCCATGGCGGAAGTCCATGCTCACTTCATACAGGATAACATCCCTGTGA 4501

AGJ-3_20 (*r7B*) TGATTCAGGAGGCCATGGCGGAAGTCCATGCTCACTTCATACAGGATAACATCCCTGTGA 4501

AY198374.1 TGATTCAGGAGGCCATGGCGGAAGTCCATGCTCACTTCATACAGGATAACATCCCTGTGA 4600

AGJ-2_20 (*r6A*) TGATTCAGGAGGCCATGGCGGAAGTCCATGCTCACTTCATACAGGATAACATCCCTGTGA 3549

AGJ-2_10 (*r6A*) TGATTCAGGAGGCCATGGCGGAAGTCCATGCTCACTTCATACAGGATAACATCCCTGTGA 3549

AGJ-2_15 (*r6A*) TGATTCAGGAGGCCATGGCGGAAGTCCATGCTCACTTCATACAGGATAACATCCCTGTGA 3549

*******************.****************************************

AGJ-1_5 (*r5B*) GCGCCGACAGTATTGAAGAGCTTCGCAGTGACACTCAGCTGCTGCGCTCCGTCCAAGGTG 4179

AGJ-1_6 (*r5B*) GCGCCGACAGTATTGAAGAGCTTCGCAGTGACACTCAGCTGCTGCGCTCCGTCCAAGGTG 4179

AGJ-1_1 (*r5A*) GCGCCGACAGTATTGAAGAGCTTCGCGGTGACACTCAGCTGCTGCGCTCCGTCCAAGGTG 4182

AGJ-1_3 (*r5C*) GCGCCGACAGTATTGAAGAGCTTCGCAGTGACACTCAGCTGCTGCGCTCCGTCCAAGGTG 4201

AGJ-1_2 (*r5A*) GCGCCGACAGTATTGAAGAGCTTCGCAGTGACACTCAGCTGCTGCGCTCCGTCCAAGGTG 4182

AGJ-1_4 (*r5A*) GCGCCGACAGTATTGAAGAGCTTCGCAGTGACACTCAGCTGCTGCGCTCCGTCCAAGGTG 4182

AGJ-3_15 (*r7A*) GCGCCGACAGTATTGAAGAGCTTCGCAGTGACACTCAGCTGCTGCGCTCCGTCCAAGGTG 4413

AGJ-3_3 (*r7B*) GCGCCGACAGTATTGAAGAGCTTCGCAGTGACACTCAGCTGCTGCGCTCCGTCCAAGGTG 4561

AGJ-3_1 (*r7B*) GCGCCGACAGTATTGAAGAGCTTCGCAGTGACACTCAGCTGCTGCGCTCCGTCCAAGGTG 4561

AGJ-3_20 (*r7B*) GCGCCGACAGTATTGAAGAGCTTCGCAGTGACACTCAGCTGCTGCGCTCCGTCCAAGGTG 4561

AY198374.1 GCGCCGACAGTATTGAAGAGCTTCGCAGTGACACTCAGCTGCTGCGCTCCGTCCAAGGTG 4660

AGJ-2_20 (*r6A*) GCGCCGACAGTATTGAAGAGCTTCGCAGTGACACTCAGCTGCTGCGCTCCGTCCAAGGTG 3609

AGJ-2_10 (*r6A*) GCGCCGACAGTATTGAAGAGCTTCGCAGTGACACTCAGCTGCTGCGCTCCGTCCAAGGTG 3609

AGJ-2_15 (*r6A*) GCGCCGACAGTATTGAAGAGCTTCGCAGTGACACTCAGCTGCTGCGCTCCGCCCAAGGTG 3609

**************************.************************ ********

AGJ-1_5 (*r5B*) TGTTGAATCAACGGCTACTGGTCCTGAACGACCTGGTGACCGGGGTCAGCCCTGATCTCG 4239

AGJ-1_6 (*r5B*) TGTTGAATCAACGGCTACTGGTCCTGAACGACCTGGTGACCGGGGTCAGCCCTGATCTCG 4239

AGJ-1_1 (*r5A*) TGTTGAATCAACGGCTACTGGTCCTGAACGACCTGGTGACCGGGGTCAGCCCTGATCTCG 4242

AGJ-1_3 (*r5C*) TGTTGAATCAGCGGCTACTGGTCCTGAACGACCTGGTGACCGGGGTCAGCCCTGATCTCG 4261

AGJ-1_2 (*r5A*) TGTTGAATCAACGGCTACTGGTCCTGAACGACCTGGTGACCGGGGTCAGCCCTGATCTCG 4242

AGJ-1_4 (*r5A*) TGTTGAATCAACGGCTACTGGTCCTGAACGACCTGGTGACCGGGATCAGCCCTGATCTCA 4242

AGJ-3_15 (*r7A*) TGTTGAACCAACGGCTGTTGGTCCTGAACGACCTGGTGACGGGGGTCAGCCCTGATCTCG 4473

AGJ-3_3 (*r7B*) TGTTGAACCAACGGCTGTTGGTCCTGAACGACCTGGTGACGGGGGTCAGCCCTGATCTCG 4621

AGJ-3_1 (*r7B*) TGTTGAACCAACGGCTGTTGGTCCTGAACGACCTGGTGACGGGGGTCAGCCCTGATCTCG 4621

AGJ-3_20 (*r7B*) TGTTGAACCAACGGCTGTTGGTCCTGAACGACCTGGTGACGGGGGTCAGCCCTGATCTCG 4621

AY198374.1 TGTTGAACCAACGGCTGTTGGTCCTGAACGACCTGGTGACGGGGGTCAGCCCTGATCTCG 4720

AGJ-2_20 (*r6A*) TGTTGAACCAACGGCTGTTGGTCCTGAACGACCTGGTGACGGGGGTCAGCCCTGATCTCG 3669

AGJ-2_10 (*r6A*) TGTTGAACCAACGGCTGTTGGTCCTGAACGACCTGGTGACGGGGGTCAGCCCTGATCTCG 3669

AGJ-2_15 (*r6A*) TGTTGAACCAACGGCTGTTGGTCCTGAACGACCTGGTGACGGGGGTCAGCCCTGATCTCG 3669

******* **.*****. ********************** ***.**************.

AGJ-1_5 (*r5B*) GCACTGCCGGCGTGCAGATCACCATCTATGTGCTAGCCGGGTTGTCAGCCATCCTTGCCT 4299

AGJ-1_6 (*r5B*) GCACTGCCGGCGTGCAGATCACCATCTATGTGCTAGCCGGGTTGTCAGCCATCCTTGCCT 4299

AGJ-1_1 (*r5A*) GCACTGCCGGCGTGCAGATCACCATCTATGTGCTAGCCGGGTTGTCAGCCATCCTTGCCT 4302

AGJ-1_3 (*r5C*) GCACTGCCGGCGTGCAGATCACCATCTATGTGCTAGCCGGGTTGTCAGCCATCCTTGCCT 4321

AGJ-1_2 (*r5A*) GCACTGCCGGCGTGCAGATCACCATCTATGTGCTAGCCGGGTTGTCAGCCATCCTTGCCT 4302

AGJ-1_4 (*r5A*) GCACTGCCGGCGTGCAGATCACCATCTATGTGCTAGCCGGGTTGTCAGCCATCCTTGCCT 4302

AGJ-3_15 (*r7A*) GCACTGCCGGCGTGCAGATCACCATCTATGTGCTAGCCGGGTTGTCAGCCATCCTTGCCT 4533

AGJ-3_3 (*r7B*) GCACTGCCGGCGTGCAGATCACCATCTATGTGCTAGCCGGGTTGTCAGCCATCCTTGCCT 4681

AGJ-3_1 (*r7B*) GCACTGCCGGCGTGCAGATCACCATCTATGTGCTAGCCGGGTTGTCAGCCATCCTTGCCT 4681

AGJ-3_20 (*r7B*) GCACTGCCGGCGTGCAGATCACCATCTATGTGCTAGCCGGGTTGTCAGCCATCCTTGCCT 4681

AY198374.1 GCACTGCCGGCGTGCAGATCACCATCTATGTGCTAGCCGGGTTGTCAGCCATCCTTGCCT 4780

AGJ-2_20 (*r6A*) GCACTGCCGGCGTGCAGATCACCATCTATGTGCTAGCCGGGTTGTCAGCCATCCTTGCCT 3729

AGJ-2_10 (*r6A*) GCACTGCCGGCGTGCAGATCACCATCTATGTGCTAGCCGGGTTGTCAGCCATCCTTGCCT 3729

AGJ-2_15 (*r6A*) GCACTGCCGGCGTGCAGATCACCATCTATGTGCTAGCCGGGTTGTCAGCCATCCTTGCCT 3729

************************************************************

AGJ-1_5 (*r5B*) TCCTGTGCCTTATTCTGCTCATCACATTCATCGTGAGGACCCGAGCTCTGAACCGCCGTT 4359

AGJ-1_6 (*r5B*) TCCTGTGCCTTATTCTGCTCATCACATTCATCGTGAGGACCCGAGCTCTGAACCGCCGTT 4359

AGJ-1_1 (*r5A*) TCCTGTGCCTTATTCTGCTCATCACATTCATCGTGAGGACCCGAGCTCTGAACCGCCGTT 4362

AGJ-1_3 (*r5C*) TCCTGTGCCTTATTCTGCTCATCACATTCATCGTGAGGACCCGAGCTCTGAACCGCCGTT 4381

AGJ-1_2 (*r5A*) TCCTGTGCCTTATTCTGCTCTTCACATTCATCGTGAGGACCCGAGCTCTGAACCGCCGTT 4362

AGJ-1_4 (*r5A*) TCCTGTGCCTTATTCTGCTCATCACATTCATCGTGAGGACCCGAGCTCTGAACCGCCGTT 4362

AGJ-3_15 (*r7A*) TCCTGTGCCTTATTCTGCTCATCACATTCATCGTGAGGACCCGAGCTCTGAACCGCCGTT 4593

AGJ-3_3 (*r7B*) TCCTGTGCCTTATTCTGCTCATCACATTCATCGTGAGGACCCGAGCTCTGAACCGCCGTT 4741

AGJ-3_1 (*r7B*) TCCTGTGCCTTATTCTGCTCATCACATTCATCGTGAGGACCCGAGCTCTGAACCGCCGTT 4741

AGJ-3_20 (*r7B*) TCCTGTGCCTTATTCTGCTCATCACATTCATCGTGAGGACCCGAGCTCTGAACCGCCGTT 4741

AY198374.1 TCCTGTGCCTTATTCTGCTCATCACATTCATCGTGAGGACCCGAGCTCTGAACCGCCGTT 4840

AGJ-2_20 (*r6A*) TCCTGTGCCTTATTCTGCTCATCACATTCATCGTGAGGACCCGAGCTCTGAACCGCCGCT 3789

AGJ-2_10 (*r6A*) TCCTGTGCCTTATTCTGCTCATCACATTCATCGTGAGGACCCGAGCTCTGAACCGCCGTT 3789

AGJ-2_15 (*r6A*) TCCTGTGCCTTATTCTGCTCATCACATTCATCGTGAGGACCCGAGCTCTGAACCGCCGTT 3789

********************:************************************* *

AGJ-1_5 (*r5B*) TGGAAGCACTGTCGATGACGAAATACGGCTCGGTGGATTCGGGGCTGAACCGAGTGGGGA 4419

AGJ-1_6 (*r5B*) TGGGAGCACTGTCGATGACGAAATACGGCTCGGTGGATTCGGGGCTGAACCGAGTGGGGA 4419

AGJ-1_1 (*r5A*) TGGAAGCACTGTCGATGACGAAATACGGCTCGGTGGATTCGGGGCTGAACCGAGTGGGGA 4422

AGJ-1_3 (*r5C*) TGGAAGCACTGTCGATGACGAAATACGGCTCGGTGGATTCGGGGCTGAACCGAGTGGGGA 4441

AGJ-1_2 (*r5A*) TGGAAGCACTGTCGATGACGAAATACGGCTCGGTGGATTCGGGGCTGAACCGAGTGGGGA 4422

AGJ-1_4 (*r5A*) TGGAAGCACTGTCGATGACGAAATACGGCTCGGTGGATTCGGGGCTGAACCGAGTGGGGA 4422

AGJ-3_15 (*r7A*) TGGAAGCACTGTCGATGACGAAATACGGCTCGGTGGATTCGGGGCTGAACCGAGTGGGGA 4653

AGJ-3_3 (*r7B*) TGGAAGCACTGTCGATGACGAAATACGGCTCGGTGGATTCGGGGCTGAACCGAGTGGGGA 4801

AGJ-3_1 (*r7B*) TGGAAGCACTGTCGATGACGAAATACGGCTCGGTGGATTCGGGGCTGAACCGAGTGGGGA 4801

AGJ-3_20 (*r7B*) TGGAAGCACTGTCGATGACGAAATACGGCTCGGTGGATTCGGGGCTGAACCGAGTGGGGA 4801

AY198374.1 TGGAAGCACTGTCGATGACGAAATACGGCTCGGTGGATTCGGGGCTGAACCGAGTGGGGA 4900

AGJ-2_20 (*r6A*) TGGAAGCACTGTCGATGACGAAATACGGCTCGGTGGATTCGGGGCTGAACCGAGTGGGGA 3849

AGJ-2_10 (*r6A*) TGGAAGCACTGTCGATGACGAAATACGGCTCGGTGGATTCGGGGCTGAACCGAGTGGGGA 3849

AGJ-2_15 (*r6A*) TGGAAGCACTGTCGATGACGAAATACGGCTCGGTGGATTCGGGGCTGAACCGAGTGGGGA 3849

***.********************************************************

AGJ-1_5 (*r5B*) TAGCGGCCCCAGGAACCAACAAACACGCCATCGAAGGCTCCAACCCCATCTGGAACCAGC 4479

AGJ-1_6 (*r5B*) TAGCGGCCCCAGGAACCAACAAACACGCCATCGAAGGCTCCAACCCCATCTGGAACGAGC 4479

AGJ-1_1 (*r5A*) TAGCGGCCCCAGGAACCAACAAACACGCCATCGAAGGCTCCAACCCCATCTGGAACGAGC 4482

AGJ-1_3 (*r5C*) TAGCGGCCCCAGGAACCAACAAACACGCCATCGAAGGCTCCAACCCCATCTGGAACGAGC 4501

AGJ-1_2 (*r5A*) TAGCGGCCCCAGGAACCAACAAACACGCCATCGAAGGCTCCAACCCCATCTGGAACGAGC 4482

AGJ-1_4 (*r5A*) TAGCGGCCCCAGGAACCAACAAACACGCCATCGAAGGCTCCAACCCCATCTGGAACGAGC 4482

AGJ-3_15 (*r7A*) TAGCGGCCCCAGGAACCAACAAACACGCCATCGAAGGCTCCAACCCCATCTGGAACGAGC 4713

AGJ-3_3 (*r7B*) TAGCGGCCCCAGGAACCAACAAACACGCCATCGAAGGCTCCAACCCCATCTGGAACGAGC 4861

AGJ-3_1 (*r7B*) TAGCGGCCCCAGGAACCAACAAACACGCCATCGAAGGCTCCGACCCCATCTGGAACGAGC 4861

AGJ-3_20 (*r7B*) TAGCGCCCCCAGGAACCAACAAACACGCCATCGAAGGCTCCAACCCCGTCTGGAACGAGC 4861

AY198374.1 TAGCGGCCCCAGGAACCAACAAACACGCCATCGAAGGCTCCAACCCCATCTGGAACGAGC 4960

AGJ-2_20 (*r6A*) TAGCGGCCCCAGGAACCAACAAACACGCCATCGAAGGCTCCAACCCCATCTGGAACGAGC 3909

AGJ-2_10 (*r6A*) TAGCGGCCCCAGGAACCAACAAACACGCCATCGAAGGCTCCAACCCCATCTGGAACGAGC 3909

AGJ-2_15 (*r6A*) TAGCGGCCCCAGGAACCAACAAACACGCCATCGAAGGCTCCAACCCCATCTGGAACGAGC 3909

***** ***********************************.*****.******** ***

AGJ-1_5 (*r5B*) AGATCAAGGCCCCGGACTTCGATGCCACCAGTGACACATCTGACGAGTCTGATCTGATCG 4539

AGJ-1_6 (*r5B*) AGATCAAGGCCCCGGACTTCGATGCCATCAGTGACACATCTGACGAGTCTGATCTGATCG 4539

AGJ-1_1 (*r5A*) AGATCAAGGCCCCGGACTTCGATGCCATCAGTGACACATCTGACGAGTCTGATCTGATCG 4542

AGJ-1_3 (*r5C*) AGATCAAGGCCCCGGACTTCGATGCCATCAGTGACACATCTGACGAGTCTGATCTGATCG 4561

AGJ-1_2 (*r5A*) AGATCAAGGCCCCGGACTTCGATGCCATCAGTGACACATCTGACGAGTCTGATCTGATCG 4542

AGJ-1_4 (*r5A*) AGATCAAGGCCCCGGACTTCGATGCCATCAGTGACACATCTGACGAGTCTGATCTGATCG 4542

AGJ-3_15 (*r7A*) AGATCAAGGCCCCGGACTTCGATGCCATCAGTGACACATCTGACGAGTCTGATCTGATCG 4773

AGJ-3_3 (*r7B*) AGATCAAGGCCCCGGACTTCGATGCCATCAGTGACACATCTGACGAGTCTGATCTGATCG 4921

AGJ-3_1 (*r7B*) AGATCAAGGCCCCGGACTTCGATGCCATCAGTGACACATCTGACGAGTCTGATCTGATCG 4921

AGJ-3_20 (*r7B*) AGATCAAGGCCCCGGACTTCGATGCCATCAGTGACACATCTGACGAGTCTGATCTGATCG 4921

AY198374.1 AGATCAAGGCCCCGGACTTCGATGCCATCAGTGACACATCTGACGAGTCTGATCTGATCG 5020

AGJ-2_20 (*r6A*) AGATCAAGGCCCCGGACTTCGATGCCATCAGTGACACATCTGACGAGTCTGATCTGATCG 3969

AGJ-2_10 (*r6A*) AGATCAAGGCCCCGGACTTCGATGCCATCAGTGACACATCTGACGAGTCTGATCTGATCG 3969

AGJ-2_15 (*r6A*) AGATCAAGGCCCCGGACTTCGATGCCATCAGTGACACATCTGACGAGTCTGATCTGATCG 3969

*************************** ********************************

AGJ-1_5 (*r5B*) GCATCGAGGATCTACCACAATTCAAGAGCGACTATTTCCCGCCTGAGGACTCGGAATCCG 4599

AGJ-1_6 (*r5B*) GCATCGAGGATCTACCACAATTCAAGAGCGACTATTTCTCGCCTGAGGACTCGGAATCCG 4599

AGJ-1_1 (*r5A*) GCATCGAGGATCTACCACAATTCAAGAGCGACTATTTCCCGCCTGAGGACTCGGAATCCG 4602

AGJ-1_3 (*r5C*) GCATCGAGGATCTACCACAATTCAAGAGCGACTATTTCCCGCCTGAGGACTCGGAATCCG 4621

AGJ-1_2 (*r5A*) GCATCGAGGATCTACCACAATTCAAGAGCGACTATTTCCCGCCTGAGGACTCGGAATCCG 4602

AGJ-1_4 (*r5A*) GCATCGAAGATCTACCACAATTCAAGAGCGACTATTTCCCGCCTGAGGACTCGGAATCCG 4602

AGJ-3_15 (*r7A*) GCATCGAGGATCTACCACAATTCAAGAGCGACTATTTCCCGCCTGAGGACTCGGAATCCG 4833

AGJ-3_3 (*r7B*) GCATCGAGGATCTACCACAATTCAAGAGCGACTATTTCCCGCCTGAGGACTCGGAATCCG 4981

AGJ-3_1 (*r7B*) GCATCGAGGATCTACCACAATTCAAGAGCGACTATTTCCCGCCTGAGGACTCGGAATCCG 4981

AGJ-3_20 (*r7B*) GCATCGAGGATCTACCACAATTCAAGAGCGACTATTTCCCGCCTGAGGACTCGGAATCCG 4981

AY198374.1 GCATCGAGGATCTACCACAATTCAAGAGCGACTATTTCCCGCCTGAGGACTCGGAATCCG 5080

AGJ-2_20 (*r6A*) GCATCGAGGATCTACCACAATTCAAGAGCGACTATTTCCCGCCTGAGGACTCGGAATCCG 4029

AGJ-2_10 (*r6A*) GCATCGAGGATCTACCACAATTCAAGAGCGACTATTTCCCGCCTGAGGACTCGGAATCCG 4029

AGJ-2_15 (*r6A*) GCATCGAGGATCTACCACAATTCAAGAGCGACTATTTCCCGCCTGAGGACTCGGAATCCG 4029

*******.****************************** *********************

AGJ-1_5 (*r5B*) CTCACGCCGCCTTTAGCGACCGCACGCCACGCGGGAACGATGCGCCTATTGCACACAGTA 4659

AGJ-1_6 (*r5B*) CTCACGCCGCCTTTAGCGACCGCACGCCACGCGGGAACGGTGCGCCTATTGCACACAGTA 4659

AGJ-1_1 (*r5A*) CTCACGCCGCCTTTAGCGACCGCACGCCACGCGGGAACGATGCGCCTATTGAACACAGTA 4662

AGJ-1_3 (*r5C*) CTCACGCCGCCTTTAGCGACCGCACGCCACGCGGGAACGATGCGCCTATTGCACACAGTA 4681

AGJ-1_2 (*r5A*) CTCACGCCGCCTTTAGCGACCGCACGCCACGCGGGAACGACGCGCCTATTGCACACAGTA 4662

AGJ-1_4 (*r5A*) CTCACGCCGCCTTTAGCGACCGCACGCCACGCGGGAACGATGCGCCTATTGCACACAGTA 4662

AGJ-3_15 (*r7A*) CTCACGCCGCCTTTAGCGACCGCACGCCACGCGGGAACGATGCGCCTATTGCACACAGTA 4893

AGJ-3_3 (*r7B*) CTCACGCCGCCTTTAGCGACCGCACGCCACGCGGGAACGATGCGCCTATTGCACACAGTA 5041

AGJ-3_1 (*r7B*) CTCACGCCGCCTTTAGCGACCGCACGCCACGCGGAAACGATGCGCCTATTGCACACAGTA 5041

AGJ-3_20 (*r7B*) CTCACGCCGCCTTTAGCGACCGCACGCCACGCGGGAACGATGCGCCTATTGCACACAGTA 5041

AY198374.1 CTCACGCCGCCTTTAGCGACCGCACGCCACGCGGGAACGATGCGCCTATTGCACACAGTA 5140

AGJ-2_20 (*r6A*) CTCACGCCGCCTTTAGCGATCGCACGCCACGCGGGAACGATGCGCCTATTGCACACAGTA 4089

AGJ-2_10 (*r6A*) CTCACGCCGCCTTTAGCGACCGCACGCCACGCGGGAACGATGCGCCTATTGCACACAGTA 4089

AGJ-2_15 (*r6A*) CTCACGCCGCCTTTAGCGACCGCACGCCACGCGGGAACGATGCGCCTATTGCACACAGTA 4089

******************* **************.****. **********.********

AGJ-1_5 (*r5B*) GCAACAACTTCGGTTTCAACACCAGCCCTTTTAGCGCGGAGTTCACTAACAGGCGCATGC 4719

AGJ-1_6 (*r5B*) GCAACAACTTCGGTTTCAACACCAGCCCTTTTAGCGCGGAGTTCACTAACAGGCGCATGC 4719

AGJ-1_1 (*r5A*) GCAACAACTTCGGTTTCAACACCAGCCCTTTTAGCGCGGAGTTCACTAACAGGCGCATGC 4722

AGJ-1_3 (*r5C*) GCAACAACTTCGGTTTCAACACCAGCCCTTTTAGCGCGGAGTTCACTAACAGGCGCATGC 4741

AGJ-1_2 (*r5A*) GCAACAACTTCGGTTTCAACACCAGCCCTTTTAGCGCGGAGTTCACTAACAGGCGCATGC 4722

AGJ-1_4 (*r5A*) GCAACAACTTCGGTTTCAACACCAGCCCTTTTAGCGCGGAGTTCACTAACAGGCGCATGC 4722

AGJ-3_15 (*r7A*) GCAACAACTTCGGTTTCAACACCAGTCCTTTTAGCGCGGAGTTCACTAACAGGCGCATGC 4953

AGJ-3_3 (*r7B*) GCAACAACTTCGGTTTCAACACCAGTCCTTTTAGCGCGGAGTTCACTAACAGGCGCATGC 5101

AGJ-3_1 (*r7B*) GCAACAACTTCGGTTTCAACACCAGTCCTTTTAGCGCGGAGTTCACTAACAGGCGCATGC 5101

AGJ-3_20 (*r7B*) GCAACAACTTCGGTTTCAACACCAGTCCTTTTAGCGCGGAGTTAACTAACAGGCGCATGC 5101

AY198374.1 GCAACAACTTCGGTTTCAACACCAGTCCTTTTAGCGCGGAGTTCACTAACAGGCGCATGC 5200

AGJ-2_20 (*r6A*) GCAACAACTTCGGTTTCAACACCAGCCCTTTTAGCGCGGAGTTCACTAACAGGCGCATGC 4149

AGJ-2_10 (*r6A*) GCAACAACTTCGGTTTCAACACCAGCCCTTTTAGCGCGGAGTTCACTAACAGGCGCATGC 4149

AGJ-2_15 (*r6A*) GCAACAACTTCGGTTTCAACACCAGCCCTTTTAGCGCGGAGTTCACTAACAGGCGCATGC 4149

************************* *****************.****************

AGJ-1_5 (*r5B*) GACCATAG 4727

AGJ-1_6 (*r5B*) GACCATAG 4727

AGJ-1_1 (*r5A*) GACCATAG 4730

AGJ-1_3 (*r5C*) GACCATAG 4749

AGJ-1_2 (*r5A*) GACCATAG 4730

AGJ-1_4 (*r5A*) GACCATAG 4730

AGJ-3_15 (*r7A*) GACCATAG 4961

AGJ-3_3 (*r7B*) GACCATAG 5109

AGJ-3_1 (*r7B*) GACCATAG 5109

AGJ-3_20 (*r7B*) GACCATAG 5109

AY198374.1 GACCATAG 5208

AGJ-2_20 (*r6A*) GACCATAG 4157

AGJ-2_10 (*r6A*) GACCATAG 4157

AGJ-2_15 (*r6A*) GACCATAG 4157

********
