# Supplementary material for: Alternative Splicing and Highly Variable Cadherin Transcripts Associated with Field-Evolved Resistance of Pink Bollworm to Bt Cotton in India
Source: PLoS One. 2014 May 19;9(5):e97900. doi: 10.1371/journal.pone.0097900 (PMC4026531; doi:10.1371/journal.pone.0097900)
Supplement: Figure S4 — Alignment of predicted amino acid sequences of pink bollworm cadherin from Anand, Gujarat (AGJ) with PgCad1 s (AY198374.1.1). Stars show amino acids conserved in all of the sequences. The symbols “:” and “.” indicate conservative amino acid substitutions scoring >0.5 and ≤0.5 in the Gonnet PAM 250 matrix, respectively. Red boxes show amino acids corresponding to lepidopteran cadherin Cry1Ac toxin binding regions. (DOCX) [file pone.0097900.s004.docx]

Figure S4. Alignment of predicted amino acid sequences of pink bollworm cadherin from Anand, Gujarat (AGJ) with *PgCad1* *s* (AY198374.1.1). Stars show amino acids conserved in all of the sequences. The symbols “:” and “.” indicate conservative amino acid substitutions scoring >0.5 and ≤0.5 in the Gonnet PAM 250 matrix, respectively. Red boxes show amino acids corresponding to lepidopteran cadherin Cry1Ac toxin binding regions.

AGJ-1_3 (*r5C*) MAGDACILVTVLLTFATSVFGQETASSRCYYMTDAIPREPKPDDLPDLEWTGGWTDWPLI 60

AGJ-1_5 (*r5B*) MAGDACILVTVLLTFATSVFGQET-SSRCYYMTDAIPREPKPDDLPDLEWTGGWTDWPLI 59

AGJ-1_6 (*r5B*) MAGDACILVTVLLTFATSVFGQET-SSRCYYMTDAIPREPKPDDLPDLEWTGGWTDWPLI 59

AGJ-1_2 (*r5A*) MAGDACILVTVLLTFATSVFGQETASSRCYYMTDAIPREPKPDGLPDLEWTGGWTDWPLI 60

AGJ-1_1 (*r5A*) MAGDACILVTVLLTFATAVFGQETASSRCYYMTDAIPREPKPDDLPDLEWTGGWTDWPLI 60

AGJ-1_4 (*r5A*) MAGDACILVTVLLTFATSVFGQETASSRCYYMTDAIPREPKPHDLPDLEWTGGWTDWPLI 60

AGJ-3_15 (*r7A*) MAGDACILVTVLLTFATSVFGQETASSRCYYMTDAIPREPKPDDLPDLEWTGGWTDWPLI 60

AGJ-2_20 (*r6A*) MAGDACILVTVLLTFATSVFGQETTSSRCYYMTDAIPREPKPDDLPDLEWTGGWTDWPLI 60

AGJ-2_10 (*r6A*) MAGDACILVTVLLTFATSVFGQETTSSRCYYMTDAIPREPKPDDLPDLEWTGGWTDWPLI 60

AGJ-2_15 (*r6A*) MAGDACILVTVLLTFATSVFGQETTSSRCYYMTDAIPREPKPDDLPDLEWTGGWTDWPLI 60

AGJ-3_1 (*r7B*) MAGDACILVTVLLTFATSVFGQETASSRCYYMTDAIPREPKPDDLPDLEWTGGWTDWPLI 60

AGJ-3_3 (*r7B*) MAGDACILVTVLLTFATSVFGQETASSRCYYMTDAIPREPKPDDLPDLEWAGGWTDWPLI 60

AGJ-3_20 (*r7B*) MAGDACILVTVLLTFATSVFGQETASSRCYYMTDAIPREPKPDDLPDLEWTGGWTDWPLI 60

AY198374.1 MAGDACILVTVLLTFATSVFGQETTSSRCYYMTDAIPREPKPDDLPDLEWTGGWTDWPLI 60

*****************:****** *****************. ******:*********

AGJ-1_3 (*r5C*) PAEPRDDVCINGWYPQLTSTSLGTIIIHMEEEIEGDVAIAKLNYDGSGTPEIVQPMVIGS 120

AGJ-1_5 (*r5B*) PAEPRDDVCINGWYPQLTSTSLGTIIIHMEEEIEGDVAIAKLNYDGSGTPEIVQPMVIGS 119

AGJ-1_6 (*r5B*) PAEPRDDVCINGWYPQLTSTSLGTIIIHMEEEIEGDVAIAKLNYDGSGTPEIVQPMVIGS 119

AGJ-1_2 (*r5A*) PAEPRDNVCINGWYPQLTSTSPGTIIIHMEEEIEGDVAIAKLNYDGSGTPEIVQPMVIGS 120

AGJ-1_1 (*r5A*) PAEPRDDVCINGWYPQLTSTSLGTIIIHMEEEIEGDVAIAKLNYDGSGTPEIVQPMVIGS 120

AGJ-1_4 (*r5A*) PAEPRDDVCINGWYPQLTSTSLGTIIIHMEEEIEGDVAIAKLNYDGSGTPEIVQPMVIGS 120

AGJ-3_15 (*r7A*) PAEPRDDVCINGWYPQLTSTSLGTIIIHMEEEIEGDVAIAKLNYDGSGTPEIVQPMVIGS 120

AGJ-2_20 (*r6A*) PAEPRDDVCINGWYPQLTSTSLGTIIIHMEEEIEGDVAIAKLNYDGSGTPEIVQPMVIGS 120

AGJ-2_10 (*r6A*) PAEPRDDVCINGWYPQLTSTSLGTIIIHMEEEIEGDVAIAKLNYDGSGTPEIVQPMVIGS 120

AGJ-2_15 (*r6A*) PAEPRDDVCINGWYPQLTSTSLGTIIIHMEEEIEGDVAIAKLNYDGSGTPEIVQPMVIGS 120

AGJ-3_1 (*r7B*) PAEPRDDVCINGWYPQLTSTSLGTIIIHMEEEIEGDVAIAKLNYDGSGTPEIVQPMVIGS 120

AGJ-3_3 (*r7B*) PAEPRDDVCINGWYPQLTSTSLGTIIIHMEEEIEGDVAIAKLNYDGSGTPEIVQPMVIGS 120

AGJ-3_20 (*r7B*) PAEPRDDVCINGWYPQLTSTSLGTIIIHMEEEIEGDVAIAKLNYDGSGTPEIVQPMVIGS 120

AY198374.1 PAEPRDDVCINGWYPQLTSTSLGTIIIHMEEEIEGDVAIAKLNYDGSGTPEIVQPMVIGS 120

******:************** **************************************

AGJ-1_3 (*r5C*) FNLLSPEIRNENGAWYLYITNSSELEIAGKIMKHQQC------------VGIHS------ 162

AGJ-1_5 (*r5B*) FNLLSPEIRNENGAWYLYITNRQDYETPTTRRYTFDVRVPDETRAARVSLSIENIDDNDP 179

AGJ-1_6 (*r5B*) FNLLSPEIRNENGAWYLYITNRQDYETPTMRRYTFDVRVPDETRAARVSLSIENIDDNDP 179

AGJ-1_2 (*r5A*) FNLLSPEIRNENGAWYLYITNRQDYETPTMRRYTFDVRVPDETRAARVSLSIENIDDNDP 180

AGJ-1_1 (*r5A*) FNLLSPEIRNENGAWYLYITNRQDYETPTMRRYTFDVRVPDETRAARVSLSIENIDDNDP 180

AGJ-1_4 (*r5A*) FNLLSPEIRNENGAWYLYITNRQDYETPTMRRYTFDVRVPDETRAARVSLSIENIDDNDP 180

AGJ-3_15 (*r7A*) FNLLSPEIRNENGAWYLYITNRQDYETPTMRRYTFDVRVPDETRAARVSLSIENIDDNDP 180

AGJ-2_20 (*r6A*) FNLLSPEIRNENGAWYLYITNRQDYETPTMRRYTFDVRVPDETRAARVSLSIENIDDNDP 180

AGJ-2_10 (*r6A*) FNLLSPEIRNENGAWYLYITNRQDYETPTMRRYTFDVRVPDETRAARVSLSIENIDDNDP 180

AGJ-2_15 (*r6A*) FNLLSPEIRNENGAWYLYITNRQDYETPTMRRYTFDVRVPDETRAARVSLSIENIDDNDP 180

AGJ-3_1 (*r7B*) FNLLSPEIRNENGAWYLYITNRQDYEAPTMRRYTFDVRVPDETRAARVSLSIENIDDNDP 180

AGJ-3_3 (*r7B*) FNLLSPEIRNENGAWYLYITNRQDYETPTMRRYTFDVRVPDETRAARVSLSIENIDDNDP 180

AGJ-3_20 (*r7B*) FNLLSPEIRNENGAWYLYITNRQDYETPTMRRYTFDVRVPDETRAARVSLSIENIDDNDP 180

AY198374.1 FNLLSPEIRNENGAWYLYITNRQDYETPTMRRYTFDVRVPDETRAARVSLSIENIDDNDP 180

********************* .: * : :.*..

AGJ-1_3 (*r5C*) ----TSECQTRLVPH--V------------------------------------------ 174

AGJ-1_5 (*r5B*) IVRVLDACQVPELGEPRLTDCVYQVSDEDGRLSIEPMTFRLTSDREDVQIFYVEPAHITG 239

AGJ-1_6 (*r5B*) IVRVLNACQVPELGEPRLTDCVYQVSDEDGRLSIEPMTFRLTSDREDVQIFYVEPAHITG 239

AGJ-1_2 (*r5A*) IVRVLDACQVPELGEPRLTDCVYQVSDEDGRLGIEPMTFRLTSDREDVQIFYVEPAHITG 240

AGJ-1_1 (*r5A*) IVRVLDACQVPELGEPRLTDCVYQVSDEDGRLSIEPMTFRLTSDREDVQIFYVEPAHITG 240

AGJ-1_4 (*r5A*) IVGVLDACQVPELGEPRLTDCVYQVSDEDGRLSIEPMTFRLTSDREDVQIFYVEPAHITG 240

AGJ-3_15 (*r7A*) IVRVLVACQVPELGEPRLTDCVYQVSDEDGRLSIEPMTFRLTSDREDVLIFYVEPAHITG 240

AGJ-2_20 (*r6A*) IVRVLDACQVPELGEPRLTDCVYQVSDEDGRLSIEPMTFRLTSDREDVQIFYVEPAHITG 240

AGJ-2_10 (*r6A*) IVRVLDACQVPELGEPRLTDCVYQVSDEDGRLSIEPMTFRLTSDREDVQIFYVEPAHITG 240

AGJ-2_15 (*r6A*) IVRVLDACQVPELGEPRLTDCVYQVSDEDGRLSIEPMTFRLTSDREDVQIFYVEPAHITG 240

AGJ-3_1 (*r7B*) IVRVLDACQVPELGEPRLTDCVYQVSDEDGRLSIEPMTFRLTSDREDVQIFYVEPAHITG 240

AGJ-3_3 (*r7B*) IVRVLDACQVPELGEPRLTDCGYQVSDEDGRLSIEPMTFRLTSDREDVQIFYVEPAHITG 240

AGJ-3_20 (*r7B*) IVRVLDACQVPELGEPRLTDCVYQVSDEDGRLSIEPMTFRLTSDREDVQIFYVEPAHITG 240

AY198374.1 IVRVLDACQVPELGEPRLTDCVYQVSDEDGRLSIEPMTFRLTSDREDVQIFYVEPAHITG 240

**. : . :

AGJ-1_3 (*r5C*) ------------------------------------------------------------ 174

AGJ-1_5 (*r5B*) DWFNMQITIGILSALNFESNPLHIFQITALDSWPNNHTVTVMVQVQNVEHRPPRWMEIFA 299

AGJ-1_6 (*r5B*) DWFNMQITIGILSALNFESNPLHIFQITALDSWPNNHTVTVMVQVQNVEHRPPRWMEIFA 299

AGJ-1_2 (*r5A*) DWFNMQITIGILSALNFESNPLHIFQITALDSWPNNHTVTVMVQVQNVEHRPPRWMEIFA 300

AGJ-1_1 (*r5A*) DWFNMQITIGILSALNFESNPLHIFRITALDSWPNNHTVTVMVQVQNVEHRPPRWMEIFA 300

AGJ-1_4 (*r5A*) DWFNMQITIGILSALNFESNPLHIFQITALDSWPNNHTVTVMVQVQNVEHRPPRWMEIFA 300

AGJ-3_15 (*r7A*) DWFNMQITIGILSALNFESNPLHIFQITALDSWPNNHTVTVMVQVQNVERRPPRWMEIFA 300

AGJ-2_20 (*r6A*) DWFNMQITIGILSALNFESNPLHIFQITALDSWPNNHAVTVMVQVQNVEHRPPRWMEIFA 300

AGJ-2_10 (*r6A*) DWFNMQITIGILSALNFESNPLHIFQITALDSWPNNHTVTVMVQVQNVEHRPPRWMEIFA 300

AGJ-2_15 (*r6A*) DWFNMQITIGILSALNFESNPLHIFQITALDSWPNNHTVTVMVQVQNVEHRPPRWMEIFA 300

AGJ-3_1 (*r7B*) DWFNMQITIGILSALNFESNPLHIFQITALDSWPNNHTVTVMVQVQNVEHRPPRWMEIFA 300

AGJ-3_3 (*r7B*) DWFNMQITIGILSALNFESNPLHIFQITALDSWPNNHTVTVMVQVQNVEHRPPRWMEIFA 300

AGJ-3_20 (*r7B*) DWFNMQITIGILSALNFESNPLHIFQITALDSWPNNHTVTVMVQVQNVEHRPPRWMEIFA 300

AY198374.1 DWFNMQITIGILSALNFESNPLHIFQITALDSWPNNHTVTVMVQVQNVEHRPPRWMEIFA 300

AGJ-1_3 (*r5C*) ------------------------------------------------------------ 174

AGJ-1_5 (*r5B*) VQQFDEMTEQQFQVRAIDGDTGIGKAIHYTLETDEEEDLFFIETLPGGHDGAIFSTAMID 359

AGJ-1_6 (*r5B*) VQQFDEMTEQQFQVRAIDGDTGIGKAIHYTLETDEEEDLFFIETLPGGHDGAIFSTAMID 359

AGJ-1_2 (*r5A*) VQQFDEMTEQQFQVRAIDGDTGIGKAIHYTLETDEEEDLFFIETLPGGHDGAIFSTAMID 360

AGJ-1_1 (*r5A*) VQQFDEMTEQQFQVRAIDGDTGIGKAIHYTLETDEEEDLFFIETLPGGHDGAIFSTAMID 360

AGJ-1_4 (*r5A*) VQQFDEMTEQQFQVRAIDGDTGIGKAIHYTLETDEEEDLFFIETLPGGHDGAIFSTAMID 360

AGJ-3_15 (*r7A*) VQQFDEMTEQQFQVRAIDGDTGIGKAIHYTLETDEEEDLFFIETLPGGHDGAIFSTAMID 360

AGJ-2_20 (*r6A*) VQQFDEMTEQQFQVRAIDGDTGIGKAIHYTLETDEEDAWI-------------------- 340

AGJ-2_10 (*r6A*) VQQFDEMTEQQFQVRAIDGDTGIGKAIHYTLETDEEDAWI-------------------- 340

AGJ-2_15 (*r6A*) VQQFDEMTEQQSQVRAIDGDTGIGKAIHYTLETDEEDAWI-------------------- 340

AGJ-3_1 (*r7B*) VQQFDEMTEQQLQVRAIDGDTGIGKAIHYTLETDEEEDLFFIETLPGGHDGAIFSTAMID 360

AGJ-3_3 (*r7B*) VQQFDEMTEQQFQVRAIDGDTGIGKAIHYTLETDEEEDLFFIGTLPGGHDGAIFSTAMID 360

AGJ-3_20 (*r7B*) VQQFDEMTEQQFQVRAIDGDTGIGKAIHYTLETDEEEDLFFIETLPGGHDGAIFSTAMID 360

AY198374.1 VQQFDEMTEQQFQVRAIDGDTGIGKAIHYTLETDEEEDLFFIETLPGGHDGAIFSTAMID 360

AGJ-1_3 (*r5C*) ------------------------------------------------------------ 174

AGJ-1_5 (*r5B*) VDRLRRDVFRLSLVVYKYDNVSFATPTPVVIIVNDINNKQPQPLQDEYTISIMEETPLSL 419

AGJ-1_6 (*r5B*) VDRLRRDVFRLSLVAYKYDNVSFATPTPVVIIVNDINNKQPQPLQDEYTISIMEETPLSL 419

AGJ-1_2 (*r5A*) VDRLRRDVFRLSLVAYKYDNVSFATPTPVVIIVNDINNKQPQPLQDEYTISIMEETPLSL 420

AGJ-1_1 (*r5A*) VDRLRRDVFRLSLVAYKYDNVSFATPTPVVIIVNDINNKQPQPLQDEYTISIMEETPLSL 420

AGJ-1_4 (*r5A*) VDRLRRDVFRLSLVAYKYDNVSFATPTPVVIIVNDINNKQPQPLQDEYTISIMEETPLSL 420

AGJ-3_15 (*r7A*) VDRLRRDVFRLSLVAYKYDNVSFATPTPVVIIVNDINNKKPQPLQDEYTISIMEETPLSL 420

AGJ-2_20 (*r6A*) ------------------------------------------------------------ 340

AGJ-2_10 (*r6A*) ------------------------------------------------------------ 340

AGJ-2_15 (*r6A*) ------------------------------------------------------------ 340

AGJ-3_1 (*r7B*) VDRLRRDVFRLSLVAYKYDNVSFATPTPVVIIVNDINNKKPQPLQDEYTISIMEETPLSL 420

AGJ-3_3 (*r7B*) VDRLRRDVFRLSLVAYKYDNVSFATPTPVVIIVNDINNKKPQPLQDEYTISIMEETPLSL 420

AGJ-3_20 (*r7B*) VDRLRRDVFRLSLVAYKYDNVSFATPTPVVIIVNDINNKKPQPLQDEYTISIMEETPLSL 420

AY198374.1 VDRLRRDVFRLSLVAYKYDNVSFATPTPVVIIVNDINNKKPQPLQDEYTISIMEETPLSL 420

AGJ-1_3 (*r5C*) ------------------------------------------------------------ 174

AGJ-1_5 (*r5B*) NFAEPFGFYDEDLIYAQFLVEIQGENPPGVEQAFYIAPTAGFQNQTFAIGTQDHRMLDYE 479

AGJ-1_6 (*r5B*) NFAEPFGFYDEDLIYAQFLVEIQGENPPGVEQAFYIAPTAGFQNQTFAIGTQDHRMLDYE 479

AGJ-1_2 (*r5A*) NFAELFGFYDEDLIYAQFLVEIQGENPPGVEQAFYIAPAAGFQNQTFAIGTQDHRMLDYE 480

AGJ-1_1 (*r5A*) NFAELFGFYDEDLIYAQFLVEIQGENPPGVEQAFYIAPTAGFQNQTFAIGTQDHRMLDYE 480

AGJ-1_4 (*r5A*) NFAELFGFYDEDLIYAQFLVEIQGENPPGVEQAFYIAPTAGFQNQTFAIGTQDHRMLDYE 480

AGJ-3_15 (*r7A*) NFAELFGFYDEDLIYAQFLVEIQGENPPGVEQAFYIAPTAGFQNQTFAIGTQDHRMLDYE 480

AGJ-2_20 (*r6A*) ------------------------------------------------------------ 340

AGJ-2_10 (*r6A*) ------------------------------------------------------------ 340

AGJ-2_15 (*r6A*) ------------------------------------------------------------ 340

AGJ-3_1 (*r7B*) NFAELFGFYDEDLIYAQFLVEIQGENPPGVEQAFYIAPTAGFQNQTFAIGTQDHRMLDYE 480

AGJ-3_3 (*r7B*) NFAELFGFYDEDLIYAQFLVEIQGENPPGVEQAFYIAPTAGFQNQTFAIGTQDHRMLDYE 480

AGJ-3_20 (*r7B*) NFAELFGFYDEDLIYAQFLVEIQGENPPGVEQAFYIAPTAGFQNQTFAIGTQDHRMLDYE 480

AY198374.1 NFAELFGFYDEDLIYAQFLVEIQGENPPGVEQAFYIAPTAGFQNQTFAIGTQDHRMLDYE 480

AGJ-1_3 (*r5C*) ------------------------------------------------------------ 174

AGJ-1_5 (*r5B*) DVPFQNIKLKVIATDRDNTNFTGVAEVNANLINWNDEEPIIEEDQLVVKFKETVPKDYHV 539

AGJ-1_6 (*r5B*) DVPFQNIKLKVIATDRDNTNFTGVAEVNVSLINWNDEEPIFEEDQLVVKFKETVPKDYHV 539

AGJ-1_2 (*r5A*) DVPFQNIKLKVIATDRDNTNFTGVAEVNVNLINWNDEEPIFEEDQLVVKFKETVPKDYHV 540

AGJ-1_1 (*r5A*) DVPFQNIKLKVIATDRDNTNFTGVAEVNVNLINWNDEEPIFEEDQLVVKFKETVPKDYHV 540

AGJ-1_4 (*r5A*) DVPFQNIKLKVIATDRDNTNFTGVAEANVNLINWNDEEPIFEEDQLIVKFKETVPKDYHV 540

AGJ-3_15 (*r7A*) DVPFQNIELKVIATDRDNTNFTGVAEVNVNLINWNDEEPIFEEDQLVVKFKETVPKDYHV 540

AGJ-2_20 (*r6A*) ------------------------------------------------------------ 340

AGJ-2_10 (*r6A*) ------------------------------------------------------------ 340

AGJ-2_15 (*r6A*) ------------------------------------------------------------ 340

AGJ-3_1 (*r7B*) DVPFQNIKLKVIATDHDNTNFTGVAEVNVNLINWNDEEPIFEEDQLVVKFKETVPKDYHV 540

AGJ-3_3 (*r7B*) DVPFQNIKLKVIATDRDNTNFTGVAEVNVNLINWNDEEPIFEEDQLVVKFKETVPKDYHV 540

AGJ-3_20 (*r7B*) DVPFQNIKLKVIATDRDNTNFTGVAEVNVNLINWNDEEPIFEEDQLVVKFKETVPKDYHV 540

AY198374.1 DVPFQNIKLKVIATDRDNTNFTGVAEVNVNLINWNDEEPIFEEDQLVVKFKETVPKDYHV 540

AGJ-1_3 (*r5C*) ------------------------------------------------------------ 174

AGJ-1_5 (*r5B*) GRLRAHDRDIGDSVVHSILGNANTFLRIDEETGDIYVTIDDAFDYHRQNEFNIQVRAQDT 599

AGJ-1_6 (*r5B*) GRLRAHDRDIGDSVVHSILGNANTFLRIDEETGDIYVTIDDAFDYHRQNEFNIQVRAQDT 599

AGJ-1_2 (*r5A*) GRLRAHDRDIGDSVVHSILGNANTFLRIDEETGDIYVTIDDAFDYHRQNEFNIQVRARDT 600

AGJ-1_1 (*r5A*) GRLRAHDRDIGDSVVHSILGNANTFLRIDEETGDIYVTIDDAFDYHRQNEFNIQVRAQDT 600

AGJ-1_4 (*r5A*) GRLRAHDRDIGDSVVHSILGNANTFLRIDEETGDIYVTIDDAFDYHRQNEFNIQVRAQDT 600

AGJ-3_15 (*r7A*) GRLRAHDRDIGDSVVHSILGNANTFLRIDEETGDIYVAIDDASDYHRQNEFNIQVRAQDT 600

AGJ-2_20 (*r6A*) ------------------------------------------------------------ 340

AGJ-2_10 (*r6A*) ------------------------------------------------------------ 340

AGJ-2_15 (*r6A*) ------------------------------------------------------------ 340

AGJ-3_1 (*r7B*) GRLRAHDRDIGDSVVHSILGNANTFLRIDEETGDIYVAIDDAFDYHRQNEFNIQVRAQDT 600

AGJ-3_3 (*r7B*) GRLRAHDRDIGDSVVHSILGNANTFLRIDEETGDIYVAIDDAFDYHRQNEFNIQVRAQDT 600

AGJ-3_20 (*r7B*) GRLRAHDRDIGDSVVHSILGNANTFLRIDEETGDIYVAIDDAFDYHRQNEFNIQVRAQDT 600

AY198374.1 GRLRAHDRDIGDSVVHSILGNANTFLRIDEETGDIYVAIDDAFDYHRQNEFNIQVRAQDT 600

AGJ-1_3 (*r5C*) ------------------------------------------------------------ 174

AGJ-1_5 (*r5B*) MSEPESRHTATAQLVIELEDVNNTPPTLRLPRVSPSVEENVPEGFEINREITATDPDTTA 659

AGJ-1_6 (*r5B*) MSEPESRHTATAQPVIELEDVNNTPPTLRLPRVSPSVEENVPEGFEINREITATDPDTTA 659

AGJ-1_2 (*r5A*) MSEPESRHTATAQLVIELEDVNNTPPTLRLPRVSPSVEENVPEGFEINREITATDPDTTA 660

AGJ-1_1 (*r5A*) MSEPESRHTATAQLVIELEDVNNTPPTLRLPRVSPSVEENVPEGFEINREITATDPDTTA 660

AGJ-1_4 (*r5A*) MSEPESRHTATAQLVIELEDVNNTPPTLRLPRVSPSVEENVPEGFEINREITATDPDTTA 660

AGJ-3_15 (*r7A*) MSEPESRHTATAQLVIELEDVNNTPPTLRLPRVSPSVEENVPEGFEVNREITATDPDTTA 660

AGJ-2_20 (*r6A*) ------------------------------------------------------------ 340

AGJ-2_10 (*r6A*) ------------------------------------------------------------ 340

AGJ-2_15 (*r6A*) ------------------------------------------------------------ 340

AGJ-3_1 (*r7B*) MSEPESRHTATAQLVIELEDVNNTPPTLRLPRVSPSVEENVPEGFEVNREITATDPDTTA 660

AGJ-3_3 (*r7B*) MSEPESRHTATAQLVIELEDVNNTPPTLRLPRVSPSVEENVPEGFEVNREITATDPDTTA 660

AGJ-3_20 (*r7B*) MSEPESRHTATAQLVIELEDVNNTPPTLRLPRVSPSVEENVPEGFEVNREITATDPDTTA 660

AY198374.1 MSEPESRHTATAQLVIELEDVNNTPPTLRLPRVSPSVEENVPEGFEINREITATDPDTTA 660

AGJ-1_3 (*r5C*) ------------------------------------------------------------ 174

AGJ-1_5 (*r5B*) YLQFEIDWDTSFATKQGRDTNPVEFHGCVDIETIFPNPADTREAVGRVVAKEIRHNVTID 719

AGJ-1_6 (*r5B*) YLQFEIDWDTSFATKQGRDTNPVEFHGCVDIETIFPNPADTREAVGRVVAKEIRHNVTID 719

AGJ-1_2 (*r5A*) YLQFEIDWDTSFATKQGRDTNPVEFHGCVDIETIIPNPADTREAVGRVVAKEIRHNVTID 720

AGJ-1_1 (*r5A*) YLQFEIDWDTSFATKQGRDTNPVEFHGCVDIETIFPNPADTREAVGRVVAKEIRHNVTID 720

AGJ-1_4 (*r5A*) YLQFEIDWDTSFATKQGRDTNPVEFHGCVDIETIFPNPADTREAVGRVVAKEIRHNVTID 720

AGJ-3_15 (*r7A*) YLQFEIDWDTSFATKQGRDTNPVEFHGCVDIETIFPNPADTREAVGRVVAKEIRHNVTID 720

AGJ-2_20 (*r6A*) ------------------------------------------------------------ 340

AGJ-2_10 (*r6A*) ------------------------------------------------------------ 340

AGJ-2_15 (*r6A*) ------------------------------------------------------------ 340

AGJ-3_1 (*r7B*) YLQFEIDWDTSFATKQGRDTNPIEFHGCVDIETIFPNPADTREAVGRVVVKEIRHNVTID 720

AGJ-3_3 (*r7B*) YLQFEIDWDTSFATKQGRDTNPIEFHGCVDIETIFPNPADTREAVGRVVAKEIRHNVTID 720

AGJ-3_20 (*r7B*) YLQFEIDWDTSFATKQGRDTNPIEFHGCVDIETIFPNPADTREAVGRVVAKEIRHNVTID 720

AY198374.1 YLQFEIDWDTSFATKQGRDTNPIEFHGCVDIETIFPNPADTREAVGRVVAKEIRHNVTID 720

AGJ-1_3 (*r5C*) ------------------------------------------------------------ 174

AGJ-1_5 (*r5B*) FEEFEFLYLTVRVRDLHTEDGRDYDESTFTIIIIDMNDNWPIWASGFLNQTFSIRERSST 779

AGJ-1_6 (*r5B*) FEEFEFLYLTVRVRDLHTEDGRDYDESTFTIIIIDMNDNWPIWASGFLNQTFSIRERSST 779

AGJ-1_2 (*r5A*) FEEFEFLYLTVRVRDLHTEDGRDYDGSTFTIIIIDMNDNWPIWASGFLNQTFSIRERSST 780

AGJ-1_1 (*r5A*) FGEFEFLYLTVRVRDLHTEDGRDYDESTFTIIIIDMNDNWPTWASGFLNQTFSIRERSST 780

AGJ-1_4 (*r5A*) FEEFEFLYLTVRVRDLHTEDGRDYDESTFTIIIIDMNDNWPIWASGFLNQTFSIRERSST 780

AGJ-3_15 (*r7A*) FEEFEFLYLTVRVRDLHTEDGRDYDVSTFTIIIIDMNDNWPIWASGFLNQTFSIRERSST 780

AGJ-2_20 (*r6A*) ------------------------------------------------------------ 340

AGJ-2_10 (*r6A*) ------------------------------------------------------------ 340

AGJ-2_15 (*r6A*) ------------------------------------------------------------ 340

AGJ-3_1 (*r7B*) FEEFEFLYLTVRVRDLHTGDGRDYDESTFTIIIIDMNDNWPIWASGFLNQTFSIRERSST 780

AGJ-3_3 (*r7B*) FEEFEFLYLTVRVRDLHTDDGRDYDESTFTIIIIDMNDNRPIWASGFLNQTFSIRERSST 780

AGJ-3_20 (*r7B*) FEEFEFLYLTVRVRDLHTDDGRDYDESTFTIIIIDMNDNWPIWASGFLNQTFSIRERSST 780

AY198374.1 FEEFEFLYLTVRVRDLHTDDGRDYDESTFTIIIIDMNDNWPIWASGFLNQTFSIRERSST 780

AGJ-1_3 (*r5C*) ------------------------------------------------------------ 174

AGJ-1_5 (*r5B*) GVVIGSVLATDIDGPLYNQVRYTIIPQEDTPEGLVQIHFVTGQITVDENGAIDADIPPRW 839

AGJ-1_6 (*r5B*) GVVIGSVLATDIDGPLYNQVRYTIIPQEDTPEGLVQIHFVTGQITVDENGAIDADIPPRW 839

AGJ-1_2 (*r5A*) GVVIGSVLATDIDGPLYNQVRYTIIPQEDTPEGLVQIHFVTGQITVDENGAIDADIPPRW 840

AGJ-1_1 (*r5A*) GVVIGSVLATDIDGPLYNQVRYTIIPQEDTPEGLVQIHFVTGQITVDESGAIDADIPPRW 840

AGJ-1_4 (*r5A*) GVVIGSVLATDIDGPLYNQVRYTIIPQEDTPEGLVQIHFVTGQITVDENGAIDADIPPRW 840

AGJ-3_15 (*r7A*) GVVIGSVLATDIDGPLYNQVRYTIIPQEDTPEGLVQIHFVTGQITVDENGAIDADIPPRW 840

AGJ-2_20 (*r6A*) ------------------------------------------------------------ 340

AGJ-2_10 (*r6A*) ------------------------------------------------------------ 340

AGJ-2_15 (*r6A*) ------------------------------------------------------------ 340

AGJ-3_1 (*r7B*) GVVIGSVLATDIDGPLYNQVRYTIIPQEDTPEGLVQIHFVTGQITVDENGAIDADIPPRW 840

AGJ-3_3 (*r7B*) GVIIGSVLATDIDGPLYNQVRYTIIPQEDTPEGLVQIHFVTGQITVDENGAIDADIPPRW 840

AGJ-3_20 (*r7B*) GVVIGSVLATDIDGPLYNQVRYTIIPQEDTPEGLVQIHFVTGQITVDENGAIDADIPPRW 840

AY198374.1 GVVIGSVLATDIDGPLYNQVRYTIIPQEDTPEGLVQIHFVTGQITVDENGAIDADIPPRW 840

AGJ-1_3 (*r5C*) ------------------------------------------------------------ 174

AGJ-1_5 (*r5B*) HLNYTVIASDKCSEENEENCPPDPVFWDTLRDNVINIVDINNKVPAADLSRFNETVYIYE 899

AGJ-1_6 (*r5B*) HLNYTVIASDKCSEENEENCPPDPVFWDTLRDNVINIVDIDNKVPAADLSRFNETVYIYE 899

AGJ-1_2 (*r5A*) HLNYTVIASDKCSEENEENCPPDPVFWDTLRDNVINIVDINNKVPAADLSRFNETVYIYE 900

AGJ-1_1 (*r5A*) HLNYTVIASDKCSEENEENCPPDPVFWDTLRDNVINIVDINNKVPAADLSRFNETVYIYE 900

AGJ-1_4 (*r5A*) HLNYTVIASDKCSEENEENCPPDPVFWDTLRDNVINIVDINNKVPAADLSRFNETVYIYE 900

AGJ-3_15 (*r7A*) HLNYTVIASDKCFEENEENCPPDPVFWDTLGDNVINIVDINNKVPAADLSRFNETVYIYE 900

AGJ-2_20 (*r6A*) ------------------------------------------------------------ 340

AGJ-2_10 (*r6A*) ------------------------------------------------------------ 340

AGJ-2_15 (*r6A*) ------------------------------------------------------------ 340

AGJ-3_1 (*r7B*) HLNYTVIASDICSEENEENCPPDPVFWDTLGDNVINIVDINNKVPAADLSRFNETVYIYE 900

AGJ-3_3 (*r7B*) HLNYTVIASDKCSEENEENCPPDPVFWDTLGDNVINIVDINNKVPAADLSRFNETVYIYE 900

AGJ-3_20 (*r7B*) HLNYTVIASDKCSEENEENCPPDPVFWDTLGDNVINIVDINNKVPAADLSRFNETVYIYE 900

AY198374.1 HLNYTVIASDKCSEENEENCPPDPVFWDTLGDNVINIVDINNKVPAADLSRFNETVYIYE 900

AGJ-1_3 (*r5C*) ------------------------------------------------------------ 174

AGJ-1_5 (*r5B*) NAPDFTNVVKIYSIDEDRDEIYHTVRYQINYAVNQRLRDFFAIDLDSGQVYVENTNNELL 959

AGJ-1_6 (*r5B*) NAPDFTNVVKIYSIDEDRDEIYHTVRYQINYAVNQRLRDFFAIDLDSGQVYVENTNNELL 959

AGJ-1_2 (*r5A*) NAPDFTNVVKIYSIDEDRDEIYHTVRYQINYAVNQRLRDFFAIDLDSGQVYVENTNNELL 960

AGJ-1_1 (*r5A*) NAPDFTNVVKIYSIDEDRDEVYHTVRYQINYAVNQRLRDFFAIDLDSGQVYVENTNNELL 960

AGJ-1_4 (*r5A*) NAPDFTNVVKIYSIDEDRDEIYHTVRYQINYAVNQRLRDFFAIDLDSGQVYVENTNNELL 960

AGJ-3_15 (*r7A*) NAPDFTNVVKIYSINEDRDEIYHTVRYQINYAVNQRLRDFFAIDLDSGQVYVENTNNELL 960

AGJ-2_20 (*r6A*) ------------------------------------------------------------ 340

AGJ-2_10 (*r6A*) ------------------------------------------------------------ 340

AGJ-2_15 (*r6A*) ------------------------------------------------------------ 340

AGJ-3_1 (*r7B*) NAPDFTNVVKIHSIDEDRDEIYHTVRYQINYAVNQRLRDFFAIDLDSGQVYVENTNNELL 960

AGJ-3_3 (*r7B*) NAPDFTNVVKIYSIDEDRDEIYHTVRYQINYAVNQRLRDFFAIDLDSGQVYVENTNNELL 960

AGJ-3_20 (*r7B*) NAPDFTNVVKIYSIDEDRDEIYHTVRYQINYAVNQRLRDFFAIDLDSGQVYVENTNNELL 960

AY198374.1 NAPDFTNVVKIYSIDEDRDEIYHTVRYQINYAVNQRLRDFFAIDLDSGQVYVENTNNELL 960

AGJ-1_3 (*r5C*) ------------------------------------------------------------ 174

AGJ-1_5 (*r5B*) DRDRGEDQHRIFINLIDNFYSEGDGNRNVNTTEVLVILLDENDNAPELPTPEELSWSISE 1019

AGJ-1_6 (*r5B*) DRDRGEDQHRIFINLIDNFYSEGDGNRNVNTTEVLVILLDENDNAPELPTPEELSWSISE 1019

AGJ-1_2 (*r5A*) DRDRGEDQHRIFINLIDNFYSEGDGNRNVNTTEVLVILLDENDNAPELPTPEELSWSISE 1020

AGJ-1_1 (*r5A*) DRDRGEDQHRIFINLIDNFYSEGDGNRNVNTTEVLVILLDENDNAPELPTPEELSWSISE 1020

AGJ-1_4 (*r5A*) DRDRGEDQHRIFINLIDNFYSEGDGNRNVNTTEVLVILLDENDNAPELPTPEELSWSISE 1020

AGJ-3_15 (*r7A*) DRDRGEDQHRIFINLIDNFYSEGDGNRNVNTTEVLVILLDENDNAPELPTPEELSWSISE 1020

AGJ-2_20 (*r6A*) ------------------------------------------------------------ 340

AGJ-2_10 (*r6A*) ------------------------------------------------------------ 340

AGJ-2_15 (*r6A*) ------------------------------------------------------------ 340

AGJ-3_1 (*r7B*) DRDRGEDQHRIFINLIDNFYSEGDGNRNVNTTEVLVILLDENDNAPELPTPEELSWSISE 1020

AGJ-3_3 (*r7B*) DRDRGEDQHRIFINLIDNFYSEGDGNRNVNTTEVLVILLDENDNAPELPTPEELSWSISE 1020

AGJ-3_20 (*r7B*) DRDRGEDQHRIFINLIDNFYSEGDGNRNVNTTEVLVILLDENDNAPELPTPEELSWSISE 1020

AY198374.1 DRDRGEDQHRIFINLIDNFYSEGDGNRNVNTTEVLVILLDENDNAPELPTPEELSWSISE 1020

AGJ-1_3 (*r5C*) ------------------------------------------------------------ 174

AGJ-1_5 (*r5B*) NLQEGITLDGESDVIYAPDIAEEDTPNSHVGYAILAMTVTNRDLDTVPSLLNMLSPNNVT 1079

AGJ-1_6 (*r5B*) NLQEGITLDGESDVIYAPDIDEEDTPNSHVGYAILAMTVTNRDLDTVPRLLNMLSPNNVT 1079

AGJ-1_2 (*r5A*) NLQEGITLDGESDVIYAPDIDEEDTPNSHVGYAILAMTVTNRDLDTVPRLLNMLSPNNVT 1080

AGJ-1_1 (*r5A*) NLQEGITLDGESDVIYAPDIDEEDTPNSHVGYAILAMTVTNRDLDTVPRLLNMLSPNNVT 1080

AGJ-1_4 (*r5A*) NLQEGITLDGESDVIYAPDIDEEDTPNSHVGYAILAMTVTNRDLDTVPRLLNMLSPNNVT 1080

AGJ-3_15 (*r7A*) NLQEGITLDGESDVIYAPDIDEEDTPNSHVGYAILAMTVTNRDLDTVPRLLNMLSPNNVT 1080

AGJ-2_20 (*r6A*) ------------------------------------------------------------ 340

AGJ-2_10 (*r6A*) ------------------------------------------------------------ 340

AGJ-2_15 (*r6A*) ------------------------------------------------------------ 340

AGJ-3_1 (*r7B*) NLQEGITLDGESDVIYAPDIDEEDTPNSHVGYAILAMTVTNRDLDTVPRLLNMLSPNNVT 1080

AGJ-3_3 (*r7B*) NLQEGITLDGESDVIYAPDIDEEDTPNSHVGYAILAMTVTNRDLDTVPRLLNMLSPNNVT 1080

AGJ-3_20 (*r7B*) NLQEGITLDGESDVIYAPDIDEEDTPNSHVGYAILAMTVTNRDLDTVPRLLNMLSPNNVT 1080

AY198374.1 NLQEGITLDGESDVIYAPDIDEEDTPNSHVGYAILAMTVTNRDLDTVPRLLNMLSPNNVT 1080

AGJ-1_3 (*r5C*) ------------------------------------------------------------ 174

AGJ-1_5 (*r5B*) GFLQTAMPLRGYWGTYDISVLKKRLA---------------------------------- 1105

AGJ-1_6 (*r5B*) GFLQTAMPLRGYWGTYDISVLKKRLA---------------------------------- 1105

AGJ-1_2 (*r5A*) GFLQTAMPLRGYWGTYDISVLKKRLA---------------------------------- 1106

AGJ-1_1 (*r5A*) GFLQTAMPLRGYWGTYDISVLKKRLA---------------------------------- 1106

AGJ-1_4 (*r5A*) EFLQTAMPLRGYWGTYDISVLKKRLA---------------------------------- 1106

AGJ-3_15 (*r7A*) GFLQTAMPLRGYWGTYDISILLMHQTTSE------------------------------- 1109

AGJ-2_20 (*r6A*) ------------------------------------------------------------ 340

AGJ-2_10 (*r6A*) ------------------------------------------------------------ 340

AGJ-2_15 (*r6A*) ------------------------------------------------------------ 340

AGJ-3_1 (*r7B*) GFLQTAMPLRGYWGTYDISILAFDHGIPQQISHEVYELEIRPYNYNPPQFVFPESGTILR 1140

AGJ-3_3 (*r7B*) GFLQTAMPLRGYWGTYDISILAFDHGIPQQISHEVYESEIRPYNYNPPQFVFPESGTILR 1140

AGJ-3_20 (*r7B*) GFLQTAMPLRGYWGTYDISILAFDHGIPQQISHEVYELEIRPYNYNPPQFVFPESGTILR 1140

AY198374.1 GFLQTAMPLRGYWGTYDISILAFDHGIPQQISHEVYELEIRPYNYNPPQFVFPESGTILR 1140

AGJ-1_3 (*r5C*) ------------------------------------------------------------ 174

AGJ-1_5 (*r5B*) ------------------------------------------------------------ 1105

AGJ-1_6 (*r5B*) ------------------------------------------------------------ 1105

AGJ-1_2 (*r5A*) ------------------------------------------------------------ 1106

AGJ-1_1 (*r5A*) ------------------------------------------------------------ 1106

AGJ-1_4 (*r5A*) ------------------------------------------------------------ 1106

AGJ-3_15 (*r7A*) ------------------------------------------------------------ 1109

AGJ-2_20 (*r6A*) ------------------------------------------------------------ 340

AGJ-2_10 (*r6A*) ------------------------------------------------------------ 340

AGJ-2_15 (*r6A*) ------------------------------------------------------------ 340

AGJ-3_1 (*r7B*) LALERAVVNNVLSLVNGDPLDRIQAIDDDGLDAGVVTFDIVGDADASNYFRVNNDGDNFG 1200

AGJ-3_3 (*r7B*) LALERAVVNNVLSLVNGDPLDRIQAIDDDGLDAGVVTFDIVGDADASNYFRVNNDGDNFG 1200

AGJ-3_20 (*r7B*) LALERAVVNNVLSLVNGDPLDRIQAIDDDGLDAGVVTFDIVGDADASNYFRVNNDGDNFG 1200

AY198374.1 LALERAVVNNVLSLVNGDPLDRIQAIDDDGLDAGVVTFDIVGDADASNYFRVNNDGDNFG 1200

AGJ-1_3 (*r5C*) ------------------------------------------------------------ 174

AGJ-1_5 (*r5B*) ------------------------------------------------------------ 1105

AGJ-1_6 (*r5B*) ------------------------------------------------------------ 1105

AGJ-1_2 (*r5A*) ------------------------------------------------------------ 1106

AGJ-1_1 (*r5A*) ------------------------------------------------------------ 1106

AGJ-1_4 (*r5A*) ------------------------------------------------------------ 1106

AGJ-3_15 (*r7A*) ------------------------------------------------------------ 1109

AGJ-2_20 (*r6A*) ------------------------------------------------------------ 340

AGJ-2_10 (*r6A*) ------------------------------------------------------------ 340

AGJ-2_15 (*r6A*) ------------------------------------------------------------ 340

AGJ-3_1 (*r7B*) TLLLTQALPEEGKEFEVTIRATDGGTEPRSYSTDSTITVLFVPTLGDPIFQDNTYSVAFF 1260

AGJ-3_3 (*r7B*) TLLLTQALPEEGKEFEVSIRATDGGTEPRSYSTDSTITVLFVPTLGDPIFQDNTYSVAFF 1260

AGJ-3_20 (*r7B*) TLLLTQALPEEGKEFEVTIRATDGGTEPRSYSTDSTITVLFVPTLGDPIFQDNTYSVAFF 1260

AY198374.1 TLLLTQALPEEGKEFEVTIRATDGGTEPRSYSTDSTITVLFVPTLGDPIFQDNTYSVAFF 1260

AGJ-1_3 (*r5C*) ------------------------------------------------------------ 174

AGJ-1_5 (*r5B*) ------------------------------------------------------------ 1105

AGJ-1_6 (*r5B*) ------------------------------------------------------------ 1105

AGJ-1_2 (*r5A*) ------------------------------------------------------------ 1106

AGJ-1_1 (*r5A*) ------------------------------------------------------------ 1106

AGJ-1_4 (*r5A*) ------------------------------------------------------------ 1106

AGJ-3_15 (*r7A*) ------------------------------------------------------------ 1109

AGJ-2_20 (*r6A*) ------------------------------------------------------------ 340

AGJ-2_10 (*r6A*) ------------------------------------------------------------ 340

AGJ-2_15 (*r6A*) ------------------------------------------------------------ 340

AGJ-3_1 (*r7B*) EKEVGLTERFSLPHAEDPKNKLCTDDCHDIYYRIFGGVDYEPFDLGPVTNVIFLKSELDR 1320

AGJ-3_3 (*r7B*) EKEVGLTERFSLPHAEDPKNKLCTDDCHDIYYRIFGGVDYEPFDLDPATNVIFLKSELDR 1320

AGJ-3_20 (*r7B*) EKEVGLTERFSLPHAEDPKNKLCTDDCHDIYYRIFGGVDYEPFDLDPVTNVIFLKSELDR 1320

AY198374.1 EKEVGLTERFSLPHAEDPKNKLCTDDCHDIYYRIFGGVDYEPFDLDPVTNVIFLKSELDR 1320

AGJ-1_3 (*r5C*) ------------------------------------------------------------ 174

AGJ-1_5 (*r5B*) ------------------------------------------------------------ 1105

AGJ-1_6 (*r5B*) ------------------------------------------------------------ 1105

AGJ-1_2 (*r5A*) ------------------------------------------------------------ 1106

AGJ-1_1 (*r5A*) ------------------------------------------------------------ 1106

AGJ-1_4 (*r5A*) ------------------------------------------------------------ 1106

AGJ-3_15 (*r7A*) ------------------------------------------------------------ 1109

AGJ-2_20 (*r6A*) ------------------------------------------------------------ 340

AGJ-2_10 (*r6A*) ------------------------------------------------------------ 340

AGJ-2_15 (*r6A*) ------------------------------------------------------------ 340

AGJ-3_1 (*r7B*) ETTATHVVQVAASNSPTGGGIPLPGSLLTVTD---------------------------- 1352

AGJ-3_3 (*r7B*) ETTATHVVQVAASNSPTGGGIPLPGSLLTVTV---------------------------- 1352

AGJ-3_20 (*r7B*) ETTATHVVQVAASNSPTGGGIPLPGSLLTVTV---------------------------- 1352

AY198374.1 ETTATHVVQVAASNSPTGGGIPLPGSLLTVTVTVREADPRPVFEQRLYTAGISTSDNINR 1380

AGJ-1_3 (*r5C*) ------------------------------------------------------------ 174

AGJ-1_5 (*r5B*) ------------------------------------------------------------ 1105

AGJ-1_6 (*r5B*) ------------------------------------------------------------ 1105

AGJ-1_2 (*r5A*) ------------------------------------------------------------ 1106

AGJ-1_1 (*r5A*) ------------------------------------------------------------ 1106

AGJ-1_4 (*r5A*) ------------------------------------------------------------ 1106

AGJ-3_15 (*r7A*) ------------------------------------------------------------ 1109

AGJ-2_20 (*r6A*) ------------------------------------------------------------ 340

AGJ-2_10 (*r6A*) ------------------------------------------------------------ 340

AGJ-2_15 (*r6A*) ------------------------------------------------------------ 340

AGJ-3_1 (*r7B*) -----TATHSENAQLTYTIEDGSMAVDSTLEAVKDSAFHLNAQTGVLILRIQPTASMQGM 1407

AGJ-3_3 (*r7B*) -----TATHSENAQLTYTIEDGSMAVDSTLEAVKDSAFHLNAQTGVLILRIQPTASMQGM 1407

AGJ-3_20 (*r7B*) -----TATHSENAQLTYTIEDGSMAVDSTLEAVKDSAFHLNAQTGVLILRIQPTASMQGM 1407

AY198374.1 ELLTVRATHSENAQLTYTIEDGSMAVDSTLEAVKDSAFHLNAQTGVLILRIQPTASMQGM 1440

AGJ-1_3 (*r5C*) ------------------------------------------------------------ 174

AGJ-1_5 (*r5B*) ------------------------------------------------------------ 1105

AGJ-1_6 (*r5B*) ------------------------------------------------------------ 1105

AGJ-1_2 (*r5A*) ------------------------------------------------------------ 1106

AGJ-1_1 (*r5A*) ------------------------------------------------------------ 1106

AGJ-1_4 (*r5A*) ------------------------------------------------------------ 1106

AGJ-3_15 (*r7A*) ------------------------------------------------------------ 1109

AGJ-2_20 (*r6A*) ------------------------------------------------------------ 340

AGJ-2_10 (*r6A*) ------------------------------------------------------------ 340

AGJ-2_15 (*r6A*) ------------------------------------------------------------ 340

AGJ-3_1 (*r7B*) FEFSVIATDPDEKTDTAEVKVYLISSQNRVSFIFLNDVETVESNRDFIAETFSVGFNMTC 1467

AGJ-3_3 (*r7B*) FEFNVIATDPDEKTDTAEVKVYLISSQNRVSFIFLNDVETVESNRDFIAETFSVGFNMTC 1467

AGJ-3_20 (*r7B*) FEFNVIATDPDEKTDTAEVKVYLISSQNRVSFIFLNDVETVESNRDFIAETFSVGFNMTC 1467

AY198374.1 FEFNVIATDPDEKTDTAEVKVYLISSQNRVSFIFLNDVETVESNRDFIAETFSVGFNMTC 1500

AGJ-1_3 (*r5C*) ------------------------------------------------------------ 174

AGJ-1_5 (*r5B*) ------------------------------------------------------------ 1105

AGJ-1_6 (*r5B*) ------------------------------------------------------------ 1105

AGJ-1_2 (*r5A*) ------------------------------------------------------------ 1106

AGJ-1_1 (*r5A*) ------------------------------------------------------------ 1106

AGJ-1_4 (*r5A*) ------------------------------------------------------------ 1106

AGJ-3_15 (*r7A*) ------------------------------------------------------------ 1109

AGJ-2_20 (*r6A*) ------------------------------------------------------------ 340

AGJ-2_10 (*r6A*) ------------------------------------------------------------ 340

AGJ-2_15 (*r6A*) ------------------------------------------------------------ 340

AGJ-3_1 (*r7B*) NIDQVLPGTNDAGVIQEAMAEVHAHFIQDNIPVSADSIEELRSDTQLLRSVQGVLNQRLL 1527

AGJ-3_3 (*r7B*) NIDQVLPGTNDAGVIQEAMAEVHAHFIQDNIPVSADSIEELRSDTQLLRSVQGVLNQRLL 1527

AGJ-3_20 (*r7B*) NIDQVLPGTNDAGVIQEAMAEVHAHFIQDNIPVSADSIEELRSDTQLLRSVQGVLNQRLL 1527

AY198374.1 NIDQVLPGTNDAGVIQEAMAEVHAHFIQDNIPVSADSIEELRSDTQLLRSVQGVLNQRLL 1560

AGJ-1_3 (*r5C*) ------------------------------------------------------------ 174

AGJ-1_5 (*r5B*) ------------------------------------------------------------ 1105

AGJ-1_6 (*r5B*) ------------------------------------------------------------ 1105

AGJ-1_2 (*r5A*) ------------------------------------------------------------ 1106

AGJ-1_1 (*r5A*) ------------------------------------------------------------ 1106

AGJ-1_4 (*r5A*) ------------------------------------------------------------ 1106

AGJ-3_15 (*r7A*) ------------------------------------------------------------ 1109

AGJ-2_20 (*r6A*) ------------------------------------------------------------ 340

AGJ-2_10 (*r6A*) ------------------------------------------------------------ 340

AGJ-2_15 (*r6A*) ------------------------------------------------------------ 340

AGJ-3_1 (*r7B*) VLNDLVTGVSPDLGTAGVQITIYVLAGLSAILAFLCLILLITFIVRTRALNRRLEALSMT 1587

AGJ-3_3 (*r7B*) VLNDLVTGVSPDLGTAGVQITIYVLAGLSAILAFLCLILLITFIVRTRALNRRLEALSMT 1587

AGJ-3_20 (*r7B*) VLNDLVTGVSPDLGTAGVQITIYVLAGLSAILAFLCLILLITFIVRTRALNRRLEALSMT 1587

AY198374.1 VLNDLVTGVSPDLGTAGVQITIYVLAGLSAILAFLCLILLITFIVRTRALNRRLEALSMT 1620

AGJ-1_3 (*r5C*) ------------------------------------------------------------ 174

AGJ-1_5 (*r5B*) ------------------------------------------------------------ 1105

AGJ-1_6 (*r5B*) ------------------------------------------------------------ 1105

AGJ-1_2 (*r5A*) ------------------------------------------------------------ 1106

AGJ-1_1 (*r5A*) ------------------------------------------------------------ 1106

AGJ-1_4 (*r5A*) ------------------------------------------------------------ 1106

AGJ-3_15 (*r7A*) ------------------------------------------------------------ 1109

AGJ-2_20 (*r6A*) ------------------------------------------------------------ 340

AGJ-2_10 (*r6A*) ------------------------------------------------------------ 340

AGJ-2_15 (*r6A*) ------------------------------------------------------------ 340

AGJ-3_1 (*r7B*) KYGSVDSGLNRVGIAAPGTNKHAIEGSDPIWNEQIKAPDFDAISDTSDESDLIGIEDLPQ 1647

AGJ-3_3 (*r7B*) KYGSVDSGLNRVGIAAPGTNKHAIEGSNPIWNEQIKAPDFDAISDTSDESDLIGIEDLPQ 1647

AGJ-3_20 (*r7B*) KYGSVDSGLNRVGIAPPGTNKHAIEGSNPVWNEQIKAPDFDAISDTSDESDLIGIEDLPQ 1647

AY198374.1 KYGSVDSGLNRVGIAAPGTNKHAIEGSNPIWNEQIKAPDFDAISDTSDESDLIGIEDLPQ 1680

AGJ-1_3 (*r5C*) ------------------------------------------------------- 174

AGJ-1_5 (*r5B*) ------------------------------------------------------- 1105

AGJ-1_6 (*r5B*) ------------------------------------------------------- 1105

AGJ-1_2 (*r5A*) ------------------------------------------------------- 1106

AGJ-1_1 (*r5A*) ------------------------------------------------------- 1106

AGJ-1_4 (*r5A*) ------------------------------------------------------- 1106

AGJ-3_15 (*r7A*) ------------------------------------------------------- 1109

AGJ-2_20 (*r6A*) ------------------------------------------------------- 340

AGJ-2_10 (*r6A*) ------------------------------------------------------- 340

AGJ-2_15 (*r6A*) ------------------------------------------------------- 340

AGJ-3_1 (*r7B*) FKSDYFPPEDSESAHAAFSDRTPRGNDAPIAHSSNNFGFNTSPFSAEFTNRRMRP 1702

AGJ-3_3 (*r7B*) FKSDYFPPEDSESAHAAFSDRTPRGNDAPIAHSSNNFGFNTSPFSAEFTNRRMRP 1702

AGJ-3_20 (*r7B*) FKSDYFPPEDSESAHAAFSDRTPRGNDAPIAHSSNNFGFNTSPFSAELTNRRMRP 1702

AY198374.1 FKSDYFPPEDSESAHAAFSDRTPRGNDAPIAHSSNNFGFNTSPFSAEFTNRRMRP 1735
