# Supplementary material for: Alternative Splicing and Highly Variable Cadherin Transcripts Associated with Field-Evolved Resistance of Pink Bollworm to Bt Cotton in India
Source: PLoS One. 2014 May 19;9(5):e97900. doi: 10.1371/journal.pone.0097900 (PMC4026531; doi:10.1371/journal.pone.0097900)
Supplement: Figure S5 — Alignment of cadherin cDNA sequences of pink bollworm from Khandwa, Madhya Pradesh (KMP) with the susceptible allele PgCad1 s (AY198374.1). Twenty-three clones from five individuals (KMP-4, KMP-5, KMP-6, KMP-7, KMP-8) had thirteen isoforms of five r alleles [r8A (KJ480763), r8B (KJ480764), r9A (KJ480765), r9B (KJ480766), r10A (KJ480767), r10B (KJ480768), r10C (KJ480769), r11A (KJ480770), r11B (KJ480771), r12A (KJ480772), r12B (KJ480773), r12C (KJ480774), and r12D (KJ480775)] and two s alleles [clones KMP-8_5, KMP-8_24, and KMP-8_46 for s6A (KJ480754), clone KMP-8_35 for s6B (KJ480755), and clone KMP-8_3 for s7 (KJ480756)]. Stars show nucleotides conserved in all of the sequences. Deletions are highlighted in gray and insertions are highlighted in yellow. Codons highlighted in red indicate the positions of premature stop codons. (DOCX) [file pone.0097900.s005.docx]

**Figure S5. Alignment of cadherin cDNA sequences of pink bollworm from Khandwa, Madhya Pradesh (KMP) with the susceptible allele *PgCad1* *s* (AY198374.1).** Twenty-three clones from five individuals (KMP-4, KMP-5, KMP-6, KMP-7, KMP-8) had thirteen isoforms of five *r* alleles [*r8A* (KJ480763), *r8B* (KJ480764), *r9A* (KJ480765), *r9B* (KJ480766), *r10A* (KJ480767), *r10B* (KJ480768), *r10C* (KJ480769), *r11A* (KJ480770), *r11B* (KJ480771), *r12A* (KJ480772), *r12B* (KJ480773), *r12C* (KJ480774), and *r12D* (KJ480775)] and two *s* alleles [clones KMP-8_5, KMP-8_24, and KMP-8_46 for *s6A* (KJ480754), clone KMP-8_35 for *s6B* (KJ480755), and clone KMP-8_3 for *s7* (KJ480756)]. Stars show nucleotides conserved in all of the sequences. Deletions are highlighted in gray and insertions are highlighted in yellow. Codons highlighted in red indicate the positions of premature stop codons.

KMP-5_2 (*r10A*) ATGGCGGGTGACGCCTGCATACTGGTGACGGTGCTTCTCACCTTCGCAACATCAGTTTTC 60

KMP-5_11 (*r10B*) ATGGCGGGTGACGCCTGCATACTGGTGACGGTGCTTCTCACCTTCGCAACATCAGTTTTC 60

KMP-5_16 (*r10C*) ATGGCGGGTGACGCCTGCATACTGGTGACGGTGCTTCTCACCTTCGCAACATCAGTTTTC 60

KMP-5_18 (*r10A*) ATGGCGGGTGACGCCTGCATACTGGTGACGGTGCTTCTCACCTTCGCAACATCAGTTTTC 60

KMP-7_3 (*r12C*) ATGGCGGGTGACGCCTGCATACTGGTGACGGTGCTTCTGACCTTCGCAACATCAGTTTTC 60

KMP-7_1 (*r12A*) ATGGCGGGTGACGCCTGCATACTGGTGACGGTGCTTCTGACCTTCGCAACATCAGTTTTC 60

KMP-7_4 (*r12D*) ATGGCGGGTGACGCCTGCATACTGGTGACGGTGCTTCTGACCTTCGCAACATCAGTTTTC 60

KMP-6_7 (*r12A*) ATGGCGGGTGACGCCTGCATACTGGTGACGGTGCTTCTGACCTTCGCAACATCAGTTTTC 60

KMP-7_2 (*r12B*) ATGGCGGGTGACGCCTGCATACTGGTGACGGTGCTTCTGACCTTCGCAACATCAGTTTTC 60

KMP-6_3 (*r11A*) ATGGCGGGTGACGCCTGCATACTGGTGACGGTGCTTCTCACCTTCGCAACATCAGTTTTC 60

KMP-6_5 (*r11B*) ATGGCGGGTGACGCCTGCATACTGGTGACGGTGCTTCTCACCTTCGCAACATCAGTTTTC 60

KMP-6_8 (*r11B*) ATGGCGGGTGACGCCTGCATACTGGTGACGGTGCTTCTCACCTTCGCAACATCAGTTTTC 60

KMP-8_3 (*s7*) ATGGCGGGTGACGCCTGCATACTGGTGACGGTGCTTCTCACCTTCGCAACATCAGTTTTC 60

KMP-4_20 (*r8B*) ATGGCGGGTGACGCCTGCATACTAGTGACGGTGCTTCTCACCTTCGCAACATCAGTTTTC 60

KMP-4_16 (*r8A*) ATGGCGGGTGACGCCTGCATACTGGTGACGGTGCTTCTCACCTTCGCAACATCAGTTTTC 60

KMP-4_26 (*r8A*) ATGGCGGGTGACGCCTGCATACTGGTGACGGTGCTTCTCACCTTCGCAACATCAGTTTTC 60

KMP-4_3 (*r8A*) ATGGCGGGTGACGCCTGCATACTGGTGACGGTGCTTCTCACCTTCGCAACATCAGTTTTC 60

KMP-4_8 (*r9A*) ATGGCGGGTGACGCCTGCATACTGGTGACGGTGCTTCTCACCTTCGCAACATCAGTTTTC 60

KMP-4_13 (*r9B*) ATGGCGGGTGACGCCTGCATACTGGTGACGGTGCTTCTCACCTTCGCAACATCAGTTTTC 60

AY198374.1 ATGGCGGGTGACGCCTGCATACTGGTGACGGTGCTTCTGACCTTCGCAACATCAGTTTTC 60

KMP-8_35 (*s6B*) ATGGCGGGTGACGCCTGCATACTGGTGACGGTGCTTCTCACCTTCGCAACATCAGTTTTC 60

KMP-8_24 (*s6A*) ATGGCGGGTGACGCCTGCATACTGGTGACGGTGCTTCTCACCTTCGCAACATCAGTTTTC 60

KMP-8_5 (*s6A*) ATGGCGGGTGACGCCTGCATACTGGTGACGGTGCTTCTCACCTTCGCAACATCAGTTTTC 60

KMP-8_46 (*s6A*) ATGGCGGGTGACGCCTGCATACTGGTGACGGTGCTTCTCACCTTCGCAACATCAGTTTTC 60

***********************.************** *********************

KMP-5_2 (*r10A*) GGGCAAGAAACAACATCGTCGAGATGTTACTACATGACTGACGCTATTCCGAGGGAACCG 120

KMP-5_11 (*r10B*) GGGCAAGAAACAACATCGTCGAGATGTTACTACATGACTGACGCTATTCCGAGGGAACCG 120

KMP-5_16 (*r10C*) GGGCAAGAAACAACATCGTCGAGATGTTACTACATGACTGACGCTATTCCGAGGGAACCG 120

KMP-5_18 (*r10A*) GGGCAAGAAACAACATCGTCGAGATGTTACTACATGACTGACGCTATTCCGAGGGAACCG 120

KMP-7_3 (*r12C*) GG-----------CATCGTCGAGATGTTACTACATGACTGACGCTATTCCGAGAGAACCG 109

KMP-7_1 (*r12A*) GGGCAAGAAACAGCATCGTCGAGATGTTACTACATGACTGACGCTATTCCGAGAGAACCG 120

KMP-7_4 (*r12D*) GG-----------CATCGTCGAGATGTTACTACATGACTGACGCTATTCCGAGAGAACCG 109

KMP-6_7 (*r12A*) GGGCAAGAAGCAGCATCGTCGAGATGTTACTACATGACTGACGCTATTCCGAGAGAACCG 120

KMP-7_2 (*r12B*) GGGCAAGAAA---CATCGTCGAGATGTTACTACATGACTGACGCTATTCCGGGAGAACCG 117

KMP-6_3 (*r11A*) GGGCAAGAAACAGCATCGTCGAGATGTTACTACATGACTGACGCTATTCCGAGAGAACCG 120

KMP-6_5 (*r11B*) GGGCAAGAAACAGCATCGTCGAGATGTTACTACATGACTGACGCTATTCCGAGAGAACCG 120

KMP-6_8 (*r11B*) GGGCAAGAAACAGCATCGTCGAGATGTTACTACATGACTGACGCTATTCCGAGAGAACCG 120

KMP-8_3 (*s7*) GGGCAAGAAA---CATCGTCGAGATGTTACTACATGACTGACGCTATTCCGAGAGAACCG 117

KMP-4_20 (*r8B*) GGGCAAGAAACAACATCGTCGAGATGTTACTACATGACTGACGCTATTCCGAGGGAACCG 120

KMP-4_16 (*r8A*) GGGCAAGAAACAACATCGTCGAGATGTTACTACATGACTGACGCTATTCCGAGGGAACCG 120

KMP-4_26 (*r8A*) GGGCAAGAAACAACATCGTCGAGATGTTACTACATGACTGACGCTATTCCGAGGGAACCG 120

KMP-4_3 (*r8A*) GGGCAAGAAACAACATCGTCGAGATGTTACTACATGACTGACGCTATTCCGAGGGAACCG 120

KMP-4_8 (*r9A*) GGGCAAGAAACAACATCGTCGAGATGTTACTACATGACTGACGCTATTCCGAGGGAACCG 120

KMP-4_13 (*r9B*) GGGCAAGAAACAACATCGTCGAGATGTTACTACATGACTGACGCTATTCCGAGGGAACCG 120

AY198374.1 GGGCAAGAAACAACATCGTCGAGATGTTACTACATGACTGACGCTATTCCGAGAGAACCG 120

KMP-8_35 (*s6B*) GGGCAAGAAACAGCATCGTCGAGATGTTACTACATGACTGACGCTATTCCGAGAGAACCG 120

KMP-8_24 (*s6A*) GGGCAAGAAA---CATCGTCGAGATGTTACTACATGACTGACGCTATTCCGAGAGAACCG 117

KMP-8_5 (*s6A*) GGGCAAGAAA---CATCGTCGAGATGTTACTACATGACTGACGCTATTCCGAGAGAACCG 117

KMP-8_46 (*s6A*) GGGCAAGAAA---CATCGTCGAGATGTTACTACATGACTGACGCTATTCCGAGAGAACCG 117

** **************************************.*.******

KMP-5_2 (*r10A*) AAACCGGATGATTTGCCTGACTTAGAATGGACTGGTGGATGGACCGACTGGCCTTTGATC 180

KMP-5_11 (*r10B*) AAACCGGATGATTTGCCTGACTTAGAATGGACTGGTGGATGGACCGACTGGCCTTTGATC 180

KMP-5_16 (*r10C*) AAACCGGATGATTTGCCTGACTTAGAATGGACTGGTGGATGGACCGACTGGCCTTTGATC 180

KMP-5_18 (*r10A*) AAACCGGATGATTTGCCTGACTTAGAATGGACTGGTGGATGGACCGACTGGCCTTTGATC 180

KMP-7_3 (*r12C*) AAACCGGATGATTTGCCTGATTTAGAATGGACTGGTGGATGGACCGACTGGCCTTTGATC 169

KMP-7_1 (*r12A*) AAACCGGATGATTTGCCTGATTTAGAATGGACTGGTGGATGGACCGACTGGCCTTTGATC 180

KMP-7_4 (*r12D*) AAACCGGATGATTTGCCTGATTTAGAATGGACTGGTGGATGGACCGACTGGCCTTTGATC 169

KMP-6_7 (*r12A*) AAACCGGATGATTTGCCTGATTTAGAATGGACTGGTGGATGGACCGACTGGCCTTTGATC 180

KMP-7_2 (*r12B*) AAACCGGATGATTTGCCTGATTTAGAATGGACTGGTGGATGGACCGACTGGCCTTTGATC 177

KMP-6_3 (*r11A*) AAACCGGATGATTTGCCTGATTTAGAATGGACTGGTGGATGGACCGACTGGCCTTTGATC 180

KMP-6_5 (*r11B*) AAACCGGATGATTTGCCTGATTTAGAATGGACTGGTGGATGGACCGACTGGCCTTTGATC 180

KMP-6_8 (*r11B*) AAACCGGATGATTTGCCTGATTTAGAATGGACTGGTGGATGGACCGACTGGCCTTTGATC 180

KMP-8_3 (*s7*) AAACCGGATGATTTGCCTGATTTAGAATGGACTGGTGGATGGACCGACTGGCCTTTGATC 177

KMP-4_20 (*r8B*) AAACCGGATGATTTGCCTGACTTAGAATGGACTGGTGGATGGACCGACTGGCCTTTGATC 180

KMP-4_16 (*r8A*) AAACCGGATGATTTGCCTGACTTAGAATGGACTGGTGGATGGACCGACTGGCCTTTGATC 180

KMP-4_26 (*r8A*) AAACCGGATGATTTGCCTGACTTAGAATGGACTGGTGGATGGACCGACTGGCCTTTGATC 180

KMP-4_3 (*r8A*) AAACCGGATGATTTGCCTGACTTAGAATGGACTGGTGGATGGACCGACTGGCCTTTGATC 180

KMP-4_8 (*r9A*) AAACCGGATGATTTGCCTGACTTAGAATGGACTGGTGGATGGACCGACTGGCCTTTGATC 180

KMP-4_13 (*r9B*) AAACCGGATGATTTGCCTGACTTAGAATGGACTGGTGGATGGACCGACTGGCCTTTGATC 180

AY198374.1 AAACCGGATGATTTGCCTGATTTAGAATGGACTGGTGGATGGACCGACTGGCCTTTGATC 180

KMP-8_35 (*s6B*) AAACCGGATGATTTGCCTGATTTAGAATGGACTGGTGGATGGACCGACTGGCCTTTGATC 180

KMP-8_24 (*s6A*) AAACCGGATGATTTGCCTGATTTAGAATGGACTGGTGGATGGACCGACTGGCCTTTGATC 177

KMP-8_5 (*s6A*) AAACCGGATGATTTGCCTGATTTAGAATGGACTGGTGGATGGACCGACTGGCCTTTGATC 177

KMP-8_46 (*s6A*) GAACCGGATGATTTGCCTGATTTAGAATGGACTGGTGGATGGACCGACTGGCCTTTGATC 177

******************* ***************************************

KMP-5_2 (*r10A*) CCGGCTGAGCCAAGAGACGACGTGTGCATAGACGGCTGGTACCCACAACTCACCAGCACT 240

KMP-5_11 (*r10B*) CCGGCTGAGCCAAGAGACGACGTGTGCATAAACGGCTGGTACCCACAACTCACCAGCACT 240

KMP-5_16 (*r10C*) CCGGCTGAGCCAAGAGACGACGTGTGCATAAACGGCTGGTACCCACAACTCACCAGCACT 240

KMP-5_18 (*r10A*) CCGGCTGAGCCAAGAGACGACGTGTGCATAAACGGCTGGTACCCACAACTCACCAGCACT 240

KMP-7_3 (*r12C*) CCGGCTGAGCCAAGAGACGACGTGTGCATAAACGGCTGGTACCCACAACTCACCAGCACT 229

KMP-7_1 (*r12A*) CCGGCTGAGCCAAGAGACGACGTGTGCATAAACGGCTGGTACCCACAACTCACCAGCACT 240

KMP-7_4 (*r12D*) CCGGCTGAGCCAAGAGACGACGTGTGCATAAACGGCTGGTACCCACAACTCACCAGCACT 229

KMP-6_7 (*r12A*) CCGGCTGAGCCAAGAGACGACGTGTGCATAAACGGCTGGTACCCACAACTCACCAGCACT 240

KMP-7_2 (*r12B*) CCGGCTGAGCCAAGAGACGACGTGTGCATAAACGGCTGGTACCCACAACTCACCAGCACT 237

KMP-6_3 (*r11A*) CCGGCTGAGCCAAGAGACGACGTGCGCATAAACGGCTGGTACCCACAACTCACCAGCACT 240

KMP-6_5 (*r11B*) CCGGCTGAGCCAAGAGACGACGTGTGCATAAACGGCTGGTACCCACAACTCACCAGCACT 240

KMP-6_8 (*r11B*) CCGGCTGAGCCAAGAGACGACGTGTGCATAAACGGCTGGTACCCACAACTCACCAGCACT 240

KMP-8_3 (*s7*) CCGGCTGAGCCAAGAGACGACGTGTGCATAAACGGCTGGTACCCACAACTCACCAGCACT 237

KMP-4_20 (*r8B*) CCGGCTGAGCCAAGAGACGACGTGTGCATAAACGGCTGGTACCCACAACTCACCAGCACT 240

KMP-4_16 (*r8A*) CCGGCTGAGCCAAGAGACGACGTGTGCATAAACGGCTGGTACCCACAACTCACCAGCACT 240

KMP-4_26 (*r8A*) CCGGCTGAGCCAAGAGACGACGTGTGCATAAACGGCTGGTACCCACAACTCACCAGCACT 240

KMP-4_3 (*r8A*) CCGGCTGAGCCAAGAGACGACGTGTGCATAAACGGCTGGTACCCACAACTCACCAGCACT 240

KMP-4_8 (*r9A*) CCGGCTGAGCCAAGAGACGACGTGTGCATAAACGGCTGGTACCCACAACTCACCAGCACT 240

KMP-4_13 (*r9B*) CCGGCTGAGCCAAGAGACGACGTGTGCATAAACGGCTGGTACCCACAACTCACCAGCACT 240

AY198374.1 CCGGCTGAGCCAAGAGACGACGTGTGCATAAACGGCTGGTACCCACAACTCACCAGCACT 240

KMP-8_35 (*s6B*) CCGGCTGAGCCAAGAGACGACGTGTGTATAAACGGCTGGTACCCACAACTCACCAGCACT 240

KMP-8_24 (*s6A*) CCGGCTGAGCCAAGAGACGACGTGTGCATAAACGGCTGGTACCCACAACTCACCAGCACT 237

KMP-8_5 (*s6A*) CCGGCTGAGCCAAGAGACGACGTGTGCATAAACGGCTGGTACCCACAACTCACCAGCACT 237

KMP-8_46 (*s6A*) CCGGCTGAGCCAAGAGACGACGTGTGCATAAACGGCTGGTACCCACAACTCACCAGCACT 237

************************ * ***.*****************************

KMP-5_2 (*r10A*) CCTCTCGGCACCATCATCATCCACATGGAAGAGGAGATCGAGGGAGATGTTGCTATCGCT 300

KMP-5_11 (*r10B*) TCTCTCGGCACCATCATCATCCACATGGAAGAGGAGATCGAGGGAGATGTTGCTATCGCT 300

KMP-5_16 (*r10C*) TCTCTCGGCACCATCATCATCCACATGGAAGAGGAGATCGAGGGAGATGTTGCTATCGCT 300

KMP-5_18 (*r10A*) TCTCTCGGCACCATCATCATCCACATGGAAGAGGAGATCGAGGGAGATGTTGCTATCGCT 300

KMP-7_3 (*r12C*) TCTCTCGGCACCATCATCATCCACATGGAAGAGGAGATCGAGGGAGATGTTGCTATCGCT 289

KMP-7_1 (*r12A*) TCTCTCGGCACCATCATCATCCACATGGAAGAGGAGATCGAGGGAGATGCTGCTATCGCT 300

KMP-7_4 (*r12D*) TCTCTCGGCACCATCATCATCCACATGGAAGAGGAGATCGAGGGAGATGTTGCTATCGCT 289

KMP-6_7 (*r12A*) TCTCTCGGCACCATCATCAGCCACATGGAAGAGGAGATCGAGGGAGATGTTGCTATCGCT 300

KMP-7_2 (*r12B*) TCTCTCGGCACCATCATCATCCACATGGAAGAGGAGATCGAGGGAGATGTTGCTATCGCT 297

KMP-6_3 (*r11A*) TCTCTCGGCACCATCATCATCCACATGGAAGAGGAGATCGAGGGAGATGTCGCTATCGCT 300

KMP-6_5 (*r11B*) TCTCTCGGCACCATCATCATCCACATGGAAGAGGAGATCGAGGGAGATGTCGCTATCGCT 300

KMP-6_8 (*r11B*) TCTCTCGGCACCATCATCATCCACATGGAAGAGGAGATCGAGGGAGATGTCGCTATCGCT 300

KMP-8_3 (*s7*) TCTCTCGGCACCATCATCATCCACATGGAAGAGGAGATCGAGGGAGATGTCGCTATCGCT 297

KMP-4_20 (*r8B*) TCTCTCGGCACCATCATCATCCACATGGAAGAGGAGATCGAGGGAGATGTTGCTATCGCT 300

KMP-4_16 (*r8A*) TCTCTCGGCACCATCATCATCCACATGGAAGAGGAGATCGAGGGAGATGTTGCTATCGCT 300

KMP-4_26 (*r8A*) TCTCTCGGCACCATCATCATCCACATGGAAGAGGAGATCGAGGGAGATGTTGCTATCGCT 300

KMP-4_3 (*r8A*) TCTCTCGGCACCATCATCATCCACATGGAAGAGGAGATCGAGGGAGATGTTGCTATCGCT 300

KMP-4_8 (*r9A*) TCTCTCGGCACCATCATCATCCACATGGAAGAGGAGATCGAGGGAGATGTTGCTATCGCT 300

KMP-4_13 (*r9B*) TCTCTCGGCACCATCATCATCCACATGGAAGAGGAGATCGAGGGAGATGTTGCTATCGCT 300

AY198374.1 TCTCTCGGCACCATCATCATCCACATGGAAGAGGAGATCGAGGGAGATGTTGCTATCGCT 300

KMP-8_35 (*s6B*) TCTCTCGGCACCATCATCATCCACATGGAAGAGGAGATCGAGGGAGATGTCGCTATCGCT 300

KMP-8_24 (*s6A*) TCTCTCGGCACCATCATCATCCACATGGAAGAGGAGATCGAGGGAGATGTCGCTATCGCT 297

KMP-8_5 (*s6A*) TCTCTCGGCACCATCATCATCCACATGGAAGAGGAGATCGAGGGAGATGTCGCTATCGCT 297

KMP-8_46 (*s6A*) TCTCTCGGCACCATCATCATCCACATGGAAGAGGAGATCGAGGGAGATGTCGCTATCGCT 297

****************** ***************************** *********

KMP-5_2 (*r10A*) AAACTTAACTATGATGGTTCTGGAACCCCAGAAATTGTCCAGCCGATGGTTGTAGGATCT 360

KMP-5_11 (*r10B*) AAACTTAACTATGATGGTTCTGGAACCCCAGAAATTGTCCAGCCGATGGTTATAGGATCT 360

KMP-5_16 (*r10C*) AAACTTAACTATGATGGTTCTGGAACCCCAGAAATTGTCCAGCCGATGGTTATAGGATCT 360

KMP-5_18 (*r10A*) AAACTTAACTATGATGGTTCTGGAACCCCAGAAATTGTCCAGCCGATGGTTATAGGATCT 360

KMP-7_3 (*r12C*) AAACTTAACTATGATGGTTCTGGAGCCCCAGAAATTGTCCAGCCGATGGTTATAGGATCT 349

KMP-7_1 (*r12A*) AAACTTAACTATGATGGTTCTGGAACCCCAGAAATTGTCCAGCCGATGGTTATAGGATCT 360

KMP-7_4 (*r12D*) AAACTTAACTATGATGGTTCTGGAACCCCAGAAATTGTCCAGCCGATGGTTATAGGATCT 349

KMP-6_7 (*r12A*) AAACTTAACTATGATGGTTCTGGAACCCCAGAAATTGTCCAGCCGATGGTTATAGGATCT 360

KMP-7_2 (*r12B*) AAACTTAACTATGATGGTTCTGGAACCCCAGAAATTGTCCAGCCGATGGTTATAGGATCT 357

KMP-6_3 (*r11A*) AAGCTTAACTATGATGGTTCTGGAACCCCAGAAATTGTCCAGCCGATGGTTATAGGATCT 360

KMP-6_5 (*r11B*) AAACTTAACTATGATGGTTCTGGAACCTCAGAAATTGTCCAGCCGATGGTTATAGGATCT 360

KMP-6_8 (*r11B*) AAACTTAGCTATGATGGTTCTGGAACCCCAGAAATTGTCCAGCCGATGGTTATAGGATCT 360

KMP-8_3 (*s7*) AAACTTAACTATGATGGTTCTGGAACCCCAGAAATTGTCCAGCCGATGGTTATAGGATCT 357

KMP-4_20 (*r8B*) AAACTTAACTATGATGGTTCTGGAACCCCAGAAATTGTCCAGCCGATGGTTATAGGATCT 360

KMP-4_16 (*r8A*) AAACTTAACTATGATGGTTCTGGAACCCCAGAAATTGTCCAGCCGATGGTTATAGGATCT 360

KMP-4_26 (*r8A*) AAACTTAACTATGATGGTTCTGGAACCCCAGAAATTGTCCAGCCGATGGTTATAGGATCT 360

KMP-4_3 (*r8A*) AAACTTAACTATGATGGTTCTGGAACCCCAGAAATTGTCCAGCCGATGGTTATAGGATCT 360

KMP-4_8 (*r9A*) AAACTTAACTATGATGGTTCTGGAACCCCAGAAATTGTCCAGCCGATGGTTATAGGATCT 360

KMP-4_13 (*r9B*) AAACTTAACTATGATGGTTCTGGAACCCCAGAAATTGTCCAGCCGATGGTTATAGGATCT 360

AY198374.1 AAACTTAACTATGATGGTTCTGGAACCCCAGAAATTGTCCAGCCGATGGTTATAGGATCT 360

KMP-8_35 (*s6B*) AAACTTAACTATGATGGTTCTGGAACCCCAGAAATTGTCCAGCCGATGGTTATAGGATCT 360

KMP-8_24 (*s6A*) AAACTTAACTATGATGGTTCTGGAACCCCAGAAATTGTCCAGCCGATGGTTATAGGATCT 357

KMP-8_5 (*s6A*) AAACTTAACTATGATGGTTCTGGAACCCCAGAAATTGTCCAGCCGATGGTTATAGGATCT 357

KMP-8_46 (*s6A*) AAACTTAACTATGATGGTTCTGGAACCCCAGAAATTGTCCAGCCGATGGTTATAGGATCT 357

**.****.****************.** ***********************.********

KMP-5_2 (*r10A*) TTTAACCTGCTAAGTCCAGAGATCCGGAATGAAAACGGGGCGTGGTACCTTTATATAACC 420

KMP-5_11 (*r10B*) TTTAACCTGCTAAGTCCAGAGATCCGGAATGAAAACGGGGCGTGGTACCTTTATATAACC 420

KMP-5_16 (*r10C*) TTTAACCTGCTAAGTCCAGAGATCCGGAATGAAAACGGGGCGTGGTACCTTTATATAACC 420

KMP-5_18 (*r10A*) TTTAACCTGCTAAGTCCAGAGATCCGGAATGAAAACGGGGCGTGGTACCTTTATATAACC 420

KMP-7_3 (*r12C*) TTTAACCTGCTAAGTCCAGAGATCCGGAATGAAAACGGGGCGTGGTACCTTTATATAACC 409

KMP-7_1 (*r12A*) TTTAACCTGCTAAGTCCAGAGATCCGGAATGAAAACGGGGCGTGGTACCTTTATATAACC 420

KMP-7_4 (*r12D*) TTTAACCTGCTAAGTCCAGAGATCCGGAATGAAAACGGGGCGTGGTACCTTTATATAACC 409

KMP-6_7 (*r12A*) TTTAACCTGCTAAGTCCAGAGATCCGGAATGAAAACGGGGCGTGGTACCTTTATATAACC 420

KMP-7_2 (*r12B*) TTTAACCTGCTAAGTCCAGAGATCCGGAATGAAAACGGGGCGTGGTACCTTTATATAACC 417

KMP-6_3 (*r11A*) TTTAACCTGCTAAGTCCAGAGATCCGGAATGAAAACGGGGCGTGGTACCTTTATATAACC 420

KMP-6_5 (*r11B*) TTTAACCTGCTAAGTCCAGAGATCCGGAATGAAAACGGGGCGTGGTACCTTTATATAACC 420

KMP-6_8 (*r11B*) TTTAACCTGCTAAGTCCAGAGATCCGGAATGAAAACGGGGCGTGGTACCTTTATATAACC 420

KMP-8_3 (*s7*) TTTAACCTGCTAAGTCCAGAGATCCGGAATGAAAACGGGGCGTGGTACCTTTATATAACC 417

KMP-4_20 (*r8B*) TTTAACCTGCTAAGTCCAGAGATCCGGAATGAAAACGGGGCGTGGTACCTTTATATAACC 420

KMP-4_16 (*r8A*) TCTAACCTGCTAAGTCCAGAGATCCGGAATGAAAACGGGGCGTGGTACCTTTATATAACC 420

KMP-4_26 (*r8A*) TTTAACCTGCTAAGTCCAGAGATCCGGAATGAAAACGGGGCGTGGTACCTTTATATAACC 420

KMP-4_3 (*r8A*) TTTAACCTGCTAAGTCCAGAGATCCGGAATGAAAACGGGGCGTGGTACCTTTATATAACC 420

KMP-4_8 (*r9A*) TTTAACCTGCTAAGTCCAGAGATCCGGAATGAAAACGGGGCGTGGTACCTTTATATAACC 420

KMP-4_13 (*r9B*) TTTAACCTGCTAAGTCCAGAGATCCGGAATGAAAACGGGGCGTGGTACCTTTATATAACC 420

AY198374.1 TTTAACCTGCTAAGTCCAGAGATCCGGAATGAAAACGGGGCGTGGTACCTTTATATAACC 420

KMP-8_35 (*s6B*) TTTAACCTGCTAAGTCCAGAGATCCGGAATGAAAACGGGGCGTGGTACCTTTATATAACC 420

KMP-8_24 (*s6A*) TTTAACCTGCTAAGTCCAGAGATCCGGAATGAAAACGGGGCGTGGTACCTTTATATAACC 417

KMP-8_5 (*s6A*) TTTAACCTGCTAAGTCCAGAGATCCGGAATGAAAACGGGGCGTGGTACCTTTATATAACC 417

KMP-8_46 (*s6A*) TTTAACCTGCTAAGTCCAGAGATCCGGAATGAAAACGGGGCGTGGTACCTTTATATAACC 417

* **********************************************************

KMP-5_2 (*r10A*) AATAGGCAAGATTATGAAACACCAACAATGCGTCGGTATACATTCGACGTCCGAGTGCCA 480

KMP-5_11 (*r10B*) AATAGGCAAGATTATGAAACACCAACAATGCGTCGGTATACATTCGACGTCCGAGTGCCA 480

KMP-5_16 (*r10C*) AATAGGCAAGATTATGAAACACCAACAATGCGTCGGTATACATTCGACGTCCGAGTGCCA 480

KMP-5_18 (*r10A*) AATAGGCAAGATTATGAAACACCAACAATGCGTCGGTATACATTCGACGTCCGAGTGCCA 480

KMP-7_3 (*r12C*) AATAG------------------------------------------------------- 414

KMP-7_1 (*r12A*) AATAGGCAAGATTATGAAACACCAACAATGCGTCGGTATACATTCGACGTCCGAGTGCCA 480

KMP-7_4 (*r12D*) AATAGGCAAGATTATGAAACACCAACAATGCGTCGGTATACATTCGACGTCCGAGTGCCA 469

KMP-6_7 (*r12A*) AATAGGCAAGATTATGAAACACCAACAATGCGTCGGTATACATTCGACGTCCGAGTGCCA 480

KMP-7_2 (*r12B*) AATAGGCAAGATTATGAAACACCAACAATGCGTCGGTATACATTCGACGTCCGAGTGCCA 477

KMP-6_3 (*r11A*) AATAGGCAAGATTATGAAACACCAACAATGCGTCGGTATACATTCGACGTCCGAGTGCCA 480

KMP-6_5 (*r11B*) AATAGGCAAGATTATGAAACACCAACAATGCGTCGGTATACATTCGACGTCCGAGTGCCA 480

KMP-6_8 (*r11B*) AATAGGCAAGATTATGAAACACCAACAATGCGTCGGTATACATTCGACGTCCGAGTGCCA 480

KMP-8_3 (*s7*) AATAGGCAAGATTATGAAACACCAACAATGCGTCGGTATACATTCGACGTCCGAGTGCCA 477

KMP-4_20 (*r8B*) AATAGGCAAGATTATGAAACACCAACAATGCGTCGGTATACATTCGACGTCCGAGTGCCA 480

KMP-4_16 (*r8A*) AATAGGCAAGATTATGAAACACCAACAATGCGTCGGTATACATTCGACGTCCGAGTGCCA 480

KMP-4_26 (*r8A*) AATAGGCAAGATTATGAAACACCAACAATGCGTCGGTATACATTCGACGTCCGAGTGCCA 480

KMP-4_3 (*r8A*) AATAGGCAAGATTATGAAACACCAACAATGCGTCGGTATACATTCGACGTCCGAGTGCCA 480

KMP-4_8 (*r9A*) AATAGGCAAGATTATGAAACACCAACAATGCGTCGGTGTACATTCGACGTCCGAGTGCCA 480

KMP-4_13 (*r9B*) AATAGGCAAGATTATGAAGCACCAACAATGCGTCGGTATACATTCGACGTCCGAGTGCCA 480

AY198374.1 AATAGGCAAGATTATGAAACACCAACAATGCGTCGGTATACATTCGACGTCCGAGTGCCA 480

KMP-8_35 (*s6B*) AATAGGCAAGATTATGAAACACCAACAATGCGTCGGTATACATTCGACGTCCGAGTGCCA 480

KMP-8_24 (*s6A*) AATAGGCAAGATTATGAAACACCAACAATGCGTCGGTATACATTCGACGTCCGAGTGCCA 477

KMP-8_5 (*s6A*) AATAGGCAAGATTATGAAACACCAACAATGCGTCGGTATACATTCGACGTCCGAGTGCCA 477

KMP-8_46 (*s6A*) AATAGGCAGGATTATGAAACACCAACAATGCGTCGGTATACATTCGACGTCCGAGTGCCA 477

*****

KMP-5_2 (*r10A*) GACGAGACTCGTGCGGCACGAGTGAGTCTGTCCATCGAAAACATTGACGATAACGACCCT 540

KMP-5_11 (*r10B*) GACGAGACTCGTGCGGCACGAGTGAGTCTGTCCATCGAAAACATTGACGATAACGACCCT 540

KMP-5_16 (*r10C*) GACGAGACTCGTGCGGCACGAGTGAGTCTGTCCATCGAAAACATTGACGATAACGACCCT 540

KMP-5_18 (*r10A*) GACGAGACTCGTGCGGCACGAGTGAGTCTGTCCATCGAAAACATTGACGATAACGACCCT 540

KMP-7_3 (*r12C*) ------------------------------------------------------------ 414

KMP-7_1 (*r12A*) GACGAGACTCGTGCCGCACGAGTGAGTCTCTCCATCGAAAACATTGACGATAACGACCCT 540

KMP-7_4 (*r12D*) GACGAGACTCGTGCCGCACGAGTGAGTCTCTCCATCGAAAACATTGACGATAACGACCCT 529

KMP-6_7 (*r12A*) GACGAGACTCGTGCCGCACGAGTGAGTCTCTCCATCGAAAACATTGACGATAACGACCCT 540

KMP-7_2 (*r12B*) GACGAGACTCGTGCCGCACGAGTGAGTCTCTCCATCGAAAACATTGACGATAACGACCCT 537

KMP-6_3 (*r11A*) GACGAGACTCGTGCCGCACGAGTGAGTCTCTCCATCGAAAACATTGACGATAACGACCCT 540

KMP-6_5 (*r11B*) GACGAGACTCGTGCGGCACGAGTGAGTCTCTCCATCGAAAACATTGACGATAACGACCCT 540

KMP-6_8 (*r11B*) GACGAGACTCGTGCGGCACGAGTGAGTCTCTCCATCGAAAACATTGACGATAACGACCCT 540

KMP-8_3 (*s7*) GACGAGACTCGTGCGGCACGAGTGAGTCTCTCCATCGAAAACATTGACGATAACGACCCT 537

KMP-4_20 (*r8B*) GACGAGACTCGTGCGGCACGAGTGAGTCTGTCCATCGAAAACATTGACGATAACGACCCT 540

KMP-4_16 (*r8A*) GACGAGACTCGTGCGGCACGAGTGAGTCTGTCCATCGAAAACATTGACGATAACGACCCT 540

KMP-4_26 (*r8A*) GACGAGACTCGTGCGGCACGAGTGAGTCTGTCCATCGAAAACATTGACGATAACGACCCT 540

KMP-4_3 (*r8A*) GACGAGACTCGTGCGGCACGAGTGAGTCTGTCCATCGAAAACATTGACGATAACGACCCT 540

KMP-4_8 (*r9A*) GACGAGACTCGTGCGGCACAAGTGAGTCTGTCCATCGAAAACATTGACGATAACGACCCT 540

KMP-4_13 (*r9B*) GACGAGACTCGTGCGGCACGAGTGAGTCTGTCCATCGAAAACATTGACGATAACGACCCT 540

AY198374.1 GACGAGACTCGTGCGGCACGAGTGAGTCTGTCCATCGAAAACATTGACGATAACGACCCT 540

KMP-8_35 (*s6B*) GACGAGACTCGTGCGGCACGAGTGAGTCTCTCCATCGAAAACATTGACGATAACGACCCT 540

KMP-8_24 (*s6A*) GACGAGACTCGTGCGGCACGAGTGAGTCTCTCCATCGAAAACATTGACGATAACGACCCT 537

KMP-8_5 (*s6A*) GACGAGACTCGTGCGGCACGAGTGAGTCTCTCCATCGAAAACATTGACGATAACGACCCT 537

KMP-8_46 (*s6A*) GACGAGACTCGTGCGGCACGAGTGAGTCTCTCCATCGAAAACATTGACGATAACGACCCT 537

KMP-5_2 (*r10A*) ATCGTCAGGGTGCTAGACGCTTGCCAAGTGCCGGAATTGGGGGAGCCTCGACTAACAGAC 600

KMP-5_11 (*r10B*) ATCGTCAGGGTGCTAGACGCTTGCCAAGTGCCGGAATTGGGGGAGCCTCGACTAACAGAC 600

KMP-5_16 (*r10C*) ATCGTCAGGGTGCTAGACGCTTGCCAAGTGCCGGAATTGGGGGAGCCTCGACTAACAGAC 600

KMP-5_18 (*r10A*) ATCGTCAGGGTGCTAGACGCTTGCCAAGTGCCGGAATTGGGGGAGCCTCGACTAACAGAC 600

KMP-7_3 (*r12C*) ---------------------------------GAATTGGGGGAGCCTCGACTAACTGAC 441

KMP-7_1 (*r12A*) ATCGTCAGGGTGCTAGACGCTTGCCAAGTGCCGGAATTGGGGGAGCCTCGACTAACTGAC 600

KMP-7_4 (*r12D*) ATCGTCAGGGTGCTGGACGCTTGCCAAGTGCCGGAATTGGGGGAGCCTCGACTAACTGAC 589

KMP-6_7 (*r12A*) ATCGTCAGCGTGCTAGACGCTTGCCAAGTGCCGGAATTGGGGGAGCCTCGACTAACTGAC 600

KMP-7_2 (*r12B*) ATCGTCAGGGTGCTAGACGCTTGCCAAGTGCCGGAATTGGGGGAGCCTCGACTAACTGAC 597

KMP-6_3 (*r11A*) ATCGTCAGCGTGCTAGACGCTTGCCAAGTGCCGGAATTGGGGGAGCCTCGACTAACTGAC 600

KMP-6_5 (*r11B*) ATCGTCAGGGTGCTAGACGCTTGCCAAGTGCCGGAATTGGGGGAGCCTCGACTAACTGAC 600

KMP-6_8 (*r11B*) ATCGTCAGGGTGCTAGACGCTTGCCAAGTGCCGGAATTGGGGGAGCCTCGACTAACTGAC 600

KMP-8_3 (*s7*) ATCGTCAGGGTGCTAGACGCTTGCCAAGTGCCGGAATTGGGGGAGCCTCGACTAACTGAC 597

KMP-4_20 (*r8B*) ATCGTCAGGGTGCTAGACGCTTGCCAAGTGCCGGAATTGGGGGAGCCTCGACTAACAGAC 600

KMP-4_16 (*r8A*) ATCGTCAGGGTGCTAGACGCTTGCCAAGTGCCGGAATTGGGGGAGCCTCGACTAACAGAC 600

KMP-4_26 (*r8A*) ATCGTCAGGGTGCTAGACGCTTGCCAAGTGCCGGAATTGGGGGAGCCTCGACTAACAGAC 600

KMP-4_3 (*r8A*) ATCGTCAGGGTGCTAGACGCTTGCCAAGTGCCGGAATTGGGGGAGCCTCGACTAACAGAC 600

KMP-4_8 (*r9A*) ATCGTCAGGGTGCTAGACGCTTGCCAAGTGCCGGAATTGGGGGAGCCTCGACTAACAGAC 600

KMP-4_13 (*r9B*) ATCGTCAGGGTGCTAGACGCTTGCCAAGTGCCGGAATTGGGGGAGCCTCGACTAACAGAC 600

AY198374.1 ATCGTCAGGGTGCTAGACGCTTGCCAAGTGCCGGAATTGGGGGAGCCTCGACTAACAGAC 600

KMP-8_35 (*s6B*) ATCGTCAGGGTGCTAGACGCTTGCCTAGTGCCGGAATTGGGGGAGCCTCGACTAACTGAC 600

KMP-8_24 (*s6A*) ATCGTCAGGGTGCTAGACGCTTGCCAAGTGCCGGAATTGGGGGAGCCTCGACTAACTGAC 597

KMP-8_5 (*s6A*) ATCGTCAGGGTGCTAGACGCTTGCCAAGTGCCGGAATTGGGGGAGCCTCGACTAACTGAC 597

KMP-8_46 (*s6A*) ATCGTCAGGGTGCTAGACGCTTGCCAAGTGCCGGAATTGGGGGAGCCTCGACTAACTGAC 597

***********************:***

KMP-5_2 (*r10A*) TGCGTTTACCAAGTGTCAGACGAAGATGGGAGGCTTAGTATCGAGCCCATGACATTCCGC 660

KMP-5_11 (*r10B*) TGCGTTTACCAAGTGTCAGACGAAGATGGGAGGCTTAGTATCGAGCCCATGACATTCCGC 660

KMP-5_16 (*r10C*) TGCGTTTACCAAGTGTCAGACGAAGATGGGAGGCTTAGTATCGAGCCCATGACATTCCGC 660

KMP-5_18 (*r10A*) TGCGTTTACCAAGTGTCAGACGAAGATGGGAGGCTTAGTATCGAGCCCATGACATTCCGC 660

KMP-7_3 (*r12C*) TGCGTTTACCAAGTGTCAGACGAAGATGGGAGGCCTAGTATCGAGCCCATGACATTCCGC 501

KMP-7_1 (*r12A*) TGCGTTTACCAAGTGTCAGACGAAGATGGGAGGCTTAGTATCGAGCCCATGACATTCCGC 660

KMP-7_4 (*r12D*) TGCGTTTACCAAGTGTCAGACGAAGATGGGAGGCTTAGTATCGAGCCCATGACATTCCGC 649

KMP-6_7 (*r12A*) TGCGTTTACCAAGTGTCAGACGAAGATGGGAGGCTTAGTATCGAGCCCATGACATTCCGC 660

KMP-7_2 (*r12B*) TGCGTTTACCAAGTGTCAGACGAAGATGGGAGGCTTAGTATCGAGCCCATGACATTCCGC 657

KMP-6_3 (*r11A*) TGCGTTTACCAAGTGTCAGACGAAGATGGGAGGCTTAGTATCGAGCCCATGACATTCCGC 660

KMP-6_5 (*r11B*) TGCGTTTACCAAGTGTCAGACGAAGATGGGAGGCTTAGTATCGAGCCCATGACATTCCGC 660

KMP-6_8 (*r11B*) TGCGTTTACCAAGTGTCAGACGAAGATGGGAGGCTTAGTATCGAGCCCATGACATTCCGC 660

KMP-8_3 (*s7*) TGCGTTTACCAAGTGTCAGACGAAGATGGGAGGCTTAGTATCGAGCCCATGACATTCCGC 657

KMP-4_20 (*r8B*) TGCGTTTACCAAGTGTCAGACGAAGATGGGAGGCTTAGTATCGAGCCCATGACATTCCGC 660

KMP-4_16 (*r8A*) TGCGTTTACCAAGTGTCAGACGAAGATGGGAGGCTTAGTATCGAGCCCATGACATTCCGC 660

KMP-4_26 (*r8A*) TGCGTTTACCAAGTGTCAGACGAAGATGGGAGGCTTAGTATCGGGCCCATGACATTCCGC 660

KMP-4_3 (*r8A*) TGCGTTTACCAAGTGTCAGACGAAGATGGGAGGCTTAGTATCGAGCCCATGACATTCCGC 660

KMP-4_8 (*r9A*) TGCGTTTACCAAGTGTCAGACGAAGATGGGAGGCTTAGTATCGAGCCCATGACATTCCGC 660

KMP-4_13 (*r9B*) TGCGTTTACCAAGTGTCAGACGAAGATGGGAGGCTTAGTATCGAGCCCATGACATTCCGC 660

AY198374.1 TGCGTTTACCAAGTGTCAGACGAAGATGGGAGGCTTAGTATCGAGCCCATGACATTCCGC 660

KMP-8_35 (*s6B*) TGCGTTTACCAAGTGTCAGACGAAGATGGGAGGCTTAGTATCGAGCCCATGACATTCCGC 660

KMP-8_24 (*s6A*) TGCGTTTACCAAGTGTCAGACGAAGATGGGAGGCTTAGTATCGAGCCCATGACATTCCGC 657

KMP-8_5 (*s6A*) TGCGTTTACCAAGTGTCAGACGAAGATGGGAGGCTTAGTATCGAGCCCATGACATTCCGC 657

KMP-8_46 (*s6A*) TGCGTTTACCAAGTGTCAGACGAAGATGGGAGGCTTAGTATCGAGCCCATGACATTCCGC 657

********************************** ********.****************

KMP-5_2 (*r10A*) CTCACATCAGACCGTGAAGACGTACAGATATTCTATGTGGAGCCAGCTCACATTACTGGT 720

KMP-5_11 (*r10B*) CTCACATCAGACCGTGAAGACGTACAGATATTCTATGTGGAGCCAGCTCACATTACTGGT 720

KMP-5_16 (*r10C*) CTCACATCAGACCGTGAAGACGTACAGATATTCTATGTGGAGCCAGCTCACATTACTGGT 720

KMP-5_18 (*r10A*) CTCACATCAGACCGTGAAGACGTACAGATATTCTATGTGGAGCCAGCTCACATTACTGGT 720

KMP-7_3 (*r12C*) CTCACATCAGACCGTGAAGACGTACAGATATTCTACGTGGAGCCAGCTCACATTACTGGT 561

KMP-7_1 (*r12A*) CTCACATCAGACCGTGAAGACGTACAGATATTCTATGTGGAGCCAGCTCACATTACTGGT 720

KMP-7_4 (*r12D*) CTCACATCAGACCGTGAAGACGTACAGATATTCTATGTGGAGCCAGCTCACATTACTGGT 709

KMP-6_7 (*r12A*) CTCACATCAGACCGTGAAGACGTACAGATATTCTATGTGGAGCCAGCTCACATTACTGGT 720

KMP-7_2 (*r12B*) CTCACATCAGACCGTGAAGACGTACAGATATTCTATGTGGAGCCAGCTCACATTACTGGT 717

KMP-6_3 (*r11A*) CTCACATCAGACCGTGAAGACGTACAGATATTCTATGTGGAGCCAGCTCACATTACTGGT 720

KMP-6_5 (*r11B*) CTCACATCAGACCGTGAAGACGTACAGATATTCTACGTGGAGCCAGCTCACATTACTGGA 720

KMP-6_8 (*r11B*) CTCACATCAGACCGTGAAGACGTACAGATATTCTACGTGGAGCCAGCTCACATTACTGGA 720

KMP-8_3 (*s7*) CTCACATCAGACCGTGAAGACGTACAGATATTCTACGTGGAGCCAGCTCACATTACTGGA 717

KMP-4_20 (*r8B*) CTCACATCAGACCGTGAAGACGTACAGATATTCTATGTGGAGCCAGCTCACATTACTGGT 720

KMP-4_16 (*r8A*) CTCACATCAGACCGTGAAGACGTACAGATATTCTATGTGGAGCCAGCTCACATTACTGGT 720

KMP-4_26 (*r8A*) CTCACATCAGACCGTGAAGACGTACAGATATTCTATGTGGAGCCAGCTCACATTACTGGT 720

KMP-4_3 (*r8A*) CTCACATCAGACCGTGAAGACGTACAGATATTCTATGTGGAGCCAGCTCACATTACTGGT 720

KMP-4_8 (*r9A*) CTCACATCAGACCGTGAAGACGTACAGATATTCTATGTGGAGCCAGCTCACATTACTGGT 720

KMP-4_13 (*r9B*) CTCACATCAGACCGTGAAGACGTACAGATATTCTATGTGGAGCCAGCTCACATTACTGGT 720

AY198374.1 CTCACATCAGACCGTGAAGACGTACAGATATTCTATGTGGAGCCAGCTCACATTACTGGT 720

KMP-8_35 (*s6B*) CTCACATCAGACCGTGAAGACGTACAGATATTCTACGTGGAGCCAGCTCACATTACTGGA 720

KMP-8_24 (*s6A*) CTCACATCAGACCGTGAAGACGTACAGATATTCTACGTGGAGCCAGCTCACATTACTGGA 717

KMP-8_5 (*s6A*) CTCACATCAGACCGTGAAGACGTACAGATATTCTACGTGGAGCCAGCTCACATTACTGGA 717

KMP-8_46 (*s6A*) CTCACATCAGACCGTGAAGACGTACAGATATTCTACGTGGAGCCAGCTCACATTACTGGA 717

*********************************** ***********************:

KMP-5_2 (*r10A*) GATTGGTTCAACATGCAAATTATTATCGGTATCCTATCAGCGCTTAACTTCGAAAGCAAC 780

KMP-5_11 (*r10B*) GATTGGTTCAACATGCAAATTACTATCGGTATCCTATCAGCGCTTAACTTCGAAAGCAAC 780

KMP-5_16 (*r10C*) GATTGGTTCAACATGCAAATTACTATCGGTATCCTATCAGCGCTTAACTTCGAAAGCAAC 780

KMP-5_18 (*r10A*) GATTGGTTCAACATGCAAATTACTATCGGTATCCTATCAGCGCTTAACTTCGAAAGCAAC 780

KMP-7_3 (*r12C*) GATTGGTTCAACATGCAAATTACTATCGGTATCCTATCAGCGCTTAACTTCGAAAGCAAC 621

KMP-7_1 (*r12A*) GATTGGTTCAACATGCAAATTACTATCGGTATCCTATCAGCGCTTAACTTCGAAAGCAAC 780

KMP-7_4 (*r12D*) GATTGGTTCAACATGCAAATTACTATCGGTATCCTATCAGCGCTTAACTTCGAAAGCAAC 769

KMP-6_7 (*r12A*) GATTGGTTCAACATGCAAATTACTATCGGTATCCTATCAGCGCTTAACTTCGAAAGCAAC 780

KMP-7_2 (*r12B*) GATTGGTTCAACATGCAAATTACTATCGGTATCCTATCAGCGCTTAACTTCGAAAGCAAC 777

KMP-6_3 (*r11A*) GATTGGTTCAACATGCAAATTACTATCGGTATCCTATCAGCGCTTAACTTCGAAAGCAAC 780

KMP-6_5 (*r11B*) GATTGGTTCAACATGCAAATTACTATCGGTATCCTATCAGCGCTTAACTTCGAAAGCAAC 780

KMP-6_8 (*r11B*) GATTGGTTCAACATGCAAATTACTATCGGTATCCTATCAGCGCTTAACTTCGAAAGCAAC 780

KMP-8_3 (*s7*) GATTGGTTCAACATGCAAATTACTATCGGTATCCTATCAGCGCTTAACTTCGAAAGCAAC 777

KMP-4_20 (*r8B*) GATTGGTTCAACATGCAAATTACTATCGGTATCCTATCAGCGCTTAACTTCGAAAGCAAC 780

KMP-4_16 (*r8A*) GATTGGTTCAACATGCAAATTACTATCGGTATCCTATCAGCGCTTAACTTCGAAAGCAAC 780

KMP-4_26 (*r8A*) GATTGGTTCAACATGCAAATTACTATCGGTATCCTATCAGCGCTTAACTTCGAAAGCAAC 780

KMP-4_3 (*r8A*) GATTGGTTCAACATGCAAATTACTATCGGTATCCTATCAGCGCTTAACTTCGAAAGCAAC 780

KMP-4_8 (*r9A*) GATTGGTTCAACATGCAAATTACTATCGGTATCCTATCAGCGCTTAACTTCGAAAGCAAC 780

KMP-4_13 (*r9B*) GATTGGTTCAACATGCAAATTACTATCGGTATCCTATCAGCGCTTAACTTCGAAAGCAAC 780

AY198374.1 GATTGGTTCAACATGCAAATTACTATCGGTATCCTATCAGCGCTTAACTTCGAAAGCAAC 780

KMP-8_35 (*s6B*) GATTGGTTCAACATGCAAATTACTATCGGTATCCTATCAGCGCTTAACTTCGAAAGCAGC 780

KMP-8_24 (*s6A*) GATTGGTTCAACATGCAAATTACTATCGGTATCCTATCAGCGCTTAACTTCGAAAGCAAC 777

KMP-8_5 (*s6A*) GATTGGTTCAACATGCAAATTACTATCGGTATCCTATCAGCGCTTAACTTCGAAAGCAAC 777

KMP-8_46 (*s6A*) GATTGGTTCAACATGCAAATTACTATCGGTATCCTATCAGCGCTTAACTTCGAAAGCAAC 777

********************** ***********************************.*

KMP-5_2 (*r10A*) CCGCTGCACATCTTTCAAATCACTGCTTTGGACTCCTGGCCCAACAACCATACGGTGACG 840

KMP-5_11 (*r10B*) CCGCTGCACATCTTTCAAATCACTGCTTTGGACTCCTGGCCCAACAACCATACGGTGACG 840

KMP-5_16 (*r10C*) CCGCTGCACATCTTTCAAATCACTGCTTTGGACTCCTGGCCCAACAACCATACGGTGACG 840

KMP-5_18 (*r10A*) CCGCTGCACATCCTTCAAATCACTGCTTTGGACTCCTGGCCCAACAACCATACGGTGACG 840

KMP-7_3 (*r12C*) CCGCTGCACATCTTTCAAATCACTGCTTTGGACTCCTGGCCCAACAACCATACGGTGACG 681

KMP-7_1 (*r12A*) CCGCTGCACATCTTTCAAATCACTGCTTTGGACTCCTGGCCCAACAACCATACGGTGACG 840

KMP-7_4 (*r12D*) CCGCTGCACATCTTTCAAATCACTGCTTTGGACTCCTGGCCCAACAACCATACGGTGACG 829

KMP-6_7 (*r12A*) CCGCTGCACATCTTTCAAATCACTGCTTTGGACTCCTGGCCCAACAACCATACGGTGACG 840

KMP-7_2 (*r12B*) CCGCTGCACATCTTTCAAATCACTGCTTTGGACTCCTGGCCCAACAACCATACGGTGACG 837

KMP-6_3 (*r11A*) CCGCTGCACATCTTTCAAATCACTGCTTTGGACTCCTGGCCCAACAACCATACGGTGACG 840

KMP-6_5 (*r11B*) CCGCTTCACATCTTTCAAATCACTGCTTTGGACTCCTGGCCCAACAACCATACGGTGACG 840

KMP-6_8 (*r11B*) CCGCTTCACATCTTTCAAATCACTGCTTTGGACTCCTGGCCCAACAACCATACGGTGACG 840

KMP-8_3 (*s7*) CCGCTTCACATCTTTCAAATCACTGCTTTGGACTCCTGGCCCAACAACCATACGGTGACG 837

KMP-4_20 (*r8B*) CCGCTGCACATCTTTCAAATCACTGCTTTGGACTCCTGGCCCAACAACCATACGGTGACG 840

KMP-4_16 (*r8A*) CCGCTGCACATCTTTCAAATCACTGCTTTGGACTCCTGGCCCAACAACCATACGGTGACG 840

KMP-4_26 (*r8A*) CCGCTGCACATCTTTCAAATCACTGCTTTGGACTCCTGGCCCAACAACCATACGGTGACG 840

KMP-4_3 (*r8A*) CCGCTGCACATCTTTCAAATCACTGCTTTGGACTCCTGGCCCAACAACCATACGGTGACG 840

KMP-4_8 (*r9A*) CCGCTGCACATCTTTCAAATCACTGCTTTGGACTCCTGGCCCAACAACCATACGGTGACG 840

KMP-4_13 (*r9B*) CCGCTGCACATCTTTCAAATCACTGCTTTGGACTCCTGGCCCAACAACCATACGGTGACG 840

AY198374.1 CCGCTGCACATCTTTCAAATCACTGCTTTGGACTCCTGGCCCAACAACCATACGGTGACG 840

KMP-8_35 (*s6B*) CCGCTTCACATCTTTCAAATCACTGCTTTGGACTCCTGGCCCAACAACCATACGGTGACG 840

KMP-8_24 (*s6A*) CCGCTTCACATCTTTCAAATCACTGCTTTGGACTCCTGGCCCAACAACCATACGGTGACG 837

KMP-8_5 (*s6A*) CCGCTTCACATCTTTCAAATCACTGCTTTGGACTCCTGGCCCAACAACCATACGGTGACG 837

KMP-8_46 (*s6A*) CCGCTTCACATCTTTCAAATCACTGCTTTGGACTCCTGGCCCAACAACCATACGGTGACG 837

***** ****** ***********************************************

KMP-5_2 (*r10A*) GTGATGGTGCAAGTCCAGAATGTGGAACACCGACCGCCGCGATGGATGGAAATCTTCGCA 900

KMP-5_11 (*r10B*) GTGATGGTGCAAGTCCAGAATGTGGAACATCGACCGCCGCGATGGATGGAAATCTTCGCA 900

KMP-5_16 (*r10C*) GTGATGGTGCAAGTCCAGAATGTGGAACACCGACCGCCGCGATGGATGGAAATCTTCGCA 900

KMP-5_18 (*r10A*) GTGATGGTGCAAGTCCAGAATGTGGAACACCGACCGCCGCGATGGATGGAAATCTTCGCA 900

KMP-7_3 (*r12C*) GTGATGGTGCAAGTCCAGAATGTGGAACACCGACCGCCGCGATGGATGGAAATCTTCGCA 741

KMP-7_1 (*r12A*) GTGATGGTGCAAGTCCAGAATGTGGAACACCGACCGCCGCGATGGATGGAAATCTTCGCA 900

KMP-7_4 (*r12D*) GTGATGGTGCAAGTCCAGAATGTGGAACACCGACCGCCGCGATGGATGGAAATCTTCGCA 889

KMP-6_7 (*r12A*) GTGATGGTGCAAGTCCAGAATGTGGAACACCGACCGCCGCGATGGATGGAAATCTTCGCA 900

KMP-7_2 (*r12B*) GTGATGGTGCAAGTCCAGAATGTGGAACACCGACCGCCGCGATGGATGGAAATCTTCGCA 897

KMP-6_3 (*r11A*) GTGATGGTGCAAGTCCAGAATGTGGAACACCGACCGCCGCGATGGATGGAAATCTTCGCA 900

KMP-6_5 (*r11B*) GTGATGGTGCAAGTCCAGAATGTGGAGCACCGACCGCCGCGATGGATGGAAATCTTCGCA 900

KMP-6_8 (*r11B*) GTGATGGTGCAAGTCCAGAATGTGGAGCACCGACCGCCGCGATGGATGGAAATCTTCGCA 900

KMP-8_3 (*s7*) GTGATGGTGCTAGTCCAGAATGTGGAACACCGACCGCCGCGATGGATGGAAATCTTCGCA 897

KMP-4_20 (*r8B*) GTGATGGTGCAAGTCCAGAATGTGGAACACCGACCGCCGCGATGGATGGAAATCTTCGCA 900

KMP-4_16 (*r8A*) GTGATGGTGCAAGTCCAGAATGTGGAACACCGACCGCCGCGATGGATGGAAATCTCCGCA 900

KMP-4_26 (*r8A*) GTGATGGTGCAAGTCCAGAATGTGGAACACCGACCGCCGCGATGGATGGAAATCTTCGCA 900

KMP-4_3 (*r8A*) GTGATGGTGCAAGTCCAGAATGTGGAACACCGACCGCCGCGATGGATGGAAATCTTCGCA 900

KMP-4_8 (*r9A*) GTGATGGTGCAAGTCCAGAATGTGGAACACCGACCGCCGCGATGGATGGAAATCTTCGCA 900

KMP-4_13 (*r9B*) GTGATGGTGCAAGTCCAGAATGTGGAACACCGACCGCCGCGATGGATGGAAATCTTCGCA 900

AY198374.1 GTGATGGTGCAAGTTCAGAATGTGGAGCACCGACCGCCGCGATGGATGGAAATCTTCGCA 900

KMP-8_35 (*s6B*) GTGATGGTGCAAGTCCAGAATGTGGAGCACCGACCGCCGCGATGGATGGAAATCTTCGCA 900

KMP-8_24 (*s6A*) GTGATGGTGCAAGTCCAGAATGTGGAGCACCGACCGCCGCGATGGATGGAAATCTTCGCA 897

KMP-8_5 (*s6A*) GTGATGGTGCAAGTCCAGAATGTGGAGCACCGACCGCCGCGATGGATGGAAATCTTCGCA 897

KMP-8_46 (*s6A*) GTGATGGTGAAAGTCCGGAATGTGGAGCACCGACCGCCGCGATGGATGGAAATCTTCGCA 897

*********.:*** *.*********.** ************************* ****

KMP-5_2 (*r10A*) GTCCAGCAGTTTGACGAGATAACGGAGCAGCAATTCCAGGTGCGCGCCATCGACGGAGAC 960

KMP-5_11 (*r10B*) GTCCAGCAGTTTGACGAGATGACGGAGCAGCAATTCCAGGTGCGCGCCATCGACGGAGAC 960

KMP-5_16 (*r10C*) GTCCAGCAGTTTGACGAGATGACGGAGCAGCAATTCCAGGTGCGCGCCATCGACGGAGAC 960

KMP-5_18 (*r10A*) GTCCAGCAGTTTGACGAGATGACGGAGCAGCAATTCCAGGTGCGCGCCATCGACGGAGAC 960

KMP-7_3 (*r12C*) GTCCAGCAGTTTGACGAGATGACGGAGCAGCAATTCCAGGTGCGCGCCATCGACGGAGAC 801

KMP-7_1 (*r12A*) GTCCAGCAGTTTGACGAGATGACGGAGCAGCAATTCCAGGTGCGCGCCATCGACGGAGAC 960

KMP-7_4 (*r12D*) GTCCAGCAGTTTGACGAGATGACGGAGCAGCAATTCCAGGTGCGCGCCATCGACGGAGAC 949

KMP-6_7 (*r12A*) GTCCAGCAGTTTGACGAGATGACGGAGCAGCAATTCCAGGTGCGCGCCATCGACGGAGAC 960

KMP-7_2 (*r12B*) GTCCAGCAGTTTGACGAGATGACGGAGCAGCAATTCCAGGTGCGCGCCATCGACGGAGAC 957

KMP-6_3 (*r11A*) GTCCAGCAGTTTGACGAGATGACGGAGCAGCAATTCCAGGTGCGCGCCATCGACGGAGAC 960

KMP-6_5 (*r11B*) GTCCAGCAGTTTGACGAGATGACGGAGCAGCAATTCCAGGTGCGCGCCATCGACGGAGAC 960

KMP-6_8 (*r11B*) GTCCAGCAGTTTGACGAGATGACGGAGCAGCAATTCCAGGTGCGCGCCATTGACGGAGAC 960

KMP-8_3 (*s7*) GTCCAGCAGTTTGACGAGATGACGGAGCAGCAATTCCAGGTGCGCGCCATCGACGGAGAC 957

KMP-4_20 (*r8B*) GTCCAGCAGTTTGACGAGATGACGGAGCAGCAATTCCAGGTGCGCGCCATCGACGGAGAC 960

KMP-4_16 (*r8A*) GTCCAGCAGTTTGACGAGATGACGGAGCAGCAATTCCAGGTGCGCGCCATCGACGGAGAC 960

KMP-4_26 (*r8A*) GTCCAGCAGTTTGACGAGATGACGGAGCAGCAATTCCAGGTGCGCGCCATCGACGGAGAC 960

KMP-4_3 (*r8A*) GTCCAGCAGTTTGACGAGATGACGGAGCAGCAATCCCAGGTGCGCGCCATCGACGGAGAC 960

KMP-4_8 (*r9A*) GTCCAGCAGTTTGACGAGATGACGGAGCAGCAATTCCAGGTGCGCGCCATCGACGGAGAC 960

KMP-4_13 (*r9B*) GTCCAGCAGTTTGACGAGATGACGGAGCAGCAATTCCAGGTGCGCGCCATCGACGGAGAC 960

AY198374.1 GTCCAGCAGTTTGACGAGATGACGGAGCAGCAATTCCAGGTGCGCGCCATCGACGGAGAC 960

KMP-8_35 (*s6B*) GTCCAGCAGTTTGACGAGATGACGGAGCAGCAGTTCCAGGTGCGCGCCATCGACGGAGAC 960

KMP-8_24 (*s6A*) GTCCAGCAGTTTGACGAGATGACGGAGCAGCAATTCCAGGTGCGCGCCATCGACGGAGAC 957

KMP-8_5 (*s6A*) GTCCAGCAGTTTGACGAGATGACGGAGCAGCAATTCCAGGTGCGCGCCATCGACGGAGAC 957

KMP-8_46 (*s6A*) GTCCAGCGGTTTGACGAGATGACGGAGCAGCAATTCCAGGTGCGCGCCATCGACGGAGAC 957

*******.************.***********.* *************** *********

KMP-5_2 (*r10A*) ACTGGCATCGGGAAAGCTATACACTATACCCTCGAGACAGATGAGGAGGGAGATTTGTTC 1020

KMP-5_11 (*r10B*) ACTGGCATCGGGAAAGCTATACACTATACCCTCGAGACAGATGAGGAAGAAGATTTGTTC 1020

KMP-5_16 (*r10C*) ACTGGCATCGGGAAAGCTATACACTATACCCTCGAGACAGATGAGGAAGAAGATTTGTTC 1020

KMP-5_18 (*r10A*) ACTGGCATCGGGAAAGCTATACACTATACCCTCGAGACAGATGAGGAAGAAGATTTGTTC 1020

KMP-7_3 (*r12C*) ACTGGCATCGGGAAAGCTATACACTATACCCTCGAGACAGATGAGGAAGAAGGTTTGTTC 861

KMP-7_1 (*r12A*) ACTGGCATCGGGAAAGCTATACACTATACCCTCGAGACAGATGAGGAAGAAGATTTGTTC 1020

KMP-7_4 (*r12D*) ACTGGCATCGGGAAAGCTATACACTATACCCTCGAGACAGATGAGGAAGAAGATTTGTTC 1009

KMP-6_7 (*r12A*) ACTGGCATCGGGAAAGCTATACACTATACCCTCGAGACAGATGAGGAAGAAGATTTGTTC 1020

KMP-7_2 (*r12B*) ACTGGCATCGGGAAAGCTATACACTATACCCTCGAGACAGATGAGGAAGAAGATTTGTTC 1017

KMP-6_3 (*r11A*) ACTGGCATCGGGAAAGCTATACACTATACCCTCGAGACAGATGAGGAAGAAGATTTGTTC 1020

KMP-6_5 (*r11B*) ACTGGCATCGGGAAAGCTATACACTATACCCTCGAGACAGATGAGGAAGAAGATTTGTTC 1020

KMP-6_8 (*r11B*) ACTGGCATCGGGAAAGCTATACACTATACCCTCGAGACAGATGAGGAAGAAGATTTGTTC 1020

KMP-8_3 (*s7*) ACTGGCATCGGGAAAGCTATACACTATACCCTCGAGACAGATGAGGAAGAAGATTTGTTC 1017

KMP-4_20 (*r8B*) ACTGGCATCGGGAAAGCTATACACTATACCCTCGAGACAGATGAGGAAGAAGATTTGTTC 1020

KMP-4_16 (*r8A*) ACTGGCATCGGGAAAGCTATACACTATACCCTCGAGACAGATGAGGAAGAAGATTTGTTC 1020

KMP-4_26 (*r8A*) ACTGGCATCGGGAAAGCTATACACTATACCCTCGAGACAGATGAGGAAGAAGATTTGTAC 1020

KMP-4_3 (*r8A*) ACTGGCATCGGGAAAGCTATACACTATACCCTCGAGACAGATGAGGAAGAAGATTTGTAC 1020

KMP-4_8 (*r9A*) ACTGGCATCGGGAAAGCTATACACTATACCCTCGAGACAGATGAGGAAGAAGATTTGTTC 1020

KMP-4_13 (*r9B*) ACTGGCATCGGGAAAGCTATACACTATACCCTCGAGACAGATGAGGAAGAAGATTTGTTC 1020

AY198374.1 ACTGGCATCGGGAAAGCTATACACTATACCCTCGAGACAGATGAGGAAGAAGATTTGTTC 1020

KMP-8_35 (*s6B*) ACTGGCATCGGGAAAGCTATACACTATACCCTCGAGACAGATGAGGA---AGATTTGTTC 1017

KMP-8_24 (*s6A*) ACTGGCATCGGGAAAGCTATACACTATACCCTCGAGACAGATGAGGAAGAAGATTTGTTC 1017

KMP-8_5 (*s6A*) ACGGGCATCGGGAAAGCTATACACTATGCCCTCGAGACAGATGAGGAAGAAGATTTGTTC 1017

KMP-8_46 (*s6A*) ACTGGCATCGGGAAAGCTATACACTATACCCTCGAGACAGATGAGGTAGAAGATTTGTTC 1017

** ************************.******************: **.*****:*

KMP-5_2 (*r10A*) TTCATCGAAACACTTCCGGGCGGCCATGACGGAGCCATCTTCAGCACTGCCATGATTGAT 1080

KMP-5_11 (*r10B*) TTCATCGAAACACTTCCGGGCGGCCATGACGGAGCCATCTTCAGCACTGCCATGATTGAT 1080

KMP-5_16 (*r10C*) TTCATCGAAACACTTCCGGGCGGCCATGACGGAGCCATCTTCAGCACTGCCATGATTGAT 1080

KMP-5_18 (*r10A*) TTCATCGAAACACTTCCGGGCGGCCATGACGGAGCCATCTTCAGCACTGCCATGATTGAT 1080

KMP-7_3 (*r12C*) TTCATCGAAACACTTCCGGGCGGCCATGACGGAGCCATCTTCAGCACTGCCATGATTGAT 921

KMP-7_1 (*r12A*) TTCATCGAAACACTTCCGGGCGGCCATGACGGAGCCATCTTCAGCACTGCCATGATTGAT 1080

KMP-7_4 (*r12D*) TTCATCGAAACACTTCCGGGCGGCCATGACGGAGCCATCTTCAGCACTGCCATGATTGAT 1069

KMP-6_7 (*r12A*) TTCATCGAAACACTTCCGGGTGGCCATGACGGAGCCATCTTCAGCACTGCCATGATTGAT 1080

KMP-7_2 (*r12B*) TTCATCGAAACACTTCCGGGCGGCCATGACGGAGCCATCTTCAGCACTGCCATGATTGAT 1077

KMP-6_3 (*r11A*) TTCATCGAAACACTTCCGGGCGGCCATGACGGAGCCATCTTCAGCACTGCCATGATTGAT 1080

KMP-6_5 (*r11B*) TTCATCGAAACACTTCCGGGCGGCCATGACGGAGCCATCTTCAGCACTGCCATGATTGAT 1080

KMP-6_8 (*r11B*) TTCATCGAAACACTTCCGGGCGGCCATGACGGAGCCATCTTCAGCACTGCCATGATTGAT 1080

KMP-8_3 (*s7*) TTCATCGAAACACTTCCGGGCGGCCATGACGGAGCCATCTTCAGCACTGCCATGATTGAT 1077

KMP-4_20 (*r8B*) TTCATCGAAACACTTCCGGGCGGCCATGACGGAGCCATCTTCAGCACTGCCATGTTTGAT 1080

KMP-4_16 (*r8A*) TTCATCGAAACACTTCCGGGCGGCCATGACGGAGCCATCTTCAGCACTGCCATGATTGAT 1080

KMP-4_26 (*r8A*) TTCATCGAAACACTTCCGGGCGGCCATGACGGAGCCATCTTCAGCACTGCCATGATTGAT 1080

KMP-4_3 (*r8A*) TTCATCGAAACACTTCCGGGCGGCCATGACGGAGCCATCTTCAGCACTGCCATGATTGAT 1080

KMP-4_8 (*r9A*) TTCATCGAAACACTTCCGGGCGGCCATGACGGAGCCATCTTCAGCACTGCCACGATTGAT 1080

KMP-4_13 (*r9B*) TTCATCGAAACACTTCCGGGCGGCCATGACGGAGCCATCTTCAGCACTGCCATGATTGAT 1080

AY198374.1 TTCATCGAAACACTTCCGGGCGGCCATGACGGAGCCATCTTCAGCACTGCCATGATTGAT 1080

KMP-8_35 (*s6B*) TTCATCGAAACACTTCCGGGCGGCCATGACGGAGCCATCTTCAGCACTGCCATGATTGAT 1077

KMP-8_24 (*s6A*) TTCATCGAAACACTTCCGGGCGGCCATGACGGAGCCATCTTCAGCACTGCCATGATTGAT 1077

KMP-8_5 (*s6A*) TTCATCGAGACACTTCCGGGCGGCCATGACGGAGCCATCTTCAGCACTGCCATGATTGAT 1077

KMP-8_46 (*s6A*) TTCATCGAAACACTTCCGGGCGGCCATGACGGAGCCATCTTCAGCACTGCCATGATTGAT 1077

********.*********** ******************************* *:*****

KMP-5_2 (*r10A*) GTGGATAGGCTCCGGCGAGATGTCTTCAGACTGTCCCTGGTGGCATACAAGTACGACAAT 1140

KMP-5_11 (*r10B*) GTGGATAGGCTCCGGCGAGATGTCTTCAGACTGTCCCTGGTGGCATACAAGTACGACAAT 1140

KMP-5_16 (*r10C*) GTGGATAGGCTCCGGCGAGATGTCTTCAGACTGCCCCTGGTGGCATACAAGTACGACAAT 1140

KMP-5_18 (*r10A*) GTGGATAGGCTCCGGCGAGATGTCTTCAGACTGTCCCTGGTGGCATACAAGTACGACAAT 1140

KMP-7_3 (*r12C*) GTGGATAGGCTCCGGCGAGATGTCTTCAGACTGTCCCTGGTGGCATACAAGTACGACAAT 981

KMP-7_1 (*r12A*) GTGGATAGGCTCCGGCGAGATGTCTTCAGACTGTCCCTGGTGGCATACAAGTACGACAAT 1140

KMP-7_4 (*r12D*) GTGGATAGGCTCCGGCGAGATGTCTTCAGACTGCCCCTGGTGGCATACAAGTACGACAAT 1129

KMP-6_7 (*r12A*) GTGGATAGGCTCCGGCGAGATGTCTTCAGACTGTCCCTGGTGGCATACAAGTACGACAAT 1140

KMP-7_2 (*r12B*) GTGGATAGGCTCCGGCGAGATGTCTTCAGACTGTCCCTGGTGGCATACAAGTACGACAAT 1137

KMP-6_3 (*r11A*) GTGGATAGGCTCCGGCGAGATGTCTTCAGACTGTCCCTGGTGGCATACAAGTACGACAAT 1140

KMP-6_5 (*r11B*) GTGGATAGGCTCCGGCGAGATGTCTTCAGACTGTCCCTGGTGGCATACAAGTACGACAAT 1140

KMP-6_8 (*r11B*) GTGGATAGGCTCCGGCGAGATGTCTTCAGACTGTCCCTGGTGGCATACAAGTACGACAAT 1140

KMP-8_3 (*s7*) GTGGATAGGCTCCGGCGAGATGTCTTCAGACTGTCCCTGGTGGCATACAAGTACGACAAT 1137

KMP-4_20 (*r8B*) GTGGATAGGCTCCGGCGAGATGTCTTCAGACTGTCCCTGGTGGCATACAAGTACGACAAT 1140

KMP-4_16 (*r8A*) GTGGATAGGCTCCGGCGAGATGTCTTTAGACTATCCCTGGTGGCATACAAGTACGACAAT 1140

KMP-4_26 (*r8A*) GTGGATAGGCTCCGGCGAGATGTCTTCAGACTATCCCTGGTGGCATACAAGTACGACAAT 1140

KMP-4_3 (*r8A*) GTGGATAGGCTCCGGCGAGATGTCTTCAGACTATCCCTGGTGGCATACAAGTACGACAAT 1140

KMP-4_8 (*r9A*) GGGGATAGGCTCCGGCGAGATGTCTTCAGACTGTCCCTGGTGGCATACAAGTACGACAAT 1140

KMP-4_13 (*r9B*) GTGGATAGGCTCCGGCGAGATGTCTTCAGACTGTCCCTGGTGGCATACAAGTACGACAAT 1140

AY198374.1 GTGGATAGGCTCCGGCGAGATGTCTTCAGACTGTCCCTGGTGGCATACAAGTACGACAAT 1140

KMP-8_35 (*s6B*) GTGGATAGGCTCCGGCGAGATGTCTTCAGACTGTCCCTGGTGGCATACAAGTACGACAAT 1137

KMP-8_24 (*s6A*) GTGGATAGGCTCCGGCGAGATGTCTTCAGACTGTCCCTGGTGGCATACAAGTACGACAAT 1137

KMP-8_5 (*s6A*) GTGGATAGGCTCCGGCGAGATGTCTTCAGACTGTCCCTGGTGGCATACAAGTACGACAAC 1137

KMP-8_46 (*s6A*) GTGGATAGGCTCCGGCGAGATGTCTTCAGACTGTCCCTGGTGGCATACAAGTACGACAAT 1137

* ************************ *****. *************************

KMP-5_2 (*r10A*) GTGTCCTTCGCCACCCCGACACCCGTCGTGATCATAGTCAATGACATCAACAACAAGAAA 1200

KMP-5_11 (*r10B*) GTGTCCTTCGCCACCCCGACACCCGTCGTGATCATAGTCAATGACATCAACAACAAGAAA 1200

KMP-5_16 (*r10C*) GTGTCCTTCGCCACCCCGACACCCGTCGTGATCATAGTCAATGACATCAACAACAAGAAA 1200

KMP-5_18 (*r10A*) GTGTCCTTCGCCACCCCGACACCCGTCGTGATCATAGTCAATGACATCAACAACAAGAAA 1200

KMP-7_3 (*r12C*) GTGTCCTTCGCCACCCCGACACCCGTCGTGATCATAGTCAACGACATCAACAACAAGCAA 1041

KMP-7_1 (*r12A*) GTGTCCTTCGCCACCCCGACACCCGTCGTGATCATAGTCAACGACATCAACAACAAGCAA 1200

KMP-7_4 (*r12D*) GTGTCCTTCGCCACCCCGACACCCGTCGTGATCATAGTCAACGACATCAACAACAAGCAA 1189

KMP-6_7 (*r12A*) GTGTCCTTCGCCACCCCGACACCCGTCGTGATCATAGTCAACGACATCAACAACAAGCAA 1200

KMP-7_2 (*r12B*) GTGTCCTTCGCCACCCCGACACCCGTCGTGATCATAGTCAACGACATCAACAACAAGCAA 1197

KMP-6_3 (*r11A*) GTGTCCTTCGCCACCCCGACACCCGTCGTGATCATAGTCAACGACATCAACAACAAGCAA 1200

KMP-6_5 (*r11B*) GTGTCCTTCGCCACCCCGACACCCGTCGTGATCATAGTTAACGACATCAACAACAAGAAA 1200

KMP-6_8 (*r11B*) GTGTCCTTCGCCACCCCGACACCCGTCGTGATCATAGTTAACGACATCAACAACAAGAAA 1200

KMP-8_3 (*s7*) GTGTCCTTCGCCACCCCGACACCCGTCGTGATCATAGTCAACGACATCAACAACAAGCAA 1197

KMP-4_20 (*r8B*) GTGTCCTTCGCCACCCCGACACCCGTCGTGATCATAGTCAATGACATCAACAACAAGAAA 1200

KMP-4_16 (*r8A*) GTGTCCTTCGCCACCCCGACACCCGTCGTGATCATAGTCAATGACATCAACAACAAGAAA 1200

KMP-4_26 (*r8A*) GTGTCCTTCGCCACCCCGACACCCGTCGTGATCATAGTCAATGACATCAACAACAAGAAA 1200

KMP-4_3 (*r8A*) GTGTCCTTCGCCACCCCGACACCCGTCGTGATCATAGTCAATGACATCAACAACAAGAAA 1200

KMP-4_8 (*r9A*) GTGTCCTTCGCCACCCCGACACCCGTCGTGATCATAGTCAATGACATCAACAACAAGAAA 1200

KMP-4_13 (*r9B*) GTGTCCTTCGCCACCCCGACACCCGTCGTGATCATAGTCAATGACATCAACAACAAGAAA 1200

AY198374.1 GTGTCCTTCGCCACCCCGACACCCGTCGTGATCATAGTCAATGACATCAACAACAAGAAA 1200

KMP-8_35 (*s6B*) GTGTCCTTCGCCACCCCGACACCCGTCGTGATCATAGTTAACGACATCAACAACAAGAAA 1197

KMP-8_24 (*s6A*) GTGTCCTTCGCCGCCCCGACACCCGTCGTGATCATAGTTAACGACATCAACAACAAGAAA 1197

KMP-8_5 (*s6A*) GTGTCCTTCGCCACCCCGACACCCGTCGTGATCATAGTCAACGACATCAACAACAAGAAA 1197

KMP-8_46 (*s6A*) GTGTCCTTCGCCACCCCGACACCCGTCGTGATCATAGTTAACGACATCAACAACAAGAAA 1197

************.************************* ** ***************.**

KMP-5_2 (*r10A*) CCCCAACCGCTGCAAGATGAGTACACAATCTCCATAATGGAAGAAACTCCACTGTCGCTG 1260

KMP-5_11 (*r10B*) CCCCAACCGCTGCAAGATGAGTACACAATCTCCATAATGGAAGAAACTCCACTGTCGCTG 1260

KMP-5_16 (*r10C*) CCCCAACCGCTGCAAGATGAGTACACAATCTCCATAATGGAAGAAACTCCACTGTCGCTG 1260

KMP-5_18 (*r10A*) CCCCAACCGCTGCAAGATGAGTACACAATCTCCATAATGGAAGAAACTCCACTGTCGCTG 1260

KMP-7_3 (*r12C*) CCCCAACCGCTGCAAGATGAGTACACAATCTCCATAATGGAAGAAACTCCACTGTCGCTG 1101

KMP-7_1 (*r12A*) CCCCAACCGCTGCAAGATGAGTGCACAATCTCCATAATGGAAGAAACTCCACTGTCGCTG 1260

KMP-7_4 (*r12D*) CCCCAACCGCTGCAAGATGAGTACACAATCTCCATAATGGAAGGAACTCCACTGTCGCTG 1249

KMP-6_7 (*r12A*) CCTCAACCGCTGCAAGATGAGTACACAATCTCCATAATGGAAGAAACTCCACTGTCGCTG 1260

KMP-7_2 (*r12B*) CCCCAACCGCTGCAAGATGAGTACACAATCTCCATAATGGAGGAAACTCCACTGTCGCTG 1257

KMP-6_3 (*r11A*) CCCCAACCGCTGCAAGATGAGTACACAATCTCCATAATGGAAGAAACTCCACTGTCGCTG 1260

KMP-6_5 (*r11B*) CCCCAACCGCTGCAAGATGAGTACACAATCTCCATAATGGAAGAAACTCCACTGTCGCTG 1260

KMP-6_8 (*r11B*) CCCCAACCGCTGCAAGATGAGTACACAATCTCCATAATGGAAGAAACTCCACTGTCGCTG 1260

KMP-8_3 (*s7*) CCCCAACCGCTGCAAGATGAGTACACAATCTCCATAATGGAAGAAACTCCACTGTCGCTG 1257

KMP-4_20 (*r8B*) CCCCAACCGCTGCAAGATGAGTACACAATCTCCATAATGGAAGAAACTCCACTGTCGCTG 1260

KMP-4_16 (*r8A*) CCCCAACCGCTGCAAGATGAGTACACAATCTCCATAATGGAAGAAACTCCACTGTCGCTG 1260

KMP-4_26 (*r8A*) CCCCAACCGCTGCAAGATGAGTACACAATCTCCATAATGGAAGAAACTCCACTGTCGCTG 1260

KMP-4_3 (*r8A*) CCCCAACCGCTGCAAGATGAGTACACAATCTCCATAATGGAAGAAACTCCACTGTCGCTG 1260

KMP-4_8 (*r9A*) CCCCAACCGCTGCAAGATGAGTACACAATCTCCATAATGGAAGAAACTCCACTGTCGCTG 1260

KMP-4_13 (*r9B*) CCCCAACCGTTGCAAGATGAGTACATAATCTCCATAATGGAAGAAACTCCACTGTCGCTG 1260

AY198374.1 CCCCAACCGCTGCAAGATGAGTACACAATCTCCATAATGGAAGAAACTCCACTGTCGCTG 1260

KMP-8_35 (*s6B*) CCCCAACCGCTGCAAGATGAGTACACAAACTCCATAATGGAAGAAACTCCACTGTCGCTG 1257

KMP-8_24 (*s6A*) CCCCAACCGCTGCAAGATGAGTACACAATCTCCATAATGGAAGAAACTCCACTGTCGCTG 1257

KMP-8_5 (*s6A*) CCCCAACCGCTGCAAGATGAGTACACAATCTCCATAATGGAAGAGACTCCACTGTCGCTG 1257

KMP-8_46 (*s6A*) CCCCAACCGCTGCAAGATGAGTACACAATCTCCATAATGGAAGAAACTCCACTGTCGCTG 1257

** ****** ************.** **:************.*..***************

KMP-5_2 (*r10A*) AATTTTGCTGAACTTTTTGGTTTCTATGATGAAGATTTGATCTACGCACAATTCTTGGTG 1320

KMP-5_11 (*r10B*) AATTTTGCTGAACTTTTTGGTTTCTATGATGAAGATTTGATCTACGCACAATTCTTGGTG 1320

KMP-5_16 (*r10C*) AATTTTGCTGAACTTTTTGGTTTCTATGATGAAGATTTGATCTACGCACAATTCTTGGTG 1320

KMP-5_18 (*r10A*) AATTTTGCTGAACTTTTTGGTTTCTATGATGAAGATTTGATCTACGCACAATTCTTGGTG 1320

KMP-7_3 (*r12C*) AATTTTGCTGAACTTTTTGGTTTCTATGATGAAGATTTGATCTACGCACAATTCTTGGTG 1161

KMP-7_1 (*r12A*) AATTTTGCTGAACTTTTTGGTTTCTATGATGAAGATTTGATCTACGCACAATTCTTGGTG 1320

KMP-7_4 (*r12D*) AATTTTGCTGAACTTTTTGGTTTCTATGATGAAGATTTGATCTACGCACGATTCTTGGTG 1309

KMP-6_7 (*r12A*) GATTTTGCTGAACTTTTTGGTTTCTATGATGAAGATTTGATCTACGCACAATTCTTGGTG 1320

KMP-7_2 (*r12B*) AATTTTGCTGAACTTTTTGGTTTCTATGATGAAGATTTGATCTACGCACAATTCTTGGTG 1317

KMP-6_3 (*r11A*) AATTTTGCTGAACTTTTTGGTTTCTATGATGAAGATTTGATCTACGCACAATTCTTGGTG 1320

KMP-6_5 (*r11B*) AATTTTGCTGAACTTTTTGGTTTCTATGATGAAGATTTGATCTACGCACAATTCTTGGTG 1320

KMP-6_8 (*r11B*) AATTTTGCTGAACTTTTTGGTTTCTATGATGAAGATTTGATCTACGCACAATTCTTGGTG 1320

KMP-8_3 (*s7*) AATTTTGCTGAACTTTTTGGTTTCTATGATGAAGATTTGATCTACGCACAATTCTTGGTG 1317

KMP-4_20 (*r8B*) AATTTTGCTGAACTTTTTGGTTTCTATGATGAAGATTTGATCTACGCACAATTCTTGGTG 1320

KMP-4_16 (*r8A*) AATTTTGCTGAACTTTTTGGTTTCTATGATGAAGATTTGATCTACGCACAATTCTTGGTG 1320

KMP-4_26 (*r8A*) AATTTTGCTGAACTTTTTGGTTTCTATGATGAAGATTTGATCTACGCACAATTCTTGGTG 1320

KMP-4_3 (*r8A*) AATTTTGTTGAACTTTTTGGTTTCTATGATGAAGATTTGATCTACGCACAATTCTTGGTG 1320

KMP-4_8 (*r9A*) AATTTTGCTGAACTTTTTGGTTTCTATGATGAAGATTTGATCTACGCACAATTCCTGGTG 1320

KMP-4_13 (*r9B*) AATTTTGCTGAACTTTTTGGTTTCTATGATGAAGATTTGATCTACGCACAATTCTTGGTG 1320

AY198374.1 AATTTTGCTGAACTTTTTGGTTTCTATGATGAAGATTTGATCTACGCACAATTCTTGGTG 1320

KMP-8_35 (*s6B*) AATTTTGCTGAACTTTTTGGTTTCTATGATGAAGATTTGATCTACGCACAATTCTTGGTG 1317

KMP-8_24 (*s6A*) AATTTTGCTGAACTTTTTAGTTTCTATGATGAAGATTTGATCTACGCACGATTCTTGGTG 1317

KMP-8_5 (*s6A*) AATTTTGCTGAACTTTTTGGTTTCTATGATGAAGATTTGATCTACGCACAATTCTTGGTG 1317

KMP-8_46 (*s6A*) AATTTTGCTGAACTTTTTGGTTTCTATGATGAAGATTTGATCTACGCACAATTCTTGGTG 1317

.****** **********.******************************.**** *****

KMP-5_2 (*r10A*) GAAATACAAGGCGAGAACCCTCCAGGCGTAGAGCAAGCGTTTTATATTGCGCCCACCGCA 1380

KMP-5_11 (*r10B*) GAAATACAAGGCGAGAACCCTCCAGGCGTGGAGCAAGCGTTTTATATTGCGCCCACCGCA 1380

KMP-5_16 (*r10C*) GAAATACAAGGCGAGAACCCTCCAGGCGTAGAGCAAGCGTTTTATATTGCGCCCACCGCA 1380

KMP-5_18 (*r10A*) GAAATACAAGGCGAGAACCCTCCAGGCGTAGAGCAAGCGTTTTATATTGCGCCCACCGCA 1380

KMP-7_3 (*r12C*) GAAGTACAAGGCGAGAACCCTCCAGGCGTAGAGCAAGCGTTTTATATTGCGCCCACCGCA 1221

KMP-7_1 (*r12A*) GAAATACAAGGCGAGAACCCTCCAGGCGTAGAGCAAGCGTTTTATATTGCGCCCACCGCA 1380

KMP-7_4 (*r12D*) GAAATACAAGGCGAGAACCCTCCAGGCGTAGAGCAAGCGTTTTATATTGCGCCCACCGCA 1369

KMP-6_7 (*r12A*) GAAATACAAGGCGAGAACCCTCCAGGCGTAGAGCAAGCGTTTTATATTGCGCCCACCGCA 1380

KMP-7_2 (*r12B*) GAAATACAAGGCGAGAACCCTCCAGGCGTAGAGCAAGCGTTTTATATTGCGCCCACCGCA 1377

KMP-6_3 (*r11A*) GAAATACAAGGCGAGAACCCTCCAGGCGTAGAGCAAGCGTTTTATATTGCGCCCACCGCA 1380

KMP-6_5 (*r11B*) GAAATACAAGGCGAGAACCCTCCAGGCGTAGAGCAAGCGTTTTATATTGCGCCCACCGCA 1380

KMP-6_8 (*r11B*) GAAATACAAGGCGAGAACCCTCCAGGCGTAGAGCAAGCGTTTTATATTGCGCCCACCGCA 1380

KMP-8_3 (*s7*) GAAATACAAGGCGAGAACCCTCCAGGCGTAGAGCAAGCGTTTTATATTGCGCCCACCGCA 1377

KMP-4_20 (*r8B*) GAAATACAAGGCGAGAACCCTCCAGGCGTAGGGCAAGCGTTTTATATTGCGCCCACCGCA 1380

KMP-4_16 (*r8A*) GAAATACAAGGCGAGAACCCTCCAGGCGTAGAGCAAGCGTTTTATATTGCGCCCACCGCA 1380

KMP-4_26 (*r8A*) GAAATACAAGGCGAGAACCCTCCAGGCGTAGAGCAAGCGTTTTATATTGCGCCCACCGCA 1380

KMP-4_3 (*r8A*) GAAATACAAGGCGAGAACCCTCCAGGCGCAGAGCAAGCGTTTTATATTGCGCCCACCGCA 1380

KMP-4_8 (*r9A*) GAAATACAAGGCGAGAACCCTCCAGGCGTAGAGCAAGCGTTTTATATTGCGCCCACCGCA 1380

KMP-4_13 (*r9B*) GAAATACAAGGCGAGAACCCTCCAGGCGTAGAGCAAGCGTTTTATATTGCGCCCACCGCA 1380

AY198374.1 GAAATACAAGGCGAGAACCCTCCAGGCGTAGAGCAAGCGTTTTATATTGCGCCCACCGCA 1380

KMP-8_35 (*s6B*) GAAATACAAGGCGAGAACCCTCCAGGCGTAGAGCAAGCGTTTTATATTGCGCCCACCGCA 1377

KMP-8_24 (*s6A*) GGAATACAAGGCGAGAACCCTCCAGGCGTAGAGCAAGCGTTTTATATTGCGCCCACCGCA 1377

KMP-8_5 (*s6A*) GAAATACAAGGCGAGAACCCTCCAGGCGTAGAGCAAGCGTTTTATATTGCGCCCACCGCA 1377

KMP-8_46 (*s6A*) GAAATACAAGGCGAGGACCCTCCAGGCGTAGAGCAAGCGTTTTATATTGCGCCCACCGCA 1377

*.*.***********.************ .*.****************************

KMP-5_2 (*r10A*) GGCTTCCAGAACCAGACATTCGCCATAGGGACTCAAGATCACCGAATGCTGGATTATGAG 1440

KMP-5_11 (*r10B*) GGCTTCCAGAACCAGACATTCGCCATAGGGACTCAAGATCACCGAATGCTGGATTATGAG 1440

KMP-5_16 (*r10C*) GGCTTCCAGAACCAGACATTCGCCATAGGGACTCAAGATCACCGAATGCTGGATTATGAG 1440

KMP-5_18 (*r10A*) GGCTTCCAGAACCAGACATTCGCCATAGGGACTCAAGATCACCGAATGCTGGATTATGAG 1440

KMP-7_3 (*r12C*) GGCTTCCAGAACCAGACATTCGCCATAGGGACTCAAGATCACCGAATGCTGGATTATGAG 1281

KMP-7_1 (*r12A*) GGCTTCCAGAACCAGACATTCGCCATAGGGACTCAAGATCACCGAATGCTGGATTATGAG 1440

KMP-7_4 (*r12D*) GGCTTCCAGAACCAGACATTCGCCATAGGGACTCAAGATCACCGAATGCTGGATTATGAG 1429

KMP-6_7 (*r12A*) GGCTTCCAGAACCAGACATTCGCCATAGGGACTCAAGATCACCGAATGCTGGATTATGAG 1440

KMP-7_2 (*r12B*) GGCTTCCAGAACCAGACATTCGCCATAGGGACTCAAGATCACCGAATGCTGGATTATGAG 1437

KMP-6_3 (*r11A*) GGCTTCCAGAACCAGACATTCGCCATAGGGACTCAAGATCACCGAATGCTGGATTATGAG 1440

KMP-6_5 (*r11B*) GGCTTCCAGAACCAGACATTCGCCATAGGGACTCAAGATCACCGAATGCTGGATTATGAG 1440

KMP-6_8 (*r11B*) GGCTTCCAGAACCAGACATTCGCCATAGGGACTCAAGATCACCGAATGCTGGATTATGAG 1440

KMP-8_3 (*s7*) GGCTTCCAGAACCAGACATTCGCCATAGGGACTCAAGATCACCGAATGCTGGATTATGAG 1437

KMP-4_20 (*r8B*) GGCTTCCAGAACCAGACATTCGCCATAGGGACTCAAGATCACCGAATGCTGGATTATGAG 1440

KMP-4_16 (*r8A*) GGCTTCCAGAACCAGACATTCGCCATAGGGACTCAAGATCACCGAATGCAGGATTATGAG 1440

KMP-4_26 (*r8A*) GGCTTCCAGAACCAGACATTCGCCATAGGGACTCAAGATCACCGAATGCTGGATTATGAG 1440

KMP-4_3 (*r8A*) GGCTTCCAGAACCAGACATTCGCCATAGGGACTCAAGATCACCGAATGCTGGATTATGAG 1440

KMP-4_8 (*r9A*) GGCTTCCAGAACCAGACATTCGCCATAGGGACTCAAGATCACCGAATGCTGGATTATGAG 1440

KMP-4_13 (*r9B*) GGCTTCCAGAACCAGACATTCGCCATAGGGACTCAAGATCACCGAATGCTGGATTATGAG 1440

AY198374.1 GGCTTCCAGAACCAGACATTCGCCATAGGGACTCAAGATCACCGAATGCTGGATTATGAG 1440

KMP-8_35 (*s6B*) GGCTTCCAGAACCAGACATTCGCCATAGGGACTCAAGATCACCGAATGCTGGATTATGAG 1437

KMP-8_24 (*s6A*) GGCTTCCAGAACCAGACATTCGCCATAGGGACTCAAGATCACCGAATGCTGGATTATGAG 1437

KMP-8_5 (*s6A*) GGCTTCCAGAACCAGACATTCGCCATAGGGACTCAAGATCACCGAATGCTGGATTATGAG 1437

KMP-8_46 (*s6A*) GGCTTCCAGAACCAGACATTCGCCATAGGGACTCAAGATCACCGAATGCTGGATTATGAG 1437

*************************************************:**********

KMP-5_2 (*r10A*) GATGTTCCTTTCCAAAACATCAAGCTCAAGGTAATAGCAACGGACCGTGACAATACCAAT 1500

KMP-5_11 (*r10B*) GATGTTCCTTTCCAAAACATCAAGCTCAAGGTAATAGCAACGGACCGTGACAATACCAAT 1500

KMP-5_16 (*r10C*) GATGTTCCTTTCCAAAACATCAAGCTCAAGGTAATAGCAACGGACCGTGACAATACCAAT 1500

KMP-5_18 (*r10A*) GATGTTCCTTTCCAAAACATCAAGCTCAAGGTAATAGCAACGGACCGTGACAATACCAAT 1500

KMP-7_3 (*r12C*) GATGTTCCTTTCCAAAACATCAAGCTCAAGGTAATAGCAACGGACCGTGACAATACCAAT 1341

KMP-7_1 (*r12A*) GATGTTCCTTTCCAAAACATCAAGCTCAAGGTAATAGCAACGGACCGTGACAATACCAAT 1500

KMP-7_4 (*r12D*) GATGTTCCTTTCCAAAACATCAAGCTCAAGGTAATAGCAACGGACCGTGACAATACCAAT 1489

KMP-6_7 (*r12A*) GATGTTCCTTTCCAAAACATCAAGCTCAAGGTAATAGCAACGGACCGTGACAATACCAAT 1500

KMP-7_2 (*r12B*) GATGTTCCTTTCCAAAACATCAAGCTCAAGGTAATAGCAACGGACCGTGACAATACCAAT 1497

KMP-6_3 (*r11A*) GATGTTCCTTTCCAAAACATCAAGCTCAAGGTAATAGCAACGGACCGTGACAATACCAAT 1500

KMP-6_5 (*r11B*) GATGTTCCTTTCCAAAACATCAAGCTCAAGGTAATAGCAACGGACCGTGACAATACCAAT 1500

KMP-6_8 (*r11B*) GATGTTCCTTTCCAAAACATCAAGCTCAAGGTAATAGCAACGGACCGTGACAATACCAAT 1500

KMP-8_3 (*s7*) GATGTTCCTTTCCAAAACATCAAGCTCAAGGTAATAGCAACGGACCGTGACAATACCAAT 1497

KMP-4_20 (*r8B*) GATGTTCCTTTCCAAAACATCAAGCTCAAGGTAATAGCAACGGACCGTGACAATACCAAT 1500

KMP-4_16 (*r8A*) GATGTTCCTTTCCAAAACATCAAGCTCAAGGTAATAGCAACGGACCGTGACAATACCAAT 1500

KMP-4_26 (*r8A*) GATGTTCCTTTCCAAAACATCAAGCTCAAGGTAATAGCAACGGACCGTGACAATACCAAT 1500

KMP-4_3 (*r8A*) GATGTTCCTTTCCAAAACATCAAGCTCAAGGTAATAGCAACGGACCGTGACAATACCAAT 1500

KMP-4_8 (*r9A*) GATGTTCCTTTCCAAAACATCAAGCTCAAGGTAATAGCAACGGACCGTGACAATACCAAT 1500

KMP-4_13 (*r9B*) GATGTTCCTTTCCAAAACATCAAGCTCAAGGTAATAGCAACGGACCGTGACAATACCAAT 1500

AY198374.1 GATGTTCCTTTCCAAAACATCAAGCTCAAGGTAATAGCAACGGACCGTGACAATACCAAT 1500

KMP-8_35 (*s6B*) GATGTTCCTTTCCAAAACATCAAGCTCAAGGTAATAGCAACGGACCGTGACAATACCAAT 1497

KMP-8_24 (*s6A*) GATGTTCCTTTCCAAAACATCAAGCTCAAGGTAATAGCAACGGACCGTGACAATACCAAT 1497

KMP-8_5 (*s6A*) GATGTTCCTTTCCAAAACATCAAGCTCAAGGTAATAGCAACGGACCGTGACAATACCAAT 1497

KMP-8_46 (*s6A*) GATGTTCCTTTCCAAAACATCAAGCTCAAGGTAATAGCAACGGACCGTGACAATACCAAT 1497

************************************************************

KMP-5_2 (*r10A*) TTTACTGGAGTCGCGGAAGTCAACGTGAACCTGATTAATTGGAACGACGAGGAGCCGATC 1560

KMP-5_11 (*r10B*) TTTACTGGAGTCGCGGAAGTCAACGTGAACCTGATTAATTGGAACGACGAGGAGCCGATC 1560

KMP-5_16 (*r10C*) TTTACTGGAGTCGCGGAAGTCAACGTGAACCTGATTAATTGGAACGACGAGGAGCCGATC 1560

KMP-5_18 (*r10A*) TTTACTGGAGTCGCGGAAGTCAACGTGAACCTGATTAATTGGAACGACGAGGAGCCGATC 1560

KMP-7_3 (*r12C*) TTTACTGGAGTCGCGGAAGTCAACGTGAACCTGATTAATTGGAACGACGAGGAGCCGATC 1401

KMP-7_1 (*r12A*) TTTACTGGAGTCGCGGAAGTCAACGTGAACCTGATTAATTGGAACGACGAGGAGCCGATC 1560

KMP-7_4 (*r12D*) TTTACTGGAGTCGCGGAAGTCAACGTGAACCTGATTAATTGGAACGACGAGGAGCCGATC 1549

KMP-6_7 (*r12A*) TTTACTGGAGTCGCGGAAGTCAACGTGAACCTGATTAATTGGAACGACGAGGAGCCGATC 1560

KMP-7_2 (*r12B*) TTTACTGGAGTCGCGGAAGTCAACGTGAACCTGATTAATTGGAACGACGAGGAGCCGATC 1557

KMP-6_3 (*r11A*) TTTACTGGAGTCGCGGAAGTCAACGTGAACCTGATTAATTGGAACGACGAGGAGCCGATC 1560

KMP-6_5 (*r11B*) TTTACTGGAGTCGCGGAAGTCAACGTGAATCTGATTAATTGGAACGACGAGGAGCCGATC 1560

KMP-6_8 (*r11B*) TTTACTGGAGTCGCGGAAGTCAACGTGAATCTGATTAATTGGAACGACGAGGAGCCGATC 1560

KMP-8_3 (*s7*) TTTACTGGAGTCGCGGAAGCCAACGTGATTCTGATTAATTGGAACGACGAGGAGCCGATC 1557

KMP-4_20 (*r8B*) TTTACTGGAGTCGCGGAAGTCAACGTGAACCTGATTAATTGGAACGACGAGGAGCCGATC 1560

KMP-4_16 (*r8A*) TTTACTGGAGTCGCGGAAGTCAACGTGAACCTGATTAATTGGAACGACGAGGAGCCGATC 1560

KMP-4_26 (*r8A*) TTTACTGGAGTCGCGGAAGTCAACGTGAACCTGATTAATTGGAACGACGAGGAGCCGATC 1560

KMP-4_3 (*r8A*) TTTACTGGAGTCGCGGAAGTCAACGTGAACCTGATTAATTGGAACGACGAGGAGCCGATC 1560

KMP-4_8 (*r9A*) TTTACTGGAGTCGCGGAAGTCAACGTGAACCTGATTAATTGGAACGACGAGGAGCCGATC 1560

KMP-4_13 (*r9B*) TTTACTGGAGTCGCGGAAGTCAACGTGAACCTGATTAATTGGAACGACGAGGAGCCGATC 1560

AY198374.1 TTTACTGGAGTCGCGGAAGTCAACGTGAACCTGATTAATTGGAACGACGAGGAGCCGATC 1560

KMP-8_35 (*s6B*) TTTACTGGAGTCGCGGAAGTCAACGTGAATCTGATTAATTGGAACGACGAGGAGCCGATC 1557

KMP-8_24 (*s6A*) TTTACTGGAGTCGCGGAAGTCAACGTGAATCTGATTAATTGGAACGACGAGGAGCCGATC 1557

KMP-8_5 (*s6A*) TTTACTGGAGTCGCGGAAGTCAACGTGAATCTGATTAATTGGAACGACGAGGAGCCGATC 1557

KMP-8_46 (*s6A*) TTTACTGGAGTCGCGGAAGTCAACGTGAATCTGATTAATTGGAACGACGAGGAGCCGATC 1557

******************* ********: ******************************

KMP-5_2 (*r10A*) TTTGAGGAAGACCAGCTCGTTGTCAAGTTCAAGGAGACTGTACCCAAGGACTATCACGTC 1620

KMP-5_11 (*r10B*) TTTGAGGAAGACCAGCTCGTTGTCAAGTTCAAGGAGACTGTACCCAAGGACTATCACGTC 1620

KMP-5_16 (*r10C*) TTTGAGGAAGACCAGCTCGTTGTCAAGTTCAAGGAGACTGTACCCAAGGACTATCACGTC 1620

KMP-5_18 (*r10A*) TTTGAGGAAGACCAGCTCGTTGTCAAGTTCAAGGAGACTGTACCCAAGGACTATCACGTC 1620

KMP-7_3 (*r12C*) TTTGAGGAAGACCAGCTCGTTGTCAAGTTCAAGGAGACTGTACCCAAGGACTATCACGTC 1461

KMP-7_1 (*r12A*) TTTGAGGAAGACCAGCTCGTTGTCAAGTTCAAGGAGACTGTACCCAAGGACTATCACGTC 1620

KMP-7_4 (*r12D*) TTTGAGGAAGACCAGCTCGTTGTCAAGTTCAAGGAGACTGTACCCAAGGACTATCACGTC 1609

KMP-6_7 (*r12A*) TTTGAGGAAGACCAGCTCGTTGTCAAGTTCAAGGAGACTGTACCCAAGGACTATCACGTC 1620

KMP-7_2 (*r12B*) TTTGAGGAAGACCAGCTCGTTGTCAAGTTCAAGGAGACTGTACCCAAGGACTATCACGTC 1617

KMP-6_3 (*r11A*) TTTGAGGAAGACCAGCTCGTTGTCAAGTTCAAGGAGACTGTACCCAAGGACTATCACGTC 1620

KMP-6_5 (*r11B*) TTTGAGGAGGACCAGCTGGTTGTCAAGTTCAAGGAGACTGTACCCAAGGACTATCACGTC 1620

KMP-6_8 (*r11B*) TTTGAGGAGGACCAGCTGGTTGTCAAGTTCAAGGAGACTGTACCCAAGGACTATCACGTC 1620

KMP-8_3 (*s7*) TTTGAGGAGGACCAGCTGGTTGTCAAGTTCAAGGAGACTGTACCCAAGGACTATCACGTC 1617

KMP-4_20 (*r8B*) TTTGAGGAAGACCAGCTCGTTGTCAAGTTCAAGGAGACTGTACCCAAGGACTATCACGTC 1620

KMP-4_16 (*r8A*) TTTGAGGAAGACCAGCTCGTTGTCAAGTTCAAGGAGACTGTACCCAAGGACTATCACGTC 1620

KMP-4_26 (*r8A*) TTTGAGGAAGACCAGCTCGTTGTCAAGTTCAAGGAGACTGTACCCAAGGACTATCACGTC 1620

KMP-4_3 (*r8A*) TTTGAGGAAGACCAGCTCGTTGTCAAGTTCAAGGAGACTGTACCCAAGGACTATCACGTC 1620

KMP-4_8 (*r9A*) TTTGAGGAAGACCAGCTCGTTGTCAAGTTCAAGGAGACTGTACCCAAGGACTATCACGTC 1620

KMP-4_13 (*r9B*) TTTGAGGAAGACCAGCTCGTTGTCAAGTTCAAGGAGACTGTACCCAAGGACTATCACGTC 1620

AY198374.1 TTTGAGGAAGACCAGCTCGTTGTCAAGTTCAAGGAGACTGTACCCAAGGACTATCACGTC 1620

KMP-8_35 (*s6B*) TTTGAGGAGGACCAGCTGGTTGTCGAGTTCAAGGAGACTGTACCCAAGGACTATCACGTC 1617

KMP-8_24 (*s6A*) TTTGAGGAGGACCAGCTGGTTGTCAAGTTCAAGGAGACTGTACCCAAGGACTATCACGTC 1617

KMP-8_5 (*s6A*) TTTGAGGAGGACCAGCTGGTTGTCAAGTTCAAGGAGACTGTACCCAAGGACTATCACGTC 1617

KMP-8_46 (*s6A*) TTTGAGGAGGACCAGCTGGTTGTCAAGTTCAAGGAGACTGTACCCAAGGACTATCACGTC 1617

******** ******** ****** ***********************************

KMP-5_2 (*r10A*) GGCAGACTGAGGGCTCACGACCGGGACATAGGAGACAGCGTTGTGCATGCCATCTTGGGA 1680

KMP-5_11 (*r10B*) GGCAGACTGAGGGCTCACGACCGGGACATAGGAGACAGCGTTGTGCATTCCATCTTGGGA 1680

KMP-5_16 (*r10C*) GGCAGACTGAGGGCTCACGACCGGGACATAGGAGACAGCGTTGTGCATTCCATCTTGGGA 1680

KMP-5_18 (*r10A*) GGCAGACTGAGGGCTCACGACCGGGACATAGGAGACAGCGTTGTGCATTCCATCTTGGGA 1680

KMP-7_3 (*r12C*) GGCAGACTGAGGGCTCACGACCGGGACATAGGAGACAGCGTTGTGCATTCCATCTTGGGA 1521

KMP-7_1 (*r12A*) GGCAGACTGAGGGCTCACGACCGGGACATAGGAGACAGCGTTGTGCATTCCATCTTGGGA 1680

KMP-7_4 (*r12D*) GGCAGACTGAGGGCTCACGACCGGGACATAGGAGACAGCGTTGT---------------- 1653

KMP-6_7 (*r12A*) GGCAGACTGAGGGCTCACGACCGGGACATAGGAGACAGCGTTGTGCATTCCATCTTGGGA 1680

KMP-7_2 (*r12B*) GGCAGACTGAGGGCTCACGACCGGGACATAGGAGACAGCGTTGT---------------- 1661

KMP-6_3 (*r11A*) GGCAGACTGAGGGCTCACGACCGGGACATAGGAGACAGCGTTGTGCATTCCATCTTGGGA 1680

KMP-6_5 (*r11B*) GGCAGACTGAGGGCTCACGACCGGGACATAGGAGACAGCGTTGTGCATTCCATCTTGGGA 1680

KMP-6_8 (*r11B*) GGCAGACTGAGGGCTCACGACCGGGACATAGGAGACAGCGTTGTGCATTCCATCTTGGGA 1680

KMP-8_3 (*s7*) GGCAGACTGAGGGCTCACGACCGGGACATAGGAGACAGCGTTGTGCATTCCATCTTGGGA 1677

KMP-4_20 (*r8B*) GGCAGACTGAGGGCTCACGACCGGGACATAGGAGACAGCGTTGTGCATTCCATCTTGGGA 1680

KMP-4_16 (*r8A*) GGCAGACTGAGGGCTCACGACCGGGACATAGGAGACAGCGTTGTGCATTCCATCTTGGGA 1680

KMP-4_26 (*r8A*) GGCAGACTGAGGGCTCACGACCGGGACATAGGAGACAGCGTTGTGCATTCCATCTTGGGA 1680

KMP-4_3 (*r8A*) GGCAGACTGAGGGCTCACGACCGGGACATAGGAGACAGCGTTGTGCATTCCATCTTGGGA 1680

KMP-4_8 (*r9A*) GGCAGACTGAGGGCTCACGACCGGGACATAGGAGACAGCGTTGTGCATTCCATCTTGGGA 1680

KMP-4_13 (*r9B*) GGCAGACTGAGGGCTCACGACCGGGACATAGGAGACAGCGTTGTGCATTCCATCTTGGGA 1680

AY198374.1 GGCAGACTGAGGGCTCACGACCGGGACATAGGAGACAGCGTTGTGCATTCCATCTTGGGA 1680

KMP-8_35 (*s6B*) GGCAGACTGAGGGCTCACGACCGGGACATAGGAGACAGCGTTGTGCATTCTATCTTGGGA 1677

KMP-8_24 (*s6A*) GGCAGACTGAGGGCTCACGACCGGGACATAGGAGACAGCGTTGTGCATTCCATCTTGGGA 1677

KMP-8_5 (*s6A*) GGCAGACTGAGGGCTCACGACCGGGACATAGGAGACAGCGTTGTGCATTCCATCTTGGGA 1677

KMP-8_46 (*s6A*) GGCAGACTGAGGGCTCACGACCGGGACATAGGAGACAGCGTTGTGCATTCCATCTTGGGA 1677

********************************************

KMP-5_2 (*r10A*) AATGCGAATACATTTTTGAGAATCGACGAAGAAACTGGCGACATATACGTAGCTATTGAT 1740

KMP-5_11 (*r10B*) AATGCGAATACATTTTTGAGAATCGACGAAGAAACTGGCGACATATACGTAGCTATTGAT 1740

KMP-5_16 (*r10C*) AATGCGAATACATTTTTGAGAATCGACGAAGAAACTGGCGACATATACGTAGCTATTGAT 1740

KMP-5_18 (*r10A*) AATGCGAATACATTTTTGAGAATCGACGAAGAAACTGGCGACATATACGTAGCTATTGAT 1740

KMP-7_3 (*r12C*) AATGCGAATACATTTTTAAGAATCGACGAAGAAACTGGCGACATCTACGTAACTATTGAT 1581

KMP-7_1 (*r12A*) AATGCGAATACATTTTTAAGAATCGACGAAGAAACTGGCGACATCTACGTAACTATTGAT 1740

KMP-7_4 (*r12D*) ------------------------------------------------------------ 1653

KMP-6_7 (*r12A*) AATGCGAATACATTTTTAAGAATCGACGAAGAAACTGGCGACATCTACGTAACTATTGAT 1740

KMP-7_2 (*r12B*) ------------------------------------------------------------ 1661

KMP-6_3 (*r11A*) AATGCGAATACATTTTTAAGAATCGACGAAGAAACTGGCGACATCTACGTAACTATTGAT 1740

KMP-6_5 (*r11B*) AATGCGAATACATTTTTGAGAATCGACGAAGAAACTGGCGACATCTACGTAGCTATTGAT 1740

KMP-6_8 (*r11B*) AATGCGAATACATTTTTGAGAATCGACGAAGAAACTGGCGACATCTACGTAGCTATTGAT 1740

KMP-8_3 (*s7*) AATGCGAATACATTTTTGAGAATCGACGAAGAAACTGGCGACATCTACGTAGCTATTGAT 1737

KMP-4_20 (*r8B*) AATGCGAATACATTTTTGAGAATCGACGAAGAAACTGGCGACATATACGTAGCTATTGAT 1740

KMP-4_16 (*r8A*) AATGCGAATACATTTTTGAGAATCGACGAAGAAACTGGCGACATATACGTAGCTATTGAT 1740

KMP-4_26 (*r8A*) AATGCGAATACATTTTTGAGAATCGACGAAGAAACTGGCGACATATACGTAGCTATTGAT 1740

KMP-4_3 (*r8A*) AATGCGAATACATTTTTGAGAATCGACGAAGAAACTGGCGACATATACGTAGCTATTGAT 1740

KMP-4_8 (*r9A*) AATGCGAATACATTTTTGAGAATCGACGAAGAAACTGGCGACATATACGTAGCTATTGAT 1740

KMP-4_13 (*r9B*) AATGCGAATACATTTTTGAGAATCGACGAAGAAACTGGCGACATATACGTAGCTATTGAT 1740

AY198374.1 AATGCGAATACATTTTTGAGAATCGACGAAGAAACTGGCGACATATACGTAGCTATTGAT 1740

KMP-8_35 (*s6B*) AATGCGAATACATTTTTGAGAATCGACGAAGAAACTGGCGACATCTACGTAGCTATTGAT 1737

KMP-8_24 (*s6A*) AATGCGAATACATTTTTGAGAATCGACGAAGAAACTGGCGACATCTACGTAGCTATTGAT 1737

KMP-8_5 (*s6A*) AATGCGAATACATTTTTGAGAATCGACGAAGAAACTGGCGACATCTACGTAGCTATTGAT 1737

KMP-8_46 (*s6A*) AATGCGAATACATTTTTGAGAATCGACGAAGAAACTGGCGACATCTACGTAGCTATTGAT 1737

KMP-5_2 (*r10A*) GACGCGTTCGATTATCACAGACAGAATGAATTTAACA----TACAAGTTCGCGCTCAGGA 1796

KMP-5_11 (*r10B*) GACGCGTTCGATTATCACAGACAGAATGAATTTAACA----TACAAGTTCGCGCTCAGGA 1796

KMP-5_16 (*r10C*) GACGCGTTCGATTATCACAGACAGAATGAATTTAACA----TACAAGTTCGCGCTCAGGA 1796

KMP-5_18 (*r10A*) GACGCGTTCGATTATCACAGACAGAATGAATTTAACA----TACAAGTTCGCGCTCAGGA 1796

KMP-7_3 (*r12C*) GACGCGTTCGATTATCACAGACAGAATGAATTTAACA----TACAAGTTCGCGCTCAGGA 1637

KMP-7_1 (*r12A*) GACGCGTTCGATTATCACAGACAGAATGAATTTAACC----TACAAGTTCGCGCTCAGGA 1796

KMP-7_4 (*r12D*) ------------------------------------------------------------ 1653

KMP-6_7 (*r12A*) GACGCGTTCGATTATCACAGACAGAATGAATTTAACA----TACAAGTTCGCGCTCAGGA 1796

KMP-7_2 (*r12B*) ----------------------------------------------GTTCGCGCTCAGGA 1675

KMP-6_3 (*r11A*) GACGCGTTCGATTATCACAGACAGAATGAATTTAACA----TACAAGTTCGCGCTCAGGA 1796

KMP-6_5 (*r11B*) GACGCGTTCGATTATCACAGACAGAATGAATTTAACA----TACAAGTTCGCGCTCAGGA 1796

KMP-6_8 (*r11B*) GACGCGTTCGATTATCACAGACAGAATGAATTTAACA----TACAAGTTCGCGCTCAGGA 1796

KMP-8_3 (*s7*) GACGCGTTCGATTATCACAGACAGAATGAATTTAACA----TACAAGTTCGCGCTCAGGA 1793

KMP-4_20 (*r8B*) GACGCGTTCGATTATCACAGACAGAATGAATTTAACATACTTACAAGTTCGCGCTCAGGA 1800

KMP-4_16 (*r8A*) GACGCGTTCGATTATCACAGACAGAATGAATTTAACATACTTACAAGTTCGCGCTCAGGA 1800

KMP-4_26 (*r8A*) GACGCGTTCGATTATCACAGACAGAATGAATTTAACATACTTACAAGTTCGCGCTCAGGA 1800

KMP-4_3 (*r8A*) GACGCGTTCGATTATCACAGACAGAATGAATTTAACATACTTACAAGTTCGCGCTCAGGA 1800

KMP-4_8 (*r9A*) GACGCGTTCGATTATCACAGACAGAATGAATTTAACA----TACAAGTTCGCGCTCAGGA 1796

KMP-4_13 (*r9B*) GACGCGTTCGATTATCACAGACAGAATGAATTTAACA----TACAAGTTCGCGCTCAGGA 1796

AY198374.1 GACGCGTTCGATTATCACAGACAGAATGAATTTAACA----TACAAGTTCGCGCTCAGGA 1796

KMP-8_35 (*s6B*) GACGCGTGCGATTATCACAGACAGAATGAATTTAACA----TACAAGTTCGCGCTCAGGA 1793

KMP-8_24 (*s6A*) GACGCGTTCGATTATCACAGACAGAATGAATTTAACA----TACAAGTTCGCGCTCAGGA 1793

KMP-8_5 (*s6A*) GACGCGTTCGATTATCACAGACAGAATGAATTTAACA----TACAAGTTCGTGCTCAGGA 1793

KMP-8_46 (*s6A*) GACGCGTTCGATTATCACAGACAGAATGAATTTAACA----TACAAGTTCGCGCTCAGGA 1793

KMP-5_2 (*r10A*) CACCATGTCGGAGCCAGAGTCCAGGCATACAGCGACTGCTCAGCTGGTCATAGAACTCGA 1856

KMP-5_11 (*r10B*) CACCATGTCGGAGCCAGAGTCCAGGCATACAGCGACTGCTCAGCTGGTCATAGAACTCGA 1856

KMP-5_16 (*r10C*) CACCATGTCGGAGCCAGAGTCCAGGCATACAGCGACTGCTCAGCTGGTCATAGAACTCGA 1856

KMP-5_18 (*r10A*) CACCATGTCGGAGCCAGAGTCCAGGCATACAGCGACTGCTCAGCTGGTCATAGAACTCGA 1856

KMP-7_3 (*r12C*) CACCATGTCGGAGCCAGAGTCCAGGCATACAGCGACTGCTCAGCTGGTCATAGAACTCGA 1697

KMP-7_1 (*r12A*) CACCATGTCGGAGCCAGAGTCCAGGCATACAGCGACTGCTCAGCTGGTCATAGAACTCGA 1856

KMP-7_4 (*r12D*) ------------------------------------------------------------ 1653

KMP-6_7 (*r12A*) CACCATGTCGGAGCCAGAGTCCAGGCATACAGCGACTGCTCAGCTGGTCATAGAACTCGA 1856

KMP-7_2 (*r12B*) CACCATGTCGGAGCCAGAGTCCAGGCATACAGCGACTGCTCAGCTGGTCATAGAACTCGA 1735

KMP-6_3 (*r11A*) CACCATGTCGGAGCCAGAGTCCAGGCATACAGCGACTGCTCAGCTGGTCATAGAACTCGA 1856

KMP-6_5 (*r11B*) CACCATGTCGGAGCCAGAGTCCAGGCATACAGCGACTGCTCAGCTGGTCATAGAACTCGA 1856

KMP-6_8 (*r11B*) CACCATGTCGGAGCCAGAGTCCAGGCATACAGCGACTGCTCAGCTGGTCATAGAACTCGA 1856

KMP-8_3 (*s7*) CACCATGTCGGAGCCAGAGTCCAGGCATACAGCGACTGCTCAGCTGGTCATAGAACTCGA 1853

KMP-4_20 (*r8B*) CACCATGTCGGAGCCAGAGTCCAGGCATACAGCGACTGCTCAGCTGGTCATAGAACTCGA 1860

KMP-4_16 (*r8A*) CACCATGTCGGAGCCAGAGTCCAGGCATACAGCGACTGCTCAGCTGGTCATAGAACTCGA 1860

KMP-4_26 (*r8A*) TACCATGTCGGAGCCAGAGTCCAGGCATACAGCGACTGCTCAGCTGGTCATAGAACTCGA 1860

KMP-4_3 (*r8A*) CACCATGTCGGAGCCAGAGTCCAGGCATACAGCGACTGCTCAGCTGGTCATAGAACTCGA 1860

KMP-4_8 (*r9A*) CACCATGCCGGAGCCAGAGTCCAGGCATACAGCGACTGCTCAGCTGGTCATAGAACTCGA 1856

KMP-4_13 (*r9B*) CACCATGTCGGAGCCAGAGTCCAGGCATACAGCGACTGCTCAGCTGGTCATAGAACTCGA 1856

AY198374.1 CACCATGTCGGAGCCAGAGTCCAGGCATACAGCGACTGCTCAGCTGGTCATAGAACTCGA 1856

KMP-8_35 (*s6B*) CACCATGTCGGAGCCAGAGTCCAGGCATACAGCGACTGCTCAGCTGGTCATAGAACTCGA 1853

KMP-8_24 (*s6A*) CACCATGTCGGAGCCAGAGTCCAGGCATACAGCGACTGCTCAGCTGGTCATAGAACTCGA 1853

KMP-8_5 (*s6A*) CACCATGTCGGAGCCAGAGTCCAGGCATACAGCGACTGCTCAGCTGGTCATAGAACTCGG 1853

KMP-8_46 (*s6A*) CACCATGTCGGAGCCAGAGTCCAGGCATACAGCGACTGCTCAGCTGGTCATAGAACTCGA 1853

KMP-5_2 (*r10A*) GGACGTCAACAACACACCTCCTACTCTGAGGCTGCCTCGCGTAAGTCCGTCTGTAGAAGA 1916

KMP-5_11 (*r10B*) GGACGTCAACAACACACCTCCTACTCTGAGGCTGCCTCGCGTAAGTCCGTCTGTAGAAGA 1916

KMP-5_16 (*r10C*) GGACGTCAACAACACACCTCCTACTCTGAGGCTGCCTCGCGTAAGTCCGTCTGTAGAAGA 1916

KMP-5_18 (*r10A*) GGACGTCAACAACACACCTCCTACTCTGAGGCTGCCTCGCGTAAGTCCGTCTGTAGAAGA 1916

KMP-7_3 (*r12C*) GGACGTCAACAACACACCTCCTACTCTGAGGCTGCCGCGCGTAAGTCCGCCTGTAGAAGA 1757

KMP-7_1 (*r12A*) GGACGTCAACAACACACCTCCTACTCTGAGGCTGCCGCGCGTAAGTCCGTCTGTAGAAGA 1916

KMP-7_4 (*r12D*) ----------------------------------CCGCGCGTAAGTCCGTCTGTAGAAGA 1679

KMP-6_7 (*r12A*) GGACGTCAACAACACACCTCCTACTCTGAGGCTGCCGCGCGTAAGTCCGTCTGTAGAAGA 1916

KMP-7_2 (*r12B*) GGACGTCAACAACACACCTCCTACTCTGAGGCTGCCGCGCGTAAGTCCGTCTGTAGAAGA 1795

KMP-6_3 (*r11A*) GGACGTCAACAACACACCTCCTACTCTGAGGCTGCCGCGCGTAAGTCCGTCTGTAGAAGA 1916

KMP-6_5 (*r11B*) GGACGTCAACAACACACCTCCTACTCTGAGGCTGCCTCGCGTAAGTCCGTCTGTAGAAGA 1916

KMP-6_8 (*r11B*) GGACGTCAACAACACACCTCCTACTCTGAGGCTGCCTCGCGTAAGTCCGTCTGTAGAAGA 1916

KMP-8_3 (*s7*) GGACGTCAACAACACACCTCCTACTCTGAGGCTGCCTCGCGTAAGTCCGTCTGTAGAAGA 1913

KMP-4_20 (*r8B*) GGACGTCAACAACACACCTCCTACTCTGAGGCTGCCTCGCGTAAGTCCGTCTGTAGAAGA 1920

KMP-4_16 (*r8A*) GGACGTCAACAACACACCTCCTACTCTGAGGCT--------------------------- 1893

KMP-4_26 (*r8A*) GGACGTCAACAACACACCTCCTACTCTGAGGCT--------------------------- 1893

KMP-4_3 (*r8A*) GGACGTCAACAACACACCTCCTACTCTGAGGCT--------------------------- 1893

KMP-4_8 (*r9A*) GGACGTCAACAACACACCTCCTACTCTGAGGCTGCCTCGCGTAAGTCCGTCTGTAGAAGA 1916

KMP-4_13 (*r9B*) GGACGTCAACAACACACCTCCTACTCTGAGGCTGCCTCGCGTAAGTCCGTCTGTAGAAGA 1916

AY198374.1 GGACGTCAACAACACACCTCCTACTCTGAGGCTGCCTCGCGTAAGTCCGTCTGTAGAAGA 1916

KMP-8_35 (*s6B*) GGACGTCAACAACACACCTCCTACTCTGAGGCTGCCTCGCGTAAGTCCGTCTGTAGAAGA 1913

KMP-8_24 (*s6A*) GGACGTCAACAACACACCTCCTACTCTGAGGCTGCCTCGCGTAAGTCCGTCTGTAGAAGA 1913

KMP-8_5 (*s6A*) GGACGTCAACAACACACCTCCTACTCTGAGGCTGCCTCGCGTAAGTCCGTCTGTAGAAGA 1913

KMP-8_46 (*s6A*) GGACGTCAACAACACACCTCCTACTCTGAGGCTGCCTCGCGTAAGTCCGTCTGTAGAAGA 1913

KMP-5_2 (*r10A*) GAATGTGCCAGAGGGCTTTGAAATCAACCGGGAGATAACCGCCACGGACCCTGACACCAC 1976

KMP-5_11 (*r10B*) GAATGTGCCAGAGGGCTTTGAAATCAACCGGGAGATAACCGCCACGGACCCTGACACCAC 1976

KMP-5_16 (*r10C*) GAATGTGCCAGAGGGCTTTGAAATCAACCGGGAGATAACCGCCACGGACCCTGACACCAC 1976

KMP-5_18 (*r10A*) GAATGTGCCAGAGGGCTTTGAAATCAACCGGGAGATAACCGCCACGGACCCTGACACCAC 1976

KMP-7_3 (*r12C*) GAATGTGCCAGAGGGCTTTGAAATCAACCGGGAGATAACCGCCACGGACCCTGACACCAC 1817

KMP-7_1 (*r12A*) GAATGTGCCAGAGGGCTTTGAAATCAACCGGGAGATAACCGCCATGGACCCTGACACCAC 1976

KMP-7_4 (*r12D*) GAATGTGCTAGAGGGCTTTGAAATCAACCGGGAGATAACCGCCACGGACCCTGACACCAC 1739

KMP-6_7 (*r12A*) GAATGTGCCAGAGGGCTTTGAAATCAACCGGGAGATAACCGCCACGGACCCTGACACCAC 1976

KMP-7_2 (*r12B*) GAATGTGCCAGAGGGCTTTGAAATCAACCGGGAGATAACCGCCACGGACCCTGACACCAC 1855

KMP-6_3 (*r11A*) GAATGTGCCAGAGGGCTTTGAAATCAACCGGGAGATAACCGCCACGGACCCTGACACCAC 1976

KMP-6_5 (*r11B*) GAATGTGCCAGAGGGCTTTGAAGTCAACCGGGAGATAACCGCCACGGACCCTGACACCAC 1976

KMP-6_8 (*r11B*) GAATGTGCCAGAGGGCTTTGAAGTCAACCGGGAGATAACCGCCACGGACCCTGACACCAC 1976

KMP-8_3 (*s7*) GAATGGGCCAGAGGGCTTTGAAGTCAACCGGGAGATAACCGCCACGGACCCTGACACCAC 1973

KMP-4_20 (*r8B*) GAATGTGCCAGAGGGCTTTGAAATCAACCGGGAGATAACCGCCACGGACCCTGACACCAC 1980

KMP-4_16 (*r8A*) ------------------------------------------------------------ 1893

KMP-4_26 (*r8A*) ------------------------------------------------------------ 1893

KMP-4_3 (*r8A*) ------------------------------------------------------------ 1893

KMP-4_8 (*r9A*) GAATGTGCCAGAGGGCTTTGAAATCAACCGGGAGATAACCGCCACGGACCCTGGCACCAC 1976

KMP-4_13 (*r9B*) GAATGTGCCAGAGGGCTTTGAAATCAACCGGGAGATAACCGCCACGGACCCTGACACCAC 1976

AY198374.1 GAATGTGCCAGAGGGCTTTGAAATCAACCGGGAGATAACCGCCACGGACCCTGACACCAC 1976

KMP-8_35 (*s6B*) GAATGTGCCAGAGGGCTTTGGAGTCAACCGGGAGATAACCGCCACGGACCCTGACACCAC 1973

KMP-8_24 (*s6A*) GAATGTGCCAGAGGGCTTTGAAGTCAACCGGGAGATAACCGCCACGGACCCTGACACCAC 1973

KMP-8_5 (*s6A*) GAATGTGCCAGAGGGCTTTGAAGTCAACCGGGAGATAACCGCCACGGACCCTGACACCAC 1973

KMP-8_46 (*s6A*) GAATGTGCCAGAGGGCTTTGAAGTCAACCGGGAGATAACCGCCACGGACCCTGACACCAC 1973

KMP-5_2 (*r10A*) AGCATACCTGCAGTTTGAAATAGATTGGGACACATCCTTTGCCACTAAACAGGGGCGTGA 2036

KMP-5_11 (*r10B*) AGCATACCTGCAGTTTGAAATAGATTGGGACACATCCTTTGCCACTAAACAGGGGCGTGA 2036

KMP-5_16 (*r10C*) AGCATACCTGCAGTTTGAAATAGATTGGGACACATCCTTTGCCACTAAACAGGGGCGTGA 2036

KMP-5_18 (*r10A*) AGCATACCTGCAGTTTGAAATAGATTGGGACACATCCTTTGCCACTAAACAGGGGCGTGA 2036

KMP-7_3 (*r12C*) AGCATACCTGCAGTTTGAAATAGATTGGGACACATCCTTTGCCACTAAACAGGGGCGTGA 1877

KMP-7_1 (*r12A*) AGCATACCTGCAGTTTGAAATAGATTGGGACACATCCTTTGCCACTAAACAGGGGCGTGA 2036

KMP-7_4 (*r12D*) AGCATACCTGCAGTTTGAAATAGATTGGGACACATCCTTTGCCACTAAACAGGGGCGCGA 1799

KMP-6_7 (*r12A*) AGCATACCTGCAGTTTGAAATAGATTGGGACACATCCTTTGCCACTAAACAGGGGCGTGA 2036

KMP-7_2 (*r12B*) AGCATACCTGCAGTTTGAAATAGATTGGGACACATCCTTTGCCACTAAACAGGGGCGTGA 1915

KMP-6_3 (*r11A*) AGCATACCTGCAGTTTGAAATAGATTGGGACACATCCTTTGCCACTAAACAGGGGCGTGT 2036

KMP-6_5 (*r11B*) AGCATACCTGCAGTTTGAAATAGATTGGGACACATCCTTTGCCACTAAACAGGGGCGTGA 2036

KMP-6_8 (*r11B*) AGCATACCTGCAGTTTGAAATAGATTGGGACACATCCTTTGCCACTAAACAGGGGCGTGA 2036

KMP-8_3 (*s7*) AGCATACCTGCAGTTTGAAATAGATTGGGACACATCCTTTGCCACTAAACAGGGGCGTGA 2033

KMP-4_20 (*r8B*) AGCATACCTGCAGTTTGAAATAGATTGGGACACATCCTTTGCCACTAAACAGGGGCGTGA 2040

KMP-4_16 (*r8A*) ------------------------------------------------------------ 1893

KMP-4_26 (*r8A*) ------------------------------------------------------------ 1893

KMP-4_3 (*r8A*) ------------------------------------------------------------ 1893

KMP-4_8 (*r9A*) AGCATACCTGCAGTTTGAAATAGATTGGGACACTTCCTTTGCCACTAAACAGGGGCGTGA 2036

KMP-4_13 (*r9B*) AGCATACCTGCAGTTTGAAATAGATTGGGACACATCCTTTGCCACTAAACAGGGGCGTGA 2036

AY198374.1 AGCATACCTGCAGTTTGAAATAGATTGGGACACATCCTTTGCCACTAAACAGGGGCGTGA 2036

KMP-8_35 (*s6B*) AGCATACTTGCAGTTTGAAATAGATTGGGACACATCCTTTGCCACTAAACAGGGGCGTGA 2033

KMP-8_24 (*s6A*) AGCATACCTGCAGTTTGAAATAGATTGGGACACATCCTTTGCCACTAAACAGGGGCGTGA 2033

KMP-8_5 (*s6A*) AGCATACCTGCAGTTTGAAATAGATTGGGACACATCCTTTGCCACTAAACAGGGGCGTGA 2033

KMP-8_46 (*s6A*) AGCATACCTGCAGTTTGAAATAGATTGGGACACATCCTTTGCCACTAAACAGGGGCGTGA 2033

KMP-5_2 (*r10A*) TACCAATCCAATAGAGTTCCACGGATGCGTGGATATAGAAACCATCTTCCCAAACCCAGC 2096

KMP-5_11 (*r10B*) TACCAATCCAATAGAGTTCCACGGATGCGTGGATATAGAAACCATCTTCCCAAACCCAGC 2096

KMP-5_16 (*r10C*) TACCAATCCAATAGAGTTCCACGGATGCGTGGATATAGAAACCATCTTCCCAAACCCAGC 2096

KMP-5_18 (*r10A*) TACCAATCCAATAGAGTTCCACGGATGCGTGGATATAGAAACCATCTTCCCAAACCCAGC 2096

KMP-7_3 (*r12C*) TACCAATCCAGTAGAGTTCCACGGATGCGTGGATATAGAAACCATCTTCCCAAACCCAGC 1937

KMP-7_1 (*r12A*) TACCAATCCAGTAGAGTTCCACGGATGCGTGGATATAGAAACCATCTTCCCAAGCCCAGC 2096

KMP-7_4 (*r12D*) TACCAATCCAGTAGAGTTCCACGGATGCGTGGATATAGAAACCATCTTCCCAAACCCAGC 1859

KMP-6_7 (*r12A*) TACCAATCCAGTAGAGTTCCACGGATGCGTGGATATAGAAACCATCTTCCCAAACCCAGC 2096

KMP-7_2 (*r12B*) TACCAATCCAGTAGAGTTCCACGGATGCGTGGATATAGAAACCATCTTCCCAAACCCAGC 1975

KMP-6_3 (*r11A*) TACCAATCCAGTAGAGTTCCACGGATGCGTGGATATAGAAACCATCTTCCCAAACCCAGC 2096

KMP-6_5 (*r11B*) TACCAATCCAATAGAGTTCCACGGATGCGTGGATATAGAAACCATCTTCCCAAACCCAGC 2096

KMP-6_8 (*r11B*) TACCAATCCAATAGAGTTCCACGGATGCGTGGATATAGAAACCATCTTCCCAAACCCAGC 2096

KMP-8_3 (*s7*) TACCAATCCAATAGAGTTCCACGGATGCGTGGATATAGAAACCATCTTCCCAAACCCAGC 2093

KMP-4_20 (*r8B*) TACCAATCCAATAGAGTTCCACGGATGCGTGGATATAGAAACCATCTTCCCAAACCCAGC 2100

KMP-4_16 (*r8A*) -----------------------GATGCGTGGATATAGAAACCATCTTCCCAAACCCAGC 1930

KMP-4_26 (*r8A*) -----------------------GATGCGTGGATATAGAAACCATCTTCCCAAACCCAGC 1930

KMP-4_3 (*r8A*) -----------------------GATGCGTGGATATAGAAACCATCTTCCCAAACCCAGC 1930

KMP-4_8 (*r9A*) TACCAATCCAATAGAGTTCCACGGATGCGTGGATATAGAAACCATCTTCCCAAACCCAGC 2096

KMP-4_13 (*r9B*) TACCAATCCAATAGAGTTCCACGGATGCGTGGATATAGAAACCATCTTCCCAAACCCAGC 2096

AY198374.1 TACCAATCCAATAGAGTTCCACGGATGCGTGGATATAGAAACCATCTTCCCAAACCCAGC 2096

KMP-8_35 (*s6B*) TACCAATCCAATAGAGTTCCACGGATGCGTGGATATAGAAACCATCTTCCCAAACCCAGC 2093

KMP-8_24 (*s6A*) TACCAATCCAATAGAGTTCCACGGATGCGTGGATATAGAAACCATCTTCCCAAACCCAGC 2093

KMP-8_5 (*s6A*) TACCAATCCAATAGAGTTCCACGGATGCGTGGATATAGAAACCATCTTCCCAAACCCAGC 2093

KMP-8_46 (*s6A*) TACCAATCCAATAGAGTTCCACGGATGCGTGGATATAGAAACCATCTTCCCAAACCCAGC 2093

******************************.******

KMP-5_2 (*r10A*) CGACACCAGAGAGGCTGTGGGGCGAGTGGTAGCGAAGGAGATCCGCCATAACGTGGCCAT 2156

KMP-5_11 (*r10B*) CGACACCAGAGAGGCTGTGGGGCGAGTGGTAGCGAAGGAGATCCGCCATAACGTGACCAT 2156

KMP-5_16 (*r10C*) CGACACCAGAGAGGCTGTGGGGCGAGTGGTAGCGAAGGAGATCCGCCATAACGTGACCAT 2156

KMP-5_18 (*r10A*) CGACACCAGAGAGGCTGTGGGGCGAGTGGTAGCGAAGGAGATCCGCCATAACGTGACCAT 2156

KMP-7_3 (*r12C*) CGACACCAGAGAAGCTGTGGGGCGAGTGGTAGCGAAGGAGATCCGCCATAACGTGACCAT 1997

KMP-7_1 (*r12A*) CGACACCAGAGAAGCTGTGGGGCGAGTGGTAGCGAAGGAGATCCGCCATAACGTGACCAT 2156

KMP-7_4 (*r12D*) CGACACCAGAGAAGCTGTGGGGCGAGTGGTAGCGAAGGAGATCCGCCATAACGTGACCAT 1919

KMP-6_7 (*r12A*) CGACACCAGAGAAGCTGTGGGGCGAGTGGTAGCGAAGGAGATCCGCCATAACGTGACCAT 2156

KMP-7_2 (*r12B*) CGACACCAGAGAAGCTGTGGGGCGAGTGGTAGCGAAGGAGATCCGCCATAACGTGACCAT 2035

KMP-6_3 (*r11A*) CGACACCAGAGAAGCTGTGGGGCGAGTGGTAGCGAAGGAGATCCGCCATAATGTGACCAT 2156

KMP-6_5 (*r11B*) CGACACCAGAGAGGCTGTGGGGCGAGTGGTAGCGAAGGAGATCCGCCATAACGTGACCAT 2156

KMP-6_8 (*r11B*) CGACACCAGAGAGGCTGTGGGGCGAGTGGTAGCGAAGGAGATCCGCCATAACGTGACCAT 2156

KMP-8_3 (*s7*) CGACACCAGAGAGGCTGTGGGGCGAGTGGTAGCGAAGGAGATCCGCCATAACGTGACCAT 2153

KMP-4_20 (*r8B*) CGACACCAGAGAGGCTGTGGGGCGAGTGGTAGCGAAGGAGATCCGCCATAACGTGACCAT 2160

KMP-4_16 (*r8A*) CGACACCAGAGAGGCTGTGGGGCGAGTGGTAGCGAAGGAGATCCGCCATAACGTGACCAT 1990

KMP-4_26 (*r8A*) CGACACCAGAGAGGCTGTGGGGCGAGTGGTAGCGAAGGAGATCCGCCATAACGTGACCAT 1990

KMP-4_3 (*r8A*) CGACACCAGAGAGGCTGTGGGGCGAGTGGTAGCGAAGGAGATCCGCCATAACGTGACCAT 1990

KMP-4_8 (*r9A*) CGACACCAGAGAGGCTGTGGGGCGAGTGGTAGCGAAGGAGATCCGCCATAACGTGACCAT 2156

KMP-4_13 (*r9B*) CGACACCAGAGAGGCTGTGGGGCGAGTGGTAGCGAAGGAGATCCGCCATAACGTGACCAT 2156

AY198374.1 CGACACCAGAGAGGCTGTGGGGCGAGTGGTAGCGAAGGAGATCCGCCATAACGTGACCAT 2156

KMP-8_35 (*s6B*) CGACACCAGAGAGGCTGTGGGGCGAGTGGTAGCGAAGGAGATCCGCCATAACGTGACCAT 2153

KMP-8_24 (*s6A*) CGACATCAGAGAGGCTGTGGGGCGAGTGGTAGCGAAGGAGATCCGCCATAACGTGACCAT 2153

KMP-8_5 (*s6A*) CGACACCAGAGAGGCTGTGGGGCGAGTGGTAGCGAAGGAGATCCGCCATAACGTGACCAT 2153

KMP-8_46 (*s6A*) CGACACCAGAGAGGCTGTGGGGCGAGTGGTAGCGAAGGAGATCCGCCATAACGTGACCAT 2153

***** ******.************************************** ***.****

KMP-5_2 (*r10A*) CGATTTTGAAGAGTTTGAATTTCTCTACCTCACAGTGAGAGTTCGGGACTTGCACACAGA 2216

KMP-5_11 (*r10B*) CGATTTTGAAGAGTTTGAATTTCTCTACCTCACAGTGAGAGTTCGGGACTTGCACACAGA 2216

KMP-5_16 (*r10C*) CGATTTTGAAGAGTTTGAATTTCTCTACCTCACAGTGAGAGTTCGGGACTTGCACACAGA 2216

KMP-5_18 (*r10A*) CGATTTTGAAGAGTTTGAATTTCTCTACCTCACAGTGAGAGTTCGGGACTTGCACACAGA 2216

KMP-7_3 (*r12C*) CGATTTTGAAGAGTTTGAATTTCTGTACCTCACAGTGAGAGTTCGAGACTTGCACACAGA 2057

KMP-7_1 (*r12A*) CGATTTTGAAGAGTTTGAATTTCTGTACCTCACAGTGAGAGTTCGAGACTTGCACACAGA 2216

KMP-7_4 (*r12D*) CGATTTTGAAGAGTTTGAATTTCTGTACCTCACAGTGAGAGTTCGAGACTTGCACACAGA 1979

KMP-6_7 (*r12A*) CGATTTTGAAGAGTTTGAATTTCTGTACCTCACAGTGAGAGTTCGAGACTTGCACACAGA 2216

KMP-7_2 (*r12B*) CGATTTTGAAGAGTTTGAATTTCTGTACCTCACAGTGAGAGTTCGAGGCTTGCACACAGA 2095

KMP-6_3 (*r11A*) CGATTTTGAAGAGTTTGAATTTCTGTACCTCACAGTGAGAGTTCGAGACTTGCACACAGA 2216

KMP-6_5 (*r11B*) CGATTTTGAAGAGTTTGAATTTCTCTACCTCACAGTGAGAGTTCGGGACTTGCACACAGA 2216

KMP-6_8 (*r11B*) CGATTTTGAAGAGTTTGAATTTCTCTACCTCACAGTGAGAGTTCGGGACTTGCACACAGA 2216

KMP-8_3 (*s7*) CGATTTTGAAGAGTTTGAATTTCTCTACCTCACAGTGAGAGTTCGGGACTTGCACACAGA 2213

KMP-4_20 (*r8B*) CGATTTTGAAGAGTTTGAATTTCTCTACCTCACAGTGAGAGTTCGGGACTTGCACACAGA 2220

KMP-4_16 (*r8A*) CGATTTTGAAGAGTTTGAATTTCTCTACCTCACAGTGAGAGTTCGGGACTTGCACACAGA 2050

KMP-4_26 (*r8A*) CGATTTTGAAGAGTTTGAATTTCTCTACCTCACAGTGAGAGTTCGGGACTTGCACACAGA 2050

KMP-4_3 (*r8A*) CGATTTTGAAGAGTTTGAATTTCTCTACCTCACAGTGAGAGTTCGGGACTTGCACACAGA 2050

KMP-4_8 (*r9A*) CGATTTTGAAGAGTTTGAATTTCTCTACCTCACAGTGAGAGTTCGGGACTTGCACACAGA 2216

KMP-4_13 (*r9B*) CGATTTTGAAGAGTTTGAATTTCTCTACCTCACAGTGAGAGTTCGGGACTTGCACACAGA 2216

AY198374.1 CGATTTTGAAGAGTTTGAATTTCTCTACCTCACAGTGAGAGTTCGGGACTTGCACACAGA 2216

KMP-8_35 (*s6B*) CGATTTTGAAGAGTTTGAATTTCTCTACCTCACAGTGAGAGTTCGGGACTTGCACACAGA 2213

KMP-8_24 (*s6A*) CGATTTTGAAGAGTTTGAATTTCTCTACCTCACAGTGAGAGTTCGGGACTTGCACACAGA 2213

KMP-8_5 (*s6A*) CGATTTTGAAGAGTTTGAATTTCTCTACCTCACAGTGAGAGTTCGGGACTTGCACACAGA 2213

KMP-8_46 (*s6A*) CGATTTTGAAGAGTTTGAATTTCTCTACCTCACAGTGAGAGTTCGGGACTTGCACACGGA 2213

************************ ********************.*.*********.**

KMP-5_2 (*r10A*) TGACGGACGAGATTATGATGAATCTACCTTCACGATAATAATAATAGATATGAACGACAA 2276

KMP-5_11 (*r10B*) TGACGGACGAGATTATGATGAATCTACCTTCACGATAATAATAATAGATATGAACGACAA 2276

KMP-5_16 (*r10C*) TGACGGACGAGATTATGATGAATCTACCTTCACGATAATAATAATAGATATGAACGACAA 2276

KMP-5_18 (*r10A*) TGACGGACGAGATTATGATGAATCTACCTTCACGATAATAATAATAGATATGAACGACAA 2276

KMP-7_3 (*r12C*) AGATGGACGAGATTATGATGAATCTACCTTCACGATAATAATAATAGATATGAACGACAA 2117

KMP-7_1 (*r12A*) AGATGGACGAGATTATGATGAACCTACCTTCACGATAATAATAATAGATATGAACGACAA 2276

KMP-7_4 (*r12D*) AGATGGACGAGATTATGATGAATCTACCTTCACGATAATAATAATAGATATGAACGACAA 2039

KMP-6_7 (*r12A*) AGATGGACGAGATTATGATGAATCTACCTTCACGATAATAATAATAGATATGAACGACAA 2276

KMP-7_2 (*r12B*) AGATGGACGAGATTATGATGAATCTACCTTCACGATAATAATAATAGATATGAACGACAA 2155

KMP-6_3 (*r11A*) AGATGGACGAGATTATGATGAATCTACCTTCACGATAATAATAATAGATATGAACGACAA 2276

KMP-6_5 (*r11B*) TGACGGACGAGATTATGATGAATCTACCTTCACGATAATAATAATAGATATGAACGACAA 2276

KMP-6_8 (*r11B*) TGACGGACGAGATTATGATGAATCTACCTTCACGATAATAATAATAGATATGAACGACAA 2276

KMP-8_3 (*s7*) TGACGGACGAGATTATGATGAATCTACCTTCACGATAATAATAATAGATATGAACGACAA 2273

KMP-4_20 (*r8B*) TGACGGACGAGATTATGATGAATCTACCTTCACGATAATAATAATAGATATGAACGACAA 2280

KMP-4_16 (*r8A*) TGACGGACGAGATTATGATGAATCTACCTTCACGATAATAATAATAGATATGAACGACAA 2110

KMP-4_26 (*r8A*) TGACGGACGAGATTATGATGAATCTACCTTCACGATAATAATAATAGATATGAACGACAA 2110

KMP-4_3 (*r8A*) TGACGGACGAGATTATGATGAATCTACCTTCACGATAATAATAATAGATATGAACGACAA 2110

KMP-4_8 (*r9A*) TGACGGACGAGATTATGATGAATCTACCTTCACGATAATAATAATAGATATGAACGACAA 2276

KMP-4_13 (*r9B*) TGACGGACGAGATTATGATGAATCTACCTTCACGATAATAATAATAGATATGAACGACAA 2276

AY198374.1 TGACGGACGAGATTATGATGAATCTACCTTCACGATAATAATAATAGATATGAACGACAA 2276

KMP-8_35 (*s6B*) TGACGGACGAGATTATGATGAATCTACCTTCACGATAATAATAATAGATATGAACGACAA 2273

KMP-8_24 (*s6A*) TGACGGACGAGATTATGATGAATCTACCTTCACGATAATAATAATAGATATGAACGACAA 2273

KMP-8_5 (*s6A*) TGACGGACGAGATTATGATGAATCTACCTTCACGATAATAATAATAGATATGAACGACAA 2273

KMP-8_46 (*s6A*) TGACGGACGAGATTATGATGAATCTACCTTCACGATAATAATAATAGATATGAACGACAA 2273

:** ****************** *************************************

KMP-5_2 (*r10A*) CTGGCCTATCTGGGCGTCTGGTTTCCTGAACCAGACCTTCAGTATCCGGGAGCGATCATC 2336

KMP-5_11 (*r10B*) CTGGCCTATCTGGGCGTCTGGTTTCCTGAACCAGACCTTCAGTATCCGGGAGCGATCATC 2336

KMP-5_16 (*r10C*) CTGGCCTATCTGGGCGTCTGGTTTCCTGAACCAGACCTTCAGTATCCGGGAGCGATCATC 2336

KMP-5_18 (*r10A*) CTGGCCTATCTGGGCGTCTGGTTTCCTGAACCAGACCTTCAGTATCCGGGAGCGATCATC 2336

KMP-7_3 (*r12C*) CTGGCCTATCTGGGCGTCTGGTTTCCTGAACCAGACCTTCAGTATTCGGGAGCGATCATC 2177

KMP-7_1 (*r12A*) CTGGCCTATCTGGGCGTCTGGTTTCCTGAACCAGACCTTCTGTATTCGGGAGCGATCATC 2336

KMP-7_4 (*r12D*) CTGGCCTATCTGGGCGTCTGGTTTCCTGAACCAGACCTTCAGTATTCGGGAGCGATCATC 2099

KMP-6_7 (*r12A*) CTGGCCTATCTGGGCGTCTGGTTTCCTGAACCAGACCTTCAGTATTCGGGAGCGATCATC 2336

KMP-7_2 (*r12B*) CTGGCCTATCTGGGCGTCTGGTTTCCTGAACCAGACCTTCAGTATTCGGGAGCGATCATC 2215

KMP-6_3 (*r11A*) CTGGCCTATCTGGGCGTCTGGTTTCCTGAACCAGACCTTCAGTATTCGGGAGCGATCATC 2336

KMP-6_5 (*r11B*) CTGGCCTATCTGGGCGTCTGGTTTCCTGAACCAGACCTTCAGTATTCGGGAGCGATCATC 2336

KMP-6_8 (*r11B*) CTGGCCTATCTGGGCGTCTGGTTTCCTGAACCAGACCTTCAGTATTCGGGAGCGATCATC 2336

KMP-8_3 (*s7*) CTGGCCTATCTGGGCGTCTGGTTTCCTGAACCAGACCTTCAGTATTCGGGAGCGATCATC 2333

KMP-4_20 (*r8B*) CTGGCCTATCTGGGCGTCTGGTTTCCTGAACCAGACCTTCAGTATCCGGGAGCGATCATC 2340

KMP-4_16 (*r8A*) CTGGCCTATCTGGGCGTCTGGTTTCCTGAACCAGACCTTCAGTATCCGGGAGCGATCATC 2170

KMP-4_26 (*r8A*) CTGGCCTATCTGGGCGTCTGGTTTCCTGAACCAGACCTTCAGTATCCGGGAGCGATCATC 2170

KMP-4_3 (*r8A*) CTGGCCTATCTGGGCGTCTGGTTTCCTGAACCAGACCTTCAGTATCCGGGAGCGATCATC 2170

KMP-4_8 (*r9A*) CTGGCCTATCTGAGCGTCTGGTTTCCTGAACCAGACCTTCAGTATTCGGGAGCGATCATC 2336

KMP-4_13 (*r9B*) CTGGCCTATCTGGGCGTCTGGTTTCCTGAACCAGACCTTCAGTATTCGGGAGCGATCATC 2336

AY198374.1 CTGGCCTATCTGGGCGTCTGGTTTCCTGAACCAGACCTTCAGTATTCGGGAGCGATCATC 2336

KMP-8_35 (*s6B*) CTGGCCTATCTGGGCGTCTGGTTTCCTGAACCAGACCTTCAGTATTCGGGAGCGATCATC 2333

KMP-8_24 (*s6A*) CTGGCCTATCTGGGCGTCTGGTTTCCTGAACCAGACCTTCAGTATTCGGGAGCGATCATC 2333

KMP-8_5 (*s6A*) CTGGCCTATCTGGGCGTCTGGTTTCCTGAACCAGACCTTCAGTATTCGGGAGCGATCATC 2333

KMP-8_46 (*s6A*) CTGGCCTATCTGGGCGTCTGGTTTCCTGAACCAGACCTTCAGTATTCGGGAGCGATCATC 2333

************.***************************:**** **************

KMP-5_2 (*r10A*) TACCGGCGTCGTCATCGGGTCCGTACTCGCTACAGACATTGATGGCCCACTTTACAACCA 2396

KMP-5_11 (*r10B*) CACCGGCGTCGTCATCGGGTCCGTACTCGCTACAGACATTGATGGCCCACTTTACAACCA 2396

KMP-5_16 (*r10C*) TACCGGCGTCGTCATCGGGTCCGTACTCGCTACAGACATTGATGGCCCACTTTACAACCA 2396

KMP-5_18 (*r10A*) TACCGGCGTCGTCATCGGGTCCATACTCGCTACAGACATTGATGGCCCACTTTACAACCA 2396

KMP-7_3 (*r12C*) TACCGGCGTCGTCATCGGGTCCGTACTCGCTACAGACATTGATGGCCCACTTTACAACCA 2237

KMP-7_1 (*r12A*) TACCGGCGTCGTCATCGGGTCCGTACTCGCTACAGACATTGATGGCCCACTTTACAACCA 2396

KMP-7_4 (*r12D*) TACCGGCGTCGTCATCGGGTCCGTACTCGCTACAGACATTGATGGCCCACTTTACGACCA 2159

KMP-6_7 (*r12A*) TACCGGCGTCGTCATCGGGTCCGTACTCGCTACAGACATTGATGGCCCACTTTACAACCA 2396

KMP-7_2 (*r12B*) TACCGGCGTCGTCATCGGGTCCGTACTCGCTACAGACATTGATGGCCCACTTTACAACCA 2275

KMP-6_3 (*r11A*) TACCGGCGTCGTCATCGGGTCCGTACTCGCTACAGACATTGATGGCCCACTTTACAACCA 2396

KMP-6_5 (*r11B*) TACCGGCGTCGTCATCGGGTCCGTACTCGCTACAGACATTGATGGCCCACTTTACAACCA 2396

KMP-6_8 (*r11B*) TACCGGCGTCGTCATCGGGTCCGTACTCGCTACAGACATTGATGGCCCACTTTACAACCA 2396

KMP-8_3 (*s7*) TACCGGCGTCGTCATCGGGTCCGTACTCGCTACAGACATTGACGGCCCACTTTACAACCA 2393

KMP-4_20 (*r8B*) TACCGGCGTCGTCATCGGGTCCGTACTCGCTACAGACATTGATGGCCCACTTTACAACCA 2400

KMP-4_16 (*r8A*) TACCGGCGTCGTCATCGGGTCCGTACTCGCTACAGACATTGATGGCCCACTTTACAACCA 2230

KMP-4_26 (*r8A*) TACCGGCGTCGTCATCGGGTCCGTACTCGCTACAGACATTGATGGCCCACTTTACAACCA 2230

KMP-4_3 (*r8A*) TACCGGCGTCGTCATCGGGTCCGTACTCGCTACAGACATTGATGGCCCACTTTACAACCA 2230

KMP-4_8 (*r9A*) TACCGGCGTCGTCATCGGGTCCGTACTCGCTACAGACATTGATGGCCCACTTTACAACCA 2396

KMP-4_13 (*r9B*) TACCGGCGTCGTCATCGGGTCCGTACTCGCTACAGACATTGATGGCCCACTTTACAACCA 2396

AY198374.1 TACCGGCGTCGTCATCGGGTCCGTACTCGCTACAGACATTGATGGCCCACTTTACAACCA 2396

KMP-8_35 (*s6B*) TACCGGCGTCGTCATCGGGTCCGTACTCGCTACAGACATTGATGGCCCACTTTGCAACCA 2393

KMP-8_24 (*s6A*) TACCGGCGTCGTCATCGGGTCCGTACTCGCTACAGACATTGATGGCCCACTTTACAACCA 2393

KMP-8_5 (*s6A*) TACCGGCGTCGTCATCGGGTCCGTACTCGCTACAGACATTGATGGCCCACTTTACAACCA 2393

KMP-8_46 (*s6A*) TACCGGCGTCGTCATCGGGTCCGTACTCGCTACAGACATTGATGGCCCACTTTACAACCA 2393

*********************.******************* **********.*.****

KMP-5_2 (*r10A*) AGTCCGGTACACCATTATCCCCCAGGAAGATACTCCTGAAGGTCTAGTCCAGATACACTT 2456

KMP-5_11 (*r10B*) AGTCCGGTACACCATTATCCCCCAGGAAGATACTCCTGAAGGTCTAGTCCAGATACACTT 2456

KMP-5_16 (*r10C*) AGTCCGGTACACCATTATCCCCCAGGAAGATACCCCTGAAGGTCTAGTCCAGATACACTT 2456

KMP-5_18 (*r10A*) AGTCCGGTACACCATTATCCCCCTGGAAGATACTCCTGAAGGTCTAGTCCAGATACACTT 2456

KMP-7_3 (*r12C*) AGTCCGGTACACCATTATCCCCCAGGAAGATACTCCTGAAGGTCTAGTCCAGATACACTT 2297

KMP-7_1 (*r12A*) AGTCCGGTACACCATCATCCCCCAGGAAGATACTCCTGAAGGTCTAGTCCAGATACACTT 2456

KMP-7_4 (*r12D*) AGTCCGGTACACCATTATCCCCCAGGAAGATACTCCTGAAGGTCTAGTCCAGATACACTT 2219

KMP-6_7 (*r12A*) AGTCCGGTACACCATTATCCCCCAGGAAGATACTCCTGAAGGTCTAGTCCAGATACACTT 2456

KMP-7_2 (*r12B*) AGTCCGGTACACCATTATCCCCCAGGAAGATACTCCTGAAGGTCTAGTCCAGATACACTT 2335

KMP-6_3 (*r11A*) AGTCCGGTACACCATTATCCCCCAGGAAGATACTCCAGAAGGTCTAGTCCAGATACACTT 2456

KMP-6_5 (*r11B*) AGTCCGGTACACCATTATCCCCCAGGAAGATACTCCTGAAGGTCTAGTCCAGATACATTT 2456

KMP-6_8 (*r11B*) AGTCCGGTACACCATTATCCCCCAGGAAGATACTCCTGAAGGTCTAGTCCAGATACATTT 2456

KMP-8_3 (*s7*) AGTCCGGTATACCATTATCCCCCAGGAAGATACTCCTGAAGGTCTAGTCCAGATACACTT 2453

KMP-4_20 (*r8B*) AGTCCGGTACACCATTATCCCCCAGGAAGATACTCCTGAAGGTCTAGTCCAGATACACTT 2460

KMP-4_16 (*r8A*) AGTCCGGTACACCATTATCCCCCAGGAAGATACTCCTGAAGGTCTAGTCCAGATACACTT 2290

KMP-4_26 (*r8A*) AGTCCGGTACACCATTATCCCCCAGGAAGATACTCCTGAAGGTCTAGTCCAGATACACTT 2290

KMP-4_3 (*r8A*) AGTCCGGTACACCATTATCCCCCAGGAAGATACTCCTGAAGGTCTAGTCCAGATACACTT 2290

KMP-4_8 (*r9A*) AGTCCGGTACACCATTATCCCCCAGGAAGATACTCCTGAAGGTCTAGTCCAGATACATTT 2456

KMP-4_13 (*r9B*) AGTCCGGTACACCATTATCCCCCAGGAAGATACTCCTGAAGGTCTAGTCCAGATACATTT 2456

AY198374.1 AGTCCGGTACACCATTATCCCCCAGGAAGATACTCCTGAAGGTCTAGTCCAGATACACTT 2456

KMP-8_35 (*s6B*) AGTCCGGTATACCATTATCCCCCAGGAAGATACTCCTGAAGGTCTAGTCCAGATACACTT 2453

KMP-8_24 (*s6A*) AGTCCGGTATACCATTATCCCCCAGGAAGATACTCCTGAAGGTCTAGTCCAGATACACTT 2453

KMP-8_5 (*s6A*) AGTCCGGTATACCATTATCCCCCAGGAAGATACTCCTGAAGGTCTAGTCCAGATACACTT 2453

KMP-8_46 (*s6A*) AGTCCGGTATACCATTATCCCCCAGGAAGATACTCCTGAAGGTCTAGTCCAGATACACTT 2453

********* ***** *******:********* **:******************** **

KMP-5_2 (*r10A*) CGTTACGGGTCAGATTACAGTTGATGAGAATGGTGCAATCGACGCTGATATTCCACCTCG 2516

KMP-5_11 (*r10B*) CGTTACGGGTCAGATTACAGTTGATGAGAATGGTGCAATCGACGCTGATATTCCACCTCG 2516

KMP-5_16 (*r10C*) CGTTACGGGTCAGATTACAGTTGATGAGAATGGTGCAATCGACGCTGATATTCCACCTCG 2516

KMP-5_18 (*r10A*) CGTTACGGGTCAGATTACAGTTGATGAGAATGGTGCAATCGACGCTGATATTCCACCTCG 2516

KMP-7_3 (*r12C*) CGTTACGGGTCAGATTACAGTTGATGAGAATGGTGCAATCGACGCTGATATTCCACCTCG 2357

KMP-7_1 (*r12A*) CGTTACGGGTCAGATTACAGTTGATGAGAATGGTGCAATCGACGCTGATATTCCACCTCG 2516

KMP-7_4 (*r12D*) CGTTACGGGTCAGATTACAGTTGATGAGAATGGTGCAATCGACGCTGATATTCCACCTCG 2279

KMP-6_7 (*r12A*) CGTTACGGGTCAGATTACAGTTGATGAGAATGGTGCAATCGACGCTGATATTCCACCTCG 2516

KMP-7_2 (*r12B*) CGTTACGGGTCAGATTACAGTTGATGAGAATGGTGCAATCGACGCTGATATTCCACCTCG 2395

KMP-6_3 (*r11A*) CGTTACGGGTCAGATTACAGTTGATGAGAATGGTGCAATCGACGCTGATATTCCACCTCG 2516

KMP-6_5 (*r11B*) CGTTACGGGTCAGATTACAGTTGATGAGAATGGTGCAATCGACGCTGATATTCCACCTCG 2516

KMP-6_8 (*r11B*) CGTTACGGGTCAGATTACAGTTGATGAGAATGGTGCAATCGACGCTGATATTCCACCTCG 2516

KMP-8_3 (*s7*) CGTTACGGGTCAGATTACAGTTGATGAGAATGGTGCAATCGACGCTGATATTCCACCTCG 2513

KMP-4_20 (*r8B*) CGTTACGGGTCAGATTACAGTTGATGAGAATGGTGCAATCGACGCTGATATTCCACCTTG 2520

KMP-4_16 (*r8A*) CGTTACGGGTCAGATTACAGTTGATGAGAATGGTGCAATCGACGCTGATATTCCACCTCG 2350

KMP-4_26 (*r8A*) CGTTACGGGTCAGATTACAGTTGATGAGAATGGTGCAATCGACGCTGATATTCCACCTCG 2350

KMP-4_3 (*r8A*) CGTTACGGGTCAGATTACAGTTGATGAGAATGGTGCAATCGACGCTGATATTCCACCTCG 2350

KMP-4_8 (*r9A*) CGTTACGGGTCAGATTACAGTTGATGAGAATGGTGCAATCGACGCTGATATTCCACCTCG 2516

KMP-4_13 (*r9B*) CGTTACGGGTCAGATTACAGTTGATGAGAATGGTGCAATCGACGCTGATATTCCACCTCG 2516

AY198374.1 CGTTACGGGTCAGATTACAGTTGATGAGAATGGTGCAATCGACGCTGATATTCCACCTCG 2516

KMP-8_35 (*s6B*) CGTTACGGGTCAGATTACAGTTGATGAGAATGGTGCAATCGACGCTGATATTCCACCTCG 2513

KMP-8_24 (*s6A*) CGTTACGGGTCAGATTACAGTTGATGAGAATGGTGCAATCGACGCTGATATTCCACCTCG 2513

KMP-8_5 (*s6A*) CGTTACGGGTCAGATTACAGTTGATGAGAATGGTGCAATCGACGCTGATATTCCACCTCG 2513

KMP-8_46 (*s6A*) CGTTACGGGTCAGATTACAGTTGATGAGAATGGTGCAATCGACGCTGATATTCCACCTCG 2513

********************************************************** *

KMP-5_2 (*r10A*) TTGGCACCTCAACTACACGGTTATAGCCAGCGACAAATGTTCCGAAGAAAATGAAGAGAA 2576

KMP-5_11 (*r10B*) TTGGCACCTCAACTACACGGTTATAGCCAGCGACAAATGTTCCGAAGAAAATGAAGAGAA 2576

KMP-5_16 (*r10C*) TTGGCACCTCAACTACACGGTTATAGCCAGCGACAAATGTTCCGAAGAAAATGAAGAGAA 2576

KMP-5_18 (*r10A*) TTGGCACCTCAACTACACGGTTATAGCCAGCGACAAATGTTCCGAAGAAAATGAAGAGAA 2576

KMP-7_3 (*r12C*) TTGGCGCCTCAACTACACAGTTATAGCCAGCGACAAATGTTCCGAAGAAAATGAAGAGAA 2417

KMP-7_1 (*r12A*) TTGGCACCTCAACTACACAGTTATAGCCAGCGACAAATGTTCCGAAGAAAATGAAGAGAA 2576

KMP-7_4 (*r12D*) TTGGCACCTCAACTACACAGTTATAGCCAGCGACAAATGTTCCGAAGAAAATGAAGAGAA 2339

KMP-6_7 (*r12A*) TTGGCACCTCAACTACACAGTTATAGCCAGCGACAAATGTTCCGAAGAAAATGAAGAGAA 2576

KMP-7_2 (*r12B*) TTGGCACCCCAACTACACAGTTATAGCCAGCGACAAATGTTCCGAAGAAAATGAAGAGAA 2455

KMP-6_3 (*r11A*) TTGGCACCTCAACTACACAGTTATAGCCAGCGACAAATGTTCCGAAGAAAATGAAGAGAA 2576

KMP-6_5 (*r11B*) TTGGCACCTCAACTACACGGTTATAGCCAGCGACAAATGTTCTGAAGAAAATGAAGAGAA 2576

KMP-6_8 (*r11B*) TTGGCACCTCAACTACACGGTTATAGCCAGCGACAAATGTTCTGAAGAAAATGAAGAGAA 2576

KMP-8_3 (*s7*) TTGGCACCTCAACTACACGATTATAGCCAGCGACAAATGTTCCGAAGAAAATGAAGAGAA 2573

KMP-4_20 (*r8B*) TTGGCACCTCAACTACACGGTTATAGCCAGCGACAAATGTTCCGAAGAAAATGAAGAGAA 2580

KMP-4_16 (*r8A*) TTGGCACCTCAACTACACGGTTATAGCCAGTGACAAATGTTCCGAAGAAAATGAAGAGAA 2410

KMP-4_26 (*r8A*) TTGGCACCTCAACTACACGGTTATAGCCAGCGACAAATGTTCCGAAGAAAATGAAGAGAA 2410

KMP-4_3 (*r8A*) TTGGCACCTCAACTACACGGTTATAGCCAGCGACAAATGTTCCGAAGAAAATGAAGAGAA 2410

KMP-4_8 (*r9A*) TTGGCACCTCAACTACACGGTTATAGCCAGCGACAAATGTTCTGAAGAAAATGAAGAGAA 2576

KMP-4_13 (*r9B*) TTGGCACCTCAACTACACGGTTATAGCCAGCGACAAATGTTCTGAAGAAAATGAAGAGAA 2576

AY198374.1 TTGGCACCTCAACTACACGGTTATAGCCAGCGACAAATGTTCCGAAGAAAATGAAGAGAA 2576

KMP-8_35 (*s6B*) TTGGCACCTCAACTACACGATTATAGCCAGCGACAAATGTTCCGAAGAAAATGAAGAGAA 2573

KMP-8_24 (*s6A*) TTGGCACCTCAACTACACGATTATAGCCAGCGACAAATGTTCCGAAGAAAATGAAGAGAA 2573

KMP-8_5 (*s6A*) TTGGCACCTCAACTACACGATTATAGCCAGCGACAAATGTTCCGAAGAAAATGAAGAGAA 2573

KMP-8_46 (*s6A*) TTGGCACCTCAACTACACGATTATAGCCAGCGACAAATGTTCCGAAGAAAATGAAGAGAA 2573

*****.** *********..********** *********** *****************

KMP-5_2 (*r10A*) CTGTCCCCCGGATCCAGTGTTCTGGGATACTCTGGGCGACAATGTAATTAACATCGTGGA 2636

KMP-5_11 (*r10B*) CTGTCCCCCGGATCCAGTGTTCTGGGATACTCTGGGCGACAATGTAATTAACATCGTGGA 2636

KMP-5_16 (*r10C*) CTGTCCCCCGGATCCAGTGTTCTGGGATACTCTGGGCGACAATGTAATTAACATCGTGGA 2636

KMP-5_18 (*r10A*) CTGTCCCCCGGATCCAGTGTTCTGGGATACTCTGGGCGACAATGTAATTAACATCGTGGA 2636

KMP-7_3 (*r12C*) CTGTCCCCCGGATCCAGTGTTCTGGGATACTCTGGGCGACAATGTAATTAACATCGTGGA 2477

KMP-7_1 (*r12A*) CTGTCCCCCGGATCCAGTGTTCTGGGATACTCTGGGCGACAATGTAATTAACATCGTGGA 2636

KMP-7_4 (*r12D*) CTGTCCCCCGGATCCAGTGTTCTGGGATACTCTGGGCGACAATGTAATTAACATCGTGGA 2399

KMP-6_7 (*r12A*) CTGTCCCCCGGATCCAGTGTTCTGGGATACTCTGGGCGACAATGTAATTAACATCGTGGA 2636

KMP-7_2 (*r12B*) CTGTCCCCCGGATCCAGTGTTCTGGGATACTCTGGGCGACAATGTAATTAACATCGTGGA 2515

KMP-6_3 (*r11A*) CTGTCCCCCGGATCCAGTGTTCTGGGATACTCTGGGCGACAATGTAATTAACATCGTGGA 2636

KMP-6_5 (*r11B*) CTGTCCCCCGGATCCAGTGTTCTGGGATACTCTGCGCGACAATGTAATTAACATCGTGGA 2636

KMP-6_8 (*r11B*) CTGTCCCCCGGATCCAGTGTTCTGGGATACTCTGCGCGACAATGTAATTAACATCGTGGA 2636

KMP-8_3 (*s7*) CTGTCCCCCGGATCCAGTGTTCTGGGATACTCTGGGCGACAATGTAATTAACATCGTGGA 2633

KMP-4_20 (*r8B*) CTGTCCCCCGGATCCAGTGTTCTGGGATACTCTGGGCGACAATGTAATTAACATCGTGGA 2640

KMP-4_16 (*r8A*) CTGTCCCCCGGATCCAGTGTTCTGGGATACTCTGGGCGACAATGTAATTAACATCGTGGA 2470

KMP-4_26 (*r8A*) CCGTCCCCCGGATCCAGTGTTCTGGGATACTCTGGGCGACAATGTAATTAACATCGTGGA 2470

KMP-4_3 (*r8A*) CTGTCTCCCGGATCCAGTGTTCTGGGATACTCTGGGCGACAATGTAATTAACATCGTGGA 2470

KMP-4_8 (*r9A*) CTGTCCCCCGGATCCAGTGTTCTGGGATACTCTGCGCGACAATGTAATTAACATCGTGGA 2636

KMP-4_13 (*r9B*) CTGTCCCCCGGATCCAGTGTTCTGGGATACTCTGCGCGACAATGTAATTAACATCGTGGA 2636

AY198374.1 CTGTCCCCCGGATCCAGTGTTCTGGGATACTCTGGGCGACAATGTAATTAACATCGTGGA 2636

KMP-8_35 (*s6B*) CTGTCCCCCGGATCCAGTGTTCTGGGATACTCTGGGCGACAATGTAATTAACATCGTGGA 2633

KMP-8_24 (*s6A*) CTGTCCCCCGGATCCAGTGTTCTGGGATACTCTGGGCGACAATGTAATTAACATCGTGGA 2633

KMP-8_5 (*s6A*) CTGTCCCCCGGATCCAGTGTTCTGGGATACTCTGGGCGACAATGTAATTAACATCGTGGA 2633

KMP-8_46 (*s6A*) CTGTCCCCCGGATCCAGTGTTCTGGGATACTCTGGGCGACAATGTAATTAACATCGTGGA 2633

* *** **************************** *************************

KMP-5_2 (*r10A*) CATAAACAACAAGGTCCCGGCAGCAGACCTCAGTCGATTCAACGAAACGGTGTACATTTA 2696

KMP-5_11 (*r10B*) CATAAACAACAAGGTCCCGGCAGCAGACCTCAGTCGATTCAACGAAACGGTGTACATTTA 2696

KMP-5_16 (*r10C*) CATAAACAACAAGGTCCCGGCAGCAGACCTCAGTCGATTCAACGAAACGGTGTACATTTA 2696

KMP-5_18 (*r10A*) CATAAACAACAAGGTCCCGGCAGCAGACCTCAGTCGATTCAACGAAACGGTGTACATTTA 2696

KMP-7_3 (*r12C*) CATAAACAACAAGGTCCCGGCAGCAGACCTCAGTAGATTCAACGAAACGGTGTACATTTA 2537

KMP-7_1 (*r12A*) CATAAACAACAAGGTCCCGGCAGTAGACCTCAGTAGATTCAACGAAACGGTGTACATTTA 2696

KMP-7_4 (*r12D*) CATAAACAACAAGGTCCCGGCAGCAGACCTCAGTAGATTCAACGAAACGGTGTACATTTA 2459

KMP-6_7 (*r12A*) CATAAACAACAAGGTCCCGGCAGCAGACCTCAGTAGATTCAACGAAACGGTGTACATTTA 2696

KMP-7_2 (*r12B*) CATAAACAACAAGGTCCCGGCAGCAGACCTCAGTAGATTCAACGAAACGGTGTACATTTA 2575

KMP-6_3 (*r11A*) CATAAACAACAAGGTCCCGGCAGCAGACCTCAGTAGATTCAACGAAACGGTGTACATTTA 2696

KMP-6_5 (*r11B*) CATAAACAACAAGGTCCCGGCAGCAGACCTCAGTCGATTCAACGAAACGGTGTACATTTA 2696

KMP-6_8 (*r11B*) CATAAACAACAAGGTCCCGGCAGCAGACCTCAGTCGATTCAACGAAACGGTGTACATTTA 2696

KMP-8_3 (*s7*) CATAAACAACAAGGTCCCGGCAGCAGACCTCAGTCGATTCAACGAAACGGTGTACATTTA 2693

KMP-4_20 (*r8B*) CATAAACAACAAGGTCCCGGCAGCAGACCTCAGTCGATTCAACGAAACGGTGTACATTTA 2700

KMP-4_16 (*r8A*) CATAAACAACAAGGTCCCGGCAGCAGACCTCAGTCGATTCAACGAAACGGTGTACATTTA 2530

KMP-4_26 (*r8A*) CATAAACAACAAGGTCCCGGCAGCAGACCTCAGTCGATTCAACGAAACGGTGTACATTTA 2530

KMP-4_3 (*r8A*) CATAAACAACAAGGTCCCGGCAGCAGACCTCAGTCGATTCAACGAAACGGTGTACATTTA 2530

KMP-4_8 (*r9A*) CATAAACAACAAGGTCCCGGCAGCAGACCTCAGTCGATCAACGAAAACGGTGTACATTTA 2696

KMP-4_13 (*r9B*) CATAAACAACAAGGTCCCGGCAGCAGACCTCAGTCGATTCAACGAAACGGTGTACATTTA 2696

AY198374.1 CATAAACAACAAGGTCCCGGCAGCAGACCTCAGTCGATTCAACGAAACGGTGTACATTTA 2696

KMP-8_35 (*s6B*) CATAAACGACAAGGTCCCGGCAGCAGACCTCAGTCGATTCAACGAAACGGTGTACATTTA 2693

KMP-8_24 (*s6A*) CATAAACAACAAGGTCCCGGCAGCAGGCCTCAGTCGATTCAACGAAACGGTGTACATTTA 2693

KMP-8_5 (*s6A*) CATAAACAACAAGGTCCCGGCAGCAGACCTCAGTCGATTCAACGAAACGGTGTACATTTA 2693

KMP-8_46 (*s6A*) CATAAACAACAAGGTCCCGGCAGCAGACCTCAGTCGATTCAACGAAACGGTGTACGTTTA 2693

*******.*************** **.*******.*** .*. .***********.****

KMP-5_2 (*r10A*) TGAAAATGCACCCGATTTCACAAACGTGGTCAAGATATACTCCATCGACGAAGACAGAGA 2756

KMP-5_11 (*r10B*) TGAAAATGCACCCGATTTCACAAACGTGGTCAAGATATACTCCATCGACGAAGACAGAGA 2756

KMP-5_16 (*r10C*) TGAAAATGCACCCGATTTCACAAACGTGGTCAAGATATACTCCATCGACGAAGACAGAGA 2756

KMP-5_18 (*r10A*) TGAAAATGCACCCGATTTCACAAACGTGGTCAAGATATACTCCATCGACGAAGACAGAGA 2756

KMP-7_3 (*r12C*) TGAAAATGCACCCGATTTCACAAACGTGGTCAAGATATACTCCATCGACGAAGACAGAGA 2597

KMP-7_1 (*r12A*) TGAAAATGCACCCGATTTCACAAACGTGGTCAAGATATACTCCATCGACGAAGACAGAGA 2756

KMP-7_4 (*r12D*) TGAAAATGCACCCGATTTCACAAACGTGGTCAAGATATACTCCATCGACGAAGACAGAGA 2519

KMP-6_7 (*r12A*) TGAAAATGCACCCGATTTCACAAACGTGGTCAAGATGTACTCCATCGACGAAGACAGAGA 2756

KMP-7_2 (*r12B*) TGAAAATGCACCCGATTTCACAAACGTGGTCAAGATATACTCCATCGACGAAGACAGAGA 2635

KMP-6_3 (*r11A*) TGAAAATGCACCCGATTTCACAAACGTGGTCAAGATATACTCCATCGACGAAGACAGAGA 2756

KMP-6_5 (*r11B*) TGAAAATGCACCCGATTTCACAAACGTGGTCAAGATATACTCCATCGACGAAGACAGAGA 2756

KMP-6_8 (*r11B*) TGAAAATGCACCCGATTTCACAAACGTGGTCAAGATATACTCCATCGACGAAGACAGAGA 2756

KMP-8_3 (*s7*) TGAAAATGCACCCGATTTCACAAACGTGGTCAAGATATACTCTATCGACGAAGACAGAGA 2753

KMP-4_20 (*r8B*) TGAAAATGCACCCGATTTCACAAACGTGGTCAAGATATACTCCATCGACGAAGACAGAGA 2760

KMP-4_16 (*r8A*) TGAAAATGCACCCGATTTCACAAACGTGGTCAAGATATACTCCATCGACGAAGACAGAGA 2590

KMP-4_26 (*r8A*) TGAAAATGCACCCGATTTCACAAACGTGGTCAAGATATACTCCATCGACGAAGACAGAGA 2590

KMP-4_3 (*r8A*) TGAAAATGCACCCGATTTCACAAACGTGGTCAAGATATACTCCATCGACGAAGACAGAGA 2590

KMP-4_8 (*r9A*) TGAAAATGCACCCGATTTCACAAACGTGGTCAAGATATACTCCATCGACGAAGACAGAGA 2756

KMP-4_13 (*r9B*) TGAAAATGCACCCGATTTCACAAACGTGGTCAAGATATACTCCATCGACGAAGACAGAGA 2756

AY198374.1 TGAAAATGCACCCGATTTCACAAACGTGGTCAAGATATACTCCATCGACGAAGACAGAGA 2756

KMP-8_35 (*s6B*) TGAAAATGCACCCGATTTCACAAACGTGGTCAAGATATACTCCATCGACGAAGACAGAGA 2753

KMP-8_24 (*s6A*) TGAAAATGCACCCGATTTCACAAACGTGGTCAAGATATACTCCATCGACGAAGACAGAGA 2753

KMP-8_5 (*s6A*) TGAAAATGCACCCGATTTCACAAACGTGGTCAAGATATACTCCATCGACGAAGACAGAGA 2753

KMP-8_46 (*s6A*) TGAAAATGCACCCGATTTCACAAACGTGGTCAAGATATACTCCATCGACGAAGACAGAGA 2753

************************************.***** *****************

KMP-5_2 (*r10A*) CGAAATATATCACACGGTGCGGTACCAGATCAATTATGCTGTGAACCAGCGGCTGCGAGA 2816

KMP-5_11 (*r10B*) CGAAATATATCACACGGTGCGGTACCAGATCAATTATGCTGTGAACCAGCGGCTGCGAGA 2816

KMP-5_16 (*r10C*) CGAAATATATCACACGGTGCGGTACCAGATCAATTATGCTGTGAACCAGCGGCTGCGAGA 2816

KMP-5_18 (*r10A*) CGAAATATATCACACGGTGCGGTACCAGATCAATTATGCTGTGAACCAGCGGCTGCGAGA 2816

KMP-7_3 (*r12C*) CGAAATATATCACACGGTGCGGTACCAGATCAATTATGCTGTGAACCAGCGGCTGCGAGA 2657

KMP-7_1 (*r12A*) CGAAATGTATCACACGGTGCGGTACCAGATCAATTATGCTGTGAACCAGCGGCTGCGAGA 2816

KMP-7_4 (*r12D*) CGAAATATATCACACGGTGCGGTACCAGATCAATTATGCTGTGAACCAGCGGCTGCGAGA 2579

KMP-6_7 (*r12A*) CGAAATATATCACGCGGTGCGGTACCAGATCAATTATGCTGTGAACCAGCGGCTGCGAGA 2816

KMP-7_2 (*r12B*) CGAAATATATCACACGGTGCGGTACCAGATCAATTATGCTGTGAACCAGCGGCTGCGAGA 2695

KMP-6_3 (*r11A*) CGAAATATATCACACGGTGCGGTACCGGATCAATTATGCTGTGAACCAGCGGCTGCGAGA 2816

KMP-6_5 (*r11B*) CGAAATATATCACACGGTGCGGTACCAGATCAATTATGCTGTGAACCAGCGGCTGCGAGA 2816

KMP-6_8 (*r11B*) CGAAATATATCACACGGTGCGGTACCAGACCAATTATGCTGTGAACCAGCGGCTGCGAGA 2816

KMP-8_3 (*s7*) CGAAATATATCACACGGTGCGGTACCAGATCAATTATGCTGTGAACCAGCGGCTGCGAGA 2813

KMP-4_20 (*r8B*) CGATATATATCACACGGTGCGGTACCAGATCAATTATGCTGTGAACCAGCGGCTGCGAGA 2820

KMP-4_16 (*r8A*) CGAAATATATCACACGGTGCGGTACCAGATCAATTATGCTGTGAACCAGCGGCTGCGAGA 2650

KMP-4_26 (*r8A*) CGAAATATATCACACGGTGCGGTACCAGATCAATTATGCTGTGAACCAGCGGCTGCGAGA 2650

KMP-4_3 (*r8A*) CGAAATATATCACACGGTGCGGTACCAGATCAATTATGCTGTGAACCAGCGGCTGCGAGA 2650

KMP-4_8 (*r9A*) CGAAATATATCACACGGTGCGGTACCAGATCAATTATGCTGTGAACCAGCGGCTGCGAGA 2816

KMP-4_13 (*r9B*) CGAAATATATCACACGGTGCGGTACCAGATCAATTATGCTGTGAACCAGCGGCTGCGAGA 2816

AY198374.1 CGAAATATATCACACGGTGCGGTACCAGATCAATTATGCTGTGAACCAGCGGCTGCGAGA 2816

KMP-8_35 (*s6B*) CGAAATATATCACACGGTGCGGTACCAGATCAATCATGCTGTGAACCAGCGGCTGCGAGA 2813

KMP-8_24 (*s6A*) CGAAATATATCACACGGTGCGGTACCAGATCAATTATGCTGTGAACCAGCGGCTGCGAGA 2813

KMP-8_5 (*s6A*) CGAAATATATCACACGGTGCGGTACCAGATCAATTATGCTGTGAACCAGCGGCTGCGAGA 2813

KMP-8_46 (*s6A*) CGAAATATATCACACGGTGCGGTACCAGATCAATTATGCTGTGAACCAGCGGCTGCGAGA 2813

***:**.******.************.** **** *************************

KMP-5_2 (*r10A*) CTTCTTCGCCATAGACCTGGATTCAGGCCAGGTGTACGTGGAAAACACCAACAATGAGCT 2876

KMP-5_11 (*r10B*) CTTCCTCGCCATAGACCTGGATTCAGGCCAGGTGTACGTGGAGAACACCAACAATGAGCT 2876

KMP-5_16 (*r10C*) CTTCTTCGCCATAGACCTGGATTCAGGCCAGGTGTACGTGGAGAACACCAACAATGAGCT 2876

KMP-5_18 (*r10A*) CTTCTTCGCCATAGACCTGGATTCAGGCCAGGTGTACGTGGAGAACACCAACAATGAGCT 2876

KMP-7_3 (*r12C*) CTTCTTCGCCATAGACCTGGATTCAGGCCAGGTGTACGTGGAGAACACCAACGATGAGCT 2717

KMP-7_1 (*r12A*) CTTCTTCGCCATAGACCTGGATTCAGGCCAGGTGTACGTGGAGAACACCAACAATGAGCT 2876

KMP-7_4 (*r12D*) CTTCTTCGCCATAGACCTGGATTCAGGCCAGGTGTACGTGGAGAACACCAACAATGAGCT 2639

KMP-6_7 (*r12A*) CTTCTTCGCCATAGACCTGGATTCAGGCCAGGTGTACGTGGAGAACACCAACAATGAGCT 2876

KMP-7_2 (*r12B*) CTTCTTCGCCATAGACCTGGATTCAGGCCAGGTGTACGTGGAGAACACCAACAATGAGCT 2755

KMP-6_3 (*r11A*) CTTCTTCGCCATAGACCTGGATTCAGGCCAGGTGTACGTGGAGAACACCAACAATGAGCT 2876

KMP-6_5 (*r11B*) CTTCTTCGCCATAGACCTGGATTCAGGCCAGGTGTACGTGGAGAACACCAACAATGAGCT 2876

KMP-6_8 (*r11B*) CTTCTTCGCCATAGACCTGGATTCAGGCCAGGTGTACGTGGAGAACACCAACAATGAGCT 2876

KMP-8_3 (*s7*) CTTCCTCGCCATAGACCTGGATTCAGGCCAGGTGTACGTGGAGAACACCAACAATGAGCT 2873

KMP-4_20 (*r8B*) CTTCTTCGCCATAGACCTGGATTCAGGCCAGGTGTACGTGGAGAACACCAACAATGAGCT 2880

KMP-4_16 (*r8A*) CTTCTTCGCCATAGACCTGGATTCAGGCCAGGTGTACGTGGAGAACACCAACAATGAGCT 2710

KMP-4_26 (*r8A*) CTTCTTCGCCATAGACCTGGATTCAGGCCAGGTGTACGTGGAGAACACCAACAATGAGCT 2710

KMP-4_3 (*r8A*) CTTCTTCGCCATAGACCTGGATTCAGGCCAGGTGTACGTGGAGAACACCAACAATGAGCT 2710

KMP-4_8 (*r9A*) CTTCTTCGCCATAGACCTGGATTCAGGCCAGGTGTACGTGGAGAACACCAACAATGAGCT 2876

KMP-4_13 (*r9B*) CTTCTTCGCCATAGACCTGGATTCAGGCCAGGTGTACGTGGAGAACACCAACAATGAGCT 2876

AY198374.1 CTTCTTCGCCATAGACCTGGATTCAGGCCAGGTGTACGTGGAGAACACCAACAATGAGCT 2876

KMP-8_35 (*s6B*) CTTCTTCGCCATAGACCTGGATTCAGGCCAGGTGTACGTGGAGAACACCAACAATGAGCT 2873

KMP-8_24 (*s6A*) CTTCTTCGCCATAGACCTGGATTCAGGCCAGGTGTACGTGGAGAACACCAACAATGAGCT 2873

KMP-8_5 (*s6A*) CTTCTTCGCCATAGACCTGGATTCAGGCCAGGTGTACGTGGAGAACACCAACAATGAGCT 2873

KMP-8_46 (*s6A*) CTTCTTCGCCATAGACCTGGATTCAGGCCAGGTGTACGTGGAGAACACCAACAATGAGCT 2873

**** *************************************.*********.*******

KMP-5_2 (*r10A*) CCTGGATCGGGACAGAGGCGAGGACCAACACAGGATATTCATTAACCTCATTGACAACTT 2936

KMP-5_11 (*r10B*) CCTGGATCGGGACAGAGGCGAGGACCAACACAGGATATTCATTAACCTCATTGACAACTT 2936

KMP-5_16 (*r10C*) CCTGGATCGGGACAGAGGCGAGGACCAACACAGGATATTCATTAACCTCATTGACAACTT 2936

KMP-5_18 (*r10A*) CCTGGATCGGGACAGAGGCGAGGACCAACACAGGATATTCATTAACCTCATTGACAACTT 2936

KMP-7_3 (*r12C*) CCTGGATCGGGACAGGGGCGAAGACCAACACAGGATATTCATTAACCTCATTGACAACTT 2777

KMP-7_1 (*r12A*) CCTGGATCGGGACAGAGGCGAAGACCAACACAGGATATTCATTAACCTCATTGACAACTT 2936

KMP-7_4 (*r12D*) CCTGGATCGGGACAGAGGCGAAGACCAACACAGGATATTCATTAACCTCATTGACAACTT 2699

KMP-6_7 (*r12A*) CCTGGATCGGGACAGAGGCGAAGACCAACACAGGATATTCATTAACCTCATTGACAACTT 2936

KMP-7_2 (*r12B*) CCTGGATCGGGACAGAGGCGAAGACCAACACAGGATATTCATTAACCTCATTGACAACTT 2815

KMP-6_3 (*r11A*) CCTGGATCGGGACAGAGGCGAAGACCAACACAGGATATTCATTAACCTCATTGACAACTT 2936

KMP-6_5 (*r11B*) CCTGGATCGGGACAGAGGCGAAGACCAACACAGGATATTCATTAACCTCATTGACAACTT 2936

KMP-6_8 (*r11B*) CCTGGATCGGGACAGAGGCGAAGACCAACACAGGATATTCATTAACCTCATTGACAACCT 2936

KMP-8_3 (*s7*) CCTGGATCGGGACAGAGGCGAAGACCAACACAGGATATTCATTAACCTCATTGACAACTT 2933

KMP-4_20 (*r8B*) CCTGGATCGGGACAGAGGCGAGGACCAACACAGGATATTCATTAACCTCATTGACAACTT 2940

KMP-4_16 (*r8A*) CCTGGATCGGGACAGAGGCGAGGACCAACACAGGATATTCATTAACCTCATTGACAACTT 2770

KMP-4_26 (*r8A*) CCTGGATCGGGACAGAGGCGAGGACCAACACAGGATATTCATTAACCTCATTGACAACTT 2770

KMP-4_3 (*r8A*) CCTGGATCGGGACAGAGGCGAGGACCAACACAGGATATTCATTAACCTCATTGACAACTT 2770

KMP-4_8 (*r9A*) CCTGGATCGGGACAGAGGCGAAGACCAACACAGGATATTCATTAACCTCATTGACAACTT 2936

KMP-4_13 (*r9B*) CCTGGATCGGGACAGAGGCGAAGACCAACACAGGATATTCATTAACCTCATTGACAACTT 2936

AY198374.1 CCTGGATCGGGACAGAGGCGAAGACCAACACAGGATATTCATTAACCTCATTGACAACTT 2936

KMP-8_35 (*s6B*) CCTGGATCGGGACAGAGGCGAAGACCAACACAGGATATTCATTAACCTCATTGACAACTT 2933

KMP-8_24 (*s6A*) CCTGGATCGGGACAGAGGCGAAGACCAACACAGGATATTCATTAACCTCATTGACAACTT 2933

KMP-8_5 (*s6A*) CCTGGATCGGGACAGAGGCGAAGACCAACACAGGATATTCATTAACCTCATTGACAACTT 2933

KMP-8_46 (*s6A*) CCTGGATCGGGACAGAGGCGAAGACCAACACAGGATATTCATTAACCTCATTGACAACTT 2933

***************.*****.************************************ *

KMP-5_2 (*r10A*) TTATAGCGAAGGAGATGGAAATAGAAATGTAAACACTACAGAGGTGCTGGTGATACTATT 2996

KMP-5_11 (*r10B*) TCATAGCGAAGGAGATGGAAATAGAAATGTAAACACTACAGAGGTGCTGGTGATACTATT 2996

KMP-5_16 (*r10C*) TTATAGCGAAGGAGATGGAAATAGAAATGTAAACACTACAGAGGTGCTAGTGATACTATT 2996

KMP-5_18 (*r10A*) TTATAGCGAAGGAGATGGAAATAGAAATGTAAACACTACAGAGGTGCTGGTGGTACTATT 2996

KMP-7_3 (*r12C*) TTATAGCGAAGGAGATGGAAATAGAAATGTAAACACTACAGAGGTGCTGGTGATACTATT 2837

KMP-7_1 (*r12A*) TTATAGCGAAGGAGATGGAAATAGAAATGTAAACACTACAGAGGTGCTGGTGGTACTATT 2996

KMP-7_4 (*r12D*) TTATAGCGAAGGAGATGGAAATAGAAATGTAAACACTACAGAGGTGCTGGTGATACTATT 2759

KMP-6_7 (*r12A*) TTATAGCGAAGGAGATGGAAATAGAAATGTAAACACTACAGAGGTGCTGGTGATACTATT 2996

KMP-7_2 (*r12B*) TTATAGCGAAGGAGATGGAAATAGAAATGTAAACACTACAGAGGTGCTGGTGATACTATT 2875

KMP-6_3 (*r11A*) TTATAGCGAAGGAGATGGAAATAGAAATGTAAACACTACAGAGGTGCTGGTGATACTATT 2996

KMP-6_5 (*r11B*) TTATAGCGAAGGAGATGGAAATAGAAATGTAAACACTACAGAGGTGCTGGTGATACTATT 2996

KMP-6_8 (*r11B*) TTATAGCGAAGGAGATGGAAATAGAAATGTAAACACTACAGAGGTGCTGGTGATACTATT 2996

KMP-8_3 (*s7*) TTATAGCGAAGGAGATGGAAATAGAAATGTAAACACTACAGAGGTGCTGGTGATACTATT 2993

KMP-4_20 (*r8B*) TTATAGCGAAGGAGATGGAAATAGAAATGTAAACACTACAGAGGTGCTGGTGATACTATT 3000

KMP-4_16 (*r8A*) TTATAGCGAAGGAGATGGAAATAGAAATGTAAACACTACAGAGGTGCTGGTGACACTATT 2830

KMP-4_26 (*r8A*) TTATAGCGAAGGAGATGGAAATAGAAATGTAAACACTACAGAGGTGCTGGTGATACTATT 2830

KMP-4_3 (*r8A*) TTATAGCGAAGGAGATGGAAATAGAAATGTAAACACTACAGAGGTGCTGGTGATACTATT 2830

KMP-4_8 (*r9A*) TTATAGCGAAGGAGATGGAAATAGAAATGTAAACACTACAGAGGTGCTGGTGATACTATT 2996

KMP-4_13 (*r9B*) TTATAGCGAAGGAGATGGAAATAGAAATGTAAACACTACAGAGGTGCTGGTGATACTATT 2996

AY198374.1 TTATAGCGAAGGAGATGGAAATAGAAATGTAAACACTACAGAGGTGCTGGTGATACTATT 2996

KMP-8_35 (*s6B*) TTATAGCGAAGGAGATGGAAATAGAAATGTAAACACTACAGAGGTGCTGGTGATACTATT 2993

KMP-8_24 (*s6A*) TTATAGCGAAGGAGATGGAAATAGAAATGTAAACACTACAGAGGTGCTGGTGATACTATT 2993

KMP-8_5 (*s6A*) TTATAGCGAAGGAGATGGAAATAGAAATGTAAACACTACAGAGGTGCTGGTGATACTATT 2993

KMP-8_46 (*s6A*) TTATAGCGAAGGAGATGGAAATAGAAATGTAAACACTACAGAGGTGCTGGTGATACTATT 2993

* **********************************************.***. ******

KMP-5_2 (*r10A*) AGATGAAAATGACAACGCTCCTGAATTGCCGACTCCAGAAGAGCTGAGTTGGAGCATTTC 3056

KMP-5_11 (*r10B*) AGATGAAAATGACAACGCTCCTGAATTGCCGACTCCAGAAGAGCTGAGTTGGAGCATTTC 3056

KMP-5_16 (*r10C*) AGATGAAAATGACAACGCTCCTGAATTGCCGACTCCAGAAGAGCTGAGTTGGAGCATTTC 3056

KMP-5_18 (*r10A*) AGATGAAAATGACAACGCTCCTGAATTGCCGACTCCAGAAGAGCTGAGTTGGAGCATTTC 3056

KMP-7_3 (*r12C*) AGATGAGAATGACAACGCTCCTGAATTGCCGACTCCAGAAGAGCTGAGTTGGAGCATTTC 2897

KMP-7_1 (*r12A*) AGATGAGAATGACAACGCTCCTGAATTGCCGACTCCAGAAGAGCTGAGTTGGAGCATTTC 3056

KMP-7_4 (*r12D*) AGATGAGAATGACAACGCTCCTGAATTGCCGACTCCAGAAGAGCTGAGTTGGAGCATTTC 2819

KMP-6_7 (*r12A*) AGATGAGAATGACAACGCTCCTGAATTGCCGACTCCAGAAGAGCTGAGTTGGAGCATTTC 3056

KMP-7_2 (*r12B*) AGATGAGAATGACAACGCTCCTGAATTGCCGACTCCAGAAGAGCTGAGTTGGAGCATTTC 2935

KMP-6_3 (*r11A*) AGATGAGAATGACAACGCTCCTGAATTGCCGACTCCAGAAGAGCTGAGTTGGAGCATTTC 3056

KMP-6_5 (*r11B*) AGATGAGAATGACAACGCTCCTGAATTGCCGACCCCAGAAGAGCTGAGTTGGAGCATTTC 3056

KMP-6_8 (*r11B*) AGATGAGAATGACAACGCTCCTGAATTGCCGACTCCAGAAGAGCTGAGTTGGAGCATTTC 3056

KMP-8_3 (*s7*) AGATGAGAATGACAACGCTCCTGAATTGCCGACTCCAGAAGAGCTGAGTTGGAGCATTTC 3053

KMP-4_20 (*r8B*) AGATGAAAATGACAACGCTCCTGAATTGCCGACTCCAGAAGAGCTGAGTTGGAGCATTTC 3060

KMP-4_16 (*r8A*) AGATGAAAATGACAACGCTCCTGAATTGCCGACTCCAGAAGAGCTGAGTTGGAGCATTTC 2890

KMP-4_26 (*r8A*) AGATGAAAATGACAACGCTCCTGAATTGCCGACTCCAGAAGAGCTGAGTTGGAGCATTTC 2890

KMP-4_3 (*r8A*) AGATGAAAATGACAACGCTCCTGAATTGCCGACTCCAGAAGAGCTGAGTTGGAGCATTTC 2890

KMP-4_8 (*r9A*) AGATGAGAATGACAACGCTCCTGAATTGCCGACTCCAGAAGAGCTGAGTTGGAGCATTTC 3056

KMP-4_13 (*r9B*) AGATGAGAATGACAACGCTCCTGAATTGCCGACTCCAGAAGAGCTGAGCTGGAGCATTTC 3056

AY198374.1 AGATGAGAATGACAACGCTCCTGAATTGCCGACTCCAGAAGAGCTGAGTTGGAGCATTTC 3056

KMP-8_35 (*s6B*) AGATGAAAATGACAACGCTCCTGAATTGCCGACTCCAGAAGAGCTGAGTTGGAGCACTTC 3053

KMP-8_24 (*s6A*) AGATGAAAATGACAACGCTCCTGAATTGCCGACTCCAGAAGAGCTGAGTTGGAGCATTTC 3053

KMP-8_5 (*s6A*) AGATGAAAATGACAACGCTCCTGAATTGCCGACTCCAGAAGAGCTGAGTTGGAGCATTTC 3053

KMP-8_46 (*s6A*) AGATGAAAATGACAACGCTCCTGAATTGCCGACTCCAGAAGAGCTGAGTTGGAGCATTTC 3053

******.************************** ************** ******* ***

KMP-5_2 (*r10A*) CGAGAATTTACAAGAGGGTATAACACTCGATGGCGAACGCGATGTAATATACGCACCGGA 3116

KMP-5_11 (*r10B*) CGAGAATTTACAAGAGGGTATAACACTCGATGGCGAACGCGATGTAATATACGCACCGGA 3116

KMP-5_16 (*r10C*) CGAGAATTTACAAGAGGGTATAACACTCGATGGCGAACGCGATGTAATATACGCACCGGA 3116

KMP-5_18 (*r10A*) CGAGAATTTACAAGAGGGTATAACACTCGATGGCGAACGCGATGTAATATACGCACCGGA 3116

KMP-7_3 (*r12C*) CGAGAATTTACAAGAGGGTATAACACTCGATGGCGAAAGCGATGTGATATACGCACCGGA 2957

KMP-7_1 (*r12A*) CGAGAATTTACAAGAGGGTATAACACTCGATGGCGAAAGCGATGTGATATACGCACCGGA 3116

KMP-7_4 (*r12D*) CGAGAATTTACAAGAGGGTATAACACTCGATGGCGAAAGCGATGTGATATACGCACCGGA 2879

KMP-6_7 (*r12A*) CGAGAATTTACAAGAGGGTATAACACTCGATGGCGAAAGCGATGTGATATACGCACCGGA 3116

KMP-7_2 (*r12B*) CGAGAATTTACAAGAGGGTATAACACTCGATGGCGAAAGCGATGTGATATACGCACCGGA 2995

KMP-6_3 (*r11A*) CGAGAATTTACAAGAGGGTATAACACTCGATGGCGAAAGCGATGTGATATACGCACCGGA 3116

KMP-6_5 (*r11B*) CGAGAATTTACAAGAGGGTATAACACTCGATGGCGAAAGCGATGTGATATACGCACCGGA 3116

KMP-6_8 (*r11B*) CGAGAATTTACAAGAGGGTATAACACTCGATGGCGAAAGCGATGTGATATACGCACCGGA 3116

KMP-8_3 (*s7*) CGAGAATTTACAAGAGGGTATAACACTCGATGGCGAAAGCGATGTGATATACGCACCGGA 3113

KMP-4_20 (*r8B*) CGAGAATTTACAAGAGGGTATAACACTCGATGGCGAACGCGATGTAATATACGCACCGGA 3120

KMP-4_16 (*r8A*) CGAGAATTTACAAGAGGGTATAACACTCGATGGCGAACGCGATGTAATATACGCACCGGA 2950

KMP-4_26 (*r8A*) CGAGAATTTACAAGAGGGTATAACACTCGATGGCGAACGCGATGTAATATACGCACCGGA 2950

KMP-4_3 (*r8A*) CGAGAATTTACAAGAGGGTATAACACTCGATGGCGAACGCGATGTAATATACGCACCGGA 2950

KMP-4_8 (*r9A*) CGAGAATTTACAAGAGGGTATAACACTCGATGGCGAAAGCGATGTGATATACGCACCGGA 3116

KMP-4_13 (*r9B*) CGAGAATTTACAAGAGGGTATAACACTCGATGGCGAAAGCGATGTGATATACGCACCGGA 3116

AY198374.1 CGAGAATTTACAAGAGGGTATAACACTCGATGGCGAAAGCGATGTGATATACGCACCGGA 3116

KMP-8_35 (*s6B*) CGAGAATTTACAAGAGGGTATAACACTCGATGGCGAACGCGATGTAATATACGCACCGGA 3113

KMP-8_24 (*s6A*) CGAGAATTTACAAGAGGGTATAACACTCGATGGCGAACGCGATGTAATATACGCACCGGA 3113

KMP-8_5 (*s6A*) CGAGAATTTACAAGAGGGTATAACACTCGATGGCGAACGCGATGTAATATACGCACCGGA 3113

KMP-8_46 (*s6A*) CGAGAATTTACAAGAGGGTATAACACTCGATGGCGAACGCGATGTAATATACGCACCGGA 3113

*************************************.*******.**************

KMP-5_2 (*r10A*) TATAGACGAAGAGGACACGCCAAACTCTCACGTTGGCTACGCAATCCTGGCCATGACAGT 3176

KMP-5_11 (*r10B*) TATAGACGAAGAGGACACGCCAAACTCTCACGTTGGCTACGCAATCCTGGCCATGACAGT 3176

KMP-5_16 (*r10C*) TATAGACGAAGAGGACACGCCAAACTCTCACGTTGGCTACGCAATCCTGGCCATGACAGT 3176

KMP-5_18 (*r10A*) TATAGACGAAGAGGACACGCCAAACTCTCACGTTGGCTACGCAATCCTGGCCATGACAGT 3176

KMP-7_3 (*r12C*) TATAGACGAAGAGGACACGCCAAACTCTCACGTTGGCTACGCAATCCTGGCCATGACAGT 3017

KMP-7_1 (*r12A*) TATATACGAAGAGGACACGCCAAACTCTCACGTTGGCTACGCAATCCTGGCCATGACAGT 3176

KMP-7_4 (*r12D*) TATAGACGAAGAGGACACGCCAAACTCTCACGTTGGCTACGCAATCCTGGCCATGACAGT 2939

KMP-6_7 (*r12A*) TATAGACGAAGAGGACACGCCAAACTCTCACGTTGGCTACGCAATCCTGGCCATGACAGT 3176

KMP-7_2 (*r12B*) TATAGACGAAGAGGACACGCCAAACTCACACGTTGGCTACGCAATCCTGGCCATGACAGT 3055

KMP-6_3 (*r11A*) TATAGACGAAGAGGACACGCCAAACTCTCACGTTGGCTACGCAATCCTGGCCATGACAGT 3176

KMP-6_5 (*r11B*) TATAGACGAAGAGGACACGCCAAACTCTCACGTTGGCTACGCAATCCTGGCCATGACAGT 3176

KMP-6_8 (*r11B*) TATAGACGAAGAGGACACGCCAAACTCTCACGTTGGCTACGCAATCCTGGCCATGACAGT 3176

KMP-8_3 (*s7*) TATAGACGAAGAGGACACGCCAAACTCTCACGTTGGCTACGCAATCCTGGCCATGACAGT 3173

KMP-4_20 (*r8B*) TATAGACGAAGAGGACACGCCAAACTCTCACGTTGGCTACGCAATCCTGGCCATGACAGT 3180

KMP-4_16 (*r8A*) TATAGACGAAGAGGACACGCCAAACTCTCACGTTGGCTACGCAATCCTGGCCATGACAGT 3010

KMP-4_26 (*r8A*) TATAGACGAAGAGGACACGCCAAACTCTCACGTTGGCTACGCAATCCTGGCCATGACAGT 3010

KMP-4_3 (*r8A*) TATAGACGAAGAGGACACGCCAAACTCTCACGTTGGCTACGCAATCCTGGCCATGACAGT 3010

KMP-4_8 (*r9A*) TATAGACGAAGAGGACACGCCAAACTCTCACGTTGGCTACGCAATCCTGGCCATGACAGT 3176

KMP-4_13 (*r9B*) TATAGACGAAGAGGACACGCCAAACTCTCACGTTGGCTACGCAATCCTGGCCATGACAGT 3176

AY198374.1 TATAGACGAAGAGGACACGCCAAACTCTCACGTTGGCTACGCAATCCTGGCCATGACAGT 3176

KMP-8_35 (*s6B*) TATAGACGAAGAGGACACGCCAAACTCTCACGTTGGCTACGCAATCCTGGCCATAACAGT 3173

KMP-8_24 (*s6A*) TATAGACGAAGAGGACACGCCAAACTCTCACGTTGGCTACGCAATCCTGGCCATAACAGT 3173

KMP-8_5 (*s6A*) TATAGACGAAGAGGACACGCCAAACTCTCACGTTGGCTACGCAACCCTGGCCATAACAGT 3173

KMP-8_46 (*s6A*) TATAGACGAAGAGGACACGCCAAACTCTCACGTTGGCTACGCAATCCTGGCCATAACAGT 3173

**** **********************:**************** *********.*****

KMP-5_2 (*r10A*) CACCAATAGAGACCTGGGCACTGTTCCGAGACTTCTCAACATGCTGTCGCCTAACAACGT 3236

KMP-5_11 (*r10B*) CACCAATAGAGACCTGGACACTGTTCCGAGACTTCTCAACATGCTGTCGCCTAACAACGT 3236

KMP-5_16 (*r10C*) CACCAATAGAGACCTGGACACTGTTCCGAGACTTCTCAACATGCTGTCGCCTAACAACGT 3236

KMP-5_18 (*r10A*) CACCAATAGAGACCTGGACACTGTTCCGAGACTTCTCAACATGCTGTCGCCTAACAACGT 3236

KMP-7_3 (*r12C*) CACCAATAGAGACCTGGACACTGTTCCGAGACTTCTCAACATGCTGTCGCCTAACAACGT 3077

KMP-7_1 (*r12A*) CACCAATAGAGACCTGGACACTGTTCCGAGACTTCTCAACATGCTGTCGCCTAACAACGT 3236

KMP-7_4 (*r12D*) CACCAATAGAGACCTGGACACTGTTCCGAGACTTCTCAACATGCTGTCGCCTAACAACGT 2999

KMP-6_7 (*r12A*) CACCAATAGAGACCTGGACACTGTTCCGAGACTTCTCAACATGCTGTCGCCTAACAACGT 3236

KMP-7_2 (*r12B*) CACCAATAGAGACCTGGACACTGTTCCGAGACTTCTCAACATGCTGTCGCCTAACAACGT 3115

KMP-6_3 (*r11A*) CACCAATAGAGACCTGGACACTGTTCCGAGACTTCTCAACATGCTGTCGCCTAACAACGT 3236

KMP-6_5 (*r11B*) CACCAATAGAGACCTGGACACTGTTCCGAGACTTCTCAACATGCTGTCGCCTAACAACGT 3236

KMP-6_8 (*r11B*) CACCAATAGAGACCTGGACACTGTTCCGAGACTTCTCAACATGCTGTCGCCTAACAACGT 3236

KMP-8_3 (*s7*) CACCAATAGAGACCTGGACACTGTTCCGAGACTTCTCAACATGCTGTCGCCTAACAACGT 3233

KMP-4_20 (*r8B*) CACCAATAGAGACCTGGACACTGTTCCGAGACTTCTCAACATGCTGTCGCCTAACAACGT 3240

KMP-4_16 (*r8A*) CACCAATAGAGACCTGGACACTGTTCCGAGACTTCTCAACATGCTGTCGCCTAACAACGT 3070

KMP-4_26 (*r8A*) CACCAATAGAGACCTGGACACTGTTCCGAGACTTCTCAACATGCTGTCGCCTAACAACGT 3070

KMP-4_3 (*r8A*) CACCAATAGAGACCTGGACACTGTTCCGAGACTTCTCAACATGCTGTCGCCTAACAACGT 3070

KMP-4_8 (*r9A*) CACCAATAGAGACCTGGACACTGTTCCGAGACTTCTCAACATGCTGTCGCCTAACAACGT 3236

KMP-4_13 (*r9B*) CACCAATAGAGACCTGGACACTGTTCCGAGACTTCTCAACATGCTGTCGCCTAACAACGT 3236

AY198374.1 CACCAATAGAGACCTGGACACTGTTCCGAGACTTCTCAACATGCTGTCGCCTAACAACGT 3236

KMP-8_35 (*s6B*) CACCAATAGAGACCTGGACACTGCTCCGAGACTTCTGAACATGCTGTCGCCTAACAACGT 3233

KMP-8_24 (*s6A*) CACCAATAGAGACCTGGACACTGTTCCGAGACTTCTGAACATGCTGTCGCCTAACAACGT 3233

KMP-8_5 (*s6A*) CACCAATAGAGACCTGGACACTGTTCCGAGACTTCCGAACATGCTGTCGCCTAACAACGT 3233

KMP-8_46 (*s6A*) CACCAATAGAGACCTGGACACTGTTCCGAGACTTCTGAACATGCTGTCGCCTAACAACGT 3233

*****************.***** *********** ***********************

KMP-5_2 (*r10A*) AACCGGATTCCTCCAGACAGCAATGCCTTTAAGAGGATATTGGGGGACTTACGATATAAG 3296

KMP-5_11 (*r10B*) AACCGGATTCCTCCAGACAGCAATGCCTTTAAGAGGATATTGGGGGACTTACGATATAAG 3296

KMP-5_16 (*r10C*) AACCGGATTCCTCCAGACAGCAATGCCTTTAAGAGGATATTGGGGGACTTACGATATAAG 3296

KMP-5_18 (*r10A*) AACCGGATTCCTCCAGACAGCAATGCCTTTAAGAGGATATTGGGGGACTTACGATATAAG 3296

KMP-7_3 (*r12C*) AACCGGATTCCTTCAGACAGCAATGCCTTTGAGAGGATATTGGGGTACTTACGATATAAG 3137

KMP-7_1 (*r12A*) AACCGGATTCCTTCAGACAGCAATGCCTTTGAGAGGATATTGGGGTACTTACGATATAAG 3296

KMP-7_4 (*r12D*) AACCGGATTCCTTCAGACAGCAATGCCTTTGAGAGGATATTGGGGTACTTACGATATAAG 3059

KMP-6_7 (*r12A*) AACCGGATTCCTTCAGACAGCAATGCCTTTGAGAGGATATTGGGGTACTTACGATATAAG 3296

KMP-7_2 (*r12B*) AACCGGATTCCTTCAGACAGCAATGCCTTTGAGAGGATATTGGGGTACTTACGATATAAG 3175

KMP-6_3 (*r11A*) AACCGGATTCCTTCAGACAGCAATGCCTTTGAGAGGATATTGGG---------------- 3280

KMP-6_5 (*r11B*) AACCGGATTCCTCCAGACAGCAATGCCTTTGAGAGGATATTGGGGGACTTACGATATAAG 3296

KMP-6_8 (*r11B*) AACCGGATTCCTCCAGACAGCAATGCCTTTGAGAGGATATTGGGGGACTTACGATATAAG 3296

KMP-8_3 (*s7*) AACCGGATTCCTTCAGACAGCAATGCCTTTGAGAGGATATTGGGATACTTACGATATAAG 3293

KMP-4_20 (*r8B*) AACCGGATTCCTCCAGACAGCAATGCCTTTAAGAGGATATTGGGGGACTTACGATATAAG 3300

KMP-4_16 (*r8A*) AACCGGATTCCTCCAGACAGCAATGCCTTTAAGAGGATATTGGGGGACTTACGATATAAG 3130

KMP-4_26 (*r8A*) AACCGGATTCCTCCAGACAGCAATGCCTTTAAGAGGATATTGGGGGACTTACGATATAAG 3130

KMP-4_3 (*r8A*) AACCGGATTCCTCCAGACAGCAATGCCTTTAAGAGGATATTGGGGGACTTACGATATAAG 3130

KMP-4_8 (*r9A*) AACCGGATTCCTCCAGACAGCAATGCCTTTGAGAGGATATTGGGGGACTTACGATATAAG 3296

KMP-4_13 (*r9B*) AACCGGATTCCTCCAGACAGCAATGCCTTTGAGAGGATATTGGGGGACTTACGATATAAG 3296

AY198374.1 AACCGGATTCCTCCAGACAGCAATGCCTTTGAGAGGATATTGGGGGACTTACGATATAAG 3296

KMP-8_35 (*s6B*) AACCGGATTCCTCCAGACAGCAATGCCTTTAAGAGGATATTGGGGGACTTACGATATAAG 3293

KMP-8_24 (*s6A*) AACCGGATTCCTCCAGACAGCAATGCCTTTAAGAGGATATTGGGGGACTTACGATATAAG 3293

KMP-8_5 (*s6A*) AACCGGATTCCTCCAGACAGCAATGCCTTTAAGAGGATATTGGGGGACTTACGATATAAG 3293

KMP-8_46 (*s6A*) AACCGGATTCCTCCAGACAGCAATGCCTTTAAGAGGATATTGGGGGACTTACGATATAAG 3293

************ *****************.*************

KMP-5_2 (*r10A*) TATACCG----------------------------------------------------- 3303

KMP-5_11 (*r10B*) TATACTG----------------------------------------------------- 3303

KMP-5_16 (*r10C*) TATACTG----------------------------------------------------- 3303

KMP-5_18 (*r10A*) TATACTG----------------------------------------------------- 3303

KMP-7_3 (*r12C*) TATACTGGCGTTCGACCACGGTATTCCTCAGCAGATATCTCATGAGGTGTATGAATTGGA 3197

KMP-7_1 (*r12A*) TATACTGGCGTTCGACCACGGTATTCCTCAGCAGATATCTCATGAGGTGTATGAATTGGA 3356

KMP-7_4 (*r12D*) TATACTGGCGTTCGACCACGGTATTCCTCAGCAGATATCTCATGAGGTGTATGAATTGGA 3119

KMP-6_7 (*r12A*) TATACTGGCGTTCGACCACGGTATTCCTCAGCAGATATCTCATGAGGTGTATGAATTGGA 3356

KMP-7_2 (*r12B*) TATACTGGCGTTCGACCACGGTATTCCTCAGCAGATATCTCATGAGGTGTATGAATTGGA 3235

KMP-6_3 (*r11A*) -------GCGTTCGACCACGGTATTCCTCAGCAGATATCTCATGAGGTGTATGAATTGGA 3333

KMP-6_5 (*r11B*) TGTACTGGCGTTCGACCACGGTATTCCTCAGCAGATATCTCATGAGGTGTGTGAATTGGA 3356

KMP-6_8 (*r11B*) TGTACTGGCGTTCGACCACGGTATTCCTCAGCAGATATCTCATGAGGTGTATGGATTGGA 3356

KMP-8_3 (*s7*) TATACTGGCGTTCGACCACGGTATTCCTCAGCAGATATCTCATGAGGTGTATGAATTGGA 3353

KMP-4_20 (*r8B*) TATACTGGCGTTCGACCACGGTATTCCTCAGCAGATATCTCATGAGGTGTATGAATTGGA 3360

KMP-4_16 (*r8A*) TATACTGGCGTTCGACCACGGTATTCCTCAGCAGATATCTCATGAGGTGTATGAATTGGA 3190

KMP-4_26 (*r8A*) TATACTGGCGTTCGACCACGGTATTCCTCAGCAGATATCTCATGAGGTGTATGAATTGGA 3190

KMP-4_3 (*r8A*) TATACTGGCGTTCGACCACGGTATTCCTCAGCAGATATCTCCTGAGGTGTATGAATTGGA 3190

KMP-4_8 (*r9A*) TGTACCGGCGTTCGACCACGGTATTCCTCGGCAGATATCTCATGAGGTGTATGAATTGGA 3356

KMP-4_13 (*r9B*) TGTACTGGCGTTCGACCACGGTATTCCTCAGCAGATATCTCATGAGGTGTATGAATTGGA 3356

AY198374.1 TATACTGGCGTTCGACCACGGTATTCCTCAGCAGATATCTCATGAGGTGTATGAATTGGA 3356

KMP-8_35 (*s6B*) TATACTGGCGTTCGACCACGGTATTCCTCAGCAGATATCTCATGAGGTGTATGAATTGGA 3353

KMP-8_24 (*s6A*) TATACTGGCGTTCGACCACGGTATTCCTCAGCAGATATCTCATGAGGTGTATGAATTGGA 3353

KMP-8_5 (*s6A*) TATACTGGCGTTCGACCACGGTATTCCTCAGCAGATATCTCATGAGGTGTATGAATTGGA 3353

KMP-8_46 (*s6A*) TATACTGGCGTTCGACCACGGTATTCCTCAGCAGATATCTCATGAGGTGTATGAATTGGA 3353

KMP-5_2 (*r10A*) ------------------------------------------------------------ 3303

KMP-5_11 (*r10B*) ------------------------------------------------------------ 3303

KMP-5_16 (*r10C*) ------------------------------------------------------------ 3303

KMP-5_18 (*r10A*) ------------------------------------------------------------ 3303

KMP-7_3 (*r12C*) AATTCGACCTTACAATTACAATCCTCCCCAGTTCGTTTTTCCTGAATCCGGGTCGATTCT 3257

KMP-7_1 (*r12A*) AATTCGACCTTACAATTACAATCCTCCCCAGTTCGTTTTTCCTGAATCCGGGACGATTCT 3416

KMP-7_4 (*r12D*) AATTCGACCTTACAATTACAATCCTCCCCAGTTCGTTTTTCCTGAATCCGGGACGATTCT 3179

KMP-6_7 (*r12A*) AATTCGACCTTACAATTACAATCCTCCCCAGTTCGTTTTTCCTGAATCCGGGACGATTCT 3416

KMP-7_2 (*r12B*) AATTCGACCTTACAATTACAATCCTCCCCAGTTCGTTTTTCCTGAATCCGGGACGATTCT 3295

KMP-6_3 (*r11A*) AATTCGACCTTACAATTACAATCCTCCCCAGTTCGTTTTTCCTGAATCCGGGACGATTCT 3393

KMP-6_5 (*r11B*) AATTCGACCTTACAATTACAATCCTCCCCAGTTCGTTTTTCCTGAATCCGGGACGATTCT 3416

KMP-6_8 (*r11B*) AATTCGACCTTACAATTACAATCCTCCCCAGTTCGTTTTTCTTGAATCCGGGCCGATTCT 3416

KMP-8_3 (*s7*) AATTCGACCTTACAATTACAATCCTCCCCAGTTCGTTTTTCCTGAATCCGGGACGATTCT 3413

KMP-4_20 (*r8B*) AATTCGACCTTACAATTACAATCCTCCCCAGTTCGTTTTTCCTGAATCCGGGACGATTCT 3420

KMP-4_16 (*r8A*) AATTCGACCTTACAATTACAATCCTCCCCAGTTCGTTTTTCCTGAATCCGGGACGATTCT 3250

KMP-4_26 (*r8A*) AATTCGACCTTACAATTACAATCCTCCCCAGTTCGTTTTTCCTGAATCCGGGACGATTCT 3250

KMP-4_3 (*r8A*) AATTCGACCTTACAATTACAATCCTCCCCAGTTCGTTTTTCCTGAATCCGGGACGATTCT 3250

KMP-4_8 (*r9A*) AATTCGACCTTACAATTACAATCCTCCCCAGTTCGTTTTTCCTGAATCCGGGACGATTCT 3416

KMP-4_13 (*r9B*) AATTCGACCTTACAATTACAATCCTCCCCAGTTCGTTTTTCCTGAATCCGGGACGATTCT 3416

AY198374.1 AATTCGACCTTACAATTACAATCCTCCCCAGTTCGTTTTTCCTGAATCCGGGACGATTCT 3416

KMP-8_35 (*s6B*) AATTCGACCTTACAATTACAATCCTCCCCAGTTCGTTTTTCCTGAATCCGGGACGATTCT 3413

KMP-8_24 (*s6A*) AATCCGACCTTACAATTACAATCCTCCCCAGTTCGTTTTCCCTGAATCCGGGACGATTCT 3413

KMP-8_5 (*s6A*) AATTCGACCTTACAATTACAATCCTCCCCAGTTCGTTTTTCCTGAATCCGGGGCGATTCT 3413

KMP-8_46 (*s6A*) AATTCGACCTTACAATTACAATCCTCCCCAGTTCGTTTTTCCTGAATCCGGGACGATTCT 3413

KMP-5_2 (*r10A*) -------------GAACGCGCAGTGGTAAATAATGTTTTGTCACTTGTAAACGGTGACCC 3350

KMP-5_11 (*r10B*) -------------GAACGCGCAGTGGTAAATAATGTTTCGTCACTTGTAAACGGTGACCC 3350

KMP-5_16 (*r10C*) -------------GAACGCGCAGTGGTAAATAATGTTTTGTCACTTGTAAACGGTGACCC 3350

KMP-5_18 (*r10A*) -------------GAACGCGCAGTGGTAAATAATGTTTTGTCACTTGTAAACGGTGACCC 3350

KMP-7_3 (*r12C*) ACGACTGGCTTTGGAACGCGCAGTGGTAAATAATGTTTTGTCACTTGTAAGCGGTGACCC 3317

KMP-7_1 (*r12A*) ACGACTGGCTTTGGAACGCGCAGTGGTAAATAATGTTTTGTCACTTGTAAACGGTGACCC 3476

KMP-7_4 (*r12D*) ACGACTGGCTTTGGAACGCGCAGTGGTAAATAATGTTTTGTCACTTGTAAACGGTGACCC 3239

KMP-6_7 (*r12A*) ACGACTGGCTTTGGAACGCGCAGTGGTAAATAATGTTTTGTCACTTGTAAACGGTGACCC 3476

KMP-7_2 (*r12B*) ACGACTGGCTTTGGAACGCGCAGTGGTAAATAATGTTTTGTCACTTGTAAACGGTGACCC 3355

KMP-6_3 (*r11A*) ACGACTGGCTTTGGAACGCGCAGTGGTAAATAATGTTTTGTCACTTGTAAACGGTGACCC 3453

KMP-6_5 (*r11B*) ACGACTGGCTTTGGAACGCGCTGTGGTAAATAATGTATTGTCACTTGTAAACGGTGACCT 3476

KMP-6_8 (*r11B*) ACGACTGGCTTTGGAACGCGCTGTGGTAAATAATGTATTGTCACTTGTAAACGGTGACCT 3476

KMP-8_3 (*s7*) ACGACTGGCTTTGGAACGCGCAGTGGTAAATAATGTTTTGTCACTTGTAAACGGTGACCC 3473

KMP-4_20 (*r8B*) ACGACTGGCTTTGGAACGCGCAGTGGTAAATAATGTTTTGTCACTTGTAAACGGTGACCC 3480

KMP-4_16 (*r8A*) ACGACTGGCTTTGGAACGCGCAGTGGTAAATAATGTTTTGTCACTTGTAAACGGTGACCC 3310

KMP-4_26 (*r8A*) ACGACTGGCTTTGGAACGCACAGTGGTAAATAATGTTTTGTCACTTGTAAACGGTGACCC 3310

KMP-4_3 (*r8A*) ACGACTGGCTTTGGAACGCACAGTGGTAAATAATGTTTTGTCACTTGTAAACGGTGACCC 3310

KMP-4_8 (*r9A*) ACGACTGGCTTTGGAACGCGCTGTGGTAAATAATGTATTGTCACTTGTAAACGGTGACCT 3476

KMP-4_13 (*r9B*) ACGACTGGCTTTGGAACGCGCTGTGGTAAATAATGTATTGTCACTTGTAAACGGTGACCT 3476

AY198374.1 ACGACTGGCTTTGGAACGCGCAGTGGTAAATAATGTTTTGTCACTTGTAAACGGTGACCC 3476

KMP-8_35 (*s6B*) ACGACTGGCTTTGGAACGCGCAGTGGTAAATAATGTTTTGTCACTTGTAAACGGTGACCC 3473

KMP-8_24 (*s6A*) ACGACTGGCTTTGGAACGCGCAGTGGTAAATAATGTTTTGTCACTTGTAAACGGTGACCC 3473

KMP-8_5 (*s6A*) ACGACTGGCTTTGGAACGCGCAGTGGTAAATAATGTTTTGTCACTTGTAAACGGTGACCC 3473

KMP-8_46 (*s6A*) ACGACTGGCTTTGGAACGCGCAGTGGTAAATAATGTTTTGTCACTTGTAAACGGTGACCC 3473

******.*:**************:* ***********.********

KMP-5_2 (*r10A*) GTTAGACAGGATACAAGCAATTGACGACGATGGTCTTGATGCTGGCGTGGTGACTTTCGA 3410

KMP-5_11 (*r10B*) GTTAGACAGGATACAAGCAATTGACGACGATGGTCTTGATGCTGGCGTGGTGACTTTCGA 3410

KMP-5_16 (*r10C*) GTTAGACAGGATACAAGTAATTGACGACGATGGTCTTGATGCTGGCGTGGTGACTTTCGA 3410

KMP-5_18 (*r10A*) GTTAGACAGGATACAAGCAATTGACGACGATGGTCTTGATGCTGGCGTGGTGACTTTCGA 3410

KMP-7_3 (*r12C*) GTTAGACAGGATACAAGCAATTGACGACGATGGTCTTGATGCTGGCGTGGTGACTTTCGA 3377

KMP-7_1 (*r12A*) GTTAGACAGGATACAAGCAATTGACGACGATGGTCTTGATGCTGGCGTGGTGACTTTCGA 3536

KMP-7_4 (*r12D*) GTTAGACAGGATACAAGCAATTGACGACGATGGTCTTGATGCTGGCGTGGTGACTTTCGA 3299

KMP-6_7 (*r12A*) GTTAGACAGGATACAAGCAATTGACGACGATGGTCTTGATGCTGGCGTGGTGACTTTCGA 3536

KMP-7_2 (*r12B*) GTTAGACAGGATACAAGCAATTGACGACGATGGTCTTGATGCTGGCGTGGTGACTTTCGA 3415

KMP-6_3 (*r11A*) GTTAGACAGGATACAAGCAATTGACGACGATGGTCTTGATGCTGGCGTGGTGACTTTCGA 3513

KMP-6_5 (*r11B*) GTTAGACAGGATACAAGCAATTGACGACGATGGTCTCGATGCTGGCGTGGTGGCTTTCGA 3536

KMP-6_8 (*r11B*) GTTAGACAGGATACAAGCAATTGACGACGATGGTCTCGATGCTGGCGTGGTGACTTTCGA 3536

KMP-8_3 (*s7*) GTTAGACAGGATACAAGCAATTGACGACGATGGTCTTGATGCTGGCGTGGTGACTTTCGA 3533

KMP-4_20 (*r8B*) GTTAGACAGGATACAAGCAATTGACGACGATGGTCTTGATGCTGGCGTGGTGACTTTCGA 3540

KMP-4_16 (*r8A*) GTTAGACAGGATACAAGCAATTGACGACGATGGTCTTGATGCTGGCGTGGTGACTTTCGA 3370

KMP-4_26 (*r8A*) GTTAGACAGGATACAAGCAATTGACGACGATGGTCTTGATGCTGGCGTGGTGACTTTCGA 3370

KMP-4_3 (*r8A*) GTTAGACAGGATACAAGCAATTGACGACGATGGTCTTGATGCAGGCGTGGTGACTTTCGA 3370

KMP-4_8 (*r9A*) GTTAGACAGGATACAAGCAATTGACGACGATGGTCTTGATGCTGGCGTGGTGACTTTCGA 3536

KMP-4_13 (*r9B*) GTTAGACAGGATACAAGCAATTGACGACGATGGTCCCGATGCTGGCGTGGTGACTTTCGA 3536

AY198374.1 GTTAGACAGGATACAAGCAATTGACGACGATGGTCTTGATGCTGGCGTGGTGACTTTCGA 3536

KMP-8_35 (*s6B*) GTTAGACAGGTTACAAGCAATTGACGACGATGGTCTTGATGCTGGCGTGGTGACTTTCGA 3533

KMP-8_24 (*s6A*) GTTAGACAGGTTACAAGCAATTGACGACGATGGTCTTGATGCTGGCGTGGTGACTTTCGA 3533

KMP-8_5 (*s6A*) GTTAGACAGGTTACAAGCAATTGACGACGATGGTCTTGATGCTGGCGTGGTGACTTTCGA 3533

KMP-8_46 (*s6A*) GTTAGACAGGTTACAAGCAATTGACGACGATGGTCTTGATGCTGGCGTGGTGACTTTCGA 3533

**********:****** ***************** *****:*********.*******

KMP-5_2 (*r10A*) TATTGTTGGAGATGCTGATGCATCAAACTACTTCAGAGTAAATAATGATGGCGACAACTT 3470

KMP-5_11 (*r10B*) TATTGTTGGAGATGCTGATGCATCAAACTACTTCAGAGTAAATAATGATGGCGACAACTT 3470

KMP-5_16 (*r10C*) TATTGTTGGAGATGCTGATGCATCAAACTACTTCAGAGTAAATAATGATGGCGACAACTT 3470

KMP-5_18 (*r10A*) TATTGTTGGAGATGCTGATGCATCAAACTCCTTCAGAGTAAATAATGATGGCGACAACTT 3470

KMP-7_3 (*r12C*) TATTGTTGGAGATGCTGATGCATCAAACTACTTCAGAGTAAATAATGATGGCGACAACTT 3437

KMP-7_1 (*r12A*) TATCGTTGGAGATGCTGATGCATCAAACTACTTCAGAGTAAATAATGATGGCGACAACTT 3596

KMP-7_4 (*r12D*) TATTGTTGGAGATGCTGATGCATCAAACTACTTCAGAGTAAATAATGATGGCGACAACTT 3359

KMP-6_7 (*r12A*) TATTGTTGGAGATGCTGATGCATCAAACTACTTCAGAGTAAATAATGATGGCGACAACTT 3596

KMP-7_2 (*r12B*) TATTGTTGGAGATGCTGATGCATCAAACTACTTCAGAGTAAATAATGATGGCGACAACTT 3475

KMP-6_3 (*r11A*) TATTGTTGGAGATGCTGATGCATCAAACTACTTCAGAGTAAATAACGATGGCGACAACTT 3573

KMP-6_5 (*r11B*) TATTGTTGGAGATGCTGATGCATCAAACTACTTCAGAGTAAATAATGATGGCGACAACTT 3596

KMP-6_8 (*r11B*) TATTGTTGGAGATGCTGATGCATCAAACTACTTCAGAGTAAATAATGATGGCGACAACTT 3596

KMP-8_3 (*s7*) TATTGTTGGAGATGCTGATGCATCAAACTACTTCAGAGTAAATAATGATGGCGACAACTT 3593

KMP-4_20 (*r8B*) TATTGTTGGAGATGCTGATGCATCAAACTACTTCAGAGTAAATAATGATGGCGACAACTT 3600

KMP-4_16 (*r8A*) TATTGTTGGAGATGCTGATGCATCAAACTACTTCAGAGTAAATAATGATGGCGACAACTT 3430

KMP-4_26 (*r8A*) TATTGTTGGAGATGCTGATGCATCAAACTACTTCAGAGTAAATAATGATGGCGACAACTT 3430

KMP-4_3 (*r8A*) TATTGTTGGAGATGCTGATGCATCAAACTACTTCAGAGTAAATAATGATGGCGACAACTT 3430

KMP-4_8 (*r9A*) TATTGTTGGAGATGCTGATGCATCAAACTACTTCAGAGTAAATAATGATGGCGACAACTT 3596

KMP-4_13 (*r9B*) TATTGTTGGAGATGCTGATGCATCAAACTACTTCAGAGTAAATAATGATGGCGACAACTT 3596

AY198374.1 TATTGTTGGAGATGCTGATGCATCAAACTACTTCAGAGTAAATAATGATGGCGACAACTT 3596

KMP-8_35 (*s6B*) TATTGTTGGAGATGCTGATGCATCAAACTACTTCAGAGTAAATAATGATGGCGACAACTT 3593

KMP-8_24 (*s6A*) TATTGTTGGAGGTGCTGATGCATCAAACTACTTCAGAGTAAATAATGATGGCGACAACTT 3593

KMP-8_5 (*s6A*) TATTGTTGGAGATGCTGATGCATCAAACTACTTCAGAGTAAATAATGATGGCGACAACTT 3593

KMP-8_46 (*s6A*) TATTGTTGGAGATGCTGATGCATCAAACTACTTCAGAGTAAATAATGATGGCGACAACTT 3593

*** *******.*****************.*************** **************

KMP-5_2 (*r10A*) TGGAACCTTGTTGCTGACACAGGCGCTTCCTGAGGAAGGCAAGGAATTTGAGGTTACCAT 3530

KMP-5_11 (*r10B*) TGGAACCTTGTTGCTGACACAGGCGCTTCCTGAGGTAGGCAAGGAATTTGAGGTTACCAT 3530

KMP-5_16 (*r10C*) TGGAACCTTGTTGCTGACACAGGCGCTTCCTGAGGAAGGCAAGGAATTTGAGGTTACCAT 3530

KMP-5_18 (*r10A*) TGGAACCTTGTTGCTGACACAGGCGCTTCCTGAGGAAGGCAAGGAATTTGAGGTTACCAT 3530

KMP-7_3 (*r12C*) TGGAACCTTGTTGCTGACACAAGCGCTTCCTGAGGAAGGCAAGGAATTTGAGGTTACCAT 3497

KMP-7_1 (*r12A*) TGGAACCTTGTTGCTGACACAGGCGCTTCCTGAGGAAGGCAAGGAATTTGAGGTTACCAT 3656

KMP-7_4 (*r12D*) TGGAACCTTGTTGCTGACACAGGCGCTTCCTGAGGAAGGCAAGGAATTTGAGGTTACCAT 3419

KMP-6_7 (*r12A*) TGGAACCTTGTTGCTGACACAGGCGCTTCCTGAGGAAGGCAAGGAATTTGAGGTTACCAT 3656

KMP-7_2 (*r12B*) TGGAACCTTGTTGCTGACACAGGCGCTTCCTGAGGAAGGCAAGGAATTTGAGGTTACCAT 3535

KMP-6_3 (*r11A*) TGGAACCTTGTTGCTGACACAGGCGCTTCCTGAGGAAGGCAAGGAATTTGAGGTTACCAT 3633

KMP-6_5 (*r11B*) TGGAACCTTGTTGCTGACACAGGCGCTTCCTGAGGAAGGCAAGGAATTTGAGGTTACCAT 3656

KMP-6_8 (*r11B*) TGGAACCTTGTTGCTGACACAGGCGCTTCCTGAGGAAGGCAAGGAATTTGAGGTTACCAT 3656

KMP-8_3 (*s7*) TGGAACCTTGTTGCTGACACAGGCGCTTCCTGAGGAAGGCAAGGAATTTGAGGTTACCAT 3653

KMP-4_20 (*r8B*) TGGAACCTTGTTGCTGACACAGGCGCTTCCTGAGGAAGGCAAGGAATTTGAGGTTACCAT 3660

KMP-4_16 (*r8A*) TGGAACCTTGTTGCTGACACAGGCGCTTCCTGAGGAAGGCAAGGAATTTGAGGTTACCAT 3490

KMP-4_26 (*r8A*) TGGAACCTTGTTGCTGACACAGGCGCTTCCTGAGGAAGGCAAGGAATTTGAGGTTACCAT 3490

KMP-4_3 (*r8A*) TGGAACCTTGTTGCTGACACAGGCGCTTCCTGAGGAAGGCAAGGAATTTGAGGTTACCAT 3490

KMP-4_8 (*r9A*) TGGAACCTTGTTGCTGACACAGGCGCTTCCTGAGGAAGGCAAGGAATTTGAGGTTACCAT 3656

KMP-4_13 (*r9B*) TGGAACCTTGTTGCTGACACAGGCGCTTCCTG---------------------------- 3628

AY198374.1 TGGAACCTTGTTGCTGACACAGGCGCTTCCTGAGGAAGGCAAGGAATTTGAGGTTACCAT 3656

KMP-8_35 (*s6B*) TGGAACCTTGTTGCTGACACAGGCGCTTCCTGAGGAAGGCAAGGAATTTGGGGTTACCAT 3653

KMP-8_24 (*s6A*) TGGAACCTTGTTGCTGACACAGGCGCTTCCTGAGGAAGGCAAGGAATTTGAGGTTACCAT 3653

KMP-8_5 (*s6A*) TGGAACCTTGTTGCTGACACAGGCGCTTCCTGAGGAAGGCAAGGAATTTGAGGTTACCAT 3653

KMP-8_46 (*s6A*) TGGAACCTTGTTGCTGACACAGGCGCTTCCTGAGGAAGGCAAGGAATTTGAGGTTACCAT 3653

*********************.**********

KMP-5_2 (*r10A*) CCGGGCTACAGACGGCGGAACGGAACCTCGATCATATTCAACAGACTCCACTATAACAGT 3590

KMP-5_11 (*r10B*) CCGGGCTACAGACGGCGGAACGGAACCTCGATCATATTCAACAGACTCCACTATAACAGT 3590

KMP-5_16 (*r10C*) CCGGGCTACAGACGGCGGAACGGAACCTCGATCATATTCAACAGACTCCACTATAACAGT 3590

KMP-5_18 (*r10A*) CCGGGCTACAGACGGCGGAACGGAACCTCGATCATATTCAACAGACTCCACTATAACAGT 3590

KMP-7_3 (*r12C*) CCGGGCTACAGACGGCGGAACAGAACCTCGATCATATTCAACAGACTCCACTATAACAGT 3557

KMP-7_1 (*r12A*) CCGGGCTACAGACGGCGGAACAGAACCTCGATCATATTCAACAGACTCAACTATAACAGT 3716

KMP-7_4 (*r12D*) CCGGGCTACAGACGGCGGAACAGAACCTCGATCATATTCAACAGACTCCACTATAGCAGT 3479

KMP-6_7 (*r12A*) CCGGGCTACAGACGGCGGAACAGAACCTCGATCATATTCAACAGACTCCACTATAACAGT 3716

KMP-7_2 (*r12B*) CCGGGCTACAGACGGCGGAACAGAACCTCGATCATATTCAACAGACTCCACTATAACAGT 3595

KMP-6_3 (*r11A*) CCGGGCTACAGACGGCGGAACAGAACCTCGATCATATTCAACAGACTCCACTATAACAGT 3693

KMP-6_5 (*r11B*) CCGGGCTACAGACGGCGGAACAGAACCTCGATCATATTCAACAGACTCCACTATAACAGT 3716

KMP-6_8 (*r11B*) CCGGGCTACAGACGGCGGAACAGAACCTCGATCATATTCAACAGACTCCACTATAACAGT 3716

KMP-8_3 (*s7*) CCGGGCTACAGACGGCGGAACAGAACCTCGATCATATTCAACAGACTCCACTATAACAGT 3713

KMP-4_20 (*r8B*) CCGGGCTACAGACGGCGGAACGGAACCTCGATCATATTCAACAGACTCCACTATAACAGT 3720

KMP-4_16 (*r8A*) CCGGGCTACAGACGGCGGAACGGAACCTCGATCATATTCAACAGACTCCACTATAACAGT 3550

KMP-4_26 (*r8A*) CCGGGCTACAGACGGCGGAACGGAACCTCGATCATATTCAACAGACTCCACTATAACAGT 3550

KMP-4_3 (*r8A*) CCGGGCTACAGACGGCGGAACGGAACCTCGATCATATTCAACAGACTCCACTATAACAGT 3550

KMP-4_8 (*r9A*) CCGGGCTACAGACGGCGGAACGGAACCTCGATCATATTCAACAGACTCCACTATAACAGT 3716

KMP-4_13 (*r9B*) ------------------------------------------------------------ 3628

AY198374.1 CCGGGCTACAGACGGCGGAACAGAACCTCGATCATATTCAACAGACTCCACTATAACAGT 3716

KMP-8_35 (*s6B*) CCGGGCTACAGACGGCGGAATAGAACCTCGATCATATTCAACAGACTCCACTATAACAGT 3713

KMP-8_24 (*s6A*) CCGGGCTACAGACGGCGGAATAGAACCTCGATCATATTCAACAGACTCCACTATAACAGT 3713

KMP-8_5 (*s6A*) CCGGGCTACAGACGGCGGAATAGAACCTCGATCATATTCAACAGACTCCACTATAACAGT 3713

KMP-8_46 (*s6A*) CCGGGCTACAGACGGCGGAATAGAACCTCGATCATATTCAACAGACTCCACTATAACAGT 3713

KMP-5_2 (*r10A*) GCTCTTCGTTCCGACTTTGGGTGATCCGATCTTTCAAGATAACACCTACTCAGTAGCATT 3650

KMP-5_11 (*r10B*) GCTCTTCGTTCCGACTTTGGGTGATCCGATCTTTCAAGATAACACTTACTCAGTAGCATT 3650

KMP-5_16 (*r10C*) GCTCTTCGTTCCGACTTTGGGTGATCCGATCTTTCAAGATAACACTTACTCAGTAGCATT 3650

KMP-5_18 (*r10A*) GCTCTTCGTTCCGACTTTGGGTGATCCGATCTTTCAAGATAACACTTACTCAGTAGCATT 3650

KMP-7_3 (*r12C*) GCTCTTCGTTCCGACTTTGGGTGATCCGATCTTTCAAGATAACACTTACTCAGTAGCATT 3617

KMP-7_1 (*r12A*) GCTCTTCGTTCCGACTTTGGGTGATCCGATTTTTCAAGATAACACTTACTCAGTAGCATT 3776

KMP-7_4 (*r12D*) GCTCTTCGTTCCGACTTTGGGTGATCCGATCTTTCAAGATAACACTTACTCAGTAGCATT 3539

KMP-6_7 (*r12A*) GCTCTTCGTTCCGACTTTGGGTGATCCGATCTTTCAAGATAACACTTACTCAGTAGCATT 3776

KMP-7_2 (*r12B*) GCTCTTCGTTCCGACCTTGGGTGATCCGATCTTTCAAGATAACACTTACTCAGTAGCATT 3655

KMP-6_3 (*r11A*) GCTCTTCGTTCCGACTTTGGGTGATCCGATCTTTCAAGATAACACTTACTCAGTAGCATT 3753

KMP-6_5 (*r11B*) CCTCTTCGTTCCGACTTTGGGTAATCCGATCTTTCAAGATAACACTTACTCAGTAGCATT 3776

KMP-6_8 (*r11B*) CCTCTTCGTTCCGACTTTGGGTGATCCGATCTTTCAAGATAACACTTACTCAGTAGCATT 3776

KMP-8_3 (*s7*) GCTCTTCGTTCCGACTTTGGGTGATCCGATCTTTCAAGATAACACTTACTCAGTAGCATT 3773

KMP-4_20 (*r8B*) GCTCTTCGTTCCGACTTTGGGTGATCCGATCTTTCAAGATAACACTTACTCAGTAGCATT 3780

KMP-4_16 (*r8A*) GCTCTTCGTTCCGACTTTGGGCGATCCGATCTTTCAAGATAACACTTACTCAGTAGCATT 3610

KMP-4_26 (*r8A*) GCTCTTCGTTCCGACTTTGGGTGATCCGATCTTTCAAGATAACACTTACTCAGTAGCATT 3610

KMP-4_3 (*r8A*) GCTCTTCGTTCCGACTTTGGGTGATCCGATCTTTCAAGATAACACTTACTCAGTAGCATT 3610

KMP-4_8 (*r9A*) GCTCTTCGTTCCGACTTTGGGTGATCCGATCTTTCAAGATAACACTTACTCAGTAGCATT 3776

KMP-4_13 (*r9B*) ------------------------------------------------------------ 3628

AY198374.1 GCTCTTCGTTCCGACTTTGGGTGATCCGATCTTTCAAGATAACACTTACTCAGTAGCATT 3776

KMP-8_35 (*s6B*) GCTCTTCGTTCCGACTTTGGGTGATCCGATCTTTCAAGATAACACTTACTCAGTAGCATT 3773

KMP-8_24 (*s6A*) GCTCTTCGTTCCGACTTTGGGTGATCCGATCTTTCAAGATAACACTTACTCAGTAGCATT 3773

KMP-8_5 (*s6A*) GCTCTTCGTTCCGACTTTGGGTGATCCGATCTTTCAAGATAACACTTACTCAGTAGCGTT 3773

KMP-8_46 (*s6A*) GCTCTTCGTTCCGACTTTGGGTGATCCGATCTTTCAAGATAACACTTACTCAGTAGCATT 3773

KMP-5_2 (*r10A*) CT---------------------------------------------------------- 3652

KMP-5_11 (*r10B*) CT---------------------------------------------------------- 3652

KMP-5_16 (*r10C*) CT---------------------------------------------------------- 3652

KMP-5_18 (*r10A*) CT---------------------------------------------------------- 3652

KMP-7_3 (*r12C*) CTTTGAAAAAGAGGTTGGCTTGACTGAGAGGTTCTCGCTCCCACATGCAGAGGACCCTAA 3677

KMP-7_1 (*r12A*) CTTTGAAAAAGAGGTTGGCTTGACTGAGAGGTTCTCGCTCCCACATGCAGAGGACCCTAA 3836

KMP-7_4 (*r12D*) CTTTGAAAAAGAGGTTGGCTTGACTGAGAGGTTCTCGCTCCCACATGCAGAGGACCCTAA 3599

KMP-6_7 (*r12A*) CTTTGAAAAAGAGGTTGGCTTGACTGAGAGGTTCTCGCTCCCACATGCAGAGGACCCTAA 3836

KMP-7_2 (*r12B*) CTTTGAAAAAGAGGTTGGCTTGACTGAGAGGTTCTCGCTCCCACATGCAGAGGACCCTAA 3715

KMP-6_3 (*r11A*) CTTTGAAAAAGAGGTTGGCTTGACTGAGAGGTTCTCGCTCCCACATGCAGAGGACCCTAA 3813

KMP-6_5 (*r11B*) CTTTGAAAAAGAGGTTGGCTTGACTGAGAGGTTCTCGCTCCCACATGCAGAGGACCCTAA 3836

KMP-6_8 (*r11B*) CTTTGAAAAAGAGGTTGGCTTGACTGAGAGGTTCTCGCTCCCACATGCAGAGGACCCTAA 3836

KMP-8_3 (*s7*) CTTTGAAAAAGAGGTTGGCTTGACTGAGAGGTTCTCGCTCCCACATGCAGGGGACCCTAA 3833

KMP-4_20 (*r8B*) CTTTGAAAAAGAGGTTGGCTTGACTGAGAGGTTCTCGCTCCCACATGCAGAGGACCCTAA 3840

KMP-4_16 (*r8A*) CTTTGAAAAAGAGGTTGGCTTGACTGAGAGGTTCTCGCTCCCACATGCAGAGGACCCTAA 3670

KMP-4_26 (*r8A*) CTTTGAAAAAGAGGTTGGCTTGACTGAGAGGTTCTCGCTCCCACATGCAGAGGACCCTAA 3670

KMP-4_3 (*r8A*) CTTTGAAAAAGAGGTTGGCTTGACTGAGAGGTTCTCGCTCCCACATGCAGAGGACCCTAA 3670

KMP-4_8 (*r9A*) CTTTGAAAAAGAGGTTGGCTTGACTGAGAGGTTCTCGCTCCCACATGCAGAGGACCCTAA 3836

KMP-4_13 (*r9B*) ------------------------------------------------------------ 3628

AY198374.1 CTTTGAAAAAGAGGTTGGCTTGACTGAGAGGTTCTCGCTCCCACATGCAGAGGACCCTAA 3836

KMP-8_35 (*s6B*) CTTTGAAAAAGAGGTTGGCTTGACTGAGAGGTTCTCGCTCCCACATGCAGAGGACCCTAA 3833

KMP-8_24 (*s6A*) CTTTGAAAAAGAGGTTGGCTTGACTGAGAGGTTCTCGCTCCCACATGCAGAGGGCCCTAA 3833

KMP-8_5 (*s6A*) CTTTGAAAAAGAGGTTGGCTTGACTGAGAGGTTCTCGCTCCCACATGCAGAGGACCCTAG 3833

KMP-8_46 (*s6A*) CTTTGAAAAAGAGGTTGGCTTGACTGAGAGGTTCTCGCTCCCACATGCAGAGGACCCTAA 3833

KMP-5_2 (*r10A*) -----------------------------------------------TTGGTGGTGTGGA 3665

KMP-5_11 (*r10B*) -----------------------------------------------TTGGTGGTGTGGA 3665

KMP-5_16 (*r10C*) -----------------------------------------------TTGGTGGTGTGGA 3665

KMP-5_18 (*r10A*) -----------------------------------------------TTGGTGGTGTGGA 3665

KMP-7_3 (*r12C*) GAACAAACTCTGCACTGACGACTGTCACGATATTTACTACAGGATCTTTGGTGGTGTGGA 3737

KMP-7_1 (*r12A*) GAACAAACTCTGCACTGACGACTGTCACGATATTTACTACAGGATCTTTGGTGGTGTGGA 3896

KMP-7_4 (*r12D*) GAACAAACTCTGCACTGACGACTGTCACGATATTTACTACAGGATCTTTGGTGGTGTGGA 3659

KMP-6_7 (*r12A*) GAACAAACTCTGCACTGACGACTGTCACGATATTTACTACAGGATCTTTGGTGGTGTGGA 3896

KMP-7_2 (*r12B*) GAACAAACTCTGCACTGACGACTGTCACGATATTTACTACAGGATCTTTGGTGGTGTGGA 3775

KMP-6_3 (*r11A*) GAACAAACTCTGCACTGACGACTGTCACGATATTTACTACAGGATCTTTGGTGGTGTGGA 3873

KMP-6_5 (*r11B*) GAACAAACTCTGCACTGACGACTGTCACGATATTTACTACAGGATTTTTGGTGGTGTGGA 3896

KMP-6_8 (*r11B*) GAACAAACTCTGCACTGACGACTGTCACGATATTTACTACAGGATCTTTGGTGGTGTGGA 3896

KMP-8_3 (*s7*) GAACAAACTCTGCACTGACGACTGTCACGATATTTACTACAGGATCTTTGGTGGTGTGGA 3893

KMP-4_20 (*r8B*) GAACAAACTCTGCACTGACGACTGTCACGATATTTACTACAGGATCTTTGGTGGTGTGGA 3900

KMP-4_16 (*r8A*) GAACAAACTCTGCACTGACGACTGTCACGATATTTACTACAGGATCTTTGGTGGTGTGGA 3730

KMP-4_26 (*r8A*) GAACAAACTCTGCACTGACGACTGTCACGATATTTACTACAGGATCTTTGGTGGTGTGGA 3730

KMP-4_3 (*r8A*) GAACAAACTCTGCACTGACGACTGTCACGATATTTACTACAGGATCTTTGGTGGTGTGGA 3730

KMP-4_8 (*r9A*) GAACAAACTCTGCACTGACGACTGTCACGATATTTACTACAGGATCTTTGGTGGTGTGGA 3896

KMP-4_13 (*r9B*) ------------------------------------------------------------ 3628

AY198374.1 GAACAAACTCTGCACTGACGACTGTCACGATATTTACTACAGGATCTTTGGTGGTGTGGA 3896

KMP-8_35 (*s6B*) GAACAAACTCTGCACTGACGACTGTCACGATATTTACTACAGGATCTTTGGTGGTGTGGA 3893

KMP-8_24 (*s6A*) GAACAAACTCTGCACTGACGACTGTCACGATATTTACTACAGGATCTTTGGTGGTGTGGA 3893

KMP-8_5 (*s6A*) GAACAAACTCTGCACTGACGACTGTCACGATATTTACTACAGGATCTTTGGTGGTGTGGA 3893

KMP-8_46 (*s6A*) GAACAAACTCTGCACTGACGACTGTCACGATATTTACTACAGGATCTTTGGTGGTGTGGA 3893

KMP-5_2 (*r10A*) TTACGAGCCATTTGACCTGGACCCGGTGACGAACGTGATCTTCCTGAAATCAGAACTAGA 3725

KMP-5_11 (*r10B*) TTACGAGCCATTTGACCTGGACCCGGTGACGAACGTGATCTTCCTGAAATCAGAACTAGA 3725

KMP-5_16 (*r10C*) TTACGAGCCATTTGACCTGGACCCGGTGACGAACGTGATCTTCCTGAAATCAGAACTAGA 3725

KMP-5_18 (*r10A*) TTACGAGCCATTTGACCTGGACCCGGTGACGAACGTGATCTTCCTGAAATCAGAACTAGA 3725

KMP-7_3 (*r12C*) TTACGAGCCATTTGACCTGGACCCGGTGACGAACGTGATCTTCCTGAAATCAGAACTAGA 3797

KMP-7_1 (*r12A*) TTACGAGCCATTTGACCTGGACCCGGTGACGAACGTGATCTTCCTGAAATCAGAACTAGA 3956

KMP-7_4 (*r12D*) TTACGAGCCATTTGACCTGGACCCGGTGACGAACGTGATCTTCCTGAAATCAGAACTAGA 3719

KMP-6_7 (*r12A*) TTACGAGCCATTTGACCTGGACCCGGTGACGAACGTGATCTTCCTGAAATCAGAACTAGA 3956

KMP-7_2 (*r12B*) TTACGAGCCATTTGACCTGGACCCGGTGACGAACGTGATCTTCCTGAAATCAGAACTAGA 3835

KMP-6_3 (*r11A*) TTACGAGCCATTTGACCTGGACCCGGTGACGAACGTGATCTTCCTGAAATCAGAACTAGA 3933

KMP-6_5 (*r11B*) TTACGAGCCATTTGACCTGGACCCGGTGACGAACGTGATCTTCCTGAAATCAGAACTGGA 3956

KMP-6_8 (*r11B*) TTACGAGCCATTTGACCTGGACCCGGTGACGAACGTGATCTTCCTGAAATTAGAACTGGA 3956

KMP-8_3 (*s7*) TTACGAGCCATTTGACCTGGACCCGGTGACGAACGTGATCTTCCTGAAATCAGAACTAGA 3953

KMP-4_20 (*r8B*) TTACGAGCCATTTGACCTGGACCCGGTGACGAACGTGATCTTCCTGAAATCAGAACTAGA 3960

KMP-4_16 (*r8A*) TTACGAGCCATTTGACCTGGACCCGGTGACGAACGTGATCTTCCTGAAATCAGAACTAGA 3790

KMP-4_26 (*r8A*) TTACGAGCCATTTGACCTGGACCCGGTGACGAACGTGATCTTCCTGAAATCAGAACTAGA 3790

KMP-4_3 (*r8A*) TTACGAGCCATTTGACCTGGACCCGGTGACGAACGTGATCTTCCTGAAATCAGAACTAGA 3790

KMP-4_8 (*r9A*) TTACGAGCCATTTGACCTGGACCCGGTGACGAACGCGATCTTCCTGAAATCAGAACTAGA 3956

KMP-4_13 (*r9B*) ------------------------------------------------------------ 3628

AY198374.1 TTACGAGCCATTTGACCTGGACCCGGTGACGAACGTGATCTTCCTGAAATCAGAACTAGA 3956

KMP-8_35 (*s6B*) TTACGAGCCATTTGACCTGGACCCGGTGACGAACGTGATCTTCCTGAAATCAGAACTAGA 3953

KMP-8_24 (*s6A*) TTACGAGCCATTTGACCTGGACCCGGTGACGAACGTGATCTTCCTGAAATCAGAACTAGA 3953

KMP-8_5 (*s6A*) TTACGAGCCATTTGACCTGGACCCGGTGACGAACGTGATCTTCCTGAAATCAGAACTAGA 3953

KMP-8_46 (*s6A*) TTACGAGCCATTTGACCTGGACCCGGTGACGAACGTGATCTTCCTGAAATCAGAACTAGA 3953

KMP-5_2 (*r10A*) CCGGGAGACCACTGCTACGCATGTGGTGCAAGTGGCAGCCAGTAATTCGCCCACAGGAGG 3785

KMP-5_11 (*r10B*) CCGGGAGACCACTGCTACGCATGTGGTGCAAGTGGCAGCCAGTAATTCGCCCACAGGAGG 3785

KMP-5_16 (*r10C*) CCGGGAGACCACTGCTACGCATGTGGTGCAAGTGGCAGCCAGTAATTCGCCCACAGGAGG 3785

KMP-5_18 (*r10A*) CCGGGAGACCACTGCTACGCATGTGGTGCAAGTGGCAGCCAGTAATTCGCCCACAGGAGG 3785

KMP-7_3 (*r12C*) CCGGGAGACCACTGCTACGCATGTGGTGCAAGTGGCAGCCAGTAATTCGCCCACAGGAGG 3857

KMP-7_1 (*r12A*) CCGGGAGACCACTGCTACGCATGTGGTGCAAGTGGCAGCCAGTAATTCGCCCACAGGAGG 4016

KMP-7_4 (*r12D*) CCGGGAGACCACTGCTACGCATGTGGTGCAAGTGGCAGCCAGTAATTCGCCCACAGGAGG 3779

KMP-6_7 (*r12A*) CCGGGAGACCACTGCTACGCATGTGGTGCAAGTGGCAGCCAGTAATTCGCCCACAGGAGG 4016

KMP-7_2 (*r12B*) CCGGGAGACCACTGCTACGCATGTGGTGCAAGTGGCAGCCAGTAATTCGCCCACAGGAGG 3895

KMP-6_3 (*r11A*) CCGGGAGACCACTGCTACGCATGTGGTGCAAGTGGCAGCCAGTAATTCGCCCACAGGAGG 3993

KMP-6_5 (*r11B*) CCGAGAGACCACTGCCACGCATGTGGTTCAAGTGGCTGCCAGTAATTCGCCCACAGGAGG 4016

KMP-6_8 (*r11B*) CCGAGAGACCACTGCCACGCATGTGGTTCAAGTGGCTGCCAGTAATTCGCCCACAGGAGG 4016

KMP-8_3 (*s7*) CCGGGAGACCACTGCTACGCATGTGGTGCAAGTGGCAGCCAGTAATTCGCCCACAGGAGG 4013

KMP-4_20 (*r8B*) CCGGGAGACCACTGCTACGCATGTGGTGCAAGTGGCAGCCAGTAATTCGCCCACAGGAGG 4020

KMP-4_16 (*r8A*) CCGGGAGACCACTGCTACGCATGTGGTGCAAGTGGCAGCCAGTAATTCGCCCACAGGAGG 3850

KMP-4_26 (*r8A*) CCGGGAGACCACTGCTACGCATGTGGTGCAAGTGGCAGCCAGTAACTCGCCCACAGGAGG 3850

KMP-4_3 (*r8A*) CCGGGAGACCACTGCTACGCATGTGGTGCAAGTGGCAGCCAGTAATTCGCCCACAGGAGG 3850

KMP-4_8 (*r9A*) CCGGGAGACCACTGCTACGCATGTGGTGCAAGTGGCAGCCAGTAATTCGCCCACAGGAGG 4016

KMP-4_13 (*r9B*) ------------------------------------------------------------ 3628

AY198374.1 CCGGGAGACCACTGCTACGCATGTGGTGCAAGTGGCAGCCAGTAATTCGCCCACAGGAGG 4016

KMP-8_35 (*s6B*) CCGGGAGACCACTGCCACGCATGTGGTGCAAGTGGCAGCCAGCAATTCGCCCACAGGAGG 4013

KMP-8_24 (*s6A*) CCGGGAGACCACTGCCACGCATGTGGTGCAAGTGGCAGCCAGTAATTCGCCCACAGGAGG 4013

KMP-8_5 (*s6A*) CCGGGAGACCACTGCCACGCATGTGGTGCAAGTGGCAGCCAGTAATTCGCCCACAGGAGG 4013

KMP-8_46 (*s6A*) CCGGGAGACCACTGCCACGCATGTGGTGCAAGTGGCAGCCAGTAATTCGCCCACAGGAGG 4013

KMP-5_2 (*r10A*) CGGAATACCACTCCCTGGGTCTCTTCTCACCGTCACTGTCACTGTACGAGAAGCGGATCC 3845

KMP-5_11 (*r10B*) CGGAATACCACTCCCTGGGTCTCTTCTCACCGTCACTGTCACTGTACGAGAAGCGGATCC 3845

KMP-5_16 (*r10C*) CGGAATACCACTCCCTGGGTCTCTTCTCACCGTCACTGTCACTGTACGAGAAGCGGATCC 3845

KMP-5_18 (*r10A*) CGGAATGCCACTCCCTGGGTCTCTTCTCACCGTCACTGTCACTGTACGAGAAGCGGATCC 3845

KMP-7_3 (*r12C*) CGGAATACCACTCCCTGGGTCTCTTCTCACCGTCACTGTCACTGTACGAGAAGCGGATCC 3917

KMP-7_1 (*r12A*) CGGAATACCACTCCCTGGGTCTCTTCTCACCGTCACTGTCACTGTACGAGAAGCGGATCC 4076

KMP-7_4 (*r12D*) CGGAATACCACTCCCTGGGTCTCTTCTCACCGTCACTGTCACTGTACGAGAAGCGGATCC 3839

KMP-6_7 (*r12A*) CGGAATACCACTCCCTGGGTCTCTTCTCACCGTCACTGTCACTGTACGAGAAGCGGATCC 4076

KMP-7_2 (*r12B*) CGGAATACCACTCCCTGGGTCTCTTCTCACCGTCACTGTCACTGTACGAGAAGCGGATCC 3955

KMP-6_3 (*r11A*) CGGAATACCACTCCCTGGGTCTCTTCTCACCGTCACTGTCACTGTACGAGAAGCGGATCC 4053

KMP-6_5 (*r11B*) CGGAATACCACTCCCTGGGTCTCTTCTCACCGTCACTGTCACTGTACGAGAAGCGGATCC 4076

KMP-6_8 (*r11B*) CGGAATACCACTCCCTGGGTCTCTTCTCACCGTCACTGTCACTGTACGAGAAGCGGATCC 4076

KMP-8_3 (*s7*) CGGAATACCACTCCCTGGGTCTCTTCTCACCGTCACTGTCACTGTACGAGAAGCGGATCC 4073

KMP-4_20 (*r8B*) CGGAATACCACTCCCTGGGTCTCTTCTCACCGTCACTGTCACTGTACGAGAAGCGGATCC 4080

KMP-4_16 (*r8A*) CGGAATACCACTCCCTGGGTCTCTTCTCGCCGTCACTGTCACTGTACGAGAAGCGGATCC 3910

KMP-4_26 (*r8A*) CGGAATACCACTCCCTGGGTCTCTTCTCACCGTCACTGTCACTGTACGAGAAGCGGATCC 3910

KMP-4_3 (*r8A*) CGGAATACCACTCCCTGGGTCTCTTCTCACCGTCACTGTCACTGTACGAGAAGCGGATCC 3910

KMP-4_8 (*r9A*) CGGAATACCACTCCCTGGGTCTCTTCTCACCGTCACTGTCACTGTACGAGAAGCGGATCC 4076

KMP-4_13 (*r9B*) ------------------------------------------------------------ 3628

AY198374.1 CGGAATACCACTCCCTGGGTCTCTTCTCACCGTCACTGTCACTGTACGAGAAGCGGATCC 4076

KMP-8_35 (*s6B*) CGGAATACCACTCCCTGGGTCTCTTTTCACCGTCACTGTCACTGTACGAGAAGCGGATCC 4073

KMP-8_24 (*s6A*) CGGAATACCACTCCCTGGGTCTCTTCTCACCGTCACTGTCACTGCACGAGAAGCGGATCC 4073

KMP-8_5 (*s6A*) CGGAATACCACTCCCTGGGTCTCTTCTCACCGTCACTGTCACTGTACGAGAAGCGGATCC 4073

KMP-8_46 (*s6A*) CGGAATACCACTCCCTGGGTCTCTTCTCACCGTCACTGTCACTGTACGAGAAGCGGATCC 4073

KMP-5_2 (*r10A*) ACGGCCTGTGTTCGAGCAGCGTCTGTACACGGCTGGCATCTCCACTTCCGATAACATCAA 3905

KMP-5_11 (*r10B*) ACGGCCTGTGTTCGAGCAGCGTCTGTACACGGCTGGCATTTCCACTTCCGATAACATCAA 3905

KMP-5_16 (*r10C*) ACGGCCTGTGTTCGAGCAGCGTCTGTACACGGCTGGCATTTCCACTTCCGATAACATCAA 3905

KMP-5_18 (*r10A*) ACGGCCTGTGTTCGAGCAGCGTCTGTACACGGCTGGCATTTCCACTTCCGATAACATCAA 3905

KMP-7_3 (*r12C*) ACGGCCTGTGTTCGAGCAGCGTCTGTACACGGCTGGCATTTCCACTTCCGATAACATCAA 3977

KMP-7_1 (*r12A*) ACGGCCTGTGTTCGAGCAGCGTCTGTACACGGCTGGCATTTCCACTTCCGATAACATCAA 4136

KMP-7_4 (*r12D*) ACGGCCTGTGTTCGAGCAGCGTCTGTGCACGGCTGGCATTTCCACTTCCGATAACATCAA 3899

KMP-6_7 (*r12A*) ACGGCCTGTGTTCGAGCAGCGTCTGTACACGGCTGGCATTTCCACTTCCGATAACATCAG 4136

KMP-7_2 (*r12B*) ACGGCTTGTGTTCGAGCAGCGTCTGTACACGGCTGGCATTTCCACTTCCGATAACATCAA 4015

KMP-6_3 (*r11A*) ACGGCCTGTGTTCGAGCAGCGTCTGTACACGGCTGGCATTTCCACTTCCGATAACATCAA 4113

KMP-6_5 (*r11B*) ACGGCCTGTGTTCGAGCAGCGTCTGTACACGGCTGGCATTTCCACTTCCGATAACATCAA 4136

KMP-6_8 (*r11B*) ACGGCCTGTGTTCGAGCAGCGTCTGTACACGGCTGGCATTTCCACTTCCGATAACATCAA 4136

KMP-8_3 (*s7*) ACGGCCTGTGTTCGGGCAGCGTCTGTACACGGCTGGCATTTCCACTTCCGATAACATCAA 4133

KMP-4_20 (*r8B*) ACGGCCTGTGTTCGAGCAGCGTCTGTACACGGCTGGCATTTCCACTTCCGATAACATCAA 4140

KMP-4_16 (*r8A*) TCGGCCTGTGTTCGAGCAGCGTCTGTACACGGCTGGCATTTCCACTTCCGATAACATCAA 3970

KMP-4_26 (*r8A*) ACGGCCTGTGTTCGAGCAGCGTCTGTACACGGCTGGCATTTCCACTTCCGATAACATCAA 3970

KMP-4_3 (*r8A*) ACGGCCTGTGTTCGAGCAGCGTCTGTACACGGCTGGCATTTCCACTTCCGATAACATCAA 3970

KMP-4_8 (*r9A*) ACGGCCTGTGTTCGAGCAGCGTCTGTACACGGCTGGCATTTCCACTTCCGATAACATCAA 4136

KMP-4_13 (*r9B*) ------------------------------------------------------------ 3628

AY198374.1 ACGGCCTGTGTTCGAGCAGCGTCTGTACACGGCTGGCATTTCCACTTCCGATAACATCAA 4136

KMP-8_35 (*s6B*) ACGGCCTGTGTTCGAGCAGCGTCTGTACACGGCTGGCATTTCCACTTCCGATAACATCAA 4133

KMP-8_24 (*s6A*) ACGGCCTGTGTTCGAGCAGCGTCTGTACACGGCTGGCATTTCCACTTCCGATAACATCAA 4133

KMP-8_5 (*s6A*) ACGGCCTGTGTTCGAGCAGCGTCTGTACACGGCTGGCATTTCCACTTCCGATAACATCAA 4133

KMP-8_46 (*s6A*) ACGGCCTGTGTTCGAGCAGCGTCTGTACACGGCTGGCATTTCCACTTCCGATAACATCAA 4133

KMP-5_2 (*r10A*) CAGGGAACTACTCACCGTTCGTGCAACTCATTCCGAAAACGCACAATTGACATA-TACCA 3964

KMP-5_11 (*r10B*) CAGGGAACTACTCACCGTTCGT-------------------------------------- 3927

KMP-5_16 (*r10C*) CAGGGAACTACTCACCGTTCGT-------------------------------------- 3927

KMP-5_18 (*r10A*) CAGGGAACTACTCACCGTTCGTGCAACTCATTCCGAAAACGCACAATTGACATA-TACCA 3964

KMP-7_3 (*r12C*) CAGGGAACTACTCACCGTTCGTGCAACTCATTCCGAAAACGCACAATTGACATAATACCA 4037

KMP-7_1 (*r12A*) CAGGGAACTACTCACCGTTCGTGCAACTCATTCCGAAAACGCACAATTGACATAATACCA 4196

KMP-7_4 (*r12D*) CAGGGAACTACTCACCGTTCGTGCAACTCATTCCGAAAACGCACAATTGACATAATACCA 3959

KMP-6_7 (*r12A*) CAGGGAACTACTCACCGTTCGTGCAACTCATTCCGAAAACGCACAATTGACATAATACCA 4196

KMP-7_2 (*r12B*) CAGGGAACTACTCACCGTTCGTGCAACTCATTCCGAAAACGCACAATTGACATAATACCA 4075

KMP-6_3 (*r11A*) CAGGGAACTACTCACCGTTTGTGCAACTCATTCCGAAAACGCACAATTGACATA-TACCA 4172

KMP-6_5 (*r11B*) CAGAGAACTACTCACCGTTCGTGCAACTCATTCCGAAAACGCACAATTGACATA-TACCA 4195

KMP-6_8 (*r11B*) CAGAGAACTACTCACCGTTCGTGCAACTCATTCCGAAAACGCACAATTGACATA-TACCA 4195

KMP-8_3 (*s7*) CAGGGAACTACTCACCGTTCGTGCAACTCATTCCGAAAACGCACAATTGACATA-TACCA 4192

KMP-4_20 (*r8B*) CAGGGAACTACTCACCGTTCGTGCAACTCATTCCGAAAACGCACAATTGACATA-TACCA 4199

KMP-4_16 (*r8A*) CAGGGAACTACTCACCGTTCGTGCAACTCATTCCGAAAACGCACAATTGACATA-TACCA 4029

KMP-4_26 (*r8A*) CAGGGAACCACTCACCGTTCGTGCAACTCATTCCGAAAACGCACAATTGACATA-TACCA 4029

KMP-4_3 (*r8A*) CAGGGAACTACTCACCGTTCGTGCAACTCATTCCGAAAACGCACAATTGACATA-TACCA 4029

KMP-4_8 (*r9A*) CAGGGAATTACTCACCGTTCGTGCAACTCATTCCGAAAACGCACAATTGACATA-TACCA 4195

KMP-4_13 (*r9B*) ------------------------------------------------------------ 3628

AY198374.1 CAGGGAACTACTCACCGTTCGTGCAACTCATTCCGAAAACGCACAATTGACATA-TACCA 4195

KMP-8_35 (*s6B*) CAGGGAACTACTCACCGTTCGTGCAACTCATTCCGAAAACGCACAATTGACATA-TACCA 4192

KMP-8_24 (*s6A*) CAGGGAACTACTCACCGTTCGTGCAACTCATTCCGAAAACGCACAATTGACATA-TACCA 4192

KMP-8_5 (*s6A*) CAGGGAACTACTCACCGTTCGTGCAACTCATTCCGAAAACGCACAATTGACATA-TACCA 4192

KMP-8_46 (*s6A*) CAGGGAACTACTCACCGTTCGTGCAACTCATTCCGAAAACGCACAATTGACATA-TACCA 4192

KMP-5_2 (*r10A*) TCGAAGACGGTTCTATGGCGGTGGACTCCACTCTGGAAGCCGTCAAGGACTCGGCGTTCC 4024

KMP-5_11 (*r10B*) ------------------------------------------------------------ 3927

KMP-5_16 (*r10C*) ------------------------------------------------------------ 3927

KMP-5_18 (*r10A*) TCGAAGACGGTTCTATGGCGGTGGACTCCACTCTGGAAGCCGTCAAGGACTCGGCGTTCC 4024

KMP-7_3 (*r12C*) TCGAAGACGGTTCTATGGCGGTGGACTCCACTCTGGAAGCCGTCAAGGACTCGGCGTTCC 4097

KMP-7_1 (*r12A*) TCGAAGACGGTTCTATGGCGGTGGACTCCACTCTGGAAGCCGTCAAGGACTCGGCGTTCC 4256

KMP-7_4 (*r12D*) TCGAAGACGGTTCTATGGCGGTGGACTCCACTCTGGAAGCCGTCAAGGACTCGGCGTTCC 4019

KMP-6_7 (*r12A*) TCGAAGACGGTTCTATGGCGGTGGACTCCACTCTGGAAGCCGTCAAGGACTCGGCGTTCC 4256

KMP-7_2 (*r12B*) TCGAAGACGGTTCTATGGCGGTGGACTCCACTCTGGAAGCCGTCAAGGACTCGGCGTTCC 4135

KMP-6_3 (*r11A*) TCGAAGATGGTTCTATGGTGGTGGACTCCACTCTGGAAGCCGTCAAGGACTCGGCGTTCC 4232

KMP-6_5 (*r11B*) TCGAAGATGGTTCTATGGTGGTGGACTCCACTCTGGAAGCCGTCAAGGACTCGGCGTGCC 4255

KMP-6_8 (*r11B*) TCGAAGATGGTTCTATGGTGGTGGACTCCACTCTGGAAGCCGTCAAGGACTCGGCGTTCC 4255

KMP-8_3 (*s7*) TCGAAGACGGTTCTATGGCGGTGGACACCACTCTGGAAGCCGTCAAGGACTCGGCGTTCC 4252

KMP-4_20 (*r8B*) TCGAAGACGGTTCTATGGCGGTGGACTCCACTCTGGAAGCCGTCAAGGACTCGGCGTTCC 4259

KMP-4_16 (*r8A*) TCGAAGACGGTTCTATGGCGGTGGACTCCACTCTGGAAGCCGTCAAGGACTCGGCGTTCC 4089

KMP-4_26 (*r8A*) TCGAAGACGGTTCTATGGCGGTGGACTCCACTCTGGAAGCCGTCAAGGACTCGGCGTTCC 4089

KMP-4_3 (*r8A*) TCGAAGACGGTTCTATGGCGGTGGACTCCACTCTGGAAGCCGTCAAGGACTCGGCGTTCC 4089

KMP-4_8 (*r9A*) TCGAAGACGGTTCTATGGCGGTGGACTCCACTCTGGAAGCCGTCAAGGACTCGGCGTTCC 4255

KMP-4_13 (*r9B*) ------------------------------------------------------------ 3628

AY198374.1 TCGAAGACGGTTCTATGGCGGTGGACTCCACTCTGGAAGCCGTCAAGGACTCGGCGTTCC 4255

KMP-8_35 (*s6B*) TCGAAGATGGTTCTATGGTGGTGGACTCCACTCTGGAAGCCGTCAAGGACTCGGCGTTTC 4252

KMP-8_24 (*s6A*) TCGAAGGTGGTTCCATGGTGGTGGACTCCACTCTGGAAGCCGTCAAGGACTCGGCGTTTC 4252

KMP-8_5 (*s6A*) TCGAAGATGGTTCTATGGTGGTGGACTCCACTCTGGAAGCCGTCAAGGACTCGGCGTTTC 4252

KMP-8_46 (*s6A*) TCGAAGATGGTTCTATGGTGGTGGACTCCACTCTGGAAGCCGTCAAGGACTCGGCGTTTC 4252

KMP-5_2 (*r10A*) ATCTGAACGCGCAGACCGGCGTCCTCATACTGAGGATACAACCTACTGCCAGCATGCAGG 4084

KMP-5_11 (*r10B*) ------------------------------------------------------------ 3927

KMP-5_16 (*r10C*) ------------------------------------------------------------ 3927

KMP-5_18 (*r10A*) ATCTGAACGCGCAGACCGGCGTCCTCATACTGAGGATACAACCTACTGCCAGCATGCAGG 4084

KMP-7_3 (*r12C*) ATCTGAACGCGCAGACCGGCGTCCTCATACTGAGGATACAACCTACTGCCAGCATGCAGG 4157

KMP-7_1 (*r12A*) ATCTGAACGCGCAGACCGGCGTCCTCATACTGAGGATACAACCTACTGCCAGCATGCGGG 4316

KMP-7_4 (*r12D*) ATCTGAACGCGCAGACCGGCGTCCTCATACTGAGGATACAACCTACTGCCAGCATGCAGG 4079

KMP-6_7 (*r12A*) ATCTAAACGCGCAGACCGGCGTCCTCATACTGAGGATACAACCTACTGCCAGCATGCAGG 4316

KMP-7_2 (*r12B*) ATCTGAACGCGCAGACCGGCGTCCTCATACTGAGGATACAACCTACTGCCAGCATGCAGG 4195

KMP-6_3 (*r11A*) ATCTGAACGCGCAGACCGGCGTCCTCATACTGAGGATACAACCTACTGCCAGCATGCAGG 4292

KMP-6_5 (*r11B*) ATCTGAACGCGCAGACCGGCGTCCTCATACTGAGGATACAACCTACTGCCAGCATGCAGG 4315

KMP-6_8 (*r11B*) ATCTGAACGCGCAGACCGGCGTCCTCATACTGAGGATACAACCTACTGCCAGCATGCAGG 4315

KMP-8_3 (*s7*) ATCTGAACGCGCAGACCGGCGTCCTCATACTGAGGATACAACCTACTGCCAGCATGCAGG 4312

KMP-4_20 (*r8B*) ATCTGAACGCGCAGACCGGCGTCCTCATACTGAGGATACAACCTACTGCCAGCATGCAGG 4319

KMP-4_16 (*r8A*) ATCTGAACGCGCAGACCGGCGTCCTCATACTGAGGATACAACCTACTGCCAGCATGCAGG 4149

KMP-4_26 (*r8A*) ATCTGAACGCGCAGACCGGCGTCCTCATACTGAGGATACAACCTACTGCCAGCATGCAGG 4149

KMP-4_3 (*r8A*) ATCTGAACGCGCAGACCGGCGTCCTCATACTGAGGATACAACCTACTGCCAGCATGCAGG 4149

KMP-4_8 (*r9A*) ATCTGAACGCGCAGACCGGCGTCCTCATACTGAGGATACAACCTACTGCCAGCATGCAGG 4315

KMP-4_13 (*r9B*) ------------------------------------------------------------ 3628

AY198374.1 ATCTGAACGCGCAGACCGGCGTCCTCATACTGAGGATACAACCTACTGCCAGCATGCAGG 4315

KMP-8_35 (*s6B*) ATCTGAACGCGCAGACCGGCGTCCTCATACTGAGGATACAACCTACTGCCAGCATGCAGG 4312

KMP-8_24 (*s6A*) ATCTGAACGCGCAGACCGGCGTCCTCATACTGAGGATACAACCTACTGCCAGCATGCAGG 4312

KMP-8_5 (*s6A*) ATCTGAACGCGCAGACCGGCGTCCTCATACTGAGGATACAACCTACTGCCAGCATGCAGG 4312

KMP-8_46 (*s6A*) ATCTGAACGCGCAGACCGGCGTCCTCATACTGAGGATACAACCTACTGCCAGCATGCAGG 4312

KMP-5_2 (*r10A*) GCATGTTTGAGTTCAACGTCATCGCTACTGACCCAGATGAGAAGACAGATACGGCAGAGG 4144

KMP-5_11 (*r10B*) ------------------------------------------------------------ 3927

KMP-5_16 (*r10C*) ------------------------------------ATGAGAAGACAGATACGGCAGAGG 3951

KMP-5_18 (*r10A*) GCATGTTTGAGTTCAACGTCATCGCTACTGACCCAGATGAGAAGACAGATACGGCAGAGG 4144

KMP-7_3 (*r12C*) GCATGTTCGGGTTCAACGTCATCGCTACTGATCCAGATGAGAAGACAGACACGGCAGAGG 4217

KMP-7_1 (*r12A*) GCATGTTCGAGTTCAACGTCATCGCTACTGATCCAGATGAGAAGACAGATACGGCAGAGG 4376

KMP-7_4 (*r12D*) GCATGTTCGAGTTCAACGTCATCGCTACTGATCCAGATGAGAAGACAGATACGGCAGAGG 4139

KMP-6_7 (*r12A*) GCATGTTCGAGTTCAACGTCATCGCTACTGATCCGGATGAGAAGACAGATACGGCAGAGG 4376

KMP-7_2 (*r12B*) GCATGTTCGAGTTCAACGTCATCGCTACTGATCCAGATGAGAAGACAGATACGGCAGAGG 4255

KMP-6_3 (*r11A*) GCATGTTTGAGTTCAACGTCATCGCTACTGTCCCAGATGAGAGGACAGATACGGCAGAGG 4352

KMP-6_5 (*r11B*) GCATGTTCGAGTTCAACGTCATCGCTACTGACCCAGATGAGAAGACAGATACGGCAGAGG 4375

KMP-6_8 (*r11B*) GCATGTTTGAGTTCAACGTCATCGCTACTGACCCAGATGAGAAGACAGATACGGCAGAGG 4375

KMP-8_3 (*s7*) GCATGTTCGAGTTCAACGTCATCGCTACTGATCCAGATGAGAAGACAGATACGGCAGGGG 4372

KMP-4_20 (*r8B*) GCATGTTTGAGTTCAACGTCATCGCTACTGACCCAGATGAGAAGACAGATACGGCAGAGG 4379

KMP-4_16 (*r8A*) GCATGTTTGAGTTCAACGTCATCGCTACTGACCCAGATGAGAAGACAGATACGGCAGAGG 4209

KMP-4_26 (*r8A*) GCATGTTTGAGTTCAACGTCATCGCTACTGACCCAGATGAGAAGACAGATACGGCAGAGG 4209

KMP-4_3 (*r8A*) GCATGTTTGAGTTCAACGTCATCGCTACTGACCCAGATGAGAAGACAGATACGGCAGAGG 4209

KMP-4_8 (*r9A*) GCATGTTTGAGTTCAACGTCATCGCTACTGACCCAGATGAGAAGACAGATACGGCAGAGG 4375

KMP-4_13 (*r9B*) ------------------------------------------------------------ 3628

AY198374.1 GCATGTTCGAGTTCAACGTCATCGCTACTGATCCAGATGAGAAGACAGATACGGCAGAGG 4375

KMP-8_35 (*s6B*) GCATGTTCGAGTTCAACGTCATCGCTACTGATCCAGATGAGAAGACAGATACGGCAGAGG 4372

KMP-8_24 (*s6A*) GCATGTTTGAGTTCAACGTCATCGCTACTGACCCAGATGAGAAGACAGATACGGCAGAGG 4372

KMP-8_5 (*s6A*) GCATGTTTGAGTTCAACGTCATCGCTACTGACCCAGATGAGAAGACAGATACGGCAGAGG 4372

KMP-8_46 (*s6A*) GCATGTTTGAGTTCAACGTCATCGCTACTGACCCAGATGAGAAGACAGATACGGCAGAGG 4372

KMP-5_2 (*r10A*) TGAAAGTCTACCTCATCTCATCCCAAAATAGGGTGTCCTTCATATTCCTGAACGATGTGG 4204

KMP-5_11 (*r10B*) ------------------------------------------------------------ 3927

KMP-5_16 (*r10C*) TGAAAGTCTACCTCATTTCATCCCAAAATAGGGTGTCCTTCATATTCCTGAACGATGTGG 4011

KMP-5_18 (*r10A*) TGAAAGTCTACCTCATTTCATCCCAAAATAGGGTGTCCTTCATATTCCTGAACGATGTGG 4204

KMP-7_3 (*r12C*) TGAAAGTCTACCTCATTTCATCCCAAAATAGGGTGTCCTTCATATTCCTGAACGATGTGG 4277

KMP-7_1 (*r12A*) TGAAAGTCTACCTCATTTCATCCCAAAATAGGGTGTCCTTCATATTCCTGAACGATGTGG 4436

KMP-7_4 (*r12D*) TGAAAGTCTACCTCATTTCATCCCAAAATAGGGTGTCCTTCATATTCCTGAACGATGTGG 4199

KMP-6_7 (*r12A*) TGAAAGTCTACCTCATTTCATCCCAAAATAGGGTGTCCTTCATATTCCTGAACGATGTGG 4436

KMP-7_2 (*r12B*) TGAAAGTCTACCCCATTTCATCCCAAAATAGGGTGTCCTTCATATTCCTGAACGATGTGG 4315

KMP-6_3 (*r11A*) TGAAAGTCTACCTCATTTCATCCCAAAATAGGGTGTCCTTCATATTCCTGAACGATGTGG 4412

KMP-6_5 (*r11B*) TGAAAGTCTACCTCATTTCATCCCAAAATAGGGTGTCCTTCATATTCCTGAACGATGTGG 4435

KMP-6_8 (*r11B*) TGAAAGTCTACCTCATTTCATCCCAAAATAGGGTGTCCTTCATATTCCTGAACGATGTGG 4435

KMP-8_3 (*s7*) TGAAAGTCTACCTCATTTCATCCCAAAATAGGGTGTCCTTCATATTCCTGAACGATGTGG 4432

KMP-4_20 (*r8B*) TGAAAGTCTACCTCATTTCATCCCAAAATAGGGTGTCCTTCATATTCCTGAACGATGTGG 4439

KMP-4_16 (*r8A*) TGAAAGTCTACCTCATTTCATCCCAAAATAGGGTGTCCTTCATATTCCTGAACGATGTGG 4269

KMP-4_26 (*r8A*) TGAAAGTCTACCTCATTTCATCCCAAAATAGGGTGTCCTTCATATTCCTGAACGATGTGG 4269

KMP-4_3 (*r8A*) TGAAAGTCTACCTCATTTCATCCCAAAATAGGGTGTCCTTCATATTCCTGAACGATGTGG 4269

KMP-4_8 (*r9A*) TGAAAGTCTACCTCATTTCATCCCAAAATAGGGTGTCCTTCATATTCCTGAACGATGTGG 4435

KMP-4_13 (*r9B*) ------------------------------------------------------------ 3628

AY198374.1 TGAAAGTCTACCTCATTTCATCCCAAAATAGGGTGTCCTTCATATTCCTGAACGATGTGG 4435

KMP-8_35 (*s6B*) TGAGAGTCTACCTCATTTCATCCCAAAATAGGGTGTCCTTCATATTCCTGAACGATGTGG 4432

KMP-8_24 (*s6A*) TGAAAGTCTACCTCATTTCATCCCAAAATAGGGTGTCCTTCATATTCCTGAACGATGTGG 4432

KMP-8_5 (*s6A*) TGAAAGTCTACCTCATTTCATCCCAAAATAGGGTGTCCTTCATATTCCTGAACGATGTGG 4432

KMP-8_46 (*s6A*) TGAAAGTCTACCTCATTTCATCCCAAAATAGGGTGTCCTTCATATTCCTGAACGATGTGG 4432

KMP-5_2 (*r10A*) AGACGGTTGAGAGTAACAGAGACTTTATCGCAGAAACGTTCAGCGTTGGCTTCGGCATGA 4264

KMP-5_11 (*r10B*) --------------------------ATCGCAGAAACGTTCAGCGTTGGCTTCAACATGA 3961

KMP-5_16 (*r10C*) AGACGGTTGAGAGTAACAGAGACTTTATCGCAGAAACGTTCAGCGTTGGCTTCGACATGA 4071

KMP-5_18 (*r10A*) AGACGGTTGAGAGTAACAGAGACTTTATCGCAGAAACGTTCAGCGTTGGCTTCAACATGA 4264

KMP-7_3 (*r12C*) AGACGGTTGAGAGTAACAGAGACTTTATCGCAGAAACGTTCAGCGTTGGCTTCAACATGA 4337

KMP-7_1 (*r12A*) AGACGGTTGAGAGTAACAGAGACTTTATCGCAGAAACGTTCAGCGTTGGCTTCAACATGA 4496

KMP-7_4 (*r12D*) AGACGGTTGAGAGTAACAGAGACTTTATCGCAGAAACGTTCAGCGTTGGCTTCAACATGA 4259

KMP-6_7 (*r12A*) AGACGGTTGAGAGTAACAGAGACTTTATCGCAGAAACGTTCAGCGTTGGCTTCAACATGA 4496

KMP-7_2 (*r12B*) AGACGGTTGAGAGTAACAGAGACTTTATCGCAGAAACGTTCAGCGTTGGCTTCAACATGA 4375

KMP-6_3 (*r11A*) AGACTGTTGAGAGTAACAGAGACTTTATCGCAGAAACGTTCAGCGTTGGCTTCAACATGA 4472

KMP-6_5 (*r11B*) AGACTGTTGAGAGTAACAGAGACTTTATCGCAGAAACGTTCAGCGTTGGCTTCAACATGA 4495

KMP-6_8 (*r11B*) AGACTGTTGAGAGTAACAGAGACTTTATCGCAGAAACGTTCAGCGTTGGCTTCAACATGA 4495

KMP-8_3 (*s7*) AGACGGTTGAGAGTAACAGAGACTTTATCGCAGAAACGTTCAGCGTTGGCTTCAACATGA 4492

KMP-4_20 (*r8B*) AGACGGTTGAGAGTAACAGAGACTTTATCGCAGAAACGTTCAGCGTTGGCTTCAACATGA 4499

KMP-4_16 (*r8A*) AGACGGTTGAGAGTAACAGAGACTTTATCGCAGAAACGTTCAGCGTTGGCTTCAACATGA 4329

KMP-4_26 (*r8A*) AGACGGTTGAGAGTAACAGAGACTTTATCGCAGAAACGTTCAGCGTTGGCTTCAACATAA 4329

KMP-4_3 (*r8A*) AGACGGTTGAGAGTAACAGAGACTTTATCGCAGAAACGTTCAGCGTTGGCTTCAACATAA 4329

KMP-4_8 (*r9A*) AGACGGTTGAGAGTAACAGAGACTTTATCGCAGAAACGTTCAGCGTTGGCTTCAACATGA 4495

KMP-4_13 (*r9B*) ------------------------------------------------------------ 3628

AY198374.1 AGACGGTTGAGAGTAACAGAGACTTTATCGCAGAAACGTTCAGCGTTGGCTTCAACATGA 4495

KMP-8_35 (*s6B*) AGACGGTTGAGAGTAACAGAGACTTTATCGCAGAAACGTTCAGCGTTGGCTTCAACATGA 4492

KMP-8_24 (*s6A*) AGACGGTTGAGAGTAACAGAGACTTTATCGCAGAAACGTTCAGCGTTGGCTTCAACATGA 4492

KMP-8_5 (*s6A*) AGACGGTTGAGAGTAACAGAGACTTTATCGCAGAAACGTTCAGCGTTGGCTTCAACATGA 4492

KMP-8_46 (*s6A*) AGACGGTTGAGAGTAACAGAGACTTTATCGCAGAAACGTTCAGCGTTGGCTTCAACATGA 4492

KMP-5_2 (*r10A*) CCTGCAATATAGATCAGGTGCTGCCGGGCACCAACGACGCCGGGGTGATTCAGGAGGCCA 4324

KMP-5_11 (*r10B*) CCTGCAATATAGATCAGGTGCTGCCGGGCACCAACGACGCCGGGGTGATTCAGGAGGCCA 4021

KMP-5_16 (*r10C*) CCTGCAATATAGATCAGGTGCTGCCGGGCACCAACGACGCCGGGGTGATTCAGGAGGCCA 4131

KMP-5_18 (*r10A*) CCTGCAATATAGATCAGGTGCTGCCGGGCACCAACGACGCCGGGGTGATTCAGGAGGCCA 4324

KMP-7_3 (*r12C*) CCTGCAATATAGATCAGGTGCTGCCGGGCACCAACGACGCCGGGGTGATTCAGGAGGCCA 4397

KMP-7_1 (*r12A*) CCTGCAATATAGATCAGGTGCTGCCGGGCACCAACGACGCCGGGGTGATTCAGGAGGCCA 4556

KMP-7_4 (*r12D*) CCTGCAATATAGATCAGGTGCTGCCGGGCACCAACGACGCCGGGGTGATTCAGGAGGCCA 4319

KMP-6_7 (*r12A*) CCTGCAATATAGATCAGGTGCTGCCGGGCACCAACGACGCCGGGGTGATTCAGGAGGCCA 4556

KMP-7_2 (*r12B*) CCTGCAATATAGATCAGGTGCTGCCGGGCACCAACGACGCCGGGGTGATTCAGGAGGCCA 4435

KMP-6_3 (*r11A*) CCTGCAATATAGATCAGGTGCTGCCGGGCACCAACGACGCCGGGGTGATTCAGGAGGCCA 4532

KMP-6_5 (*r11B*) CCTGCAATATAGATCAGGTGCTGCCGGGCACCAACGACGCCGGGGTGATTCAGGAGGCCA 4555

KMP-6_8 (*r11B*) CCTGCAATATAGATCAGGTGCTGCCGGGCACCAACGACGCCGGGGTGATTCAGGAGGCCA 4555

KMP-8_3 (*s7*) CCTGCAATATAGATCAGGTGCTGCCGGGCACCAACGACGCCGGGGTGATTCAGGAGGCCA 4552

KMP-4_20 (*r8B*) CCTGCAATATAGATCAGGTGCTGCCGGGCACCAACGACGCCGGGGTGATTCAGGAGGCCA 4559

KMP-4_16 (*r8A*) CCTGCAATATAGATCAGGTGCTGCCGGGCACCAACGACGCCGGGGTGATTCAGGAGGCCA 4389

KMP-4_26 (*r8A*) CCTGCAATATAGATCAGGTGCTGCCGGGCACCAACGACGCCGGGGTGATTCAGGAGGCCA 4389

KMP-4_3 (*r8A*) CCTGCAATATAGATCAGGTGCTGCCGGGCACCAACGACGCCGGGGTGATTCAGGAGGCCA 4389

KMP-4_8 (*r9A*) CCTGCAATATAGATCAGGTGCTGCCGGGCACCAACAACGCCGGGGTGATTCAGGAGGCCA 4555

KMP-4_13 (*r9B*) ------------------------------------------------------------ 3628

AY198374.1 CCTGCAATATAGATCAGGTGCTGCCGGGCACCAACGACGCCGGGGTGATTCAGGAGGCCA 4555

KMP-8_35 (*s6B*) CCTGCAATATAGATCAGGTGCTGCCGGGCACCAACGACGCCGGGGTGATTCAGGAGGCCA 4552

KMP-8_24 (*s6A*) CCTGCAATATAGATCAGGTGCTGCCGGGCACCAACGACGCCGGGGTGATTCAGGAGGCCA 4552

KMP-8_5 (*s6A*) CCTGCAATATAGATCAGGTGCTGCCGGGCACCAACGACGCCGGGGTGATTCAGGAGGCCA 4552

KMP-8_46 (*s6A*) CCTGCAATATAGATCAGGTGCTGCCGGGCACCAACGACGCCGGGGTGATTCAGGAGGCCA 4552

KMP-5_2 (*r10A*) TGGCGGAAGTCCACGCTCACTTCATACAGGATAACATCCCTGTGAGCGCCGACAGTATTG 4384

KMP-5_11 (*r10B*) TGGCGGAAGTCCATGCTCACTTCATACAGGATAACATCCCTGTGAGCGCCGACAGTATTG 4081

KMP-5_16 (*r10C*) TGGCGGAAGTCCATGCTCACTTCATACAGGATAACATCCCTGTGAGCGCCGACAGTATTG 4191

KMP-5_18 (*r10A*) TGGCGGAAGTCCATGCTCACTTCATACAGGATAACATCCCTGTGAGCGCCGACAGTATTG 4384

KMP-7_3 (*r12C*) TGGCGGAAGTCCATGCTCACTTCATACAGGATAACATCCCTGTGAGCGCCGACAGTATTG 4457

KMP-7_1 (*r12A*) TGGCGGAAGTCCATGCTCACTTCATACAGGATAACATCCCTGTGAGCGCCGACAGTATTG 4616

KMP-7_4 (*r12D*) TGGCGGAAGTCCATGCTCACTTCATACAGGATAACATCCCTGTGAGCGCCGACAGTATTG 4379

KMP-6_7 (*r12A*) TGGCGGAAGTCCATGCTCACTTCATACAGGATAACATCCCTGTGAGCGCCGACAGTATTG 4616

KMP-7_2 (*r12B*) TGGCGGAAGTCCATGCTCACTTCATACAGGATAACATCCCTGTGAGCGCCGACAGTATTG 4495

KMP-6_3 (*r11A*) TGGCGGAAGTCCATGCTCACTTCATACAGGATAACATCCCTGTGAGCGCCGACAGTATTG 4592

KMP-6_5 (*r11B*) TGGCGGAAGTCCATGCTCACTTCATACAGGATAACATCCCTGTGAGCGCCGACAGTATTG 4615

KMP-6_8 (*r11B*) TGGCGGAAGTCCATGCTCACTTCATACAGGATAACATCCCTGTGAGCGCCGACAGTATTG 4615

KMP-8_3 (*s7*) TGGCGGAAGTCCATGCTCGCTTCATACAGGATAACATCCCTGTGAGCGCCGACAGTATTG 4612

KMP-4_20 (*r8B*) TGGCGGAAGTCCATGCTCACTTCATACAGGATAACATCCCTGTGAGCGCCGACAGTATTG 4619

KMP-4_16 (*r8A*) TGGCGGAAGTCCATGCTCACTTCATACAGGATAACATCCCTGTGAGCGCCGACAGTATTG 4449

KMP-4_26 (*r8A*) TGGCGGAAGTCCATGCTCACTTCATACAGGATAACATCCCTGTGAGCGCCGACAGTATTG 4449

KMP-4_3 (*r8A*) TGGCGGAAGTCCATGCTCACTTCATACAGGATAACATCCCTGTGAGCGCCGACAGTATTG 4449

KMP-4_8 (*r9A*) TGGCGGAAGTCCATGCTCACTTCATACAGGATAACATCCCTGTGAGCGCCGACAGTATTG 4615

KMP-4_13 (*r9B*) ------------------------------------------------------------ 3628

AY198374.1 TGGCGGAAGTCCATGCTCACTTCATACAGGATAACATCCCTGTGAGCGCCGACAGTATTG 4615

KMP-8_35 (*s6B*) TGGCGGAAGTCCATGCTCACTTCATACAGGATAACATCCCTGTGAGCGCCGACAGTATTG 4612

KMP-8_24 (*s6A*) TGGCGGAAGTCCATGCTCACTTCATACAGGATAACATCCCTGTGAGCGCCGACAGTATTG 4612

KMP-8_5 (*s6A*) TGGCGGAAGTCCATGCTCACTTCATACAGGATAACATCCCTGTGAGCGCCGACAGTATTG 4612

KMP-8_46 (*s6A*) TGGCGGAAGTCCATGCTCACTTCATACAGGATAACATCCCTGTGAGCGCCGACAGTATTG 4612

KMP-5_2 (*r10A*) AAGAGCTTCGCAGTGACACTCAGCTGC--------------------------------- 4411

KMP-5_11 (*r10B*) AAGAGCTTCGCAGTGACACTCAGCTGC--------------------------------- 4108

KMP-5_16 (*r10C*) AAGAGCTTCGCAGTGACACTCAGCTGC--------------------------------- 4218

KMP-5_18 (*r10A*) AAGAGCTTCGCAGTGACACTCAGCTGC--------------------------------- 4411

KMP-7_3 (*r12C*) AAGAGCTTCGCAGTGACACTCAGCTGC--------------------------------- 4484

KMP-7_1 (*r12A*) AAGAGCTTCGCAGTGACACTCAGCTGC--------------------------------- 4643

KMP-7_4 (*r12D*) AAGAGCTTCGCAGTGACACTCAGCTGC--------------------------------- 4406

KMP-6_7 (*r12A*) AAGAGCTTCGCAGTGACACTCAGCTGC--------------------------------- 4643

KMP-7_2 (*r12B*) AAGAGCTTCGCAGTGACACTCAGCTGC--------------------------------- 4522

KMP-6_3 (*r11A*) AAGAGCTTCGCAGTGACACTCAGCTGCTGCGGTCACCGGGTGAGAGCCTTCAGCGCTCCC 4652

KMP-6_5 (*r11B*) AAGAGCTTCGCAGTGACACTCAGCTGCTGCGGTCACCGGGTGAGAGCCTTCAGCGCTCCC 4675

KMP-6_8 (*r11B*) AAGAGCTTCGCAGTGACACTCAGCTGCTGCGGTCACCGGGTGAGAGCCTTCAGCGCTCCC 4675

KMP-8_3 (*s7*) AAGAGCTTCGCAGTGACACTCAGCTGC--------------------------------- 4639

KMP-4_20 (*r8B*) AAGAGCTTCGCAGTGACACTCAGCTGC--------------------------------- 4646

KMP-4_16 (*r8A*) AAGAGCTTCGCAGTGACACTCAGCTGC--------------------------------- 4476

KMP-4_26 (*r8A*) AAGAGCTTCGCAGTGACACTCAGCTGC--------------------------------- 4476

KMP-4_3 (*r8A*) AAGAGCTTCGCAGTGACACTCAGCTGC--------------------------------- 4476

KMP-4_8 (*r9A*) AAGAGCTTCGCAGTGACACTCAGCTGC--------------------------------- 4642

KMP-4_13 (*r9B*) ------------------------------------------------------------ 3628

AY198374.1 AAGAGCTTCGCAGTGACACTCAGCTGC--------------------------------- 4642

KMP-8_35 (*s6B*) AAGAGCTTCGCAGTGACACTCAGCTGC--------------------------------- 4639

KMP-8_24 (*s6A*) AAGAGCTTCGCAGTGACACTCAGCTGC--------------------------------- 4639

KMP-8_5 (*s6A*) AAGAGCTTCGCAGTGACACTCAGCTGC--------------------------------- 4639

KMP-8_46 (*s6A*) AAGAACTTCGCAGTGACACTCAGCTGC--------------------------------- 4639

KMP-5_2 (*r10A*) ------------------------------------------------------------ 4411

KMP-5_11 (*r10B*) ------------------------------------------------------------ 4108

KMP-5_16 (*r10C*) ------------------------------------------------------------ 4218

KMP-5_18 (*r10A*) ------------------------------------------------------------ 4411

KMP-7_3 (*r12C*) ------------------------------------------------------------ 4484

KMP-7_1 (*r12A*) ------------------------------------------------------------ 4643

KMP-7_4 (*r12D*) ------------------------------------------------------------ 4406

KMP-6_7 (*r12A*) ------------------------------------------------------------ 4643

KMP-7_2 (*r12B*) ------------------------------------------------------------ 4522

KMP-6_3 (*r11A*) CATTTGTCCGGCCAAGTAGTTAATGCCATTTGCGGCAAATCTACAATAAGTCACGTCAAA 4712

KMP-6_5 (*r11B*) CATTTGTCCGGCCAAGTAGTTAATGCCATTTGCGGCAAATCTACAATAAGTCACGTCAAA 4735

KMP-6_8 (*r11B*) CATTTGTCCGGCCAAGTAGTTAATGCCATTTGCGGCAAATCTACAATAAGTCACGTCAAA 4735

KMP-8_3 (*s7*) ------------------------------------------------------------ 4639

KMP-4_20 (*r8B*) ------------------------------------------------------------ 4646

KMP-4_16 (*r8A*) ------------------------------------------------------------ 4476

KMP-4_26 (*r8A*) ------------------------------------------------------------ 4476

KMP-4_3 (*r8A*) ------------------------------------------------------------ 4476

KMP-4_8 (*r9A*) ------------------------------------------------------------ 4642

KMP-4_13 (*r9B*) ------------------------------------------------------------ 3628

AY198374.1 ------------------------------------------------------------ 4642

KMP-8_35 (*s6B*) ------------------------------------------------------------ 4639

KMP-8_24 (*s6A*) ------------------------------------------------------------ 4639

KMP-8_5 (*s6A*) ------------------------------------------------------------ 4639

KMP-8_46 (*s6A*) ------------------------------------------------------------ 4639

KMP-5_2 (*r10A*) ------------------------------------TGCGCTCCGTCCAAGGTGTGTTGA 4435

KMP-5_11 (*r10B*) ------------------------------------TGCGCTCCGTCCAAGGTGTGTTGA 4132

KMP-5_16 (*r10C*) ------------------------------------TGCGCTCCGTCCAAGGTGTGTTGA 4242

KMP-5_18 (*r10A*) ------------------------------------TGCGCTCCGTCCAAGGTGTGTTGA 4435

KMP-7_3 (*r12C*) ------------------------------------TGCGCTCCGTCCAAGGTGTGTTGA 4508

KMP-7_1 (*r12A*) ------------------------------------TGCGCTCCGTCCAAGGTGTGTTGA 4667

KMP-7_4 (*r12D*) ------------------------------------TGCGCTCCGTCCAAGGTGTGTTGA 4430

KMP-6_7 (*r12A*) ------------------------------------TGCGCTCCGTCCAAGGTGTGTCGA 4667

KMP-7_2 (*r12B*) ------------------------------------TGCGCTCCGTCCAAGGTGTGTTGA 4546

KMP-6_3 (*r11A*) AAAAAAAAAAAAAAA--AAAAAAAAAAAAAAGCTGCTGCGCTCCGTCCAAGGTGTGTTGA 4770

KMP-6_5 (*r11B*) AAAAAAAAAAAAA----AAAAAAAAAAAAAAGCTGCTGCGCTCCGTCCAAGGTGTGTTGA 4791

KMP-6_8 (*r11B*) AAAAAAAAAAAAAAAAAAAAAAAAAAAAAAAGCCGCTGCGCTCCGTCCAAGGTGTGTTGA 4795

KMP-8_3 (*s7*) ------------------------------------TGCGCTCCGTCCAAGGTGTGTTGA 4663

KMP-4_20 (*r8B*) ------------------------------------TGCGCTCCGTCCAAGGTGTGTTGA 4670

KMP-4_16 (*r8A*) ------------------------------------TGCGCTCCGTCCAAGGTGTGTTGA 4500

KMP-4_26 (*r8A*) ------------------------------------TGCGCTCCGTCCAAGGTGTGTTGA 4500

KMP-4_3 (*r8A*) ------------------------------------TGCGCTCCGTCCAAGGTGTGTTGA 4500

KMP-4_8 (*r9A*) ------------------------------------TGCGCTCCGTCCAAGGTGTGTTGA 4666

KMP-4_13 (*r9B*) ------------------------------------------------------------ 3628

AY198374.1 ------------------------------------TGCGCTCCGTCCAAGGTGTGTTGA 4666

KMP-8_35 (*s6B*) ------------------------------------TGCGCTCCGTCCAAGGTGTGTTGA 4663

KMP-8_24 (*s6A*) ------------------------------------TGCGCTCCGTCCAAGGTGTGTTGA 4663

KMP-8_5 (*s6A*) ------------------------------------TGCGCTCCGTCCAAGGTGTGTTGA 4663

KMP-8_46 (*s6A*) ------------------------------------TGCGCTCCGTCCAAGGTGTGCTGA 4663

KMP-5_2 (*r10A*) ACCAACGGCTGTTGGTCCTGAACGACCTGGTGACGGGGGTCAGCCCTGATCTCGGCACTG 4495

KMP-5_11 (*r10B*) ACCAACGGCTGTTGGTCCTGAACGACCTGGTGACGGGGGTCAGCCCTGATCTCGGCACTG 4192

KMP-5_16 (*r10C*) ACCAACGGCTGTTGGTCCTGAACGACCTGGTGACGGGGGTCAGCCCTGATCTCGGCACTG 4302

KMP-5_18 (*r10A*) ACCAACGGCTGTTGGTCCTGAACGACCTGGTGACGGGGGTCAGCCCTGATCTCGGCACTG 4495

KMP-7_3 (*r12C*) ACCAACGGCTGTTGGTCCTGAACGACCTGGTGACGGGGGTCAGCCCTGATCTCGGCACTG 4568

KMP-7_1 (*r12A*) ACCAACGGCTGTTGGTCCTGAACGACCTGGTGACGGGGGTCAGCCCTGATCTCGGCACTG 4727

KMP-7_4 (*r12D*) ACCAACGGCTGTTGGTCCTGAACGACCTGGTGACGGGGGTCAGCCCTGATCTCGGCACTG 4490

KMP-6_7 (*r12A*) ACCAACGGCTGTTGGTCCTGAACGACCTGGTGACGGGGGTCAGCCCTGATCTCGGCACTG 4727

KMP-7_2 (*r12B*) ACCAACGGCTGTTGGTCCTGAACGACCTGGTGACGGGGGTCAGCCCTGATCTCGGCACTG 4606

KMP-6_3 (*r11A*) ACCAACGGCTGTTGGTCCTGAACGACCTGGTGACCGGGGTCAGCCCTGATCTCGGCACTG 4830

KMP-6_5 (*r11B*) ACCAACGGCTGTTGGTCCTGAACGACCTGGTGACCGGGGTCAGCCCTGATCTCGGCACTG 4851

KMP-6_8 (*r11B*) ACCAACGGCTGTTGGTCCTGAACGACCTGGTGACCGGGGTCAGCCCTGATCTCGGCACCG 4855

KMP-8_3 (*s7*) ACCAACGGCTGTTGGTCCTGGACGACCTGGTGACGGGGGTCAGCCCTGATCTCGGCACTG 4723

KMP-4_20 (*r8B*) ACCAACGGCTGTTGGTCCTGAACGACCTGGTGACGGGGGTCAGCCCTGATCTCGGCACTG 4730

KMP-4_16 (*r8A*) ACCAACGGCTGTTGGTCCTGAACGACCTGGTGACGGGGGTCAGCCCTGATCTCGGCACTG 4560

KMP-4_26 (*r8A*) ACCAACGGCTGTTGGTCCTGAACGACCTGGTGACGGGGGTCAGCCCTGATCTCGGCACTG 4560

KMP-4_3 (*r8A*) ACCAACGGCTGTTGGTCCTGAACGACCTGGTGACGGGGGTCAGCCCTGATCTCGGCACTG 4560

KMP-4_8 (*r9A*) ACCAACGGCTGTTGGTCCTGAACGACCTGGTGACGGGGGTCAGCCCTGATCTCGGCACTG 4726

KMP-4_13 (*r9B*) ------------------------------------------------------------ 3628

AY198374.1 ACCAACGGCTGTTGGTCCTGAACGACCTGGTGACGGGGGTCAGCCCTGATCTCGGCACTG 4726

KMP-8_35 (*s6B*) ACCAACGGCTGTTGGTCCTGAACGACCTGGTGACGGGGGTCAGCCCTGATCTCGGCACTG 4723

KMP-8_24 (*s6A*) ACCAACGGCTGTTGGTCCTGAACGACCTGGTGACCGGGGTCAGCCCTGATCTCGGCACTG 4723

KMP-8_5 (*s6A*) ACCAACGGCTGTTGGTCCTGAACGACCTGGTGACCGGGGTCAGCCCTGATCTCGGCACTG 4723

KMP-8_46 (*s6A*) ACCAACGGCTGTTGGTCCTGAACGACCTGGTGACCGGGGTCAGCCCTGATCTCGGCACTG 4723

KMP-5_2 (*r10A*) CCGGCGTGCAGATCACCATCTATGTGCTAGCCGGGTTGTCAGCCATCCTTGCCTTCCTGT 4555

KMP-5_11 (*r10B*) CCGGCGTGCGGATCACCATCTATGTGCTAGCCGGGTTGTCAGCCATCCTTGCCTTCCTGT 4252

KMP-5_16 (*r10C*) CCGGCGTGCAGATCACCATCTATGTGCTAGCCGGGTTGTCAGCCATCCTTGCCTTCCTGT 4362

KMP-5_18 (*r10A*) CCGGCGTGCAGATCACCATCTATGTGCTAGCCGGGTTGTCAGCCATCCTTGCCTTCCTGT 4555

KMP-7_3 (*r12C*) CCGGCGTGCAGATCACCATCTATGTGCTAGCCGGGTTGTCAGCCATCCTTGCCTTCCTGT 4628

KMP-7_1 (*r12A*) CCGGCATGCAGATCACCATCTATGTGCTAGCCGGGTTGTCAGCCATCCTTGCCTTCCTGT 4787

KMP-7_4 (*r12D*) CCGGCGTGCAGATCACCATCTATGTGCTAGCCGGGTTGTCAGCCATCCTTGCCTTCCTGT 4550

KMP-6_7 (*r12A*) CCGGCGTGCAGATCACCATCTATGTGCTAGCCGGGTTGTCAGCCATCCTCGCCTTCCTGT 4787

KMP-7_2 (*r12B*) CCGGCGTGCAGATCACCATCTATGTGCTAGCCGGGTTGTCAGCCATCCTTGCCTTCCTGT 4666

KMP-6_3 (*r11A*) CCGGCGTGCAGATCACCATCTATGTGCTAGCCGGGTTGTCAGCCATCCTTGCCTTCCTGT 4890

KMP-6_5 (*r11B*) CCGGCGTGCAGATCACCATCTATGTGCTAGCCGGGTTGTCAGCCATCCTTGCCTTCCTGT 4911

KMP-6_8 (*r11B*) CCGGCGTGCAGATCACCATCTATGTGCTAGCCGGGTTGTCAGCCATCCTTGCCTTCCTGT 4915

KMP-8_3 (*s7*) CCGGCGTGCAGATCACCATCTATGTGCTAGCCGGGTTGTCAGCCATCCTTGCCTTCCTGT 4783

KMP-4_20 (*r8B*) CCGGCGTGCAGATCACCATCTATGTGCTAGCCGGGTTGTCAGCCATCCTTGCCTTCCTGT 4790

KMP-4_16 (*r8A*) CCGGCGTGCAGATCACCATCTATGTGCTAGCCGGGTTGTCAGCCATCCTTGCCTTCCTGT 4620

KMP-4_26 (*r8A*) CCGGCGTGCAGATCACCATCTATGTGCTAGCCGGGTTGTCAGCCATCCTTGCCTTCCTGT 4620

KMP-4_3 (*r8A*) CCGGCGTGCAGATCACCATCTATGTGCTAGCCGGGTTGTCAGCCATCCTTGCCTTCCTGT 4620

KMP-4_8 (*r9A*) CCGGCGTGCAGATCACCATCTATGTGCTAGCCGGGTTGTCAGCCATCCTTGCCTTCCTGT 4786

KMP-4_13 (*r9B*) -----------------------------------------------------------T 3629

AY198374.1 CCGGCGTGCAGATCACCATCTATGTGCTAGCCGGGTTGTCAGCCATCCTTGCCTTCCTGT 4786

KMP-8_35 (*s6B*) CCGGCGTGCAGATCACCATCTATGTGCTAGCCGGGTTGTCAGCTATCCTTGCCTTCCTGT 4783

KMP-8_24 (*s6A*) CCGGCGTGCAGATCACCATCTATGTGCTAGCCGGGTTGTCAGCCATCCTTGCCTTCCTGT 4783

KMP-8_5 (*s6A*) CCGGCGTGCAGATCACCATCTATGTGCTAGCCGGGTTGTCAGCCATCCTTGCCTTCCTGT 4783

KMP-8_46 (*s6A*) CCGGCGTGCAGATCACCATCTATGTGCTAGCCGGGTTGTCAGCCATCCTTGCCTTCCTGT 4783

*

KMP-5_2 (*r10A*) GCCTTATTCTGCTCATCACATTCATCGTGAGGACCCGAGCTCTGAACCGCCGTTTGGAAG 4615

KMP-5_11 (*r10B*) GCCTTATTCTGCTCATCACATTCATCGTGAGGACCCGAGCTCTGAGCCGCCGTTTGGAAG 4312

KMP-5_16 (*r10C*) GCCTTATTCTGCTCATCACATTCATCGTGAGGACCCGAGCTCTGAACCGCCGTTTGGAAG 4422

KMP-5_18 (*r10A*) GCCTTATTCTGCTCATCACATTCATCGTGAGGACCCGAGCTCTGAACCGCCGTTTGGAAG 4615

KMP-7_3 (*r12C*) GCCTTACTCTGCTCATCACATTCATCGTGAGGACCCGAGCTCTGAACCGCCGTTTGGAAG 4688

KMP-7_1 (*r12A*) GCCTTATTCTGCTCATCACATTCATCGTGAGGACCCGAGCTCTGAACCGCCGTTTGGAAG 4847

KMP-7_4 (*r12D*) GCCTTATTCTGCTCATCACATTCATCGTGAGGACCCGAGCTCTGAACCGCCGTTTGGAAG 4610

KMP-6_7 (*r12A*) GCCTTATTCTGCTCATCACATTCATCGTGAGGACCCGAGCTCTGAACCGCCGTTTGGAAG 4847

KMP-7_2 (*r12B*) GCCTTATTCTGCTCATCACATTCATCGTGAGGACCCGAGCTCTGAACCGCCGTTTGGAAG 4726

KMP-6_3 (*r11A*) GCCTTATTCTGCTCATCACATTCATCGTGAGGACCCGAGCTCTGAACCGCCGTTTGGAAG 4950

KMP-6_5 (*r11B*) GCCTTATTCTGCTCATCACATTCATCGTGAGGACCCGAGCTCTGAACCGCCGTTTGGAAG 4971

KMP-6_8 (*r11B*) GCCTTATTCTGCTCATCACATTCATCGTGAGGACCCGAGCTCTGAACCGCCGTTTGGAAG 4975

KMP-8_3 (*s7*) GCCTTATTCTGCTCATCACATTCATCGTGAGGTCCCGAGCTCTGAACCGCCGTTTGGAAG 4843

KMP-4_20 (*r8B*) GCCTTATTCTGCTCATCACATTCATCGTGAGGACCCGAGCTCTGAACCGCCGTTTGGAAG 4850

KMP-4_16 (*r8A*) GCCTTATTCTGCCCATCACATTCATCGTGAGGACCCGAGCTCTGAACCGCCGTTTGGAAG 4680

KMP-4_26 (*r8A*) GCCTTATTCTGCTCATCACATTCATCGTGAGGACCCGAGCTCTGAACCGCCGTTTGGAAG 4680

KMP-4_3 (*r8A*) GCCTTATTCTGCTCATCACATTCATCGTGAGGACCCGAGCTCTGAACCGCCGTTTGGAAG 4680

KMP-4_8 (*r9A*) GCCTTATTCTGCTCATCACATTCATCGTGAGGACCCGAGCT------------------- 4827

KMP-4_13 (*r9B*) GCCTTATTTTGCTCATCACATTCATCGTGAGGACCCGAGCTCTGAACCGCCGTTTGGAAG 3688

AY198374.1 GCCTTATTCTGCTCATCACATTCATCGTGAGGACCCGAGCTCTGAACCGCCGTTTGGAAG 4846

KMP-8_35 (*s6B*) GCCTTATTCTGCTCATCACATTCATCGTGAGGACCCGAGCTCTGAACCGCCGTTTGGAAG 4843

KMP-8_24 (*s6A*) GCCTTATTCTGCTCATCACATTCATCGTGAGGACCCGAGCTCTGAACCGCCGTTTGGAAG 4843

KMP-8_5 (*s6A*) GCCTTATTCTGCTCATCACATTCATCGTGAGGACCCGAGCTCTGAACCGCCATTTGGAAG 4843

KMP-8_46 (*s6A*) GCCTTATTCTGCTCATCACATTCATCGTGAGGACCCGAGCTCTGAACCGCCGTTTGGAAG 4843

****** * *** *******************:********

KMP-5_2 (*r10A*) CACTGTCGATGACGAAATACGGCTCGGTGGATTCGGGGCTGAACCGAGTGGGGATAGCGG 4675

KMP-5_11 (*r10B*) CACTGTCGATGACGAAATACGGCTCGGCGGATTCGGGGCTGAACCGAGTGGGGATAGCGG 4372

KMP-5_16 (*r10C*) CACTGTCGATGACGAAATACGGCTCGGTGGATTCGGGGCTGAACCGAGTGGGGATAGCGG 4482

KMP-5_18 (*r10A*) CACTGTCGATGACGAAATACGGCTCGGTGGATTCGGGGCTGAACCGAGTGGGGATAGCGG 4675

KMP-7_3 (*r12C*) CACTGTCGATGACGAAATATGGCTCGGTGGATTCGGGGCTGAACCGAGTGGGGATAGCGG 4748

KMP-7_1 (*r12A*) CACTGTCGATGACGAAATACGGCTCGGTGGATTCGGGGCTGAACCGAGTGGGGATAGCGG 4907

KMP-7_4 (*r12D*) CACTGTCGATGACGAAGTACGGCTCGGTGGATTCGGGGCTGAACCGAGTGGGGATAGCGG 4670

KMP-6_7 (*r12A*) CACTGCCGATGACGAAATACGGCTCGGTGGATTCGGGGCTGAACCGAGTGGGGATAGCGG 4907

KMP-7_2 (*r12B*) CACTGTCGATGACGAAATACGGCTCGGTGGATTCGGGGCTGAACCGAGTGGGGATAGCGG 4786

KMP-6_3 (*r11A*) CACCGTCGATGACGAAATACGGCTCGGTGGATTCGGGGCTGAACCGAGTGGGGATAGCGG 5010

KMP-6_5 (*r11B*) CACTGTCGATGACGAAATACGGCTCGGTGGATTCGGGGCTGAACCGAGTGGGGATAGCGG 5031

KMP-6_8 (*r11B*) CACTGTCGTTGACGAAATACGGCTCGGTGGATTCGGGGCTGAACCGAGTGGGGATAGCGG 5035

KMP-8_3 (*s7*) CACTGTCGATGACGAAATACGGCTCGGTGGATTCGGGGCTGAACCGAGTGGGGATAGCGG 4903

KMP-4_20 (*r8B*) CACTGTCGATGACGAAATACGGCTCGGTGGATTCGGGGCTGAACCGAGTGGGGATAGCGG 4910

KMP-4_16 (*r8A*) CACTGTCGATGACGAAATACGGCTCGGTGGATTCGGGGCTGAACCGAGTGGGGATAGCGG 4740

KMP-4_26 (*r8A*) CACTGTCGATGACGAAATACGGCTCGGTGGATTCGGGGCTGAACCGAGTGGGGATAGCGG 4740

KMP-4_3 (*r8A*) CACTGTCGATGACGAAATACGGCTCGGTGGATTCGGGGCTGAACCGAGTGGGGATAGCGG 4740

KMP-4_8 (*r9A*) ------------------------------------------------------------ 4827

KMP-4_13 (*r9B*) CACTGTCGATGACGAAATACGGCTCGGTGGATTCGG-GCTGAACCGAGTGGGGATAGCGG 3748

AY198374.1 CACTGTCGATGACGAAATACGGCTCGGTGGATTCGGGGCTGAACCGAGTGGGGATAGCGG 4906

KMP-8_35 (*s6B*) CACTGTCGATGACGAAATACGGCTCGGTGGATTCGGGGCTGAACCGAGTGGGGATAGCGG 4903

KMP-8_24 (*s6A*) CACTGTCGATGACGAAATACGGCTCGGTGGATTCGGGGCTGAACCGAGTGGGGATAGCGG 4903

KMP-8_5 (*s6A*) CACTGTCGATGACGAAATACGGCTCGGTGGATTCGGGGCTGAACCGAGTGGGGATAGCGG 4903

KMP-8_46 (*s6A*) CACTGTCGATGACGAAATACGGCTCGGTGGATTCGGGGCTGAACCGAGTGGGGATAGCGG 4903

KMP-5_2 (*r10A*) CCCCAGGAACCAACAAACACGCCATCGAAGGCTCCAACCCCATCTGGAACGAGCAGATCA 4735

KMP-5_11 (*r10B*) CCCCAGGAACCAACAAACACGCCATCGAAGGCTCCAACCCCATCTGGAACGAGCAGATCA 4432

KMP-5_16 (*r10C*) CCCCAGGAACCAACAAACACGCCATCGAAGGCTCCAACCCCATCTGGAACGAGCAGATCA 4542

KMP-5_18 (*r10A*) CCCCAGGAACCAACAAACACGCCATCGAAGGCTCCAACCCCATCTGGAACGAGCAGATCA 4735

KMP-7_3 (*r12C*) CCCCAGGAACCAACAAACACGCCATCGAAGGCTCCAACCCCATCTGGAACGAGCAGATCA 4808

KMP-7_1 (*r12A*) CCCCAGGAACCAACAAACACGCCATCGAAGGCTCCAACCCCATCTGGAACGAGCAGATCA 4967

KMP-7_4 (*r12D*) CCCCAGGAACCAACAAACACGCCATCGAAGGCTCCAACCCCATCTGGAACGAGCAGATCA 4730

KMP-6_7 (*r12A*) CCCCAGGAACCAACAAACACGCCATCGAAGGCTCCAACCCCATCTGGAACGAGCAGATCA 4967

KMP-7_2 (*r12B*) CCCCAGGAACCAACAAACACGCCATCGAAGGCTCCAACCCCATCTGGAACGAGCAGATCA 4846

KMP-6_3 (*r11A*) CCCCAGGAACCAACAAACACGCCATCGAAGGCTCCAACCCCATCTGGAACGAGCAGATCA 5070

KMP-6_5 (*r11B*) CCCCGGGAACCAACAAACACGCCATCGAAGGCTCCAACCCCATCTGGAACGAGCAGATCA 5091

KMP-6_8 (*r11B*) CCCCAGGAACCAACAAACACGCCATCGAAGGCTCCAACCCCATCTGGAACGAGCAGATCA 5095

KMP-8_3 (*s7*) CCCCAGGAACCAACAAACACGCCATCGAAGGCTCCAACCCCATCTGGAACGAGCAGATCA 4963

KMP-4_20 (*r8B*) CCCCAGGAACCAACAAACACGCCATCGAAGGCTCCAACCCCATCTGGAACGAGCAGATCA 4970

KMP-4_16 (*r8A*) CCCCAGGAACCAACAAACACGCCATCGAAGGCTCCAACCCCATCTGGAACGAGCAGATCA 4800

KMP-4_26 (*r8A*) CCCCAGGAACCAACAAACACGCCATCGAAGGCTCCAACCCCATCTGGAACGAGCAGATCA 4800

KMP-4_3 (*r8A*) CCCCAGGAACCAACAAACACGCCATCGAAGGCTCCAACCCCATCTGGAACGAGCAGATCA 4800

KMP-4_8 (*r9A*) ------------------------------------------------------------ 4827

KMP-4_13 (*r9B*) CCCCAGGAACCAACAAACACGCCATCGAAGGCTCCAACCCCATCTGGAACGAGCAGATCA 3808

AY198374.1 CCCCAGGAACCAACAAACACGCCATCGAAGGCTCCAACCCCATCTGGAACGAGCAGATCA 4966

KMP-8_35 (*s6B*) CCCCAGGAACCAACAAACACGCCATCGAAGGCTCCAACCCCATCTGGAACGAGCAGATCA 4963

KMP-8_24 (*s6A*) CCCCAGGAACCAACAAACACGCCATCGAAGGCTCCAACCCCATCTGGAACGAGCAGATCA 4963

KMP-8_5 (*s6A*) CCCCAGGAACCAACAAACACGCCATCGAAGGCTCCAACCCCATCTGGAACGAGCAGATCA 4963

KMP-8_46 (*s6A*) CCCCAGGAACCAACAAACACGCCATCGAAGGCTCCAACCCCATCTGGAACGAGCAGATCA 4963

KMP-5_2 (*r10A*) AGGCCCCGGACTTCGATGCCATCAGTGACACATCTGACGAGTCTGATCTGATCGGCATCG 4795

KMP-5_11 (*r10B*) AGGCCCCGGACTTCGATGCCATCAGTGACACATCTGACGAGTCTGATCTGATCGGCATCG 4492

KMP-5_16 (*r10C*) AGGCCCCGGACTTCGATGCCATCAGTGACACATCTGACGAGTCTGATCTGATCGGCATCG 4602

KMP-5_18 (*r10A*) AGGCCCCGGACTTCGATGCCATCAGTGACACATCTGACGAGTCTGATCTGATCGGCATCG 4795

KMP-7_3 (*r12C*) AGGCCCCGGACTTCGATGCCATCAGTGACACATCTGACGAGTCTGATCTGATCGGCATCG 4868

KMP-7_1 (*r12A*) AGGCCCCGGACTTCGATGCCATCAGTGACACATCTGACGAGTCTGATCTGATCGGCATCG 5027

KMP-7_4 (*r12D*) AGGCCCCGGACTTCGATGCCATCAGTGACACATCTGACGAGTCTGATCTGATCGGCATCG 4790

KMP-6_7 (*r12A*) AGGCCCCGGACTTCGATGCCATCAGTGACACATCTGACGAGTCTGATCTGATCGGCATCG 5027

KMP-7_2 (*r12B*) AGGCCCCGGACTTCGATGCCATCAGTGACACATCTGACGAGTCTGATCTGATCGGCATCG 4906

KMP-6_3 (*r11A*) AGGCCCCGGACTTCGATGCCATCAGTGGCACATCTGACGAGTCTGATCTGATCGGCATCG 5130

KMP-6_5 (*r11B*) AGGCCCCGGACTTCGATGCCATCAGTGACACATCTGACGAGTCTGATCTGATCGGCATCG 5151

KMP-6_8 (*r11B*) AGGCCCCGGACTTCGATGCCATCAGTGACACATCTGACGAGTCTGATCTGATCGGCATCG 5155

KMP-8_3 (*s7*) AGGCCCCGGACTTCGATGCCATCAGTGACACATCTGACGAGTCTGATCTGATCGGCATCG 5023

KMP-4_20 (*r8B*) AGGCCCCGGACTTCGATGCCATCAGTGACACATCTGACGAGTCTGATCTGATCGGCATCG 5030

KMP-4_16 (*r8A*) AGGCCCCGGACTTCGATGCCATCAGTGACACATCTGACGAGTCTGATCTGATCGGCATCG 4860

KMP-4_26 (*r8A*) AGGCCCCGGACTTCGATGCCATCAGTGACACATCTGACGAGTCTGATCTGATCGGCATCG 4860

KMP-4_3 (*r8A*) AGGCCCCGGACTTCGATGCCATCAGTGACACATCTGACGAGTCTGATCTGATCGGCATCG 4860

KMP-4_8 (*r9A*) --------------------------GACACATCTGACGAGTCTGATCTGATCGGCATCG 4861

KMP-4_13 (*r9B*) CAGCCCCGGACTTCGATGCCATCAGTGACACATCTGACGAGTCTGATCTGATCGGCATCG 3868

AY198374.1 AGGCCCCGGACTTCGATGCCATCAGTGACACATCTGACGAGTCTGATCTGATCGGCATCG 5026

KMP-8_35 (*s6B*) AGGCCCCGGACTTCGATGCCATCAGTGACACATCTGACGAGTCTGATCTGATCGGCATCG 5023

KMP-8_24 (*s6A*) AGGCCCCGGACTTCGATGCCATCAGTGACACATCTGACGAGTCTGATCTGATCGGCATCG 5023

KMP-8_5 (*s6A*) AGGCCCCGGACTTCGATGCCATCAGTGACACATCTGACGAGTCTGATCTGATCGGCATCG 5023

KMP-8_46 (*s6A*) AGGCCCCGGACTTCGATGCCATCAGTGACACATCTGGCGAGTCTGATCTGATCGGCATCG 5023

**********.***********************

KMP-5_2 (*r10A*) AGGATCTACCACAATTCAAGAGCGACTATTTCCCGCCTGAGGACTCGGAATCCGCTCACG 4855

KMP-5_11 (*r10B*) AGGATCTACCACAATTCAAGAGCGACTATTTCCCGCCTGAGGACTCGGAATCCGCTCACG 4552

KMP-5_16 (*r10C*) AGGATCTACCACAATTCAAGAGCGACTATTTCCCGCCTGAGGACTCGGAATCCGCTCACG 4662

KMP-5_18 (*r10A*) AGGATCTACCACAATTCAAGAGCGACTATTTCCCGCCTGAGGACTCGGAATCCGCTCACG 4855

KMP-7_3 (*r12C*) AGGATCTACCACAATTCAAGAGCGACTATTTCCCGCCTGAGGACTCGGAATCCGCTCACG 4928

KMP-7_1 (*r12A*) AGGATCTACCACAATTCAAGAGCGACTATTTCCCGCCTGAGGACTCGGAATCCGCTCACG 5087

KMP-7_4 (*r12D*) AGGATCTACCACAATTCAAGAGCGACTATTTCCCGCCTGAGGACTCGGAATCCGCTCACG 4850

KMP-6_7 (*r12A*) AGGATCTACCACAATTCAAGAGCGACTATTTCCCGCCTGAGGACTCGGAATCCGCTCACG 5087

KMP-7_2 (*r12B*) AGGATCTACCACAATTCAAGAGCGACTATTTCCCGCCTGAGGACTCGGAATCCGCTCACG 4966

KMP-6_3 (*r11A*) AGGATCTACCACAATTCAAGAGCGACTATTTCCCGCCTGAGGACTCGGAATCCGCTCACG 5190

KMP-6_5 (*r11B*) AGGATCTACCACAATTCAAGAGCGACTATTTCCCGCCTGAGGACTCGGAATCCGCTCACG 5211

KMP-6_8 (*r11B*) AGGATCTACCACAATTCAAGAGCGACTATTTCCCGCCTGAGGACTCGGAATCCGCTCACG 5215

KMP-8_3 (*s7*) AGGATCTACCACAATTCAAGAGCGACTATTTCCCGCCTGAGGACTCGGAATCCGCTCACG 5083

KMP-4_20 (*r8B*) AGGATCTACCACAATTCAAGAGCGACTATTTCCCGCCTGAGGACTCGGAATCCGCTCACG 5090

KMP-4_16 (*r8A*) AGGATCTACCACAATTCAAGAGCGACTATTTCCCGCCTGAGGACTCGGAATCCGCTCACG 4920

KMP-4_26 (*r8A*) AGGATCTACCACAATTCAAGAGCGACTATTTCCCGCCTGAGGACTCGGAATCCGCTCACG 4920

KMP-4_3 (*r8A*) AGGATCTACCACAATTCAAGAGCGACCATTTCCCGCCTGAGGACTCGGAATCCGCTCACG 4920

KMP-4_8 (*r9A*) AGGATCTACCACAATTCAAGAGCGACCATTTCCCGCCTGAGGACTCGGAATCCGCTCACG 4921

KMP-4_13 (*r9B*) AGGATCTACCACAATTCAAGAGCGACTATTTCCCGCCTGAGGACTCGGAATCCGCTCACG 3928

AY198374.1 AGGATCTACCACAATTCAAGAGCGACTATTTCCCGCCTGAGGACTCGGAATCCGCTCACG 5086

KMP-8_35 (*s6B*) AGGATCTACCACAATTCAAGAGCGACTATTTCCCGCCTGAGGACTCGGAATCCGCTCACG 5083

KMP-8_24 (*s6A*) AGGATCTACCACAATTCAAGAGCGACTATTTCCCGCCTGAGGACTCGGAATCCGCTCACG 5083

KMP-8_5 (*s6A*) AGGATCTACCACAATTCAAGAGCGACTATTTCCCGCCTGAGGACTCGGAATCCGCTCACG 5083

KMP-8_46 (*s6A*) AGGATCTACCACAATTCAAGAGCGACTATTTCCCGCCTGAGGACTCGGAATCCGCTCACG 5083

************************** *********************************

KMP-5_2 (*r10A*) CCGCCTTTAGCGACCGCACGCCACGCGGGAACGATGCGCCTATTGCACACAGTAGCAACA 4915

KMP-5_11 (*r10B*) CCGCCTTTAGCGACCGCACGCCACGCGGGAACGATGCGCCTATTGCACACAGTAGCAACA 4612

KMP-5_16 (*r10C*) CCGCCTTTAGCGACCGCACGCCACGCGGGAACGATGCGCCTATTGCACACAGTAGCAACA 4722

KMP-5_18 (*r10A*) CCGCCTTTAGCGACCGCACGCCACGCGGGAACGATGCGCCTATTGCACACAGTAGCAACA 4915

KMP-7_3 (*r12C*) CCGCCTTTAGCGACCGCACGCCACGCGGGAACGATGCGCCTATTGCACACAGTAGCAACA 4988

KMP-7_1 (*r12A*) CCGCCTTTAGCGACCGCACGCCACGCGGGAACGATGCGCCTATTGCACACAGTAGCAACA 5147

KMP-7_4 (*r12D*) CCGCCTTTAGCGACCGCACGCCACGCGGGAACGATGCGCCTATTGCACACAGTAGCAACA 4910

KMP-6_7 (*r12A*) CCGCCTTTGGCGACCGCACGCCACGCGGGAACGATGCGCCTATTGCACACAGTAGCAACA 5147

KMP-7_2 (*r12B*) CCGCCTTTAGCGACCGCACGCCACGCGGGAACGATGCGCCTATTGCACACAGTAGCAACA 5026

KMP-6_3 (*r11A*) CCGCCTTTAGCGACCGCACGCCACGCGGGAACGATGCGCCTATTGCACACAGTAGCAACA 5250

KMP-6_5 (*r11B*) CCGCCTTTAGCGACCGCACGCCACGCGGGAACGATGCGCCTATTGCACACAGTAGCAACA 5271

KMP-6_8 (*r11B*) CCGCCTTTAGCGACCGCACGCCACGCGGGAACGATGCGCCTATTGCACACAGTAGCAACA 5275

KMP-8_3 (*s7*) CCGCCTTTAGCGACCGCACGCCACGCGGGAACGATGCGCCTATTGCACACAGTAGCAACA 5143

KMP-4_20 (*r8B*) CCGCCTTTAGCGACCGCACGCCACGCGGGAACGATGCGCCTATTGCACACAGTAGCAACA 5150

KMP-4_16 (*r8A*) CCGCCTTTAGCGACCGCACGCCACGCGGGAACGATGCGCCTATTGCACACAGTAGCAACA 4980

KMP-4_26 (*r8A*) CCGCCTTTAGCGACCGCACGCCACGCGGGAACGATGCGCCTATTGCACACAGTAGCAACA 4980

KMP-4_3 (*r8A*) CCGCCTTTAGCGACCGCATGCCACGCGGGAACGATGCGCCTATTGCACACAGTAGCAACA 4980

KMP-4_8 (*r9A*) CCGCCTTTAGCGACCGCACGCCACGCGGGAACGATGCGCCTATTGCACACAGTAGCAACA 4981

KMP-4_13 (*r9B*) CCGCCTTTAGCGACCGCACGCCACGCGGGAACGATGCGCCTATTGCACACAGTAGCAACA 3988

AY198374.1 CCGCCTTTAGCGACCGCACGCCACGCGGGAACGATGCGCCTATTGCACACAGTAGCAACA 5146

KMP-8_35 (*s6B*) CCGCCTTTAGCGACCGCACGCCACGCGGGAACGATGCGCCTATTGCACACAGTAGCAACA 5143

KMP-8_24 (*s6A*) CCGCCTTTAGCGACCGCACGCCACGCGGGAACGATGCGCCTATTGCACACAGTAGCAACA 5143

KMP-8_5 (*s6A*) CCGCCTTTAGCGACCGCACGCCACGCGGGAACGATGCGCCTATTGCACACAGTAGCAACA 5143

KMP-8_46 (*s6A*) CCGCCTTTAGCGACCGCACGCCACGCGGGAACGATGCGCCTATTGCACACAGTAGCAACA 5143

********.********* *****************************************

KMP-5_2 (*r10A*) ACTTCGGTTTCAACACCAGCCCTTTTAGCGCGGAGTTCACTAACAGGCGCATGCGACCAT 4975

KMP-5_11 (*r10B*) ACTTCGGTTTCAACACCAGCCCTTTTAGCGCGGAGTTCACTAACAGGCGCATGCGACCAT 4672

KMP-5_16 (*r10C*) ACTTCGGTTTCAACACCAGCCCTTTTAGCGCGGAGTTCACTAACAGGCGCATGCGACCAT 4782

KMP-5_18 (*r10A*) ACTTCGGTTTCAACACCAGCCCTTTTAGCGCGGAGTTCACTAACAGGCGCATGCGACCAT 4975

KMP-7_3 (*r12C*) ACTTCGGTTTCAACACCAGTCCTTTTAGCGCGGAGTTCACTAACAGGCGCATGCGACCAT 5048

KMP-7_1 (*r12A*) ACTTCGGTTTCAACACCAGTCCTTTTAGCGCGGAGTTCACTAACAGGCGCATGCGACCAT 5207

KMP-7_4 (*r12D*) ACTTCGGTTTCAACACCAGTCCTTTTAGCGCGGAGTTCACTAACAGGCGCATGCGACCAT 4970

KMP-6_7 (*r12A*) ACTTCGGTTTCAACACCAGTCCTTTTAGCGCGGAGTTCACTAACAGGCGCATGCGACCAT 5207

KMP-7_2 (*r12B*) ACTTCGGTTTCAACACCAGTCCTTTTAGCGCGGAGTTCACTAACAGGCGCATGCGACCAT 5086

KMP-6_3 (*r11A*) ACTTCGGTTTCAACACCAGCCCTTTTAGCGCGGAGTTCACTAACAGGCGCATGCGACCAT 5310

KMP-6_5 (*r11B*) ACTTCGGTTTCAACACCAGCCCTTTTAGCGCGGAGTTCACTAACAGGCGCATGCGACCAT 5331

KMP-6_8 (*r11B*) ACTTCGGTTTCAACACCAGCCCTTTTAGCGCGGAGTTCACTAACAGGCGCATGCGACCAT 5335

KMP-8_3 (*s7*) ACTTCGGTTTCAACACCAGTCCTTTTAGCGCGGAGTTCACTAACAGGCGCATGCGACCAT 5203

KMP-4_20 (*r8B*) ACTTCGGTTTCAACACCAGCCCTTTTAGCGCGGAGTTCACTAACAGGCGCATGCGACCAT 5210

KMP-4_16 (*r8A*) ACTTCGGTTTCAACACCAGCCCTTTTAGCGCGGAGTTCACTAACAGGCGCATGCGACCAT 5040

KMP-4_26 (*r8A*) ACTTCGGTTTCAACACCAGCCCTTTTAGCGCGGAGTTCACTAACAGGCGCATGCGACCAT 5040

KMP-4_3 (*r8A*) ACTTCGGTTTCAACACCAGCCCTTTTAGCGCGGAGTTCACTAACAGGCGCATGCGACCAT 5040

KMP-4_8 (*r9A*) ACTTCGGTTTCAACACCAGCCCTTTTAGCGCGGAGTTCACTAACAGGCGCATGCGACCAT 5041

KMP-4_13 (*r9B*) ACTTCGGTTTCAACACCAGCCCTTTTAGCGCGGAGTTCACTAACAGGCGCATGCGACCAT 4048

AY198374.1 ACTTCGGTTTCAACACCAGTCCTTTTAGCGCGGAGTTCACTAACAGGCGCATGCGACCAT 5206

KMP-8_35 (*s6B*) ACTTCGGTTTCAACACCAGTCCTTTTAGCGCGGAGTTCACTAACAGGCGCATGCGACCAT 5203

KMP-8_24 (*s6A*) ACTTCGGTTTCAACACCAGCCCTTTTAGCGCGGAGTTCACTAACAGGCGCATGCGACCAT 5203

KMP-8_5 (*s6A*) ACTTCGGTTTCAACACCAGCCCTTTTAGCGCGGAGTTCACTAACAGGCGCATGCGACCAT 5203

KMP-8_46 (*s6A*) ACTTCGGTTTCAACACCAGCCCTTTTAGCGCGGAGTTCACTAACAGGCGCATGCGACCAT 5203

******************* ****************************************

KMP-5_2 (*r10A*) AG 4977

KMP-5_11 (*r10B*) AG 4674

KMP-5_16 (*r10C*) AG 4784

KMP-5_18 (*r10A*) AG 4977

KMP-7_3 (*r12C*) AG 5050

KMP-7_1 (*r12A*) AG 5209

KMP-7_4 (*r12D*) AG 4972

KMP-6_7 (*r12A*) AG 5209

KMP-7_2 (*r12B*) AG 5088

KMP-6_3 (*r11A*) AG 5312

KMP-6_5 (*r11B*) AG 5333

KMP-6_8 (*r11B*) AG 5337

KMP-8_3 (*s7*) AG 5205

KMP-4_20 (*r8B*) AG 5212

KMP-4_16 (*r8A*) AG 5042

KMP-4_26 (*r8A*) AG 5042

KMP-4_3 (*r8A*) AG 5042

KMP-4_8 (*r9A*) AG 5043

KMP-4_13 (*r9B*) AG 4050

AY198374.1 AG 5208

KMP-8_35 (*s6B*) AG 5205

KMP-8_24 (*s6A*) AG 5205

KMP-8_5 (*s6A*) AG 5205

KMP-8_46 (*s6A*) AG 5205

**
