# Supplementary material for: Alternative Splicing and Highly Variable Cadherin Transcripts Associated with Field-Evolved Resistance of Pink Bollworm to Bt Cotton in India
Source: PLoS One. 2014 May 19;9(5):e97900. doi: 10.1371/journal.pone.0097900 (PMC4026531; doi:10.1371/journal.pone.0097900)
Supplement: Figure S6 — Alignment of predicted amino acid sequences of pink bollworm cadherin from Khandwa, Madhya Pradesh (KMP) with PgCad1 s (AY198374.1). Stars show amino acids conserved in all of the sequences. The symbols “:” and “.” indicate conservative amino acid substitutions scoring >0.5 and ≤0.5 in the Gonnet PAM 250 matrix, respectively. Red boxes show amino acids corresponding to lepidopteran cadherin Cry1Ac toxin binding regions. (DOCX) [file pone.0097900.s006.docx]

**Figure S6. Alignment of predicted amino acid sequences of pink bollworm cadherin from Khandwa, Madhya Pradesh (KMP) with *PgCad1* *s* (AY198374.1).** Stars show amino acids conserved in all of the sequences. The symbols “:” and “.” indicate conservative amino acid substitutions scoring >0.5 and ≤0.5 in the Gonnet PAM 250 matrix, respectively. Red boxes show amino acids corresponding to lepidopteran cadherin Cry1Ac toxin binding regions.

KMP-4_20 (*r8B*) MAGDACILVTVLLTFATSVFGQETTSSRCYYMTDAIPREPKPDDLPDLEWTGGWTDWPLI 60

KMP-4_16 (*r8A*) MAGDACILVTVLLTFATSVFGQETTSSRCYYMTDAIPREPKPDDLPDLEWTGGWTDWPLI 60

KMP-4_3 (*r8A*) MAGDACILVTVLLTFATSVFGQETTSSRCYYMTDAIPREPKPDDLPDLEWTGGWTDWPLI 60

KMP-4_26 (*r8A*) MAGDACILVTVLLTFATSVFGQETTSSRCYYMTDAIPREPKPDDLPDLEWTGGWTDWPLI 60

KMP-4_8 (*r9A*) MAGDACILVTVLLTFATSVFGQETTSSRCYYMTDAIPREPKPDDLPDLEWTGGWTDWPLI 60

KMP-4_13 (*r9B*) MAGDACILVTVLLTFATSVFGQETTSSRCYYMTDAIPREPKPDDLPDLEWTGGWTDWPLI 60

KMP-5_2 (*r10A*) MAGDACILVTVLLTFATSVFGQETTSSRCYYMTDAIPREPKPDDLPDLEWTGGWTDWPLI 60

KMP-5_16 (*r10C*) MAGDACILVTVLLTFATSVFGQETTSSRCYYMTDAIPREPKPDDLPDLEWTGGWTDWPLI 60

KMP-5_18 (*r10A*) MAGDACILVTVLLTFATSVFGQETTSSRCYYMTDAIPREPKPDDLPDLEWTGGWTDWPLI 60

KMP-5_11 (*r10B*) MAGDACILVTVLLTFATSVFGQETTSSRCYYMTDAIPREPKPDDLPDLEWTGGWTDWPLI 60

KMP-6_7 (*r12A*) MAGDACILVTVLLTFATSVFGQEAASSRCYYMTDAIPREPKPDDLPDLEWTGGWTDWPLI 60

KMP-7_1 (*r12A*) MAGDACILVTVLLTFATSVFGQETASSRCYYMTDAIPREPKPDDLPDLEWTGGWTDWPLI 60

KMP-7_2 (*r12B*) MAGDACILVTVLLTFATSVFGQET-SSRCYYMTDAIPGEPKPDDLPDLEWTGGWTDWPLI 59

KMP-6_3 (*r11A*) MAGDACILVTVLLTFATSVFGQETASSRCYYMTDAIPREPKPDDLPDLEWTGGWTDWPLI 60

KMP-6_5 (*r11B*) MAGDACILVTVLLTFATSVFGQETASSRCYYMTDAIPREPKPDDLPDLEWTGGWTDWPLI 60

KMP-6_8 (*r11B*) MAGDACILVTVLLTFATSVFGQETASSRCYYMTDAIPREPKPDDLPDLEWTGGWTDWPLI 60

KMP-7_3 (*r12C*) MAGDACILVTVLLTFATSVFGIVEMLLHD------------------------------- 29

KMP-7_4 (*r12D*) MAGDACILVTVLLTFATSVFGIVEMLLHD------------------------------- 29

KMP-8_3 (*s7*) MAGDACILVTVLLTFATSVFGQET-SSRCYYMTDAIPREPKPDDLPDLEWTGGWTDWPLI 59

KMP-8_24 (*s6A*) MAGDACILVTVLLTFATSVFGQET-SSRCYYMTDAIPREPKPDDLPDLEWTGGWTDWPLI 59

KMP-8_5 (*s6A*) MAGDACILVTVLLTFATSVFGQET-SSRCYYMTDAIPREPKPDDLPDLEWTGGWTDWPLI 59

KMP-8_46 (*s6A*) MAGDACILVTVLLTFATSVFGQET-SSRCYYMTDAIPREPEPDDLPDLEWTGGWTDWPLI 59

KMP-8_35 (*s6B*) MAGDACILVTVLLTFATSVFGQETASSRCYYMTDAIPREPKPDDLPDLEWTGGWTDWPLI 60

AY198374.1 MAGDACILVTVLLTFATSVFGQETTSSRCYYMTDAIPREPKPDDLPDLEWTGGWTDWPLI 60

********************* :

KMP-4_20 (*r8B*) PAEPRDDVCINGWYPQLTSTSLGTIIIHMEEEIEGDVAIAKLNYDGSGTPEIVQPMVIGS 120

KMP-4_16 (*r8A*) PAEPRDDVCINGWYPQLTSTSLGTIIIHMEEEIEGDVAIAKLNYDGSGTPEIVQPMVIGS 120

KMP-4_3 (*r8A*) PAEPRDDVCINGWYPQLTSTSLGTIIIHMEEEIEGDVAIAKLNYDGSGTPEIVQPMVIGS 120

KMP-4_26 (*r8A*) PAEPRDDVCINGWYPQLTSTSLGTIIIHMEEEIEGDVAIAKLNYDGSGTPEIVQPMVIGS 120

KMP-4_8 (*r9A*) PAEPRDDVCINGWYPQLTSTSLGTIIIHMEEEIEGDVAIAKLNYDGSGTPEIVQPMVIGS 120

KMP-4_13 (*r9B*) PAEPRDDVCINGWYPQLTSTSLGTIIIHMEEEIEGDVAIAKLNYDGSGTPEIVQPMVIGS 120

KMP-5_2 (*r10A*) PAEPRDDVCIDGWYPQLTSTPLGTIIIHMEEEIEGDVAIAKLNYDGSGTPEIVQPMVVGS 120

KMP-5_16 (*r10C*) PAEPRDDVCINGWYPQLTSTSLGTIIIHMEEEIEGDVAIAKLNYDGSGTPEIVQPMVIGS 120

KMP-5_18 (*r10A*) PAEPRDDVCINGWYPQLTSTSLGTIIIHMEEEIEGDVAIAKLNYDGSGTPEIVQPMVIGS 120

KMP-5_11 (*r10B*) PAEPRDDVCINGWYPQLTSTSLGTIIIHMEEEIEGDVAIAKLNYDGSGTPEIVQPMVIGS 120

KMP-6_7 (*r12A*) PAEPRDDVCINGWYPQLTSTSLGTIISHMEEEIEGDVAIAKLNYDGSGTPEIVQPMVIGS 120

KMP-7_1 (*r12A*) PAEPRDDVCINGWYPQLTSTSLGTIIIHMEEEIEGDAAIAKLNYDGSGTPEIVQPMVIGS 120

KMP-7_2 (*r12B*) PAEPRDDVCINGWYPQLTSTSLGTIIIHMEEEIEGDVAIAKLNYDGSGTPEIVQPMVIGS 119

KMP-6_3 (*r11A*) PAEPRDDVRINGWYPQLTSTSLGTIIIHMEEEIEGDVAIAKLNYDGSGTPEIVQPMVIGS 120

KMP-6_5 (*r11B*) PAEPRDDVCINGWYPQLTSTSLGTIIIHMEEEIEGDVAIAKLNYDGSGTSEIVQPMVIGS 120

KMP-6_8 (*r11B*) PAEPRDDVCINGWYPQLTSTSLGTIIIHMEEEIEGDVAIAKLSYDGSGTPEIVQPMVIGS 120

KMP-7_3 (*r12C*) ------------------------------------------------------------ 29

KMP-7_4 (*r12D*) ------------------------------------------------------------ 29

KMP-8_3 (*s7*) PAEPRDDVCINGWYPQLTSTSLGTIIIHMEEEIEGDVAIAKLNYDGSGTPEIVQPMVIGS 119

KMP-8_24 (*s6A*) PAEPRDDVCINGWYPQLTSTSLGTIIIHMEEEIEGDVAIAKLNYDGSGTPEIVQPMVIGS 119

KMP-8_5 (*s6A*) PAEPRDDVCINGWYPQLTSTSLGTIIIHMEEEIEGDVAIAKLNYDGSGTPEIVQPMVIGS 119

KMP-8_46 (*s6A*) PAEPRDDVCINGWYPQLTSTSLGTIIIHMEEEIEGDVAIAKLNYDGSGTPEIVQPMVIGS 119

KMP-8_35 (*s6B*) PAEPRDDVCINGWYPQLTSTSLGTIIIHMEEEIEGDVAIAKLNYDGSGTPEIVQPMVIGS 120

AY198374.1 PAEPRDDVCINGWYPQLTSTSLGTIIIHMEEEIEGDVAIAKLNYDGSGTPEIVQPMVIGS 120

KMP-4_20 (*r8B*) FNLLSPEIRNENGAWYLYITNRQDYETPTMRRYTFDVRVPDETRAARVSLSIENIDDNDP 180

KMP-4_16 (*r8A*) SNLLSPEIRNENGAWYLYITNRQDYETPTMRRYTFDVRVPDETRAARVSLSIENIDDNDP 180

KMP-4_3 (*r8A*) FNLLSPEIRNENGAWYLYITNRQDYETPTMRRYTFDVRVPDETRAARVSLSIENIDDNDP 180

KMP-4_26 (*r8A*) FNLLSPEIRNENGAWYLYITNRQDYETPTMRRYTFDVRVPDETRAARVSLSIENIDDNDP 180

KMP-4_8 (*r9A*) FNLLSPEIRNENGAWYLYITNRQDYETPTMRRCTFDVRVPDETRAAQVSLSIENIDDNDP 180

KMP-4_13 (*r9B*) FNLLSPEIRNENGAWYLYITNRQDYEAPTMRRYTFDVRVPDETRAARVSLSIENIDDNDP 180

KMP-5_2 (*r10A*) FNLLSPEIRNENGAWYLYITNRQDYETPTMRRYTFDVRVPDETRAARVSLSIENIDDNDP 180

KMP-5_16 (*r10C*) FNLLSPEIRNENGAWYLYITNRQDYETPTMRRYTFDVRVPDETRAARVSLSIENIDDNDP 180

KMP-5_18 (*r10A*) FNLLSPEIRNENGAWYLYITNRQDYETPTMRRYTFDVRVPDETRAARVSLSIENIDDNDP 180

KMP-5_11 (*r10B*) FNLLSPEIRNENGAWYLYITNRQDYETPTMRRYTFDVRVPDETRAARVSLSIENIDDNDP 180

KMP-6_7 (*r12A*) FNLLSPEIRNENGAWYLYITNRQDYETPTMRRYTFDVRVPDETRAARVSLSIENIDDNDP 180

KMP-7_1 (*r12A*) FNLLSPEIRNENGAWYLYITNRQDYETPTMRRYTFDVRVPDETRAARVSLSIENIDDNDP 180

KMP-7_2 (*r12B*) FNLLSPEIRNENGAWYLYITNRQDYETPTMRRYTFDVRVPDETRAARVSLSIENIDDNDP 179

KMP-6_3 (*r11A*) FNLLSPEIRNENGAWYLYITNRQDYETPTMRRYTFDVRVPDETRAARVSLSIENIDDNDP 180

KMP-6_5 (*r11B*) FNLLSPEIRNENGAWYLYITNRQDYETPTMRRYTFDVRVPDETRAARVSLSIENIDDNDP 180

KMP-6_8 (*r11B*) FNLLSPEIRNENGAWYLYITNRQDYETPTMRRYTFDVRVPDETRAARVSLSIENIDDNDP 180

KMP-7_3 (*r12C*) ------------------------------------------------------------ 29

KMP-7_4 (*r12D*) ------------------------------------------------------------ 29

KMP-8_3 (*s7*) FNLLSPEIRNENGAWYLYITNRQDYETPTMRRYTFDVRVPDETRAARVSLSIENIDDNDP 179

KMP-8_24 (*s6A*) FNLLSPEIRNENGAWYLYITNRQDYETPTMRRYTFDVRVPDETRAARVSLSIENIDDNDP 179

KMP-8_5 (*s6A*) FNLLSPEIRNENGAWYLYITNRQDYETPTMRRYTFDVRVPDETRAARVSLSIENIDDNDP 179

KMP-8_46 (*s6A*) FNLLSPEIRNENGAWYLYITNRQDYETPTMRRYTFDVRVPDETRAARVSLSIENIDDNDP 179

KMP-8_35 (*s6B*) FNLLSPEIRNENGAWYLYITNRQDYETPTMRRYTFDVRVPDETRAARVSLSIENIDDNDP 180

AY198374.1 FNLLSPEIRNENGAWYLYITNRQDYETPTMRRYTFDVRVPDETRAARVSLSIENIDDNDP 180

KMP-4_20 (*r8B*) IVRVLDACQVPELGEPRLTDCVYQVSDEDGRLSIEPMTFRLTSDREDVQIFYVEPAHITG 240

KMP-4_16 (*r8A*) IVRVLDACQVPELGEPRLTDCVYQVSDEDGRLSIEPMTFRLTSDREDVQIFYVEPAHITG 240

KMP-4_3 (*r8A*) IVRVLDACQVPELGEPRLTDCVYQVSDEDGRLSIEPMTFRLTSDREDVQIFYVEPAHITG 240

KMP-4_26 (*r8A*) IVRVLDACQVPELGEPRLTDCVYQVSDEDGRLSIGPMTFRLTSDREDVQIFYVEPAHITG 240

KMP-4_8 (*r9A*) IVRVLDACQVPELGEPRLTDCVYQVSDEDGRLSIEPMTFRLTSDREDVQIFYVEPAHITG 240

KMP-4_13 (*r9B*) IVRVLDACQVPELGEPRLTDCVYQVSDEDGRLSIEPMTFRLTSDREDVQIFYVEPAHITG 240

KMP-5_2 (*r10A*) IVRVLDACQVPELGEPRLTDCVYQVSDEDGRLSIEPMTFRLTSDREDVQIFYVEPAHITG 240

KMP-5_16 (*r10C*) IVRVLDACQVPELGEPRLTDCVYQVSDEDGRLSIEPMTFRLTSDREDVQIFYVEPAHITG 240

KMP-5_18 (*r10A*) IVRVLDACQVPELGEPRLTDCVYQVSDEDGRLSIEPMTFRLTSDREDVQIFYVEPAHITG 240

KMP-5_11 (*r10B*) IVRVLDACQVPELGEPRLTDCVYQVSDEDGRLSIEPMTFRLTSDREDVQIFYVEPAHITG 240

KMP-6_7 (*r12A*) IVSVLDACQVPELGEPRLTDCVYQVSDEDGRLSIEPMTFRLTSDREDVQIFYVEPAHITG 240

KMP-7_1 (*r12A*) IVRVLDACQVPELGEPRLTDCVYQVSDEDGRLSIEPMTFRLTSDREDVQIFYVEPAHITG 240

KMP-7_2 (*r12B*) IVRVLDACQVPELGEPRLTDCVYQVSDEDGRLSIEPMTFRLTSDREDVQIFYVEPAHITG 239

KMP-6_3 (*r11A*) IVSVLDACQVPELGEPRLTDCVYQVSDEDGRLSIEPMTFRLTSDREDVQIFYVEPAHITG 240

KMP-6_5 (*r11B*) IVRVLDACQVPELGEPRLTDCVYQVSDEDGRLSIEPMTFRLTSDREDVQIFYVEPAHITG 240

KMP-6_8 (*r11B*) IVRVLDACQVPELGEPRLTDCVYQVSDEDGRLSIEPMTFRLTSDREDVQIFYVEPAHITG 240

KMP-7_3 (*r12C*) ------------------------------------------------------------ 29

KMP-7_4 (*r12D*) ------------------------------------------------------------ 29

KMP-8_3 (*s7*) IVRVLDACQVPELGEPRLTDCVYQVSDEDGRLSIEPMTFRLTSDREDVQIFYVEPAHITG 239

KMP-8_24 (*s6A*) IVRVLDACQVPELGEPRLTDCVYQVSDEDGRLSIEPMTFRLTSDREDVQIFYVEPAHITG 239

KMP-8_5 (*s6A*) IVRVLDACQVPELGEPRLTDCVYQVSDEDGRLSIEPMTFRLTSDREDVQIFYVEPAHITG 239

KMP-8_46 (*s6A*) IVRVLDACQVPELGEPRLTDCVYQVSDEDGRLSIEPMTFRLTSDREDVQIFYVEPAHITG 239

KMP-8_35 (*s6B*) IVRVLDACLVPELGEPRLTDCVYQVSDEDGRLSIEPMTFRLTSDREDVQIFYVEPAHITG 240

AY198374.1 IVRVLDACQVPELGEPRLTDCVYQVSDEDGRLSIEPMTFRLTSDREDVQIFYVEPAHITG 240

KMP-4_20 (*r8B*) DWFNMQITIGILSALNFESNPLHIFQITALDSWPNNHTVTVMVQVQNVEHRPPRWMEIFA 300

KMP-4_16 (*r8A*) DWFNMQITIGILSALNFESNPLHIFQITALDSWPNNHTVTVMVQVQNVEHRPPRWMEISA 300

KMP-4_3 (*r8A*) DWFNMQITIGILSALNFESNPLHIFQITALDSWPNNHTVTVMVQVQNVEHRPPRWMEIFA 300

KMP-4_26 (*r8A*) DWFNMQITIGILSALNFESNPLHIFQITALDSWPNNHTVTVMVQVQNVEHRPPRWMEIFA 300

KMP-4_8 (*r9A*) DWFNMQITIGILSALNFESNPLHIFQITALDSWPNNHTVTVMVQVQNVEHRPPRWMEIFA 300

KMP-4_13 (*r9B*) DWFNMQITIGILSALNFESNPLHIFQITALDSWPNNHTVTVMVQVQNVEHRPPRWMEIFA 300

KMP-5_2 (*r10A*) DWFNMQIIIGILSALNFESNPLHIFQITALDSWPNNHTVTVMVQVQNVEHRPPRWMEIFA 300

KMP-5_16 (*r10C*) DWFNMQITIGILSALNFESNPLHIFQITALDSWPNNHTVTVMVQVQNVEHRPPRWMEIFA 300

KMP-5_18 (*r10A*) DWFNMQITIGILSALNFESNPLHILQITALDSWPNNHTVTVMVQVQNVEHRPPRWMEIFA 300

KMP-5_11 (*r10B*) DWFNMQITIGILSALNFESNPLHIFQITALDSWPNNHTVTVMVQVQNVEHRPPRWMEIFA 300

KMP-6_7 (*r12A*) DWFNMQITIGILSALNFESNPLHIFQITALDSWPNNHTVTVMVQVQNVEHRPPRWMEIFA 300

KMP-7_1 (*r12A*) DWFNMQITIGILSALNFESNPLHIFQITALDSWPNNHTVTVMVQVQNVEHRPPRWMEIFA 300

KMP-7_2 (*r12B*) DWFNMQITIGILSALNFESNPLHIFQITALDSWPNNHTVTVMVQVQNVEHRPPRWMEIFA 299

KMP-6_3 (*r11A*) DWFNMQITIGILSALNFESNPLHIFQITALDSWPNNHTVTVMVQVQNVEHRPPRWMEIFA 300

KMP-6_5 (*r11B*) DWFNMQITIGILSALNFESNPLHIFQITALDSWPNNHTVTVMVQVQNVEHRPPRWMEIFA 300

KMP-6_8 (*r11B*) DWFNMQITIGILSALNFESNPLHIFQITALDSWPNNHTVTVMVQVQNVEHRPPRWMEIFA 300

KMP-7_3 (*r12C*) ------------------------------------------------------------ 29

KMP-7_4 (*r12D*) ------------------------------------------------------------ 29

KMP-8_3 (*s7*) DWFNMQITIGILSALNFESNPLHIFQITALDSWPNNHTVTVMVLVQNVEHRPPRWMEIFA 299

KMP-8_24 (*s6A*) DWFNMQITIGILSALNFESNPLHIFQITALDSWPNNHTVTVMVQVQNVEHRPPRWMEIFA 299

KMP-8_5 (*s6A*) DWFNMQITIGILSALNFESNPLHIFQITALDSWPNNHTVTVMVQVQNVEHRPPRWMEIFA 299

KMP-8_46 (*s6A*) DWFNMQITIGILSALNFESNPLHIFQITALDSWPNNHTVTVMVKVRNVEHRPPRWMEIFA 299

KMP-8_35 (*s6B*) DWFNMQITIGILSALNFESSPLHIFQITALDSWPNNHTVTVMVQVQNVEHRPPRWMEIFA 300

AY198374.1 DWFNMQITIGILSALNFESNPLHIFQITALDSWPNNHTVTVMVQVQNVEHRPPRWMEIFA 300

KMP-4_20 (*r8B*) VQQFDEMTEQQFQVRAIDGDTGIGKAIHYTLETDEEEDLFFIETLPGGHDGAIFSTAMFD 360

KMP-4_16 (*r8A*) VQQFDEMTEQQFQVRAIDGDTGIGKAIHYTLETDEEEDLFFIETLPGGHDGAIFSTAMID 360

KMP-4_3 (*r8A*) VQQFDEMTEQQSQVRAIDGDTGIGKAIHYTLETDEEEDLYFIETLPGGHDGAIFSTAMID 360

KMP-4_26 (*r8A*) VQQFDEMTEQQFQVRAIDGDTGIGKAIHYTLETDEEEDLYFIETLPGGHDGAIFSTAMID 360

KMP-4_8 (*r9A*) VQQFDEMTEQQFQVRAIDGDTGIGKAIHYTLETDEEEDLFFIETLPGGHDGAIFSTATID 360

KMP-4_13 (*r9B*) VQQFDEMTEQQFQVRAIDGDTGIGKAIHYTLETDEEEDLFFIETLPGGHDGAIFSTAMID 360

KMP-5_2 (*r10A*) VQQFDEITEQQFQVRAIDGDTGIGKAIHYTLETDEEGDLFFIETLPGGHDGAIFSTAMID 360

KMP-5_16 (*r10C*) VQQFDEMTEQQFQVRAIDGDTGIGKAIHYTLETDEEEDLFFIETLPGGHDGAIFSTAMID 360

KMP-5_18 (*r10A*) VQQFDEMTEQQFQVRAIDGDTGIGKAIHYTLETDEEEDLFFIETLPGGHDGAIFSTAMID 360

KMP-5_11 (*r10B*) VQQFDEMTEQQFQVRAIDGDTGIGKAIHYTLETDEEEDLFFIETLPGGHDGAIFSTAMID 360

KMP-6_7 (*r12A*) VQQFDEMTEQQFQVRAIDGDTGIGKAIHYTLETDEEEDLFFIETLPGGHDGAIFSTAMID 360

KMP-7_1 (*r12A*) VQQFDEMTEQQFQVRAIDGDTGIGKAIHYTLETDEEEDLFFIETLPGGHDGAIFSTAMID 360

KMP-7_2 (*r12B*) VQQFDEMTEQQFQVRAIDGDTGIGKAIHYTLETDEEEDLFFIETLPGGHDGAIFSTAMID 359

KMP-6_3 (*r11A*) VQQFDEMTEQQFQVRAIDGDTGIGKAIHYTLETDEEEDLFFIETLPGGHDGAIFSTAMID 360

KMP-6_5 (*r11B*) VQQFDEMTEQQFQVRAIDGDTGIGKAIHYTLETDEEEDLFFIETLPGGHDGAIFSTAMID 360

KMP-6_8 (*r11B*) VQQFDEMTEQQFQVRAIDGDTGIGKAIHYTLETDEEEDLFFIETLPGGHDGAIFSTAMID 360

KMP-7_3 (*r12C*) ------------------------------------------------------------ 29

KMP-7_4 (*r12D*) ------------------------------------------------------------ 29

KMP-8_3 (*s7*) VQQFDEMTEQQFQVRAIDGDTGIGKAIHYTLETDEEEDLFFIETLPGGHDGAIFSTAMID 359

KMP-8_24 (*s6A*) VQQFDEMTEQQFQVRAIDGDTGIGKAIHYTLETDEEEDLFFIETLPGGHDGAIFSTAMID 359

KMP-8_5 (*s6A*) VQQFDEMTEQQFQVRAIDGDTGIGKAIHYALETDEEEDLFFIETLPGGHDGAIFSTAMID 359

KMP-8_46 (*s6A*) VQRFDEMTEQQFQVRAIDGDTGIGKAIHYTLETDEVEDLFFIETLPGGHDGAIFSTAMID 359

KMP-8_35 (*s6B*) VQQFDEMTEQQFQVRAIDGDTGIGKAIHYTLETDE-EDLFFIETLPGGHDGAIFSTAMID 359

AY198374.1 VQQFDEMTEQQFQVRAIDGDTGIGKAIHYTLETDEEEDLFFIETLPGGHDGAIFSTAMID 360

KMP-4_20 (*r8B*) VDRLRRDVFRLSLVAYKYDNVSFATPTPVVIIVNDINNKKPQPLQDEYTISIMEETPLSL 420

KMP-4_16 (*r8A*) VDRLRRDVFRLSLVAYKYDNVSFATPTPVVIIVNDINNKKPQPLQDEYTISIMEETPLSL 420

KMP-4_3 (*r8A*) VDRLRRDVFRLSLVAYKYDNVSFATPTPVVIIVNDINNKKPQPLQDEYTISIMEETPLSL 420

KMP-4_26 (*r8A*) VDRLRRDVFRLSLVAYKYDNVSFATPTPVVIIVNDINNKKPQPLQDEYTISIMEETPLSL 420

KMP-4_8 (*r9A*) GDRLRRDVFRLSLVAYKYDNVSFATPTPVVIIVNDINNKKPQPLQDEYTISIMEETPLSL 420

KMP-4_13 (*r9B*) VDRLRRDVFRLSLVAYKYDNVSFATPTPVVIIVNDINNKKPQPLQDEYIISIMEETPLSL 420

KMP-5_2 (*r10A*) VDRLRRDVFRLSLVAYKYDNVSFATPTPVVIIVNDINNKKPQPLQDEYTISIMEETPLSL 420

KMP-5_16 (*r10C*) VDRLRRDVFRLPLVAYKYDNVSFATPTPVVIIVNDINNKKPQPLQDEYTISIMEETPLSL 420

KMP-5_18 (*r10A*) VDRLRRDVFRLSLVAYKYDNVSFATPTPVVIIVNDINNKKPQPLQDEYTISIMEETPLSL 420

KMP-5_11 (*r10B*) VDRLRRDVFRLSLVAYKYDNVSFATPTPVVIIVNDINNKKPQPLQDEYTISIMEETPLSL 420

KMP-6_7 (*r12A*) VDRLRRDVFRLSLVAYKYDNVSFATPTPVVIIVNDINNKQPQPLQDEYTISIMEETPLSL 420

KMP-7_1 (*r12A*) VDRLRRDVFRLSLVAYKYDNVSFATPTPVVIIVNDINNKQPQPLQDECTISIMEETPLSL 420

KMP-7_2 (*r12B*) VDRLRRDVFRLSLVAYKYDNVSFATPTPVVIIVNDINNKQPQPLQDEYTISIMEETPLSL 419

KMP-6_3 (*r11A*) VDRLRRDVFRLSLVAYKYDNVSFATPTPVVIIVNDINNKQPQPLQDEYTISIMEETPLSL 420

KMP-6_5 (*r11B*) VDRLRRDVFRLSLVAYKYDNVSFATPTPVVIIVNDINNKKPQPLQDEYTISIMEETPLSL 420

KMP-6_8 (*r11B*) VDRLRRDVFRLSLVAYKYDNVSFATPTPVVIIVNDINNKKPQPLQDEYTISIMEETPLSL 420

KMP-7_3 (*r12C*) ------------------------------------------------------------ 29

KMP-7_4 (*r12D*) ------------------------------------------------------------ 29

KMP-8_3 (*s7*) VDRLRRDVFRLSLVAYKYDNVSFATPTPVVIIVNDINNKQPQPLQDEYTISIMEETPLSL 419

KMP-8_24 (*s6A*) VDRLRRDVFRLSLVAYKYDNVSFAAPTPVVIIVNDINNKKPQPLQDEYTISIMEETPLSL 419

KMP-8_5 (*s6A*) VDRLRRDVFRLSLVAYKYDNVSFATPTPVVIIVNDINNKKPQPLQDEYTISIMEETPLSL 419

KMP-8_46 (*s6A*) VDRLRRDVFRLSLVAYKYDNVSFATPTPVVIIVNDINNKKPQPLQDEYTISIMEETPLSL 419

KMP-8_35 (*s6B*) VDRLRRDVFRLSLVAYKYDNVSFATPTPVVIIVNDINNKKPQPLQDEYTNSIMEETPLSL 419

AY198374.1 VDRLRRDVFRLSLVAYKYDNVSFATPTPVVIIVNDINNKKPQPLQDEYTISIMEETPLSL 420

KMP-4_20 (*r8B*) NFAELFGFYDEDLIYAQFLVEIQGENPPGVGQAFYIAPTAGFQNQTFAIGTQDHRMLDYE 480

KMP-4_16 (*r8A*) NFAELFGFYDEDLIYAQFLVEIQGENPPGVEQAFYIAPTAGFQNQTFAIGTQDHRMQDYE 480

KMP-4_3 (*r8A*) NFVELFGFYDEDLIYAQFLVEIQGENPPGAEQAFYIAPTAGFQNQTFAIGTQDHRMLDYE 480

KMP-4_26 (*r8A*) NFAELFGFYDEDLIYAQFLVEIQGENPPGVEQAFYIAPTAGFQNQTFAIGTQDHRMLDYE 480

KMP-4_8 (*r9A*) NFAELFGFYDEDLIYAQFLVEIQGENPPGVEQAFYIAPTAGFQNQTFAIGTQDHRMLDYE 480

KMP-4_13 (*r9B*) NFAELFGFYDEDLIYAQFLVEIQGENPPGVEQAFYIAPTAGFQNQTFAIGTQDHRMLDYE 480

KMP-5_2 (*r10A*) NFAELFGFYDEDLIYAQFLVEIQGENPPGVEQAFYIAPTAGFQNQTFAIGTQDHRMLDYE 480

KMP-5_16 (*r10C*) NFAELFGFYDEDLIYAQFLVEIQGENPPGVEQAFYIAPTAGFQNQTFAIGTQDHRMLDYE 480

KMP-5_18 (*r10A*) NFAELFGFYDEDLIYAQFLVEIQGENPPGVEQAFYIAPTAGFQNQTFAIGTQDHRMLDYE 480

KMP-5_11 (*r10B*) NFAELFGFYDEDLIYAQFLVEIQGENPPGVEQAFYIAPTAGFQNQTFAIGTQDHRMLDYE 480

KMP-6_7 (*r12A*) DFAELFGFYDEDLIYAQFLVEIQGENPPGVEQAFYIAPTAGFQNQTFAIGTQDHRMLDYE 480

KMP-7_1 (*r12A*) NFAELFGFYDEDLIYAQFLVEIQGENPPGVEQAFYIAPTAGFQNQTFAIGTQDHRMLDYE 480

KMP-7_2 (*r12B*) NFAELFGFYDEDLIYAQFLVEIQGENPPGVEQAFYIAPTAGFQNQTFAIGTQDHRMLDYE 479

KMP-6_3 (*r11A*) NFAELFGFYDEDLIYAQFLVEIQGENPPGVEQAFYIAPTAGFQNQTFAIGTQDHRMLDYE 480

KMP-6_5 (*r11B*) NFAELFGFYDEDLIYAQFLVEIQGENPPGVEQAFYIAPTAGFQNQTFAIGTQDHRMLDYE 480

KMP-6_8 (*r11B*) NFAELFGFYDEDLIYAQFLVEIQGENPPGVEQAFYIAPTAGFQNQTFAIGTQDHRMLDYE 480

KMP-7_3 (*r12C*) ------------------------------------------------------------ 29

KMP-7_4 (*r12D*) ------------------------------------------------------------ 29

KMP-8_3 (*s7*) NFAELFGFYDEDLIYAQFLVEIQGENPPGVEQAFYIAPTAGFQNQTFAIGTQDHRMLDYE 479

KMP-8_24 (*s6A*) NFAELFSFYDEDLIYARFLVGIQGENPPGVEQAFYIAPTAGFQNQTFAIGTQDHRMLDYE 479

KMP-8_5 (*s6A*) NFAELFGFYDEDLIYAQFLVEIQGENPPGVEQAFYIAPTAGFQNQTFAIGTQDHRMLDYE 479

KMP-8_46 (*s6A*) NFAELFGFYDEDLIYAQFLVEIQGEDPPGVEQAFYIAPTAGFQNQTFAIGTQDHRMLDYE 479

KMP-8_35 (*s6B*) NFAELFGFYDEDLIYAQFLVEIQGENPPGVEQAFYIAPTAGFQNQTFAIGTQDHRMLDYE 479

AY198374.1 NFAELFGFYDEDLIYAQFLVEIQGENPPGVEQAFYIAPTAGFQNQTFAIGTQDHRMLDYE 480

KMP-4_20 (*r8B*) DVPFQNIKLKVIATDRDNTNFTGVAEVNVNLINWNDEEPIFEEDQLVVKFKETVPKDYHV 540

KMP-4_16 (*r8A*) DVPFQNIKLKVIATDRDNTNFTGVAEVNVNLINWNDEEPIFEEDQLVVKFKETVPKDYHV 540

KMP-4_3 (*r8A*) DVPFQNIKLKVIATDRDNTNFTGVAEVNVNLINWNDEEPIFEEDQLVVKFKETVPKDYHV 540

KMP-4_26 (*r8A*) DVPFQNIKLKVIATDRDNTNFTGVAEVNVNLINWNDEEPIFEEDQLVVKFKETVPKDYHV 540

KMP-4_8 (*r9A*) DVPFQNIKLKVIATDRDNTNFTGVAEVNVNLINWNDEEPIFEEDQLVVKFKETVPKDYHV 540

KMP-4_13 (*r9B*) DVPFQNIKLKVIATDRDNTNFTGVAEVNVNLINWNDEEPIFEEDQLVVKFKETVPKDYHV 540

KMP-5_2 (*r10A*) DVPFQNIKLKVIATDRDNTNFTGVAEVNVNLINWNDEEPIFEEDQLVVKFKETVPKDYHV 540

KMP-5_16 (*r10C*) DVPFQNIKLKVIATDRDNTNFTGVAEVNVNLINWNDEEPIFEEDQLVVKFKETVPKDYHV 540

KMP-5_18 (*r10A*) DVPFQNIKLKVIATDRDNTNFTGVAEVNVNLINWNDEEPIFEEDQLVVKFKETVPKDYHV 540

KMP-5_11 (*r10B*) DVPFQNIKLKVIATDRDNTNFTGVAEVNVNLINWNDEEPIFEEDQLVVKFKETVPKDYHV 540

KMP-6_7 (*r12A*) DVPFQNIKLKVIATDRDNTNFTGVAEVNVNLINWNDEEPIFEEDQLVVKFKETVPKDYHV 540

KMP-7_1 (*r12A*) DVPFQNIKLKVIATDRDNTNFTGVAEVNVNLINWNDEEPIFEEDQLVVKFKETVPKDYHV 540

KMP-7_2 (*r12B*) DVPFQNIKLKVIATDRDNTNFTGVAEVNVNLINWNDEEPIFEEDQLVVKFKETVPKDYHV 539

KMP-6_3 (*r11A*) DVPFQNIKLKVIATDRDNTNFTGVAEVNVNLINWNDEEPIFEEDQLVVKFKETVPKDYHV 540

KMP-6_5 (*r11B*) DVPFQNIKLKVIATDRDNTNFTGVAEVNVNLINWNDEEPIFEEDQLVVKFKETVPKDYHV 540

KMP-6_8 (*r11B*) DVPFQNIKLKVIATDRDNTNFTGVAEVNVNLINWNDEEPIFEEDQLVVKFKETVPKDYHV 540

KMP-7_3 (*r12C*) ------------------------------------------------------------ 29

KMP-7_4 (*r12D*) ------------------------------------------------------------ 29

KMP-8_3 (*s7*) DVPFQNIKLKVIATDRDNTNFTGVAEANVILINWNDEEPIFEEDQLVVKFKETVPKDYHV 539

KMP-8_24 (*s6A*) DVPFQNIKLKVIATDRDNTNFTGVAEVNVNLINWNDEEPIFEEDQLVVKFKETVPKDYHV 539

KMP-8_5 (*s6A*) DVPFQNIKLKVIATDRDNTNFTGVAEVNVNLINWNDEEPIFEEDQLVVKFKETVPKDYHV 539

KMP-8_46 (*s6A*) DVPFQNIKLKVIATDRDNTNFTGVAEVNVNLINWNDEEPIFEEDQLVVKFKETVPKDYHV 539

KMP-8_35 (*s6B*) DVPFQNIKLKVIATDRDNTNFTGVAEVNVNLINWNDEEPIFEEDQLVVEFKETVPKDYHV 539

AY198374.1 DVPFQNIKLKVIATDRDNTNFTGVAEVNVNLINWNDEEPIFEEDQLVVKFKETVPKDYHV 540

KMP-4_20 (*r8B*) GRLRAHDRDIGDSVVHSILGNANTFLRIDEETGDIYVAIDDAFDYHRQNEFNILTSSRSG 600

KMP-4_16 (*r8A*) GRLRAHDRDIGDSVVHSILGNANTFLRIDEETGDIYVAIDDAFDYHRQNEFNILTSSRSG 600

KMP-4_3 (*r8A*) GRLRAHDRDIGDSVVHSILGNANTFLRIDEETGDIYVAIDDAFDYHRQNEFNILTSSRSG 600

KMP-4_26 (*r8A*) GRLRAHDRDIGDSVVHSILGNANTFLRIDEETGDIYVAIDDAFDYHRQNEFNILTSSRSG 600

KMP-4_8 (*r9A*) GRLRAHDRDIGDSVVHSILGNANTFLRIDEETGDIYVAIDDAFDYHRQNEFNIQVRAQDT 600

KMP-4_13 (*r9B*) GRLRAHDRDIGDSVVHSILGNANTFLRIDEETGDIYVAIDDAFDYHRQNEFNIQVRAQDT 600

KMP-5_2 (*r10A*) GRLRAHDRDIGDSVVHAILGNANTFLRIDEETGDIYVAIDDAFDYHRQNEFNIQVRAQDT 600

KMP-5_16 (*r10C*) GRLRAHDRDIGDSVVHSILGNANTFLRIDEETGDIYVAIDDAFDYHRQNEFNIQVRAQDT 600

KMP-5_18 (*r10A*) GRLRAHDRDIGDSVVHSILGNANTFLRIDEETGDIYVAIDDAFDYHRQNEFNIQVRAQDT 600

KMP-5_11 (*r10B*) GRLRAHDRDIGDSVVHSILGNANTFLRIDEETGDIYVAIDDAFDYHRQNEFNIQVRAQDT 600

KMP-6_7 (*r12A*) GRLRAHDRDIGDSVVHSILGNANTFLRIDEETGDIYVTIDDAFDYHRQNEFNIQVRAQDT 600

KMP-7_1 (*r12A*) GRLRAHDRDIGDSVVHSILGNANTFLRIDEETGDIYVTIDDAFDYHRQNEFNLQVRAQDT 600

KMP-7_2 (*r12B*) GRLRAHDRDIGDSVVFALRTPCRSQSP----------GIQRLLSWS-------------- 575

KMP-6_3 (*r11A*) GRLRAHDRDIGDSVVHSILGNANTFLRIDEETGDIYVTIDDAFDYHRQNEFNIQVRAQDT 600

KMP-6_5 (*r11B*) GRLRAHDRDIGDSVVHSILGNANTFLRIDEETGDIYVAIDDAFDYHRQNEFNIQVRAQDT 600

KMP-6_8 (*r11B*) GRLRAHDRDIGDSVVHSILGNANTFLRIDEETGDIYVAIDDAFDYHRQNEFNIQVRAQDT 600

KMP-7_3 (*r12C*) ------------------------------------------------------------ 29

KMP-7_4 (*r12D*) ------------------------------------------------------------ 29

KMP-8_3 (*s7*) GRLRAHDRDIGDSVVHSILGNANTFLRIDEETGDIYVAIDDAFDYHRQNEFNIQVRAQDT 599

KMP-8_24 (*s6A*) GRLRAHDRDIGDSVVHSILGNANTFLRIDEETGDIYVAIDDAFDYHRQNEFNIQVRAQDT 599

KMP-8_5 (*s6A*) GRLRAHDRDIGDSVVHSILGNANTFLRIDEETGDIYVAIDDAFDYHRQNEFNIQVRAQDT 599

KMP-8_46 (*s6A*) GRLRAHDRDIGDSVVHSILGNANTFLRIDEETGDIYVAIDDAFDYHRQNEFNIQVRAQDT 599

KMP-8_35 (*s6B*) GRLRAHDRDIGDSVVHSILGNANTFLRIDEETGDIYVAIDDACDYHRQNEFNIQVRAQDT 599

AY198374.1 GRLRAHDRDIGDSVVHSILGNANTFLRIDEETGDIYVAIDDAFDYHRQNEFNIQVRAQDT 600

KMP-4_20 (*r8B*) HHVG-------ARVQ-AYSDCSAGHRTRGRQQHTSYSEAASRKSVCRRECARGL------ 646

KMP-4_16 (*r8A*) HHVG-------ARVQ-AYSDCSAGHRTRGRQQHTSYSEADAWI----------------- 635

KMP-4_3 (*r8A*) HHVG-------ARVQ-AYSDCSAGHRTRGRQQHTSYSEADAWI----------------- 635

KMP-4_26 (*r8A*) YHVG-------ARVQ-AYSDCSAGHRTRGRQQHTSYSEADAWI----------------- 635

KMP-4_8 (*r9A*) MPEPESRHTATAQLVIELEDVNNTPPTL-RLP--RV------SPSVEENVPEGFEINREI 651

KMP-4_13 (*r9B*) MSEPESRHTATAQLVIELEDVNNTPPTL-RLP--RV------SPSVEENVPEGFEINREI 651

KMP-5_2 (*r10A*) MSEPESRHTATAQLVIELEDVNNTPPTL-RLP--RV------SPSVEENVPEGFEINREI 651

KMP-5_16 (*r10C*) MSEPESRHTATAQLVIELEDVNNTPPTL-RLP--RV------SPSVEENVPEGFEINREI 651

KMP-5_18 (*r10A*) MSEPESRHTATAQLVIELEDVNNTPPTL-RLP--RV------SPSVEENVPEGFEINREI 651

KMP-5_11 (*r10B*) MSEPESRHTATAQLVIELEDVNNTPPTL-RLP--RV------SPSVEENVPEGFEINREI 651

KMP-6_7 (*r12A*) MSEPESRHTATAQLVIELEDVNNTPPTL-RLP--RV------SPSVEENVPEGFEINREI 651

KMP-7_1 (*r12A*) MSEPESRHTATAQLVIELEDVNNTPPTL-RLP--RV------SPSVEENVPEGFEINREI 651

KMP-7_2 (*r12B*) ------------------------------------------------------------ 575

KMP-6_3 (*r11A*) MSEPESRHTATAQLVIELEDVNNTPPTL-RLP--RV------SPSVEENVPEGFEINREI 651

KMP-6_5 (*r11B*) MSEPESRHTATAQLVIELEDVNNTPPTL-RLP--RV------SPSVEENVPEGFEVNREI 651

KMP-6_8 (*r11B*) MSEPESRHTATAQLVIELEDVNNTPPTL-RLP--RV------SPSVEENVPEGFEVNREI 651

KMP-7_3 (*r12C*) ------------------------------------------------------------ 29

KMP-7_4 (*r12D*) ------------------------------------------------------------ 29

KMP-8_3 (*s7*) MSEPESRHTATAQLVIELEDVNNTPPTL-RLP--RV------SPSVEENGPEGFEVNREI 650

KMP-8_24 (*s6A*) MSEPESRHTATAQLVIELEDVNNTPPTL-RLP--RV------SPSVEENVPEGFEVNREI 650

KMP-8_5 (*s6A*) MSEPESRHTATAQLVIELGDVNNTPPTL-RLP--RV------SPSVEENVPEGFEVNREI 650

KMP-8_46 (*s6A*) MSEPESRHTATAQLVIELEDVNNTPPTL-RLP--RV------SPSVEENVPEGFEVNREI 650

KMP-8_35 (*s6B*) MSEPESRHTATAQLVIELEDVNNTPPTL-RLP--RV------SPSVEENVPEGFGVNREI 650

AY198374.1 MSEPESRHTATAQLVIELEDVNNTPPTL-RLP--RV------SPSVEENVPEGFEINREI 651

KMP-4_20 (*r8B*) ------------------------------------------------------------ 646

KMP-4_16 (*r8A*) ------------------------------------------------------------ 635

KMP-4_3 (*r8A*) ------------------------------------------------------------ 635

KMP-4_26 (*r8A*) ------------------------------------------------------------ 635

KMP-4_8 (*r9A*) TATDPGTTAYLQFEIDWDTSFATKQGRDTNPIEFHGCVDIETIFPNPADTREAVGRVVAK 711

KMP-4_13 (*r9B*) TATDPDTTAYLQFEIDWDTSFATKQGRDTNPIEFHGCVDIETIFPNPADTREAVGRVVAK 711

KMP-5_2 (*r10A*) TATDPDTTAYLQFEIDWDTSFATKQGRDTNPIEFHGCVDIETIFPNPADTREAVGRVVAK 711

KMP-5_16 (*r10C*) TATDPDTTAYLQFEIDWDTSFATKQGRDTNPIEFHGCVDIETIFPNPADTREAVGRVVAK 711

KMP-5_18 (*r10A*) TATDPDTTAYLQFEIDWDTSFATKQGRDTNPIEFHGCVDIETIFPNPADTREAVGRVVAK 711

KMP-5_11 (*r10B*) TATDPDTTAYLQFEIDWDTSFATKQGRDTNPIEFHGCVDIETIFPNPADTREAVGRVVAK 711

KMP-6_7 (*r12A*) TATDPDTTAYLQFEIDWDTSFATKQGRDTNPVEFHGCVDIETIFPNPADTREAVGRVVAK 711

KMP-7_1 (*r12A*) TAMDPDTTAYLQFEIDWDTSFATKQGRDTNPVEFHGCVDIETIFPSPADTREAVGRVVAK 711

KMP-7_2 (*r12B*) ------------------------------------------------------------ 575

KMP-6_3 (*r11A*) TATDPDTTAYLQFEIDWDTSFATKQGRVTNPVEFHGCVDIETIFPNPADTREAVGRVVAK 711

KMP-6_5 (*r11B*) TATDPDTTAYLQFEIDWDTSFATKQGRDTNPIEFHGCVDIETIFPNPADTREAVGRVVAK 711

KMP-6_8 (*r11B*) TATDPDTTAYLQFEIDWDTSFATKQGRDTNPIEFHGCVDIETIFPNPADTREAVGRVVAK 711

KMP-7_3 (*r12C*) ------------------------------------------------------------ 29

KMP-7_4 (*r12D*) ------------------------------------------------------------ 29

KMP-8_3 (*s7*) TATDPDTTAYLQFEIDWDTSFATKQGRDTNPIEFHGCVDIETIFPNPADTREAVGRVVAK 710

KMP-8_24 (*s6A*) TATDPDTTAYLQFEIDWDTSFATKQGRDTNPIEFHGCVDIETIFPNPADIREAVGRVVAK 710

KMP-8_5 (*s6A*) TATDPDTTAYLQFEIDWDTSFATKQGRDTNPIEFHGCVDIETIFPNPADTREAVGRVVAK 710

KMP-8_46 (*s6A*) TATDPDTTAYLQFEIDWDTSFATKQGRDTNPIEFHGCVDIETIFPNPADTREAVGRVVAK 710

KMP-8_35 (*s6B*) TATDPDTTAYLQFEIDWDTSFATKQGRDTNPIEFHGCVDIETIFPNPADTREAVGRVVAK 710

AY198374.1 TATDPDTTAYLQFEIDWDTSFATKQGRDTNPIEFHGCVDIETIFPNPADTREAVGRVVAK 711

KMP-4_20 (*r8B*) ------------------------------------------------------------ 646

KMP-4_16 (*r8A*) ------------------------------------------------------------ 635

KMP-4_3 (*r8A*) ------------------------------------------------------------ 635

KMP-4_26 (*r8A*) ------------------------------------------------------------ 635

KMP-4_8 (*r9A*) EIRHNVTIDFEEFEFLYLTVRVRDLHTDDGRDYDESTFTIIIIDMNDNWPI--------- 762

KMP-4_13 (*r9B*) EIRHNVTIDFEEFEFLYLTVRVRDLHTDDGRDYDESTFTIIIIDMNDNWPIWASGFLNQT 771

KMP-5_2 (*r10A*) EIRHNVAIDFEEFEFLYLTVRVRDLHTDDGRDYDESTFTIIIIDMNDNWPIWASGFLNQT 771

KMP-5_16 (*r10C*) EIRHNVTIDFEEFEFLYLTVRVRDLHTDDGRDYDESTFTIIIIDMNDNWPIWASGFLNQT 771

KMP-5_18 (*r10A*) EIRHNVTIDFEEFEFLYLTVRVRDLHTDDGRDYDESTFTIIIIDMNDNWPIWASGFLNQT 771

KMP-5_11 (*r10B*) EIRHNVTIDFEEFEFLYLTVRVRDLHTDDGRDYDESTFTIIIIDMNDNWPIWASGFLNQT 771

KMP-6_7 (*r12A*) EIRHNVTIDFEEFEFLYLTVRVRDLHTEDGRDYDESTFTIIIIDMNDNWPIWASGFLNQT 771

KMP-7_1 (*r12A*) EIRHNVTIDFEEFEFLYLTVRVRDLHTEDGRDYDEPTFTIIIIDMNDNWPIWASGFLNQT 771

KMP-7_2 (*r12B*) ------------------------------------------------------------ 575

KMP-6_3 (*r11A*) EIRHNVTIDFEEFEFLYLTVRVRDLHTEDGRDYDESTFTIIIIDMNDNWPIWASGFLNQT 771

KMP-6_5 (*r11B*) EIRHNVTIDFEEFEFLYLTVRVRDLHTDDGRDYDESTFTIIIIDMNDNWPIWASGFLNQT 771

KMP-6_8 (*r11B*) EIRHNVTIDFEEFEFLYLTVRVRDLHTDDGRDYDESTFTIIIIDMNDNWPIWASGFLNQT 771

KMP-7_3 (*r12C*) ------------------------------------------------------------ 29

KMP-7_4 (*r12D*) ------------------------------------------------------------ 29

KMP-8_3 (*s7*) EIRHNVTIDFEEFEFLYLTVRVRDLHTDDGRDYDESTFTIIIIDMNDNWPIWASGFLNQT 770

KMP-8_24 (*s6A*) EIRHNVTIDFEEFEFLYLTVRVRDLHTDDGRDYDESTFTIIIIDMNDNWPIWASGFLNQT 770

KMP-8_5 (*s6A*) EIRHNVTIDFEEFEFLYLTVRVRDLHTDDGRDYDESTFTIIIIDMNDNWPIWASGFLNQT 770

KMP-8_46 (*s6A*) EIRHNVTIDFEEFEFLYLTVRVRDLHTDDGRDYDESTFTIIIIDMNDNWPIWASGFLNQT 770

KMP-8_35 (*s6B*) EIRHNVTIDFEEFEFLYLTVRVRDLHTDDGRDYDESTFTIIIIDMNDNWPIWASGFLNQT 770

AY198374.1 EIRHNVTIDFEEFEFLYLTVRVRDLHTDDGRDYDESTFTIIIIDMNDNWPIWASGFLNQT 771

KMP-4_20 (*r8B*) ------------------------------------------------------------ 646

KMP-4_16 (*r8A*) ------------------------------------------------------------ 635

KMP-4_3 (*r8A*) ------------------------------------------------------------ 635

KMP-4_26 (*r8A*) ------------------------------------------------------------ 635

KMP-4_8 (*r9A*) ------------------------------------------------------------ 762

KMP-4_13 (*r9B*) FSIRERSSTGVVIGSVLATDIDGPLYNQVRYTIIPQEDTPEGLVQIHFVTGQITVDENGA 831

KMP-5_2 (*r10A*) FSIRERSSTGVVIGSVLATDIDGPLYNQVRYTIIPQEDTPEGLVQIHFVTGQITVDENGA 831

KMP-5_16 (*r10C*) FSIRERSSTGVVIGSVLATDIDGPLYNQVRYTIIPQEDTPEGLVQIHFVTGQITVDENGA 831

KMP-5_18 (*r10A*) FSIRERSSTGVVIGSILATDIDGPLYNQVRYTIIPLEDTPEGLVQIHFVTGQITVDENGA 831

KMP-5_11 (*r10B*) FSIRERSSTGVVIGSVLATDIDGPLYNQVRYTIIPQEDTPEGLVQIHFVTGQITVDENGA 831

KMP-6_7 (*r12A*) FSIRERSSTGVVIGSVLATDIDGPLYNQVRYTIIPQEDTPEGLVQIHFVTGQITVDENGA 831

KMP-7_1 (*r12A*) FCIRERSSTGVVIGSVLATDIDGPLYNQVRYTIIPQEDTPEGLVQIHFVTGQITVDENGA 831

KMP-7_2 (*r12B*) ------------------------------------------------------------ 575

KMP-6_3 (*r11A*) FSIRERSSTGVVIGSVLATDIDGPLYNQVRYTIIPQEDTPEGLVQIHFVTGQITVDENGA 831

KMP-6_5 (*r11B*) FSIRERSSTGVVIGSVLATDIDGPLYNQVRYTIIPQEDTPEGLVQIHFVTGQITVDENGA 831

KMP-6_8 (*r11B*) FSIRERSSTGVVIGSVLATDIDGPLYNQVRYTIIPQEDTPEGLVQIHFVTGQITVDENGA 831

KMP-7_3 (*r12C*) ------------------------------------------------------------ 29

KMP-7_4 (*r12D*) ------------------------------------------------------------ 29

KMP-8_3 (*s7*) FSIRERSSTGVVIGSVLATDIDGPLYNQVRYTIIPQEDTPEGLVQIHFVTGQITVDENGA 830

KMP-8_24 (*s6A*) FSIRERSSTGVVIGSVLATDIDGPLYNQVRYTIIPQEDTPEGLVQIHFVTGQITVDENGA 830

KMP-8_5 (*s6A*) FSIRERSSTGVVIGSVLATDIDGPLYNQVRYTIIPQEDTPEGLVQIHFVTGQITVDENGA 830

KMP-8_46 (*s6A*) FSIRERSSTGVVIGSVLATDIDGPLYNQVRYTIIPQEDTPEGLVQIHFVTGQITVDENGA 830

KMP-8_35 (*s6B*) FSIRERSSTGVVIGSVLATDIDGPLCNQVRYTIIPQEDTPEGLVQIHFVTGQITVDENGA 830

AY198374.1 FSIRERSSTGVVIGSVLATDIDGPLYNQVRYTIIPQEDTPEGLVQIHFVTGQITVDENGA 831

KMP-4_20 (*r8B*) ------------------------------------------------------------ 646

KMP-4_16 (*r8A*) ------------------------------------------------------------ 635

KMP-4_3 (*r8A*) ------------------------------------------------------------ 635

KMP-4_26 (*r8A*) ------------------------------------------------------------ 635

KMP-4_8 (*r9A*) ------------------------------------------------------------ 762

KMP-4_13 (*r9B*) IDADIPPRWHLNYTVIASDKCSEENEENCPPDPVFWDTLRDNVINIVDINNKVPAADLSR 891

KMP-5_2 (*r10A*) IDADIPPRWHLNYTVIASDKCSEENEENCPPDPVFWDTLGDNVINIVDINNKVPAADLSR 891

KMP-5_16 (*r10C*) IDADIPPRWHLNYTVIASDKCSEENEENCPPDPVFWDTLGDNVINIVDINNKVPAADLSR 891

KMP-5_18 (*r10A*) IDADIPPRWHLNYTVIASDKCSEENEENCPPDPVFWDTLGDNVINIVDINNKVPAADLSR 891

KMP-5_11 (*r10B*) IDADIPPRWHLNYTVIASDKCSEENEENCPPDPVFWDTLGDNVINIVDINNKVPAADLSR 891

KMP-6_7 (*r12A*) IDADIPPRWHLNYTVIASDKCSEENEENCPPDPVFWDTLGDNVINIVDINNKVPAADLSR 891

KMP-7_1 (*r12A*) IDADIPPRWHLNYTVIASDKCSEENEENCPPDPVFWDTLGDNVINIVDINNKVPAVDLSR 891

KMP-7_2 (*r12B*) ------------------------------------------------------------ 575

KMP-6_3 (*r11A*) IDADIPPRWHLNYTVIASDKCSEENEENCPPDPVFWDTLGDNVINIVDINNKVPAADLSR 891

KMP-6_5 (*r11B*) IDADIPPRWHLNYTVIASDKCSEENEENCPPDPVFWDTLRDNVINIVDINNKVPAADLSR 891

KMP-6_8 (*r11B*) IDADIPPRWHLNYTVIASDKCSEENEENCPPDPVFWDTLRDNVINIVDINNKVPAADLSR 891

KMP-7_3 (*r12C*) ------------------------------------------------------------ 29

KMP-7_4 (*r12D*) ------------------------------------------------------------ 29

KMP-8_3 (*s7*) IDADIPPRWHLNYTIIASDKCSEENEENCPPDPVFWDTLGDNVINIVDINNKVPAADLSR 890

KMP-8_24 (*s6A*) IDADIPPRWHLNYTIIASDKCSEENEENCPPDPVFWDTLGDNVINIVDINNKVPAAGLSR 890

KMP-8_5 (*s6A*) IDADIPPRWHLNYTIIASDKCSEENEENCPPDPVFWDTLGDNVINIVDINNKVPAADLSR 890

KMP-8_46 (*s6A*) IDADIPPRWHLNYTIIASDKCSEENEENCPPDPVFWDTLGDNVINIVDINNKVPAADLSR 890

KMP-8_35 (*s6B*) IDADIPPRWHLNYTIIASDKCSEENEENCPPDPVFWDTLGDNVINIVDINDKVPAADLSR 890

AY198374.1 IDADIPPRWHLNYTVIASDKCSEENEENCPPDPVFWDTLGDNVINIVDINNKVPAADLSR 891

KMP-4_20 (*r8B*) ------------------------------------------------------------ 646

KMP-4_16 (*r8A*) ------------------------------------------------------------ 635

KMP-4_3 (*r8A*) ------------------------------------------------------------ 635

KMP-4_26 (*r8A*) ------------------------------------------------------------ 635

KMP-4_8 (*r9A*) ------------------------------------------------------------ 762

KMP-4_13 (*r9B*) FNETVYIYENAPDFTNVVKIYSIDEDRDEIYHTVRYQINYAVNQRLRDFFAIDLDSGQVY 951

KMP-5_2 (*r10A*) FNETVYIYENAPDFTNVVKIYSIDEDRDEIYHTVRYQINYAVNQRLRDFFAIDLDSGQVY 951

KMP-5_16 (*r10C*) FNETVYIYENAPDFTNVVKIYSIDEDRDEIYHTVRYQINYAVNQRLRDFFAIDLDSGQVY 951

KMP-5_18 (*r10A*) FNETVYIYENAPDFTNVVKIYSIDEDRDEIYHTVRYQINYAVNQRLRDFFAIDLDSGQVY 951

KMP-5_11 (*r10B*) FNETVYIYENAPDFTNVVKIYSIDEDRDEIYHTVRYQINYAVNQRLRDFLAIDLDSGQVY 951

KMP-6_7 (*r12A*) FNETVYIYENAPDFTNVVKMYSIDEDRDEIYHAVRYQINYAVNQRLRDFFAIDLDSGQVY 951

KMP-7_1 (*r12A*) FNETVYIYENAPDFTNVVKIYSIDEDRDEMYHTVRYQINYAVNQRLRDFFAIDLDSGQVY 951

KMP-7_2 (*r12B*) ------------------------------------------------------------ 575

KMP-6_3 (*r11A*) FNETVYIYENAPDFTNVVKIYSIDEDRDEIYHTVRYRINYAVNQRLRDFFAIDLDSGQVY 951

KMP-6_5 (*r11B*) FNETVYIYENAPDFTNVVKIYSIDEDRDEIYHTVRYQINYAVNQRLRDFFAIDLDSGQVY 951

KMP-6_8 (*r11B*) FNETVYIYENAPDFTNVVKIYSIDEDRDEIYHTVRYQTNYAVNQRLRDFFAIDLDSGQVY 951

KMP-7_3 (*r12C*) ------------------------------------------------------------ 29

KMP-7_4 (*r12D*) ------------------------------------------------------------ 29

KMP-8_3 (*s7*) FNETVYIYENAPDFTNVVKIYSIDEDRDEIYHTVRYQINYAVNQRLRDFLAIDLDSGQVY 950

KMP-8_24 (*s6A*) FNETVYIYENAPDFTNVVKIYSIDEDRDEIYHTVRYQINYAVNQRLRDFFAIDLDSGQVY 950

KMP-8_5 (*s6A*) FNETVYIYENAPDFTNVVKIYSIDEDRDEIYHTVRYQINYAVNQRLRDFFAIDLDSGQVY 950

KMP-8_46 (*s6A*) FNETVYVYENAPDFTNVVKIYSIDEDRDEIYHTVRYQINYAVNQRLRDFFAIDLDSGQVY 950

KMP-8_35 (*s6B*) FNETVYIYENAPDFTNVVKIYSIDEDRDEIYHTVRYQINHAVNQRLRDFFAIDLDSGQVY 950

AY198374.1 FNETVYIYENAPDFTNVVKIYSIDEDRDEIYHTVRYQINYAVNQRLRDFFAIDLDSGQVY 951

KMP-4_20 (*r8B*) ------------------------------------------------------------ 646

KMP-4_16 (*r8A*) ------------------------------------------------------------ 635

KMP-4_3 (*r8A*) ------------------------------------------------------------ 635

KMP-4_26 (*r8A*) ------------------------------------------------------------ 635

KMP-4_8 (*r9A*) ------------------------------------------------------------ 762

KMP-4_13 (*r9B*) VENTNNELLDRDRGEDQHRIFINLIDNFYSEGDGNRNVNTTEVLVILLDENDNAPELPTP 1011

KMP-5_2 (*r10A*) VENTNNELLDRDRGEDQHRIFINLIDNFYSEGDGNRNVNTTEVLVILLDENDNAPELPTP 1011

KMP-5_16 (*r10C*) VENTNNELLDRDRGEDQHRIFINLIDNFYSEGDGNRNVNTTEVLVILLDENDNAPELPTP 1011

KMP-5_18 (*r10A*) VENTNNELLDRDRGEDQHRIFINLIDNFYSEGDGNRNVNTTEVLVVLLDENDNAPELPTP 1011

KMP-5_11 (*r10B*) VENTNNELLDRDRGEDQHRIFINLIDNFHSEGDGNRNVNTTEVLVILLDENDNAPELPTP 1011

KMP-6_7 (*r12A*) VENTNNELLDRDRGEDQHRIFINLIDNFYSEGDGNRNVNTTEVLVILLDENDNAPELPTP 1011

KMP-7_1 (*r12A*) VENTNNELLDRDRGEDQHRIFINLIDNFYSEGDGNRNVNTTEVLVVLLDENDNAPELPTP 1011

KMP-7_2 (*r12B*) ------------------------------------------------------------ 575

KMP-6_3 (*r11A*) VENTNNELLDRDRGEDQHRIFINLIDNFYSEGDGNRNVNTTEVLVILLDENDNAPELPTP 1011

KMP-6_5 (*r11B*) VENTNNELLDRDRGEDQHRIFINLIDNFYSEGDGNRNVNTTEVLVILLDENDNAPELPTP 1011

KMP-6_8 (*r11B*) VENTNNELLDRDRGEDQHRIFINLIDNLYSEGDGNRNVNTTEVLVILLDENDNAPELPTP 1011

KMP-7_3 (*r12C*) ------------------------------------------------------------ 29

KMP-7_4 (*r12D*) ------------------------------------------------------------ 29

KMP-8_3 (*s7*) VENTNNELLDRDRGEDQHRIFINLIDNFYSEGDGNRNVNTTEVLVILLDENDNAPELPTP 1010

KMP-8_24 (*s6A*) VENTNNELLDRDRGEDQHRIFINLIDNFYSEGDGNRNVNTTEVLVILLDENDNAPELPTP 1010

KMP-8_5 (*s6A*) VENTNNELLDRDRGEDQHRIFINLIDNFYSEGDGNRNVNTTEVLVILLDENDNAPELPTP 1010

KMP-8_46 (*s6A*) VENTNNELLDRDRGEDQHRIFINLIDNFYSEGDGNRNVNTTEVLVILLDENDNAPELPTP 1010

KMP-8_35 (*s6B*) VENTNNELLDRDRGEDQHRIFINLIDNFYSEGDGNRNVNTTEVLVILLDENDNAPELPTP 1010

AY198374.1 VENTNNELLDRDRGEDQHRIFINLIDNFYSEGDGNRNVNTTEVLVILLDENDNAPELPTP 1011

KMP-4_20 (*r8B*) ------------------------------------------------------------ 646

KMP-4_16 (*r8A*) ------------------------------------------------------------ 635

KMP-4_3 (*r8A*) ------------------------------------------------------------ 635

KMP-4_26 (*r8A*) ------------------------------------------------------------ 635

KMP-4_8 (*r9A*) ------------------------------------------------------------ 762

KMP-4_13 (*r9B*) EELSWSISENLQEGITLDGESDVIYAPDIDEEDTPNSHVGYAILAMTVTNRDLDTVPRLL 1071

KMP-5_2 (*r10A*) EELSWSISENLQEGITLDGERDVIYAPDIDEEDTPNSHVGYAILAMTVTNRDLGTVPRLL 1071

KMP-5_16 (*r10C*) EELSWSISENLQEGITLDGERDVIYAPDIDEEDTPNSHVGYAILAMTVTNRDLDTVPRLL 1071

KMP-5_18 (*r10A*) EELSWSISENLQEGITLDGERDVIYAPDIDEEDTPNSHVGYAILAMTVTNRDLDTVPRLL 1071

KMP-5_11 (*r10B*) EELSWSISENLQEGITLDGERDVIYAPDIDEEDTPNSHVGYAILAMTVTNRDLDTVPRLL 1071

KMP-6_7 (*r12A*) EELSWSISENLQEGITLDGESDVIYAPDIDEEDTPNSHVGYAILAMTVTNRDLDTVPRLL 1071

KMP-7_1 (*r12A*) EELSWSISENLQEGITLDGESDVIYAPDIYEEDTPNSHVGYAILAMTVTNRDLDTVPRLL 1071

KMP-7_2 (*r12B*) ------------------------------------------------------------ 575

KMP-6_3 (*r11A*) EELSWSISENLQEGITLDGESDVIYAPDIDEEDTPNSHVGYAILAMTVTNRDLDTVPRLL 1071

KMP-6_5 (*r11B*) EELSWSISENLQEGITLDGESDVIYAPDIDEEDTPNSHVGYAILAMTVTNRDLDTVPRLL 1071

KMP-6_8 (*r11B*) EELSWSISENLQEGITLDGESDVIYAPDIDEEDTPNSHVGYAILAMTVTNRDLDTVPRLL 1071

KMP-7_3 (*r12C*) ------------------------------------------------------------ 29

KMP-7_4 (*r12D*) ------------------------------------------------------------ 29

KMP-8_3 (*s7*) EELSWSISENLQEGITLDGESDVIYAPDIDEEDTPNSHVGYAILAMTVTNRDLDTVPRLL 1070

KMP-8_24 (*s6A*) EELSWSISENLQEGITLDGERDVIYAPDIDEEDTPNSHVGYAILAITVTNRDLDTVPRLL 1070

KMP-8_5 (*s6A*) EELSWSISENLQEGITLDGERDVIYAPDIDEEDTPNSHVGYATLAITVTNRDLDTVPRLP 1070

KMP-8_46 (*s6A*) EELSWSISENLQEGITLDGERDVIYAPDIDEEDTPNSHVGYAILAITVTNRDLDTVPRLL 1070

KMP-8_35 (*s6B*) EELSWSTSENLQEGITLDGERDVIYAPDIDEEDTPNSHVGYAILAITVTNRDLDTAPRLL 1070

AY198374.1 EELSWSISENLQEGITLDGESDVIYAPDIDEEDTPNSHVGYAILAMTVTNRDLDTVPRLL 1071

KMP-4_20 (*r8B*) ------------------------------------------------------------ 646

KMP-4_16 (*r8A*) ------------------------------------------------------------ 635

KMP-4_3 (*r8A*) ------------------------------------------------------------ 635

KMP-4_26 (*r8A*) ------------------------------------------------------------ 635

KMP-4_8 (*r9A*) ------------------------------------------------------------ 762

KMP-4_13 (*r9B*) NMLSPNNVTGFLQTAMPLRGYWGTYDISVLAFDHGIPQQISHEVYELEIRPYNYNPPQFV 1131

KMP-5_2 (*r10A*) NMLSPNNVTGFLQTAMPLRGYWGTYDISIP------------------------------ 1101

KMP-5_16 (*r10C*) NMLSPNNVTGFLQTAMPLRGYWGTYDISIL------------------------------ 1101

KMP-5_18 (*r10A*) NMLSPNNVTGFLQTAMPLRGYWGTYDISIL------------------------------ 1101

KMP-5_11 (*r10B*) NMLSPNNVTGFLQTAMPLRGYWGTYDISIL------------------------------ 1101

KMP-6_7 (*r12A*) NMLSPNNVTGFLQTAMPLRGYWGTYDISILAFDHGIPQQISHEVYELEIRPYNYNPPQFV 1131

KMP-7_1 (*r12A*) NMLSPNNVTGFLQTAMPLRGYWGTYDISILAFDHGIPQQISHEVYELEIRPYNYNPPQFV 1131

KMP-7_2 (*r12B*) ------------------------------------------------------------ 575

KMP-6_3 (*r11A*) NMLSPNNVTGFLQTAMPLRGYWGVRPRYSSADIS-------------------------- 1105

KMP-6_5 (*r11B*) NMLSPNNVTGFLQTAMPLRGYWGTYDISVLAFDHGIPQQISHEVCELEIRPYNYNPPQFV 1131

KMP-6_8 (*r11B*) NMLSPNNVTGFLQTAMPLRGYWGTYDISVLAFDHGIPQQISHEVYGLEIRPYNYNPPQFV 1131

KMP-7_3 (*r12C*) ------------------------------------------------------------ 29

KMP-7_4 (*r12D*) ------------------------------------------------------------ 29

KMP-8_3 (*s7*) NMLSPNNVTGFLQTAMPLRGYWDTYDISILAFDHGIPQQISHEVYELEIRPYNYNPPQFV 1130

KMP-8_24 (*s6A*) NMLSPNNVTGFLQTAMPLRGYWGTYDISILAFDHGIPQQISHEVYELEIRPYNYNPPQFV 1130

KMP-8_5 (*s6A*) NMLSPNNVTGFLQTAMPLRGYWGTYDISILAFDHGIPQQISHEVYELEIRPYNYNPPQFV 1130

KMP-8_46 (*s6A*) NMLSPNNVTGFLQTAMPLRGYWGTYDISILAFDHGIPQQISHEVYELEIRPYNYNPPQFV 1130

KMP-8_35 (*s6B*) NMLSPNNVTGFLQTAMPLRGYWGTYDISILAFDHGIPQQISHEVYELEIRPYNYNPPQFV 1130

AY198374.1 NMLSPNNVTGFLQTAMPLRGYWGTYDISILAFDHGIPQQISHEVYELEIRPYNYNPPQFV 1131

KMP-4_20 (*r8B*) ------------------------------------------------------------ 646

KMP-4_16 (*r8A*) ------------------------------------------------------------ 635

KMP-4_3 (*r8A*) ------------------------------------------------------------ 635

KMP-4_26 (*r8A*) ------------------------------------------------------------ 635

KMP-4_8 (*r9A*) ------------------------------------------------------------ 762

KMP-4_13 (*r9B*) FPESGTILRLALERAVVNNVLSLVNGDLLDRIQAIDDDGPDAGVVTFDIVGDADASNYFR 1191

KMP-5_2 (*r10A*) ------------ERAVVNNVLSLVNGDPLDRIQAIDDDGLDAGVVTFDIVGDADASNYFR 1149

KMP-5_16 (*r10C*) ------------ERAVVNNVLSLVNGDPLDRIQVIDDDGLDAGVVTFDIVGDADASNYFR 1149

KMP-5_18 (*r10A*) ------------ERAVVNNVLSLVNGDPLDRIQAIDDDGLDAGVVTFDIVGDADASNSFR 1149

KMP-5_11 (*r10B*) ------------ERAVVNNVSSLVNGDPLDRIQAIDDDGLDAGVVTFDIVGDADASNYFR 1149

KMP-6_7 (*r12A*) FPESGTILRLALERAVVNNVLSLVNGDPLDRIQAIDDDGLDAGVVTFDIVGDADASNYFR 1191

KMP-7_1 (*r12A*) FPESGTILRLALERAVVNNVLSLVNGDPLDRIQAIDDDGLDAGVVTFDIVGDADASNYFR 1191

KMP-7_2 (*r12B*) ------------------------------------------------------------ 575

KMP-6_3 (*r11A*) ------------------------------------------------------------ 1105

KMP-6_5 (*r11B*) FPESGTILRLALERAVVNNVLSLVNGDLLDRIQAIDDDGLDAGVVAFDIVGDADASNYFR 1191

KMP-6_8 (*r11B*) FLESGPILRLALERAVVNNVLSLVNGDLLDRIQAIDDDGLDAGVVTFDIVGDADASNYFR 1191

KMP-7_3 (*r12C*) ------------------------------------------------------------ 29

KMP-7_4 (*r12D*) ------------------------------------------------------------ 29

KMP-8_3 (*s7*) FPESGTILRLALERAVVNNVLSLVNGDPLDRIQAIDDDGLDAGVVTFDIVGDADASNYFR 1190

KMP-8_24 (*s6A*) FPESGTILRLALERAVVNNVLSLVNGDPLDRLQAIDDDGLDAGVVTFDIVGGADASNYFR 1190

KMP-8_5 (*s6A*) FPESGAILRLALERAVVNNVLSLVNGDPLDRLQAIDDDGLDAGVVTFDIVGDADASNYFR 1190

KMP-8_46 (*s6A*) FPESGTILRLALERAVVNNVLSLVNGDPLDRLQAIDDDGLDAGVVTFDIVGDADASNYFR 1190

KMP-8_35 (*s6B*) FPESGTILRLALERAVVNNVLSLVNGDPLDRLQAIDDDGLDAGVVTFDIVGDADASNYFR 1190

AY198374.1 FPESGTILRLALERAVVNNVLSLVNGDPLDRIQAIDDDGLDAGVVTFDIVGDADASNYFR 1191

KMP-4_20 (*r8B*) ------------------------------------------------------------ 646

KMP-4_16 (*r8A*) ------------------------------------------------------------ 635

KMP-4_3 (*r8A*) ------------------------------------------------------------ 635

KMP-4_26 (*r8A*) ------------------------------------------------------------ 635

KMP-4_8 (*r9A*) ------------------------------------------------------------ 762

KMP-4_13 (*r9B*) VNNDGDNFGTLLLTQALPVPYFAHHI---------------------------------- 1217

KMP-5_2 (*r10A*) VNNDGDNFGTLLLTQALPEEGKEFEVTIRATDGGTEPRSYSTDSTITVLFVPTLGDPIFQ 1209

KMP-5_16 (*r10C*) VNNDGDNFGTLLLTQALPEEGKEFEVTIRATDGGTEPRSYSTDSTITVLFVPTLGDPIFQ 1209

KMP-5_18 (*r10A*) VNNDGDNFGTLLLTQALPEEGKEFEVTIRATDGGTEPRSYSTDSTITVLFVPTLGDPIFQ 1209

KMP-5_11 (*r10B*) VNNDGDNFGTLLLTQALPEVGKEFEVTIRATDGGTEPRSYSTDSTITVLFVPTLGDPIFQ 1209

KMP-6_7 (*r12A*) VNNDGDNFGTLLLTQALPEEGKEFEVTIRATDGGTEPRSYSTDSTITVLFVPTLGDPIFQ 1251

KMP-7_1 (*r12A*) VNNDGDNFGTLLLTQALPEEGKEFEVTIRATDGGTEPRSYSTDSTITVLFVPTLGDPIFQ 1251

KMP-7_2 (*r12B*) ------------------------------------------------------------ 575

KMP-6_3 (*r11A*) ------------------------------------------------------------ 1105

KMP-6_5 (*r11B*) VNNDGDNFGTLLLTQALPEEGKEFEVTIRATDGGTEPRSYSTDSTITVLFVPTLGNPIFQ 1251

KMP-6_8 (*r11B*) VNNDGDNFGTLLLTQALPEEGKEFEVTIRATDGGTEPRSYSTDSTITVLFVPTLGDPIFQ 1251

KMP-7_3 (*r12C*) ------------------------------------------------------------ 29

KMP-7_4 (*r12D*) ------------------------------------------------------------ 29

KMP-8_3 (*s7*) VNNDGDNFGTLLLTQALPEEGKEFEVTIRATDGGTEPRSYSTDSTITVLFVPTLGDPIFQ 1250

KMP-8_24 (*s6A*) VNNDGDNFGTLLLTQALPEEGKEFEVTIRATDGGIEPRSYSTDSTITVLFVPTLGDPIFQ 1250

KMP-8_5 (*s6A*) VNNDGDNFGTLLLTQALPEEGKEFEVTIRATDGGIEPRSYSTDSTITVLFVPTLGDPIFQ 1250

KMP-8_46 (*s6A*) VNNDGDNFGTLLLTQALPEEGKEFEVTIRATDGGIEPRSYSTDSTITVLFVPTLGDPIFQ 1250

KMP-8_35 (*s6B*) VNNDGDNFGTLLLTQALPEEGKEFGVTIRATDGGIEPRSYSTDSTITVLFVPTLGDPIFQ 1250

AY198374.1 VNNDGDNFGTLLLTQALPEEGKEFEVTIRATDGGTEPRSYSTDSTITVLFVPTLGDPIFQ 1251

KMP-4_20 (*r8B*) ------------------------------------------------------------ 646

KMP-4_16 (*r8A*) ------------------------------------------------------------ 635

KMP-4_3 (*r8A*) ------------------------------------------------------------ 635

KMP-4_26 (*r8A*) ------------------------------------------------------------ 635

KMP-4_8 (*r9A*) ------------------------------------------------------------ 762

KMP-4_13 (*r9B*) ------------------------------------------------------------ 1217

KMP-5_2 (*r10A*) DNTYSVAF-----------------------------------FGGVDYEPFDLDPVTNV 1234

KMP-5_16 (*r10C*) DNTYSVAF-----------------------------------FGGVDYEPFDLDPVTNV 1234

KMP-5_18 (*r10A*) DNTYSVAF-----------------------------------FGGVDYEPFDLDPVTNV 1234

KMP-5_11 (*r10B*) DNTYSVAF-----------------------------------FGGVDYEPFDLDPVTNV 1234

KMP-6_7 (*r12A*) DNTYSVAFFEKEVGLTERFSLPHAEDPKNKLCTDDCHDIYYRIFGGVDYEPFDLDPVTNV 1311

KMP-7_1 (*r12A*) DNTYSVAFFEKEVGLTERFSLPHAEDPKNKLCTDDCHDIYYRIFGGVDYEPFDLDPVTNV 1311

KMP-7_2 (*r12B*) ------------------------------------------------------------ 575

KMP-6_3 (*r11A*) ------------------------------------------------------------ 1105

KMP-6_5 (*r11B*) DNTYSVAFFEKEVGLTERFSLPHAEDPKNKLCTDDCHDIYYRIFGGVDYEPFDLDPVTNV 1311

KMP-6_8 (*r11B*) DNTYSVAFFEKEVGLTERFSLPHAEDPKNKLCTDDCHDIYYRIFGGVDYEPFDLDPVTNV 1311

KMP-7_3 (*r12C*) ------------------------------------------------------------ 29

KMP-7_4 (*r12D*) ------------------------------------------------------------ 29

KMP-8_3 (*s7*) DNTYSVAFFEKEVGLTERFSLPHAGDPKNKLCTDDCHDIYYRIFGGVDYEPFDLDPVTNV 1310

KMP-8_24 (*s6A*) DNTYSVAFFEKEVGLTERFSLPHAEGPKNKLCTDDCHDIYYRIFGGVDYEPFDLDPVTNV 1310

KMP-8_5 (*s6A*) DNTYSVAFFEKEVGLTERFSLPHAEDPRNKLCTDDCHDIYYRIFGGVDYEPFDLDPVTNV 1310

KMP-8_46 (*s6A*) DNTYSVAFFEKEVGLTERFSLPHAEDPKNKLCTDDCHDIYYRIFGGVDYEPFDLDPVTNV 1310

KMP-8_35 (*s6B*) DNTYSVAFFEKEVGLTERFSLPHAEDPKNKLCTDDCHDIYYRIFGGVDYEPFDLDPVTNV 1310

AY198374.1 DNTYSVAFFEKEVGLTERFSLPHAEDPKNKLCTDDCHDIYYRIFGGVDYEPFDLDPVTNV 1311

KMP-4_20 (*r8B*) ------------------------------------------------------------ 646

KMP-4_16 (*r8A*) ------------------------------------------------------------ 635

KMP-4_3 (*r8A*) ------------------------------------------------------------ 635

KMP-4_26 (*r8A*) ------------------------------------------------------------ 635

KMP-4_8 (*r9A*) ------------------------------------------------------------ 762

KMP-4_13 (*r9B*) -------HRED-------------P---------------------SSEPP--------- 1227

KMP-5_2 (*r10A*) IFLKSELDRETTATHVVQVAASNSPTGGGIPLPGSLLTVTVTVREADPRPVFEQRLYTAG 1294

KMP-5_16 (*r10C*) IFLKSELDRETTATHVVQVAASNSPTGGGIPLPGSLLTVTVTVREADPRPVFEQRLYTAG 1294

KMP-5_18 (*r10A*) IFLKSELDRETTATHVVQVAASNSPTGGGMPLPGSLLTVTVTVREADPRPVFEQRLYTAG 1294

KMP-5_11 (*r10B*) IFLKSELDRETTATHVVQVAASNSPTGGGIPLPGSLLTVTVTVREADPRPVFEQRLYTAG 1294

KMP-6_7 (*r12A*) IFLKSELDRETTATHVVQVAASNSPTGGGIPLPGSLLTVTVTVREADPRPVFEQRLYTAG 1371

KMP-7_1 (*r12A*) IFLKSELDRETTATHVVQVAASNSPTGGGIPLPGSLLTVTVTVREADPRPVFEQRLYTAG 1371

KMP-7_2 (*r12B*) ------------------------------------------------------------ 575

KMP-6_3 (*r11A*) ------------------------------------------------------------ 1105

KMP-6_5 (*r11B*) IFLKSELDRETTATHVVQVAASNSPTGGGIPLPGSLLTVTVTVREADPRPVFEQRLYTAG 1371

KMP-6_8 (*r11B*) IFLKLELDRETTATHVVQVAASNSPTGGGIPLPGSLLTVTVTVREADPRPVFEQRLYTAG 1371

KMP-7_3 (*r12C*) ------------------------------------------------------------ 29

KMP-7_4 (*r12D*) ------------------------------------------------------------ 29

KMP-8_3 (*s7*) IFLKSELDRETTATHVVQVAASNSPTGGGIPLPGSLLTVTVTVREADPRPVFGQRLYTAG 1370

KMP-8_24 (*s6A*) IFLKSELDRETTATHVVQVAASNSPTGGGIPLPGSLLTVTVTAREADPRPVFEQRLYTAG 1370

KMP-8_5 (*s6A*) IFLKSELDRETTATHVVQVAASNSPTGGGIPLPGSLLTVTVTVREADPRPVFEQRLYTAG 1370

KMP-8_46 (*s6A*) IFLKSELDRETTATHVVQVAASNSPTGGGIPLPGSLLTVTVTVREADPRPVFEQRLYTAG 1370

KMP-8_35 (*s6B*) IFLKSELDRETTATHVVQVAASNSPTGGGIPLPGSLFTVTVTVREADPRPVFEQRLYTAG 1370

AY198374.1 IFLKSELDRETTATHVVQVAASNSPTGGGIPLPGSLLTVTVTVREADPRPVFEQRLYTAG 1371

KMP-4_20 (*r8B*) ------------------------------------------------------------ 646

KMP-4_16 (*r8A*) ------------------------------------------------------------ 635

KMP-4_3 (*r8A*) ------------------------------------------------------------ 635

KMP-4_26 (*r8A*) ------------------------------------------------------------ 635

KMP-4_8 (*r9A*) ------------------------------------------------------------ 762

KMP-4_13 (*r9B*) ------------------------------------------------------------ 1227

KMP-5_2 (*r10A*) ISTSDNINRELLTVRATHSENAQLTYTIEDGSMAVDSTLEAVKDSAFHLNAQTGVLILRI 1354

KMP-5_16 (*r10C*) ISTSDNINRELLTVRMRRQIRQR------------------------------------- 1317

KMP-5_18 (*r10A*) ISTSDNINRELLTVRATHSENAQLTYTIEDGSMAVDSTLEAVKDSAFHLNAQTGVLILRI 1354

KMP-5_11 (*r10B*) ISTSDNINRELLTVR--------------------------------------------- 1309

KMP-6_7 (*r12A*) ISTSDNISRELLTVRATHSENAQLT----------------------------------- 1396

KMP-7_1 (*r12A*) ISTSDNINRELLTVRATHSENAQLT----------------------------------- 1396

KMP-7_2 (*r12B*) ------------------------------------------------------------ 575

KMP-6_3 (*r11A*) ------------------------------------------------------------ 1105

KMP-6_5 (*r11B*) ISTSDNINRELLTVRATHSENAQLTYTIEDGSMVVDSTLEAVKDSACHLNAQTGVLILRI 1431

KMP-6_8 (*r11B*) ISTSDNINRELLTVRATHSENAQLTYTIEDGSMVVDSTLEAVKDSAFHLNAQTGVLILRI 1431

KMP-7_3 (*r12C*) ------------------------------------------------------------ 29

KMP-7_4 (*r12D*) ------------------------------------------------------------ 29

KMP-8_3 (*s7*) ISTSDNINRELLTVRATHSENAQLTYTIEDGSMAVDTTLEAVKDSAFHLNAQTGVLILRI 1430

KMP-8_24 (*s6A*) ISTSDNINRELLTVRATHSENAQLTYTIEGGSMVVDSTLEAVKDSAFHLNAQTGVLILRI 1430

KMP-8_5 (*s6A*) ISTSDNINRELLTVRATHSENAQLTYTIEDGSMVVDSTLEAVKDSAFHLNAQTGVLILRI 1430

KMP-8_46 (*s6A*) ISTSDNINRELLTVRATHSENAQLTYTIEDGSMVVDSTLEAVKDSAFHLNAQTGVLILRI 1430

KMP-8_35 (*s6B*) ISTSDNINRELLTVRATHSENAQLTYTIEDGSMVVDSTLEAVKDSAFHLNAQTGVLILRI 1430

AY198374.1 ISTSDNINRELLTVRATHSENAQLTYTIEDGSMAVDSTLEAVKDSAFHLNAQTGVLILRI 1431

KMP-4_20 (*r8B*) ------------------------------------------------------------ 646

KMP-4_16 (*r8A*) ------------------------------------------------------------ 635

KMP-4_3 (*r8A*) ------------------------------------------------------------ 635

KMP-4_26 (*r8A*) ------------------------------------------------------------ 635

KMP-4_8 (*r9A*) ------------------------------------------------------------ 762

KMP-4_13 (*r9B*) ------------------------------------------------------------ 1227

KMP-5_2 (*r10A*) QPTASMQGMFEFNVIATDPDEKTDTAEVKVYLISSQNRVSFIFLNDVETVESNRDFIAET 1414

KMP-5_16 (*r10C*) ------------------------------------------------------------ 1317

KMP-5_18 (*r10A*) QPTASMQGMFEFNVIATDPDEKTDTAEVKVYLISSQNRVSFIFLNDVETVESNRDFIAET 1414

KMP-5_11 (*r10B*) --------------------------------------------------------IAET 1313

KMP-6_7 (*r12A*) ------------------------------------------------------------ 1396

KMP-7_1 (*r12A*) ------------------------------------------------------------ 1396

KMP-7_2 (*r12B*) ------------------------------------------------------------ 575

KMP-6_3 (*r11A*) ------------------------------------------------------------ 1105

KMP-6_5 (*r11B*) QPTASMQGMFEFNVIATDPDEKTDTAEVKVYLISSQNRVSFIFLNDVETVESNRDFIAET 1491

KMP-6_8 (*r11B*) QPTASMQGMFEFNVIATDPDEKTDTAEVKVYLISSQNRVSFIFLNDVETVESNRDFIAET 1491

KMP-7_3 (*r12C*) ------------------------------------------------------------ 29

KMP-7_4 (*r12D*) ------------------------------------------------------------ 29

KMP-8_3 (*s7*) QPTASMQGMFEFNVIATDPDEKTDTAGVKVYLISSQNRVSFIFLNDVETVESNRDFIAET 1490

KMP-8_24 (*s6A*) QPTASMQGMFEFNVIATDPDEKTDTAEVKVYLISSQNRVSFIFLNDVETVESNRDFIAET 1490

KMP-8_5 (*s6A*) QPTASMQGMFEFNVIATDPDEKTDTAEVKVYLISSQNRVSFIFLNDVETVESNRDFIAET 1490

KMP-8_46 (*s6A*) QPTASMQGMFEFNVIATDPDEKTDTAEVKVYLISSQNRVSFIFLNDVETVESNRDFIAET 1490

KMP-8_35 (*s6B*) QPTASMQGMFEFNVIATDPDEKTDTAEVRVYLISSQNRVSFIFLNDVETVESNRDFIAET 1490

AY198374.1 QPTASMQGMFEFNVIATDPDEKTDTAEVKVYLISSQNRVSFIFLNDVETVESNRDFIAET 1491

KMP-4_20 (*r8B*) ------------------------------------------------------------ 646

KMP-4_16 (*r8A*) ------------------------------------------------------------ 635

KMP-4_3 (*r8A*) ------------------------------------------------------------ 635

KMP-4_26 (*r8A*) ------------------------------------------------------------ 635

KMP-4_8 (*r9A*) ------------------------------------------------------------ 762

KMP-4_13 (*r9B*) ----FGSTV---------------------------DD---------------------- 1234

KMP-5_2 (*r10A*) FSVGFGMTCNIDQVLPGTNDAGVIQEAMAEVHAHFIQDNIPVSADSIEELRSDTQLLRSV 1474

KMP-5_16 (*r10C*) ------------------------------------------------------------ 1317

KMP-5_18 (*r10A*) FSVGFNMTCNIDQVLPGTNDAGVIQEAMAEVHAHFIQDNIPVSADSIEELRSDTQLLRSV 1474

KMP-5_11 (*r10B*) FSVGFNMTCNIDQVLPGTNDAGVIQEAMAEVHAHFIQDNIPVSADSIEELRSDTQLLRSV 1373

KMP-6_7 (*r12A*) ------------------------------------------------------------ 1396

KMP-7_1 (*r12A*) ------------------------------------------------------------ 1396

KMP-7_2 (*r12B*) ------------------------------------------------------------ 575

KMP-6_3 (*r11A*) ------------------------------------------------------------ 1105

KMP-6_5 (*r11B*) FSVGFNMTCNIDQVLPGTNDAGVIQEAMAEVHAHFIQDNIPVSADSIEELRSDTQLLRSP 1551

KMP-6_8 (*r11B*) FSVGFNMTCNIDQVLPGTNDAGVIQEAMAEVHAHFIQDNIPVSADSIEELRSDTQLLRSP 1551

KMP-7_3 (*r12C*) ------------------------------------------------------------ 29

KMP-7_4 (*r12D*) ------------------------------------------------------------ 29

KMP-8_3 (*s7*) FSVGFNMTCNIDQVLPGTNDAGVIQEAMAEVHARFIQDNIPVSADSIEELRSDTQLLRSV 1550

KMP-8_24 (*s6A*) FSVGFNMTCNIDQVLPGTNDAGVIQEAMAEVHAHFIQDNIPVSADSIEELRSDTQLLRSV 1550

KMP-8_5 (*s6A*) FSVGFNMTCNIDQVLPGTNDAGVIQEAMAEVHAHFIQDNIPVSADSIEELRSDTQLLRSV 1550

KMP-8_46 (*s6A*) FSVGFNMTCNIDQVLPGTNDAGVIQEAMAEVHAHFIQDNIPVSADSIEELRSDTQLLRSV 1550

KMP-8_35 (*s6B*) FSVGFNMTCNIDQVLPGTNDAGVIQEAMAEVHAHFIQDNIPVSADSIEELRSDTQLLRSV 1550

AY198374.1 FSVGFNMTCNIDQVLPGTNDAGVIQEAMAEVHAHFIQDNIPVSADSIEELRSDTQLLRSV 1551

KMP-4_20 (*r8B*) ------------------------------------------------------------ 646

KMP-4_16 (*r8A*) ------------------------------------------------------------ 635

KMP-4_3 (*r8A*) ------------------------------------------------------------ 635

KMP-4_26 (*r8A*) ------------------------------------------------------------ 635

KMP-4_8 (*r9A*) ------------------------------------------------------------ 762

KMP-4_13 (*r9B*) ------------------------------------------------------------ 1234

KMP-5_2 (*r10A*) QGVLNQRLLVLNDLVTGVSPDLGTAGVQITIYVLAGLSAILAFLCLILLITFIVRTRALN 1534

KMP-5_16 (*r10C*) ------------------------------------------------------------ 1317

KMP-5_18 (*r10A*) QGVLNQRLLVLNDLVTGVSPDLGTAGVQITIYVLAGLSAILAFLCLILLITFIVRTRALN 1534

KMP-5_11 (*r10B*) QGVLNQRLLVLNDLVTGVSPDLGTAGVRITIYVLAGLSAILAFLCLILLITFIVRTRALS 1433

KMP-6_7 (*r12A*) ------------------------------------------------------------ 1396

KMP-7_1 (*r12A*) ------------------------------------------------------------ 1396

KMP-7_2 (*r12B*) ------------------------------------------------------------ 575

KMP-6_3 (*r11A*) ------------------------------------------------------------ 1105

KMP-6_5 (*r11B*) GESLQRSPFVRPSS---------------------------------------------- 1565

KMP-6_8 (*r11B*) GESLQRSPFVRPSS---------------------------------------------- 1565

KMP-7_3 (*r12C*) ------------------------------------------------------------ 29

KMP-7_4 (*r12D*) ------------------------------------------------------------ 29

KMP-8_3 (*s7*) QGVLNQRLLVLDDLVTGVSPDLGTAGVQITIYVLAGLSAILAFLCLILLITFIVRSRALN 1610

KMP-8_24 (*s6A*) QGVLNQRLLVLNDLVTGVSPDLGTAGVQITIYVLAGLSAILAFLCLILLITFIVRTRALN 1610

KMP-8_5 (*s6A*) QGVLNQRLLVLNDLVTGVSPDLGTAGVQITIYVLAGLSAILAFLCLILLITFIVRTRALN 1610

KMP-8_46 (*s6A*) QGVLNQRLLVLNDLVTGVSPDLGTAGVQITIYVLAGLSAILAFLCLILLITFIVRTRALN 1610

KMP-8_35 (*s6B*) QGVLNQRLLVLNDLVTGVSPDLGTAGVQITIYVLAGLSAILAFLCLILLITFIVRTRALN 1610

AY198374.1 QGVLNQRLLVLNDLVTGVSPDLGTAGVQITIYVLAGLSAILAFLCLILLITFIVRTRALN 1611

KMP-4_20 (*r8B*) ------------------------------------------------------------ 646

KMP-4_16 (*r8A*) ------------------------------------------------------------ 635

KMP-4_3 (*r8A*) ------------------------------------------------------------ 635

KMP-4_26 (*r8A*) ------------------------------------------------------------ 635

KMP-4_8 (*r9A*) ------------------------------------------------------------ 762

KMP-4_13 (*r9B*) ---------EIRLGGFGLNRVGIAAPGTNKHAIEGSNPIWNEQIKAPDFDAISDTSDESD 1285

KMP-5_2 (*r10A*) RRLEALSMTKYGSVDSGLNRVGIAAPGTNKHAIEGSNPIWNEQIKAPDFDAISDTSDESD 1594

KMP-5_16 (*r10C*) ------------------------------------------------------------ 1317

KMP-5_18 (*r10A*) RRLEALSMTKYGSVDSGLNRVGIAAPGTNKHAIEGSNPIWNEQIKAPDFDAISDTSDESD 1594

KMP-5_11 (*r10B*) RRLEALSMTKYGSADSGLNRVGIAAPGTNKHAIEGSNPIWNEQIKAPDFDAISDTSDESD 1493

KMP-6_7 (*r12A*) ------------------------------------------------------------ 1396

KMP-7_1 (*r12A*) ------------------------------------------------------------ 1396

KMP-7_2 (*r12B*) ------------------------------------------------------------ 575

KMP-6_3 (*r11A*) ------------------------------------------------------------ 1105

KMP-6_5 (*r11B*) ------------------------------------------------------------ 1565

KMP-6_8 (*r11B*) ------------------------------------------------------------ 1565

KMP-7_3 (*r12C*) ------------------------------------------------------------ 29

KMP-7_4 (*r12D*) ------------------------------------------------------------ 29

KMP-8_3 (*s7*) RRLEALSMTKYGSVDSGLNRVGIAAPGTNKHAIEGSNPIWNEQIKAPDFDAISDTSDESD 1670

KMP-8_24 (*s6A*) RRLEALSMTKYGSVDSGLNRVGIAAPGTNKHAIEGSNPIWNEQIKAPDFDAISDTSDESD 1670

KMP-8_5 (*s6A*) RHLEALSMTKYGSVDSGLNRVGIAAPGTNKHAIEGSNPIWNEQIKAPDFDAISDTSDESD 1670

KMP-8_46 (*s6A*) RRLEALSMTKYGSVDSGLNRVGIAAPGTNKHAIEGSNPIWNEQIKAPDFDAISDTSGESD 1670

KMP-8_35 (*s6B*) RRLEALSMTKYGSVDSGLNRVGIAAPGTNKHAIEGSNPIWNEQIKAPDFDAISDTSDESD 1670

AY198374.1 RRLEALSMTKYGSVDSGLNRVGIAAPGTNKHAIEGSNPIWNEQIKAPDFDAISDTSDESD 1671

KMP-4_20 (*r8B*) ------------------------------------------------------------ 646

KMP-4_16 (*r8A*) ------------------------------------------------------------ 635

KMP-4_3 (*r8A*) ------------------------------------------------------------ 635

KMP-4_26 (*r8A*) ------------------------------------------------------------ 635

KMP-4_8 (*r9A*) ------------------------------------------------------------ 762

KMP-4_13 (*r9B*) LIGIEDLPQFKSDYFPPEDSESAHAAFSDRTPRGNDAPIAHSSNNFGFNTSPFSAEFTNR 1345

KMP-5_2 (*r10A*) LIGIEDLPQFKSDYFPPEDSESAHAAFSDRTPRGNDAPIAHSSNNFGFNTSPFSAEFTNR 1654

KMP-5_16 (*r10C*) ------------------------------------------------------------ 1317

KMP-5_18 (*r10A*) LIGIEDLPQFKSDYFPPEDSESAHAAFSDRTPRGNDAPIAHSSNNFGFNTSPFSAEFTNR 1654

KMP-5_11 (*r10B*) LIGIEDLPQFKSDYFPPEDSESAHAAFSDRTPRGNDAPIAHSSNNFGFNTSPFSAEFTNR 1553

KMP-6_7 (*r12A*) ------------------------------------------------------------ 1396

KMP-7_1 (*r12A*) ------------------------------------------------------------ 1396

KMP-7_2 (*r12B*) ------------------------------------------------------------ 575

KMP-6_3 (*r11A*) ------------------------------------------------------------ 1105

KMP-6_5 (*r11B*) ------------------------------------------------------------ 1565

KMP-6_8 (*r11B*) ------------------------------------------------------------ 1565

KMP-7_3 (*r12C*) ------------------------------------------------------------ 29

KMP-7_4 (*r12D*) ------------------------------------------------------------ 29

KMP-8_3 (*s7*) LIGIEDLPQFKSDYFPPEDSESAHAAFSDRTPRGNDAPIAHSSNNFGFNTSPFSAEFTNR 1730

KMP-8_24 (*s6A*) LIGIEDLPQFKSDYFPPEDSESAHAAFSDRTPRGNDAPIAHSSNNFGFNTSPFSAEFTNR 1730

KMP-8_5 (*s6A*) LIGIEDLPQFKSDYFPPEDSESAHAAFSDRTPRGNDAPIAHSSNNFGFNTSPFSAEFTNR 1730

KMP-8_46 (*s6A*) LIGIEDLPQFKSDYFPPEDSESAHAAFSDRTPRGNDAPIAHSSNNFGFNTSPFSAEFTNR 1730

KMP-8_35 (*s6B*) LIGIEDLPQFKSDYFPPEDSESAHAAFSDRTPRGNDAPIAHSSNNFGFNTSPFSAEFTNR 1730

AY198374.1 LIGIEDLPQFKSDYFPPEDSESAHAAFSDRTPRGNDAPIAHSSNNFGFNTSPFSAEFTNR 1731

KMP-4_20 (*r8B*) ---- 646

KMP-4_16 (*r8A*) ---- 635

KMP-4_3 (*r8A*) ---- 635

KMP-4_26 (*r8A*) ---- 635

KMP-4_8 (*r9A*) ---- 762

KMP-4_13 (*r9B*) RMRP 1349

KMP-5_2 (*r10A*) RMRP 1658

KMP-5_16 (*r10C*) ---- 1317

KMP-5_18 (*r10A*) RMRP 1658

KMP-5_11 (*r10B*) RMRP 1557

KMP-6_7 (*r12A*) ---- 1396

KMP-7_1 (*r12A*) ---- 1396

KMP-7_2 (*r12B*) ---- 575

KMP-6_3 (*r11A*) ---- 1105

KMP-6_5 (*r11B*) ---- 1565

KMP-6_8 (*r11B*) ---- 1565

KMP-7_3 (*r12C*) ---- 29

KMP-7_4 (*r12D*) ---- 29

KMP-8_3 (*s7*) RMRP 1734

KMP-8_24 (*s6A*) RMRP 1734

KMP-8_5 (*s6A*) RMRP 1734

KMP-8_46 (*s6A*) RMRP 1734

KMP-8_35 (*s6B*) RMRP 1734

AY198374.1 RMRP 1735
