# Supplementary material for: Alternative Splicing and Highly Variable Cadherin Transcripts Associated with Field-Evolved Resistance of Pink Bollworm to Bt Cotton in India
Source: PLoS One. 2014 May 19;9(5):e97900. doi: 10.1371/journal.pone.0097900 (PMC4026531; doi:10.1371/journal.pone.0097900)
Supplement: Figure S7 — Alignment of cadherin cDNA sequences corresponding to susceptible alleles from Akola, Maharashtra (AMH) and Khandwa, Madhya Pradesh (KMP) with the susceptible allele PgCad1 s (AY198374.1). Fourteen clones from four individuals (AMH-1, AMH-2, AMH-3, KMP-8) have 27 allelic sites [single nucleotide polymorphisms that occur more than once and are not from C-to-U or A-to-I (G) RNA editing]. A total of seven s alleles are present from four individuals, including AMH-1 with two alleles, s1 (clone AMH-1_2, KJ480749) and s2 (clones AMH-1_7 and 11, KJ480750), AMH-2 with s3 (clones AMH-2_1, 4, and 5, KJ480751), AMH-3 with s4 (clones AMH-3_1 and 13, KJ480752) and s5 (clone AMH-3_16, KJ480753), and two alleles from KMP-8 (s6A from clones KMP-8_5, 24, and 46, KJ480754; s6B from clone KMP-8_35, KJ480755; and s7 from KMP-8_3, KJ480756). Allelic bases are shown in red boxes. Stars show nucleotides conserved in all of the sequences. Deletions from mis-spliced mRNA are highlighted in gray. (DOCX) [file pone.0097900.s007.docx]

Figure S7. Alignment of cadherin cDNA sequences corresponding to susceptible alleles from Akola, Maharashtra (AMH) and Khandwa, Madhya Pradesh (KMP) with the susceptible allele *PgCad1* *s* (AY198374.1). Fourteen clones from four individuals (AMH-1, AMH-2, AMH-3, KMP-8) have 27 allelic sites [single nucleotide polymorphisms that occur more than once and are not from C-to-U or A-to-I (G) RNA editing]. A total of seven *s* alleles are present from four individuals, including AMH-1 with two alleles, *s1* (clone AMH-1_2, KJ480749) and *s2* (clones AMH-1_7 and 11, KJ480750), AMH-2 with *s3* (clones AMH-2_1, 4, and 5, KJ480751), AMH-3 with *s4* (clones AMH-3_1 and 13, KJ480752) and *s5* (clone AMH-3_16, KJ480753), and two alleles from KMP-8 (*s6A* from clones KMP-8_5, 24, and 46, KJ480754; *s6B* from clone KMP-8_35, KJ480755; and *s7* from KMP-8_3, KJ480756). Allelic bases are shown in red boxes. Stars show nucleotides conserved in all of the sequences. Deletions from mis-spliced mRNA are highlighted in gray.

KMP-8_35 ATGGCGGGTGACGCCTGCATACTGGTGACGGTGCTTCTCACCTTCGCAACATCAGTTTTC 60

KMP-8_5 ATGGCGGGTGACGCCTGCATACTGGTGACGGTGCTTCTCACCTTCGCAACATCAGTTTTC 60

KMP-8_24 ATGGCGGGTGACGCCTGCATACTGGTGACGGTGCTTCTCACCTTCGCAACATCAGTTTTC 60

KMP-8_46 ATGGCGGGTGACGCCTGCATACTGGTGACGGTGCTTCTCACCTTCGCAACATCAGTTTTC 60

AMH-3_16 ATGGCGGGTGACGCCTGCATACTGGTGACGGTGCTTCTGACCTTCGCAACATCAGTTTTC 60

AMH-2_5 ATGGCGGGTGACGCCTGCATACTGGTGACGGTGCTTCTGATCTTCGCAATATCAGTTTTC 60

AMH-2_1 ATGGCGGGTGACGCCTGCATACTGGTGACGGTGCTTCTGATCTTCGCAATATCAGTTTTC 60

AMH-2_4 ATGGCGGGTGACGCCTGCATACTGGTGACGGTGCTTCTGATCTTCGCAATATCAGTTTTC 60

AMH-3_1 ATGGCGGGTGACGCCTGCATACTGGTGACGGTGCTTCTGGCCTTCGCAACATCAGTTTTC 60

AMH-3_13 ATGGCGGGTGACGCCTGCATACTGGTGACGGTGCTTCTGACCTTCGCAACATCAGTTTTC 60

AMH-1_7 ATGGCGGGTGACGCCTGCATACTGGTGACGGTGCTTCTGACCTTCGCAACATCAGTTTTC 60

AMH-1_11 ATGGCGGGTGACGCCTGCATACTGGTGACGGTGCTTCTGACCTTCGCAACATCAGTTTTC 60

KMP-8_3 ATGGCGGGTGACGCCTGCATACTGGTGACGGTGCTTCTCACCTTCGCAACATCAGTTTTC 60

AMH-1_2 ATGGCGGGTGACGCCTGCATACTGGTGACGGTGCTTCTCACCTTCGCAACATCAGTTTTC 60

AY198374.1 ATGGCGGGTGACGCCTGCATACTGGTGACGGTGCTTCTGACCTTCGCAACATCAGTTTTC 60

************************************** . ******** **********

KMP-8_35 GGGCAAGAAACAGCATCGTCGAGATGTTACTACATGACTGACGCTATTCCGAGAGAACCG 120

KMP-8_5 GGGCAAGAAAC---ATCGTCGAGATGTTACTACATGACTGACGCTATTCCGAGAGAACCG 117

KMP-8_24 GGGCAAGAAAC---ATCGTCGAGATGTTACTACATGACTGACGCTATTCCGAGAGAACCG 117

KMP-8_46 GGGCAAGAAAC---ATCGTCGAGATGTTACTACATGACTGACGCTATTCCGAGAGAACCG 117

AMH-3_16 GGGCAAGAAAC---ATCGTCGAGATGTTACTACATGACTGACGCTATTCCGAGAGAACCG 117

AMH-2_5 GGGCAAGAAACAGCATCGTCGAGATGTTACTACATGACTGACGCTATTCCGAGAGAACCG 120

AMH-2_1 GGGCAAGAAACAGCATCGTCGAGATGTTACTACATGACTGACGCTATTCCGAGAGAACCG 120

AMH-2_4 GGGCAAGAAACAGCATCGTCGAGATGTTACTACATGACTGACGCTATTCCGAGAGAACCG 120

AMH-3_1 GGGCAAGAAACAGCATCGTCGAGATGTTACTACATGACTGACGCTATTCCGAGAGAACCG 120

AMH-3_13 GGGCAAGAAACAGCATCGTCGAGATGTTACTACATGACTGACGCTATTCCGAGAGAACCG 120

AMH-1_7 GGGCAAGAAACAGCATCGTCGAGATGTTACTACATGACTGACGCTATTCCGAGAGAACCG 120

AMH-1_11 GGGCAAGAAACAGCATCGTCGAGATGTTACTACATGACTGACGCTATTCCGAGAGAACCG 120

KMP-8_3 GGGCAAGAAAC---ATCGTCGAGATGTTACTACATGACTGACGCTATTCCGAGAGAACCG 117

AMH-1_2 GGGCAAGAAACAACATCGTCGAGATGTTACTACATGACTGACGCTATTCCGAGGGAACCG 120

AY198374.1 GGGCAAGAAACAACATCGTCGAGATGTTACTACATGACTGACGCTATTCCGAGAGAACCG 120

*********** ***************************************.******

KMP-8_35 AAACCGGATGATTTGCCTGATTTAGAATGGACTGGTGGATGGACCGACTGGCCTTTGATC 180

KMP-8_5 AAACCGGATGATTTGCCTGATTTAGAATGGACTGGTGGATGGACCGACTGGCCTTTGATC 177

KMP-8_24 AAACCGGATGATTTGCCTGATTTAGAATGGACTGGTGGATGGACCGACTGGCCTTTGATC 177

KMP-8_46 GAACCGGATGATTTGCCTGATTTAGAATGGACTGGTGGATGGACCGACTGGCCTTTGATC 177

AMH-3_16 AAACCGGATGATTTGCCTGATTTAGAATGGACTGGTGGATGGACCAACTGGCCTTTGATC 177

AMH-2_5 AAACCGGATGATTTGCCTGATTTAGAATGGACTGGTGGATGGACCGACTGGCCTTTGATC 180

AMH-2_1 AAACCGGATGATTTGCCTGATTTAGAATGGACTGGTGGATGGACCGACTGGCCTTTGATC 180

AMH-2_4 AAACCGGATGATTTGCCTGATTTAGAATGGACTGGTGGATGGACCGACTGGCCTTTGATC 180

AMH-3_1 AAACCGGATGATTTGCCTGATTTAGAATGGACTGGTGGATGGACCGACTGGCCTCTGATC 180

AMH-3_13 AAACCGGATGATTTGCCTGATTTAGAATGGACTGGTGGATGGACCGACTGGCCTTTGATC 180

AMH-1_7 AAACCGGATGATTTGCCTGATTTAGAATGGACTGGTGGATGGACCGACTGGCCTTTGATC 180

AMH-1_11 AAACCGGATGATTTACCTGATTTAGAATGGACTGGTGGATGGACCGACTGGCCTTTGATC 180

KMP-8_3 AAACCGGATGATTTGCCTGATTTAGAATGGACTGGTGGATGGACCGACTGGCCTTTGATC 177

AMH-1_2 AAACCGGATGATTTGCCTGACTTAGAATGGACTGGTGGATGGACCGACTGGCCTTTGATC 180

AY198374.1 AAACCGGATGATTTGCCTGATTTAGAATGGACTGGTGGATGGACCGACTGGCCTTTGATC 180

.*************.***** ************************.******** *****

KMP-8_35 CCGGCTGAGCCAAGAGACGACGTGTGTATAAACGGCTGGTACCCACAACTCACCAGCACT 240

KMP-8_5 CCGGCTGAGCCAAGAGACGACGTGTGCATAAACGGCTGGTACCCACAACTCACCAGCACT 237

KMP-8_24 CCGGCTGAGCCAAGAGACGACGTGTGCATAAACGGCTGGTACCCACAACTCACCAGCACT 237

KMP-8_46 CCGGCTGAGCCAAGAGACGACGTGTGCATAAACGGCTGGTACCCACAACTCACCAGCACT 237

AMH-3_16 CCGGCTGAGCCAAGAGACGACGTGTGCATAAACGGCTGGTACCCACAACTCACCAGCACT 237

AMH-2_5 CCGGCTGAGCCAAGAGACGACGTGTGCATAAACGACTGGTACCCACAACTCACCAGCACT 240

AMH-2_1 CCGGCTGAGCCAAGAGACGACGTGTGCATAAACGGCTGGTACCCACAACTCACCAGCACT 240

AMH-2_4 CCGGCTGAGCCAAGAGACGACGTGTGCATAAACGGCTGGTACCCACAACTCACCAGCACT 240

AMH-3_1 CCGGCTGAGCCAAGAGACGACGTGTGCATAAACGGCTGGTACCCACAACTCACCAGCACT 240

AMH-3_13 CCGGCTGAGCCAAGAGACGACGTGTGCATAAACGGCTGGTACCCACAACTCACCAGCACT 240

AMH-1_7 CCGGCTGAGCCAAGAGACGACGTGTGCATAAACGGCTGGTACCCACAACTCACCAGCACT 240

AMH-1_11 CCGGCTGAGCCAAGAGACGACGTGTGCATAAACGGCTGGTACCCACAACTCACCAGCACT 240

KMP-8_3 CCGGCTGAGCCAAGAGACGACGTGTGCATAAACGGCTGGTACCCACAACTCACCAGCACT 237

AMH-1_2 CCGGCTGAGCCAAGAGACGACGTGTGCATAAACGGCTGGTACCCACAACTCACCAGCACT 240

AY198374.1 CCGGCTGAGCCAAGAGACGACGTGTGCATAAACGGCTGGTACCCACAACTCACCAGCACT 240

************************** *******.*************************

KMP-8_35 TCTCTCGGCACCATCATCATCCACATGGAAGAGGAGATCGAGGGAGATGTCGCTATCGCT 300

KMP-8_5 TCTCTCGGCACCATCATCATCCACATGGAAGAGGAGATCGAGGGAGATGTCGCTATCGCT 297

KMP-8_24 TCTCTCGGCACCATCATCATCCACATGGAAGAGGAGATCGAGGGAGATGTCGCTATCGCT 297

KMP-8_46 TCTCTCGGCACCATCATCATCCACATGGAAGAGGAGATCGAGGGAGATGTCGCTATCGCT 297

AMH-3_16 TCTCTCGGCACCATCATCATCCACATGGAAGAGGAGATCGAGGGAGATGTTGCTATCGCT 297

AMH-2_5 TCTCTCGGCACCATCATCATCCACATGGAAGAGGAGATCGAGGGAGATGTTGCTATCGCT 300

AMH-2_1 TCTCTCGGCACCATCATCATCCACATGGAAGAGGAGATCGAGGGAGATGTTGCTATCGCT 300

AMH-2_4 TCTCTCGGCACCATCATCATCCACATGGAAGAGGAGATCGAGGGAGATGTTGCTATCGCT 300

AMH-3_1 TCTCTCGGCACCATCATCATCCACATGGAAGAGGAGATCGAGGGAGATGTTGCTATCGCT 300

AMH-3_13 TCTCTCGGCACCATCATCATCCACATGGAAGAGGAGATCGAGGGAGATGTTGCTATCGCT 300

AMH-1_7 TCTCTCGGCACCATCATCATCCACATGGAAGAGGAGATCGAGGGAGATGTTGCTATCGCT 300

AMH-1_11 TCTCTCGGCACCATCATCATCCACATGGAAGAGGAGATCGAGGGAGATGTTGCTATCGCT 300

KMP-8_3 TCTCTCGGCACCATCATCATCCACATGGAAGAGGAGATCGAGGGAGATGTCGCTATCGCT 297

AMH-1_2 TCTCTCGGCACCATCATCATCCACATGGAAGAGGAGATCGAGGGAGATGTTGCTATCGCT 300

AY198374.1 TCTCTCGGCACCATCATCATCCACATGGAAGAGGAGATCGAGGGAGATGTTGCTATCGCT 300

************************************************** *********

KMP-8_35 AAACTTAACTATGATGGTTCTGGAACCCCAGAAATTGTCCAGCCGATGGTTATAGGATCT 360

KMP-8_5 AAACTTAACTATGATGGTTCTGGAACCCCAGAAATTGTCCAGCCGATGGTTATAGGATCT 357

KMP-8_24 AAACTTAACTATGATGGTTCTGGAACCCCAGAAATTGTCCAGCCGATGGTTATAGGATCT 357

KMP-8_46 AAACTTAACTATGATGGTTCTGGAACCCCAGAAATTGTCCAGCCGATGGTTATAGGATCT 357

AMH-3_16 AAACTTAACTATGATGGTTCTGGAACCCCAGAAATTGTCCAGCCGATGGTTATAGGATCT 357

AMH-2_5 AAACTTAACTATGATGGTTCTGGAACCCCAGAAATTGTCCAGCCGATGGTTATAGGATCT 360

AMH-2_1 AAACTTTACTATGATGGTTCTGGAACCCCAGAAATTGTCCAGCCGATGGTTATAGGATCT 360

AMH-2_4 AAACTTAACTATGATGGTTCTGGAACCCCAGAAATTGTCCAGCCGATGGTTATAGGATCT 360

AMH-3_1 AAACTTAACTATGATGGTTCTGGAACCCCAGAAATTGTCCAGCCGATGGTTATAGGATCT 360

AMH-3_13 AAACTTAACTATGATGGTTCTGGAACCCCAGAAATTGTCCAGCCGATGGTTATAGGATCT 360

AMH-1_7 AAACTTAACTATGATGGTTCTGGAACCCCAGAAATTGTCCAGCCGATGGTTATAGGATCT 360

AMH-1_11 AAACTTAACTATGATGGTTCTGGAACCCCAGAAATTGTCCAGCCGATGGTTATAGGATCT 360

KMP-8_3 AAACTTAACTATGATGGTTCTGGAACCCCAGAAATTGTCCAGCCGATGGTTATAGGATCT 357

AMH-1_2 AAACTTAACTATGATGGTTCTGGAACCCCAGAAATTGTCCAGCCGATGGTTATAGGATCT 360

AY198374.1 AAACTTAACTATGATGGTTCTGGAACCCCAGAAATTGTCCAGCCGATGGTTATAGGATCT 360

******:*****************************************************

KMP-8_35 TTTAACCTGCTAAGTCCAGAGATCCGGAATGAAAACGGGGCGTGGTACCTTTATATAACC 420

KMP-8_5 TTTAACCTGCTAAGTCCAGAGATCCGGAATGAAAACGGGGCGTGGTACCTTTATATAACC 417

KMP-8_24 TTTAACCTGCTAAGTCCAGAGATCCGGAATGAAAACGGGGCGTGGTACCTTTATATAACC 417

KMP-8_46 TTTAACCTGCTAAGTCCAGAGATCCGGAATGAAAACGGGGCGTGGTACCTTTATATAACC 417

AMH-3_16 TTTAACCTGCTAAGTCCAGAGATCCGGAATGAAAACGGGGCGTGGTACCTTTATATAACC 417

AMH-2_5 TTTAACCTGCTAAGTCCAGGGATCCGGAATGAAAACGGGGCGTGGTACCTTTATATAACC 420

AMH-2_1 TTTAACCTGCTAAGTCCAGAGATCCGGAATGAAAACGGGGCGTGGTACCTTTATATAACC 420

AMH-2_4 TTTAACCTGCTAAGTCCAGAGATCCTGAATGAAAACGGGGCGTGGTACCTTTATATAACC 420

AMH-3_1 TTTAACCTGCTAAGTCCAGAGATCCGGAATGAAAACGGGGCGTGGTACCTTTATATAACC 420

AMH-3_13 TTTAACCTGCTAAGTCCAGAGATCCGGAATGAAAACGGGGCGTGGTACCTTTATATAACC 420

AMH-1_7 TTTAACCTGCTAAGTCCAGAGATCCGGAATGAAAACGGGGCGTGGTACCTTTATATAACC 420

AMH-1_11 TTTAACCTGCTAAGTCCAGAGATCCGGAATGAAAACGGGGCGTGGTACCTTTATATAACC 420

KMP-8_3 TTTAACCTGCTAAGTCCAGAGATCCGGAATGAAAACGGGGCGTGGTACCTTTATATAACC 417

AMH-1_2 TTTAACCTGCTAAGTCCAGGGATCCGGAATGAAAACGGGGCGTGGTACCTTTATATAACC 420

AY198374.1 TTTAACCTGCTAAGTCCAGAGATCCGGAATGAAAACGGGGCGTGGTACCTTTATATAACC 420

*******************.***** **********************************

KMP-8_35 AATAGGCAAGATTATGAAACACCAACAATGCGTCGGTATACATTCGACGTCCGAGTGCCA 480

KMP-8_5 AATAGGCAAGATTATGAAACACCAACAATGCGTCGGTATACATTCGACGTCCGAGTGCCA 477

KMP-8_24 AATAGGCAAGATTATGAAACACCAACAATGCGTCGGTATACATTCGACGTCCGAGTGCCA 477

KMP-8_46 AATAGGCAGGATTATGAAACACCAACAATGCGTCGGTATACATTCGACGTCCGAGTGCCA 477

AMH-3_16 AATAGGCAAGATTATGAAACGCCAACAATGCGTCGGTATACATTCGACGTCCGAGTGCCA 477

AMH-2_5 AATAGGCAAGATTATGAAACACCAACAATGCGTCGGTATACATTCGACGTCCGAGTGCCA 480

AMH-2_1 AATAGGCAAGATTATGAAACACCAACAATGCGTCGGTATACATTCGACGTCCGAGTGCCA 480

AMH-2_4 AATAGGCAAGATTATGAAACACCAACAATGCGTCGGTATACATTCGACGTCCGAGTGCCA 480

AMH-3_1 AATAGGCAAGATTATGAAACACCAACAATGCGTCGGTATACATTCGACGTCCGAGTGCCA 480

AMH-3_13 AATAGGCAAGATTATGAAACACCAACAATGCGTCGGTATACATTCGACGTCCGAGTGCCA 480

AMH-1_7 AATAGGCAAGATTATGAAACACCAACAATGCGTCGGTATACATTCGACGTCCGAGTGCCA 480

AMH-1_11 AATAGGCAAGATTATGAAACACCAACAATGCGTCGGTATACATTCGACGTCCGAGTGCCA 480

KMP-8_3 AATAGGCAAGATTATGAAACACCAACAATGCGTCGGTATACATTCGACGTCCGAGTGCCA 477

AMH-1_2 AATAGGCAAGATTATGAAACACCAACAATGCGTCGGTATACATTCGACGTCCGAGTGCCA 480

AY198374.1 AATAGGCAAGATTATGAAACACCAACAATGCGTCGGTATACATTCGACGTCCGAGTGCCA 480

********.***********.***************************************

KMP-8_35 GACGAGACTCGTGCGGCACGAGTGAGTCTCTCCATCGAAAACATTGACGATAACGACCCT 540

KMP-8_5 GACGAGACTCGTGCGGCACGAGTGAGTCTCTCCATCGAAAACATTGACGATAACGACCCT 537

KMP-8_24 GACGAGACTCGTGCGGCACGAGTGAGTCTCTCCATCGAAAACATTGACGATAACGACCCT 537

KMP-8_46 GACGAGACTCGTGCGGCACGAGTGAGTCTCTCCATCGAAAACATTGACGATAACGACCCT 537

AMH-3_16 GACGAGACTCGTGCCGCACGAGTGAGTCTCTCCATCGAAAACATTGACGATAACGACCCT 537

AMH-2_5 GACGAGACTCGTGCGGCACGAGTGAGTCTCTCCATCGAAAACATTGACGATAACGACCCT 540

AMH-2_1 GACGAGACTCGTGCGGCACGAGTGAGTCTCTCCATCGAAAACATTGACGATAACGACCCT 540

AMH-2_4 GACGAGACTCGTGCGGCACGAGTGAGTCTCTCCATCGAAAACATTGACGATAACGACCCT 540

AMH-3_1 GACGAGACTCGTGCCGCACGAGTGAGTCTCTCCATCGAAAACATTGACGATAACGACCCT 540

AMH-3_13 GACGAGACTCGTGCCGCACGAGTGAGTCTCTCCATCGAAAACATTGACGATAACGACCCT 540

AMH-1_7 GACGAGACTCGTGCCGCACGAGTGAGTCTCTCCATCGAAAACATTGACGATAACGACCCT 540

AMH-1_11 GACGAGACTCGTGCCGCACGAGTGAGTCTCTCCATCGAAAACATTGACGATAACGACCCT 540

KMP-8_3 GACGAGACTCGTGCGGCACGAGTGAGTCTCTCCATCGAAAACATTGACGATAACGACCCT 537

AMH-1_2 GACGAGACTCGTGCGGCACGAGTGAGTCCGTCCATCGAAAACATTGACGATAACGACCCT 540

AY198374.1 GACGAGACTCGTGCGGCACGAGTGAGTCTGTCCATCGAAAACATTGACGATAACGACCCT 540

************** ************* ******************************

KMP-8_35 ATCGTCAGGGTGCTAGACGCTTGCCTAGTGCCGGAATTGGGGGAGCCTCGACTAACTGAC 600

KMP-8_5 ATCGTCAGGGTGCTAGACGCTTGCCAAGTGCCGGAATTGGGGGAGCCTCGACTAACTGAC 597

KMP-8_24 ATCGTCAGGGTGCTAGACGCTTGCCAAGTGCCGGAATTGGGGGAGCCTCGACTAACTGAC 597

KMP-8_46 ATCGTCAGGGTGCTAGACGCTTGCCAAGTGCCGGAATTGGGGGAGCCTCGACTAACTGAC 597

AMH-3_16 ATCGTCAGGGTGCTAGACGCTTGCCAAGTGCCGGAATTGGGGGAGCCTCGACTAACTGAC 597

AMH-2_5 ATCGTCAGGGTGCTAGACGCTTGCCAAGTGCCGGAATTGGGGGAGCCTCGACTAACAGAC 600

AMH-2_1 ATCGTCAGGGTGCTAGACGCTTGCCAAGTGCCGGAATTGGGGGAGCCTCGACTAACAGAC 600

AMH-2_4 ATCGTCAGGGTGCTAGACGCTTGCCAAGTGCCGGAATTGGGGGAGCCTCGACTAACAGAC 600

AMH-3_1 ATCGTCAGGGTGCTAGACGCTTGCCAAGTGCCGGAATTGGGGGAGCCTCGACTAACTGAC 600

AMH-3_13 ATCGTCAGGGTGCTAGACGCTTGCCAAGTGCCGGAATTGGGGGAGCCTCGACTAACTGAC 600

AMH-1_7 ATCGTCAGGGTGCTAGACGCTTGCCAAGTGCCGGAATTGGGGGAGCCTCGACTAACTGAC 600

AMH-1_11 ATCGTCAGGGTGCTAGACGCTTGCCAAGTGCCGGAATTGGGGGAGCCTCGACTAACTGAC 600

KMP-8_3 ATCGTCAGGGTGCTAGACGCTTGCCAAGTGCCGGAATTGGGGGAGCCTCGACTAACTGAC 597

AMH-1_2 ATCGTCAGGGTGCTAGACGCTTGCCAAGTGCCGGAATTGGGGGAGCCTCGACTAACAGAC 600

AY198374.1 ATCGTCAGGGTGCTAGACGCTTGCCAAGTGCCGGAATTGGGGGAGCCTCGACTAACAGAC 600

*************************:******************************:***

KMP-8_35 TGCGTTTACCAAGTGTCAGACGAAGATGGGAGGCTTAGTATCGAGCCCATGACATTCCGC 660

KMP-8_5 TGCGTTTACCAAGTGTCAGACGAAGATGGGAGGCTTAGTATCGAGCCCATGACATTCCGC 657

KMP-8_24 TGCGTTTACCAAGTGTCAGACGAAGATGGGAGGCTTAGTATCGAGCCCATGACATTCCGC 657

KMP-8_46 TGCGTTTACCAAGTGTCAGACGAAGATGGGAGGCTTAGTATCGAGCCCATGACATTCCGC 657

AMH-3_16 TGCGTTTACCGAGTGTCAGACGAAGATGGGAGGCTTAGTATCGAGCCCATGACATTCCGC 657

AMH-2_5 TGCGTTTACCAAGTGTCAGACGAAGATGGGAGGCTTAGTATCGAGCCCATGACATTCCGC 660

AMH-2_1 TGCGTTTACCAAGTGTCAGACGAAGATGGGAGGCTTAGTATCGAGCCCATGACATTCCGC 660

AMH-2_4 TGCGTTTACCAAGTGTCAGACGAAGATGGGAGGCTTAGTATCGAGCCCATGACATTCCGC 660

AMH-3_1 TGCGCTTACCAAGTGTCAGACGAAGATGGGAGGCTTAGTATCGAGCCCATGACATTCCGC 660

AMH-3_13 TGCGTTTACCAAGTGTCAGACGAAGATGGGAGGCTTAGTATCGAGCCCATGACATTCCGC 660

AMH-1_7 TGCGTTTACCAAGTGTCAGACGAAGATGGGAGGCTTAGTATCGAGCCCATGACATTCCGC 660

AMH-1_11 TGCGTTTACCAAGTGTCAGACGAAGATGGGAGGCTTAGTATCGAGCCCATGACATTCCGC 660

KMP-8_3 TGCGTTTACCAAGTGTCAGACGAAGATGGGAGGCTTAGTATCGAGCCCATGACATTCCGC 657

AMH-1_2 TGCGTTTACCAAGTGTCAGACGAAGATGGGAGGCTTAGTATCGAGCCCATGACATTCCGC 660

AY198374.1 TGCGTTTACCAAGTGTCAGACGAAGATGGGAGGCTTAGTATCGAGCCCATGACATTCCGC 660

**** *****.*************************************************

KMP-8_35 CTCACATCAGACCGTGAAGACGTACAGATATTCTACGTGGAGCCAGCTCACATTACTGGA 720

KMP-8_5 CTCACATCAGACCGTGAAGACGTACAGATATTCTACGTGGAGCCAGCTCACATTACTGGA 717

KMP-8_24 CTCACATCAGACCGTGAAGACGTACAGATATTCTACGTGGAGCCAGCTCACATTACTGGA 717

KMP-8_46 CTCACATCAGACCGTGAAGACGTACAGATATTCTACGTGGAGCCAGCTCACATTACTGGA 717

AMH-3_16 CTCACATCAGACCGTGAAGACGTACAGATATTCTATGTGGAGCCAGCTCACATTACTGGT 717

AMH-2_5 CTCACATCAGACCGTGAAGACGTACAGATATTCTATGTGGAGCCAGCTCACATTACTGGT 720

AMH-2_1 CTCACATCAGACCGTGAAGACGTACAGATATTCTATGTGGAGCCAGCTCACATTACTGGT 720

AMH-2_4 CTCACATCAGACCGTGAAGACGTACAGATATTCTATGTGGAGCCAGCTCACATTACTGGT 720

AMH-3_1 CTCACATCGGACCGTGAAGACGTACAGATATTCTATGTGGAGCCAGCTCACATTACTGGT 720

AMH-3_13 CTCACATCAGACCGTGAAGACGTACAGATATTCTATGTGGAGCCAGCTCACATTACTGGT 720

AMH-1_7 CTCACATCAGACCGTGAAGACGTGCAGATATTCTATGTGGAGCCAGCTCACATTACTGGT 720

AMH-1_11 CTCACATCAGACCGTGAAGACGTACAGATATTCTATGTGGAGCCAGCTCACATTACTGGT 720

KMP-8_3 CTCACATCAGACCGTGAAGACGTACAGATATTCTACGTGGAGCCAGCTCACATTACTGGA 717

AMH-1_2 CTCACATCAGACCGTGAAGACGTACAGATATTCTATGTGGAGCCAGCTCACATTACTGGT 720

AY198374.1 CTCACATCAGACCGTGAAGACGTACAGATATTCTATGTGGAGCCAGCTCACATTACTGGT 720

********.**************.*********** ***********************:

KMP-8_35 GATTGGTTCAACATGCAAATTACTATCGGTATCCTATCAGCGCTTAACTTCGAAAGCAGC 780

KMP-8_5 GATTGGTTCAACATGCAAATTACTATCGGTATCCTATCAGCGCTTAACTTCGAAAGCAAC 777

KMP-8_24 GATTGGTTCAACATGCAAATTACTATCGGTATCCTATCAGCGCTTAACTTCGAAAGCAAC 777

KMP-8_46 GATTGGTTCAACATGCAAATTACTATCGGTATCCTATCAGCGCTTAACTTCGAAAGCAAC 777

AMH-3_16 GATTGGTTCAACATGCAAATTACTATCGGTATCCTATCAGCGCTTAACTTCGAAAGCAAC 777

AMH-2_5 GATTGGTTCAACATGCAAATTACTATCGGTATCCTATCAGCGCTTAACTTCGAAAGCAAC 780

AMH-2_1 GATTGGTTCAACATGCAAATTACTATCGGTATCCTATCAGCGCTTAACTTCGAAAGCAAC 780

AMH-2_4 GATTGGTTCAACATGCAAATTACTATCGGTATCCTATCAGCGCTTAACTTCGAAAGCAAC 780

AMH-3_1 GATTGGTTCAACATGCAAATTACTATCGGTATCCTATCAGCGCTTAACTTCGAAAGCAAC 780

AMH-3_13 GATTGGTTCAACATGCAAATTACTATCGGTATCCTATCAGCGCTTAACTTCGAAAGCAAC 780

AMH-1_7 GATTGGTTCAACATGCAAATTACTATCGGTATCCTATCAGCGCTTAACTTCGAAAGCAAC 780

AMH-1_11 GATTGGTTCAACATGCAAATTACTATCGGTATCCTATCAGCGCTTAACTTCGAAAGCAAC 780

KMP-8_3 GATTGGTTCAACATGCAAATTACTATCGGTATCCTATCAGCGCTTAACTTCGAAAGCAAC 777

AMH-1_2 GATTGGTTCAACATGCAAATTACTATCGGTATCCTATCAGCGCTTAACTTCGAAAGCAAC 780

AY198374.1 GATTGGTTCAACATGCAAATTACTATCGGTATCCTATCAGCGCTTAACTTCGAAAGCAAC 780

**********************************************************.*

KMP-8_35 CCGCTTCACATCTTTCAAATCACTGCTTTGGACTCCTGGCCCAACAACCATACGGTGACG 840

KMP-8_5 CCGCTTCACATCTTTCAAATCACTGCTTTGGACTCCTGGCCCAACAACCATACGGTGACG 837

KMP-8_24 CCGCTTCACATCTTTCAAATCACTGCTTTGGACTCCTGGCCCAACAACCATACGGTGACG 837

KMP-8_46 CCGCTTCACATCTTTCAAATCACTGCTTTGGACTCCTGGCCCAACAACCATACGGTGACG 837

AMH-3_16 CCGCTTCACATCTTTCAAATCACTGCTTTGGACTCCTGGCCCAACAACCATACGGTGACG 837

AMH-2_5 CCGCTGCACATCTTTCAAATCACTGCTTTGGACTCCTGGCCCAACAACCATACGGTGACG 840

AMH-2_1 CCGCCGCACATCTTTCAAATCACTGCTTTGGACTCCTGGCCCAACTACCATACGGTGACG 840

AMH-2_4 CCGCTGCACATCTTTCAAATCACTGCTTTGGACTCCTGGCCCAACAACCATACGGTGACG 840

AMH-3_1 CCGCTGCACATCTTTCAAATCACTGCTTTGGACTCCTGGCCCAACAACCATACGGTGACG 840

AMH-3_13 CCGCTGCACATCTTTCAAATCACTGCTTTGGACTCCTGGCCCAACAACCATACGGTGACG 840

AMH-1_7 CCGCTGCACATCTTTCAAATCACTGCTTTGGACTCCTGGCCCAACAACCATACGGTGACG 840

AMH-1_11 CCGCTGCACATCTTTCAAATCACTGCTTTGGACTCCTGGCCCAACAACCATACGGTGACG 840

KMP-8_3 CCGCTTCACATCTTTCAAATCACTGCTTTGGACTCCTGGCCCAACAACCATACGGTGACG 837

AMH-1_2 CCGCTGCACATCTTTCAAATCACTGCTTTGGACTCCTGGCCCAACAACCATACGGTGACG 840

AY198374.1 CCGCTGCACATCTTTCAAATCACTGCTTTGGACTCCTGGCCCAACAACCATACGGTGACG 840

**** ***************************************:**************

KMP-8_35 GTGATGGTGCAAGTCCAGAATGTGGAGCACCGACCGCCGCGATGGATGGAAATCTTCGCA 900

KMP-8_5 GTGATGGTGCAAGTCCAGAATGTGGAGCACCGACCGCCGCGATGGATGGAAATCTTCGCA 897

KMP-8_24 GTGATGGTGCAAGTCCAGAATGTGGAGCACCGACCGCCGCGATGGATGGAAATCTTCGCA 897

KMP-8_46 GTGATGGTGAAAGTCCGGAATGTGGAGCACCGACCGCCGCGATGGATGGAAATCTTCGCA 897

AMH-3_16 GTGATGGTGCAAGTCCAGAATGTGGAACACCGACCGCCGCGATGGATGGAAATCTTCGCA 897

AMH-2_5 GTGATGGTGCGAGTCCAGAATGTGGAGTACCGACCGCCGCGATGGATGGAAATCTTCGCA 900

AMH-2_1 GTGATGGTGCAAGTCCAGAATGTGGAGCACCGACCGCCGCGATGGATGGAAATCTTCGCA 900

AMH-2_4 GTGATGGTGCAAGTCCAGAATGTGGAGCACCGACCGCCGCGATGGATGGAAATCTTCGCA 900

AMH-3_1 GTGATGGTGCAAGTCCAGAATGTGGAACACCGACCGCCGCGATGGATGGAAATCTTCGCA 900

AMH-3_13 GTGATGGTGCAAGTCCAGAATGTGGAACACCGACCGCCGCGATGGATGGAAATCTTCGCA 900

AMH-1_7 GTGATGGTGCAAGTCCAGAATGTGGAACACCGACCGCCGCGATGGATGGAAATCTTCGCA 900

AMH-1_11 GTGATGGTGCAAGTCCAGAATGTGGAACACCGACCGCCGCGATGGATGGAAATCTCCGCA 900

KMP-8_3 GTGATGGTGCTAGTCCAGAATGTGGAACACCGACCGCCGCGATGGATGGAAATCTTCGCA 897

AMH-1_2 GTGATGGTGCAAGTCCAGAATGTGGAACACCGACCGCCGCGATGGATGGAAATCTTCGCA 900

AY198374.1 GTGATGGTGCAAGTTCAGAATGTGGAGCACCGACCGCCGCGATGGATGGAAATCTTCGCA 900

*********. *** *.*********. *************************** ****

KMP-8_35 GTCCAGCAGTTTGACGAGATGACGGAGCAGCAGTTCCAGGTGCGCGCCATCGACGGAGAC 960

KMP-8_5 GTCCAGCAGTTTGACGAGATGACGGAGCAGCAATTCCAGGTGCGCGCCATCGACGGAGAC 957

KMP-8_24 GTCCAGCAGTTTGACGAGATGACGGAGCAGCAATTCCAGGTGCGCGCCATCGACGGAGAC 957

KMP-8_46 GTCCAGCGGTTTGACGAGATGACGGAGCAGCAATTCCAGGTGCGCGCCATCGACGGAGAC 957

AMH-3_16 GTCCAGCAGTTTGACGAGATGACGGAGCAGCAATTCCAGGTGCGCGCCATCGACGGAGAC 957

AMH-2_5 GTCCAGCAGTTTGACGAGATGACGGAGCAGCAATTCCAGGTGCGCGCCATCGACGGAGAC 960

AMH-2_1 GTCCAGCAGTTTGACGAGATGACGGAGCAGCAATTCCAGGTGCGCGCTATCGACGGAGAC 960

AMH-2_4 GCCCAGCAGTTTGACGAGATGACGGAGCAGCAATTCCAGGTGCGCGCCATCGACGGAGAC 960

AMH-3_1 GTCCAGCAGTTTGACGAGATGACGGAGCAGCAATTCCAGGTGCGCGCCATCGACGGAGAC 960

AMH-3_13 GTCCAGCAGTTTGACGAGATGACGGAGCAGCAATTCCAGGTGCGCGCCATCGACGGAGAC 960

AMH-1_7 GTCCAGCAGTTTGACGAGATGACGGAACAGCAATTCCAGGTGCGCGCCATCGACGGAGAC 960

AMH-1_11 GTCCAGCAGTTTGACGAGATGACGGAGCAGCAATTCCAGGTGCGCGCCATCGACGGAGAC 960

KMP-8_3 GTCCAGCAGTTTGACGAGATGACGGAGCAGCAATTCCAGGTGCGCGCCATCGACGGAGAC 957

AMH-1_2 GTCCAGCAGTTTGACGAGATGACGGAGCAGCAATTCCAGGTGCGCGCCATCGACGGAGAC 960

AY198374.1 GTCCAGCAGTTTGACGAGATGACGGAGCAGCAATTCCAGGTGCGCGCCATCGACGGAGAC 960

* *****.******************.*****.************** ************

KMP-8_35 ACTGGCATCGGGAAAGCTATACACTATACCCTCGAGACAGATGAGGA---AGATTTGTTC 1017

KMP-8_5 ACGGGCATCGGGAAAGCTATACACTATGCCCTCGAGACAGATGAGGAAGAAGATTTGTTC 1017

KMP-8_24 ACTGGCATCGGGAAAGCTATACACTATACCCTCGAGACAGATGAGGAAGAAGATTTGTTC 1017

KMP-8_46 ACTGGCATCGGGAAAGCTATACACTATACCCTCGAGACAGATGAGGTAGAAGATTTGTTC 1017

AMH-3_16 ACTGGCATCGGGAAAGCTATACACTATACCCTCGAGACAGATGAGGAAGAAGATTTGTTC 1017

AMH-2_5 ACTGGCATCGGGAAAGCTATACACTATACCCTCGAGACAGATGAGGAAGAAGATTTGTTC 1020

AMH-2_1 ACTGGCATCGGGAAAGCTATACACTATACCCTCGAGACAGATGAGGAAGAAGATTTGTTC 1020

AMH-2_4 ACTGGCATCGGGAAAGCTATACACTATACCCTCGAGACAGATGAGGAAGAAGATTTGTTC 1020

AMH-3_1 ACTGGCATCGGGAAAGCTATACACTATACCCTCGAGACAGATGAGGAAGAAGATTTGTTC 1020

AMH-3_13 ACTGGCATCGGGAAAGCTATACACTATACCCTCGAGACAGATGAGGAAGAAGATTTGTTC 1020

AMH-1_7 ACTGGCATCGGGAAAGCTATACACTATACCCTCGAGACAGATGAGGAAGAAGATTTGTTC 1020

AMH-1_11 ACTGGCATCGGGAAAGCTATACACTATACCCTCGAGACAGATGAGGAAGAAGATTTGTTC 1020

KMP-8_3 ACTGGCATCGGGAAAGCTATACACTATACCCTCGAGACAGATGAGGAAGAAGATTTGTTC 1017

AMH-1_2 ACTGGCATCGGGAAAGCTATACACTATACCCTCGAGACAGATGAGGAAGAAGATTTGTTC 1020

AY198374.1 ACTGGCATCGGGAAAGCTATACACTATACCCTCGAGACAGATGAGGAAGAAGATTTGTTC 1020

** ************************.******************: **********

KMP-8_35 TTCATCGAAACACTTCCGGGCGGCCATGACGGAGCCATCTTCAGCACTGCCATGATTGAT 1077

KMP-8_5 TTCATCGAGACACTTCCGGGCGGCCATGACGGAGCCATCTTCAGCACTGCCATGATTGAT 1077

KMP-8_24 TTCATCGAAACACTTCCGGGCGGCCATGACGGAGCCATCTTCAGCACTGCCATGATTGAT 1077

KMP-8_46 TTCATCGAAACACTTCCGGGCGGCCATGACGGAGCCATCTTCAGCACTGCCATGATTGAT 1077

AMH-3_16 TTCATCGAAACACTTCCGGGCGGCCATGACGGAGCCATCTTCAGCACTGCCATGATTGAT 1077

AMH-2_5 TTCATCGAAACACTTCCGGGCGGCCATGACGGAGCCATCTTCGGCACTGCCATGATTGAT 1080

AMH-2_1 TTCATCGAAACACTTCCGGGCGGCCATGATGGAGCCATCTTCAGCACTGCCATGATTGAT 1080

AMH-2_4 TTCATCGAAACACTTCCGGGCGGCCATGACGGAGCCATCTTCAGCACTGCCATGATTGAT 1080

AMH-3_1 TTCATCGAAACACTTCCGGGCGGCCATGACGGAGCCATCTTCAGCACTGCCATGATTGAT 1080

AMH-3_13 TTCATCGAAACACTTCCGGGCGGCCATGACGGAGCCATCTTCAGCACTGCCATGATTGAT 1080

AMH-1_7 TTCATCGAAACACTTCCGGGCGGCCATGACGGAGCCATCTTCAGCACTGCCATGATTGAT 1080

AMH-1_11 TTCATCGAAACACTTCCGGGCGGCCATGACGGAGCCATCTTCAGCACTGCCATGATTGAT 1080

KMP-8_3 TTCATCGAAACACTTCCGGGCGGCCATGACGGAGCCATCTTCAGCACTGCCATGATTGAT 1077

AMH-1_2 TTCATCAAAACACTTCCGGGCGGCCATGACGGAGCCATCTTCAGCACTGCCATGATTGAT 1080

AY198374.1 TTCATCGAAACACTTCCGGGCGGCCATGACGGAGCCATCTTCAGCACTGCCATGATTGAT 1080

******.*.******************** ************.*****************

KMP-8_35 GTGGATAGGCTCCGGCGAGATGTCTTCAGACTGTCCCTGGTGGCATACAAGTACGACAAT 1137

KMP-8_5 GTGGATAGGCTCCGGCGAGATGTCTTCAGACTGTCCCTGGTGGCATACAAGTACGACAAC 1137

KMP-8_24 GTGGATAGGCTCCGGCGAGATGTCTTCAGACTGTCCCTGGTGGCATACAAGTACGACAAT 1137

KMP-8_46 GTGGATAGGCTCCGGCGAGATGTCTTCAGACTGTCCCTGGTGGCATACAAGTACGACAAT 1137

AMH-3_16 GTGGATAGGCTCCGGCGAGATGTCTTCAGACTGTCCCTGGTGGCATACAAGTACGACAAT 1137

AMH-2_5 GTGGATAGGTTCCGGCGAGATGTCTTCAGACTGTCCCTGGTGGCATACAAGTACGACAAT 1140

AMH-2_1 GTGGATAGGCTCCGGCGAGATGTCTTCAGACTGTCCCTGGTGGCATACAAGTACGACAAT 1140

AMH-2_4 GTGGATAGGCTCCGGCGAGATGTCTTCAGACTGTCCCTGGTGGCATACAAGTACGACAAT 1140

AMH-3_1 GTGGATAGGCTCCGGCGAGATGTCTTCAGACTGTCCCTGGTGGCATACAAGTACGACAAT 1140

AMH-3_13 GTGGATAGGCTCCGGCGAGATGTCTTCAGACTGTCCCTGGTGGCATACAAGTACGACAAT 1140

AMH-1_7 GTGGATAGGCTCCGGCGAGATGTCTTCAGACTGTCCCTGGTGGCATACAAGTACGACAAT 1140

AMH-1_11 GTGGATAGGCTCCGGCGAGATGTCTTCAGACTGTCCCTGGTGGCATACAAGTACGACAAT 1140

KMP-8_3 GTGGATAGGCTCCGGCGAGATGTCTTCAGACTGTCCCTGGTGGCATACAAGTACGACAAT 1137

AMH-1_2 GTGGATAGGCTCCGGCGAGATGTCTTCAGACTGTCCCTGGTGGCATACAAGTACGACAAT 1140

AY198374.1 GTGGATAGGCTCCGGCGAGATGTCTTCAGACTGTCCCTGGTGGCATACAAGTACGACAAT 1140

********* *************************************************

KMP-8_35 GTGTCCTTCGCCACCCCGACACCCGTCGTGATCATAGTTAACGACATCAACAACAAGAAA 1197

KMP-8_5 GTGTCCTTCGCCACCCCGACACCCGTCGTGATCATAGTCAACGACATCAACAACAAGAAA 1197

KMP-8_24 GTGTCCTTCGCCGCCCCGACACCCGTCGTGATCATAGTTAACGACATCAACAACAAGAAA 1197

KMP-8_46 GTGTCCTTCGCCACCCCGACACCCGTCGTGATCATAGTTAACGACATCAACAACAAGAAA 1197

AMH-3_16 GTGTCCTTCGCCACCCCGACACCCGTCGTGATCATAGTCAACGACATCAACAACAAGCAA 1197

AMH-2_5 GTGTCCTTCGCCACCCCGACACCCGTCGTGATCATAGTCAACGACATCAACAACAAGCAA 1200

AMH-2_1 GTGTCCTTCGCCACCCCGACACCCGTCGTGATCATAGTCAACGACATCAACAACAAGCAA 1200

AMH-2_4 GTGTCCTTCGCCACCCCAACACCCGTCGTGATCATAGTCAACGACATCAACAACAAGCAA 1200

AMH-3_1 GTGTCCTTCGCCACCCCGACACCCGTCGTGATCATAGTCAACGACATCAACAACAAGCAA 1200

AMH-3_13 GTGTCCTTCGCCACCCCGACACCCGTCGTGATCATAGTCAACGACATCAACAACAAGCAA 1200

AMH-1_7 GTGTCCTTCGCCACCCCGACACCCGTCGTGATCATAGTCAACGACATCAACAACAAGCAA 1200

AMH-1_11 GTGTCCTTCGCCACCCCGACACCCGTCGTGATCATAGTCAACGACATCAACAACAAGCAA 1200

KMP-8_3 GTGTCCTTCGCCACCCCGACACCCGTCGTGATCATAGTCAACGACATCAACAACAAGCAA 1197

AMH-1_2 GTGTCCTTCGCCACCCCGACACCCGTCGTGATCATAGTCAATGACATCAACAACAAGAAA 1200

AY198374.1 GTGTCCTTCGCCACCCCGACACCCGTCGTGATCATAGTCAATGACATCAACAACAAGAAA 1200

************.****.******************** ** ***************.**

KMP-8_35 CCCCAACCGCTGCAAGATGAGTACACAAACTCCATAATGGAAGAAACTCCACTGTCGCTG 1257

KMP-8_5 CCCCAACCGCTGCAAGATGAGTACACAATCTCCATAATGGAAGAGACTCCACTGTCGCTG 1257

KMP-8_24 CCCCAACCGCTGCAAGATGAGTACACAATCTCCATAATGGAAGAAACTCCACTGTCGCTG 1257

KMP-8_46 CCCCAACCGCTGCAAGATGAGTACACAATCTCCATAATGGAAGAAACTCCACTGTCGCTG 1257

AMH-3_16 CCCCAACCGCTGCAAGATGAGTACACAATCTCCATAATGGAAGAAACTCCACTGTCGCTG 1257

AMH-2_5 CCCCAACCGCTGCAAGATGAGTACACAATCTCCATAATGGAAGAAACTCCACTGTCGCTG 1260

AMH-2_1 CCCCAACCGCTGCAAGATGAGTACACAATCTCCATAATGGAAGAAACTCCACTGTCGCTG 1260

AMH-2_4 CCCCAACCGCTGCAAGATGAGTACACAATCTCCATAATGGAAGAAACTCCACTGTCGCTG 1260

AMH-3_1 CCCCAACCGCTGCAAGATGAGTACACAATCTCCATAATGGAAGAAACTCCACTGTCGCTG 1260

AMH-3_13 CCCCAACCGCCGCAAGATGAGTACACAATCTCCATAATGGAAGAAACTCCACTGTCGCTG 1260

AMH-1_7 CCCCAACCGCTGCAAGATGAGTACACAATCTCCATAATGGAAGAAACTCCACTGTCGCTG 1260

AMH-1_11 CCCCAACCGCTGCAAGATGAGTACACAATCTCCATAATGGAAGAAACTCCACTGTCGCTG 1260

KMP-8_3 CCCCAACCGCTGCAAGATGAGTACACAATCTCCATAATGGAAGAAACTCCACTGTCGCTG 1257

AMH-1_2 CCCCAACCGCTGCAAGATGAGTACACAATCTCCATAATGGAAGAAACTCCACTGTCGCTG 1260

AY198374.1 CCCCAACCGCTGCAAGATGAGTACACAATCTCCATAATGGAAGAAACTCCACTGTCGCTG 1260

********** *****************:***************.***************

KMP-8_35 AATTTTGCTGAACTTTTTGGTTTCTATGATGAAGATTTGATCTACGCACAATTCTTGGTG 1317

KMP-8_5 AATTTTGCTGAACTTTTTGGTTTCTATGATGAAGATTTGATCTACGCACAATTCTTGGTG 1317

KMP-8_24 AATTTTGCTGAACTTTTTAGTTTCTATGATGAAGATTTGATCTACGCACGATTCTTGGTG 1317

KMP-8_46 AATTTTGCTGAACTTTTTGGTTTCTATGATGAAGATTTGATCTACGCACAATTCTTGGTG 1317

AMH-3_16 AATTTTGCTGAACTTTTTGGTTTCTATGATGAAGATTTGATCTACGCACAATTCTTGGTG 1317

AMH-2_5 AATTTTGCTGAACTTTTTGGTTTCTATGTTGAAGATTTGATCTACGCACAATTCTTGGTG 1320

AMH-2_1 AATTTTGCTGAACTTTTTGGTTTCTATGATGAAGATTTGATCTACGCACAATTCTTGGTG 1320

AMH-2_4 AATTTTGCTGAACTTTTTGGTTTCTATGATGAAGATTTGATCTACGCGCAATTCTTGGTG 1320

AMH-3_1 AATTTTGCTGAACTTTTTGGTTTCTATGATGAAGATTTGATCTACGCACAATTCTTGGTG 1320

AMH-3_13 AATTTTGCTGAACTTTTTGGTTTCTATGATGAAGATTTGATCTACGCACAATTCTTGGTG 1320

AMH-1_7 AATTTTGCTGAACTTTTTGGTTTCTATGATGAAGATTTGATCTACGCACAATTCTTGGTG 1320

AMH-1_11 AATTTTGCTGAACTTTTTGGTTTCTATGATGAAGATTTGATCTACGCACAATTCTTGGTG 1320

KMP-8_3 AATTTTGCTGAACTTTTTGGTTTCTATGATGAAGATTTGATCTACGCACAATTCTTGGTG 1317

AMH-1_2 AATTTTGCTGAACTTTTTGGTTTCTATGATGAAGATTTGATCTACGCACAATTCTTGGTG 1320

AY198374.1 AATTTTGCTGAACTTTTTGGTTTCTATGATGAAGATTTGATCTACGCACAATTCTTGGTG 1320

******************.*********:******************.*.**********

KMP-8_35 GAAATACAAGGCGAGAACCCTCCAGGCGTAGAGCAAGCGTTTTATATTGCGCCCACCGCA 1377

KMP-8_5 GAAATACAAGGCGAGAACCCTCCAGGCGTAGAGCAAGCGTTTTATATTGCGCCCACCGCA 1377

KMP-8_24 GGAATACAAGGCGAGAACCCTCCAGGCGTAGAGCAAGCGTTTTATATTGCGCCCACCGCA 1377

KMP-8_46 GAAATACAAGGCGAGGACCCTCCAGGCGTAGAGCAAGCGTTTTATATTGCGCCCACCGCA 1377

AMH-3_16 GAAATACAAGGCGAGAACCCTCCAGGCGTAGAGCAAGCGTTTTATATTGCGCCCACCGCA 1377

AMH-2_5 GAAATACAAGGCGAGAACCCTCCAGGCGTAGAGCAAGCGTTTTATATTGCGCCCACCGCA 1380

AMH-2_1 GAAATACAAGGCGAGAACCCTCCAGGCGTAGAGCAAGCGTTTTATATTGCGCCCACCGCA 1380

AMH-2_4 GAAATACAAGGCGAGAACCCTCCAGGCGTAGAGCAAGCGTTTTATATTGCGCCCACCGCA 1380

AMH-3_1 GAAATACAAGGCGAGAACCCTCCAGGCGTAGAGCAAGCGTTTTATATTGCGCCCACCGCA 1380

AMH-3_13 GGAATACAAGGCGAGAACCCTCCAGGCGTAGAGCAAGCGTTTTATATTGCGCCCACCGCA 1380

AMH-1_7 GAAATACAAGGCGAGAACCCTCCAGGCGTAGAGCAAGCGTTTTATATTGCGCCCACCGCA 1380

AMH-1_11 GAAATACAAGGCGAGAACCCTCCAGGCGTAGAGCAAGCGTTTTATATTGCGCCCACCGCA 1380

KMP-8_3 GAAATACAAGGCGAGAACCCTCCAGGCGTAGAGCAAGCGTTTTATATTGCGCCCACCGCA 1377

AMH-1_2 GAAATACAAGGCGAGAACCCTCCAGGCGTAGAGCAAGCGTTTTATATTGCGCCCACCGCA 1380

AY198374.1 GAAATACAAGGCGAGAACCCTCCAGGCGTAGAGCAAGCGTTTTATATTGCGCCCACCGCA 1380

*.*************.********************************************

KMP-8_35 GGCTTCCAGAACCAGACATTCGCCATAGGGACTCAAGATCACCGAATGCTGGATTATGAG 1437

KMP-8_5 GGCTTCCAGAACCAGACATTCGCCATAGGGACTCAAGATCACCGAATGCTGGATTATGAG 1437

KMP-8_24 GGCTTCCAGAACCAGACATTCGCCATAGGGACTCAAGATCACCGAATGCTGGATTATGAG 1437

KMP-8_46 GGCTTCCAGAACCAGACATTCGCCATAGGGACTCAAGATCACCGAATGCTGGATTATGAG 1437

AMH-3_16 GGCTTCCAGAACCAGACATTCGCCATAGGGACTCAAGATCACCGAATGCTGGATTATGAG 1437

AMH-2_5 GGCTTCCAGAACCAGACATTCGCCATAGGGACTCAAGATCACCGAATGCTGGATTATGAG 1440

AMH-2_1 GGCTTCCAGAACCAGACATTCGCCATAGGGACTCAAGATCACCGAATGCTGGATTATGAG 1440

AMH-2_4 GGCTTCCAGAACCAGACATTCGCCATAGGGACTCAAGATCACCGAATGCTGGATTATGAG 1440

AMH-3_1 GGCTTCCAGAACCAGACATTCGCCATAGGGACTCAAGATCACCGAATGCTGGATTATGAG 1440

AMH-3_13 GGCTTCCAGAACCAGACATTCGCCATAGGGACTCAAGATCACCGAATGCTGGATTATGAG 1440

AMH-1_7 GGCTTCCAGAACCAGACATTCGCCATAGGGACTCAAGATCACCGAATGCTGGATTATGAG 1440

AMH-1_11 GGCTTCCAGAACCAGACATTCGCCATAGGGACTCAAGATCACCGAATGCTGGATTATGAG 1440

KMP-8_3 GGCTTCCAGAACCAGACATTCGCCATAGGGACTCAAGATCACCGAATGCTGGATTATGAG 1437

AMH-1_2 GGCTTCCAGAACCAGACATTCGCCATAGGGACTCAAGATCACCGAATGCTGGATTATGAG 1440

AY198374.1 GGCTTCCAGAACCAGACATTCGCCATAGGGACTCAAGATCACCGAATGCTGGATTATGAG 1440

************************************************************

KMP-8_35 GATGTTCCTTTCCAAAACATCAAGCTCAAGGTAATAGCAACGGACCGTGACAATACCAAT 1497

KMP-8_5 GATGTTCCTTTCCAAAACATCAAGCTCAAGGTAATAGCAACGGACCGTGACAATACCAAT 1497

KMP-8_24 GATGTTCCTTTCCAAAACATCAAGCTCAAGGTAATAGCAACGGACCGTGACAATACCAAT 1497

KMP-8_46 GATGTTCCTTTCCAAAACATCAAGCTCAAGGTAATAGCAACGGACCGTGACAATACCAAT 1497

AMH-3_16 GATGTTCCTTTCCAAAACATCAAGCTCAAGGTAATAGCAACGGACCGTGACAATACCAAT 1497

AMH-2_5 GATGTTCCCTTCCAAAACATCAAGCTCAAGGTAATAGCAACGGACCGTGACAATACCAAT 1500

AMH-2_1 GATGTTCCTTTCCAAAACATCAAGCTCAAGGTAATAGCAACGGACCGTGACAATACCAAT 1500

AMH-2_4 GATGTTCCTTTCCAAAACATCAAGCTCAAGGTAATAGCAACGGACCGTGACAATACCAAT 1500

AMH-3_1 GATGTTCCTTTCCAAAACATCAAGCTCAAGGTAATAGCAACGGACCGTGACAATACCAAT 1500

AMH-3_13 GATGTTCCTTTCCAAAACATCAAGCTCAAGGTAATAGCAACGGACCGTGACAATACCAAT 1500

AMH-1_7 GATGTTCCTTTCCAAAACATCAAGCTCAAGGTAATAGCAACGGACCGTGACAATACCAAT 1500

AMH-1_11 GATGTTCCTTTCCAAAACATCAAGCTCAAGGTAATAGCAACGGACCGTGACAATACCAAT 1500

KMP-8_3 GATGTTCCTTTCCAAAACATCAAGCTCAAGGTAATAGCAACGGACCGTGACAATACCAAT 1497

AMH-1_2 GATGTTCCTTTCCAAAACATCAAGCTCAAGGTAATAGCAACGGACCGTGACAATACCAAT 1500

AY198374.1 GATGTTCCTTTCCAAAACATCAAGCTCAAGGTAATAGCAACGGACCGTGACAATACCAAT 1500

******** ***************************************************

KMP-8_35 TTTACTGGAGTCGCGGAAGTCAACGTGAATCTGATTAATTGGAACGACGAGGAGCCGATC 1557

KMP-8_5 TTTACTGGAGTCGCGGAAGTCAACGTGAATCTGATTAATTGGAACGACGAGGAGCCGATC 1557

KMP-8_24 TTTACTGGAGTCGCGGAAGTCAACGTGAATCTGATTAATTGGAACGACGAGGAGCCGATC 1557

KMP-8_46 TTTACTGGAGTCGCGGAAGTCAACGTGAATCTGATTAATTGGAACGACGAGGAGCCGATC 1557

AMH-3_16 TTTACTGGAGTCGCGGAAGTCAACGTGAACCTGATTAATTGGAACGACGAGGAGCCGATC 1557

AMH-2_5 TTCACTGGAGTGGCGGAAGTCAACGTGAATCTGATTAATTGGAACGACGAGGAGCCGATC 1560

AMH-2_1 TTCACTGGAGTGGCGGAAGTCAACGTGAATCTGATTAATTGGAACGACGAGGAGCCGATC 1560

AMH-2_4 TTCACTGGAGTGGCGGAAGTCAACGTGAATCTGATTAATTGGAACGACGAGGAGCCGATC 1560

AMH-3_1 TTTACTGGAGTCGCGGAAGTCAACGTGAACCTGATTAATTGGAACGACGAGGAGCCGATC 1560

AMH-3_13 TTTACTGGAGTCGCGGAAGTCAACGTGAACCTGATTAATTGGAACGACGAGGAGCCGATC 1560

AMH-1_7 CTTACTGGAGTCGCGGAAGTCAACGTGAACCTGATTAATTGGGACGACGAGGAGCCGATC 1560

AMH-1_11 TTTACTGGAGTCGCGGAAGTCAACGTGAACCTGATTAATTGGAACGACGAGGAGCCGATC 1560

KMP-8_3 TTTACTGGAGTCGCGGAAGCCAACGTGATTCTGATTAATTGGAACGACGAGGAGCCGATC 1557

AMH-1_2 TTTACTGGAGTCGCGGAAGTCAACGTGAACCTGATTAATTGGAACGACGAGGAGCCGATC 1560

AY198374.1 TTTACTGGAGTCGCGGAAGTCAACGTGAACCTGATTAATTGGAACGACGAGGAGCCGATC 1560

* ******** ******* ********: ************.*****************

KMP-8_35 TTTGAGGAGGACCAGCTGGTTGTCGAGTTCAAGGAGACTGTACCCAAGGACTATCACGTC 1617

KMP-8_5 TTTGAGGAGGACCAGCTGGTTGTCAAGTTCAAGGAGACTGTACCCAAGGACTATCACGTC 1617

KMP-8_24 TTTGAGGAGGACCAGCTGGTTGTCAAGTTCAAGGAGACTGTACCCAAGGACTATCACGTC 1617

KMP-8_46 TTTGAGGAGGACCAGCTGGTTGTCAAGTTCAAGGAGACTGTACCCAAGGACTATCACGTC 1617

AMH-3_16 TTTGAGGAAGACCAGCTCGTTGTCAAGTTCAAGGAGACTGTACCCAAGGACTATCACGTC 1617

AMH-2_5 TTTGAGGAGGACCAGCTGGTTGTCAAGCTCAAGGAGACTGTACCCAAGGACTATCACGTC 1620

AMH-2_1 TTTGAGGAGGACCAGCTGGTTGTCAAGTTCAAGGAGACTGTACCCAAGGACTATCACGTC 1620

AMH-2_4 TTTGAGGAGGACCAGCTGGTTGTCAAGTTCAAGGAGACTGTACCCAAGGACTATCACGTC 1620

AMH-3_1 TTTGAGGAAGACCAGCTCGTTGTCAAGTTCAAGGAGACTGTACCCAAGGACTATCACGTC 1620

AMH-3_13 TTTGAGGAAGACCAGCTCGTTGTCAAGTTCAAGGAGACTGTACCCAAGGACTATCACGTC 1620

AMH-1_7 TTTGAGGAAGACCAGCTCGTTGTCAAGTTCAAGGAGACTGTACCCAAGGACTATCACGTC 1620

AMH-1_11 TTTGAGGAAGACCAGCTCGTTGTCAAGTTCAAGGAGACTGTACCTAAGGACTATCACGTC 1620

KMP-8_3 TTTGAGGAGGACCAGCTGGTTGTCAAGTTCAAGGAGACTGTACCCAAGGACTATCACGTC 1617

AMH-1_2 TTTGAGGAAGACCAGCTCGTTGTCAAGTTCAAGGAGACTGTACCCAAGGACTATCACGTC 1620

AY198374.1 TTTGAGGAAGACCAGCTCGTTGTCAAGTTCAAGGAGACTGTACCCAAGGACTATCACGTC 1620

********.******** ******.** **************** ***************

KMP-8_35 GGCAGACTGAGGGCTCACGACCGGGACATAGGAGACAGCGTTGTGCATTCTATCTTGGGA 1677

KMP-8_5 GGCAGACTGAGGGCTCACGACCGGGACATAGGAGACAGCGTTGTGCATTCCATCTTGGGA 1677

KMP-8_24 GGCAGACTGAGGGCTCACGACCGGGACATAGGAGACAGCGTTGTGCATTCCATCTTGGGA 1677

KMP-8_46 GGCAGACTGAGGGCTCACGACCGGGACATAGGAGACAGCGTTGTGCATTCCATCTTGGGA 1677

AMH-3_16 GGCAGACTGAGGGCTCACGACCGGGACATAGGAGACAGCGTTGTGCATTCCATCTTGGGA 1677

AMH-2_5 GGCAGACTGAGGGCTCACGACCGGGACATAGGAGACAGCGTTGTGCATTCCATCTTGGGA 1680

AMH-2_1 GGCAGACTGAGGGCTCACGACCGGGACATAGGAGACAGCGTTGTGCATTCCATCTTGGGA 1680

AMH-2_4 GGCAGACTGAGGGCTCACGACCGGGACATAGGAGACAGCGTTGTGCATTCCATCTTGGGA 1680

AMH-3_1 GGCAGACTGAGGGCTCACGACCGGGACATAGGAGACAGCGTTGTGCATTCCATCTTGGGA 1680

AMH-3_13 GGCAGACTGAGGGCTCACGACCGGGACATAGGAGACAGCGTTGTGCATTCCATCTTGGGA 1680

AMH-1_7 GGCAGACTGAGGGCTCACGACCGGGACATAGGAGACAGCGTTGTGCATTCCATCTTGGGA 1680

AMH-1_11 GGCAGACTGAGGGCTCACGACCGGGACATAGGAGACAGCGTTGTGCATTCCATCTTGGGA 1680

KMP-8_3 GGCAGACTGAGGGCTCACGACCGGGACATAGGAGACAGCGTTGTGCATTCCATCTTGGGA 1677

AMH-1_2 GGCAGACTGAGGGCTCACGACCGGGACATAGGAGACAGCGTTGTGCATTCCATCTTGGGA 1680

AY198374.1 GGCAGACTGAGGGCTCACGACCGGGACATAGGAGACAGCGTTGTGCATTCCATCTTGGGA 1680

************************************************** *********

KMP-8_35 AATGCGAATACATTTTTGAGAATCGACGAAGAAACTGGCGACATCTACGTAGCTATTGAT 1737

KMP-8_5 AATGCGAATACATTTTTGAGAATCGACGAAGAAACTGGCGACATCTACGTAGCTATTGAT 1737

KMP-8_24 AATGCGAATACATTTTTGAGAATCGACGAAGAAACTGGCGACATCTACGTAGCTATTGAT 1737

KMP-8_46 AATGCGAATACATTTTTGAGAATCGACGAAGAAACTGGCGACATCTACGTAGCTATTGAT 1737

AMH-3_16 AATGCGAATACATTTTTAAGAATCGACGAAGAAACTGGCGACATCTACGTAACTATTGAT 1737

AMH-2_5 AATGCGAATACATTTTTGAGAATCGACGAAGAAACTGGCGACATCTACGTAGCTATTGAT 1740

AMH-2_1 AATGCGAATACATTTTTGAGAATCGACGAAGAAACTGGCGACATCTACGTAGCTATTGAT 1740

AMH-2_4 AATGCGAATACATTTTTGAGAATCGACGAAGAAACTGGCGACATCTACGTAGCTATTGAT 1740

AMH-3_1 AATGCGAATACATTTTTAAGAATCGACGAAGAAACTGGCGACATCTACGTAACTATTGAT 1740

AMH-3_13 AATGCGAATACATTTTTAAGAATCGACGAAGAAACTGGCGACATCTACGTAACTATTGAT 1740

AMH-1_7 AATGCGAATACATTTTTAAGAATCGACGAAGAAACTGGCGACATCTACGTAACTATTGAT 1740

AMH-1_11 AATGCGAATACATTTTTAAGAATCGACGAAGAAACTGGCGACATCTACGTAACTATTGAT 1740

KMP-8_3 AATGCGAATACATTTTTGAGAATCGACGAAGAAACTGGCGACATCTACGTAGCTATTGAT 1737

AMH-1_2 AATGCGAATACATTTTTGAGAATCGACGAAGAAACTGGCGACATATACGTAGCTATTGAT 1740

AY198374.1 AATGCGAATACATTTTTGAGAATCGACGAAGAAACTGGCGACATATACGTAGCTATTGAT 1740

*****************.**************************.******.********

KMP-8_35 GACGCGTGCGATTATCACAGACAGAATGAATTTAACATACAAGTTCGCGCTCAGGACACC 1797

KMP-8_5 GACGCGTTCGATTATCACAGACAGAATGAATTTAACATACAAGTTCGTGCTCAGGACACC 1797

KMP-8_24 GACGCGTTCGATTATCACAGACAGAATGAATTTAACATACAAGTTCGCGCTCAGGACACC 1797

KMP-8_46 GACGCGTTCGATTATCACAGACAGAATGAATTTAACATACAAGTTCGCGCTCAGGACACC 1797

AMH-3_16 GACGCGTTCGATTATCACAGACAGAATGAATTTAACATACAAGTTCGCGCTCAGGACACC 1797

AMH-2_5 GACGCGTTCGATTATCACAGACAGAATGAATTTAACATACAAGTTCGCGCTCAGGACACC 1800

AMH-2_1 GACGCGTTCGATTATCACAGACAGAATGAATTTAACATACAAGTTCGCGCTCAGGACACC 1800

AMH-2_4 GACGCGTTCGATTATCACAGACAGAATGAATTTAACATACAAGTTCGCGCTCAGGACACC 1800

AMH-3_1 GACGCGTTCGATTATCACAGACAGAATGAATTTAACATACAAGTTCGCGCTCAGGACACC 1800

AMH-3_13 GACGCGTTCGATTATCACAGACAGAATGAATTTAACATACAAGTTCGCGCTCAGGACACC 1800

AMH-1_7 GACGCGTTCGATTATCACAGACAGAATGAATTTAACATACAAGTTCGCGCTCAGGACACC 1800

AMH-1_11 GACGCGTTCGATTATCACAGACAGAATGAATTTAACATACAAGTTCGCGCTCAGGACACC 1800

KMP-8_3 GACGCGTTCGATTATCACAGACAGAATGAATTTAACATACAAGTTCGCGCTCAGGACACC 1797

AMH-1_2 GACGCGTTCGATTATCACAGACAGAATGAATTTAACATACAAGTTCGCGCTCAGGACACC 1800

AY198374.1 GACGCGTTCGATTATCACAGACAGAATGAATTTAACATACAAGTTCGCGCTCAGGACACC 1800

******* *************************************** ************

KMP-8_35 ATGTCGGAGCCAGAGTCCAGGCATACAGCGACTGCTCAGCTGGTCATAGAACTCGAGGAC 1857

KMP-8_5 ATGTCGGAGCCAGAGTCCAGGCATACAGCGACTGCTCAGCTGGTCATAGAACTCGGGGAC 1857

KMP-8_24 ATGTCGGAGCCAGAGTCCAGGCATACAGCGACTGCTCAGCTGGTCATAGAACTCGAGGAC 1857

KMP-8_46 ATGTCGGAGCCAGAGTCCAGGCATACAGCGACTGCTCAGCTGGTCATAGAACTCGAGGAC 1857

AMH-3_16 ATGTCGGAGCCAGAGTCCAGGCATACAGCGACTGCTCAGCTGGTCATAGAACTCGAGGAC 1857

AMH-2_5 ATGTCGGAGCCAGAGTCCAGGCATACAGCGACTGCTCAGCTGGTCATAGAACTCGAGGAC 1860

AMH-2_1 ATGTCGGAGCCAGAGTCCAGGCATACAGCGACTGCTCAGCTGGTCATAGAACTCGAGGAC 1860

AMH-2_4 ATGTCGGAGCCAGAGTCCAGGCATACAGCGACTGCTCAGCTGGTCATAGAACTCGAGGAC 1860

AMH-3_1 ATGTCGGAGCCAGAGTCCAGGCATACAGCGACTGCTCAGCTGGTCATAGAACTCGAGGAC 1860

AMH-3_13 ATGTCGGAGCCAGAGTCCAGGCATACAGCGACTGCTCAGCTGGTCATAGAACTCGAGGAC 1860

AMH-1_7 ATGTCGGAGCCAGAGTCCAGGCATACAGCGACTGCTCAGCTGGTCATAGAACTCGAGGAC 1860

AMH-1_11 ATGTCGGAGCCAGAGTCCAGGCATACAGCGACTGCTCAGCTGGTCATAGAACTCGAGGAC 1860

KMP-8_3 ATGTCGGAGCCAGAGTCCAGGCATACAGCGACTGCTCAGCTGGTCATAGAACTCGAGGAC 1857

AMH-1_2 ATGTCGGAGCCAGAGTCCAGGCATACAGCGACTGCTCAGCTGGTCATAGAACTCGAGGAC 1860

AY198374.1 ATGTCGGAGCCAGAGTCCAGGCATACAGCGACTGCTCAGCTGGTCATAGAACTCGAGGAC 1860

*******************************************************.****

KMP-8_35 GTCAACAACACACCTCCTACTCTGAGGCTGCCTCGCGTAAGTCCGTCTGTAGAAGAGAAT 1917

KMP-8_5 GTCAACAACACACCTCCTACTCTGAGGCTGCCTCGCGTAAGTCCGTCTGTAGAAGAGAAT 1917

KMP-8_24 GTCAACAACACACCTCCTACTCTGAGGCTGCCTCGCGTAAGTCCGTCTGTAGAAGAGAAT 1917

KMP-8_46 GTCAACAACACACCTCCTACTCTGAGGCTGCCTCGCGTAAGTCCGTCTGTAGAAGAGAAT 1917

AMH-3_16 GTCAACAACACACCTCCTACTCTGAGGCTGCCGCGCGTAAGTCCGTCTGTAGAAGAGAAT 1917

AMH-2_5 GTCAACAACACACCTCCCACTCTGAGGCTGCCTCGCGTAAGTCCGTCTGTAGAAGAGAAT 1920

AMH-2_1 GTCAACAACACACCTCCCACTCTGAGGCTGCCTCGCGTAAGTCCGTCTGTAGAAGAGAAT 1920

AMH-2_4 GTCAACAACACACCTCCCACTCTGAGGCCGCCTCGCGTAAGTCCGTCTGTAGAAGAGAAT 1920

AMH-3_1 GTCAACAACACACCTCCTACTCTGAGGCTGCCGCGCGTAAGTCCGTCTGTAGAAGAGAAT 1920

AMH-3_13 GTCAACAACACACCTCCTACTCTGAGGCTGCCGCGCGTAAGTCCGTCTGTAGAAGAGAAT 1920

AMH-1_7 GTCAACAACACACCTCCTACTCTGAGGCTGCCGCGCGTAAGTCCGTCTGTAGAAGAGAAT 1920

AMH-1_11 GTCAACAACACACCTCCTACTCTGAGGCTGCCGCGCGTAAGTCCGTTTGTAGAAGAGAAT 1920

KMP-8_3 GTCAACAACACACCTCCTACTCTGAGGCTGCCTCGCGTAAGTCCGTCTGTAGAAGAGAAT 1917

AMH-1_2 GTCAACAACACACCTCCTACTCTGAGGCTGCCTCGCGTAAGTCCGTCTGTAGAAGAGAAT 1920

AY198374.1 GTCAACAACACACCTCCTACTCTGAGGCTGCCTCGCGTAAGTCCGTCTGTAGAAGAGAAT 1920

***************** ********** *** ************* *************

KMP-8_35 GTGCCAGAGGGCTTTGGAGTCAACCGGGAGATAACCGCCACGGACCCTGACACCACAGCA 1977

KMP-8_5 GTGCCAGAGGGCTTTGAAGTCAACCGGGAGATAACCGCCACGGACCCTGACACCACAGCA 1977

KMP-8_24 GTGCCAGAGGGCTTTGAAGTCAACCGGGAGATAACCGCCACGGACCCTGACACCACAGCA 1977

KMP-8_46 GTGCCAGAGGGCTTTGAAGTCAACCGGGAGATAACCGCCACGGACCCTGACACCACAGCA 1977

AMH-3_16 GTGCCAGAGGGCTTTGAAATCAACCGGGAGATAACCGCCACGGACCCTGACACCACAGCA 1977

AMH-2_5 GTGCCAGAGGGCTTTGAAATCAACCGGGAGATAACCGCCACGGACCCTGACACCACAGCA 1980

AMH-2_1 GTGCCAGAGGGCTTTGAAATCAACCGGGAGATAACCGCCACGGACCCAGACACCACAGCA 1980

AMH-2_4 GTGCCAGAGGGCTTTGAAATCAACCGGGAGATAACCGCCACGGACCCTGACACCACAGCA 1980

AMH-3_1 GTGCCAGAGGGCTTTGAAATCAACCGGGAGATAACCGCCACGGACCCTGACACCACAGCA 1980

AMH-3_13 GTGCCAGAGGGCTTTGAAATCAACCGGGAGATAACCGCCACGGACCCTGACACCGCAGCA 1980

AMH-1_7 GTGCCAGAGGGCTTTGAAATCAACCGGGAGATAACCGCCACGGACCCTGACACCACAGCA 1980

AMH-1_11 GTGCCAGAGGGCTTTGAAATCAACCGGGAGATAACCGCCACGGACCCTGACACCACAGCA 1980

KMP-8_3 GGGCCAGAGGGCTTTGAAGTCAACCGGGAGATAACCGCCACGGACCCTGACACCACAGCA 1977

AMH-1_2 GTGCCAGAGGGCTTTGAAATCAACCGGGAGATAACCGCCACGGACCCTGACACCACAGCA 1980

AY198374.1 GTGCCAGAGGGCTTTGAAATCAACCGGGAGATAACCGCCACGGACCCTGACACCACAGCA 1980

* **************.*.****************************:******.*****

KMP-8_35 TACTTGCAGTTTGAAATAGATTGGGACACATCCTTTGCCACTAAACAGGGGCGTGATACC 2037

KMP-8_5 TACCTGCAGTTTGAAATAGATTGGGACACATCCTTTGCCACTAAACAGGGGCGTGATACC 2037

KMP-8_24 TACCTGCAGTTTGAAATAGATTGGGACACATCCTTTGCCACTAAACAGGGGCGTGATACC 2037

KMP-8_46 TACCTGCAGTTTGAAATAGATTGGGACACATCCTTTGCCACTAAACAGGGGCGTGATACC 2037

AMH-3_16 TACCTGCAGTTTGAAATAGATTGGGACACATCCTTTGCCACTAAACAGGGGCGTGATACC 2037

AMH-2_5 TACCTGCAGTTTGAAATAGATTGGGACACATCCTTTGCCACTAAACAGGGGCGTGATACC 2040

AMH-2_1 TACCTGCAGTTTGAAATAGATTGGGACACATCCTTTGCCACTAAACAGGGGCGTGATACC 2040

AMH-2_4 TACCTGCAGTTTGAAATAGATTGGGACACATCCTTTGCCACTAAACAGGGGCGTGATACC 2040

AMH-3_1 TACCTGCAGTTTGAAATAGATTGGGACACATCCTTTGCCACTAAACAGGGGCGTGATACC 2040

AMH-3_13 TACCTGCAGTTTGAAATAGATTGGGACACATCCTTTGCCACTAAACAGGGGCGTGATACC 2040

AMH-1_7 TACCTGCAGTTTGAAATAGATTGGGACACATCCTTTGCCACTAAACAGGGGCGTGATACC 2040

AMH-1_11 TACCTGCAGTTTGAAATAGATTGGGACACATCCTTTGCCACTAAACAGGGGCGTGATACC 2040

KMP-8_3 TACCTGCAGTTTGAAATAGATTGGGACACATCCTTTGCCACTAAACAGGGGCGTGATACC 2037

AMH-1_2 TACCTGCAGTTTGAAATAGATTGGGACACATCCTTTGCCACTAAACAGGGGCGTGATACC 2040

AY198374.1 TACCTGCAGTTTGAAATAGATTGGGACACATCCTTTGCCACTAAACAGGGGCGTGATACC 2040

*** ********************************************************

KMP-8_35 AATCCAATAGAGTTCCACGGATGCGTGGATATAGAAACCATCTTCCCAAACCCAGCCGAC 2097

KMP-8_5 AATCCAATAGAGTTCCACGGATGCGTGGATATAGAAACCATCTTCCCAAACCCAGCCGAC 2097

KMP-8_24 AATCCAATAGAGTTCCACGGATGCGTGGATATAGAAACCATCTTCCCAAACCCAGCCGAC 2097

KMP-8_46 AATCCAATAGAGTTCCACGGATGCGTGGATATAGAAACCATCTTCCCAAACCCAGCCGAC 2097

AMH-3_16 AATCCAATAGAGTTCCACGGATGCGTGGATATAGAAACCATCTTCCCAAACCCAGCCGAC 2097

AMH-2_5 AATCCAATAGAGTTCCACGGATGCGTGGATATAGAAACCATCTTCCCAAACCCAGCCGAC 2100

AMH-2_1 AATCCAATAGAGTTCCACGAATGCGTGGATATAGAAACCATCTTCCCAAACCCAGCCGAC 2100

AMH-2_4 AATCCAATAGAGTTCCACGGATGCGTGGATATAGAAACCATTTTCCCAAACCCAGCCGAC 2100

AMH-3_1 AATCCAATAGAGTTCCACGGATGCGTGGATATAGAAACCATCTTCCCAAACCCAGCCGAC 2100

AMH-3_13 AATCCAATAGAGTTCCACGGATGCGTGGATATAGAAACCATCTTCCCAAACCCAGCCGAC 2100

AMH-1_7 AATCCAGTAGAGTTCCACGGATGCGTGGATATAGAAACCATCTTCCCAAACCCAGCCGAC 2100

AMH-1_11 AATCCAGTAGAGTTCCACGGATGCGTGGATATAGAAACCATCTTCCCAAACCCAGCCGAC 2100

KMP-8_3 AATCCAATAGAGTTCCACGGATGCGTGGATATAGAAACCATCTTCCCAAACCCAGCCGAC 2097

AMH-1_2 AATCCAATAGAGTTCCACGGATGCGTGGATATAGAAACCATCTTCCCAAACCCAGCCGAC 2100

AY198374.1 AATCCAATAGAGTTCCACGGATGCGTGGATATAGAAACCATCTTCCCAAACCCAGCCGAC 2100

******.************.********************* ******************

KMP-8_35 ACCAGAGAGGCTGTGGGGCGAGTGGTAGCGAAGGAGATCCGCCATAACGTGACCATCGAT 2157

KMP-8_5 ACCAGAGAGGCTGTGGGGCGAGTGGTAGCGAAGGAGATCCGCCATAACGTGACCATCGAT 2157

KMP-8_24 ATCAGAGAGGCTGTGGGGCGAGTGGTAGCGAAGGAGATCCGCCATAACGTGACCATCGAT 2157

KMP-8_46 ACCAGAGAGGCTGTGGGGCGAGTGGTAGCGAAGGAGATCCGCCATAACGTGACCATCGAT 2157

AMH-3_16 ACCAGAGAAGCTGTGGGGCGAGTGGTAGCGAAGGAGATCCGCCATAACGTGACCATCGAT 2157

AMH-2_5 ACCGGAGAGGCTGTGGGGCGAGTGGTAGCGAAGGAGATCCGCCATAACGTGACCATCGAT 2160

AMH-2_1 ACCAGAGAGGCTGTGGGGCGAGTGGTAGCGAAGGAGATCCGCCATAACGTGACCATCGAT 2160

AMH-2_4 ACCAGAGAGGCTGTGGGGCGAGTGGTAGCGAAGGAGATCCGCCATAACGTGACCATCGAT 2160

AMH-3_1 ACCAGAGAAGCTGTGGGGCGAGTGGTAGCGAAGGAGATCCGCCATAACGTGACCATCGAT 2160

AMH-3_13 ACCGGAGAAGCTGTGGGGCGAGTGGTAGCGAAGGAGATCCGCCATAACGTGACCATCGAT 2160

AMH-1_7 ACCAGAGAAGCTGTGGGGCGAGTGGTAGCGAAGGAGATCCGCCATAACGTGACCATCGAT 2160

AMH-1_11 ACCAGAGAAGCTGTGGGGCGAGTGGTAGCGAAGGAGATCCGCCATAACGTGACCATCGAT 2160

KMP-8_3 ACCAGAGAGGCTGTGGGGCGAGTGGTAGCGAAGGAGATCCGCCATAACGTGACCATCGAT 2157

AMH-1_2 ACCAGAGAGGCTGTGGGGCGAGTGGTAGCGAAGGAGATCCGCCATAACGTGACCATCGAT 2160

AY198374.1 ACCAGAGAGGCTGTGGGGCGAGTGGTAGCGAAGGAGATCCGCCATAACGTGACCATCGAT 2160

* *.****.***************************************************

KMP-8_35 TTTGAAGAGTTTGAATTTCTCTACCTCACAGTGAGAGTTCGGGACTTGCACACAGATGAC 2217

KMP-8_5 TTTGAAGAGTTTGAATTTCTCTACCTCACAGTGAGAGTTCGGGACTTGCACACAGATGAC 2217

KMP-8_24 TTTGAAGAGTTTGAATTTCTCTACCTCACAGTGAGAGTTCGGGACTTGCACACAGATGAC 2217

KMP-8_46 TTTGAAGAGTTTGAATTTCTCTACCTCACAGTGAGAGTTCGGGACTTGCACACGGATGAC 2217

AMH-3_16 TTTGAAGAGTTTGAATTTCTCTACCTCACAGTGAGAGTTCGAGACTTGCACACAGAAGAT 2217

AMH-2_5 TTTGAAGAGTTTGAATTTCGCTACCTCACAGTGAGAGTTCGGGACTTGCACACAGATGAC 2220

AMH-2_1 TTTGAAGAGTTTGAATTTCTCTACCTCACAGTGAGAGTTCGGGACTTGCACACAGATGAC 2220

AMH-2_4 TTCGAAGAGTTTGAATCTCTCTACCTCACAGTGAGAGTTCGGGACTTGCACACAGATGAC 2220

AMH-3_1 TTTGAAGAGTTTGAACTTCTCTACCTCACAGTGAGAGTTCGAGACTTGCACACAGAAGAT 2220

AMH-3_13 TTTGAAGAGTTTGAATTTCTCTACCTCACAGTGAGAGTTCGAGACTTGCACACAGAAGAT 2220

AMH-1_7 TTTGAAGAGTTTGAATTTCTGTACCTCACAGTGAGAGTTCGAGACTTGCACACAGAAGAT 2220

AMH-1_11 TTCGAAGAGTTTGAATTTCTGTACCTCACAGTGAGAGTTCGAGACTTGCACACAGAAGAT 2220

KMP-8_3 TTTGAAGAGTTTGAATTTCTCTACCTCACAGTGAGAGTTCGGGACTTGCACACAGATGAC 2217

AMH-1_2 TTTGAAGAGTTTGAATTTCTCTACCTCACAGTGAGAGTTCGGGACTTGCACACAGATGAC 2220

AY198374.1 TTTGAAGAGTTTGAATTTCTCTACCTCACAGTGAGAGTTCGGGACTTGCACACAGATGAC 2220

** ************ ** ********************.***********.**:**

KMP-8_35 GGACGAGATTATGATGAATCTACCTTCACGATAATAATAATAGATATGAACGACAACTGG 2277

KMP-8_5 GGACGAGATTATGATGAATCTACCTTCACGATAATAATAATAGATATGAACGACAACTGG 2277

KMP-8_24 GGACGAGATTATGATGAATCTACCTTCACGATAATAATAATAGATATGAACGACAACTGG 2277

KMP-8_46 GGACGAGATTATGATGAATCTACCTTCACGATAATAATAATAGATATGAACGACAACTGG 2277

AMH-3_16 GGACGAGATTATGATGAATCTACCTTCACGATAATAATAATAGATATGAACGACAACTGG 2277

AMH-2_5 GGACGAGATTATGATGAATCTACCTTCACGATAATAATAATAGATATGAACGACAACTGG 2280

AMH-2_1 GGACGAGATTATGATGAATCTACCTTCACGATAATAATAATAGATATGAACGACAACTGG 2280

AMH-2_4 GGACGAGATTATGATGAATCTACCTTCACGATAATAATAATAGATATGAACGACAACTGG 2280

AMH-3_1 GGACGAGATTATGATGAATCTACCTTCACGATAATAATAATAGATATGAACGACAACTGG 2280

AMH-3_13 GGACGAGATTATGATGAATCTACCTTCACGATAATAATAATAGATGCGAACGACAACTGG 2280

AMH-1_7 GGACGAGATTATGATGAATCTACCTTCACGATAATAATAATAGATGTGAACGACAACTGG 2280

AMH-1_11 GGACGAGATTATGATGAATCTACCTTCACGATAATAATAATAGATATGAACGACAACTGG 2280

KMP-8_3 GGACGAGATTATGATGAATCTACCTTCACGATAATAATAATAGATATGAACGACAACTGG 2277

AMH-1_2 GGACGAGATTATGATGAATCTACTTTCACGATAATAATAATAGATATGAACGACAACTGG 2280

AY198374.1 GGACGAGATTATGATGAATCTACCTTCACGATAATAATAATAGATATGAACGACAACTGG 2280

*********************** *********************. *************

KMP-8_35 CCTATCTGGGCGTCTGGTTTCCTGAACCAGACCTTCAGTATTCGGGAGCGATCATCTACC 2337

KMP-8_5 CCTATCTGGGCGTCTGGTTTCCTGAACCAGACCTTCAGTATTCGGGAGCGATCATCTACC 2337

KMP-8_24 CCTATCTGGGCGTCTGGTTTCCTGAACCAGACCTTCAGTATTCGGGAGCGATCATCTACC 2337

KMP-8_46 CCTATCTGGGCGTCTGGTTTCCTGAACCAGACCTTCAGTATTCGGGAGCGATCATCTACC 2337

AMH-3_16 CCTATCTGGGCGTCTGGTTTCCTGAACCAGACCTTCAGTATTCGGGAGCGATCATCTACC 2337

AMH-2_5 CCTATCTGGGCGTCTGGTTTCCTGAACCAGACCTTCAGTATCCGGGAGCGATCATCTACC 2340

AMH-2_1 CCTATCTGGGCGTCTGGTTTCCTGAACCAGACCTTCAGTATCCGGGAGCGATCATCTACC 2340

AMH-2_4 CCTATCTGGGCGTCTGGTTTCCTGAACCAGACCTTCAGTATCCGGGAGCGATCATCTACC 2340

AMH-3_1 CCTATCTGGGCGTCTGGTTTCCTGAACCAGACCTTCAGTATTCGGGAGCGATCATCTACC 2340

AMH-3_13 CCTATCTGGGCGTCTGGTTTCCTGAACCAGACCTTCAGTATTCGGGAGCGATCATCTACC 2340

AMH-1_7 CCGATCTGGGCGTCTGGTTTCCTGAACCAGACCTTCAGTATTCGGGAGCGATCATCTACC 2340

AMH-1_11 CCTATCTGGGCGTCTGGTTTCCTGAACCAGACCTTCAGTATTCGGGAGCGATCATCTACC 2340

KMP-8_3 CCTATCTGGGCGTCTGGTTTCCTGAACCAGACCTTCAGTATTCGGGAGCGATCATCTACC 2337

AMH-1_2 CCTATCTGGGCGTCTGGTTTCCTGAACCAGACCTTCAGTATCCGGGAGCGATCATCTACC 2340

AY198374.1 CCTATCTGGGCGTCTGGTTTCCTGAACCAGACCTTCAGTATTCGGGAGCGATCATCTACC 2340

** ************************************** ******************

KMP-8_35 GGCGTCGTCATCGGGTCCGTACTCGCTACAGACATTGATGGCCCACTTTGCAACCAAGTC 2397

KMP-8_5 GGCGTCGTCATCGGGTCCGTACTCGCTACAGACATTGATGGCCCACTTTACAACCAAGTC 2397

KMP-8_24 GGCGTCGTCATCGGGTCCGTACTCGCTACAGACATTGATGGCCCACTTTACAACCAAGTC 2397

KMP-8_46 GGCGTCGTCATCGGGTCCGTACTCGCTACAGACATTGATGGCCCACTTTACAACCAAGTC 2397

AMH-3_16 GGCGTCGTCATCGGGTCCGTACTCGCTACAGACATTGATGGCCCACTTTACAACCAAGTC 2397

AMH-2_5 GGCGTCGTCATCGGGTCCGTACTCGCTACAGACATTGATGGCCCACTTTACAACCAAGTC 2400

AMH-2_1 GGCGTCGTCATCGGGTCCGTACTCGCTACAGACATTGATGGCCCACTTTACAACCAAGTC 2400

AMH-2_4 GGCGTCGTCATCGGGTCCGTACTCGCTACAGACATTGATGGCCCACTTTACAACCAAGTC 2400

AMH-3_1 GGCGTCGTCATCGGGTCCGTACTCGCTACAGACATTGATGGCCCACTTTACAACCAAGTC 2400

AMH-3_13 GGCGTCGTCATCGGGTCCGTACTCGCTACAGACATTGATGGCCCACTTTACAACCAAGTC 2400

AMH-1_7 GGCGTCGTCATCGGGTCCGTACTCGCTACAGACATTGATGGCCCACTTTACAACCAAGTC 2400

AMH-1_11 GGCGTCGTCATCGGGTCCGTACTCGCTACAGACATTGATGGCCCACTTTACAACCAAGTC 2400

KMP-8_3 GGCGTCGTCATCGGGTCCGTACTCGCTACAGACATTGACGGCCCACTTTACAACCAAGTC 2397

AMH-1_2 GGCGTCGTCATCGGGTCCGTACTCGCTACAGACATTGATGGCCCACTTTACAACCAAGTC 2400

AY198374.1 GGCGTCGTCATCGGGTCCGTACTCGCTACAGACATTGATGGCCCACTTTACAACCAAGTC 2400

************************************** **********.**********

KMP-8_35 CGGTATACCATTATCCCCCAGGAAGATACTCCTGAAGGTCTAGTCCAGATACACTTCGTT 2457

KMP-8_5 CGGTATACCATTATCCCCCAGGAAGATACTCCTGAAGGTCTAGTCCAGATACACTTCGTT 2457

KMP-8_24 CGGTATACCATTATCCCCCAGGAAGATACTCCTGAAGGTCTAGTCCAGATACACTTCGTT 2457

KMP-8_46 CGGTATACCATTATCCCCCAGGAAGATACTCCTGAAGGTCTAGTCCAGATACACTTCGTT 2457

AMH-3_16 CGGTACACCATTATCCCCCAGGAAGATACTCCTGAAGGTCTAGTCCAGATACACTTCGAT 2457

AMH-2_5 CGGTACACAATTATCCCCCAGGAAGATACTCCTGAAGGTCTAGTCCAGATACATTTCGTC 2460

AMH-2_1 CGGTACACAATTATCCCCCAGGAAGATACTCCTGAAGGTCTAGTCCAGATACATTTCGTT 2460

AMH-2_4 CGGTACACAATTATCCCCCAGGAAGATACTCCTGAAGGTCTAGTCCAGATACATTTCGTT 2460

AMH-3_1 CGGTACACCATTATCCCCCAGGAAGATACTCCTGAAGGTCTAGTCCAGATACACTTCGTT 2460

AMH-3_13 CGGTACACCATTATCCCCCAGGAAGATACTCCTGAAGGTCTAGTCCAGATACACTTCGTT 2460

AMH-1_7 CGGTACACCATTATCCCCCAGGAAGATACTCCTGAAGGTCTAGTCCAGATACACTTCGTT 2460

AMH-1_11 CGGTACACCATTATCCCCCAGGAAGATACTCCTGAAGGCCTAGTCCAGATACACTTCGTT 2460

KMP-8_3 CGGTATACCATTATCCCCCAGGAAGATACTCCTGAAGGTCTAGTCCAGATACACTTCGTT 2457

AMH-1_2 CGGTACACCATTATCCCCCAGGAAGATACTCCTGAAGGTCCAGTCCAGATACACTTCGTT 2460

AY198374.1 CGGTACACCATTATCCCCCAGGAAGATACTCCTGAAGGTCTAGTCCAGATACACTTCGTT 2460

***** **.***************************** * ************ ****:

KMP-8_35 ACGGGTCAGATTACAGTTGATGAGAATGGTGCAATCGACGCTGATATTCCACCTCGTTGG 2517

KMP-8_5 ACGGGTCAGATTACAGTTGATGAGAATGGTGCAATCGACGCTGATATTCCACCTCGTTGG 2517

KMP-8_24 ACGGGTCAGATTACAGTTGATGAGAATGGTGCAATCGACGCTGATATTCCACCTCGTTGG 2517

KMP-8_46 ACGGGTCAGATTACAGTTGATGAGAATGGTGCAATCGACGCTGATATTCCACCTCGTTGG 2517

AMH-3_16 ACGGGTCAGATTACAGTTGATGAGAATGGTGCAATCGACGCTGATATTCCACCTCGTTGG 2517

AMH-2_5 ACGGGTCAGATTACAGTTGATGAGAATGGTGCAATCGACGCTGATATTCCACCTCGTTGG 2520

AMH-2_1 ACGGGTCAGATTACAGTTGATGAGAATGGTGCAATCGACGCTGATATTCCACCTCGTTGG 2520

AMH-2_4 ACGGGTCAGATTACAGTTGATGAGAATGGTGCAATCGACGCTGATATTCCACCTCGTTGG 2520

AMH-3_1 ACGGGTCAGATTACAGTTGATGAGAATGGTGCAATCGACGCTGATATTCCACCTCGTTGG 2520

AMH-3_13 ACGGGTCAGATTACAGTTGATGAGAATGGTGCAATCGACGCTGATATTCCACCTCGTTGG 2520

AMH-1_7 ACGGGTCAGATTACAGTTGATGAGAATGGTGCAATCGACGCTGATATTCCACCTCGTTGG 2520

AMH-1_11 ACGGGTCAGATTACAGTTGATGAGAATGGTGCAATCGACGCTGATATTCCACCTCGTTGG 2520

KMP-8_3 ACGGGTCAGATTACAGTTGATGAGAATGGTGCAATCGACGCTGATATTCCACCTCGTTGG 2517

AMH-1_2 ACGGGTCAGATTACAGTTGATGAGAATGGTGCAATCGACGCTGATATTCCACCTCGTTGG 2520

AY198374.1 ACGGGTCAGATTACAGTTGATGAGAATGGTGCAATCGACGCTGATATTCCACCTCGTTGG 2520

************************************************************

KMP-8_35 CACCTCAACTACACGATTATAGCCAGCGACAAATGTTCCGAAGAAAATGAAGAGAACTGT 2577

KMP-8_5 CACCTCAACTACACGATTATAGCCAGCGACAAATGTTCCGAAGAAAATGAAGAGAACTGT 2577

KMP-8_24 CACCTCAACTACACGATTATAGCCAGCGACAAATGTTCCGAAGAAAATGAAGAGAACTGT 2577

KMP-8_46 CACCTCAACTACACGATTATAGCCAGCGACAAATGTTCCGAAGAAAATGAAGAGAACTGT 2577

AMH-3_16 CACCTCAACTACGCGGTTATAGCCAGCGACAAATGTTCTGAAGAAAATGAAGAGAACTGT 2577

AMH-2_5 CACCTCAACTACACGGTTATAGCCAGCGACAAATGTTCTGAAGAAAATGAAGAGAACTGT 2580

AMH-2_1 CACCTCAACTACACGGTTATAGCCAGCGACAAATGTTCTGAAGAAAATGAAGAGAACTGT 2580

AMH-2_4 CACCTCAACTACACGGTTATAGCCAGCGACAAATGCTCTGAAGAAAATGAAGAGAACTGT 2580

AMH-3_1 CACCTCAACTACACGGTTATAGCCAGCGACAAATGTTCCGAAGAAAATGAAGAGAACTGT 2580

AMH-3_13 CACCTCAACTACACGGTTATAGCCAGCGACAAATGTTCCGAAGAAAATGAAGAGAACCGT 2580

AMH-1_7 CACCTCAACTACACAGTTATAGCCAGCGACAAATGTTCCGAAGAAAATGAAGAGAACTGT 2580

AMH-1_11 CACCTCAACTACACAGTTATAGCCAGCGACAAATGTTCCGAAGAAAATGAAGAGAACTGT 2580

KMP-8_3 CACCTCAACTACACGATTATAGCCAGCGACAAATGTTCCGAAGAAAATGAAGAGAACTGT 2577

AMH-1_2 CACCTCAACTACACGGTTATAGCCAGCGACAAATGTTCCGAAGAAAATGAAGAGAACTGT 2580

AY198374.1 CACCTCAACTACACGGTTATAGCCAGCGACAAATGTTCCGAAGAAAATGAAGAGAACTGT 2580

************.*..******************* ** ****************** **

KMP-8_35 CCCCCGGATCCAGTGTTCTGGGATACTCTGGGCGACAATGTAATTAACATCGTGGACATA 2637

KMP-8_5 CCCCCGGATCCAGTGTTCTGGGATACTCTGGGCGACAATGTAATTAACATCGTGGACATA 2637

KMP-8_24 CCCCCGGATCCAGTGTTCTGGGATACTCTGGGCGACAATGTAATTAACATCGTGGACATA 2637

KMP-8_46 CCCCCGGATCCAGTGTTCTGGGATACTCTGGGCGACAATGTAATTAACATCGTGGACATA 2637

AMH-3_16 CCCCCGGATCCAGTATTCTGGGATACTCTGCGCGACAACGTAATTAACATCGTGGACATA 2637

AMH-2_5 CCCCCGGATCCAGTGTTCTGGGATACTCTGCGCGACAATGTAATTAACATCGTGGACATA 2640

AMH-2_1 CCCCCGGATCCAGTGTTCTGGGATACTCTGCGCGACAATGTAATTAACATCGTGGACATA 2640

AMH-2_4 CCCCCGGATCCAGTGTTCTGGGATACTCTGCGCGACAATGTAATTAACATCGTGGACATA 2640

AMH-3_1 CCCCCGGATCCAGTGTTCTGGGATACTCTGGGTGACAATGTAATTAACATCGTGGACATA 2640

AMH-3_13 CCCCCGGATCCAGTGTCCTGGGATACTCTGGGTGACAATGTAATTAACATCGTGGACATA 2640

AMH-1_7 CCCCCGGATCCAGTGTTCTGGGATACTCTGGGCGACAATGTAATTAACATCGTGGACATA 2640

AMH-1_11 CCCCCGGATCCAGTGTTCTGGGATACTCTGGGCGACAATGTAATTAACATCGTGGACATA 2640

KMP-8_3 CCCCCGGATCCAGTGTTCTGGGATACTCTGGGCGACAATGTAATTAACATCGTGGACATA 2637

AMH-1_2 CCCCCGGATCCAGTGTTCTGGGATACTCTGGGCGACAATGTAATTAACATCGTGGACATA 2640

AY198374.1 CCCCCGGATCCAGTGTTCTGGGATACTCTGGGCGACAATGTAATTAACATCGTGGACATA 2640

**************.* ************* * ***** *********************

KMP-8_35 AACGACAAGGTCCCGGCAGCAGACCTCAGTCGATTCAACGAAACGGTGTACATTTATGAA 2697

KMP-8_5 AACAACAAGGTCCCGGCAGCAGACCTCAGTCGATTCAACGAAACGGTGTACATTTATGAA 2697

KMP-8_24 AACAACAAGGTCCCGGCAGCAGGCCTCAGTCGATTCAACGAAACGGTGTACATTTATGAA 2697

KMP-8_46 AACAACAAGGTCCCGGCAGCAGACCTCAGTCGATTCAACGAAACGGTGTACGTTTATGAA 2697

AMH-3_16 AACAACAAGGTCCCGGCAGCAGACCTCAGTAGACTCAACGAAACGGTGTACATTCATGAA 2697

AMH-2_5 AACAACAAGGTCCCGGCAGCAGACCTCAGTCGATTCAACGAAACGGTGTACATTTATGAA 2700

AMH-2_1 AACAACAAGGTCCCGGCAGCAGACCTCAGTCGATTCAACGAAACGGTGTACATTTATGAA 2700

AMH-2_4 AACAACAAGGTCCCGGCAGCAGACCTCAGTCGATTCAACGAAACGGTGTACATTTACGAA 2700

AMH-3_1 AACAACAAGGTCCCGGCAGCAGACCTCAGTCGATTCAACGAAACGGTGTACATTTATGAA 2700

AMH-3_13 AACAACAAGGTCCCGGCAGCAGACCTCAGTCGATTCAACGAAACGGTGTACATTTATGAA 2700

AMH-1_7 AACAACAAGGTCCCGGCAGCAGACCTCAGTAGATTCAACGAAACGGTGTACATTTATGAA 2700

AMH-1_11 AACAACAAGGTCCCGGCAGCAGACCTCAGTAGATTCAACGAAACGGTGTACATTTATGAA 2700

KMP-8_3 AACAACAAGGTCCCGGCAGCAGACCTCAGTCGATTCAACGAAACGGTGTACATTTATGAA 2697

AMH-1_2 AACAACAAGGTCCCGGCAGCAGACCTCAGTCGATTCAACGAAACGGTGTACATTTATGAA 2700

AY198374.1 AACAACAAGGTCCCGGCAGCAGACCTCAGTCGATTCAACGAAACGGTGTACATTTATGAA 2700

***.******************.*******.** *****************.** * ***

KMP-8_35 AATGCACCCGATTTCACAAACGTGGTCAAGATATACTCCATCGACGAAGACAGAGACGAA 2757

KMP-8_5 AATGCACCCGATTTCACAAACGTGGTCAAGATATACTCCATCGACGAAGACAGAGACGAA 2757

KMP-8_24 AATGCACCCGATTTCACAAACGTGGTCAAGATATACTCCATCGACGAAGACAGAGACGAA 2757

KMP-8_46 AATGCACCCGATTTCACAAACGTGGTCAAGATATACTCCATCGACGAAGACAGAGACGAA 2757

AMH-3_16 AATGCACCCGATTTCACAAACGTGGTCAAGATATACTCCATCGACGAAGACAGAGACGAA 2757

AMH-2_5 AATGCACCCGATTTCACAAACGTGGTCAAGATATACTCCATCGACGAAGACAGAGACGAA 2760

AMH-2_1 AATGCACCCGATTTCACAAACGTGGTCAAGATATACTCCATCGACGAAGACAGAGACGAA 2760

AMH-2_4 AATGCACCCGATTTCACAAACGTGGTCAAGATATACTCCATCGACGAAGACAGAGACGAA 2760

AMH-3_1 AATGCACCCGATTTCACAAACGTGGTCAAGATATACTCCATCGACGAAGACAGAGACGAA 2760

AMH-3_13 AATGCACCCGATTTCACAAACGTGGTCAAGATATACTCCATCGACGAAGACAGAGACGAA 2760

AMH-1_7 AATGCACCCGATTTCACAAACGTGGTCAAGATATACTCCATCGACGAAGGCAGAGACGAA 2760

AMH-1_11 AATGCACCCGATTTCACAAACGTGGTCAAGATATACTCCATCGACGAAGACAGAGACGAA 2760

KMP-8_3 AATGCACCCGATTTCACAAACGTGGTCAAGATATACTCTATCGACGAAGACAGAGACGAA 2757

AMH-1_2 AATGCACCCGATTTCACAAACGTGGTCAAGATATACTCCATCGACGAAGACAGAGACGAA 2760

AY198374.1 AATGCACCCGATTTCACAAACGTGGTCAAGATATACTCCATCGACGAAGACAGAGACGAA 2760

************************************** **********.**********

KMP-8_35 ATATATCACACGGTGCGGTACCAGATCAATCATGCTGTGAACCAGCGGCTGCGAGACTTC 2817

KMP-8_5 ATATATCACACGGTGCGGTACCAGATCAATTATGCTGTGAACCAGCGGCTGCGAGACTTC 2817

KMP-8_24 ATATATCACACGGTGCGGTACCAGATCAATTATGCTGTGAACCAGCGGCTGCGAGACTTC 2817

KMP-8_46 ATATATCACACGGTGCGGTACCAGATCAATTATGCTGTGAACCAGCGGCTGCGAGACTTC 2817

AMH-3_16 ATATATCACACGGTGCGGTACCAGATCAATTATGCTGTGAACCAGCGGCTGCGAGGTTTC 2817

AMH-2_5 ATATATCACACGGTGCGGTACCAGATCAATTATGCTGTGAACCAGCGGCTGCGAGACTTC 2820

AMH-2_1 ATATATCACACGGTGCGGTACCAGATCAATTATGCTGTGAACCAGCGGCTGCGAGACTTC 2820

AMH-2_4 ATATATCACACGGTGCGGTACCAGATCAATTATGCTGTGAACCAGCGGCTGCGAGACTTC 2820

AMH-3_1 ATATATCACACGGTGCGGTACCAGATCAATTGTGCTGTGAACCAGCGGCTGCGAGACTTC 2820

AMH-3_13 ATATATCACACGGTGCGGTACCAGATCAATTATGCTGTGAACCAGCGGCTGCGAGACTTC 2820

AMH-1_7 ATATATCACACGGTGCGGTACCAGATCAATTATGCTGTGAACCAGCGGCTGCGAGACTTC 2820

AMH-1_11 ATATATCACACGGTGCGGTACCAGATCAATTATGCTGTGAACCAGCGGCTGCGAGACTTC 2820

KMP-8_3 ATATATCACACGGTGCGGTACCAGATCAATTATGCTGTGAACCAGCGGCTGCGAGACTTC 2817

AMH-1_2 ATATATCACACGGTGCGGTACCAGATCAATTATGCTGTGAACCAGCGGCTGCGAGACTTC 2820

AY198374.1 ATATATCACACGGTGCGGTACCAGATCAATTATGCTGTGAACCAGCGGCTGCGAGACTTC 2820

****************************** .***********************. ***

KMP-8_35 TTCGCCATAGACCTGGATTCAGGCCAGGTGTACGTGGAGAACACCAACAATGAGCTCCTG 2877

KMP-8_5 TTCGCCATAGACCTGGATTCAGGCCAGGTGTACGTGGAGAACACCAACAATGAGCTCCTG 2877

KMP-8_24 TTCGCCATAGACCTGGATTCAGGCCAGGTGTACGTGGAGAACACCAACAATGAGCTCCTG 2877

KMP-8_46 TTCGCCATAGACCTGGATTCAGGCCAGGTGTACGTGGAGAACACCAACAATGAGCTCCTG 2877

AMH-3_16 TTCGCCATAGACCTGGATTCAGGCCAGGTGTACGTGGAGAACACCAACAATGAGCTCCTG 2877

AMH-2_5 TTCGCCATAGACCTGGATTCAGGCCAGGTGTACGTGGAGAACACCAACAATGAGCTCCTG 2880

AMH-2_1 TTCGCCATAGACCTGGATTCAGGCCAGGTGTACGTGGAGAACACCAACAATGAGCTCCTG 2880

AMH-2_4 TTCGCCATAGACCTGGATTCAGGCCAGGTGTACGTGGAGAACACCAACAATGAGCTCCTG 2880

AMH-3_1 TTCGCCATAGACCTGGATTCAGGCCAGGTGTACGTGGAGAACACCAACAATGAGCTCCTG 2880

AMH-3_13 TTCGCCATAGACCTGGATTCAGGCCAGGTGTACGTGGAGAACGCCAACAATGAGCTCCTG 2880

AMH-1_7 TTCGCCATAGACCTGGATTCAGGCCAGGTGTACGTGGAGAACACCAACAATGAGCTCCTG 2880

AMH-1_11 TTCGCCATAGACCTGGATTCAGGCCAGGTGTACGTGGAGAACACCAACAATGAGCTCCTG 2880

KMP-8_3 CTCGCCATAGACCTGGATTCAGGCCAGGTGTACGTGGAGAACACCAACAATGAGCTCCTG 2877

AMH-1_2 TTCGCCATAGACCTGGATTCAGGCCAGGTGTACGTGGAGAACACCAACAATGAGCTCCTG 2880

AY198374.1 TTCGCCATAGACCTGGATTCAGGCCAGGTGTACGTGGAGAACACCAACAATGAGCTCCTG 2880

*****************************************.*****************

KMP-8_35 GATCGGGACAGAGGCGAAGACCAACACAGGATATTCATTAACCTCATTGACAACTTTTAT 2937

KMP-8_5 GATCGGGACAGAGGCGAAGACCAACACAGGATATTCATTAACCTCATTGACAACTTTTAT 2937

KMP-8_24 GATCGGGACAGAGGCGAAGACCAACACAGGATATTCATTAACCTCATTGACAACTTTTAT 2937

KMP-8_46 GATCGGGACAGAGGCGAAGACCAACACAGGATATTCATTAACCTCATTGACAACTTTTAT 2937

AMH-3_16 GATCGGGACAGAGGCGAAGACCAACACAGGATATTCATTAACCTCATTGACAACTTTTAT 2937

AMH-2_5 GATCGGGACAGAGGCGAAGACCAACACAGGATATTCATTAACCTCATTGACAACTTTTAT 2940

AMH-2_1 GATCGGGGCAGAGGCGAAGACCAACACAGGATATTCATTAACTTCATTGACAACTTTTAT 2940

AMH-2_4 GATCGGGACAGAGGCGAAGACCAACACAGGATATTCATTAACCTCATTGACAACTTTTAT 2940

AMH-3_1 GATCGGGGCAGAGGCGAAGACCAACACAGGATATTCATTAACCACATTGACAACTTTTAT 2940

AMH-3_13 GATCGGGACAGAGGCGAAGACCAACACAGGATATTCATTAACCTCATTGACAACTTTTAT 2940

AMH-1_7 GATCGGGACGGAGGCGAAGACCAACACAGGATATTCATTAACCTCATTGACAACTTTTAT 2940

AMH-1_11 GATCGGGACAGAGGCGAAGACCAACACAGGATATTCATTAACCTCATTGACAACTTTTAT 2940

KMP-8_3 GATCGGGACAGAGGCGAAGACCAACACAGGATATTCATTAACCTCATTGACAACTTTTAT 2937

AMH-1_2 GATCGGGACAGAGGCGAGGACCAACACAGGATATTCATTAACCTCATTGACAACTTTTAT 2940

AY198374.1 GATCGGGACAGAGGCGAAGACCAACACAGGATATTCATTAACCTCATTGACAACTTTTAT 2940

*******.*.*******.************************ :****************

KMP-8_35 AGCGAAGGAGATGGAAATAGAAATGTAAACACTACAGAGGTGCTGGTGATACTATTAGAT 2997

KMP-8_5 AGCGAAGGAGATGGAAATAGAAATGTAAACACTACAGAGGTGCTGGTGATACTATTAGAT 2997

KMP-8_24 AGCGAAGGAGATGGAAATAGAAATGTAAACACTACAGAGGTGCTGGTGATACTATTAGAT 2997

KMP-8_46 AGCGAAGGAGATGGAAATAGAAATGTAAACACTACAGAGGTGCTGGTGATACTATTAGAT 2997

AMH-3_16 AGCGAAGGAGATGGAAATAGAAATGTAAACACTACAGAGGTGCTGGTGATACTATTAGAT 2997

AMH-2_5 AGCGAAGGAGATGGAAATAGAAATGTAAACACTACAGAGGTGCTGGTGATACTATTAGAT 3000

AMH-2_1 AGCGAAGGAGATGGAAATAGAAATGTAAACACTACAGAGGTGCTGGTGATACTATTAGAT 3000

AMH-2_4 AGCGAAGGAGATGGAAATAGAAATGTAAACACTACAGAGGTGCTGGTGATACTATTAGAT 3000

AMH-3_1 AGCGAAGGAGATGGAAATAGAAATGTAAACACTACAGAGGTGCTGGTGATACTATTAGAT 3000

AMH-3_13 AGCGAAGGAGATGGAAATAGAAATGTAAACACCACAGAGGTGCTGGTGATACTATTAGAT 3000

AMH-1_7 AGCGAAGGAGATGGAAATAGAAATGTAAACACTACAGAGGTGCTGGTGATACTATTAGAT 3000

AMH-1_11 AGCGAAGGAGATGGAAATAGAAATGTAAACACTACAGAGGTGCTGGTGATACTATTAGAT 3000

KMP-8_3 AGCGAAGGAGATGGAAATAGAAATGTAAACACTACAGAGGTGCTGGTGATACTATTAGAT 2997

AMH-1_2 AGCGAAGGAGATGGAAATAGAAATGTAAACACTACAGAGGTGCTGGTGATACTATTAGAT 3000

AY198374.1 AGCGAAGGAGATGGAAATAGAAATGTAAACACTACAGAGGTGCTGGTGATACTATTAGAT 3000

******************************** ***************************

KMP-8_35 GAAAATGACAACGCTCCTGAATTGCCGACTCCAGAAGAGCTGAGTTGGAGCACTTCCGAG 3057

KMP-8_5 GAAAATGACAACGCTCCTGAATTGCCGACTCCAGAAGAGCTGAGTTGGAGCATTTCCGAG 3057

KMP-8_24 GAAAATGACAACGCTCCTGAATTGCCGACTCCAGAAGAGCTGAGTTGGAGCATTTCCGAG 3057

KMP-8_46 GAAAATGACAACGCTCCTGAATTGCCGACTCCAGAAGAGCTGAGTTGGAGCATTTCCGAG 3057

AMH-3_16 GAGAATGACAACGCTCCTGAATTGCCGACTCCAGAAGAGCTGAGTTGGAGCATTTCCGAG 3057

AMH-2_5 GAGAATGACAACGCTCCTGAATTGCCGACTCCAGAAGAGCTGAGTTGGAGCATTTCCGAG 3060

AMH-2_1 GAGAATGACAACGCTCCTGAATTGCCGACTCCAGAAGAGCTGAGTTGGAGCATTTCCGAG 3060

AMH-2_4 GAGAATGACAACGCTCCTGAATTGCCGACTCCAGAAGAGCTGAGTTGGAGCATTTCCGAG 3060

AMH-3_1 GAAAATGACAACGCTCCTGAATTGCCGACTCCAGAAGAGCTGAGTTGGAGCATTTCCGAG 3060

AMH-3_13 GAAAATGACAACGCTCCTGAATTGCCGACTCCAGAAGAGCTGAGTTGGAGCATTTCCGAG 3060

AMH-1_7 GAGAATGACAACGCTCCTGAATTGTCGACTCCAGAAGAGCTGAGTTGGAGCATTTCCGAG 3060

AMH-1_11 GAGAATGACAACGCTCCTGAATTGCCGACTCCAGAAGAGCTGAGTTGGAGCATTTCCGAG 3060

KMP-8_3 GAGAATGACAACGCTCCTGAATTGCCGACTCCAGAAGAGCTGAGTTGGAGCATTTCCGAG 3057

AMH-1_2 GAAAATGACAACGCTCCTGAATTGCCGACTCCAGAAGAGCTGAGTTGGAGCATTTCCGAG 3060

AY198374.1 GAGAATGACAACGCTCCTGAATTGCCGACTCCAGAAGAGCTGAGTTGGAGCATTTCCGAG 3060

**.********************* *************************** *******

KMP-8_35 AATTTACAAGAGGGTATAACACTCGATGGCGAACGCGATGTAATATACGCACCGGATATA 3117

KMP-8_5 AATTTACAAGAGGGTATAACACTCGATGGCGAACGCGATGTAATATACGCACCGGATATA 3117

KMP-8_24 AATTTACAAGAGGGTATAACACTCGATGGCGAACGCGATGTAATATACGCACCGGATATA 3117

KMP-8_46 AATTTACAAGAGGGTATAACACTCGATGGCGAACGCGATGTAATATACGCACCGGATATA 3117

AMH-3_16 AATTTACAAGAGGGTATAACACTCGATGGCGAAAGCGATGTGATATACGCACCGGATATA 3117

AMH-2_5 AATTTACAAGAGGGTATAACACTCGATGGCGAAAGCGATGTGATATACGCACCGGATATA 3120

AMH-2_1 AATTTACAAGAGGGTATAACACTCGATGGCGAAAGCGATGTGGTATACGCACCGGATATA 3120

AMH-2_4 AATTTACAAGAGGGTATAACACTCGATGGCGAAAGCGATGTGATATACGCACCGGATATA 3120

AMH-3_1 AATTTACAAGAGGGTATAACACTCGATGGCGAACGCGATGTAATATACGCACCGGATATA 3120

AMH-3_13 AATTTACAAGAGGGTATAACGCTCGATGGCGAACGCGATGTAATATACGCACCGGATATA 3120

AMH-1_7 AATTTACAAGAGGGTATAACACTCGATGGCGAAAGCGATGTGATATACGCACCGGATATA 3120

AMH-1_11 AATTTACAAGAGGGTATAACACTCGATGGCGAAAGCGATGTGATATACGCACCGGATATA 3120

KMP-8_3 AATTTACAAGAGGGTATAACACTCGATGGCGAAAGCGATGTGATATACGCACCGGATATA 3117

AMH-1_2 AATTTACAAGAGGGTATAACACTCGATGGCGAACGCGATGTAATATACGCACCGGATATA 3120

AY198374.1 AATTTACAAGAGGGTATAACACTCGATGGCGAAAGCGATGTGATATACGCACCGGATATA 3120

********************.************.*******..*****************

KMP-8_35 GACGAAGAGGACACGCCAAACTCTCACGTTGGCTACGCAATCCTGGCCATAACAGTCACC 3177

KMP-8_5 GACGAAGAGGACACGCCAAACTCTCACGTTGGCTACGCAACCCTGGCCATAACAGTCACC 3177

KMP-8_24 GACGAAGAGGACACGCCAAACTCTCACGTTGGCTACGCAATCCTGGCCATAACAGTCACC 3177

KMP-8_46 GACGAAGAGGACACGCCAAACTCTCACGTTGGCTACGCAATCCTGGCCATAACAGTCACC 3177

AMH-3_16 GACGAAGAGGACACGCCAAACTCTCACGTTGGCTACGCAATCCTGGCCATGACAGTCACC 3177

AMH-2_5 GACGAAGAGGACACGCCAAACTCTCACGTTGGCTACGCAATCCTGGCCATGACAGTCACC 3180

AMH-2_1 GACGAAGAGGACACGCCAAACTCTCACGTTGGCTACGCAATCCTGGCCATGACAGTCACC 3180

AMH-2_4 GACGAAGAGGACACGCCAAACTCTCACGTTGGCTACGCAATCCTGGCCATGACAGTCACC 3180

AMH-3_1 GACGAAGAGGACACGCCAAACTCTCACGTTGGCTACGCAATCCTGGCCATGACAGTCACC 3180

AMH-3_13 GACGAAGAGGACACGCCAAACTCTCACGTTGGCTACGCAATCCTGGCCATGACAGTCACC 3180

AMH-1_7 GACGAAGAGGACACGCCAAACTCTCACGTTGGCTACGCAATCCTGGCCATGACAGTCACC 3180

AMH-1_11 GACGAAGAGGACACGCCAAACTCTCACGTTGGCTACGCAATCCTGGCCATGACAGTCACC 3180

KMP-8_3 GACGAAGAGGACACGCCAAACTCTCACGTTGGCTACGCAATCCTGGCCATGACAGTCACC 3177

AMH-1_2 GACGAAGAGGACACGCCAAACTCTCACGTTGGCTACGCAATCCTGGCCATGACAGTCACC 3180

AY198374.1 GACGAAGAGGACACGCCAAACTCTCACGTTGGCTACGCAATCCTGGCCATGACAGTCACC 3180

**************************************** *********.*********

KMP-8_35 AATAGAGACCTGGACACTGCTCCGAGACTTCTGAACATGCTGTCGCCTAACAACGTAACC 3237

KMP-8_5 AATAGAGACCTGGACACTGTTCCGAGACTTCCGAACATGCTGTCGCCTAACAACGTAACC 3237

KMP-8_24 AATAGAGACCTGGACACTGTTCCGAGACTTCTGAACATGCTGTCGCCTAACAACGTAACC 3237

KMP-8_46 AATAGAGACCTGGACACTGTTCCGAGACTTCTGAACATGCTGTCGCCTAACAACGTAACC 3237

AMH-3_16 AATAGAGACCTGGACACTGTTCCGAGACTTCTCAACATGCTGTCGCCTAACAACGTAACC 3237

AMH-2_5 AATAGAGACCTGGACACTGTTCCGAGACTTCTCAACATGCTGTCGCCTAACAACGTAACC 3240

AMH-2_1 AATAGAGACCTGGACACTGTTCCGAGACTTCTCAACATGCTGTCGCCTAACAACGTAACC 3240

AMH-2_4 AATAGAGACCTGGACACTGTTCCGAGACTTCTCAACATGCTGTCGCCTAACAACGTAACC 3240

AMH-3_1 AATAGAGACCTGGACACTGTTCCGAGACTTTTCAACATGCTGTCGCCTAACAACGTAACC 3240

AMH-3_13 AATAGAGACCTGGACACTGTTCCGAGACTTCTCAACATGCTGTCGCCTAACAACGTAACC 3240

AMH-1_7 AATAGAGACCTGGACACTGTTCCGAGACTTCTCAACATGCTGTCGCCTAACAACGTAACC 3240

AMH-1_11 AATAGAGACCTGGACACTGTTCCGAGACTTCTCAACATGCTGTCGCCTAACAACGTAACC 3240

KMP-8_3 AATAGAGACCTGGACACTGTTCCGAGACTTCTCAACATGCTGTCGCCTAACAACGTAACC 3237

AMH-1_2 AATAGAGACCTGGACACTGTTCCGAGACTTCTCAACATGCTGTCGCCTAACAACGTAACC 3240

AY198374.1 AATAGAGACCTGGACACTGTTCCGAGACTTCTCAACATGCTGTCGCCTAACAACGTAACC 3240

******************* ********** ***************************

KMP-8_35 GGATTCCTCCAGACAGCAATGCCTTTAAGAGGATATTGGGGGACTTACGATATAAGTATA 3297

KMP-8_5 GGATTCCTCCAGACAGCAATGCCTTTAAGAGGATATTGGGGGACTTACGATATAAGTATA 3297

KMP-8_24 GGATTCCTCCAGACAGCAATGCCTTTAAGAGGATATTGGGGGACTTACGATATAAGTATA 3297

KMP-8_46 GGATTCCTCCAGACAGCAATGCCTTTAAGAGGATATTGGGGGACTTACGATATAAGTATA 3297

AMH-3_16 GGATTCCTCCAGACAGCAATGCCTTTGAGAGGATATTGGGGGACTTACGATATAAGTGTA 3297

AMH-2_5 GGATTCCTCCAGACAGCAATGCCTTTGAGAGGATATTGGGGGACTTACGATATAAGTGTA 3300

AMH-2_1 GGATTCCTCCAGACAGCAATGCCTTTGAGAGGATATTGGGGGACTTACGATATAAGTGTA 3300

AMH-2_4 GGATTCCTCCAGACAGCAATGCCTTTGAGAGGATATTGGGGGACTTACGATATAAGTGTA 3300

AMH-3_1 GGATTCCTCCAGACAGCAATGCCTTTAAGAGGATATTGGGGGACTTACGATATAAGTATA 3300

AMH-3_13 GGATTCCTCCAGACAGCAATGCCTTTAAGAGGATATTGGGGGACTTACGATATAAGTATA 3300

AMH-1_7 GGATTCCTTCAGACAGCAATGCCTTTGAGAGGATATTGGGGTACTTACGATATAAGTATA 3300

AMH-1_11 GGATTCCTTCAGACAGCAATGCCTTTGAGAGGATATTGGGGTACTTACGATATAAGTATA 3300

KMP-8_3 GGATTCCTTCAGACAGCAATGCCTTTGAGAGGATATTGGGATACTTACGATATAAGTATA 3297

AMH-1_2 GGATTCCTCCAGACAGCAATGCCTTTAAGAGGATATTGTGGGACTTACGATATAAGTATA 3300

AY198374.1 GGATTCCTCCAGACAGCAATGCCTTTGAGAGGATATTGGGGGACTTACGATATAAGTATA 3300

******** *****************.*********** *. ***************.**

KMP-8_35 CTGGCGTTCGACCACGGTATTCCTCAGCAGATATCTCATGAGGTGTATGAATTGGAAATT 3357

KMP-8_5 CTGGCGTTCGACCACGGTATTCCTCAGCAGATATCTCATGAGGTGTATGAATTGGAAATT 3357

KMP-8_24 CTGGCGTTCGACCACGGTATTCCTCAGCAGATATCTCATGAGGTGTATGAATTGGAAATC 3357

KMP-8_46 CTGGCGTTCGACCACGGTATTCCTCAGCAGATATCTCATGAGGTGTATGAATTGGAAATT 3357

AMH-3_16 CTGGCGTTCGACCACGGTATCCCTCAGCAGATATCTCATGAGGTGTATGAATTGGAAATT 3357

AMH-2_5 CTGGCGTTCGACCACGGTATTCCTCAGCAGATATCTCATGAGGTGTATGAATTGGAAATT 3360

AMH-2_1 CTGGCGTTCGACCACGGTATTCCTCAGCAGATATCTCATGAGGTGTATGAATTGGAAATT 3360

AMH-2_4 CTGGCGTTCGACCACGGTATTCCTCAGCAGATATCTCATGAGGTGTATGAATTGGAAATT 3360

AMH-3_1 CTGGCGTTCGACCACGGTATTCCTCAGCAGATATCTCATGAGGTGTATGAATTGGAAATT 3360

AMH-3_13 CTGGCGTTCGACCACGGTATTCCTCAGCAGATATCTCATGAGGTGTATGAATTGGAAATT 3360

AMH-1_7 CTGGCGTTCGACCACGGTATTCCTCAGCAGATATCTCATGAGGTGTATGAATTGGAAATT 3360

AMH-1_11 CTGGCGTTCGACCACGGTATTCCTCAGCAGATATCTCATGAGGTGTATGAATTGGAAATT 3360

KMP-8_3 CTGGCGTTCGACCACGGTATTCCTCAGCAGATATCTCATGAGGTGTATGAATTGGAAATT 3357

AMH-1_2 CTGGCGTTCGACCACGGTATTCCTCAGCAGATATCTCATGAGGTGTATGAATTGGAAATT 3360

AY198374.1 CTGGCGTTCGACCACGGTATTCCTCAGCAGATATCTCATGAGGTGTATGAATTGGAAATT 3360

******************** **************************************

KMP-8_35 CGACCTTACAATTACAATCCTCCCCAGTTCGTTTTTCCTGAATCCGGGACGATTCTACGA 3417

KMP-8_5 CGACCTTACAATTACAATCCTCCCCAGTTCGTTTTTCCTGAATCCGGGGCGATTCTACGA 3417

KMP-8_24 CGACCTTACAATTACAATCCTCCCCAGTTCGTTTTCCCTGAATCCGGGACGATTCTACGA 3417

KMP-8_46 CGACCTTACAATTACAATCCTCCCCAGTTCGTTTTTCCTGAATCCGGGACGATTCTACGA 3417

AMH-3_16 CGACCTTACAATTACAATCCTCCCCAGTTCGTTTTTCCCGAATCCGGGACGATTCTACGA 3417

AMH-2_5 CGACCTTACAATTACAATCCTCCCCAGTTCGTTTTTCCTGAATCCGGGACGATTCTACGA 3420

AMH-2_1 CGACCTTACAATTACAATCCTCCCCAGTTCGTTTTTCCTGAATCCGGGACGATTCTACGA 3420

AMH-2_4 CGACCTTTCAATTACAATCCTCCCCAGTTCGTTTTTCCTGAATCCGGGACGATTCTACGA 3420

AMH-3_1 CGACCCTACAATTACAATCCTCCTCAGTTCGTTTTTCCTGAATCCGGGACGATTCTACGA 3420

AMH-3_13 CGACCTTACAATTACAATCCTCCTCAGTTCGTTTTTCCTGAATCCGGGACGATTCTACGA 3420

AMH-1_7 CGACCTTACAATTACAATCCTCCCCAGTTCGTTTTTCCTGAATCCGGGACGATTCTACGA 3420

AMH-1_11 CGACCTTACAATTACAATCCTCCCCAGTTCGTTTTTCCTGAATCCGGGACGATTCTACGA 3420

KMP-8_3 CGACCTTACAATTACAATCCTCCCCAGTTCGTTTTTCCTGAATCCGGGACGATTCTACGA 3417

AMH-1_2 CGACCTTACAATTACAATCCTCCCCAGTTCGTTTTTCCTGAATCCGGGACGATTCTACGA 3420

AY198374.1 CGACCTTACAATTACAATCCTCCCCAGTTCGTTTTTCCTGAATCCGGGACGATTCTACGA 3420

***** *:*************** *********** ** *********.***********

KMP-8_35 CTGGCTTTGGAACGCGCAGTGGTAAATAATGTTTTGTCACTTGTAAACGGTGACCCGTTA 3477

KMP-8_5 CTGGCTTTGGAACGCGCAGTGGTAAATAATGTTTTGTCACTTGTAAACGGTGACCCGTTA 3477

KMP-8_24 CTGGCTTTGGAACGCGCAGTGGTAAATAATGTTTTGTCACTTGTAAACGGTGACCCGTTA 3477

KMP-8_46 CTGGCTTTGGAACGCGCAGTGGTAAATAATGTTTTGTCACTTGTAAACGGTGACCCGTTA 3477

AMH-3_16 CTGGCTTTGGAACGCGCTGTGGTAAATAATGTATTGTCACTTGTAAACGGTGACCCGTTA 3477

AMH-2_5 CTGGCTTTGGAACGCGCTGTGGTAAATAATGTATTGTCACTTGTAAACGGTGACCTGTTA 3480

AMH-2_1 CTGGCTTTGGAACGCGCTGTGGTAAATAATGTATTGTCACTTGTAAACGGTGACCTGTTA 3480

AMH-2_4 CTGGCTTTGGAACGCGCTGTGGTAAATAATGTATTGTCACTTGTAAACGGTGACCTGTTA 3480

AMH-3_1 CTGGCTTTGGAACGCGCAGTGGTAAATAATGTTTTGTCACTTGTAAACGGTGACCCGTTA 3480

AMH-3_13 CTGGCTTTGGAACGCGCAGTGGTAAATAATGTTTTGTCACTTGTAAACGGTGACCCGTTA 3480

AMH-1_7 CTGGCTTTGGAACGCGCAGTGGTAAATAATGTTTTGTCACTTGTAAACGGTGACCCGTTA 3480

AMH-1_11 CTGGCTTTGGAACGCGCAGTGGTAAATAATGTTTTGTCACTTGTAAACGGTGACCCGTTA 3480

KMP-8_3 CTGGCTTTGGAACGCGCAGTGGTAAATAATGTTTTGTCACTTGTAAACGGTGACCCGTTA 3477

AMH-1_2 CTGGCTTTGGAACGCGCAGTGGTAAATAATGTTTTGTCACTTGTAAACGGTGACCCGTTA 3480

AY198374.1 CTGGCTTTGGAACGCGCAGTGGTAAATAATGTTTTGTCACTTGTAAACGGTGACCCGTTA 3480

*****************:**************:********************** ****

KMP-8_35 GACAGGTTACAAGCAATTGACGACGATGGTCTTGATGCTGGCGTGGTGACTTTCGATATT 3537

KMP-8_5 GACAGGTTACAAGCAATTGACGACGATGGTCTTGATGCTGGCGTGGTGACTTTCGATATT 3537

KMP-8_24 GACAGGTTACAAGCAATTGACGACGATGGTCTTGATGCTGGCGTGGTGACTTTCGATATT 3537

KMP-8_46 GACAGGTTACAAGCAATTGACGACGATGGTCTTGATGCTGGCGTGGTGACTTTCGATATT 3537

AMH-3_16 GACAGGATACAAGCAGTTGACGACGATGGTCTCGATGCTGGCGTGGTGACTTTCGATATT 3537

AMH-2_5 GACAGGATACAAGCAATTGACGACGATGGTCTCGATGCTGGCGTGGTGACTTTCGATATT 3540

AMH-2_1 GACAGGATACAAGCAATTGACGACGATGGTCTCGATGCTGGCGTGGTGACTTTCGATATT 3540

AMH-2_4 GACAGGATACAAGCAATTGACGACGATGGTCTCGATGCTGGCGTGGTGACTTTCGATATT 3540

AMH-3_1 GACAGGATACAAGCAATTGACGACGATGGTCTTGATGCTGGCGTGGTGACTTTCGATATT 3540

AMH-3_13 GACAGGATACAAGCAATTGACGACGATGGTCTTGATGCTGGCGTGGTGACTTTCGATATT 3540

AMH-1_7 GACAGGATACAAGCAATTGACGACGATGGTCTTGATGCTGGCGTGGTGACTTTCGATATT 3540

AMH-1_11 GACAGGATGCAAGCAATTGACGACGATGGTCTTGATGCTGGCGTGGTGACTTTCGATATT 3540

KMP-8_3 GACAGGATACAAGCAATTGACGACGATGGTCTTGATGCTGGCGTGGTGACTTTCGATATT 3537

AMH-1_2 GACAGGATACAAGCAATTGACGACGATGGTCTTGATGCTGGCGTGGCGACTTTCGATATT 3540

AY198374.1 GACAGGATACAAGCAATTGACGACGATGGTCTTGATGCTGGCGTGGTGACTTTCGATATT 3540

******:*.******.**************** ************* *************

KMP-8_35 GTTGGAGATGCTGATGCATCAAACTACTTCAGAGTAAATAATGATGGCGACAACTTTGGA 3597

KMP-8_5 GTTGGAGATGCTGATGCATCAAACTACTTCAGAGTAAATAATGATGGCGACAACTTTGGA 3597

KMP-8_24 GTTGGAGGTGCTGATGCATCAAACTACTTCAGAGTAAATAATGATGGCGACAACTTTGGA 3597

KMP-8_46 GTTGGAGATGCTGATGCATCAAACTACTTCAGAGTAAATAATGATGGCGACAACTTTGGA 3597

AMH-3_16 GTTGGAGATGCTGATGCATCAAACTACTTCAGAGTAAATAATGATGGCGACAATTTTGGA 3597

AMH-2_5 GTTGGAGATGCTGATGCATCAAACTACTTCAGAGTAAATAATGATGGCGACAACTTTGGA 3600

AMH-2_1 GTTGGAGATGCTGATGCATCAAACTACTTCAGAGTAAATAATGATGGCGACAACTTTGGA 3600

AMH-2_4 GTTGGAGATGCTGATGCATCAAACTACTTCAGAGTAAATAATGATGGCGACAACTTTGGA 3600

AMH-3_1 GTTGGAGATGCTGATGCATCAAACTACTTCAGAGTAAATAATGATGGCGACAACTTTGGA 3600

AMH-3_13 GTTGGAGATGCTGATGCATCAAACTACTTCAGAGTAAATAATGATGGCGACAACTTTGGA 3600

AMH-1_7 GTTGGAGATGCTGATGCATCAAACTACTTCAGAGTAAATAATGATGGCGACAACTTTGGA 3600

AMH-1_11 GTTGGAGATGCTGATGCATCAAACTACTTCAGAGTAAATAATGATGGCGACAACTTTGGA 3600

KMP-8_3 GTTGGAGATGCTGATGCATCAAACTACTTCAGAGTAAATAATGATGGCGACAACTTTGGA 3597

AMH-1_2 GTTGGAGATGCTGATGCATCAAACTACTTCAGAGTAAATAATGATGGCGACAACTTTGGA 3600

AY198374.1 GTTGGAGATGCTGATGCATCAAACTACTTCAGAGTAAATAATGATGGCGACAACTTTGGA 3600

*******.********************************************* ******

KMP-8_35 ACCTTGTTGCTGACACAGGCGCTTCCTGAGGAAGGCAAGGAATTTGGGGTTACCATCCGG 3657

KMP-8_5 ACCTTGTTGCTGACACAGGCGCTTCCTGAGGAAGGCAAGGAATTTGAGGTTACCATCCGG 3657

KMP-8_24 ACCTTGTTGCTGACACAGGCGCTTCCTGAGGAAGGCAAGGAATTTGAGGTTACCATCCGG 3657

KMP-8_46 ACCTTGTTGCTGACACAGGCGCTTCCTGAGGAAGGCAAGGAATTTGAGGTTACCATCCGG 3657

AMH-3_16 ACCTTGTTGCTGACACAGGCGCTTCCTGAGGAAGGCAAGGAATTTGAGGTTACCATCCGG 3657

AMH-2_5 ACCTTGTTGCTGACACAGGCGCTTCCTGAGGAAGGCAAGGAATTTGAGGTTACCATCCGG 3660

AMH-2_1 ACCTTGTTGCTGACACAGGCGCTTCCTGAGGAAGGCAAGGAATTTGAGGTTACCATCCGG 3660

AMH-2_4 ACCTTGTTGCTGACACAGGCGCTTCCTGAGGAAGGCAAGGAATTTGAGGTTACCATCCGG 3660

AMH-3_1 ACCTTGTTGCTGACACAGGCGCTTCCTGAGGAAGGCAAGGAATTTGAGGTTACCATCCGG 3660

AMH-3_13 ACCTTGTTGCTGACACAGGCGCTTCCTGAGGAAGGCAAGGAATTTGAGGTTACCATCCGG 3660

AMH-1_7 ACCTTGTTGCTGACACAGGCGCTTCCTGAGGAAGGCAAGGAATTTGAGGTTACCATCCGG 3660

AMH-1_11 ACCTTGTTGCTGACACAGGCGCTTCCCGAGGAAGGCAAGGAATTTGAGGTTACCATCCGG 3660

KMP-8_3 ACCTTGTTGCTGACACAGGCGCTTCCTGAGGAAGGCAAGGAATTTGAGGTTACCATCCGG 3657

AMH-1_2 ACCTTGTTGCTGACACAGGCGCTTCCTGAGGAAGGCAAGGAATTTGAGGTTACCATCCGG 3660

AY198374.1 ACCTTGTTGCTGACACAGGCGCTTCCTGAGGAAGGCAAGGAATTTGAGGTTACCATCCGG 3660

************************** *******************.*************

KMP-8_35 GCTACAGACGGCGGAATAGAACCTCGATCATATTCAACAGACTCCACTATAACAGTGCTC 3717

KMP-8_5 GCTACAGACGGCGGAATAGAACCTCGATCATATTCAACAGACTCCACTATAACAGTGCTC 3717

KMP-8_24 GCTACAGACGGCGGAATAGAACCTCGATCATATTCAACAGACTCCACTATAACAGTGCTC 3717

KMP-8_46 GCTACAGACGGCGGAATAGAACCTCGATCATATTCAACAGACTCCACTATAACAGTGCTC 3717

AMH-3_16 GCTACAGACGGCGGAACGGAACCTCGATCATATTCAACAGACTCTACTATAACAGTCCTC 3717

AMH-2_5 GCTACAGACGGCGGAACAGAACCTCGATCATATTCAACAGACTCCACTATAACAGTCCTC 3720

AMH-2_1 GCTACAGACGGCGGAACAGAACCTCGATCATATTCAACAGACTCCACTATAACAGTCCTC 3720

AMH-2_4 GCTACAGACGGCGGAACAGAACCTCGATCATATTCAACAGACTCCACTATAACAGTCCTC 3720

AMH-3_1 GCTACAGGCGGCGGAACGGAACCTCGATCATATTCAACAGACTCCACTATAACAGTGCTC 3720

AMH-3_13 GCTACAGGCGGCGGAACGGAACCTCGATCATATTCAACAGACTCCACTATAACAGTGCTC 3720

AMH-1_7 GCTACAGACGGCGGAACAGAACCTCGATCATATTCAACAGACTCCACTATAACAGTGCTC 3720

AMH-1_11 GCTACAGACGGCGGAACAGAACCTCGATCATATTCAACAGACTCCACTATAACAGTGCTC 3720

KMP-8_3 GCTACAGACGGCGGAACAGAACCTCGATCATATTCAACAGACTCCACTATAACAGTGCTC 3717

AMH-1_2 GCTACAGACGGCGGAACGGAACCTCGATCATATTCAACAGACTCCACTATAACAGTGCTC 3720

AY198374.1 GCTACAGACGGCGGAACAGAACCTCGATCATATTCAACAGACTCCACTATAACAGTGCTC 3720

*******.******** .************************** *********** ***

KMP-8_35 TTCGTTCCGACTTTGGGTGATCCGATCTTTCAAGATAACACTTACTCAGTAGCATTCTTT 3777

KMP-8_5 TTCGTTCCGACTTTGGGTGATCCGATCTTTCAAGATAACACTTACTCAGTAGCGTTCTTT 3777

KMP-8_24 TTCGTTCCGACTTTGGGTGATCCGATCTTTCAAGATAACACTTACTCAGTAGCATTCTTT 3777

KMP-8_46 TTCGTTCCGACTTTGGGTGATCCGATCTTTCAAGATAACACTTACTCAGTAGCATTCTTT 3777

AMH-3_16 TTCGTTCCGACTTTGGGTGATCCGATCTTTCAAGATAACACTTACTCAGTAGCATTCTTT 3777

AMH-2_5 TTCGTTCCGACTTTGGGTGATCCGATCTTTCAAGATAACACTTACTCAGTAGCATTCTTT 3780

AMH-2_1 TTCGTTCCGACTTTGGGTGATCCGATCTTTCAAGATAACACTTACTCAGTAGCATTCTTT 3780

AMH-2_4 TTCGTTCCGACTTTGGGTGATCCGATCTTTCAAGATAACACTTACTCAGTAGCATTCTTT 3780

AMH-3_1 TTCGTTCCGACTTTGGGTGATCCGATATTTCAAGATAACACTTACTCAGTAGCATTCTTT 3780

AMH-3_13 TTCGTTCCGACTTTGGGTGATCCGATATTTCAAGATAACACTTACTCAGTAGCATTCTTT 3780

AMH-1_7 TTCGTTCCGACTTTGGGTGATCCGATCTTTCAAGATAACACTTACTCGGTAGCATTCTTT 3780

AMH-1_11 TTCGTTCCGACTTTGGGTGATCCGATCTTTCAAGATAACACTTACTCAGTAGCATTCTTT 3780

KMP-8_3 TTCGTTCCGACTTTGGGTGATCCGATCTTTCAAGATAACACTTACTCAGTAGCATTCTTT 3777

AMH-1_2 TTCGTTCCGACTTTGGGTGATCCGATCTTTCAAGATAACACTTACTCAGTAGCATTCTTT 3780

AY198374.1 TTCGTTCCGACTTTGGGTGATCCGATCTTTCAAGATAACACTTACTCAGTAGCATTCTTT 3780

**************************.********************.*****.******

KMP-8_35 GAAAAAGAGGTTGGCTTGACTGAGAGGTTCTCGCTCCCACATGCAGAGGACCCTAAGAAC 3837

KMP-8_5 GAAAAAGAGGTTGGCTTGACTGAGAGGTTCTCGCTCCCACATGCAGAGGACCCTAGGAAC 3837

KMP-8_24 GAAAAAGAGGTTGGCTTGACTGAGAGGTTCTCGCTCCCACATGCAGAGGGCCCTAAGAAC 3837

KMP-8_46 GAAAAAGAGGTTGGCTTGACTGAGAGGTTCTCGCTCCCACATGCAGAGGACCCTAAGAAC 3837

AMH-3_16 GAAAAAGAGGTTGGCTTGACTGAGAGGTTCACGCTCCCACTAGCAGAGGACCCTAAGAAC 3837

AMH-2_5 GAAAAAGAGGTTGGCTTGACTGAGAGGTTCTCGCTCCCACATGCAGAGGACCCTAAGAAC 3840

AMH-2_1 GAAAAAGAGGTTGGCTTGACTGAGAGGTTCTCGCTCCCACATGCAGAGGACCCTAAGAAC 3840

AMH-2_4 GAAAAAGAGGTTGGCTTGACTGAGAGGTTCTCGCTCCCACATGCAGAGGACCCTAAGAAC 3840

AMH-3_1 GAAAAAGAGGTTGGCTTGACTGAGAGGTTCTCGCTCCCACTAGCAGAGGACCCTAAGAAC 3840

AMH-3_13 GAAAAAGAGGTTGGCTTGACTGAGAGGTTCTCGCTCCCACTAGCAGAGGACCCTAAGAAC 3840

AMH-1_7 GAAAAAGAGGTTGGCTTGACTGAGAGGTTCTCGCTCCCACATGCAGAGGACCCTAAGAAC 3840

AMH-1_11 GAAAAAGAGGTTGGCTTGACTGAGAGGTTCTCGCTCCCACATGCAGAGGACCCTAAGAAC 3840

KMP-8_3 GAAAAAGAGGTTGGCTTGACTGAGAGGTTCTCGCTCCCACATGCAGGGGACCCTAAGAAC 3837

AMH-1_2 GAAAAAGAGGTTGGCTTGACTGAGAGGTTCTCGCTCCCACATGCAGAGGACCCTAAGAAC 3840

AY198374.1 GAAAAAGAGGTTGGCTTGACTGAGAGGTTCTCGCTCCCACATGCAGAGGACCCTAAGAAC 3840

******************************:*********::****.**.*****.****

KMP-8_35 AAACTCTGCACTGACGACTGTCACGATATTTACTACAGGATCTTTGGTGGTGTGGATTAC 3897

KMP-8_5 AAACTCTGCACTGACGACTGTCACGATATTTACTACAGGATCTTTGGTGGTGTGGATTAC 3897

KMP-8_24 AAACTCTGCACTGACGACTGTCACGATATTTACTACAGGATCTTTGGTGGTGTGGATTAC 3897

KMP-8_46 AAACTCTGCACTGACGACTGTCACGATATTTACTACAGGATCTTTGGTGGTGTGGATTAC 3897

AMH-3_16 AAACTTTGCACTGACGACTGTCACGATATTTACTACAGGATCTTTGGTGGTGTGGATTAC 3897

AMH-2_5 AAACTCTGCACTGACGACTGTCACGATATTTACTACAGGATCTTTGGTGGTGTGGATTAC 3900

AMH-2_1 AAACTCTGCACTGACGACTGTCACGATATTTACTACAGGATCTTTGGTGGTGTGGATTAC 3900

AMH-2_4 AAACTCTGCACTGACGACTGTCACGATATTTACTACAGGATCTTTGGTGGTGTGGATTAC 3900

AMH-3_1 AAACTCTGCACTGACGACTGTCACGATATTTACTACAGGATCTTTGGTGGTGTGGATTAC 3900

AMH-3_13 AAACTCTGCACTGACGACTGTCACGATATTTACTACAGGATCTTTGGTGGTGCGGATTAC 3900

AMH-1_7 AAACTCTGCACTGACGACTGTCACGATATTTACTACAGGATCTTTGGTAGTGTGGATTAC 3900

AMH-1_11 AAACTCTGCACTGACGACTGTCACGATATTTACTACAGGATCTTTGGTGGTGTGGATTAC 3900

KMP-8_3 AAACTCTGCACTGACGACTGTCACGATATTTACTACAGGATCTTTGGTGGTGTGGATTAC 3897

AMH-1_2 AAACTCTGCACTGACGACTGTCACGATATTTACTACAGGATCTTTGGTGGTGTGGATTAC 3900

AY198374.1 AAACTCTGCACTGACGACTGTCACGATATTTACTACAGGATCTTTGGTGGTGTGGATTAC 3900

***** ******************************************.*** *******

KMP-8_35 GAGCCATTTGACCTGGACCCGGTGACGAACGTGATCTTCCTGAAATCAGAACTAGACCGG 3957

KMP-8_5 GAGCCATTTGACCTGGACCCGGTGACGAACGTGATCTTCCTGAAATCAGAACTAGACCGG 3957

KMP-8_24 GAGCCATTTGACCTGGACCCGGTGACGAACGTGATCTTCCTGAAATCAGAACTAGACCGG 3957

KMP-8_46 GAGCCATTTGACCTGGACCCGGTGACGAACGTGATCTTCCTGAAATCAGAACTAGACCGG 3957

AMH-3_16 GAGCCATTTGACCTGGACCCGGTGACGAACGTGATCTTCCTGAAATCAGAACTAGACCGG 3957

AMH-2_5 GAGCCATTTGACCTGGACCCGGTGACGAACGTGATCTTCCTGAAATCAGAACTGGACCGA 3960

AMH-2_1 GAGCCATTTGACCTGGACCCGGTGACGAACGTGATCTTCCTGAAATCAGAACTGGACCGA 3960

AMH-2_4 GAGCCATTTGACCTGGACCCGGTGACGAACGTGATCTTCCTGAAATCAGAACTGGACCGA 3960

AMH-3_1 GAGCCATTTGACCTGGACCCGGTGACGAACGTGATCTTCCTGAAATCAGAACTAGACCGG 3960

AMH-3_13 GAGCCATTTGACCTGGACCCGGTGACGAACGTGATCTTCCTGAAATCAGAACTAGACCGG 3960

AMH-1_7 GAGCCATTTGACCTGGACCCGGTGACGAACGTGATCTTCCTGAAATCAGAACTAGACCGG 3960

AMH-1_11 GAGCCATTTGACCTGGACCCGGTGACGAACGTGATCTTCCTGAAATCAGAACTAGACCGG 3960

KMP-8_3 GAGCCATTTGACCTGGACCCGGTGACGAACGTGATCTTCCTGAAATCAGAACTAGACCGG 3957

AMH-1_2 GAGCCATTTGACCTGGACCCGGTGACGAACGTGATCTTCCTGAAATCAGAACTAGACCGG 3960

AY198374.1 GAGCCATTTGACCTGGACCCGGTGACGAACGTGATCTTCCTGAAATCAGAACTAGACCGG 3960

*****************************************************.*****.

KMP-8_35 GAGACCACTGCCACGCATGTGGTGCAAGTGGCAGCCAGCAATTCGCCCACAGGAGGCGGA 4017

KMP-8_5 GAGACCACTGCCACGCATGTGGTGCAAGTGGCAGCCAGTAATTCGCCCACAGGAGGCGGA 4017

KMP-8_24 GAGACCACTGCCACGCATGTGGTGCAAGTGGCAGCCAGTAATTCGCCCACAGGAGGCGGA 4017

KMP-8_46 GAGACCACTGCCACGCATGTGGTGCAAGTGGCAGCCAGTAATTCGCCCACAGGAGGCGGA 4017

AMH-3_16 GATACCACTGCCACGCATGTGGTGCAAGTGGCAGCCAGTAATTCGCCCACAGGAGGCGGA 4017

AMH-2_5 GAGACCACTGCCACGCATGTGGTTCAAGTGGCTGCCAGTAATTCGCCCACAGGAGGCGGA 4020

AMH-2_1 GAGACCACTGCCACGCATGTGGTTCAAGTGGCTGCCAGTAATTCGCCCACAGGAGGCGGA 4020

AMH-2_4 GAGACCACTGCCACGCAGGTGGTTCAAGTGGCTGCCAGTAATTCGCCCACAGGAGGCGGA 4020

AMH-3_1 GAGACCACTGCCACGCATGTGGTGCAAGTGGCAGCCAGTAATTCGCCCACAGGAGGCGGA 4020

AMH-3_13 GAGACCACTGCCACGCATGTGGTGCAAGTGGCAGCCAGTAATTCGCCCACAGGAGGCGGA 4020

AMH-1_7 GAGACCACTGCTACGCATGTGGTGCAAGTGGCAGCCAGTAATTCGCCCACAGGAGGCGGA 4020

AMH-1_11 GAGACCACTGCTACGCATGTGGTGCAAGTGGCAGCCAGTAATTCGCCCACAGGAGGCGGA 4020

KMP-8_3 GAGACCACTGCTACGCATGTGGTGCAAGTGGCAGCCAGTAATTCGCCCACAGGAGGCGGA 4017

AMH-1_2 GAGACCACTGCTACGCATGTGGTGCAAGTGGCAGCCAGTAATTCGCCCACAGGAGGCGGA 4020

AY198374.1 GAGACCACTGCTACGCATGTGGTGCAAGTGGCAGCCAGTAATTCGCCCACAGGAGGCGGA 4020

** ******** ***** ***** ********:***** *********************

KMP-8_35 ATACCACTCCCTGGGTCTCTTTTCACCGTCACTGTCACTGTACGAGAAGCGGATCCACGG 4077

KMP-8_5 ATACCACTCCCTGGGTCTCTTCTCACCGTCACTGTCACTGTACGAGAAGCGGATCCACGG 4077

KMP-8_24 ATACCACTCCCTGGGTCTCTTCTCACCGTCACTGTCACTGCACGAGAAGCGGATCCACGG 4077

KMP-8_46 ATACCACTCCCTGGGTCTCTTCTCACCGTCACTGTCACTGTACGAGAAGCGGATCCACGG 4077

AMH-3_16 ATACCACTCCCTGGGTCTCTTCTCACCGTCACTGTCACTGTACGAGAAGCGGATCCACGG 4077

AMH-2_5 ATACCACTCCCTGGGTCTCTTCTCACCGTCACTGTCACTGTACGAGAAGCGGATCCACGG 4080

AMH-2_1 ATACCACTCCCTGGGTCTCTTCTCACCGTCACTGTCACTGTACGAGAAGCGGATCCACGG 4080

AMH-2_4 ATACCACTCCCTGGGTCTCTTCTCACCGTCACTGTCACTGTACGAGAAGCGGATCCACGG 4080

AMH-3_1 ATACCACTCCCTGGGTCTCTTCTCACCGTCACTGTCACTGTACGAGAAGCGGATCCACGG 4080

AMH-3_13 ATACCACTCCCTGGGTCTCTTCTCACCGTCACTGTCACTGTACGAGAAGCGGATCCACGG 4080

AMH-1_7 ATACCACTCCCTGGGTCTCTTCTCACCGTCACTGTCACTGTACGAGAAGCGGATCCACGG 4080

AMH-1_11 ATACCACTCCCTGGGTCTCTTCTCACCGTCACTGTCACTGTACGAGAAGCGGATCCACGG 4080

KMP-8_3 ATACCACTCCCTGGGTCTCTTCTCACCGTCACTGTCACTGTACGAGAAGCGGATCCACGG 4077

AMH-1_2 ATACCACTCCCTGGGTCTCTTCTCACCGTCACTGTCACTGTACGAGAAGCGGATCCACGG 4080

AY198374.1 ATACCACTCCCTGGGTCTCTTCTCACCGTCACTGTCACTGTACGAGAAGCGGATCCACGG 4080

********************* ****************** *******************

KMP-8_35 CCTGTGTTCGAGCAGCGTCTGTACACGGCTGGCATTTCCACTTCCGATAACATCAACAGG 4137

KMP-8_5 CCTGTGTTCGAGCAGCGTCTGTACACGGCTGGCATTTCCACTTCCGATAACATCAACAGG 4137

KMP-8_24 CCTGTGTTCGAGCAGCGTCTGTACACGGCTGGCATTTCCACTTCCGATAACATCAACAGG 4137

KMP-8_46 CCTGTGTTCGAGCAGCGTCTGTACACGGCTGGCATTTCCACTTCCGATAACATCAACAGG 4137

AMH-3_16 CCTGTGTTCGAGCAGCGTCTGTACACGGCTGGCATTTCCACTTCCGATAACATCAACAGG 4137

AMH-2_5 CCTGTGTTCGAGCAGCGTCTGTACACGGCTGGCATTTCCACTTCCGATAACATCAACAGA 4140

AMH-2_1 CCTGTGTTCGAGCAGCGTCTGTACACGGCTGGCATTTCCACTTCCGATAACATCAACAGA 4140

AMH-2_4 CCTGTGTTCGAGCAGCGTCTGTACACGGCTGGCATTTCCACTTCCGATAACATCAACAGA 4140

AMH-3_1 CCTGTGTTCGAGCAGCGTCTGTACACGGCTGGCATTTCCACTTCCGATAACATCAACAGG 4140

AMH-3_13 CCTGTGTTCGAGCAGCGTCTGTACACGGCTGGCATTTCCACTTCCGATAACATCAACAGG 4140

AMH-1_7 CCTGTGTTCGAGCAGCGTCTGTACACGGCTGACATTTCCACTTCCGATAACATCAACAGG 4140

AMH-1_11 CCTGTGTTCGAGCAGCGTCTGTACACGGCTGGCATTTCCACTTCCGATAACATCAACAGG 4140

KMP-8_3 CCTGTGTTCGGGCAGCGTCTGTACACGGCTGGCATTTCCACTTCCGATAACATCAACAGG 4137

AMH-1_2 CCTGTGTTCGAGCAGCGTCTGTACACGGCTGGCATTTCCACTTCCGATAACATCAACAGG 4140

AY198374.1 CCTGTGTTCGAGCAGCGTCTGTACACGGCTGGCATTTCCACTTCCGATAACATCAACAGG 4140

**********.********************.***************************.

KMP-8_35 GAACTACTCACCGTTCGTGCAACTCATTCCGAAAACGCACAATTGACATATACCATCGAA 4197

KMP-8_5 GAACTACTCACCGTTCGTGCAACTCATTCCGAAAACGCACAATTGACATATACCATCGAA 4197

KMP-8_24 GAACTACTCACCGTTCGTGCAACTCATTCCGAAAACGCACAATTGACATATACCATCGAA 4197

KMP-8_46 GAACTACTCACCGTTCGTGCAACTCATTCCGAAAACGCACAATTGACATATACCATCGAA 4197

AMH-3_16 GAACTACTCACCGTTCGTGCAACTCATTCCGAAAACGCACAATTGACATATACTATCGAA 4197

AMH-2_5 GAACTACTCACCGTTCGTGCGACTCATTCCGAAAACGCACAATTGACATATACCATCGAA 4200

AMH-2_1 GAACTACTCACCGTTCGTGCAACTCATTCCGAAAACGCACAATTGACATATACCATCGAA 4200

AMH-2_4 GAACTACTCACCGTTCGTGCAACTCATTCCGAAAACGCACAATTGACATATACCATCGAA 4200

AMH-3_1 GAACTACTCACCGTTCGTGCAACTCATTCCGAAAACGCACAATTGACATATACCATCGAA 4200

AMH-3_13 GAACTACTCACCGTTCGTGCAACTCATTCCGAAAACGCACAATTGACATATACCATCGAA 4200

AMH-1_7 GGACTACTCACCGTTCGTGCAACTCATTCCGAAAACGCACAATTGACATATACCATCGAA 4200

AMH-1_11 GAACTACTCACCGTTCGTGCAACTCATTCCGAAAACGCACAATTGACATATACCATCGAA 4200

KMP-8_3 GAACTACTCACCGTTCGTGCAACTCATTCCGAAAACGCACAATTGACATATACCATCGAA 4197

AMH-1_2 GAACTACTCACCGTTCGTGCAACTCATTCCGAAAACGCACAATTGACATATACCATCGAA 4200

AY198374.1 GAACTACTCACCGTTCGTGCAACTCATTCCGAAAACGCACAATTGACATATACCATCGAA 4200

*.******************.******************************** ******

KMP-8_35 GATGGTTCTATGGTGGTGGACTCCACTCTGGAAGCCGTCAAGGACTCGGCGTTTCATCTG 4257

KMP-8_5 GATGGTTCTATGGTGGTGGACTCCACTCTGGAAGCCGTCAAGGACTCGGCGTTTCATCTG 4257

KMP-8_24 GGTGGTTCCATGGTGGTGGACTCCACTCTGGAAGCCGTCAAGGACTCGGCGTTTCATCTG 4257

KMP-8_46 GATGGTTCTATGGTGGTGGACTCCACTCTGGAAGCCGTCAAGGACTCGGCGTTTCATCTG 4257

AMH-3_16 GACGGTTCTATGGTGGTGGACTCCACTCTGGAAGCCGTCAAGGACTCGGCGTTCCATCTG 4257

AMH-2_5 GATGGTTCTATGGTGGTGGACTCCACTCTGGAAGCCGTCAAGGACTCGGCGTTCCATCTG 4260

AMH-2_1 AATGGTTCTATGGTGGTGGACTCCACTCTGGAAGCCGTCAAGGACTCGGCGTTCCATCTG 4260

AMH-2_4 GATGGTTCTATGGTGGTGGACTCCACTCTGGAAGCCGTCAAGGACTCGGCGTTCCATCTG 4260

AMH-3_1 GATGGTTCTATGGTGGTGGACTCCACTCTGGAAGCCGTCAAGGACTCGGCGTTCCATCTG 4260

AMH-3_13 GATGGTTCTATGGTGGTGGACTCCACTCTGGAAGCCGTCAAGGACTCGGCGTTCCATCTG 4260

AMH-1_7 GACGGTTCTATGGCGGTGGACTCCACTCTGGAAGCCGTCAAGGACTCGGCGTTCCATCTG 4260

AMH-1_11 GACGGTTCTATGGCGGTGGACTCCACTCTGGAAGCCGTCAAGGACTCGGCGCTCCATCTG 4260

KMP-8_3 GACGGTTCTATGGCGGTGGACACCACTCTGGAAGCCGTCAAGGACTCGGCGTTCCATCTG 4257

AMH-1_2 GACGGTTCTATGGCGGTGGACTCCACTCTGGAAGCCGTCAAGGACTCGGCGTTCCATCTG 4260

AY198374.1 GACGGTTCTATGGCGGTGGACTCCACTCTGGAAGCCGTCAAGGACTCGGCGTTCCATCTG 4260

.. ***** **** *******:***************************** * ******

KMP-8_35 AACGCGCAGACCGGCGTCCTCATACTGAGGATACAACCTACTGCCAGCATGCAGGGCATG 4317

KMP-8_5 AACGCGCAGACCGGCGTCCTCATACTGAGGATACAACCTACTGCCAGCATGCAGGGCATG 4317

KMP-8_24 AACGCGCAGACCGGCGTCCTCATACTGAGGATACAACCTACTGCCAGCATGCAGGGCATG 4317

KMP-8_46 AACGCGCAGACCGGCGTCCTCATACTGAGGATACAACCTACTGCCAGCATGCAGGGCATG 4317

AMH-3_16 AACGCGCAGACCGGCGTCCTCATACTGAGGATACAACCTACTGCCAGCATGCAGGGCATG 4317

AMH-2_5 AACGCGCAGACCGGCGTCCTCATACTGAGGATACAACCTACTGCCAGCATGCAGGGCATG 4320

AMH-2_1 AACGCGCAGACCGGCGTCCTCATACTGAGGATACAACCTACTGCCAGCATGCAGGGCATG 4320

AMH-2_4 AACGCGCAGACCGGCGTCCTCATACTGAGGATACAACCTACTGCCAGCATGCAGGGCATG 4320

AMH-3_1 AACGCGCAGACCGGCGTCCTCATACTGAGGATACAACCTACTGCCAGCATGCAGGGCATG 4320

AMH-3_13 AACGCGCAGACCGGCGTCCTCATACTGAGGATACAACCTACTGCCAGCATGCAGGGCATG 4320

AMH-1_7 AACGCGCAGACCGGCGTCCTCATACTGAGGATACAACCTACTGCCAGCATGCAGGGCATG 4320

AMH-1_11 AACGCGCAGACCGGCGTCCTCATACTGAGGATACAACCTACTGCCAGCATGCAGGGCATG 4320

KMP-8_3 AACGCGCAGACCGGCGTCCTCATACTGAGGATACAACCTACTGCCAGCATGCAGGGCATG 4317

AMH-1_2 AACGCGCAGACCGGCGTCCTCATACTGAGGATACAACCTACTGCCAGCATGCAGGGCATG 4320

AY198374.1 AACGCGCAGACCGGCGTCCTCATACTGAGGATACAACCTACTGCCAGCATGCAGGGCATG 4320

************************************************************

KMP-8_35 TTCGAGTTCAACGTCATCGCTACTGATCCAGATGAGAAGACAGATACGGCAGAGGTGAGA 4377

KMP-8_5 TTTGAGTTCAACGTCATCGCTACTGACCCAGATGAGAAGACAGATACGGCAGAGGTGAAA 4377

KMP-8_24 TTTGAGTTCAACGTCATCGCTACTGACCCAGATGAGAAGACAGATACGGCAGAGGTGAAA 4377

KMP-8_46 TTTGAGTTCAACGTCATCGCTACTGACCCAGATGAGAAGACAGATACGGCAGAGGTGAAA 4377

AMH-3_16 TTCGAGTTCAACGTCATCGCTACTGACCCAGATGAGAAGACAGATACGGCAGAGGTGAAA 4377

AMH-2_5 TTTGAGTTCAACGTCATCGCTACTGACCCAGATGAGAAGACAGATACGGCAGAGGTGAAA 4380

AMH-2_1 TTTGAGTTCAACGTCATCGCTACTGACCCAGATGAGAAGACAGATACGGCAGAGGTGAAA 4380

AMH-2_4 TTTGAGTTCAACGTCATCGCTACTGACCCAGATGAGAGGACAGATACGGCAGAGGTGAAA 4380

AMH-3_1 TTTGAGTTCAACGTCATCGCTACTGACCCAGATGAGAAGACAGATACGGCAGAGGTGAAA 4380

AMH-3_13 TTTGAGTTCAACGTCATCGCTACTGACCCAGATGAGAAGACAGATACGGCAGAGGTGAAA 4380

AMH-1_7 TTCGAGTTCAACGTCATCGCTACTGATCCAGATGAGAAGACAGATGCGGCAGAGGTGAAA 4380

AMH-1_11 TTCGAGTTCAACGTCGTCGCTACTGATCCAGATGAGAAGACAGATACGGCAGAGGTGAAA 4380

KMP-8_3 TTCGAGTTCAACGTCATCGCTACTGATCCAGATGAGAAGACAGATACGGCAGGGGTGAAA 4377

AMH-1_2 TTTGAGTTCAACGTCATCGCTACTGACCCAGATGAGAAGACAGATACGGCAGAGGTGAAA 4380

AY198374.1 TTCGAGTTCAACGTCATCGCTACTGATCCAGATGAGAAGACAGATACGGCAGAGGTGAAA 4380

** ************.********** **********.*******.******.*****.*

KMP-8_35 GTCTACCTCATTTCATCCCAAAATAGGGTGTCCTTCATATTCCTGAACGATGTGGAGACG 4437

KMP-8_5 GTCTACCTCATTTCATCCCAAAATAGGGTGTCCTTCATATTCCTGAACGATGTGGAGACG 4437

KMP-8_24 GTCTACCTCATTTCATCCCAAAATAGGGTGTCCTTCATATTCCTGAACGATGTGGAGACG 4437

KMP-8_46 GTCTACCTCATTTCATCCCAAAATAGGGTGTCCTTCATATTCCTGAACGATGTGGAGACG 4437

AMH-3_16 GTCTACCTCATTTCATCCCAAAATAGGGTGTCCTTCATATTCCTGAACGATGTGGAGACG 4437

AMH-2_5 GTCTACCTCATTTCATCCCAAAATAGGGTGTCCTTCATATTCCTGAACGATGTGGAGACT 4440

AMH-2_1 GTCTACCTCATTTCATCCCAAAATAGGGTGTCCTTCATATTCCTGAACGATGTGGAGACT 4440

AMH-2_4 GTCTACCTCATTTCATCCCAAAATAGGGTGTCCTTCATATTCCTGAACGATGTGGAGACT 4440

AMH-3_1 GTCTACCTCATTTCATCCCAAAATAGGGTGTCCTTCATATTCCTGAACGATGTGGAGACG 4440

AMH-3_13 GTCTACCTCATTTCGTCCCAAAATAGGGTGTCCTTCATATTCCTGAACGATGTGGAGACG 4440

AMH-1_7 GTCTACCTCATTTCATCCCAAAATAGGGTGTCCTTCATATTCCTGAACGATGTGGAGACG 4440

AMH-1_11 GTCTACCTCATTTCATCCCAAAATAGGGTGTCCTTCATATTCCTGAACGATGTGGAGACG 4440

KMP-8_3 GTCTACCTCATTTCATCCCAAAATAGGGTGTCCTTCATATTCCTGAACGATGTGGAGACG 4437

AMH-1_2 GTCTACCTCATTTCATCCCAAAATGGGGTGTCCTTCATATTCCTGAACGATGTGGAGACG 4440

AY198374.1 GTCTACCTCATTTCATCCCAAAATAGGGTGTCCTTCATATTCCTGAACGATGTGGAGACG 4440

**************.*********.**********************************

KMP-8_35 GTTGAGAGTAACAGAGACTTTATCGCAGAAACGTTCAGCGTTGGCTTCAACATGACCTGC 4497

KMP-8_5 GTTGAGAGTAACAGAGACTTTATCGCAGAAACGTTCAGCGTTGGCTTCAACATGACCTGC 4497

KMP-8_24 GTTGAGAGTAACAGAGACTTTATCGCAGAAACGTTCAGCGTTGGCTTCAACATGACCTGC 4497

KMP-8_46 GTTGAGAGTAACAGAGACTTTATCGCAGAAACGTTCAGCGTTGGCTTCAACATGACCTGC 4497

AMH-3_16 GTTGAGAGTAACAGAGACTTTATCGCAGAAACGTTCAGCGTTGGCTTCAACATGACCTGC 4497

AMH-2_5 GTTGAGAGTAACAGAGACTTTATCGCAGAAACGTTCAGCGTTGGCTTCAACATGACCTGC 4500

AMH-2_1 GTTGAGAGTAACGGAGACTTTATCGCAGAAGCGTTCAGCGTTGGCTTCAACATGACCTGC 4500

AMH-2_4 GTTGAGAGTAACAGAGACTTTATCGCAGAAACGATCAGCGTTGGCTTCAACATGACCTGC 4500

AMH-3_1 GTTGAGAGTAACAGAGACTTTATCGCAGAAACGTTCAGCGTTGGCTTCAACATGACCTGC 4500

AMH-3_13 GTTGAGAGTAACAGAGACTTTATCGCAGAAACGTTCAGCGTTGGCTTCAACATGACCTGC 4500

AMH-1_7 GTTGAGAGTAACAGAGACTTTATCGCAGAAACGTTCAGCGTTGGCTTCAACATGACCTGC 4500

AMH-1_11 GTTGAGAGTAACAGAGACTTTATCGCAGAAACGTTCAGCGTTGGCTTCAACATGACCTGC 4500

KMP-8_3 GTTGAGAGTAACAGAGACTTTATCGCAGAAACGTTCAGCGTTGGCTTCAACATGACCTGC 4497

AMH-1_2 GTTGAGAGTAACAGAGACTTTATCGCAGAAACGTTCAGCGTTGGCTTCAACATGACCTGC 4500

AY198374.1 GTTGAGAGTAACAGAGACTTTATCGCAGAAACGTTCAGCGTTGGCTTCAACATGACCTGC 4500

************.*****************.**:**************************

KMP-8_35 AATATAGATCAGGTGCTGCCGGGCACCAACGACGCCGGGGTGATTCAGGAGGCCATGGCG 4557

KMP-8_5 AATATAGATCAGGTGCTGCCGGGCACCAACGACGCCGGGGTGATTCAGGAGGCCATGGCG 4557

KMP-8_24 AATATAGATCAGGTGCTGCCGGGCACCAACGACGCCGGGGTGATTCAGGAGGCCATGGCG 4557

KMP-8_46 AATATAGATCAGGTGCTGCCGGGCACCAACGACGCCGGGGTGATTCAGGAGGCCATGGCG 4557

AMH-3_16 AATATAGATCAGGTGCTGCCGGGCACCAACGACGCCGGGGTGATTCAGGAGGCCATGGCG 4557

AMH-2_5 AATATAGATCAGGTGCTGCCGGGCACCAACGACGCCGGGGTGATTCAGGAGGCCATGGCG 4560

AMH-2_1 AATATAGATCAGGTGCTGCCGGGCACCAACGACGCCGGGGTGATTCAGGAGGCCATGGCG 4560

AMH-2_4 AATATAGATCAGGTGCTGCCGGGCACCAACGACGCCGGGGTGATTCAGGAGGCCATGGCG 4560

AMH-3_1 AATATAGATCAGGTGCTGCCGGGCACCAACGACGCCGGGGTGATTCAGGAGGCCATGGCG 4560

AMH-3_13 AATATAGATCAGGTGCTGCCGGGCACCAACGACGCCGGGGTGATTCAGGAGGCCATGGCG 4560

AMH-1_7 AATATAGATCAGGTGCTGCCGGGCACCAACGACGCCGGGGTGATTCAGGAGGCCATGGCG 4560

AMH-1_11 AATATAGATCAGGTGCTGCCGGGCACCAACGACGCCGGGGTGATTCAGGAGGCCATGGCG 4560

KMP-8_3 AATATAGATCAGGTGCTGCCGGGCACCAACGACGCCGGGGTGATTCAGGAGGCCATGGCG 4557

AMH-1_2 AATATAGATCAGGTGCTGCCGGGCACCAACGACGCCGGGGTGATTCAGGAGGCCATGGCG 4560

AY198374.1 AATATAGATCAGGTGCTGCCGGGCACCAACGACGCCGGGGTGATTCAGGAGGCCATGGCG 4560

************************************************************

KMP-8_35 GAAGTCCATGCTCACTTCATACAGGATAACATCCCTGTGAGCGCCGACAGTATTGAAGAG 4617

KMP-8_5 GAAGTCCATGCTCACTTCATACAGGATAACATCCCTGTGAGCGCCGACAGTATTGAAGAG 4617

KMP-8_24 GAAGTCCATGCTCACTTCATACAGGATAACATCCCTGTGAGCGCCGACAGTATTGAAGAG 4617

KMP-8_46 GAAGTCCATGCTCACTTCATACAGGATAACATCCCTGTGAGCGCCGACAGTATTGAAGAA 4617

AMH-3_16 GAAGTCCATGCTCACTTCATACAGGATAACATCCCTGTGAGCGCCGACAGTATTGAAGAG 4617

AMH-2_5 GAAGTCCATGCTCACTTCATACAGGATAACATCCCTGTGAGCGCCGACGGTATTGAAGAG 4620

AMH-2_1 GAAGTCCATGCTCACTTCATACAGGATAACATCCCTGTGAGCGCCGACAGTATTGAAGAG 4620

AMH-2_4 GAAGTCCATGCTCACTTCATACAGGATAACATCCCTGTGAGCGCCGACAGTATTGAAGAG 4620

AMH-3_1 GAAGTCCACGCTCACTTCATACAGGATAACATCCCTGTGAGCGCCGACAGTATTGAAGAG 4620

AMH-3_13 GAAGTCCACGCTCACTTCATACAGGATAACATCCCTGTGAGCGCCGACAGTACTGAAGAG 4620

AMH-1_7 GAAGTCCATGCTCACTTCATGCAGGATAACATCCCTGTGAGCGCCGACAGTATTGAAGAG 4620

AMH-1_11 GAAGTCCATGCTCACTTCATACAGGATAACATCCCTGTGAGCGCCGACAGTATTGAAGAG 4620

KMP-8_3 GAAGTCCATGCTCGCTTCATACAGGATAACATCCCTGTGAGCGCCGACAGTATTGAAGAG 4617

AMH-1_2 GAAGTCCATGCTCACTTCATACAGGATAACATCCCTGTGAGCGCCGACAGTATTGAAGAG 4620

AY198374.1 GAAGTCCATGCTCACTTCATACAGGATAACATCCCTGTGAGCGCCGACAGTATTGAAGAG 4620

******** ****.******.***************************.*** ******.

KMP-8_35 CTTCGCAGTGACACTCAGCTGCTGCGCTCCGTCCAAGGTGTGTTGAACCAACGGCTGTTG 4677

KMP-8_5 CTTCGCAGTGACACTCAGCTGCTGCGCTCCGTCCAAGGTGTGTTGAACCAACGGCTGTTG 4677

KMP-8_24 CTTCGCAGTGACACTCAGCTGCTGCGCTCCGTCCAAGGTGTGTTGAACCAACGGCTGTTG 4677

KMP-8_46 CTTCGCAGTGACACTCAGCTGCTGCGCTCCGTCCAAGGTGTGCTGAACCAACGGCTGTTG 4677

AMH-3_16 CTTCGCAGTGACACTCAGCTGCTGCGCTCCGTCCAAGGTGTGTTGAATCAACGGCTACTG 4677

AMH-2_5 CTTCGCAGTGACACTCAGCTGCTGCGCTCCGTCCAAGGTGCGTTGAACCAACGGCTGTTG 4680

AMH-2_1 CTTCGCAGTGACACTCAGCTGCTGCGCTCCGTCCAAGGTGTGTTGAACCAACGGCTGTTG 4680

AMH-2_4 CTTCGCAGTGACACTCAGCTGCTGCGCTCCGTCCAAGGTGTGTTGAATCAACGGCTGTTG 4680

AMH-3_1 CTTCGCGGTGACACTCAGCTGCTGCGCTCCGTCCAAGGTGTGTTGAACCAACGGCTGTTG 4680

AMH-3_13 CTTCGCAGTGACACTCAGCTGCTGCGCTCCGTCCAAGGTGTGTTGAACCAACGGCTGTTG 4680

AMH-1_7 CTTCGCAGTGACACTCAGCTGCTGCGCTCCGTCCAAGGTGTGTTGAACCAACGGCTGTTG 4680

AMH-1_11 CTTCGCAGTGACACTCAGCTGCTGCGCTCCGTCCAAGGTGTGTTGAACCAACGGCTGTTG 4680

KMP-8_3 CTTCGCAGTGACACTCAGCTGCTGCGCTCCGTCCAAGGTGTGTTGAACCAACGGCTGTTG 4677

AMH-1_2 CTTCGCAGTGACACTCAGCTGCTGCGCTCCGTCCAAGGTGTGTTGAACCGACGGCTGTTG 4680

AY198374.1 CTTCGCAGTGACACTCAGCTGCTGCGCTCCGTCCAAGGTGTGTTGAACCAACGGCTGTTG 4680

******.********************************* * **** *.******. **

KMP-8_35 GTCCTGAACGACCTGGTGACGGGGGTCAGCCCTGATCTCGGCACTGCCGGCGTGCAGATC 4737

KMP-8_5 GTCCTGAACGACCTGGTGACCGGGGTCAGCCCTGATCTCGGCACTGCCGGCGTGCAGATC 4737

KMP-8_24 GTCCTGAACGACCTGGTGACCGGGGTCAGCCCTGATCTCGGCACTGCCGGCGTGCAGATC 4737

KMP-8_46 GTCCTGAACGACCTGGTGACCGGGGTCAGCCCTGATCTCGGCACTGCCGGCGTGCAGATC 4737

AMH-3_16 GTCCTGAACGACCTGGTGACCGGGGTCAGCCCTGATCTCGGCACTGCCGGCGTGCAGATC 4737

AMH-2_5 GTCCTGAACGACCTGGTGACCGGGGTCAGCCCTGATCTCGGCACTGCCGGCGTGCAGATC 4740

AMH-2_1 GTCCTGAACGACCTGGTGACCGGGGTCAGCCCTGATCTCGGCACTGCCGGCGTGCAGATC 4740

AMH-2_4 GTCCTGAACGACCTGGTGACCGGGGTCAGCCCTGATCTCGGCACTGCCGGCGTGCAGATC 4740

AMH-3_1 GTCCTGAACGACCTGGTGACGGGGGTCAGCCCTGATCTCGGCACTGCCGGCGTGCAGATC 4740

AMH-3_13 GTCCTGAACGACCTGGTGACGGGGGTCAGCCCTGATCTCGGCACTGCCGGCGTGCAGATC 4740

AMH-1_7 GTCCTGGACGACCTGGTGACGGGGGTCAGCCCTGATCTCGGCACTGCCGGCGTGCAGATC 4740

AMH-1_11 GTCCTGAACGACCTGGTGACGGGGGTCAGCCCTGATCTCGGCACTGCCGGCGTGCAGATC 4740

KMP-8_3 GTCCTGGACGACCTGGTGACGGGGGTCAGCCCTGATCTCGGCACTGCCGGCGTGCAGATC 4737

AMH-1_2 GTCCTGAACGACCTGGTGACGGGGGTCAGCCCTGATCTCGGCACTGCCGGCGTGCAGATC 4740

AY198374.1 GTCCTGAACGACCTGGTGACGGGGGTCAGCCCTGATCTCGGCACTGCCGGCGTGCAGATC 4740

******.************* ***************************************

KMP-8_35 ACCATCTATGTGCTAGCCGGGTTGTCAGCTATCCTTGCCTTCCTGTGCCTTATTCTGCTC 4797

KMP-8_5 ACCATCTATGTGCTAGCCGGGTTGTCAGCCATCCTTGCCTTCCTGTGCCTTATTCTGCTC 4797

KMP-8_24 ACCATCTATGTGCTAGCCGGGTTGTCAGCCATCCTTGCCTTCCTGTGCCTTATTCTGCTC 4797

KMP-8_46 ACCATCTATGTGCTAGCCGGGTTGTCAGCCATCCTTGCCTTCCTGTGCCTTATTCTGCTC 4797

AMH-3_16 ACCATCTATGTGCTAGCCGGGTTGTCAGCCATCCTTGCCTTCCTGTGCCTTATTCTGCTC 4797

AMH-2_5 ACCATCTATGTGCTAGCCGGGTTGTCAGCCATCCTTGCCTTCCTGTGCCTTATTCTGCTC 4800

AMH-2_1 ACCATCTATGTGCTAGCCGGGTTGTCAGCCATCCTTGCCTTCCTGTGCCTTATTCTGCTC 4800

AMH-2_4 ACCATCTATGTGCTAGCCGGGTTGTCAGCCATCCTTGCCTTCCTGTGCCTTATTCTGCTC 4800

AMH-3_1 ACCATCTATGTGCTAGCCGGGTTGTCAGCCATCCTTGCCTTCCTGTGCCTTATTCTGCTC 4800

AMH-3_13 ACCATCTATGTGCTAGCCGGGTTGTCAGCCATCCTTGCCTTCCTGTGCCTTATTCTGCTC 4800

AMH-1_7 ACCATCTATGTGCTAGCCGGGTTGTCAGCCATCCTTGCCTTCCTGTGCCTTATTCTGCTC 4800

AMH-1_11 ACCATCTATGTGCTAGCCGGGTTGTCAGCCATCCTTGCCTTCCTGTGCCTTATTCTGCTC 4800

KMP-8_3 ACCATCTATGTGCTAGCCGGGTTGTCAGCCATCCTTGCCTTCCTGTGCCTTATTCTGCTC 4797

AMH-1_2 ACCATCTATGTGCTAGCCGGGTTGTCAGCCATCCTTGCCTTCCTGTGCCTTATTCTGCTC 4800

AY198374.1 ACCATCTATGTGCTAGCCGGGTTGTCAGCCATCCTTGCCTTCCTGTGCCTTATTCTGCTC 4800

***************************** ******************************

KMP-8_35 ATCACATTCATCGTGAGGACCCGAGCTCTGAACCGCCGTTTGGAAGCACTGTCGATGACG 4857

KMP-8_5 ATCACATTCATCGTGAGGACCCGAGCTCTGAACCGCCATTTGGAAGCACTGTCGATGACG 4857

KMP-8_24 ATCACATTCATCGTGAGGACCCGAGCTCTGAACCGCCGTTTGGAAGCACTGTCGATGACG 4857

KMP-8_46 ATCACATTCATCGTGAGGACCCGAGCTCTGAACCGCCGTTTGGAAGCACTGTCGATGACG 4857

AMH-3_16 ATCACATTCATCGTGAGGACCCGAGCTCTGAACCGCCGTTTGGAAGCACTGTCGATGACG 4857

AMH-2_5 ATCACATTCATCGTGAGGACCCGAGCTCTGAACCGCCGTTTGGAAGCACTGTCGATGACG 4860

AMH-2_1 ATCACATTCATCGTGAGGACCCGAGCTCTGAACCGCCGTTTGGAAGCACTGTCGATGACG 4860

AMH-2_4 ATCACATTCATCGTGAGGACCCGAGCTCTGAACCGCCGTTTGGAAGCACTGTCGATGACG 4860

AMH-3_1 ATCACATTCATCGTGAGGACCCGAGCTCTGAACCGCCGTTTGGAAGCACTGTCGATGACG 4860

AMH-3_13 ATCACATTCATCGTGAGGACCCGAGCTCTGAACCGCCGTTTGGAAGCACTGTCGATGACG 4860

AMH-1_7 ATCACATTCATCGTGAGGACCCGAGCTCTGAACCGCCGTTTGGAAGCACTGTCGATGACG 4860

AMH-1_11 ATCACATTCATCGTGAGGACCCGAGCTCTGAACCGCCGTTTGGAAGCACTGTCGATGACG 4860

KMP-8_3 ATCACATTCATCGTGAGGTCCCGAGCTCTGAACCGCCGTTTGGAAGCACTGTCGATGACG 4857

AMH-1_2 ATCACATTCATCGTGAGGACCCGAGCTCTGAACCGCCGTTTGGAAGCACTGTCGGTGACG 4860

AY198374.1 ATCACATTCATCGTGAGGACCCGAGCTCTGAACCGCCGTTTGGAAGCACTGTCGATGACG 4860

******************:******************.****************.*****

KMP-8_35 AAATACGGCTCGGTGGATTCGGGGCTGAACCGAGTGGGGATAGCGGCCCCAGGAACCAAC 4917

KMP-8_5 AAATACGGCTCGGTGGATTCGGGGCTGAACCGAGTGGGGATAGCGGCCCCAGGAACCAAC 4917

KMP-8_24 AAATACGGCTCGGTGGATTCGGGGCTGAACCGAGTGGGGATAGCGGCCCCAGGAACCAAC 4917

KMP-8_46 AAATACGGCTCGGTGGATTCGGGGCTGAACCGAGTGGGGATAGCGGCCCCAGGAACCAAC 4917

AMH-3_16 AAATACGGCTCGGTGGATTCGGGGCTGAACCGAGTGGGGATAGCGGCCCCAGGAACCAAC 4917

AMH-2_5 AAATACGGCTCGGTGGATTCGGGGCTGAACCGAGTGGGGATAGCGGCCCCAGGAACCAAC 4920

AMH-2_1 AAATACGGCTCGGTGGATTCGGGGCTGAACCGAGTGGGGATAGCGGCCCCAGGAACCAAC 4920

AMH-2_4 AAATACGGCTCGGTGGATTCGGGGCTGAACCGAGTGGGGATAGCGGCCCCAGGAACCAAC 4920

AMH-3_1 AAATACGGCTCGGTGGATTCAGGGCTGAACCGAGTGGGGATAGCGGCCCCAGGAACCAAC 4920

AMH-3_13 AAATACGGCTCGGTGGATTCAGGGCTGAGCCGAGTGGGGATAGCGGCCCCAGGAACCAAC 4920

AMH-1_7 AAATACGGCTCGGTGGATTCGGGGCTGAACCGAGTGGGGATAGCGGCCCCAGGAACCAAC 4920

AMH-1_11 AAATACGGCTCGGTGGATTCGGGGCTGAACCGAGTGGGGATAGCGGCCCCAGGAACCAAC 4920

KMP-8_3 AAATACGGCTCGGTGGATTCGGGGCTGAACCGAGTGGGGATAGCGGCCCCAGGAACCAAC 4917

AMH-1_2 AAGTACGGCTCGGTGGATTCGGGGCTGAACCGAGTGGGGATAGCGGCCCCAGGAACCAAC 4920

AY198374.1 AAATACGGCTCGGTGGATTCGGGGCTGAACCGAGTGGGGATAGCGGCCCCAGGAACCAAC 4920

**.*****************.*******.*******************************

KMP-8_35 AAACACGCCATCGAAGGCTCCAACCCCATCTGGAACGAGCAGATCAAGGCCCCGGACTTC 4977

KMP-8_5 AAACACGCCATCGAAGGCTCCAACCCCATCTGGAACGAGCAGATCAAGGCCCCGGACTTC 4977

KMP-8_24 AAACACGCCATCGAAGGCTCCAACCCCATCTGGAACGAGCAGATCAAGGCCCCGGACTTC 4977

KMP-8_46 AAACACGCCATCGAAGGCTCCAACCCCATCTGGAACGAGCAGATCAAGGCCCCGGACTTC 4977

AMH-3_16 AAACACGCCATCGAAGGCTCCAACCCCATCTGGAACGAGCAGATCAAGGCCCCGGACTTC 4977

AMH-2_5 AAACACGCCATCGAAGGCTCCAACCCCATCTGGAACGAGCAGATCAAGGCCCCGGACTTC 4980

AMH-2_1 AAACACGCCATCGAAGGCTCCAACCCCATCTGGAACGAGCAGATCAAGGCCCCGGACTTC 4980

AMH-2_4 AAACACGCCATCGAAGGCTCCAACCCCATCTGGAACGAGCAGATCAAGGCCCCGGACTTC 4980

AMH-3_1 AAACACGCCATCGAAGGCTCCAACCCCATCTGGAACGAGCAGATCAAGGCCCCGGACTTC 4980

AMH-3_13 AAACACGCCATCGAAGGCTCCAACCCCATCTGGAACGAGCAGATCAAGGCCCCGGACTTC 4980

AMH-1_7 AAACACGCCATCGAAGGCTCCCACCCCATCTGGAACGAGCAGATCAAGGCCCCGGACTTC 4980

AMH-1_11 AAACACGCCATCGAAGGCTCCAACCCCATCTGGAACGAGCAGATCAAGGCCCCGGACTTC 4980

KMP-8_3 AAACACGCCATCGAAGGCTCCAACCCCATCTGGAACGAGCAGATCAAGGCCCCGGACTTC 4977

AMH-1_2 AAACACGCCATCGAAGGCTCCAACCCCATCTGGAACGAGCAGATCAAGGCCCCGGACTTC 4980

AY198374.1 AAACACGCCATCGAAGGCTCCAACCCCATCTGGAACGAGCAGATCAAGGCCCCGGACTTC 4980

*********************.**************************************

KMP-8_35 GATGCCATCAGTGACACATCTGACGAGTCTGATCTGATCGGCATCGAGGATCTACCACAA 5037

KMP-8_5 GATGCCATCAGTGACACATCTGACGAGTCTGATCTGATCGGCATCGAGGATCTACCACAA 5037

KMP-8_24 GATGCCATCAGTGACACATCTGACGAGTCTGATCTGATCGGCATCGAGGATCTACCACAA 5037

KMP-8_46 GATGCCATCAGTGACACATCTGGCGAGTCTGATCTGATCGGCATCGAGGATCTACCACAA 5037

AMH-3_16 GATGCCATCAGTGACACATCTGACGAGTCTGATCTGATCGGCATCGAGGATCTACCACAA 5037

AMH-2_5 GATGCCATCAGTGACACATCTGACGAGTCTGATCTGATCGGCATCGAGGATCTACCACAA 5040

AMH-2_1 GATGCCATCAGTGACACATCTGACGAGTCTGATCTGATCGGCATCGAGGATCTACCACAA 5040

AMH-2_4 GATGCCATCAGTGACACATCTGACGAGTCTGATCTGATCGGCATCGAGGATCTACCACAA 5040

AMH-3_1 GATGCCATCAGTGACACATCTGACGAGTCTGATCTGATCGGCATCGAGGATCTACCACAA 5040

AMH-3_13 GATGCCATCAGTGACACATCTGACGAGTCTGATCTGATCGGCATCGAGGATCTACCACAA 5040

AMH-1_7 GATGCCATCAGTGACACATCTGACGAGTCTGATCTGATCGGCATCGAGGATCTACCACAA 5040

AMH-1_11 GATGCCATCAGTGACACATCTGACGAGTCTGATCTGATCGGCATCGAGGATCTACCACAA 5040

KMP-8_3 GATGCCATCAGTGACACATCTGACGAGTCTGATCTGATCGGCATCGAGGATCTACCACAA 5037

AMH-1_2 GATGCCATCAGTGACACATCTGACGAGTCTGATCTGATCGGCATCGAGGATCTACCACAA 5040

AY198374.1 GATGCCATCAGTGACACATCTGACGAGTCTGATCTGATCGGCATCGAGGATCTACCACAA 5040

**********************.*************************************

KMP-8_35 TTCAAGAGCGACTATTTCCCGCCTGAGGACTCGGAATCCGCTCACGCCGCCTTTAGCGAC 5097

KMP-8_5 TTCAAGAGCGACTATTTCCCGCCTGAGGACTCGGAATCCGCTCACGCCGCCTTTAGCGAC 5097

KMP-8_24 TTCAAGAGCGACTATTTCCCGCCTGAGGACTCGGAATCCGCTCACGCCGCCTTTAGCGAC 5097

KMP-8_46 TTCAAGAGCGACTATTTCCCGCCTGAGGACTCGGAATCCGCTCACGCCGCCTTTAGCGAC 5097

AMH-3_16 TTCAGGAGCGACTATTTCCCGCCTGAGGACTCGGAATCCGCTCACGCCTCCTTTAGCGAC 5097

AMH-2_5 TTCAAGAGCGACTATTTCCCGCCTGAGGACTCGGAATCCGCTCACGCCGCCTTTAGCGAC 5100

AMH-2_1 TTCAAGAGCGACTATTTCCCGCCTGAGGACTCGGAATCCGCTCACGCCGCCTTTAGCGAC 5100

AMH-2_4 TTCAAGAGCGACTATTTCCCGCCTGAGGACTCGGAATCCGCTCACGCCGCCTTTAGCGAC 5100

AMH-3_1 TTCAAGAGCGACTATTTCCCGCCTGAGGACTCGGAATCCGCTCACGCCGCCTTTAGCGAC 5100

AMH-3_13 TTCAAGAGCGACTATTTCCCGCCTGAGGACTCGGAATCCGCTCACGCCGCCTTTAGCGAC 5100

AMH-1_7 TTCAAGAGCGACTATTTCCCGCCTGAGGACTCGGAATCCGCTCACGCCGCCTTTAGCGAC 5100

AMH-1_11 TTCAAGAGCGACTATTTCCCGCCTGAGGACTCGGAATCCGCTCACGCCGCCTTTAGCGAC 5100

KMP-8_3 TTCAAGAGCGACTATTTCCCGCCTGAGGACTCGGAATCCGCTCACGCCGCCTTTAGCGAC 5097

AMH-1_2 TTCAAGAGCGGCTATTTCCCGCCTGAGGACTCGGAATCCGCTCACGCCGCCTTTAGCGAC 5100

AY198374.1 TTCAAGAGCGACTATTTCCCGCCTGAGGACTCGGAATCCGCTCACGCCGCCTTTAGCGAC 5100

****.*****.************************************* ***********

KMP-8_35 CGCACGCCACGCGGGAACGATGCGCCTATTGCACACAGTAGCAACAACTTCGGTTTCAAC 5157

KMP-8_5 CGCACGCCACGCGGGAACGATGCGCCTATTGCACACAGTAGCAACAACTTCGGTTTCAAC 5157

KMP-8_24 CGCACGCCACGCGGGAACGATGCGCCTATTGCACACAGTAGCAACAACTTCGGTTTCAAC 5157

KMP-8_46 CGCACGCCACGCGGGAACGATGCGCCTATTGCACACAGTAGCAACAACTTCGGTTTCAAC 5157

AMH-3_16 CGCACGCCACGCGGGAACGATGCGCCTATTGCACACAGTAGCAACAACTTCGGTTTCAAC 5157

AMH-2_5 CGCACGCCACGCGGGAACGATGCGCCTATTGCACACAGTAGCAACAACTTCGGTTTCAAC 5160

AMH-2_1 CGCACGCCACGCGGGAACGATGCGCCTATTGCACACAGTAGCAACAACTTCGGTTTCAAC 5160

AMH-2_4 CGCACGCCACGCGGGAACGATGCGCCTATTGCACACAGTAGCAACAACTTCGGTTTCAAC 5160

AMH-3_1 CGCACGCCACGCGGGAACGATGCGCCTATTGCACACAGTAGCAACAACTTCGGTTTCAAC 5160

AMH-3_13 CGCACGCCACGCGGGAACGATGCGCCTATTGCACACAGTAGCAACAACTTCGGTTTCAAC 5160

AMH-1_7 CGCACGCCACGCGGGAACGATGCGCCTATTGCACACAGTAGCAACAACTTCGGTTTCAAC 5160

AMH-1_11 CGCACGCCACGCGGGAACGATGCGCCTATTGCACACAGTAGCAACAACTTCGGTTTCAAC 5160

KMP-8_3 CGCACGCCACGCGGGAACGATGCGCCTATTGCACACAGTAGCAACAACTTCGGTTTCAAC 5157

AMH-1_2 CGCACGCCACGCGGGAACGATGCGCCTATTGCACACAGTAGCAACAACTTCGGTTTCAAC 5160

AY198374.1 CGCACGCCACGCGGGAACGATGCGCCTATTGCACACAGTAGCAACAACTTCGGTTTCAAC 5160

************************************************************

KMP-8_35 ACCAGTCCTTTTAGCGCGGAGTTCACTAACAGGCGCATGCGACCATAG 5205

KMP-8_5 ACCAGCCCTTTTAGCGCGGAGTTCACTAACAGGCGCATGCGACCATAG 5205

KMP-8_24 ACCAGCCCTTTTAGCGCGGAGTTCACTAACAGGCGCATGCGACCATAG 5205

KMP-8_46 ACCAGCCCTTTTAGCGCGGAGTTCACTAACAGGCGCATGCGACCATAG 5205

AMH-3_16 ACCAGCCCTTTTAGCGCGGAGTTCACTAACAGGCGCATGCGACCATAG 5205

AMH-2_5 ACCAGCCCTTTTAGCGCGGGGTTCACTAACAGGCGCATGCGACCATAG 5208

AMH-2_1 ACCAGCCCTTTTAGCGCGGAGTTCACTAACAGGCGCATGCGACCATAG 5208

AMH-2_4 ACCAGCCCTTTTAGCGCGGAGTTCACTAACAGGCGCATGCGACCATAG 5208

AMH-3_1 ACCAGCCCTTTTAGCGCGGAGTTCACTAACAGGCGCATGCGACCATAG 5208

AMH-3_13 ACCAGCCCTTTTAGCGCGGAGTTCACTAACAGGCGCATGCGACCATAG 5208

AMH-1_7 ACCAGTCCTTTTAGCGCGGAGTTCACTAACAGGCGCATGCGACCATAG 5208

AMH-1_11 ACCAGTCCTTTTAGCGCGGAGTTCACTAACAGGCGCATGCGACCATAG 5208

KMP-8_3 ACCAGTCCTTTTAGCGCGGAGTTCACTAACAGGCGCATGCGACCATAG 5205

AMH-1_2 ACCAGCCCTTTTAGCGCGGAGTTCACTAACAGGCGCATGCGACCATAG 5208

AY198374.1 ACCAGTCCTTTTAGCGCGGAGTTCACTAACAGGCGCATGCGACCATAG 5208

***** *************.****************************
