# Supplementary material for: Alternative Splicing and Highly Variable Cadherin Transcripts Associated with Field-Evolved Resistance of Pink Bollworm to Bt Cotton in India
Source: PLoS One. 2014 May 19;9(5):e97900. doi: 10.1371/journal.pone.0097900 (PMC4026531; doi:10.1371/journal.pone.0097900)
Supplement: Figure S8 — Partial genomic DNA sequencing of seven novel disrupted cadherin alleles in pink bollworm larvae from Anand (AGJ) in Gujarat and Khandwa (KMP) in Madhya Pradesh. Four mutations (found in isoforms r5A, r8B, r11B, and r12A) have altered gDNA, whereas 16 mutations are due to post-transcription modifications. Green-highlighted sequences show location of sense and antisense primers (from Table S3). Exon coding regions are shown as normal text and introns are highlighted in gray. Exon/intron splice junction nucleotides are highlighted in light blue. Yellow-highlighted sequence indicates insertions. Pink-highlighted sequence indicates gaps in sequencing. The 20 gDNA fragments shown are r5A_20-81, r5B_227-228, r5C_89-10, r7A_20-165, r7B_164-163, r8A_186-166, r8B_219-220, r9A_171-25, r9B_58-87, r10A-r10C_20-21, r10A-r10C_169-170, r10B_86-167, r10C_24-85, r11A_20-49, r11B_171-172, r12A_221-222, r12B_168-187, r12C-r12D_227-228, r12C_89-10, and r12D_186-73 (GenBank accession KJ724990-KJ725008). Note that r12A_221-222 does not have an accession number because it does not meet the minimum number of bases required by GenBank. (DOCX) [file pone.0097900.s008.docx]

**Figure S8.** **Partial genomic DNA sequencing of seven novel disrupted cadherin alleles in pink bollworm larvae from Anand (AGJ) in Gujarat and Khandwa (KMP) in Madhya Pradesh.** Four mutations (found in isoforms *r5A*, *r8B*, *r11B*, and *r12A*) have altered gDNA, whereas 16 mutations are due to post-transcription modifications. Green-highlighted sequences show location of sense and antisense primers (from Table S3). Exon coding regions are shown as normal text and introns are highlighted in gray. Exon/intron splice junction nucleotides are highlighted in light blue. Yellow-highlighted sequence indicates insertions. Pink-highlighted sequence indicates gaps in sequencing. The 20 gDNA fragments shown are r5A_20-81, r5B_227-228, r5C_89-10, r7A_20-165, r7B_164-163, r8A_186-166, r8B_219-220, r9A_171-25, r9B_58-87, r10A-r10C_20-21, r10A-r10C_169-170, r10B_86-167, r10C_24-85, r11A_20-49, r11B_171-172, r12A_221-222, r12B_168-187, r12C-r12D_227-228, r12C_89-10, and r12D_186-73 (GenBank accession KJ724990-KJ725008). Note that r12A_221-222 does not have an accession number because it does not meet the minimum number of bases required by GenBank.

**>Pectinophora gossypiella isolate AGJ-1 clone r5A_20-81 cadherin (Cad1) gene, partial sequence (KJ724990)**

ATGCTGTCGCCTAACAACGTAACCGGATTCCTCCAGACAGCAATGCCTTTGAGAGGATATTGGGGGACTTACGATATAAGTGTACTGGTAAGTCGAATGAGTCGAATGGTAACGACCTCCGTGGTCCAGTGGTTGAGCGTTGGGCTCACGATCCGAAGGTCTTTGGCGGGTCAATAGTAACCCTGACACCAGAGTTGATGAGGTTGGTAATCCACCTCACAACCCACACGATAGAAGAAGTGATCATAGCTTCTTTTGACGTGATCTATCCCTAGCATTCACACATGGCAATGGAAATCACTGAAGGCCGATCGCTTATACCAATGCCCGTATTTCTTATTGTACCTATTAAATTTGTCTGTGAAAGACACACATACAATACAAGGAACAAAAATAAAATTGCTATCCATAATTCTAGGCTAAGTAAATTAAATTCATCTTCTTTGGGGTTATGTACTTACACGTTTTTACAATAAAAAAACAGACGGTATTTTGAATAAGTACGTCAGAACATAAAAAAGCTAATTACGTGAGCGACAGACGGAGGGTGAAGAAGAGAAACTAGAATCGCTTAGAATGAAATTATATTTTAAATTTACAGCTAAACCTAACTAACCTAGGTGCGAATGAGTCCGAAGTTAGGACTACACTGAATTTTACTTAAAATCTATTTTACATTATGGCTAAATCGAGGTCACAATTTGGTGGCGTGATTGACGTCAGCGCATCGCGTTCGGGCGCTGGCTCAGCAGGCTGGCTGTTCCATTAATTATGCTGACTTGAACTCGTGGAACGACGCTGGTCACGGCGTACGGGTGTGAGCAGGCGGGCGAAGGTACCGCTTACGTAACATCTCCACCCTCAGAACAGCACCTATTCGAAGACTATCGTCGAGAATAGTGCTGGTCACCATTTCTTGGCTTATCTTCTTTCAAGGTGTAATAACCTGGCCTTCCTGCACGGAGAATCGATGTTCCTGGTTGAATCGATGGTTTGATATCATGTTAGGTTCTTGGATTCTTCCCGGTCAGATTACGGCTGTATCGTAGTAATTGCTTGCAAGTAACTTAGATAACATTTCATATATTTTCGTCGTTACTTGTAATTTGAAGTAGCTTCATCTCGAAGTTCCCACAATGTTTGGTTTTGGTGCATTTCTTTAGTGGCTTGTGTGGTCCTCGCTGCGCTTCATTTCCAATGCCCTAGGCGGGAAAGGAATTTACCGTTCTGATGTTGGAAATCCATGGCCTATTACGACTCGGAGCGGTGGATAGGTGGTCTATTGCCCGAGCATGGGTGCCTGGTGGACTTCGGGGATGGCGAGGTGCGCATATACATTAGTCTTCAGGGCGTACTGCATAACCATCGGTGTGTCCCGCTTCTGGGATTCCTGGAAAACCTGGATGCACTGGAGGACTGGGACGAACCATAAGGTACTACACCAGCGGAGTAATTATTCTTCTTTTTTTTCTGACCTCGTTTTTCTTCACTTATATAATTTTCTTCTTTTTCTTTTTCTTACATCTTTTCTTGGCTTAATTTTAATCTTAATTATTTACACTCTGTTCCCTTCTTCATTATCGATGCTTGGATAACCTCCGTTGTGACTTCACGTAGATTCTGAATACTCGAGATGGTGCTTGATAGTTACCCGGGCAGCCCGGAGCACGGTGTTCGATGTGAGATACGCCTGAATTTGCTCGCGAACTGACCCGCGAGTATCCTACCGACTGCGCCAATTATGATCGCGGTAGCCGGAGTACTCAAAAAAAACCAAAAATTTCTAACACTAATTTAATTACGAAAAATAACAAGTTACAAAAATTACTTATATTAACTTAATTTTACGGGCGAGTTGACCTGATCGATACAAGAATCAAAGTAACTCGCAGTGGCGCGATCACGTTACGTTGGAGTCTCATCGGCGTCAGCAGTTTACCCCACTCGCAGGAATGAGGCCGTGACACGTGTGGGTATTGCAGCGTCTGCAGTTGGGGCCGTGTGCAATATTTCCCTGCAGATGGAGTATTCAGCACATTCCCTTCTGTGTGATGACGTAACAAGCTCATGTGAAACTTACTTTATGTAAAAAGCTTATAATTTACTAATAAGATCAATGATTACCCAAATGATAAGTGTCTGGTATTCAATGTGTTTAAGTTTAAAAGATAGGTGGTAACATTACTTTGCACAAACGCACACACAAACATCTCTCACCCTAAACGCATATACCTGCTCCGTTCCGTCGTGGGTAATAATAAATGTAATTAAAATTATACTAACCGTAATTAACAACACTTTGCTTTACTTAATACATAACATACATAAACTGCCTATATACGTCCCACTGCTGGGCACAGGCCTCCCCTCAATCAACCGGAGGGGGTATGGAGCATACTCCACCACGCTGCTCCACTGCGGGTTGGTGGAGGTGTTTTTACGGCTAATAGCCGAGACCAACGGCTTAACGTGCCCTCCGAAGCACGGAATCATCTTACTTTTTCGGACAATCAGGTGATTCAAGCCTGAAAAGTCCTTACCAAACAAAGGACAGTCTCAGAAAGTGATTTCGACAATGTCCCCATTGGGAATCGAACCCGGACCACCAGATCGTGAGCCTAACGCTCTAACCACTAGACCACGGAGGCTGTTTACTTAATACTACCTGATTATTGAGTGATGGGTTCAACTTTATGAATTAAATGAGGATTAATTGGATGAATTTGATTCACTTGACCACACAATCCACGTGCCACTTATAGTCGATCTTGATTTTTCTTTAATCTTCGCCAATCACTTCGTATTAATTTATCTACAATTTATTGATTGTGATCTTCTCTGAAATCTAGCTCGAAGGACCATGAATATGCCTGATATGCCTTTTTACTGATTTTTAAGGGATTTAGGTAGCAATGAAACAGCACCGTGTTCGACGTAATTACGTGTAATCAATGGAACCGAAAAAAAAACGCCTGTCAAAAACAATTAACTAATAAAAACTATTGCAAATAAATTGCTATTATAAATTGAAGAGGCGTTTCGAATAATATTTATTCTAAACACTAATATTGTGGCGCCCCCTAGTGAGTTACAGACATGACACCTGTCTAATAACATGAACTCATCAAACACAAACCCGTTATTGAAAACCGCATCAAAATCGGATTATTAGTTTAGAAGATAATTAATAACATTTCTTTGCACAAACGCACACACAAACATCTCTCACCCTAAACGCATATACCTGCTCCGTTCCGTCGTGGGTAAAAATAACCATTATCTCTGTAATGAAAGCCTTATTCGTAAATCGGTAAAGTACGTATCAAATTGGCATTGTTGTTTCAACGCCTGAAAGTATTTTCATACGTCTAGGCCTACGATTTCTTACTAATAATGCGTTTTTTCTTTTGTGGACGGACTTGCCTCACACTGCATTGGAAGACCGTCTACTTGACATGTTTTAAAAAAAACGATTGTATATCAAAAGATTACGGCGCTTTCTCTTATATTTAGACTTATAAACATACCCAAGTAAATTATCTACCATACCCGATCATATGACTTGGTCTACAAATAATATCTTCTTCAATAAACTCAGTCAAACTCAAATTTTAGAACAGTATGCCGGTCTTCCCCACATTGACCTAAATAAGTTTAATAGAAAAAAGAAAACGTTTTTCGACAGTTTTAGATCTACCTTTAATCAGAATTTCATAATTTATCTTTGACCTAGGAAACTACCCAATTAGGCAATTACTTGTATTTCTTGTTACTATCAATTCTTTTTCCAGAAAAAGAGGTTGGCTTGACTGAGAGGTTCTCGCTCC

**>Pectinophora gossypiella isolate AGJ-1 clone r5B_227-228 cadherin (Cad1) gene, partial sequence (KJ724991)**

TACTGGTGACGGTGCTTCTGACCTTCGCAACATCAGTTTTCGGGCAAGAAAGTAATCATCATCATTAATTTAAGAACCACGCTCTTGTCGGTGTAGTATTCTCCATTATTGTCTATTAACGTTGAAAGAAAGAAAAGGAATAATCTATCCAGAATAATCTAATTAATGTCGTAAAACCGAATGGTCTTTTAAAATCCGAACCCCATGGTCTGGAAATCTGAGTCTCAGTGCGCTTTAGCCTGTAATAAACACTTAAGTACTTACCTGTGAGATGCAGGGGTTACTGTTATGTACATTTTTTTCACAGCTTAAAGAGAACGCTGACATCAGTAAGTACTTACCTGTTAAAATGTGATATATCTACCTTCAGACGTTTCCGATAATCCTTTTGAGGTTCGGCCTAAAAAGGTGGCTGTGGGCAGTGGCCCGTTTACGCAGAGCCAAATCTTATGATGGATAGGACCTATTAACCATTATCGACTAAGCACCATCGTAACTACCTATCAGAATTTTAGTGACATCGTAACAAATACGTGATTCTGAGTTGATATCAAGTGTGACATGTTTTGTCTTCTTAAATTTCATTTCTATACTTTTGCGATGGAAAATTCCACTTGATATTACTCAGAATATTGAGCTGAATCATCCCTCAAAGTTTTCGTTACGATGTCACTAACACCCTGTCATATATCCTTACCTAATTTTATAAAATTGACAGCAGCATCGTCGAGATGTTACTACATGACTGACGCTATTCCGAGAGAACCGAAACCGGATGATT

**>Pectinophora gossypiella isolate AGJ-1 clone r5C_89-10 cadherin (Cad1) gene, partial sequence (KJ724992)**

GGAACCCCAGAAATTGTCCAGCCGATGGTTATAGGATCTTTTAACCTGCTAAGTCCAGAGATCCGGAATGAAAACGGGGCGTGGTACCTTTATATAACCAATAGGTAAAGACTTCGTATCATATCTAACCTATAGGATCCTTTCAATGCAATGGTCACCTCTTCATCTTTCCATCTTCCCCTGCTTAAGGTGGATGCCGAAGTATGGATCATCTTACTTTCGGACATTTGGTCCAATGTGGAATTGGTGGACAATTTGCAACCTTTTAGACGATAGACTTTTGAAGCCAACAAAAACATGCAACAAACCACCACCACCGAAATAACGTATATGTACAGAAGGCACTTTGAAACTTCAATCAGCAACAATGACTTTAAATCTAATGAACACATGCGGTGTCCAAATTCCAATGTCATACGTACCACATTAAGAATGAAAGTTCAATGTGTCTGTGAAAATGTTAATTCTAGTTCTGAACTTGAAATCGCAGGCAAGATTATGAAACACCAACAATGCGTCGGTATACATTCGACGTCCGAGTGCCAGACGAGACTCGTGCCGCACGAGTGAGTCTCTCCATCGAAAACATTGACGATAACGACCCTATCGTCAGGGTGCTAGACGCTTGCCAAGTGCCGGTGAGTCAACAACCTATTCAATTTGACTAGAAAGAACAAAAATCCGTAAACACATTATCCGTGTAAACATACACCCGTAATTCGTAAAGGCAATTGGGCAGAGCTACAAGTAATCAAAGACAACTTGCAGCCACTCTTAATACGAAGTTAATAAGACAAAATAATATCATCTGTCGCAAATAAAACAAAAACATAACAGAACATTTAAGCAGTCTGAGGTACATAAAACAACAATAGAACTAAAAATAGAGAAGGTCCTAATATTGATCCCTGCGGAACACCAATTTCCACAATAATTGTCCTTTTTACCAGATTAGTACTCTCATCGAAAAATATTGTCACTTATATTTTACTTTTGACCAGTGATAGAAATGAATTAAGTAAGTACTACTTCTTTTGTATTTTATGCAAGAATTCATAGAGAGGTTGTTGAGAACGTTGAAGCCTTTGATATATATAATATTATATTATGTTACTAGTTGAAGCAGTGTTCTGTAGCTGTTCAATTTGTTATTTCCCAGGAATTGGGGGAGCCTCGACTAACTGACTGCGTTTACCAAGTGTCAGACGAAGATGGGAGGCTTAGTATCGAGCCCATGACATTCCGCCTCACATCAGACCGTGAAGACGTACAGATATTCTATGTGGAGCCAGCTCACATTACTGGTGATTGGTTCAACATGCAAATTACTATCGGTATCCTATCAGCGCTTAACTTCGAAAGCAACCCGCTGCACATCTT

**>Pectinophora gossypiella isolate AGJ-3 clone r7A_20-165 cadherin (Cad1) gene, partial sequence (KJ724993)**

ATGCTGTCGCCTAACAACGTAACCGGATTCCTTCAGACAGCAATGCCTTTGAGAGGATATTGGGGTACTTACGATATAAGTATACTGGTAAGTCGAATGAGTCGAATGGTAACGATCTCCGTGGTCCAGTGGTTGAGCGTTGGGCTCACGATCCGAAGGTCTTTGGCGGGTCAATAGTAACCCTGACACCAGAGTTGATGAGGTTGGTAATCCACCTCACAACCCACTATCGTGTGGGTTGTGAGGTGGAAGAAGTGATCATAGCTTCTTTTGACGTGATCTATCCCTAGCATTCACACATGGCAATGGAAAGCACTGAAGGCCGATCGCTTATACGTACCAATGCCCGTTTTTCTTATTGTACCTATTAAATTTGTCTGTGAAAGACACACATACAATACAAGGAACAAAAATAAAATTGCTATCCATAATTCTAGGTTAAGTAAATTAAATTCATCTTCTTTGGGGTTATGTACTTACACGTTTTTACAATAAAAAAACAGACAGTAAGTATTTTGAATAAGTACCTACGTCAGAACATAAATTTATAGCTCATGTGAAACTTACTTTATGTAAAATTCTTATAATTAACTAATAAGATCAATGATTACCCAAATGATAAAAGTGCCTGGTATTCAATGTGTTTCTCTAGTTATTAAATAACTTGTACTTATGTATAAGATGTGTTGCTGTTGCAGTTTCTGGCCATTTCTTCTCCAGCTAGCCACCTAGCGGGATGACGTAGTTTAAAAAAATATTAAATTGACCTTCAACATGTAATGTACCTGTCTGTCTGTGTCTGTGGTGTCTGGAAGTAATAAATGTTTCTTTCTTTCAACAAGTTTATCTATGATAATTACGTTGAACAAACGATTCTGATTCTGTCTTTGTCCATGACAGGCGTTCGACCACGGTATTCCTCAGCAGATATCTCATGAGGTGTATGAATTGGAAATTCGACCTTACAATTACAATCCTCCCCAGTTCGTTTTTCCTGAATCCGGGACGATTCTACGACTGGCTTTGGTAAGATTTCTATTATTGTAACTCAAAAATTGCGATAATAATTTCAAAATGGCGTTTTAAGAAAGAGTAAGCAGATGGCGATAGTGTATGGCCGCCGTTAGAATCCGGAAGTCTAAAATAGTCACATAGAATTCATCTAAAAAGCTATAATTTGATGCCATTTGCGCATAAAATAAAATAAAATGTCCACATACTTAGAAGAAATTCATCTAAAAAAAGCTATAATATGATGACATTTGTGCATAAAATAAAATAAAGTGCGCAGTCTGGGGGTTCCAAATGGCCACATAGAAGCAATTCATGTAAAATGCTATAATTTGATGACATTTGCGCATAAAATAAAGTGCGCAGTATCAACAAGTATCAAATAACAATATAGCTTTTATCGCTTTATTTGACAGCTGTCAGACTAATAACACCTAAACGCAAGCCAGCATTATCACATAATTAAACCCTATAACTATGAGATTCAAAAACATCAAATCAAGTTGCTTTTAGGGATAGTAGTAGATACGTATAGTCAGAAGTAAATAGTGACAGTCAAAGTATACATAATAGTTAGCAACATCCACAATGACCAATAATTAGGGATAATCAAGGTGGCCATATAGATAGTAACACTCTAAGTGACCAAATACATCGGCACAACTTTTTGAACACGCAGGTTGTGCCGATGTTTTTCGTTACTTAGAGTGTTACTATCTATACGGCCGCCTTGAAAGTCCCTAATTATTGGACATTCTGGATCTATATTCGCATGCCAACTCTTCTTTCTTATGGCTCTGTGGTTTTGATTCAAATCTCTGCCGAATTATTAACATTCTTGAAATACGTTACAGGAACGCGCAGTGgtaaataatgttttgtcacttgtaaacggtgacccgttagacaggatacaagcaattgacgacgatggtcttgatgctggcgtggtgactttcgatattgttggagatggtgagtaatttgccaaatcttctttctttcttatcgtgtgggttgtgaggtggaataccagcctcatcaaccctggtgtcagggttattattgagctaccaaagacccctgacatggctcatgtaacgattactcactaacatcagtaagcagaaaccgggaccaacgtcttaacgtgcctgccgaagcacggatctaagaagttgattcagtgtttcggagggcacgttaagccgttgatcccggctattggccgtaaaaatacctccaccgacccgcagtggagcaacgtggtggagtatgctctataccccctccggttgattgaggagaggcctgtgctataggctatttaagttagttttagatctgtctttatttagtaataagtaatcagaatttcataatttatcttgaacctagtacacgatacaattcatacttagctatttcctgcttacagctgatgcatcaaactacttcagagtaaataatgatggcgacaactttggaaccttgttgctgacac

**>Pectinophora gossypiella isolate AGJ-3 clone r7B_164-163 cadherin (Cad1) gene, partial sequence (KJ724994)**

GCAAGTGGCAGCCAGTAATTCGCCCACAGGAGGCGGAATACCACTCCCTGGGTCTCTTCTCACCGTCACTGTCACTGTAAGTTAAATCAAGGCAAAAATGTCTTTACTCATCTCAATTGGGGCAGTCTGACGTACATCCATCGCAAAATGAACTAAGTTCCCACACTTCATCGAGTTTTCTGTTAGAAATGAAACGATAAGAAGGTTCCAGTTACGCATACTTCTTCTTCCCACGTAAGGGTCGTGAGACAGCGATAGACCTCATCATCAACCCTGATGTTAGGGTTCTATTGGGCCGCCAAAGGCTAGTATTAAAGGTCTACTAGGGCAAGTCTCGAAATAGTAACCGGGACCGACTGCTTAACGTGCCCTCCGGAGCACGGATCATCTGACTTTCGGACAATTCCCTATATTCCCTAGGGTGGTATTAATAAACTAATATCAGCTAAGACTGCCCTCAAGACCATGCTCAAGTCCCTGTTTTTATATAAGAACTGTCACATTGACATGATTTTGAGAACAGTCTTAAAGCTAAGATTAGTTTATTAATACCACCCTAGGGAACATGAAATAATACATAAAATATGTGGCATGGTACCCTCGTACTATGCTAACTTCGGTATCCCGTTAGCCATAAGTTGTGACTAGGGATCAATGGTCGAACGGAAGTTGTGCATCGATCTGAGCAAATTTCGTCAGCGCTTTCTAGGACTGCATTGGATAATGTAGGCGCCATCTGTTGGGTATAGGCGGAACTAGATGGTGAACTAGTTGGGTCCGTCACTTGCACTTATTTACAAGGGTACTTAATCTTAACAACTCCCGTATAGGTACGAGAAGCGGATCCACGGCCTGTGTTCGAGCAGCGTCTGTACACGGCTGGCATTTCCACTTCCGATAACATCAACAGGGAACTACTCACCGTTCGTGTAAGTTCTCCTTCTCTCTCTCTATTTAAGAGCTGCGCTCTTGTCGGTGGAGTAATCGCCATTCCTCTCTTCTTCCCGCCAAAACCTTCACCTCCCGATACGACACGACCTGCACCTTCTCTTTTATTTGTTTCATAAATGTTATCCTAGGTCTAGGTCCCTTCCTCTCTTCCCTTCAATTTTTTCTTCTATGATGTTTGTTATAAAAGAATCGTGTCGTATCAGGTGGCCAATCATATTTCCTCTCCGGTTCTCTATAGTATCTTCAACTCTTTCCAGTACTTTTTCATTTGACACCATTTCCGTCCAGCTTTTTACTTATTAATGATATTAATAAATAAAAAAACCCCTTCTCTACCCCTTCCTCTCTTCCCTTCAATTTTTCCTTCTATAATGTTTGTTATAAATGAATCGTGTCGTATCAAGTTCTACTTATTTGTACCATATTCATTAGCTCCGACCACATTTCACAGGGGCATTAACTACCAAAGAATGAATGAATTAAGAATGAGTATTTATTCGCATAATCAATTACAAGAATTGTGTTCGAAATACCGAAACAGACAGAATAGTAAAAAATAAGTTTACCAAAGCAATCATCAATTATTGAAAATAAATGAATTCAAGATATAAAAAATTGTTGATTTGGATAAAAAATAATAAGAATAAAAGGACACTAAAAAGGAAATAAAAGTCTACAAGTAAATGTTATACTTACCTACCTATCACTTTTATTGTAAGCCCTCATCATCATCAATTTAAGAGCCACGCTCTTGTCGGTGTAGCATTTTCCATTCCAGTCTATCAAAGGCCAATTCCTTGACTTCCCTATAAGACACGACGTTAACATTTTCTTTAATCTGTTCCATGTAAGCTCTTCTTGGTCTTCCCCTTTCTCCCTTTCCTTCTAGCTTTCCTTCTATGATGTTTTTAATAAATTCGTCGTGTCAAAATTGTAAGCCCTATGCTATAGTTTTTTGTCTTTAGTAaggtatttgtgtctcatcacctttttgtctgatcgcatttaccactttaatttaagctttttataaattccctaccaacgactgttccttcaaattcacttacaaaccgattctttgatcaggcaactcattccgaaaacgcacaattgacatataccatcgaagacggttctatgg

**>Pectinophora gossypiella isolate KMP-4 clone r8A_186-166 cadherin (Cad1) gene, partial sequence (KJ724995)**

AGCTATTGATGACGCGTTCGATTATCACAGACAGAATGAATTTAACATACAAGTAAGTAAGAATAACATAACATAAAGTACCTATCGCCGAAGGCAGAAGTGTGTATTTAGTACATATACCCTCAGCTATTTAAGCTCCATATAAGGCTGCGTTTCCATTGACACGGAGCTGTGCGGAGATGAGCGGAGATGTGCAGGAATCGACCAATCACCGTGAGGGAGACAGTGACAGAGCGTCTTCGTTTTTCGCTCTCTCGAACGCTGTGATTGTTCAAATCCGCACATCTATATTACGCTCATCTCCGCCCATTTCCGCGTCAGTGGAAACCCGGCCTAATAGGGGGCGAATCTAATGTCATTCTTCTTCTTCTTCTTCTAGTGCCTGTCCATTACTGGACGTTGGCCGTCAGCTTTGAGAACTCTTCTCTGTCAGAAGCAAGACGAAACAGCTGTTCTACGGTTGTAACGCCGGTCCATTCGCGGATGTTCCGCAGCCATGACTTCTTCCTCCTCCCTCGACGTCGTAATGTCATTAGCCGGGCACAAATCCTGTAAACCACCCGATAACATCCATCTTTAGATGATCTAACCGAGCTTTCTCTCGTGTTAGAACAACGTGATAGATTGAGCCGTATCGCCGTCTATAACAATCAATAATTAAAGCACATAATAACGGGTTCTTACCGCGTTTAAATGGGGATATGACACTCCCGATATTTCGACACTGTTGCAAGTGCCATGATCACGGGATGACTGATGAGATTGAAGTGGAGTAGGTAGATCCATAATTTTCTACGGGCAGACATATCTGTCTACCGCACTGCGGTTTGTCGTAGTGAGTTTGGTGCGAGTTCAGATTTTTTTATNNNNNNNNNNNNNNNNNNNNNNNNNNNNNNNNNNNNNNNNNNNNNNNNNNNNNNNNNNNNNNNNNNNNNNNNNNNNNNNNNNNNNNNNNNNNNNNNNNNNTCATAGAATCTGGGTGTGTTCCTGACATAATTCCTCCAGGTTCGCGCTCAGGACACCATGTCGGAGCCAGAGTCCAGGCATACAGCGACTGCTCAGCTGGTCATAGAACTCGAGGACGTCAACAACACACCTCCTACTCTGAGGCTGGTAAGACATCAAAATCGATCATACCACAACTCTTTTTCTTACCGGCCTCCGTGGCCCAGTGGTTGAGCGTTGTGCTCACGATCCGGAGGCCCCGGGTTCGAACCCGGTGGGGACACATCACAAAAAATCACTTTGTGATCCCTAGTCCGTCCCTTTCCTTTTCGGCGGATAAGAAAATGACAGGTATAACTTAAAATAAACTTACATGGTGTGTACTGGAATTAGCACCATTGTAGATTTGCCGCAAATGGCATTAACTCCTTAACTAACTATGCAATCATCATCGTCAGCCCATTAACGTCCCCACTGCTGGGGCACGAGCCCTCCCTATGGATGGATAGGGAGATCGGGCCTTTGACCATCACGCGGGCCCAGTGCGGATTGATGGTTATTAACGACTATGCTAATGCAGCCGGGACCAACGGCTTAACGTGCCTTCCGAAGCACGGAGGAGCTCGAGATGAAAACGGTTTTTTTTGTGGTCATCCATCCTATGATCGGCCTTCGCGAAAGTTGCTTAACTTCAAGAATCGCAGACCGAGCGCGTTTACCGCTGCGTCACCGAGCTCCTCAACTATGCAATACTTCTCCTTAACTATGTAATACTTTAATGCCATTCCTCTCTTCTTCCCGCCAAATCCTTCACCTCCTGATACAACACGACCTGCACCTTCTCTTTTATTTATTTCATAAATGTTCTCCTAGGTCTAGGTCCCTCCCTCTCTTCCCTTCAATTTTTTCTTCTATGATGTTTGTTATAAAAGAATCGTGTCGTATCAGGTGGCCAATCATATTTCCTCTCCGGTTCTCTATAGTATCTTCAACTCTTTCCAGTACTTTTTCATTTGACACCATTTCCGTCCAGCTTTTTACTTATTAATGATATTAATAAATAAAAAAACCGAAACGATCCTGTTTTATCTAAACCGATTTCTTTCTTATNNNNNNNNNNNNNNNNNNNNNNNNNNNNNNNNNNNNNNNNNNNNNNNNNNNNNNNNNNNNNNNNNNNNNNNNNNNNNNNNNNNNNNNNNNNNNNNNNNNNCCTCGCGTAAGTCCGTCTGTAGAAGAGAATGTGCCAGAGGGCTTTGAAATCAACCGGGAGATAACCGCCACGGACCCTGACACCACAGCATACCTGCAGTTTGAAATAGATTGGGACACATCCTTTGCCACTAAACAGGGGCGTGATACCAATCCAATAGAGTTCCACGGGTAAGGCTCTGGTCATCACGAATACATATACTTTTTTCGTTTCATGTTTTCCTTTCTTTTTTTTTAATCCAGTTTGTATTACATAATAATTACAGTCGGTGTAAGGAAAGGGAAAAATAAAGAGTTTATGTGACTTTATCACAGAAACAGTGTTTTATGAGCTCCCTTACAGAAGCAGGCCTGTCCACATACGCAGCACCCGATCACGAGTTGACGCGAAAGCCTTCGCACAAATTTTGAGGGAAAGGTGCTACTTCACAGAGCAACAGGGATCTCTTGATATACTTACATATACATCAATGCTTCATACCTCTTGCTGATAAGAATGAGCCTACCCTATACCTACTTATCGCGCGAAAACGATCTTTAAACACGTGGCCGTAATCGATACTAACGCATTCTACTGACTTACATCGTCATCATCATCAGCCCATTAACGGCCCCACTGCTGGCCTTCCCTATGGATGGATAGGGAGATCGGGCCTTAAACCATCACGCGGGTCCAGTGCGGATTGATGGTTATTAACGACTGCTGATGCAGCCGGGACCAACGGCTTAACGTGCCTTCCGAAGCACGGAGGAGCTCGAGATGAAAACATTTTTTAATTTTTTTTGTGGTCACCCATCCTATGACCGGTCTTACTTACTTATACATATCTCTCTTTTATTACTTCTAGAGAAGAAACTAGGAAAGTGAGTTTGCCTGGAAGAAATTTAAGCAAAAAGGCCTTTTGTTCGTTTGTGTATTTTGTATTTCTGTGTTATTATGTTCCTTTTGTGAGTAACAACATGCTGTAATGTAATAACACACTGGACGTACTCATTTATTTATTTATATATAAAAAGGTCACCTTTTTTTTTGACGTCACTTATTGAAAATAAAAGTCTAATAATGTACTTTGTATGGAACTGTCTGATTTTAATTGCCTACTTATACTTNNNNNNNNNNNNNNNNNNNNNNNNNNNNNNNNNNNNNNNNNNNNNNNNNNNNNNNNNNNNNNNNNNNNNNNNNNNNNNNNNNNNNNNNNNNNNNNNNNNNACGAGAAAGTATTTTTGGCATGTCTTGTCTACCCTCAGCGCTCTTCATTTGTCCGGCAAATCTATAGTAAGTCACTTCAAAATAAACCCATCTCCATATTATCATCATCATCTCCCTAGCATTATCCCGTTTTCCACAGGGTCCGCTTACCTAACTTGCAGATTTGACGGGTCCGGTTTTTTTATCTCCATTTATAACCAATAACCACTGTCAAAATTTACGGTTTCATAAGATTACGACTTTAGGGTGGTAATGGTGCTAATTCCTGTAAATACCATCTAATTTTATTTTAAGTTATATCTGTCATTTTCTTATCCGCCGAAAAGGAAAGGGACGGGTAATCGACAAGCATAAAATTTATGGAACACACGTCAATTTTAAGCACAAATCTAAACCAACCGTCTAAAAATTTTACATCCGTCAATAACCCGACACAGTTAAGTAGACAGCACGTCAAACGGGTTGCATACCAGAGACGTACCTTTTGATTCGCCCGGGTTATTCATTCATTCACTCATTCTTCCTAAAATTAAGAGCTGTGAATCATCCGTCCCTTTCCTTTTCGACGGATATGAAAATGACGGATATAACTTTAAATAAAATTAGGCGGTGTCTGCAGGAATTGGGGCCATTAATAAACTAATCTCAGCTTCGAGACTGCCCTCAAGATCATGTCAATGTGACAGTTCTCATATAAAAACAGAGACTTGAGCATGATCTTGAGGGCAGTCTCAGCTGAGATTCGTTTATTAATACCACCCCAAAGTCGTAATCTTATGAATCTTATGGTCCCAATCATGACCAGGCACCGAAAGATCTTTATCGCTCCATCACACAATGCCATTTGCAGCCTTATCCTTTGTGACACAGATGCGTGGATATAGAAACCATCTTCCCAAACCC

**>Pectinophora gossypiella isolate KMP-4 clone r8B_219-220 cadherin (Cad1) gene, partial sequence (KJ724996)**

CATTCCATCTTGGGAAATGCGAATACATTTTTGAGAATCGACGAAGAAACTGGCGACATATACGTAGCTATTGATGACGCGTTCGATTATCACAGACAGAATGAATTTAACATACTTACAAGTAAGTAAGAATAACATAACATAAAGTACCTATCGCCGAAGGCAGAAGTGTGTATTTAGTACATATACCCTCAGCTATTTAAGCTCCATATAAGGCTGCGTTTCCATTGACACGGAGCTGTGCGGAGATGAGCGGAGATGTGCAGGAATCGACCAATCACCGTGAGGGAGACAGTGACAGAGCGTCTTCGTTTTTCGCTCTCTCGAACGCTGTGATTGTTCAAATCCGCACATCTATATTACGCTCATCTCCGCCCATTTCCGCGTCAGTGGAAACCCGGCCTAATAGGGGGCGAATCTAATGTCATTCTTCTTCTTCTTCTTCTAGTGCCTGTCCATTACTGGACGTTGGCCGTCAGCTTTGAGAACTCTTCTCTGTCAGAAGCAAGACGAAACAGCTGTTCTACGGTTGTAACGCCGGTCCATTCGCGGATGTTCCGCAGCCATGACTTCTTCCTCCTCCCTCGACGTCGTAATGTCATTAGCCGGGCACAAATCCTGTAAACCACCCGATAACATCCATCTTTAGATGATCTAACCGAGCTTTCTCTCGTGTTAGAACAACGTGATAGATTGAGCCGTATCGCCGTCTATAACAATCAATAATTAAAGCACATAATAACGGGTTCTTACCGCGTTTAAATGGGGATATGACACTCCCGATATTTCGACACTGTTGCAAGTGCCATGATCACGGGATGACTGATGAGATTGAAGTGGAGTAGGTAGATCCATAATTTTCTACGGGCAGACATATCTGTCTACCGCACTGCGGTTTGTCGTAGTGAGTTTGGTGCGAGTTCAGATTTTTTTATTATCTGGGTGTGTTCCTGACATAATTCTGATGATGCCTCAGGTTCGCGCTCAGGACACCATGTCGGAGCCAGAGTCCAGGCATACAGCGACTGCTCAGCTGGTCATA

**>Pectinophora gossypiella isolate KMP-4 clone r9A_171-25 cadherin (Cad1) gene, partial sequence (KJ724997)**

TCCATGCTCACTTCATACAGGATAACATCCCTGTGAGCGCCGACAGTATTGAAGAGTGAGTTAACCTTAAGCCTAATAAGGATTAAATACGTTAAAATGGCAACTCTCCGCAGCGACTGAGCTAGGTTTACTCTCTTCCTCACCCCCCCTCGGTCTTACTTTAGTCTTCAATCATATGGCATCAGACGTTACACACACAGATCCACGTGCACGTTAACGTTAAAATATGAATTCACTGGTTTAGAGCCCGAGCCGGAATTCGAACCACGGACACTACGATAAAAGCGCATACCGTTCTCCGAACAGAGTTATTACGGATTTTGAAATCCAGGGTTTTTTCTTTAACAAAAATTTTAAGTTGTGCATAATATTCTTTTATTTTCCTTTTAAAGGCTTCGCAGTGACACTCAGCTGCTGCGCTCCGTCCAAGGTGTGTTGAACCAACGGCTGTTGGTCCTGAACGACCTGGTGACCGGGGTCAGCCCTGATCTCGGCACTGCCGGCGTGCAGATCACCATCTATGTGCTAGCCGGGTTGTCAGCCATCCTTGCCTTCCTGTGCCTTATTCTGCTCATCACATTCATCGTGAGGACCCGAGCGTAAGTATTCTTTTATTTTCTGGCGTAATTATTAACCTAGTTATACAATGGGTTTTGGATTTTGAAAGGACATTCGGGCAGGGACTTTAACCTGCAACCTGACAGAGAGCAGCAGTAACTGACAGTACTATTCCGGCGGAGGAGAGGAGTCCTCGCTTTGAAATAGACTTCTCTGGGCGCCATTCCACTCGTATCAGACAGGGTACTGCGGGGCGGAAGGCAAGAGGGAAACCACTGCCCTATTTTTCCCTAAAAAGTAACATGAAGAATGCTACACCGACAAGAGCTGATTCGGGCATAGGACTTTGAGGTATTTTATGTTCAAGCAGATAGTGTGTGTAGTGTGCGTAGTGAAATCTTGGGAAAGGGCGCGCGTGATTGTTCATTTTATCTGTTTATGAACATTATTGGTTCAAGTGGCTGCATGGACTCTTGTGATGGATTTTATGTTCTATAATACATGTAGGTAAGTTTTAAGGTTTATTAAGGTAAGGTATATTATTCCAAGGCAGACTTATGCAATAAACCTGTAAGAAGTCTTGTTTTATGTTGAATTATGTTGTTGACTACAGTAATACTACATATTCTCAGCTTATACTGAGTAATAGGTAATTAAAATGGCCGCATCAAAGCAATTTATCTAAGAAAGCAATATCGGTTTTTGACATTTGTTCGCATTGCGCACTTACTTTTATATGCGCAAATGTCAAATTGCTATATTGCTTTCTTGGATGAATTGTTTCGATGTGGCCATTTTAACCCCCCAGATGTTTGTTTCCAGTCTGAACCGCCGTTTGGAAGCACTGTCGATGACGAAATACGGCTCGGTGGATTCGGGGCTGAACCGAGTGGGGATAGCGGCCCCAGGAACCAACAAACACGCCATCGAAGGCTCCAACCCCATCTGGAACGAGCAGATCAAGGCCCCGGACTTCGATGCCATCAGGTCAGTTCACCAGCAGTAAAATGCTTACAGCGCAAATAAAAACTGCTCATGTTGTCTGAATCATCCCGGTCTCAGTTAGGGTATCACTAACATCCGTACGTATTTGTATAGCTACCTGTACGTATTTGTAGGGGTGTACGTACGATTACTGTCGGGAATGATTCAGATCATTATTCTGAGTTAATATAAAGTGAAAAATTTCATCTCAAAAGTGTAGAATTGAAAATAAATTTAAAATAAATACACAAAAAAAATCATGGTGATAAGTGGAATTTTCCATCGCAAAAATGAAAACATACTAAAAAGAATCGTGAATTTTTCGACGGAAAATTCCATTTAATATTAACTCAGAATCATGGTCTGAATGATCTCCCTCAATATTCGTTACGATGCCACTAACCACCCCGTTTTATATGATCGAAAATGTCACGGCTACTATCTCTTTAGAAGTTGACTCGAACTGATAGTAGTGACTTGTGATCCGTTCTCAGAATTTTAATATACCACATCTATTACATGTAGTACGTGTTGCGATTTTAGCACCCTTACTCTAACCTTCCATTTGCAGTGACACATCTGACGAGTCTGATCTGATCGGCATCGAGGATCTACCACAATTCAAGAGCGACTATTTCCCGCCTGAGGACTCGGAATCCGCTCACGCCGCCTTTAGCGACCGCACGGTAAGTTGTTTTTTTTTTTTGACGTGACTTATTTTAGTAACAACTACTTGGTCGGACAAATGGGAAGCTCTTAGGGCTCTCACCCGGAACAAAGCCTGGGGGTGCACAGTTGGGCGACATCTGAGAGGATTAATATTTGAAATAATTAATTGACCCTAGTGGGGTGACAGCGAAAAGCGCTGAATGAGAGAAATCGTCGACCAAGCCGCGGTGAGGTCGCTAATATAACTGACTCCAAAGTTAGTGATGTCGGTCAATCGACGTAGGTTTTAAACCTACGCGAAAGATGAAGATGAAAGGGAAGCCATCAGCTAAAGTCTCCAGTCCCCCAGGGCTACGATTTCGTCATACCTATTTTATTTATTTTATATGGACAGCCGATCCGGTAGCGAAAATCGCGGGTCGGTATGGTCAGATAGCTCGGATTGTCGCTCGAATGTCCCTTCAATAGGCACATAGTAAGTCACCATCTTCCTAGCATTATCCCGTTTCACACAGGGTCCGCTTTCCTAACCTGAAGATTTGACAGGTCCGGTTTTTTACAGAAGCGACTGCCTGTCTGACCTTTTACCCGCGAAGGCAAAACCAGACCAATACAGGTTAGGCCACAAACCACCGAAAATGCATTTCTCGGGAATGTGGGTTCACGATGTTTTCCTTCACCGCTAAACACATGATAATCATTTACGATCTAAACGTGAATTCGAAAACAAATTCGACAATCATTATCTTTATGATTTTCTATAGGTACATAGTAAGTACTTAATGTTTATTGTTATTCTAACGACCTCTGTGGTCCAGTGGTTGAGCGTTGGGCTCACGATCACGAAGGTCCGGGGTTCGATTCCCGGTGGGACATATCACAAAAATTACTTTATGGTTCCTAGTCTGGTTAGGACATTACCGGCTGATCACCTGATTGTCCCGAAAGTAAGATGATCCATGCTTCGGAAGCCACGTTAAGGTCCCAGTTACTACTTATTGAGGTACGTAAGTAGTCGTTACATGAGCCATGTCAGGGTTTTTGTCGGCTCAATAGTAACCCTGACACCAGTGTTGATGAGGTTGGTACTCCACCTCATTCATAACCCACACGAGAGAAGAAGAGATTGTTACTCTATGCCATGACCTAATTAGTGTTTTGTCTTTCAGCCACGCGGGAACGATGCGCCTATTGCACACAGTAGCAACAACTTCGGTTTCAACACCAGCCCTTTTAGCGCGGAGTTCACTAACAGGCGCATGCGACCATAG

**>Pectinophora gossypiella isolate KMP-4 clone r9B_58-87 cadherin (Cad1) gene, partial sequence (KJ724998)**

TGGAACCTTGTTGCTGACACAGGCGCTTCCTGAGGAAGGCAAGGAATTTGAGGTTGGTAGATATGTAATATTGTTTAACATAACGTAGCATAAAGTGTGTCATCTAAATATATAAAAGGAGAAACTGACTGACTGATATATCAACGCACAGTCTAAATGGCTAAACGTAGGCACTTGAAATTTAGAAGGGACGTAGCTTAGGTACCGTAGAGGTGCACTAAGAAAGGAATTCCCGAAATTCCCACGGGAACGGGAATTAGCGGGAAAATCCTTCCACGCGGACGAAGTCGCGGGCAAAAGCTAGTATTATATATTATGTCGTGGCCAGATCGTAACATTGATCATTTCATGATGTGTTAGACCATTCCATGAAATGGTAATTTTTCATGTAAACTGTCTGTGTAAAACGTGGTGTTTTGTATGAAGTGTCCGGGGTGTGCTCAAGGGCCTTAATAAATAAGCCTGAAATACAGTAGCAAACATGATTTAAGTAAAATTCCGTATCTCATGTACCTAGGTTACCATCCGGGCTACAGACGGCGGAACAGAACCTCGATCATATTCAACAGACTCCACTATAACAGTCCTCTTCGTTCCGACTTTGGGTGATCCGATCTTTCAAGATAACACTTACTCAGTAGCATTCTTTGGTAAGTATGTTATTTCCTGCGAGTTTAAAAAGGCCACATAGAAGCAATTCATCTAAAAAGCAACATTGCTATTGGATTTTCTGCATATAAAAGTGTAACATATAAATGCATTTTTTAGATTAATTGCTTCAATACGGTCTTTTAGACCCCCTGGTACGTAATTATTTGAAATGGCTAAGTACCTACTATTCAGCCTGCAAAGGACTGTCTCACAAAGTGATTTTGTTTCCAAGGATAATTGAGCCTAACGCTCTAACCATTAGATCATGAGATTGTATAAGAACCAACAAAATTAGGTATTTTCTGTAATGAATACACACACTTCTGCAATATTTTTTCAGTGTTTTTTATTAAGTATTATTTTTTAAATTACTTACACGTGCTGTGTCGGTAAACCACAAGGAGCGCTATCTGTGGCTAACACCAGGTTTTGGAGCATAAAACCAGAATACGGTAGTTTCCAACTAGTCAAATCAGTTACTTTTTACTTAACATCAAAACACGAAATTACTAATTTTAAGTACTTACTTATGGAATTTGTTGAAAGCAATCTGTGAAAAACGTGACAAAATGTCGCATTCATTATTACTTTTTTCTTTGTTAAAATCCATAAATAAGTTTAATAGATAAAAGAAATAGTTTTTCATCAGTTTTAGATCTATCTTTCTTTAGTAATCAGAATTTCAGAATTTATCTTTGACCTAGGAAACTACCAAATTACGTTTATTGTTACTATACTTAATTCATTTTCCAGAAAAAGAGGTTGGCTTGACTGAGAGGTTCTCGCTCCCACATGCAGAGGACCCTAAGAACAAACTCTGCACTGACGACTGTCACGATATTTACTACAGGATCTTTGGTAAGCGCAATATTCTATCATTAGCCGTTTAATTACTTTTATCCCCTTTTTATATTTTTCCCTTAATTTTCCACTTTATTAAAAAATTATATTCTATGTTATGTTAGTAAGTACCTGACAGGGGACAGCAGTAACTGTCAGTACTATAGACTACACTGTACGCTATTCCACTCCCGTCAGACAGAGTAATGTGGGGCGGAAGGCAAGAGGGAAACCACTGCCCTATTTTTCCCTAAAAAAGTAGCATGGAGAATGCTACACTGACAAGAGTGTGGCTCTTAAATTAGTGATGATGATGAACGTAACCACCACGCTGCTCCAGTGCGGGTTGATGAAGGTGTTTGGGCTAGTAGCCCGGAACCAAAGCGGCTTAATGTACATAAAGTGGATAATTAACATAAGTAGGTACCTACACCAAATTCTGTGGTCAAGGAAGGCTTCTGTAGCCAATTGGGGTTGCATTCTAGCTGGTCATTTTTTGACGCCAAATATCTTAGTAGAAATAAAACATCAAAAGACCGTTCCTTTGGCATTGACTTTAGCTGTGTTGGTCCAGGAGTTTAGATCCTTGTAAAATTCTTGTCTAGACCATACAAGACCAATATTGCAAGAACCGTTAATACCGTTCTTCTAGAAGAAGACCGACCATCGAAAGACCTTTGCAGTGACATGTAACTCATCGGTGTTGGTGATGTAGACCAGAATGAATTCTTCTTTTAACTTATAGGTGGTGTGGATTACGAGCCATTTGACCTGGACCCGGTGACGAACGTGATCTTCCTGAAATCAGAACTAGACCGGGAGACCACTGCTACGCATGTGGTGCAAGTGGCAGCCAGTAATTCGCCCACAGGAGGCGGAATACCACTCCCTGGGTCTCTTCTCACCGTCACTGTCACTGTAAGTTAAATCNNNNNNNNNNNNNNNNNNNNNNNNNNNNNNNNNNNNNNNNNNNNNNNNNNNNNNNNNNNNNNNNNNNNNNNNNNNNNNNNNNNNNNNNNNNNNNNNNNNNCATTTTTTAGATGAATTGGGTCCATGGGGGTCCATGTGGCCTTTTTAACCCCCCCAGGACATGAATACACGAGGACATTATCCCTGTAGGGCACATCATTCGGACACAGGAAGCCATAGTTTAACTAACTCAAGCACGTTATTTCAGATCGCAGAAACGTTCAGCGTTGGCTTCAACATGACCTGCAATATAGATCAGGTGCTGCCGGGCACCAACGACGCCGGGGTGATTCAGGAGGCCATGGCGGAAGTCCATGCTCACTTCATACAGGATAACATCCCTGTGAGCGCCGACAGTATTGAAGAGTGAGTTAACCTTAAGCCTAATAAGGATTAAATACGTTAAAATGGCAACTCTCCGCAGCGACTGAGCTAGGTTTACTCTCTTCCTCACCCCCCCCTCGGTCTTACTTTAGTCTTCAATCGTATGGGCATCAGACGTTACACACACAGATCCACGTGCACGTTAACGTTAAAATATGAATTCACTGGTTTAGAGCCCGAGCCGGAATTCGAACCACGGACACTACGATAAAAGCGCATACCGTTCTCCGAACAGAGTTATTACGGATTTTGAAATCCAGGGTTTTTTCTTTAACAAAAATTTTAAGTTGTGCGTAATATTCTTTTATTTTCCTTTTAAAGGCTTCGCAGTGACACTCAGCTGCTGCGCTCCGTCCAAGGTGTGTTGAACCAACGGCTGTTGGTCCTGAACGACCTGGTGACGGGGGTCAGCCCTGATCTCGGCACTGCCGGCGTGCAGATCACCATCTATGTGCTAGCCGGGTTGTCAGCCATCCTTGCCTTCCTGTGCCTTATTCTGCTCATCACATTCATCGTGAGGACCCGAGCGTAAGTATTCTTTTATTTTATGACGTAATTATTAACCTAGTTATACAATGGGTTTTGGGTTTTGAAAGGACATTCGGGCAGGGACTGTAACCTGGAACCTGACAGAGAGCAGCAATAACTGACAGTACTATTCAGGCGGAGGAGAGGAGTCCACGCTTTGAAATAGACTACTCTGGGCGCCATTCCACTCGCATCAGACAGGGTACTGCGGGGCGGAAGGCAAGAGGGAAACCACTGCCCTATTTTTCCCTAAAAAGTAACATGAAGAATGCTACACCGACAAGAGCTGATTCGGGCATAGGATTTTAAGGTATTTTATGTTCAAGCAGATGGTGTGTGTAGTGTGCGTAGTGAAATCTTGGGAAAGGGCGCGCGTGATTGTTCATTTTATCTGTTTATGAACATTATTGGTTCAAGTGGCTGCATGGACTCTTGTGATGGATTTTATGTTCTATAAGACATGTAGGTAAGTTTTTAGGTTTATTAAGGTAAGGTATATTATTCCAAGGCAGACTTATGCAATAAACCTGTAAGAAGTCTTGTTTTATGTTGAATTATGTTGTTGACTACAGTAATACTACATATTCTCAGCTTATACTGAGTAATAGGTAATTAAAATGGCCACATCAAAGCAATTTATCTAAGAAAGCAATATCGGTTTTTGACATTTGTTCGCATTGCGCACTTACTTTTATATGCGCAAATGTCAAATTGCTATATTGCTTTCTTTGATGAATTGCTACGATGTGGCCATTTTAACCCCCCAGATGTTTGTTTCCAGTCTGAACCGCCGTTTGGAAGCACTGTCGATGACGAAATACGGCTCGGTGGATTCGGGGCTGAACCGAGTGGGGATAGCGGCCCCAGGAACCAACA

**>Pectinophora gossypiella isolate KMP-5 clone r10A-r10C_20-21 cadherin (Cad1) gene, partial sequence (KJ724999)**

ATGCTGTCGCCTAACAACGTAACCGGATTCCTCCAGACAGCAATGCCTTTAAGAGGATATTGGGGGACTTACGATATAAGTATACTGGTAAGTCGAATGAGTCGAATGGTAATGACCTCCGTGGTCCAGTGGTTGAGTGTTGGGCTCACGATCCGAAGGTCTTTGGCGGGTCAATAGTAACCCTGACACCAGAGTTGATGAGGTTGATAATCCACCTCACAACCCACACGATAGAAGAAGTGATCATAGCTTCTTTTGACGTGATCTATCCCTAGCATTCACACATGGCAATGGAAAGCACTGAAGGCCGATCGCTTATACCAATGCCCGTTTTTCTTATTGTACCTATTAAATTTGTCTGTGAAAGACACACATACAATACAAGGAACAAAAATAAAATTGCTATCCATAATTCTAGGCTAAGTAAATTAAATTCATCTTCTTTGGGGTTATGTACTTACACGTTTTTACAATAAAAAAACAGACAGTATTTTGAATAAGTACGTCAGAACATAAATTTAAAGCTCATGTGAAACTTACTTTATGTAAAAAGCTTATAATTTACTAATAAGATCAATGATTACCCAAATGATAAAAGTGCCTGGTATTCAATGTGTTTCTCTAGTTATTAAATAACTTGTAATGTACTTATGTATAAGATGTGTTGCTGTTGCAGTTTCTTGCCATTTCTTCTCCAGCTATAACACCTTGCGGAATGACGTAGTTTCAAAAAATATTAAATTGACCTTCAATAAGTTTATCTATGATAATTACGTTGAATAAATGATTCTGATTCTGTCTTTGTGCATGACAGGCGTTCGACCACGGTATTCCTCAGCAGATATCTCATGAGGTGTATGAATTGGAAATTCGACCTTACAATTACAATCCTCCCCAGTTCGTTTTTCCTGAATCCGGGACGATTCTACGACTGGCTTTGGTAAGATTTCTATTATTGTAACTCAAAAATTGCGATAATAATTTCAAAATGGCGTTTTAAGAAAAAGTAAGCAGATGGCGATAGTGTATGGCCACCGTTAGAATCCGGAAGTCTAAAATAGTCACATACAATTCATCTAAAAAGCTATAATTTGATGCCATTTGCGCATAAAATAAAATAAAATATCCACATACTTAGAAGGAATTCATCTAAAAAAGCTATAATATGATGACATTTGTGCATAAAATAAAATAAAGTGCGCAGTCTGGGGGTTCCAAATGGCCACATAGAAGCAATTCATGTAAAATGCTATAATTTGATGACATTTGCGCATAAAATAAAGTGCGCAATATCAACAAGTATCAAATAACAATATCGCTTTTATCGCTTTATTTGACAGCTGTTAGACTAATAACACCTAAACGCAAGCCAGCATCATCACATAATTAAACCCTATAACTATGAGATTCAAAAACATCAAATCTAGTTGCTTTTAGAGATAGTAGTAGATACGTACAGTCAGAAGCAAATAGTTAGTGACAGTCAAAGTATACATAATAGTTAGCAACATCCACAATGACCAATAATTAGGGATAATCAAGGTGGCCATATAGATAGTAACACTCTAAGTGACCAAATACATCGGCACAACTTTTTGAACACGCAGGTTGTGCCGATGTTTTTCGTTACTTAGAGTGTTACTATCTATACGGCCGCCTTGAATGTCCCTAATTATTGGACATTCTGGATCTATATTCGCATGCCAACTCTTCTTTCTTATGGCTCTGTGGTTTTGATTCAAATCTCTGCCGAATTATTAACATTCTTGAAATACGTTACAGGAACGCGCAGTGGTAAATAATGTTTTGTCACTTGTAAACGGTGACCCGTTAGACAGGATACAAGCAATTGACGAC

**>Pectinophora gossypiella isolate KMP-5 clone r10A-r10C_169-170 cadherin (Cad1) gene, partial sequence (KJ725000)**

ACTTTGGGTGATCCGATCTTTCAAGATAACACTTACTCAGTAGCATTCTTTGGTAAGTATGTTATTTCCTGCGAGTTTAAAAAGGCCACATAGAAGCAATTCATCTAAAAAGCAACATTGCTATTGGATTTTCTGCATATAAAAGTGTAACATATAAATGCATTTTTTAGATTAATTGCTTCAATACGGCCTTTTAGACCCCCTGGTACGTAATTATTTGAAATGGCTAAGTACCTACTATTCAGCCTGCAAAGGACTGTCTCACAAAGTGATTTTGTTTCCAAGGATAATTGAGCCTAACGCTCTAACCATTAGACCATGAGATTGTATAAGAACCAACAAAATTAGGTATTTTCTGTAATGAATACACACACTTCTGCAATATTTTTTCAGTGTTTTTTATTAAGTATTATTTTTTAAATTACTTACACGTGCTGTGTCGGTAAACCACAAGGAGCGCTATCTGTGGCTAACACCAAGTTTTGGAGCATAAAACCAGAATAGGGTAGTTTCCAACTAGTCAAATCAGTTACATTTTACTTAACGTCAAAACACGAAATTACTAATTTTAAGTACTTATGGAATTTGTTGAAAGCAACCTGTGAAAAACGTGACAAAATGTCGCATTCATTATTACTTTTTTCTTTGTAACATCCATAAATAAGTTTACCTAATAGAAAAAAGAAAACGTTTTTCGTCAGTTTTAGATCTATCTTTATTTAGTAATCAGAATTTCACAATTTATCTTTGACCTAGGAAACTAACAAATTACGTTTATTGTTCTTAATTCATTTTCCAGAAAAAGAGGTTGGCTTGACTGAGAGGTTCTCGCTCCCACATGCAGAGGACCCTAAGAACAAACTCTGCACTGACGACTGTCACGATATTTACTACAGGATCTTTGGTAAGCGCAATCTTCTATCATTAGCCGTTTAATTACTTTTATCCCCTTTTTATATATTTCCCTTAATTTTCCACTTTATTAAAAAATAATATTCTATGTTATGTTAGTAAGTACCTGACAGGGGACAGCAGTAACTGTCAGTACTATAGACTACACTGTACGCTATTCCACTCCCGTCAGACAGAGTAATGTGGGGCGGAAGGCAAGAGGGAAACCACTGCCCTATTTTTCCCTAAAAAAGTAGCATGGAGAATGCTACACTGACAAGAGTGTGGCTCTTAAATTAGTGATGATGATGAACGTAACCACCACGCTGCTCCAGTGCGGGTTGATGAAGGTGTTTGGGCTAGTAGCCCGGAACCAAAGCGACTTAATGAACATAAAGTGGATAATTAACATAAGTAGGTACCTACACCAAATTCTGTGGTCAAGGAAGGCTTCTGTAGCCAATTGGGGTTGCATTCTAGCTGGTTATTTTTTGACGCCAAATATCTTAGTAGAAATAAAACATCAAAAGACCGTTCCTTTAGCATTGACTTTAGCTGTGTTGGTCCAGGAGTTTAGATCCTTGTAAAATTCTTGTCTAGACCATACAAGACCAATATTGCAAGAACCGTTAATACCGTTCTTCTAGAAGAAGACCGACCATCGAAAGACCTTTGCAGTGACATGTAACTCATCGGTGTTGGTGATGTAGACCAGAATGAATTCTTCTTTTAACTTATAGGTGGTGTGGATTACGAGCCATTTGACCTGGACCCGGTGACGAACGTGATCTTCCTGAAATCAGAACTAGACCGGGAGACCACTG

**>Pectinophora gossypiella isolate KMP-5 clone r10B_86-167 cadherin (Cad1) gene, partial sequence (KJ725001)**

TTCATCCCAAAATAGGGTGTCCTTCATATTCCTGAACGATGTGGAGACGGTTGAGAGTAACAGAGACTTTGTAAGTACAAAAAGTCAAAAAAAAAAGAAACAAGTAAAGTTTGTAAGTCTATATTTTATCGTCGGATGATGATGATCACCATCATCAATTTTATTGGAAAATAAGTAAGGAAAATAATAAGTAATATTGAAGTAAGTATTGAAGTCGGTCTTAAGATCACCTTCGGGTTTAAGATAATGTCTTACCCCCACTGCAGCACGAAGTTTATTACGTAGCTCGCTCAGCCTTCACCATACCTGGATATCTTATGAAGCGGGATGTTTCACCGAGTGCCGCCGAATTTACAATAAGCGGAAACTTTGCGAATTCGCGCCGAGACGGCGACTTGACATAGATTCGCAAATGTCAACACTCCATTGCGCTGCGGTCGCTTTTACAGACGCAATATAATATGCTAGAGCCAACCACAGATTCTTAAAAACATTTCCGTCACAACAGCTGGGTGAGTTAAAGAGGCCACATTGAAGCAATTCATCTAAAAAACCATGATTGCTATTTGACAGTTTTTTAAACAGTTTTAAGACAGTAATTAAACAGAAATTTAGTAAAAAAGGTTATTATAGGCTAGTGATTGATTATTATAGTGAGTGATTATTTAGAAGATATAAATGCATGATATTAACTGTCTGGGAACTGATTTTAGGCAGCCAAATTACGTCCGTAAAAACGTCTAGTGCCCTGTCCCGAGGTTTTTCTTGCAGCTTCTCTTCTTCGGCTATACACGTTGTGAAGTAGTTTTCGGCGGATGAGACGTTCGTTATGTAAAAAATGCCGATGCAAGGTATAACTATGATACCTACCGAATACAGAATGTTTTTGAATTTGACATTGCGCACTTTTACATACGAAAATGCATTATTGCATTATTGCTTTTTTAGATCTTCTATCGTGTGGATTGTGAAGTGAACCAGCGTCAACCCTGGTGTCAGGGTTATTATTGAGCCGCCAAAAGCCCCTGACATGGCTCATGTAACGTTTACTCACTTACATCAGTAAGTAGTAACCGGGACCAACGGCTTAACGTGCCTTCCGAGGCACGGATCATCTTACTTTCGGACAATCAGGTGATCAACCTGTAATGTCCTAACCAAACTAGGGATCACAAAGTGATTTTTGTGATATGTCCCAAACGGGTTTCGAACCCGGGACCTCCGGATTGTGAGCCCAATGCTCACCACTGGACCAGAGAGGCCATTTTTTAGATGAATTGGGTCCATGGGGGTCCATGTGGCCTTTTTAACCCCCCAGGACATGACTACACGAGGACATTATCCCTGTAGGGCACATCATTCGGACAGCCTGAAGCCATAGTTTAACTAACTCAAGCACGTTATTTCAGATCGCAGAAACGTTCAGCGTTGGCTTCAACATGACCTGCAATATAGATCAGGTGCTGCCGG

**>Pectinophora gossypiella isolate KMP-5 clone r10C_24-85 cadherin (Cad1) gene, partial sequence (KJ725002)**

ACGGCTGGCATTTCCACTTCCGATAACATCAACAGGGAACTACTCACCGTTCGTGTAAGTTCTCCTTCTCTCTCTCTATTTAAGAGCTGCGCTCTTGTCGGTGGAGTAATCGCCATTCCTCTCTTCTTCCCGCCAAAACCTTCACCTCCCGATACGACACGACCTGCACCTTCTCTTTTATTTGTTTCATAAATGTTATCCTAGGTCTACCCCTTCCTCTCTTCCCTTCAATTTTTCCTTCTATAATGTTTGTTATAAATGAATCGTGTCGTATCAAGTTCTACTTATTTGTACCATATTCATTAGCTCCGACCACATTTCACAGGGGCATTAACTACCAAAGAATGAATGAATTAAGAATGAGTCTTTATTCGCATAATCAATTACAAGAATTGTGTTCGAAATACCGAAACAGACAGAATAGTAAAAAATAAGTTTACCAAAGCAATCATCAATTATTGAAAATAAATGAATTCAAGATATAAAAATTTGTTGATTTGGATAAAAAATAATAAGAATAAAAGGACACTAAAAAGGAAATAAAAGTCTACAAGTAAATGTTATACTTACCTACCTATCACTTTTATTGTAAGCCCTCATCATCATCAATTTAAGAGCCACGCTCTTGTCGGTGTAGCATTTTCCATTCCAGTCTATCAAAGGCCAATTCCTTGACTTCCCTATAAGACACGACGTTAACATTTTCTTTAATCTGTTCCATGTAAGCTCTTCTTGGTCTTCCCCTTTCTCCCTTTCCTTCTATGATGTTTTTAATAAATTCGTCGTGTCAAAATTGTAAGCCCTATGCTATAGTTTTTTGTCTTTAGTAGGTATTTGTGTCTCATCACCTTTTTGTCTGATCGCATATACCACTTTAATTTAAGCTTTTTATAAATTCCCTACCAACGACTGTTCCTTCAAATTCACTTACAAACCGATTCTTTGATCAGGCAACTCATTCCGAAAACGCACAATTGACATATACCATCGAAGACGGTTCTATGGCGGTGGACTCCACTCTGGAAGCCGTCAAGGACTCGGCGTTCCATCTGAACGCGCAGACCGGCGTCCTCATACTGAGGATACAACCTACTGCCAGCATGCAGGGCATGTTCGAGTTCAACGTCATCGCTACTGATCCAGGTAATGGGGCACAGCTACGCAAGGACTTTGAGGCAAGGGGGTAACAGTGTTGTCTGAAAAAAAGGCGGTGATCGGCGTCCTCACACTGAGGATACAGTCACAGGATTTGTGTTCCACGTCATCAGCTGATTCAGAGAGGCGTGCGCTATAGAATCATAACATAAACATAAACAGCCTATATACGTCCCACTGCTGGGCACAGGCCTCCCCTCAATCAACCGGAGGGGTATGGAGCATACTCCACGCTACTCCAATGCGGGTTGGTGGAGGTGTTTTTACAGTTTGTAGAACGCTTGCCTCTCACTTTGAGGTCGCAGGTTCGAATCCAGCACAGGCCTAAACCAATTATTGTCGAATTTGTTTTCGAATTTATGTTTGGATCATAAATGATTATCACGTGCTCAACAAGAGAAATGCATTTTCGAAGGTATGTGACCTAACCTGTATTGGGCTGGTTTTCCCTGGTTGGAAGGTAAGACATGCCTGTCTTACCTTTCGCTTCTTTAAAAACCGGACTTGTCAAATCTTCAGGTTAGGCAAGCGGACCCTGTGAAATACGGATTAAGGCTAGGGAGATGATGGTGATGAGTCACTTCCAAGTTTTTAACCAAAAGGATTGGATTCGGATTCGTAAATGTAAATATTAGACCTCTGTAACATAATAGCCACATAGCTAGGTGATCAAGGAACCAAAGACAATACTTACTACCTTATTCAAGATTCATTTCCTGAAGAATGTAAAATTTCCAAACACAAAAATCAGTGAGTACTTATTTTATATTCTTACTTATTCCAGATGAGAAGACAGATACGGCAGAGGTGAAAGTCTACCTCATTTCATCCCAAAATAGGGTGTCCTTC

**>Pectinophora gossypiella isolate KMP-6 clone r11A_20-49 cadherin (Cad1) gene, partial sequence (KJ725003)**

ATGCTGTCGCCTACCAACGTAACCGGATTCCTCCAGACAGCAATGCCTTTGAGAGGATATTGGGGGACTTACGATATAAGTGTACTGGTAAGTCGAATGAGTCGAATGGTAACGACCTCCGTGGTCCAGTGGTTGAGCGTTGGGCTCACGATCCGAAGGTCTTTGGCGGGTCAATAGTAACCCTGACACCAGAGTTGATGAGGTTGGTAATCCACCTCACAACCCACACGATAGAAGAAGTGATCATAGCTTCTTTTGACGTGATCTATCCCTAGCATTCACACATGGCAATGGAAATCACTGAAGGCCGATCGCTTATACCAATGCCCGTATTTCTTATTGTACCAATGCCCGTATTTCTTATTGTCTGTGAAAGACACACATACAATACAAGGAACATAAATAAAATTGCTATCCATAATTCTAGGCTAAGTAAATTAAATTCATCTTCTTTGGGGTTATGTACTTACACGTTTTTACAATAAAAAAACAGACACTACTTTGAATAAGTACGTCAGAACATAAATTTAAAGCTCATGTGAAACTTACTTTATGTAAAAAGCTTATAATTTACTAATAAGATCAATGATTACCCAAATGATAAAAGTGCCTGGTATTCAATGTGTTTCTCTAGTTATTAAATAACTTGTAATGTACTTATGTATAAGATGTGTTGCTGTTGCAGTTTCTTGCCATTTCTTCTCCAGCTAGCCACCTAGCGGGATGACGTAGTTTAAAAAAATATTAAATTGACCTTCAACATGTAATGTACCTGTCTGTCTGTGTCTGTGGTGTCTGGAAGTAATAAATGTTTCTTTCTTTCTTTTTTTCTTTCAACAAGTTTATCTATGATAATTACTTTGAACAAACGATTTTGATTCTGTCTTTGTCCATGACAGGCGTTCGACCACGGTACTCCTCAGCAGATAT

**>Pectinophora gossypiella isolate KMP-6 clone r11B_171-172 cadherin (Cad1) gene, partial sequence (KJ725004)**

TCCATGCTCACTTCATACAGGATAACATCCCTGTGAGCGCCGACAGTATTGAAGAGTGAGTTAACCTTAAGCCTAATAAGGATTAAATACGTTAAAATGGCAACTCTCCGCAGCGACTGAGCTAGGTTTACTCTCTTCCTCACCCCCCCTCGGTCTTACTTTAGTCTTCAATCATAGGGCATCAGACGTTACACACACAGATCCACGTGCACGTTAACGTTAAAATATGAATTCACTGGTTTAGAGCCCGAGCCGGAATTCGAACCACGGACACTACGATAAAAGCGCATACCGTTCTCCGAACAGAGTTATTACGGATTTTGAAATCCAGGGTTTTTTCTTTAACAAAAATTTTAAGTTGTGCATAATATTCTTTTATTTTCCTTTTAAAGGCTTCGCAGTGACACTCAGCTGCTGCGGTCACCGGGTGAGAGCCTTCAGCGCTCCCCATTTGTCCGGCCAAGTAGTTAATGCCATCTGCGGCAAATCTACAATAAGTCACGTCAAAAAAAAAAAAAAAAAAAAAAAAAAAAAAGCTGCTGCGCTCCGTCCAAGGTGTGTTGAACCAACGGCTGTTGGTCCTGAACGACCTGGTGACCGGGGTCAGCCCTGATCTCGGCACTGCCGGCGTGCAGATCACCATCTATGTGCTAGCCGGGTTGTCAGCCATCCTTGCCTTCCTGTGCCTTATTCTGCT

**>Pectinophora gossypiella isolate KMP-6, KMP-7 clone r12A_221-222 cadherin (Cad1) gene, partial sequence**

ACTCATTCCGAAAACGCACAATTGACATAATACCATCGAAGACGGTTCTATGGCGGTGGACTCCACTCTGGAAGCCGTCAAGGACTCGGCGTTCCATCTGAACGCGCAGACCGGCGTCCTCATACTGAGGATACAACCTACTGCCAGCATGCAGGGCATGTTCGAGTTCAACGTCATCGCTACTGATCCAG

**>Pectinophora gossypiella isolate KMP-7 clone r12B_168-187 cadherin (Cad1) gene, partial sequence (KJ725005)**

ACCAGCTCGTTGTCAAGTTCAAGGAGACTGTACCCAAGGACTATCACGTCGGCAGACTGAGGGCTCACGACCGGGACATAGGAGACAGCGTTGTGTAAGTATTGTAATATGCTACTCTCATGAGTGTCGATTTGTGTAAGTAAATGAATTTGGTAGAAACAACTAAACGAGTTATGAACCTTGTCACTTAAACTTAAACTTGTCTTTAACTCGACCTGTATCCCTCTTATTTGAACAGTCGCTCAGCAAATGCCGAGTACCAAATGTATGGAAGCGCGCTTATATAACACCAATTTTTAAAAAAGGAACCCGTACCGAAATATCAAATTATAGGCCTATTTCTAAACTTTGTTTACTTTCGAAGATCTTTGAGAAAATAATCTATGGGCAGGTATACCCAGCATTATCTTCCAGTCTAAGCCCGTCTCAGCACGGCTTCTTAAAGGGACGCTCGGCTGTGTCAAATTTAGCCCTACTAAATACCTTCGTTACTGCAGCCATGGCTGACGGCCAGCAGGTCGATGTCTATTGAATATGCACCCTTATAATCGTGTTATCGTGGGCAACACTACTAGTGGTCATAGACCTTTTTACTTTTTACTTATTATAGACTTTTGTGCTCTCTGCCCGTTCTCTCTTGCATTTTACCTGTCAAACGTAATGGCCGTACTTTTACCTTTATAAGTTAATCCTATTGAATCTCACTGACTGTATATTTATCATGATATCCTAAAGTAATAATAAATTTTATTTGTGGCTCACAAGAATATTGTTTTGATAATTCCGGTCAACCACCATTGGCTTTTGTCGAAAAGCNTTTTCAATGTCATATACACGGATTACAGCAAAGCTTTTGATCGCATTCAACATGATATTCTGCTTAAGAAACTCTTCTTAGTTGGTATCCAGGGGGATTTGCTGAGATGGTTTGCCTCTTACGTAGACAATCGCTCTCAGGCAGTCGTAATAAACAATTATATTTCTAGTTGGGTTTCCACCCCTAGCGGCGTTCCCCAAGGATCCTTGCTTGGTCCATTGCTATTCGTCATCTTTGTAAATGATATTGACAGTTGTCTCCGATCGGCTCACCTGTTAAGTTTTGCTGACGACATGAAGATTTTTACAAAAATCTCTTCTTCCGAAGATGCTCGACTACTTCAGGCGGACCTAAGACGTTTGGAGGTGTACTGTCAGCAGAACAGACTAGACTTAAACCCAGCTAAATGTTCGACAATGACATTCACACGTAAGCGGAACCCCATTTGTTACAACTACGTCCTCAAGTCGCAGATACTCCGGCGGGTTACGAGTGTGCGCGATCTGGGTGTTATCCACGACTCCCAATTGCTGTTCGATGAACACATCGAGGCCATCATTTCTAAAGCCACGAAATCTTTAGGTTTTTTAATGCGTAATTCAAAAGATTTTACTAATGCAAAAACTTTCAAGATTTTATATTGTTCATTTGTGCGTAGCCATTTGGAATACGCGTCACAGATTTGGAATCCAATGTACGCTACATACATTAGTAGACTTGAAAATGTTCAAAGAAAATTTATACGTTATTTATGCATTCGTACAAAAACTCCTTATAATTCAATAAGTTATGCCGATGTGTGTATTAGACATCACATGTTACTGTTACAAGACAGACGTTCTATTTCTGATGTTATTTTTCTAATGAAAATTTCTCTTAGTCTCATCGACTGCCCCGAGCTGCTctcacttttgaacataaggatcccagctaggtgcctacgtaattcccatcttctttataattcaccatgcaccaCtaattacaggtcgaatacgtttatgcttcgtgcagttaaaacttttaattctttatctagcaaactggacgtagaCntattcaacgatagttgcgagtcagtaaggcgtcaactcacaaagtacctttgtaacaaaaacaattaaaaattgtatataattaatagttttgttattgtatacatctaactatttgttgtttttatcatttaacttatattgcgcgtgcgggactcaatcttctacaacgttgcttactgtcaagactcaacgcttttttttttgtaactaattattctaattctatttgtgagaacctttaattggttttgatgttgttattccagtctttaacttgtttttgataactgttggttctcctagaattaaaaaataaaaataaaataaaaataaaataaacttttctggcaaattgaactgtaagtatcactaaatatcaaatatgttaacgtgacaaggttctaaagtgggtcatctttgcacaaaaatcatattgcctggcggcgtgtttttgtcgctccatctttgtgcatttaagcttaagcttatggtaatcatgtttggtaaaaacattccggctcgaagccatattatggctatatgctcctctgggctttattccaaagaatgaacacataaacagcctataaacgtcccactgctgagcacaggcctcccccaaatcaaccggagggggcaatgagcatactccaccacgatgctccaccaaacaacattaacacaacacttatactacaacacaatactgttaaacgattaagtatacaacattgtttgtgttgacaggcattccatcttgggaaatgcgaat

**>Pectinophora gossypiella isolate KMP-7 clone r12C-r12D_227-228 cadherin (Cad1) gene, partial sequence (KJ725006)**

TACTGGTGACGGTGCTTCTGACCTTCGCAACATCAGTTTTCGGGCAAGAAAGTAATCATCATCATTAATTTAAGAACCACGCTCTTGTCGGTGTAGTATTCTCCATTATTGTCTATTAACGTTGAAAGAAAGAAAAGGAATAATCTATCCAGAATAATCTAATTAATGTCGTAAAACCGAATGGTCTTTTAAAATCCGAACCCCATGGTCTGGAAATCTGAGTCTCAGTGCGCTTTAGCCTGTAATAAACACTTAAGTACTTACCTGTGAGATGCAGGGGTTACTGTTATGTACATTTTTTTCACAGCTTAAAGAGAACGCTGACATCAGTAAGTACTTACCTGTTAAAATGTGATATATCTACCTTCAGACGTTTCCGATAATCCTTTTGAGGTTCGGCCTAAAAAGGTGGCTGTGGGCAGTGGCCCGTTTACGCAGAGCCAAATCTTATGATGGATAGGACCTATTAACCATTATCGACTAAGCACCATCGTAACTACCTATCAGAATTTTAGTGACATCGTAACAAATACGTGATTCTGAGTTGATATCAAGTGTGACATGTTTTGTCTTCTTAAATTTCATTTCTATACTTTTGCGATGGAAAATTCCACTTGATATTACTCAGAATATTGAGCTGAATCATCCCTCAAAGTTTTCGTTACGATGTCACTAACACCCTGTCATATATCCTTACCTAATTTTATAAAATTGACAGCAGCATCGTCGAGATGTTACTACATGACTGACGCTATTCCGAGAGAACCGAAACCGGATGATT

**>Pectinophora gossypiella isolate KMP-7 clone r12C_89-10 cadherin (Cad1) gene, partial sequence (KJ725007)**

GGAACCCCAGAAATTGTCCAGCCGATGGTTATAGGATCTTTTAACCTGCTAAGTCCAGAGATCCGGAATGAAAACGGGGCGTGGTACCTTTATATAACCAATAGGTAAAGACTTCGTATCATATCTAACCTATAGGATCCTTTCAATGCAATGGTCACCTCTTCATCTTTCCATCTTCCCCTGCTTAAGGTGGATGCCGAAGTATGGATCATCTTACTTTCGGACATTTGGTCCAATGTGGAATTGGTGGACAATTTGCAACCTTTTAGACGATAGACTTTTGAAGCCAACAAAAACATGCAACAAACCACCACCACCGAAATAACGTATATGTACAGAAGGCACTTTGAAACTTCAATCAGCAACAATGACTTTAAATCTAATGAACACATGCGGTGTCCAAATTCCAATGTCATACGTACCACATTAAGAATGAAAGTTCAATGTGTCTGTGAAAATGTTAATTCTAGTTCTGAACTTGAAATCGCAGGCAAGATTATGAAACACCAACAATGCGTCGGTATACATTCGACGTCCGAGTGCCAGACGAGACTCGTGCCGCACGAGTGAGTCTCTCCATCGAAAACATTGACGATAACGACCCTATCGTCAGGGTGCTAGACGCTTGCCAAGTGCCGGTGAGTCAACAACCTATTCAATTTGACTAGAAAGAACAAAAATCCGTAAACACATTATCCGTGTAAACATACACCCGTAATTCGTAAAGGCAATTGGGCAGAGCTACAAGTAATCAAAGACAACTTGCAGCCACTCTTAATACGAAGTTAATAAGACAAAATAATATCATCTGTCGCAAATAAAACAAAAACATAACAGAACATTTAAGCAGTCTGAGGTACATAAAACAACAATAGAACTAAAAATAGAGAAGGTCCTAATATTGATCCCTGCGGAACACCAATTTCCACAATAATTGTCCTTTTTACCAGATTAGTACTCTCATCGAAAAATATTGTCACTTATATTTTACTTTTGACCAGTGATAGAAATGAATTAAGTAAGTACTACTTCTTTTGTATTTTATGCAAGAATTCATAGAGAGGTTGTTGAGAACGTTGAAGCCTTTGATATATATAATATTATATTATGTTACTAGTTGAAGCAGTGTTCTGTAGCTGTTCAATTTGTTATTTCCCAGGAATTGGGGGAGCCTCGACTAACTGACTGCGTTTACCAAGTGTCAGACGAAGATGGGAGGCTTAGTATCGAGCCCATGACATTCCGCCTCACATCAGACCGTGAAGACGTACAGATATTCTATGTGGAGCCAGCTCACATTACTGGTGATTGGTTCAACATGCAAATTACTATCGGTATCCTATCAGCGCTTAACTTCGAAAGCAACCCGCTGCACATCTT

**>Pectinophora gossypiella isolate KMP-7 clone r12D_186-73 cadherin (Cad1) gene, partial sequence (KJ725008)**

AGCTATTGATGACGCGTTCGATTATCACAGACAGAATGAATTTAACATACAAGTAAGTAAGAATAACATAACATAAAGTACCTATCGCCGAAGGCAGAAGTGTGTATTTAGTACATATACCCACTCCTTAGCTATTTAAGCTCCATGTAAGGCTGCGTTTCCATTGACGCGGAGCTGTGCGGAGATGAGCGGAGATGTGCAGGAATCGACCAATCACCGTGAGGGAGACAGTGACAGAGCGTCTTCGTTTTTCGCTCTCTCGAACGCTGTGATTGGTCAAATCCGCACATCTATATTACGCTCAtctccgcccatttccgcgtcagtggaaacccggcctactagggggcgaatctaatgtcattaaccgggcacaaatcctgtaaaccacccgataacatccatctttagatgatctaaccgagctttctctcgtgttagaacaacgtgatagattgagccgtatcgccgtctataacaatcaataattaaagcacataataacgggttcttaccgcgtttaaatggggatatgacactcccgatatttcgacactgttgcaagtgccatgatcacgggatgactgatgagattgaagtggagtaggtaatccataattttctacgggcagacatatctgtctaccgcaCTGCGGTTTGTCGTAGTGAGTTTGGTGCGATTTCAAATTTTTTTTATTATCTGGGTGTGTTCCTGACATAATTCTGATGATGCCTCCGGTTCGCGCTCAGGACACCATGTCGGAGCCAGAGTCCAGGCATACAGCGACTGCTCAGCTGGTCATAGAACTCGAGGACGTCAACAACACACCTCCTACTCTGAGGCTGGTAAGACATCAAAATCGATCATACCACCACTCTTCTTCTTACCGGCCTCCGTGGCCCAGTGGTTGAGCGTTGTGCTCACGATCCGGAGGCCCCGGGTTCGAATCCCGGTGGGGACACATCACAAAAAATCACTTTGTGATCCCTAGTTTGGTTACGACATTACAGGCTGATCACCTGATCGTCCGAAAAGTAACATGATCCGTGCTTCGGAAGGCACGTTAAGCCGTTGCTCCCGGTTACTACTTACTAATGTAAGTATGTAGTCGTTACATGAGCCATGTCAGGTGCCTTTGGCGGCTCAGTAGTAACCCTGACACCAGGGTTAATGAGGTTGGTATTCCACCTCACAACCCACACGATAAGAAAAAGGTAGGTAGTACGTACGTAGGTCAGCTGGAAGAAATCTCTTTTTTAGAGATAAGCTTACCTGTACTCTCTGATTTCTTTGTGTGTTTCTTTCTATATTCTATTGTCTAGAAATAAATAAAAATAAGAAGATACCATCACTCAAACGTAGAACAATCAAAAAGCAAGCTGCTGAATCAACTACGATCCTTTTTTCTTTGACGTGATTGATTCCTGCAGACACCTCCTAATTTTATTTCAAGTTATGCTCACAATTTTCTTATCCTCCGAAAAGGAAAGGGACGGATGATTGACAACTGTTAATTTTAAAATTAAAGAACCATCCGGGCGAATACAATAGGCATCTCGCTATATGCAATCTCTCGCTCTACTGATGTTTGACGTTTGCTGGCAGCTTTTTGTCGGGTTATTGGCCAATATAAAATTATGGAAGGATTTTTAAAAATTTCTGCCAAAAATTGACGTGTGTTCCATAAATTTTATGCCTGTCGATCACTCGTCCCTTTCCATTTCGGCAGATAAGAAAATGACAGGTATAACTTAAAATAAGCTTAGATGGTGTGTATGTGTACCGGAATCAACACTATTGTAGATTTGCTGCAAGTGGCATTAACTCCTTAGCTAACTATGTAATACTTTATATATATATATATATATATATATATATATTGTATATTGTATATAGTGTGTATTTTTTATAAACCAATATAGAAAGTATATAATATGTTTATATATCTATGAAATATGTATTATATTATAATTTATTTAAATGTGGTTTGTAGGTTTAGGTTAATATTGTGTCTTATATTAGTATAAGTAATATCTAAATAATTTGTACGCGTATGTATGTCTATCTATATAATATTATACGTTGAAAGTCTCTCTTGTCACGTAGGTCAGCTGGAAGAAATCTCTTTGTTTAGAGATAAGCTTGCCTGTACTATCTGTTTTCTTTGTATGTTTCTTATGTCTGTTTCATTGTGTACAAATAAATAAATAAAAAAAAAAATACTTTAATGATCTCTCTCTCTTTTTATTTAAGAGCTGCGCTCTTGTCGGTGGAATAATCGCCATTCCTCTCTTCTTCCCGCCAAATCCTTCACCTCCTGATACAACACGACCTGCACCTTCTCTTTTATTTATTTCATAAATGTTCTCCTAGGTCTAGGTCCCTTCCTCTCTTCCCTTAAATTTTTTCTTCTATGATGTTTGTTATAAaagaatcgtgtcgtatcaggtggccaatcatatttcctctccggttctctatagtcttcattatggttctcttttcttcaactctttccagtactttttcaTttgacaccatttccttccagctttttacttattaatgatattaataaataaaaaaaccgaaacgatcctgttttatctaaaccgatttctttcttatatcttctatgtattttagccgcgcgtaagtccgtctgtagaagagaaTgtgcca
