# Supplementary material for: Alternative Splicing and Highly Variable Cadherin Transcripts Associated with Field-Evolved Resistance of Pink Bollworm to Bt Cotton in India
Source: PLoS One. 2014 May 19;9(5):e97900. doi: 10.1371/journal.pone.0097900 (PMC4026531; doi:10.1371/journal.pone.0097900)
Supplement: Table S1 — Pink bollworm from India screened for cadherin alleles r1-r3 from Arizona. (DOCX) [file pone.0097900.s009.docx]

Table S1. Pink bollworm from India screened for cadherin alleles *r1-r3* from Arizona.

| Collection site^a^ | GPS  coordinates | Collection date | Life stage | Source | n |
| --- | --- | --- | --- | --- | --- |
| Gujarat, Anand (AGJ) | 22° 32’ 23” N  72° 58’ 71” E | 01/2011 | 4^th^ instar | Bt cotton | 2 |
|  |  | 01/2011 | Pupa | Bt cotton | 7^b^ |
|  |  | 01/2011 | Adult | Bt cotton | 10^b^ |
| Gujarat, Rajkot | 22° 32’ 23” N  72° 58’ 71” E | 01/2011 | 2^nd^-4^th^ instar | Bt cotton | 27 |
| Haryana, Hisar | 29° 5’ 24” N  75° 25’ 48” E | 10/2010 | Adult | Pheromone trap | 91 |
| Madhya Pradesh, Khandwa (KMP) | 21° 49’ 48” N  76° 19’ 48’ E | 12/2010 | Adult | Pheromone trap | 15 |
|  |  | 12/2010 | Adult | Bt cotton | 50^b^ |
|  |  | 12/2010 | 4^th^ instar | Bt cotton | 38 |
| Maharashtra, Akola (AMH) | 20° 42’ 3” N  77° 15’ 13” E | 01/2011 | 4^th^ instar | Bt cotton | 24 |
|  |  | 12/2010 | Adult | Pheromone trap | 40 |
|  |  | 12/2010 | 4^th^ instar | Non-Bt cotton | 6 |
|  |  | 12/2010 | Adult | Non-Bt cotton & Bt cotton | 40^b^ |
|  |  | 01/2011 | Adult | Non-Bt cotton | 20^b^ |
|  |  | 01/2011 | 4^th^ instar | Non-Bt cotton | 5^c^ |
| Rajasthan, Banswara | 23° 33’ 0” N  74° 27’ 0” E | 11/2010 | Adult | Pheromone trap | 10 |
| Rajasthan, Sangaria | 29° 47’ 0” N  74° 28’ 0” E | 10/2010 | Adult | Pheromone trap | 40 |

^a^ State and nearest city from which pink bollworm collections were made. Abbreviations of select collection sites are provided in parenthesis.

^b^ Larvae collected from Bt cotton bolls and reared in laboratory on untreated diet.

^c^ Larvae collected from Bt cotton bolls and reared in laboratory on diet containing 10 micrograms Cry1Ac per mL diet.
