# Supplementary material for: Alternative Splicing and Highly Variable Cadherin Transcripts Associated with Field-Evolved Resistance of Pink Bollworm to Bt Cotton in India
Source: PLoS One. 2014 May 19;9(5):e97900. doi: 10.1371/journal.pone.0097900 (PMC4026531; doi:10.1371/journal.pone.0097900)
Supplement: Table S2 — gDNA sequencing of eight novel disrupted cadherin alleles in pink bollworm larvae from Anand (AGJ) in Gujarat and Khandwa (KMP) in Madhya Pradesh. (DOCX) [file pone.0097900.s010.docx]

Table S2. gDNA sequencing of eight novel disrupted cadherin alleles in pink bollworm larvae from Anand (AGJ) in Gujarat and Khandwa (KMP) in Madhya Pradesh.

| Allele | Iso-form | cDNA deletion  size (bp) | cDNA insertion  size (bp) | cDNA  exon(s) affected | gDNA  primers | gDNA fragment  size (bp) | Amplified gDNA region  intact^a^ |
| --- | --- | --- | --- | --- | --- | --- | --- |
| *r5* | *r5A* | 478^b^ | - | 21-24 | 20PgCad5  81PgCad3 | 3,827 | No |
| *r5* | *r5B* | 3, 478 | - | 1 | 227PgCad5  228PgCad3 | 781 | Yes |
| *r5* | *r5C* | 478 | 20^c^ | 5 | 89PgCad5  10PgCad3 | 1,380 | Yes^d^ |
| *r6* | *r6A* | 1051 | - | 8-13 | - | - | NA^e^ |
| *r7* | *r7A* | 247 | - | 21-22 | 20PgCad5  165PgCad3 | 2,515 | Yes |
| *r7* | *r7B* | 99 | - | 27 | 164PgCad5  163PgCad3 | 2,095 | Yes |
| *r8* | *r8A* | 170 | 4 | 13 | 186PgCad5  166PgCad3 | ~4000^f^ | Yes |
| *r8* | *r8B* | - | 4 | No | 219PgCad5  220PgCad3 | 1,042 | No |
| *r9* | *r9A* | 165 | - | 32 | 171PgCad5  25PgCad3 | 3,482 | Yes |
| *r9* | *r9B* | 1157 | - | 23-31 | 58PgCad5  87PgCad3 | ~7000^g^ | Yes |
| *r10* | *r10A*-*r10C* | 126 | - | 21 | 20PgCad5  21PgCad3 | 1,857 | Yes |
| *r10* | *r10A*-*r10C* | 105 | - | 25 | 169PgCad5  170PgCad3 | 1,715 | Yes |
| *r10* | *r10B* | 303 | - | 28-29 | 86PgCad5  167PgCad3 | 1,470 | Yes |
| *r10* | *r10C* | 193 | - | 28 | 24PgCad5  85PgCad3 | 2,004 | Yes |
| *r11* | *r11A* | 23 | 127 | No | 20PgCad5  49PgCad3 | 930 | Yes |
| *r11* | *r11B* | - | 125 | No | 171PgCad5  172PgCad3 | 697 | No |
| *r12* | *r12A* | - | 1 | No | 221PgCad5 222PgCad3 | 191 | No |
| *r12* | *r12B* | 118 | 1 | 11 | 168PgCad5  187PgCad3 | 2,647 | Yes |
| *r12* | *r12C, r12D* | 11 | 1 | 1 | 227PgCad5  228PgCad3 | 781 | Yes |
| *r12* | *r12C* | 148 | 1 | 5 | 89PgCad5  10PgCad3 | 1,380 | Yes |
| *r12* | *r12D* | 230 | 1 | 11-12 | 186PgCad5  73PgCad3 | 2,683 | Yes |

^a^ gDNA was intact in region amplified by indicated PCR primers

^b^ Mutations shown in bold text are the ones for which primers were used to amplify corresponding gDNA

^c^ 20-bp insertion in *r5B* corresponds to last 20 nucleotides from intron 4

^d^ While 20-bp insertion at start of exon 5 may result from aberrant mRNA splicing, the 478-bp deletion affecting exons 21-24 is due to 3,120-bp insert and not mis-splicing

^e^ gDNA was not sequenced for this allele

^f^  PCR product of approximately 4 kb was amplified using primers 186PgCad5 and 166PgCad3 and cloned. DNA sequencing confirmed the presence of exon 13 in gDNA thereby implicating mis-splicing of mRNA as source of 170-bp deletion in *r8A* (See Figure S8).

^g^ PCR product of approximately 7 kb was amplified using primers 58PgCad5 and 87PgCad3 and cloned. DNA sequencing confirmed the presence of exons 23-26 and 30-32 with corresponding introns. These results confirm that deletion of 1,157 bp (exons 23-31) in *r9B* was due to mis-splicing of mRNA (See Figure S8).
