# Supplementary material for: Alternative Splicing and Highly Variable Cadherin Transcripts Associated with Field-Evolved Resistance of Pink Bollworm to Bt Cotton in India
Source: PLoS One. 2014 May 19;9(5):e97900. doi: 10.1371/journal.pone.0097900 (PMC4026531; doi:10.1371/journal.pone.0097900)
Supplement: Table S3 — Nucleotide primers used to amplify and sequence PgCad1 from India pink bollworm. (DOCX) [file pone.0097900.s011.docx]

**Table S3. Nucleotide primers used to amplify and sequence *PgCad1* from India pink bollworm.**

| **Primer** | **Primer DNA Sequence** | **Direction** | **Application** |
| --- | --- | --- | --- |
| M13 reverse | 5'-CAGGAAACAGCTATGAC-3' | Sense | Vector primer for DNA sequencing |
| T7 | 5'-TAATACGACTCACTATAGGG-3' | Antisense | Vector primer for DNA sequencing |
| 52PgCad5 | 5'-ATGGCGGGTGACGCCTGCATAC-3' | Sense | Cloning cDNA & DNA sequencing |
| 25PgCad3 | 5'-CTATGGTCGCATGCGCCTGTTAGT-3' | Antisense | Cloning cDNA, PCR cloning of genomic fragments & DNA sequencing |
| 89PgCad5 | 5’-GGAACCCCAGAAATTGTCCAGCC-3' | Sense | DNA sequencing & PCR cloning of genomic fragments |
| 10PgCad3 | 5’-AAGATGTGCAGCGGGTTGCTTT-3’ | Antisense | DNA sequencing & PCR cloning of genomic fragments |
| 57PgCad3 | 5'-TCAGTCTGCCGACGTGATAG-3' | Antisense | DNA sequencing |
| 70PgCad5 | 5'-GACCGCCGCGATGGATGGAAAT-3' | Sense | DNA sequencing |
| 72PgCad5 | 5'-GACTGTACCCAAGGACTATCACGTCGG-3' | Sense | DNA sequencing |
| 73PgCad3 | 5'-TGGCACATTCTCTTCTACAGACGGACT-3' | Antisense | DNA sequencing |
| 75PgCad3 | 5'-ATGTCTGTAGCGAGTACGGACCCG-3' | Antisense | DNA sequencing |
| 76PgCad5 | 5'-CTGAACCAGACCTTCAGTATTCGGGAG-3' | Sense | DNA sequencing |
| 77PgCad3 | 5'-CACGTTTGTGAAATCGGGTGCATT-3' | Antisense | DNA sequencing |
| 78PgCad5 | 5'-AAATGCACCCGATTTCACAAACGTG-3' | Sense | DNA sequencing |
| 79PgCad3 | 5'-TTAGGCGACAGCATGTTGAGAAGTCTC-3' | Antisense | DNA sequencing |
| 20PgCad5 | 5'-ATGCTGTCGCCTAACAACGTAAC-3' | Sense | DNA sequencing & PCR cloning of genomic fragments |
| 49PgCad3 | 5'-ATATCTGCTGAGGAGTACCGTGGTCGAA-3' | Antisense | PCR cloning of genomic fragments |
| 81PgCad3 | 5'-GGAGCGAGAACCTCTCAGTCAAGCC-3' | Antisense | DNA sequencing & PCR cloning of genomic fragments |
| 85PgCad3 | 5'-GAAGGACACCCTATTTTGGGAT-3' | Antisense | DNA sequencing & PCR cloning of genomic fragments |
| 58PgCad5 | 5'-TGGAACCTTGTTGCTGACAC-3' | Sense | PCR cloning of genomic fragments |
| 86PgCad5 | 5'-TTCATCCCAAAATAGGGTGTCCTTCAT-3' | Sense | PCR cloning of genomic fragments |
| 87PgCad3 | 5'-TGTTGGTTCCTGGGGCCGCTAT-3' | Antisense | DNA sequencing & PCR cloning of genomic fragments |
| 165PgCad3 | 5'-GTGTCAGCAACAAGGTTCCAAA-3' | Antisense | PCR cloning of genomic fragments |
| 164PgCad5 | 5'-GCAAGTGGCAGCCAGTAATTC-3' | Sense | PCR cloning of genomic fragments |
| 163PgCad3 | 5'-CCATAGAACCGTCTTCGATGGTA-3' | Antisense | DNA sequencing & PCR cloning of genomic fragments |
| 24PgCad5 | 5'-ACGGCTGGCATTTCCACTTC-3' | Sense | PCR cloning of genomic fragments |
| 167PgCad3 | 5'-CCGGCAGCACCTGATCTATATT-3' | Antisense | PCR cloning of genomic fragments |
| 21PgCad3 | 5'-GTCGTCAATTGCTTGTATCCTGTC-3' | Antisense | PCR cloning of genomic fragments |
| 169PgCad5 | 5'-ACTTTGGGTGATCCGATCTTTC-3' | Sense | PCR cloning of genomic fragments |
| 166PgCad3 | 5'-GGGTTTGGGAAGATGGTTTCTA-3' | Antisense | PCR cloning of genomic fragments |
| 170PgCad3 | 5'-CAGTGGTCTCCCGGTCTAGTTC-3' | Antisense | DNA sequencing & PCR cloning of genomic fragments |
| 171PgCad5 | 5'-TCCATGCTCACTTCATACAGGA-3' | Sense | DNA sequencing & PCR cloning of genomic fragments |
| 172PgCad3 | 5'-AGCAGAATAAGGCACAGGAAGG-3' | Antisense | PCR cloning of genomic fragments |
| 168PgCad5 | 5'-ACCAGCTCGTTGTCAAGTTCAA-3' | Sense | PCR cloning of genomic fragments |
| 187PgCad3 | 5'-ATTCGCATTTCCCAAGATGG-3' | Antisense | DNA sequencing & PCR cloning of genomic fragments |
| 186PgCad5 | 5'-AGCTATTGATGACGCGTTCG-3' | Sense | DNA sequencing & PCR cloning of genomic fragments |
| 173PgCad5 | 5'-GTGCGAATGAGTCCGAAGTT-3' | Sense | DNA sequencing of genomic fragment |
| 174PgCad5 | 5'-GACTTGAACTCGTGGAACGAC-3' | Sense | DNA sequencing of genomic fragment |
| 175PgCad3 | 5'-ATGTTTGTGTGTGCGTTTGTGC-3' | Antisense | DNA sequencing of genomic fragment |
| 176PgCad3 | 5'-AACGGGTTTGTGTTTGATGAGTTC-3' | Antisense | DNA sequencing of genomic fragment |
| 181PgCad5 | 5'-CTGAATTTGCTCGCGAACTG-3' | Sense | DNA sequencing of genomic fragment |
| 182PgCad3 | 5'-GAGCAGCGTGGTGGAGTATG-3' | Antisense | DNA sequencing of genomic fragment |
| 183PgCad3 | 5’-TGTGTGCGTTTGTGCAAAGT-3' | Antisense | DNA sequencing of genomic fragment |
| 22PgCad5 | 5'-TTTGAGAGGATATTGGGGGACTTA-3' | Sense | DNA sequencing of genomic fragment |
| 50PgCad3 | 5'-CTGCTGAGGAATACCGTGTGC-3' | Antisense | DNA sequencing of genomic fragment |
| 23PgCad3 | 5'-TCACCGTTTACAAGTGACAAAACA-3' | Antisense | DNA sequencing of genomic fragment |
| 84PgCad5 | 5'-CTGTGTTCGAGCAGCGTCTGTA-3' | Sense | DNA sequencing of genomic fragment |
| 83PgCad3 | 5'-TGTACAGACGCTGCTCGAACACAGG-3' | Antisense | DNA sequencing of genomic fragment |
| 54PgCad5 | 5’-CTCATGAGGTGTATGAATTGGAA-3' | Sense | DNA sequencing of genomic fragment |
| 188PgCad3 | 5'-CCCCAGACTGCGCACTTTAT-3' | Antisense | DNA sequencing of genomic fragment |
| 184PgCad5 | 5'-TCGAACGGAAGTTGTGCATC-3' | Sense | DNA sequencing of genomic fragment |
| 185PgCad3 | 5'-GGCGGGAAGAAGAGAGGAAT-3' | Antisense | DNA sequencing of genomic fragment |
| 162PgCad5 | 5'-GGTTCTATGGCGGTGGACTC-3' | Sense | DNA sequencing of genomic fragment |
| 163PgCad3 | 5'-CCATAGAACCGTCTTCGATGGTA-3' | Antisense | DNA sequencing of genomic fragment |
| 82PgCad5 | 5'-AAAGAGGTTGGCTTGACTGAGAGGTTC-3' | Sense | DNA sequencing of genomic fragment |
| 177PgCad5 | 5'-CCCCTTTCTCCCTTTCCTTC-3' | Sense | DNA sequencing of genomic fragment |
| 178PgCad3 | 5'-TATAGCGCACGCCTCTCTGA-3' | Antisense | DNA sequencing of genomic fragment |
| 179PgCad5 | 5'-CGTGACAAAATGTCGCATTCA-3' | Sense | DNA sequencing of genomic fragment |
| 180PgCad3 | 5'-TCTGTCTGACGGGAGTGGAA-3' | Antisense | DNA sequencing of genomic fragment |
| 189PgCad5 | 5'-TCTGGGTGTGTTCCTGACAT-3' | Sense | DNA sequencing of genomic fragment |
| 190PgCad3 | 5'-ATGTCAGGAACACACCCAGA-3' | Antisense | DNA sequencing of genomic fragment |
| 191PgCad5 | 5'-TTTAAATGTGGTTTGTAGGTTTAGGTT-3' | Sense | DNA sequencing of genomic fragment |
| 192PgCad3 | 5'-TTAACCTAAACCTACAAACCACATTT-3' | Antisense | DNA sequencing of genomic fragment |
| 193PgCad5 | 5'-CACCATTGGCTTTTGTCGAA-3' | Sense | DNA sequencing of genomic fragment |
| 194PgCad3 | 5'-GACGCCTTACTGACTCGCAAC-3' | Antisense | DNA sequencing of genomic fragment |
| 223PgCad5 | 5'-ATTCGGGCAGGGACTTTAAC-3' | Sense | DNA sequencing of genomic fragment |
| 224PgCad3 | 5'-ACCCTGTGTGAAACGGGATA-3' | Antisense | DNA sequencing of genomic fragment |
| 225PgCad5 | 5'-CATTGCTATTGGATTTTCTGCAT-3' | Sense | DNA sequencing of genomic fragment |
| 226PgCad3 | 5'-GGCGCCCAGAGAAGTCTATT-3' | Antisense | DNA sequencing of genomic fragment |
| 219PgCad5 | 5'-CATTCCATCTTGGGAAATGC-3' | Sense | PCR cloning of genomic fragments |
| 220PgCad3 | 5'-TATGACCAGCTGAGCAGTCG-3' | Antisense | PCR cloning of genomic fragments |
| 221PgCad5 | 5'-ACTCATTCCGAAAACGCACA-3' | Sense | PCR cloning of genomic fragments |
| 222PgCad3 | 5'-CTGGATCAGTAGCGATGACG-3' | Antisense | PCR cloning of genomic fragments |
| 227PgCad5 | 5'-TACTGGTGACGGTGCTTCTG-3' | Sense | PCR cloning of genomic fragments |
| 228PgCad3 | 5'-AATCATCCGGTTTCGGTTCT-3' | Antisense | PCR cloning of genomic fragments |
| 229PgCad5 | 5'-AATCTTCGCAGTCCAGCAGT-3' | Sense | PCR cloning of genomic fragments |
| 230PgCad3 | 5'-TACTCATCTTGCAGCGGTTG-3' | Antisense | PCR cloning of genomic fragments |
